# Mapping the virome in wild-caught *Aedes aegypti* from Cairns and Bangkok

**DOI:** 10.1038/s41598-018-22945-y

**Published:** 2018-03-16

**Authors:** Martha Zakrzewski, Gordana Rašić, Jonathan Darbro, Lutz Krause, Yee S. Poo, Igor Filipović, Rhys Parry, Sassan Asgari, Greg Devine, Andreas Suhrbier

**Affiliations:** 1 Medical Genomics, QIMR Berghofer Medical Research Institute, Brisbane, Qld 4029 Australia; 2 Mosquito Control, QIMR Berghofer Medical Research Institute, Brisbane, Qld 4029 Australia; 3 Inflammation Biology, QIMR Berghofer Medical Research Institute, Brisbane, Qld 4029 Australia; 4 School of Biological Sciences, The University of Queensland, Brisbane, QLD 4029 Australia; 5 Present Address: Metro North Public Health Unit, Bryden Street, Windsor, QLD 4030 Australia; 6 Present Address: The University of Queensland Diamantina Institute, Brisbane, QLD Australia

## Abstract

Medically important arboviruses such as dengue, Zika, and chikungunya viruses are primarily transmitted by the globally distributed mosquito *Aedes aegypti*. Increasing evidence suggests that transmission can be influenced by mosquito viromes. Herein RNA-Seq was used to characterize RNA metaviromes of wild-caught *Ae*. *aegypti* from Bangkok (Thailand) and from Cairns (Australia). The two mosquito populations showed a high degree of similarity in their viromes. BLAST searches of assembled contigs suggest up to 27 insect-specific viruses may infect *Ae*. *aegypti*, with up to 23 of these currently uncharacterized and up to 16 infecting mosquitoes from both Cairns and Bangkok. Three characterized viruses dominated, Phasi Charoen-like virus, Humaita-Tubiacanga virus and Cell fusing agent virus, and comparisons with other available RNA-Seq datasets suggested infection levels with these viruses may vary in laboratory-reared mosquitoes. As expected, mosquitoes from Bangkok showed higher mitochondrial diversity and carried alleles associated with knock-down resistance to pyrethroids. Blood meal reads primarily mapped to human genes, with a small number also showing homology with rat/mouse and dog genes. These results highlight the wide spectrum of data that can be obtained from such RNA-Seq analyses, and suggests differing viromes may need to be considered in arbovirus vector competence studies.

## Introduction

In recent years metagenomics (the study of genetic content of entire communities recovered directly from environmental samples) has provided new insights into the substantial complexity and diversity of RNA viruses of invertebrates^1^. Of particular interest to arbovirology is the identification of an increasing number of insect-specific viruses (ISVs) that infect mosquitoes, but that are unable to infect vertebrates^2,3^. Several ISVs have been shown to suppress (or enhance^4^) replication of medically important arboviruses such as dengue, West Nile and chikungunya viruses, suggesting they play an important role in modulating vector competence^5–9^.

The mosquito *Aedes aegypti* is a primary vector for transmission of a number of arboviruses such as dengue, Zika, and chikungunya viruses that infect tens of millions of people in tropical and subtropical regions around the globe every year. Herein we use a metagenomic approach to describe the virome of two wild-caught, geographically distant *Ae*. *aegypti* populations, one from Cairns (Australia) collected in 2014 and one from Bangkok (Thailand) collected in 2015. Both mosquito populations are associated with dengue transmission. The Cairns mosquitoes were also collected near the sites of release of *Wolbachia*-infected *Ae*. *aegypti* mosquitoes, which was undertaken in 2012–13 as a control measure to suppress dengue transmission^10,11^.

In order to identify potential new mosquito-associated viruses (including those with no DNA intermediates), we undertook RNA sequencing of total RNA isolated from pooled mosquitoes caught in the wild. The study highlights the range of data that can be obtained from such analyses, and provides an initial map of the diversity and abundance of ISV infections in wild-caught *Ae*. *aegypti*. While the viromes of wild-caught *Ae*. *aegypti* from Cairns and Bangkok were surprisingly similar, analyses of other available RNA-Seq data sets suggested that the levels of major ISVs in laboratory reared mosquitoes may be different.

## Results

### RNA-Seq of pooled wild-caught mosquitoes

**Figure 1:**
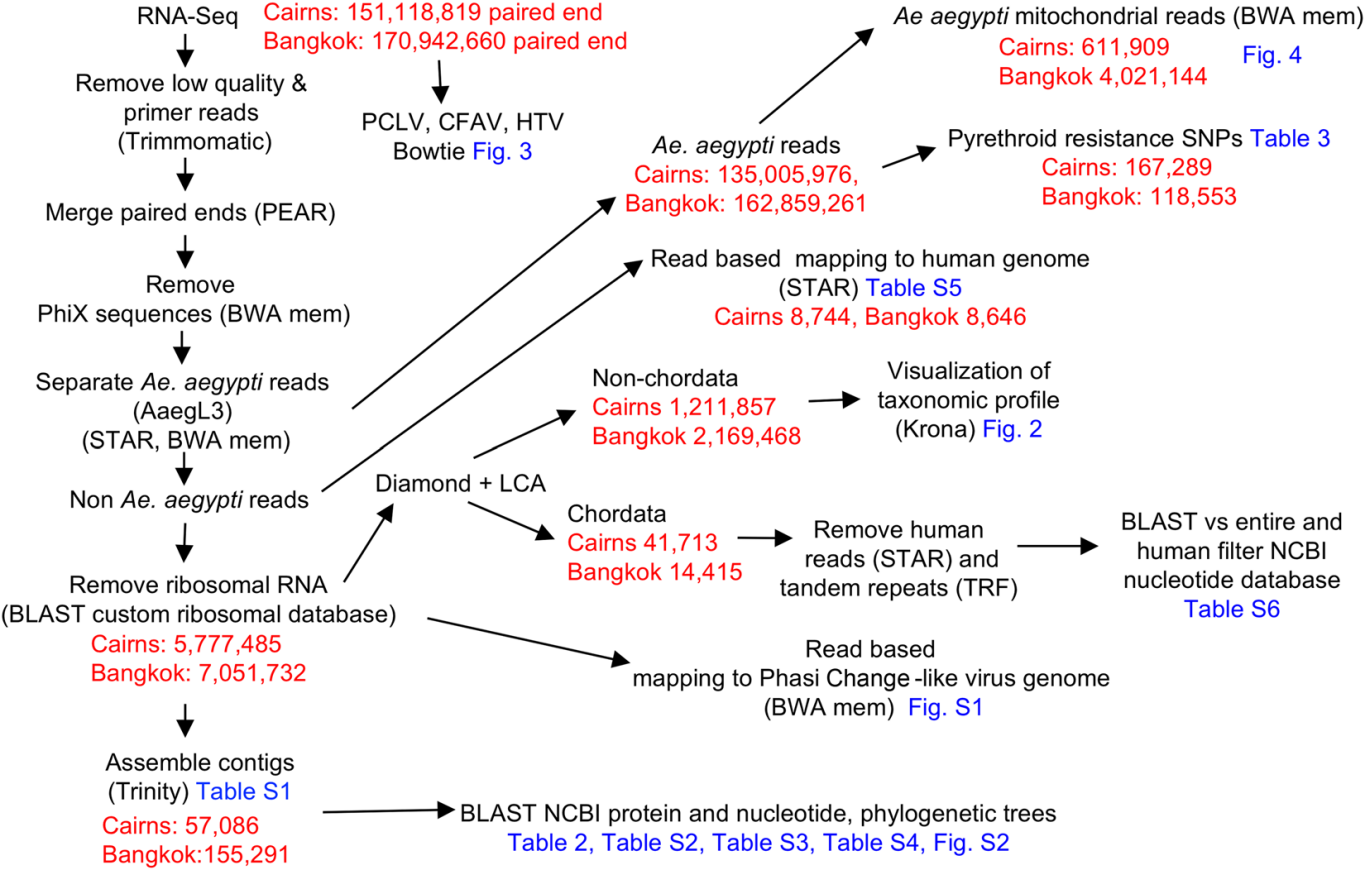
Bioinformatics work-flow. The bioinformatics work-flow is shown with read numbers and where the final data is presented (blue). Raw sequence reads were quality filtered using Trimmomatic and paired end reads were merged using the software PEAR. *Ae*. *aegypti* reads were identified using STAR and BWA mem and used for variant analyses. Non-mosquito reads were subsequently aligned to a custom ribosomal database using BLAST. Reads not matching a ribosomal reference sequence were taxonomically assigned using DIAMOND and the lowest common ancestor (LCA) approach. Non-chordate sequences were analyzed using BLAST to identify blood meal species. Contigs assembled with Trinity from non-mosquito and non-ribosomal reads were taxonomically assigned using the best BLAST hit.

Pools of 150–200 *Ae*. *aegypti* mosquitoes were collected from Cairns and Bangkok and were independently processed and subjected to RNA-Seq analysis in 2014 and 2015, respectively, using the Illumina HiSeq. 2500 platform. For the Cairns mosquito pool ≈151 million paired end sequence reads were generated providing 45.34 Gb of data. For the Bangkok mosquito pool ≈170 million paired end reads were generated providing 51.28 Gb of data. The bioinformatics work-flow of the RNA-Seq-derived sequences is described in Fig. 1.

### Phasi Charoen-like, Cell fusion agent, and Humaita-Tubiacanga viruses

**Figure 2:**
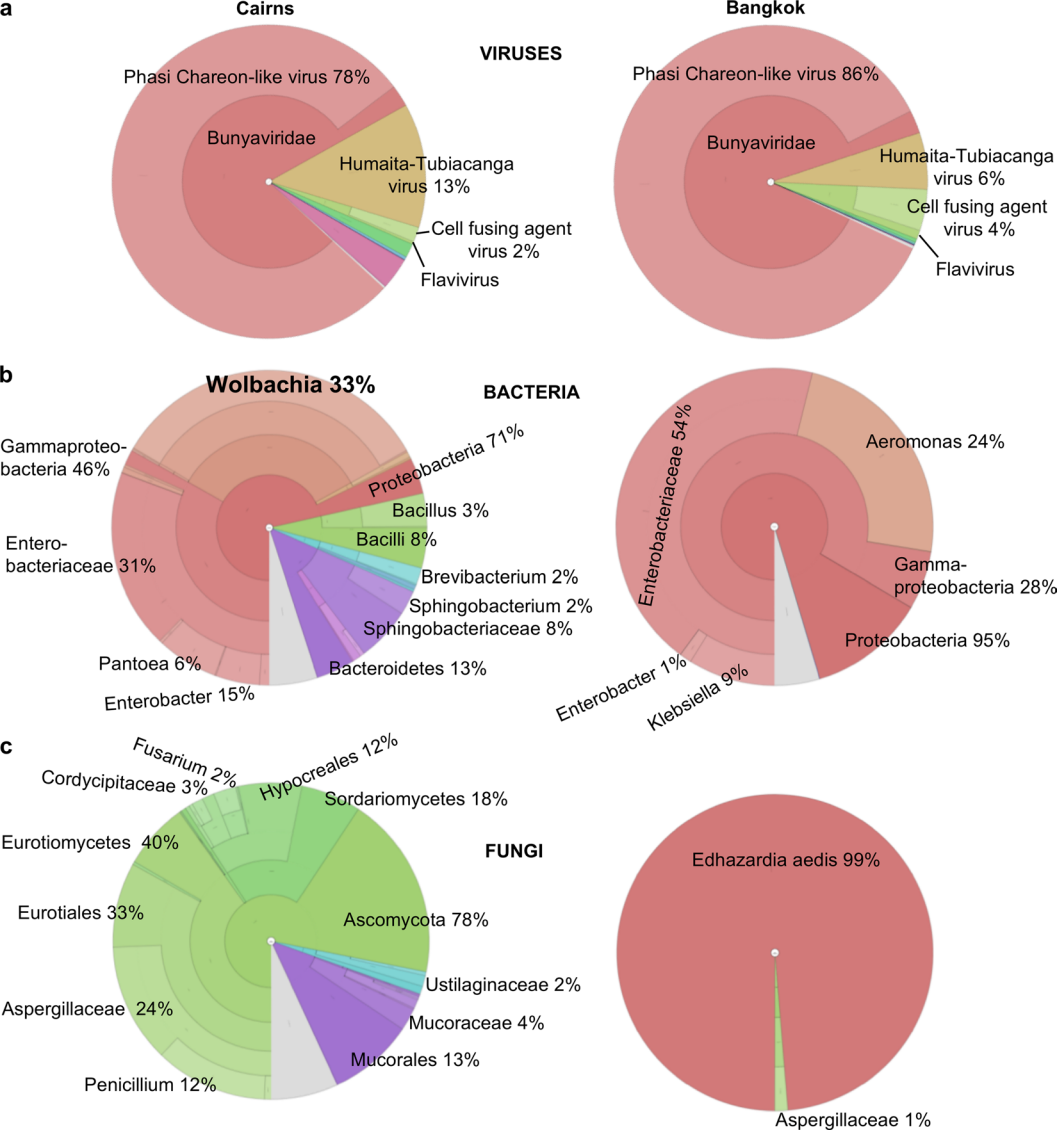
Overview of main ISVs, bacteria and fungi. Read based taxonomic classification using DIAMOND (NCBI protein database, 18/03/16) and the Lowest Common Ancestor (LCA) approach, with results visualized by Krona. (**a**) Viruses. Percentages of all viral reads (Cairns 959,502, Bangkok 2,074,841) aligning to the indicated virus species. For Cairns 697,301 reads, and for Bangkok 1,711,929 reads, aligned to Phasi Charoen-like virus. (**b**) Bacteria. Percentages of all bacterial reads aligning to the indicated genera or family. For Cairns, 31,587 reads aligned to the genus *Wolbachia*. (**c**) Fungi. Percentages of all fungal reads aligning with the indicated genera, family or species; 107,802 reads aligned to the phylum *Ascomycota* for Cairns and 47,038 reads aligned to *Edhazardia aedis* for Bangkok.

After removal of mosquito and ribosomal sequences, the remaining reads (Fig. 1; 1,211,857 for Cairns and 2,169,468 for Bangkok) were taxonomically assigned using DIAMOND (NCBI protein data base) and the Lowest Common Ancestor (LCA) approach. An overview of the metaviromes (visualized using Krona^12^) is shown in Fig. 2a. The similarity of viral sequences contrasted with the substantial differences in bacterial (Fig. 2b) and fungal (Fig. 2c) sequences (described below) obtained from the two mosquito samples.

The metaviromes (Fig. 2a) were dominated by three known ISVs that have previously been identified in *Ae*. *aegypti* (although not in Australian *Ae*. *aegypti*): (i) Phasi Charoen-like virus (PCLV), a phlebovirus (family *Bunyaviridae*) previously shown to be abundant in Thai mosquitoes^13^ (Cairns 99.3% and Bangkok 100% of the genome covered with mean read depth of 7311 and 35,248, respectively); (ii) Humaita-Tubiacanga virus (HTV), an unclassified virus identified in *Ae*. *aegypti* from Brazil^14^ (Cairns 99.6% and Bangkok 99.3% of the genome covered with mean read depth of 9383 and 11,088, respectively) and (iii) Cell fusing agent virus (CFAV), a flavivirus previously found in Thai and American *Ae*. *aegypti*^15,16^ (Cairns 96.5% and Bangkok 98% of the genome covered with mean read depth of 327 and 2,483, respectively).

The high read coverage for PCLV across the genome (Fig. S1a) allowed the identification of single nucleotide polymorphisms (SNPs). PCLV genomes from Bangkok mosquitoes showed a higher level of genetic diversity than those from Cairns mosquitoes (Fig. S1b), suggesting a greater level of virus swarm complexity or quasispecies^17^ in the former. This increased ISV diversity is perhaps consistent with the higher genetic diversity seen in the host *Ae*. *aegypti* population from Bangkok (see below). A similar association was suggested for dengue virus and *Ae*. *aegypti*^18^, perhaps arguing that both ISVs and arboviruses evolve in response to the genotype of their mosquito hosts.

### RNA-Seq data from laboratory mosquitoes

**Figure 3:**
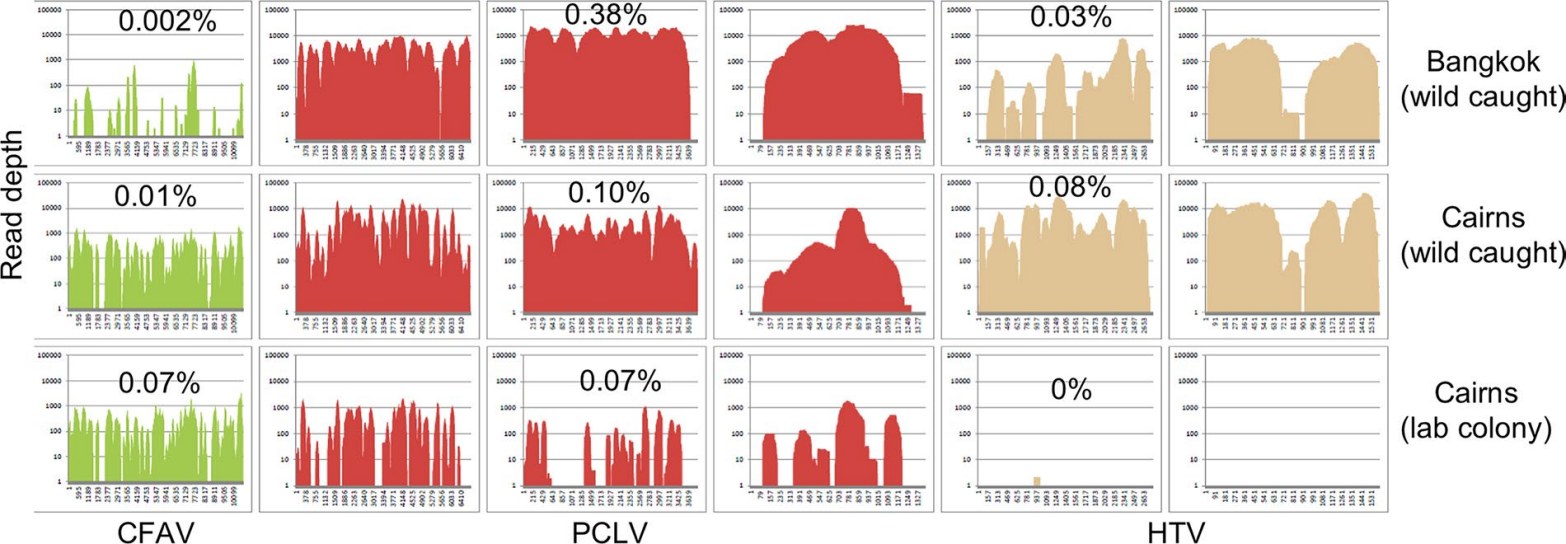
Reads mapping to CFAV, PCLV and HTV. RNA-Seq reads (generated without polyA enrichment) from (i) wild-caught Bangkok and Cairns *Ae*. *aegypti* and (ii) *Ae*. *aegypti* originally from Cairns that had been reared in the laboratory for ≈2 years, were mapped (using Bowtie allowing a 5% mismatch) to the genomes of CFAV, PCLV and HTV (see Methods for accession numbers). The y-axis (1 to 100,000) represents the number of reads that cover each position in the genome. The x-axis is the genome position, with the entire genome for each virus represented. The percentages represent viral reads as a percent of *Ae*. *aegypti* reads.

We obtained access to RNA-Seq data (65,273,690 reads) from a colony established from mosquitoes collected in Cairns in March 2014. RNA-Seq was performed on a pool of ≈25, 12 day old, sugar fed, *Wolbachia*-negative *Ae*. *aegypti* in May 2016 (Asgari *et al*. unpublished). Importantly, (as herein) a polyA enrichment step was not included prior to library preparation and sequencing was undertaken using the same Illumina platform at the Australian Genome Research Facility. The number of reads mapping to the indicated ISV, expressed as a percentage of reads mapping to the *Ae*. *aegypti* genome in the same data set was calculated. This provides a nominal approximation of the level of infection with the indicated ISV within each mosquito population. Although, there was some variation in ISV infection levels, the standout result was that only 2 reads from the Cairns laboratory colony aligned to HTV (Fig. 3), whereas 33,602 reads mapped to CFAV and 35,134 reads mapped to PCLV. HTV infection levels in these laboratory reared mosquitoes were thus very much lower than those found in wild-caught mosquitoes.

RNA-Seq studies of pooled *Ae*. *aegypti* from other laboratory mosquito colonies are also available from NCBI Sequence Read Archive^19^ and were aligned to PCLV, HTV and CFAV genomes. These RNA-Seq analyses were performed using a different methodology (e.g. 75 instead of 100 base pair reads) and, importantly, they included a mRNA enrichment step, whereby polyadenylated RNA was enriched prior to generation of libraries^19^. Such enrichment would substantially reduce PCLV, HTV, CFAV RNA levels, as the RNA from such viruses are generally not polyadenylated. Nevertheless, reads to all three of these viruses were identified, albeit at low levels (Table 1). Interestingly, there were no reads mapping to PCLV and HTV in 4 out of the 7 colonies (Table 1, StGeR, LivpS, DeltaR, NwOrS) and no reads mapping to CFAV in 4 different colonies (Table 1, NakhRc, CaynR, StGeR, NwOrS). Some of these colonies had been in the laboratory for only 2 generations (Tables 1, 2 gens), whereas others had been reared in the laboratory for many years (Table 1). These data (together with Fig. 3) suggest that the virome or virus abundance (at least with respect to these 3 dominant viruses) may vary in different mosquito populations and/or may change when mosquitoes are reared in the laboratory.

**Table 1:** Reads aligning to PCLV, HTV and CFAV in publicly available RNA-Seq data sets.

Mosquito population	In lab.	Total reads, millions	Phasi Charoen-like virus		Humaita-Tubiacanga virus		Cell fusing agent virus	
			Reads mapped	% of total	Reads mapped	% of total	Reads mapped	% of total
PhetR	2 gens	229 m	1,869	0.0008	1,194	0.0005	534	0.0002
NakhRc	2 gens	254 m	889	0.0003	674	0.0003	ND	0
CaynR	2 gens	241 m	1,653	0.0007	169	0.0001	ND	0
StGeR	2 gens	238 m	ND	0	ND	0	ND	0
LivpS	1930s	284 m	ND	0	ND	0	393	0.0001
DeltaR	1990s	197 m	ND	0	ND	0	389	0.0002
NwOrS	1980s	167 m	ND	0	ND	0	ND	0
Aag2 cell line		22 m	196	0.0009	ND	0	3,563	0.0159

RNA-Seq data (generated with a polyA enrichment step) from pools of ≈90 mosquitoes per pool and Aag2 cell line^19^ were obtained from NCBI, accession numbers (top to bottom) ERX1788144-6, ERX1788141-3, ERX1788135-7, ERX1788138-40, ERX1788126-8, ERX1788132-4, ERX1788129-31, Aag2 - SRX1367297, SRX1366586. Using BWA mem these were aligned to CFAV, PCLV and HTV genomes. A small number of reads mapped to Tongilchon virus 1^70^; 6 for LivpS and 7 for NwOrS. Colony identifiers: PhetR - Thailand (Phetchaburi); NakhRc - Thailand (Nakonsawan); CaynR - French Guiana (Cayenne); StGeR - French Guiana (St-Georges); LivpS - Liverpool strain (Benin); DeltaR - French Polynesia (Bora-Bora); NwOrS - USA (New Orleans). ND – not detected, no reads mapping. In lab: 2 gens - mosquitoes reared for 2 generations in the laboratory; 1930 s - mosquitoes introduced to the lab in the 1930 s.

**Table 2:** Summary of contig-based virus identification.

Classification	Virus or *nearest match*	Host	Genes	Range of % acid identity amino	
Known ISVs of *Ae aegypti*				CAIRNS	BANGKOK
Genus; *Phlebovirus* ssRNA(−)	Phasi Charoen-like virus	*Ae aegypti*	Nucleocapsid, Glycoprotein, RdRp	97–98	54–100
Genus; *Flavivirus* ssRNA(+)	Cell fusing agent virus	*Ae aegypti*	Polyprotein	99	98
Unclassified ssRNA(+)	Humaita-Tubiacanga virus	*Ae aegypti*	Capsid Replicase	97–98	97–98
Unclassified dsRNA (possibly a Totivirus)	Unnamed	*Ae aegypti* Thailand	PArp-RdRp, partial (Contig 5425)	95–98	91–99
Subfamily: *Densovirinae* ssDNA	*Aedes aegyti* *densovirus 2*	*Ae aegypti*	Non-structural protein 1 & capsid	NI	98
**Mosquito-associated viruses**					
Genus; *Flavivirus*	*Xishuangbanna flavivirus*	*Ae albopictus*	RdRp Polyprotein	60	59–67
Genus: *Orthomyxovirus* ssRNA(−)	*Whidbey virus*	*Aedes dorsalis*	PB1, PA, PB2	76–85	76–85
Unclassified. Order; *Mononegavirales* ssRNA(−)	*Xincheng mosquito Virus*	*Anopheles sinensis*	Glycoprotein	37–59	34–39
Family; *Bunyaviridae* Phasmavirus like. ssRNA(−)	*Wuhan mosquito virus* 2	*Culex*, *Anopheles*, *Aedes sp*	Glycoprotein precursor	34–44	42
Family *Rhabdoviridae* ssRNA(−)	*Tongilchonvirus* 1	*Culex*, *Anopheles*, *Aedes sp*	Glycoprotein precursor	NI	57
Unclassified; *Rhabdoviridae* ssRNA(−)	*Wuhan mosquito virus* 9	*Culex*, *Aedes sp*	Glycoprotein ORF1	28–37	30–37
Genus; *Totivirus* dsRNA	*Anopheles totivirus*	*Anopheles gambiae*	RdRp Putative capsid	27–45	28–45
Unclassified	*Kaiowa virus*	*Brazilian mosquitoes*	Putative glycoprotein	75	73
Unclassified	*Hubei toti-like virus10*	Mosquito	Hypothetical protein 1	39–46	Reads map
Unclassified	*dsRNA virus environmental sample*	*Ochlero-tatus sierrensis*	Proline-alanine-rich protein	37	NI
Oribivirus	*Unnamed*	*Ochlerotatus caspius & detritus*	RdRp AGW51764.1	87	NI
Unclassified virus	*Croada virus*	*Psorophora pools*	Putative glycoprotein	Reads map	73
Unclassified RNA virus	*Wenzhou sobemo-like virus* 4	Mosquito	Hypothetical protein	NI	68
Unclassified virus	*Hubei mosquito virus* 2	Mosquito	Hypothetical protein	NI	84
Family; *Chuviridae* ssRNA(−)	*Chuvirus Mos8Chu0*	*Culiseta minnesotae*	Putative nucleoprotein	Reads map	48
**Other insect-associated viruses**					
Unclassified	*Blackford virus*	*D*. *tristis*	Putative polyprotein	44–48	45–47
Unclassified RNA virus	*Hubei tombus-like virus* 40	*Coleoptera*	Hypothetical proteins	51	NI
Unclassified	*Diaphorina citri associated C virus*	Psyllid	RdRp	34	NI
Family: *Rhabdoviridae* ssRNA(−)	*Wuhan ant virus*	*Camponotus japonicus*	RdRp	32–53	NI
Family*; Baculoviridae* dsDNA	*Autographa californica nucleo-polyhedrovirus*	*Spodoptera frugiperda*	ORF B, AcOrf-4 & AcOrf-5 peptides	69	56–100
Unclassified	*Chaq virus*	Pachypsylla psyllid	Orf1	NI	38–59
Unclassified RNA	*Hubei virga-like virus* 12	*Dipteria*	Hypothetical protein	NI	31
**Other**					
Unclassified RNA viruses	*Beihai barnacle virus* 12	*Barnacle*	RdRp	NI	44
**Plant viruses**					
Unclassified phlebovirus-like	*Citrus concave gum-associated virus*	*Citrus sinensis*	RdRp	45	NI
Genus: *Closterovirus*	*Carrot closterovirus*	Carrot	ORF2	36	NI
*Phenuiviridae ssRNA(−)*	*Watermelon crinkle leaf-associated virus* 2	*Citrullus lanatus*	Nucleocapsid Polymerase	35–49	NI
Genus *Tobamovirus* ssRNA(+)	*Cucumber fruit mottle mosaic virus*	Cucumber	Unnamed protein	NI	28

Contigs were assembled from reads obtained from sequencing Cairns and Bangkok *Ae. aegypti* and were used for virus identification using the NCBI protein database. The range of percentage amino acid identities for one or more contigs is shown. Underlining (first column) highlights viruses or nearest relatives (italics), which appear to be unique to Cairns or Bangkok mosquitoes. Grey shading (NI - Not Identified) indicates where no contigs or reads matching the contig for the indicated virus were identified. “Reads map” indicates where no contigs were assembled, but reads from Cairns did map to the contig assembled from the Bangkok data or *vice verse*. The full data set, including contig information and references, is shown in Tables S2a and S2b.

Publicly available RNA-Seq data from the Aag2 cell line was also analyzed and abundant reads mapped to CFAV (Table 1). This was expected as CFAV was first isolated from this cell line^20^. Reads also mapped to PCLV (Table 1), with this virus also previously reported in Aag2 cells^21^.

### Contig assembly and identification of new viruses

To find evidence for potential new viruses, contigs were independently assembled from Cairns and Bangkok sequence reads (Table S1) using Trinity and compared to NCBI protein and nucleotide databases using BLAST (Fig. 1). As expected from the read-based analysis (Fig. 2a), a number of contigs from both Cairns and Bangkok mosquitoes aligned (with high amino acid sequence identity) to PCLV, CFAV, and HTV sequences (a summary is provided in Table 2, with full details in Table S2). A contig from Bangkok *Ae*.*aegypti* also aligned with 98% amino acid sequence identity to *Ae*. *aegypti* densovirus 2, a DNA virus previously identified in *Ae*. *aegypti* from India^22^ (Tables 2, S2). A number of contigs also aligned with high homology to an unnamed putative virus previously identified by contig assembly in *Ae*. *aegypti* from Thailand (Tables 2, S2). This virus is probably a totivirus as it shows homology to Hubei toti-like virus 10 and forms a cluster with toti-like viruses in the phylogenetic tree analysis (Fig. S2), with an *Anopheles* totivirus having been described previously^23^.

Some Endogenous Viral Elements (EVEs) sequences were removed to generate the non *Ae*. *aegypti* reads (Fig. 1); for instance, reads mapping to Liao Ning virus were removed^24^. However, a number of contigs aligned with high nucleotide identity to other known *Ae*. *aegypti* EVEs^25^ (Table S3). Contigs showing homology with flavivirus genomes, but containing multiple stop codons, were also classified as EVEs (Table S3). Although we have tried to remove EVEs, we cannot formally exclude the possibility that other contigs are derived from EVEs^26^. Sequences from persistent viral cDNA arising from mosquito reverse-transcriptase activity^27^ would likely not be efficiently represented in our data, as purified RNA was used for the RNA-Seq analyses.

The remaining contigs showed much less homology to known viruses (or EVEs), and closest relatives were identified by BLAST searches. The power of the approach is somewhat validated in that for most of these contigs the nearest matches where ISVs (Table 2 and S2). The results were often similar in both mosquito populations, with many contigs from both populations containing homologous overlapping nucleotide sequences (Table S2, overlapping contigs are shaded in grey). The high sequence similarities between shared ISVs from Cairns and Bangkok were also clearly evident when phylogenetic trees were generated from the protein sequences of the RNA-dependent RNA polymerases (RdRps) encoded by the assembled contigs (Fig. S2).

The contig analyses suggested there are up to 5 unique ISVs in Cairns mosquitoes (Tables 2, S2a underlined) and up to 6 unique ISVs in Bangkok mosquitoes (Tables 2, S2b underlined). In some cases, Bangkok reads aligned to contigs assembled from the Cairns data, and *vice versa*. For instance, although no contigs showing homology to Chuvirus Mos8Chu0 were assembled from Cairns reads, a number of Cairns reads did map (Table 2, Reads map) to the contig assembled from Bangkok reads that showed homology to Chuvirus Mos8Chu0 (Table S2, c914_g1_il). An ISV showing homology to Chuvirus Mos8Chu0 thus appears to be present in both Cairns and Bangkok mosquitoes. Where an ISV is likely present in both mosquito populations, the virus is not underlined in the Classification column in Tables 2 and S2. The analysis suggested up to 23 new uncharacterized ISVs are present in *Ae*. *aegypti* populations. Both mosquito populations also appear to share infection with up to 16 different ISVs (4 previously described and 12 uncharacterized) (summarized in Table 2). It should be noted that many of these ISVs have multiple genome segments and homology was often quite low, making it challenging to confidently ascribe contigs to distinct putative new ISVs. However, the phylogentic tree analysis (Fig. S2) does confirm (at least for those ISVs where RdRp references sequences are available) that two related contigs have not been ascribed to 2 distinct new putative ISVs in Table 2.

Plant viruses were also identified (as reported previously in a survey of DNA sequencing of mosquitoes^28^) and (perhaps not surprisingly) were unique to each population (Tables 2 and S2). Mycoviruses were also identified and were also largely unique to each population (Table S4). These mycoviruses may in part arise from fungi growing on dead mosquitoes in the traps.

### Bacterial and fungal sequences

The bacterial and fungal sequences showed a considerable number of differences between mosquitoes from Cairns and Bangkok (Fig. 2b,c). The most prominent difference in bacterial composition was 32,232 reads taxonomically assigned to the genus *Wolbachia* for Cairns mosquitoes, whereas no reads from Bangkok mosquitoes were assigned to *Wolbachia* (Fig. 2b). *Wolbachia* are not naturally found in *Ae*. *aegypti*, but detection of *Wolbachia* reads in the Cairns mosquitoes might be expected as *Wolbachia* (*w*Mel) has become stably established in these mosquito populations^11^. *Wolbachia* reads comprised a reasonably high percentage (2.7%; 32,232/1,211,857) of non-chordate, non-mosquito reads (Fig. 1). However, based on the locations of the traps and recent mapping of the spread of *Wolbachia*^29^, we estimate that the percentage of mosquitoes infected with *Wolbachia* caught in the traps is likely to be below (possibly well below) 30%. Any ability of *Wolbachia* to suppress ISVs^20,21^ would thus not be readily discernible from this data set.

Other differences in the bacteriome include bacteria from the genera *Pantoea*, *Sphingobacterium* and *Bacillus* in the Cairns mosquitoes, and bacteria from the genera *Klebsiella* and *Aeromonas* in the Bangkok mosquitoes (Fig. 2b). Many of these bacterial genera have previously been identified in *Ae*. *aegypti*, including *Enterobacteriaceae*, *Aeromonas*, *Pantoea*, *Bacillus*, *Sphingobacteria* and *Klebsiella*^30–32^.

The fungal composition differed substantially between the Australian and Thai data sets (Fig. 2c). Sequences from the microsporidian *Ae*. *aegypti* parasite *Edhazardia aedis*^33^ dominated fungal RNA transcripts in the Bangkok mosquitoes, whereas no reads from Cairns mosquitoes mapped to the reference genome of *E*. *aedis* (USNM 41457, V4b) using BWA. No reads from either Cairns or Bangkok mosquitoes mapped to the reference genome of *Vavraia culicis*, which has been reported to infect *Ae*. *aegypti*^33^. The presence of *Penicillium* in the Cairns mosquitoes (Fig. 2c) is supported by the detection of all four proteins of the *Penicillium chrysogenium* virus (Table S3). *Penicillium* species have been reported to infect a number of mosquitoes including *Aedes* species^34^. Fungi in the *Cordycipitaceae* family (order *Hyocreales*) infect aquatic larval stages of a range of culicid dipterans including *Ae*. *aegypti*^35^.

To what extent these bacterial and fungal sequences arise from environmental contaminants (e.g. *Aspergillaceae*) and/or change in abundance after mosquitoes have been captured remains unclear. Irrespective of such confounding issues, the differences provide a clear and contrasting internal control for the similarities seen in the viromes (Fig. 2).

### Blood meal RNA

As RNA is regarded as relatively unstable, one might expect limited RNA sequence data from ingested blood meals. However, of the non *Ae*. *aegypti* reads, 8646 (Cairns) and 8744 (Bangkok) reads (Fig. 1) mapped to *Homo sapiens* genes (Table S5), consistent with the anthropophilic feeding behavior of *Ae*. *aegypti*^36,37^. These reads mapped to a large number of genes; in total, 327 genes were identified that had at least 5 reads in either of the two datasets. The highest frequency of mapping was to 28 S ribosomal, 16 S and 12 S mitochondrial ribosomal, hemoglobin and ubiquitin genes, and 7SL RNA (Table S5). The ribosomal species likely represent an underestimation, as library preparation included a ribosomal RNA depletion step. Hemoglobin mRNA can be found in red blood cells^38^, 7SL is an abundant cytoplasmic RNA^39^ and ubiquitin mRNA can be a major stress-induced transcript in mammalian cells^40^. These RNA species are thus presumably abundant and/or stable in the midgut environment.

Previous work suggests *Ae*. *aegypti* does occasionally feed on bovines, swine, cats, rats, and/or chickens^37^. To determine whether the RNA-Seq approach might also provide evidence for these low frequency hosts, non-human chordate reads were aligned using BLAST (Fig. 1, Table S6). A number aligned to mouse/rat genes (79 for Cairns and 190 for Bangkok), 5 Bangkok reads aligned to dog genes, and 1 Cairns and 1 Bangkok read aligned to cow genes (Table S6). These reads all aligned with lower scores to human genes (Table S6), supporting the view they were not derived from human genes. Taken together these results argue that RNA-Seq is able to identify mosquito blood meal hosts. However, the use of pools (to contain costs) results in loss of individual mosquito data, which can be cost-effectively retained when using ELISA^37^ or PCR-based methods^41,42^.

A current methodology for blood meal species identification involves extraction of DNA followed by PCR using primers targeting mitochondrial cytochrome b and/or cytochrome c oxidase subunit I gene sequences^41,42^. No sequences mapped to the mitochondrial genomes of rat (*Rattus norvegicus*, NC_001665.2), dog (*Canis lupus familiaris*, NC_002008.4), pig (*Sus scrofa*, NC_000845.1) or chicken (*Gallus gallus*, NC_001323.1).

### Mosquito genetic diversity: mitochondrial SNPs

**Figure 4:**
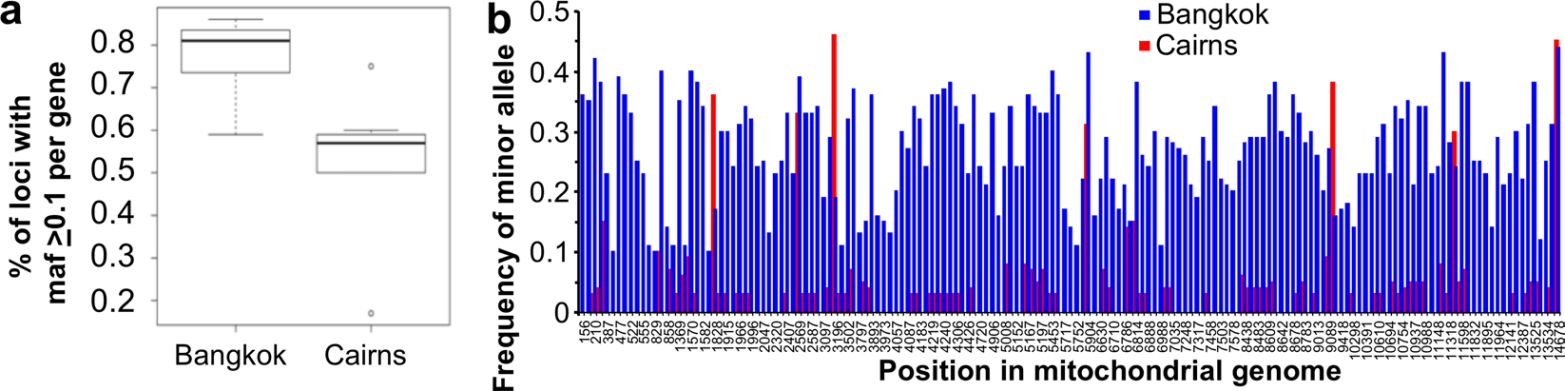
Mitochondrial sequence diversity in *Ae*. *aegypti* from Cairns and Bangkok. For Cairns 611,909 and for Bangkok 4,021,144 reads aligned with the *Ae*. *aegypti* mitochondrial genome AaegL3. Only nucleotide sites with at least 100 reads in both Cairns and Bangkok data sets were considered. (**a**) Box plot of the percentage of polymorphic sites (where the minor allele frequency > 0.1) for per site with a read coverage of > 100, using seven mitochondrial genes (ND2, COX1, COX2, COX3, ND4, CYTB, rRNA). (**b**) The minor allele frequency for nucleotide positions in the mitochondrial genome with a read depth > 100 reads. Only sites with a minor allele frequency of > 0.1 in Cairns and/or Bangkok data sets are shown.

A common method for assessing genetic diversity in a mosquito population is to analyze mitochondrial sequence diversity^29,43^. Reads were mapped to the *Ae*. *aegypti* mitochondrial genome (NCBI acc. num. EU352212.1) using BWA mem (Fig. 1). To compare the genetic diversity between the two samples, only sites that had > 100 read coverage and a minor allele frequency (maf) of > 0.1 were considered. The mitochondrial genome diversity (expressed as a percentage of polymorphic sites per gene) was higher in mitochondrial reads from Bangkok than those from Cairns (Fig. 4a). As an alternative representation of the data, the maf (where maf > 0.1 in either Bangkok or Cairns data sets) was plotted against the position in the mitochondrial genome, again using only positions with a read coverage of >100 (Fig. 4b).

Mosquitoes from Bangkok thus displayed higher mitochondrial diversity than those from Cairns (Fig. 4). The lower mitochondrial diversity in Australian *Ae*. *aegypti* is consistent with previous studies^29^, and the data illustrates how RNA-Seq can provide information on the genetic diversity of the mosquito populations.

### SNPs associated with resistance to pyrethroid insecticides

Resistance to pyrethroid insecticides or “knockdown resistance (kdr)” often involves point mutations in the insect Voltage Gated Sodium Channel gene (*VGSC*)^44^. Such resistance and associated mutations are now widespread globally and are present in *Ae*. *aegypti* populations from Bangkok^41^, while pyrethroid susceptibility has been maintained in *Ae*. *aegypti* from northern Queensland (Australia)^42^, where Cairns is located. Reads were mapped to *Ae*. *aegypti VGSC* gene sequence (VectorBase AAEL006019-RD); 167,289 reads from the Cairns and 118,553 from the Bangkok dataset (Fig. 1). Several polymorphisms recently reported as putative “resistance” kdr non-synonymous mutations^44^ were detected in mosquitoes from Bangkok (consistent with^41^), but not in mosquitoes from Cairns (consistent with^42^) (Table 3). The Cairns mosquitoes did, however, show a series of synonymous SNPs recently reported from other parts of the world^19^ (Table 3). No reads mapped to the most commonly analyzed “kdr” mutation sites in exons 20, 21 and 31 (sites 989, 1011, 1016, 1534)^45^.

**Table 3:** Voltage-gated sodium channel gene SNPs in the *Ae*. *aegypti* genomes.

Exon	bp position in exon	Codon change	Amino acid change	Cairns	Read depth	Bangkok	Read depth
15	115,365	Tcc/Ccc	Y	T 100%	87	T 77% C 23%	26
24	155,477	aCc/aTc	Y	C 100%	26	C 71% T 29%	14
24	155,519	tTc/tGc	Y	T 100%	55	T 36% C 64%	22
15	115,283	aaG/aaA	N	G 44% A 56%	41	G 100%	21
15	115,313	ttC/ttT	N	C 59% T 41%	61	C 100%	27
25	155,890	ttT/ttC	N	T 65% C 35%	23	T 100%	61

Amino acid change Y; non-synonymous SNPs associated with pyrethroid resistance (knockdown resistance, kdr) (VectorBase: AAEL006019-RD, supercont1.186: 18,685–170,948). Amino acid change N; synonymous SNPs found in Cairns *Ae*. *aegypti*.

## Discussion

The work described herein highlights the wide spectrum of information that can be obtained from RNA-Seq analysis of wild-caught mosquitoes. The study provided insights into the microbiome, the source of blood meals, the genetic diversity of the mosquito population and insecticide resistance SNPs. In particular, the study illustrates the rich virome of *Ae*. *aegypti*.

A striking pattern that emerges from the data is the similarity in the ISV populations that were identified in *Ae*. *aegypti* from the two continents, with an estimated 16 ISV infections shared by the mosquitoes from Cairns and Bangkok. This includes known ISVs and several new viruses, which are presumably also ISVs, given their homology to known ISVs (Tables 2 and S2). Although not entirely clear, *Ae*. *aegypti* probably reached Asia and Australia in the late 1700’s^46^. Australia’s extensive bio-security measures and the minimal international shipping traffic in Cairns, would likely limit introduction of Asian mosquitoes into the Australian population. The similar viromes (and high ISV contig sequence homologies, Fig. S2) in the Cairns and Bangkok mosquito populations may thus suggests that many ISVs had co-evolved with their *Ae*. *aegypti* hosts for considerable periods (and had formed stable host/virus relationships) well before the 1700s. Such co-evolution is clearly fostered by the ability of many ISVs to be transmitted directly from the female mosquito to their progeny via infected eggs^2^. In contrast, most mosquito-associated bacteria (with the exception of endosymbionts like *Wolbachia*) and fungi are acquired from the environment at the larval or adult stages^47^.

The studies presented herein argue that for one of the world’s most important vector species, we still have a number of ISVs to characterize. Considering both mosquito populations, up to 23 uncharacterized ISVs would appear to exist (Tables 2 and S2, Fig. S2) including, for instance, (i) a probable *Ae*. *aegypti* totivirus, previously seen in sequence data from Thai *Ae*. *aegypti*, (ii) a flavivirus (showing homology to Xishuangbanna flavivirus that was identified in *Ae*. *albopictus*), (iii) an orthomyxovirus (nearest relative Whidby virus), (iv) a mononegavirus (nearest relative Xincheng mosquito virus), (v) a bunyavirus (nearest relative Wuhan mosquito virus 2), (vi) a rhabdovirus (nearest relative Wuhan mosquito virus 9), (vii) a virus related to Blackford virus and (viii) a baculovirus closely related to Autographa californica nucleo-polyhedrovirus. A number of viral contigs also suggested 5–6 unique ISVs may be present in each population. Most of the contigs identified ISVs as the best match out of all the sequences in the NCBI data base (Tables 2 and S2), and clustered with purported ISVs (Fig. S2), suggesting the RNA-Seq approach adopted herein is relatively robust. Isolation of replicating ISVs and obtaining their full genomic sequence would, however, arguably remain the tried and tested method for completely unravelling the clearly quite complex *Ae*. *aegypti* virome.

Analyses of other RNA-Seq data sets with respect to abundance of the three main ISVs, PCLV, CFAV and HTV (Table 1, Fig. 3), perhaps suggests the intriguing possibility that certain ISV infections can diminish substantially when mosquitoes are introduced to the laboratory (although it should be reiterated that the data in Table 1 was obtained after polyA enrichment). Conditions in the wild and in the laboratory clearly differ, with the optimal laboratory conditions generally increasing mosquito fitness. For instance, laboratory reared mosquitoes are usually larger than wild mosquitoes^48,49^. Increased fitness and/or consistent laboratory conditions may improve certain mosquito anti-viral activities^50^, with temperature fluctuations (largely absent in the laboratory) reported to affect insect immunity^51,52^. Improved nutritional status has also been shown to reduce viral transmission in at least one setting^53^. However, serial RNA-Seq experiments would be needed to establish whether, how quickly and under what conditions ISV infections might change as mosquitoes are introduced into the laboratory. Such studies would be greatly facilitated if we had a considerably more detailed picture of the ISVs that infected *Ae*. *aegypti*.

That ISVs can affect transmission of medically important arboviruses is now considered likely^4,6,54,55^. That laboratory vector competence studies can be inconsistent and may not reliably reflect transmission in the field is becoming increasingly recognized^56^. The mosquito virome may thus represent a complicating, hard to control variable that may need to be characterized and considered in association with vector competence assessments.

## Methods

### Mosquito collection

Mosquitoes were collected in Cairns (Earlville, Bungalow, Parramatta Park, Manoora areas), Queensland (Australia) March-June 2014 using BG Sentinel mosquito traps^57^. About 150 *Ae*. *aegypti* mosquitoes were dismembered and placed into RNAlater (Life Technologies), kept at 4 °C and transported to QIMR Berghofer. Mosquitoes were similarly collected in Bangkok (Thailand) June 2015 and ≈150–200 *Ae*. *aegypti* mosquitoes were dismembered and placed in RNALater kept at 4 °C and transported to QIMR B.

### RNA preparation and RNA-Seq

Mosquitoes were homogenized in TRIzol (Invitrogen) using 4 × 2.8 mm ceramic beads (MO BIO Inc., Carlsbad, USA) and a Precellys24 Tissue Homogeniser (Bertin Technologies, Montigny-le-Bretonneux, France) (3 × 30 s, 6000 rpm on ice). Homogenates were centrifuged (12,000 g × 10 min) and RNA extracted from the supernatants as per manufacturer’s instructions. RNA concentration and purity was determined by Nanodrop ND 1000 (NanoDrop Technologies Inc.). The RNA samples were DNase treated using RNase-Free DNase Set (Qiagen), purified using an RNeasy MinElute Kit, and sent to the Australian Genome Research Facility (Melbourne, Australia). The Cairns and Bangkok samples were processed and sequenced independently using identical protocols; the former in 2014, the latter in 2015.

Library preparation and sequencing were conducted by the Australian Genome Research Facility (Melbourne, Australia). The Ribo-Zero Gold Kit (Human/Mouse/Rat) was used to deplete ribosomal RNA. cDNA libraries were prepared using a TruSeq RNA Sample Prep Kit (v2) (Illumina Inc. San Diego, USA) and were sequenced from both ends (150 bp) using 1 lane for each sample and the Illumina HiSeq. 2500 Sequencer (Illumina Inc.). The CASAVA v1.8.2 pipeline was used to separate the bar-coded sequences and extract 150 base pair, paired end reads into FASTQ files.

### Read-based analysis

Raw paired-end files were processed for removal of Illumina adaptor sequences, trimmed and quality-based filtered using Trimmomatic software v0.32^58^. The remaining reads were merged using PEAR v0.9.6^59^. PhiX control sequences were identified using BWA mem and excluded from the data set. The merged and unmerged reads were mapped onto the reference genome of *Ae*. *aegypti* v3.30^60^ using STAR v2.4.2a^61^ and BWA mem. A custom database of ribosomal RNA sequences was generated using SILVA v123 LSU, SSU and 5 S rRNA (RF00001), 5.8 S rRNA (RF00002), tRNA (RF00005), Ribonumclease P (RF00010, RF00011, RF00373). BLAST^62^ was applied to identify ribosomal sequences in the transcriptome data using an E-value cut-off of 10^–05^. Reads with an identity of > 60% and 60% of the read length covered were marked as ribosomal and excluded from further analysis. DIAMOND^63^ with an E-value cut-off of 10^−05^ was used to search for matches in the NCBI protein reference database (version 18 March 2016). The read sequences were assigned to the taxonomic lowest common ancestor (LCA) using all hits whose score was at most 10% lower than the best score. Reads that were assigned to Chordata or Diptera were excluded from the downstream analysis. The taxonomic assignments were visualized using Krona^12^ showing only taxa with relative abundance of at least 0.01%.

### Contig-based analysis

*De novo* assembly was performed with the Trinity software^64^ using the paired-end mode. Genes were identified in viral contigs using BLAST. First, a viral protein database was constructed by extracting all viral sequences (taxonomy identifier: 10239) from the NCBI protein collection NR database. This database was used as a reference for a blastX search with an E-value cut-off of 10^−05^. Contigs with a match to this database were subsequently blasted against the complete NCBI protein and nucleotide database to include only contigs that had a hit to a viral reference sequence. The Cairns reads and Bangkok reads were mapped against the Bangkok contigs and Cairns contigs using BWA mem, respectively, to identify unique and shared viruses.

### Phylogenetic tree reconstruction of viral RNA-dependent RNA polymerases

Phylogenetic trees of the protein sequences of the viral RdRp transcripts encoded by the assembled contigs were reconstructed to infer the phylogenetic relationships among RNA viruses from the two different locations Cairns and Bangkok. Reference sequences of the closest BLAST matches of RdRp discovered in this study were downloaded from GenBank. All protein sequences were aligned using MAFFT v7.380^65^ employing the E-INS-i algorithm. The alignment was trimmed to ensure it contains only the RdRP sequences. Ambiguously aligned regions were removed using TrimAl tool v1.2^66^. PhyML v3.0^67^ using the smart model selection approach^68^ was applied to reconstruct the phylogenetic trees.

### Phasi Charoen SNPs

Reads were mapped to Phasi Charoen-like virus (KM001085.1, KM001086.1, KM001087.1) using BWA mem and the distribution of reads on the Phasi Charoen-like virus genome was visualized using a custom script. For the identification of single nucleotide variants in the Phasi Charoen-like virus, reads were mapped against the reference sequences using BWA MEM. SNPs were identified using samtools mpileup and bcftools. Variants with an allele frequency of <20% or with less than 100 reads coverage were excluded. Read coverage and SNPs were visualized using custom scripts.

### Interrogation of other RNA-Seq data sets

Reads from the data sets (ERX1788144-6, ERX1788141-3, ERX1788135-7, ERX1788138-40, ERX1788126-8, ERX1788132-4, ERX1788129-31, Aag2 - SRX1367297, SRX1366586) were mapped to Phasi Charoen-like virus (KM001085.1, KM001086.1, KM001087.1), Cell fusing agent virus (NC_001564) and Humaita-Tubiacanga virus (KR003801, KR003802.1) using Bowtie 1.2.0^69^ or BLAST.

### *Ae*. *aegypti* mitochondrial diversity analysis

Illumina reads that mapped to the mitochondrial *Ae*. *aegypti* genome AaegL3 using BWA mem and STAR were subsequently processed with samtools mpileup and bcftools for the identification of SNPs. Only SNPs with a read coverage of at least 100 and a minor allele frequency of at least 0.1 were included.

### “kdr” SNP analyses

*Ae*. *aegypti* reads were aligned using BWA mem to the voltage gated voltage-gated sodium channel gene (VectorBase: AAEL006019-RD, supercont1.186: 18,685-170,948). SNPs were identified using samtools mpileup and Integrated Genome Viewer (Broad Institute).

### Blood meal species identification

All trimmed and quality-controlled reads were mapped to the human reference genome (ensemble release 75; human assembly GRCh37) using STAR v2.5.2a. Genes were derived based on the genome coordinates. Only genes with at least 5 reads were included in the output. Reads assigned to the phylum Chordata using DIAMOND and LCA were filtered for non-human reads and non-repetitive elements using STAR v2.4.2a with the human genome as reference and tandem repeat finder version 4.7b, respectively. The remaining reads were aligned to the entire NCBI nucleotide database using BLAST. Reads matching mammalian reference sequences with at least 95% alignment identity and 95% sequence read coverage were subsequently aligned to a human filtered (taxonomy identifier 9606) NCBI nucleotide database.

### Data Availability

The Illumina mosquito sequencing data generated for this study are available from NCBI SRA, bioproject number PRJNA413709; BioSamples SAMN07764273 (Bangkok), SAMN07764275 (Cairns), SAMN07764276 (Cairns).

## Electronic supplementary material

Supplementary Information
