# Supplementary material for: Mapping the virome in wild-caught Aedes aegypti from Cairns and Bangkok
Source: Sci Rep. 2018 Mar 16;8:4690. doi: 10.1038/s41598-018-22945-y (PMC5856816; doi:10.1038/s41598-018-22945-y)
Supplement: Supplementary file 1 — Supplementary Information [file 41598_2018_22945_MOESM1_ESM.pdf]

Supplementary information for:

Mapping the virome in wild-caught *Aedes aegypti* from Cairns and Bangkok

Martha Zakrzewski[1], Gordana Rasic[2], Jonathan Darbro[2], Lutz Krause[1], Yee S. Poo[3], Igor Filipovic[2], Rhys Parry[4], Sassan Asgari[4], Greg Devine[2], Andreas Suhrbier[3,*]

[1] Medical Genomics, QIMR Berghofer Medical Research Institute, Brisbane, Qld. 4029, Australia.

[2] Mosquito Control, QIMR Berghofer Medical Research Institute, Brisbane, Qld. 4029, Australia.

[3] Inflammation Biology, QIMR Berghofer Medical Research Institute, Brisbane, Qld. 4029, Australia.

[4] School of Biological Sciences, The University of Queensland, Brisbane, Qld. 2072, Australia

[*]Correspondence: Andreas.Suhrbier@qimrberghofer.edu.au

Running title: Mapping the *Aedes aegypti* virome

Key words: *Aedes aegypti*, insect-specific virus, Phasi Charoen-like virus, Humaita-Tubiacanga virus, Cell fusing agent virus.

Figure S1. (**a**) Read based mapping (using data from Cairns mosquitoes) to PCLV genome segments using BWA mem (colour coding represents percent nucleotide identity). (**b**) Single nucleotide polymorphisms (SNPs) in reads mapping PCLV. SNPs with at least 100 reads and an allele frequency of at least 20% are shown. Cairns 698,434 reads and Bangkok 2,080,162 reads.

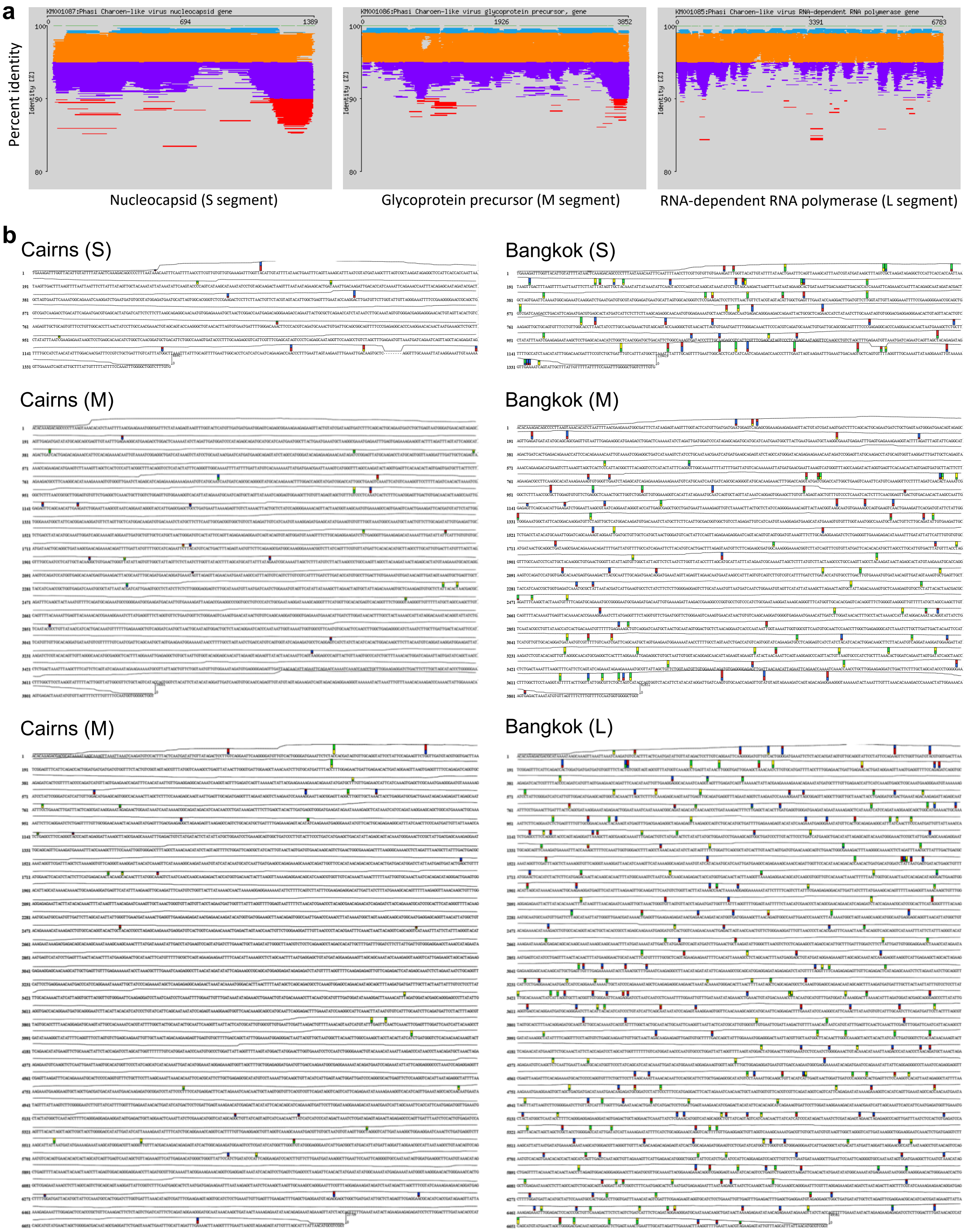

Figure S2: Maximum likelihood phylogeny of (**a)** putative negative-sense RNA viruses and (**b**) putative positive-sense and double-stranded RNA viruses. Phylogentic trees of the viral RdRp protein sequences encoded on Cairns and Bangkok contigs and reference sequences from GenBank. Viruses shared between Cairns and Bangkok cluster closely within a clade. Some viruses were unique to each geographic location.

a

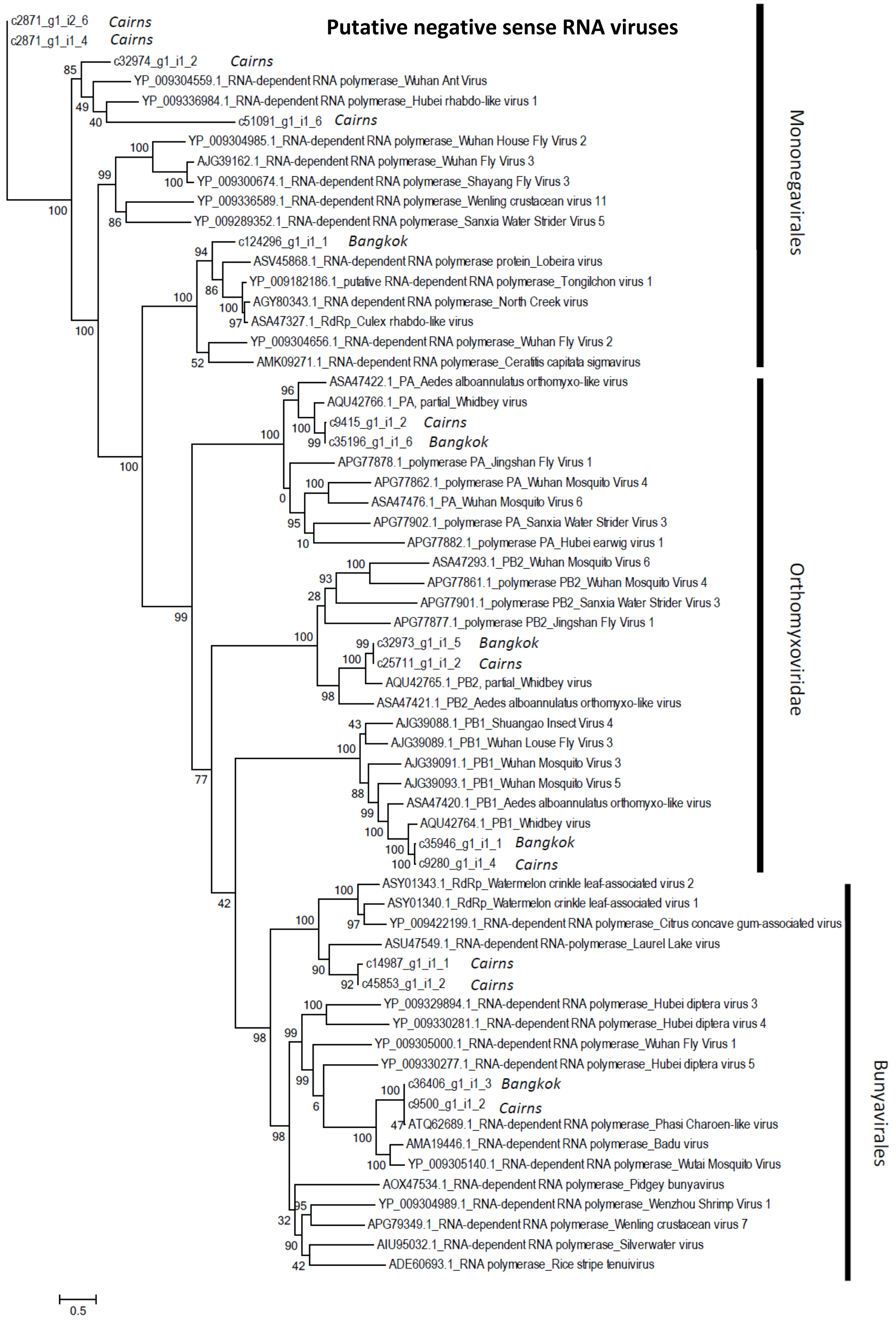

b

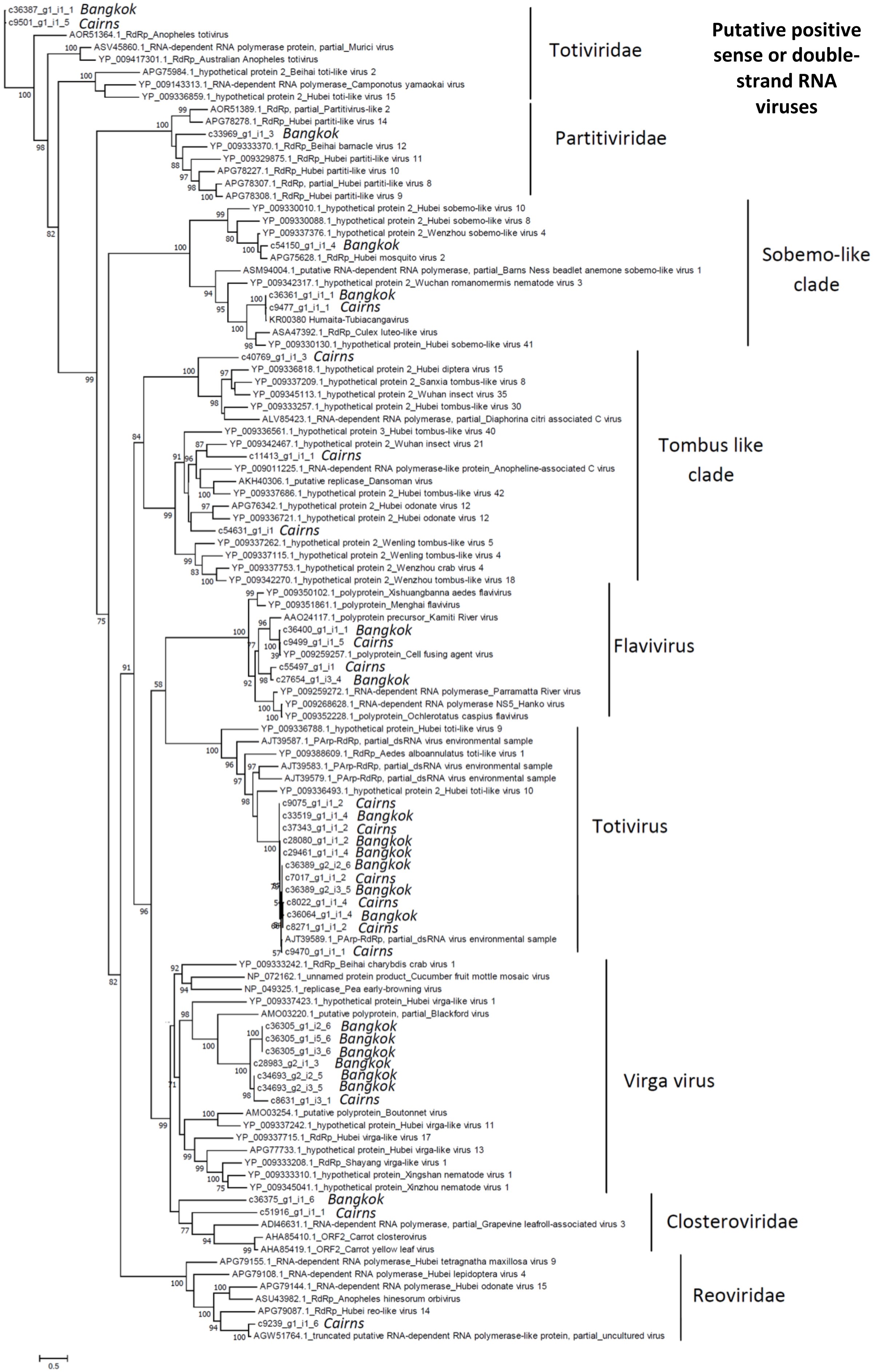
Putative positive sense or double-strand RNA viruses
c36387_g1_i1_1 Bangkok
c9501_g1_i1_5 Cairns
AOR51364.1_RdRp_Anopheles totivirus
ASV45860.1_RNA-dependent RNA polymerase protein, partial_Murici virus
YP_009417301.1_RdRp_Australian Anopheles totivirus
APG75984.1_hypothetical protein 2_Beihai toti-like virus 2
YP_009143313.1_RNA-dependent RNA polymerase_Camponotus yamaokai virus
YP_009336859.1_hypothetical protein 2_Hubei toti-like virus 15
Totiviridae
AOR51389.1_RdRp, partial_Partitivirus-like 2
APG78278.1_RdRp_Hubei partiti-like virus 14
c33969_g1_i1_3 Bangkok
YP_009333370.1_RdRp_Beihai barnacle virus 12
YP_009329875.1_RdRp_Hubei partiti-like virus 11
APG78227.1_RdRp_Hubei partiti-like virus 10
APG78307.1_RdRp, partial_Hubei partiti-like virus 8
APG78308.1_RdRp_Hubei partiti-like virus 9
Partitiviridae
YP_009330010.1_hypothetical protein 2_Hubei sobemo-like virus 10
YP_009330088.1_hypothetical protein 2_Hubei sobemo-like virus 8
YP_009337376.1_hypothetical protein 2_Wenzhou sobemo-like virus 4
c54150_g1_i1_4 Bangkok
APG75628.1_RdRp_Hubei mosquito virus 2
ASM94004.1_putative RNA-dependent RNA polymerase, partial_Barns Ness beadlet anemone sobemo-like virus 1
YP_009342317.1_hypothetical protein 2_Wuchan romanomermis nematode virus 3
c36361_g1_i1_1 Bangkok
c9477_g1_i1_1 Cairns
KR00380 Humaita-Tubiacangavirus
ASA47392.1_RdRp_Culex luteo-like virus
YP_009330130.1_hypothetical protein_Hubei sobemo-like virus 41
Sobemo-like clade
c40769_g1_i1_3 Cairns
YP_009336818.1_hypothetical protein 2_Hubei diptera virus 15
YP_009337209.1_hypothetical protein 2_Sanxia tombus-like virus 8
YP_009345113.1_hypothetical protein 2_Wuhan insect virus 35
YP_009333257.1_hypothetical protein 2_Hubei tombus-like virus 30
ALV85423.1_RNA-dependent RNA polymerase, partial_Diaphorina citri associated C virus
YP_009336561.1_hypothetical protein 3_Hubei tombus-like virus 40
YP_009342467.1_hypothetical protein 2_Wuhan insect virus 21
c11413_g1_i1_1 Cairns
YP_009011225.1_RNA-dependent RNA polymerase-like protein_Anopheline-associated C virus
AKH40306.1_putative replicase_Dansoman virus
YP_009337686.1_hypothetical protein 2_Hubei tombus-like virus 42
APG76342.1_hypothetical protein 2_Hubei odonate virus 12
YP_009336721.1_hypothetical protein 2_Hubei odonate virus 12
c54631_g1_i1 Cairns
YP_009337262.1_hypothetical protein 2_Wenling tombus-like virus 5
YP_009337115.1_hypothetical protein 2_Wenling tombus-like virus 4
YP_009337753.1_hypothetical protein 2_Wenzhou crab virus 4
YP_009342270.1_hypothetical protein 2_Wenzhou tombus-like virus 18
Tombus like clade
YP_009350102.1_polyprotein_Xishuangbanna aedes flavivirus
YP_009351861.1_polyprotein_Menghai flavivirus
AAO24117.1_polyprotein precursor_Kamiti River virus
c36400_g1_i1_1 Bangkok
c9499_g1_i1_5 Cairns
YP_009259257.1_polyprotein_Cell fusing agent virus
c55497_g1_i1 Cairns
c27654_g1_i3_4 Bangkok
YP_009259272.1_RNA-dependent RNA polymerase_Parramatta River virus
YP_009268628.1_RNA-dependent RNA polymerase NS5_Hanko virus
YP_009352228.1_polyprotein_Ochlerotatus caspius flavivirus
Flavivirus
YP_009336788.1_hypothetical protein_Hubei toti-like virus 9
AJT39587.1_PArp-RdRp, partial_dsRNA virus environmental sample
YP_009388609.1_RdRp_Aedes alboannulatus toti-like virus 1
AJT39583.1_PArp-RdRp, partial_dsRNA virus environmental sample
AJT39579.1_PArp-RdRp, partial_dsRNA virus environmental sample
YP_009336493.1_hypothetical protein 2_Hubei toti-like virus 10
c9075_g1_i1_2 Cairns
c33519_g1_i1_4 Bangkok
c37343_g1_i1_2 Cairns
c28080_g1_i1_2 Bangkok
c29461_g1_i1_4 Bangkok
c36389_g2_i2_6 Bangkok
c7017_g1_i1_2 Cairns
c36389_g2_i3_5 Bangkok
c8022_g1_i1_4 Cairns
c36064_g1_i1_4 Bangkok
c8271_g1_i1_2 Cairns
AJT39589.1_PArp-RdRp, partial_dsRNA virus environmental sample
c9470_g1_i1_1 Cairns
Totivirus
YP_009333242.1_RdRp_Beihai charybdis crab virus 1
NP_072162.1_unnamed protein product_Cucumber fruit mottle mosaic virus
NP_049325.1_replicase_Pea early-browning virus
YP_009337423.1_hypothetical protein_Hubei virga-like virus 1
AMO03220.1_putative polyprotein, partial_Blackford virus
c36305_g1_i2_6 Bangkok
c36305_g1_i5_6 Bangkok
c36305_g1_i3_6 Bangkok
c28983_g2_i1_3 Bangkok
c34693_g2_i2_5 Bangkok
c34693_g2_i3_5 Bangkok
c8631_g1_i3_1 Cairns
AMO03254.1_putative polyprotein_Boutonnet virus
YP_009337242.1_hypothetical protein_Hubei virga-like virus 11
YP_009337715.1_RdRp_Hubei virga-like virus 17
APG77733.1_hypothetical protein_Hubei virga-like virus 13
YP_009333208.1_RdRp_Shayang virga-like virus 1
YP_009333310.1_hypothetical protein_Xingshan nematode virus 1
YP_009345041.1_hypothetical protein_Xinzhou nematode virus 1
Virga virus
c36375_g1_i1_6 Bangkok
c51916_g1_i1_1 Cairns
ADI46631.1_RNA-dependent RNA polymerase, partial_Grapevine leafroll-associated virus 3
AHA85410.1_ORF2_Carrot closterovirus
AHA85419.1_ORF2_Carrot yellow leaf virus
Closteroviridae
APG79155.1_RNA-dependent RNA polymerase_Hubei tetragnatha maxillosa virus 9
APG79108.1_RNA-dependent RNA polymerase_Hubei lepidoptera virus 4
APG79144.1_RNA-dependent RNA polymerase_Hubei odonate virus 15
ASU43982.1_RdRp_Anopheles hinesorum orbivirus
APG79087.1_RdRp_Hubei reo-like virus 14
c9239_g1_i1_6 Cairns
AGW51764.1_truncated putative RNA-dependent RNA polymerase-like protein, partial_uncultured virus
Reoviridae
0.5

Table S1. Contig nucleotide sequences (len = nucleotide length).

Cairns contigs

```
>c54631_g1_i1_len=221
TGCTGTGATATTATCCGCACACTTGCAAAGTTTCACACCACTGTCAGCGATAAGTCCGCACCGTACCTCGTGTGTGCTAAAGCCTTGTCCTACTAT
TACACAGATGCACACACACCACTTGTTGGCCCTTTGGCTAGATCTCTTGTCAGACTACTCCTTCCCCAGCTCAATCATCGCAAGCTTAGACGGATT
ATTCGTGATGCAACTCGAGCACTACCCTG

>c45395_g1_i1_len=228
GAATGGTTAGAGCTCATCAATAAGACCATAGGGTGCTGGTCAGCCAAGGATTCTAGCGATATGCCATCTCTGGTACATAGGCTGGTGATAGCCGGG
GAGGACGGCAAACTTAATGGGATTGGTTCAACCCTCAGTGGTAATTCCAAGTACACGACAACAGTCAACAACGCCATTAAGCATGGGAACCCGGGA
AAATTTGAAAGCTTGTTGGGATCCATGATGAGGGAG

>c35321_g1_i1_len=563
GTTGAAATCAATAATTTTGAAACAAGATCTCTCGATAAAGATAATATTAATTTGGGATACTCATGCGATAAAAAACCATCACCCATTGATTTTAAA
CAAGAAAATCTAAATAAAGAGGAAAAATCCGAACTTTTGAAGGTATTAAATAAGTTCCGTGACGTTTTTCGTACAGACGATACTGTACCATTAACG
TTCACCAATGCGATACGGCATCAAATAAACACTAAGGACGACTTACCTGTGTATACAAAATCGTATCGATATCCCTTTTTCCATAAAGCAGAAGTG
CAAAATCAAATCGGGAAAATGCTAAATCAGGGCATTATTCGTCCTTCGAATAGCCCATGGTCTTCACCGATATGGATAGTCCCGAAGAAATTAGAT
TCTTCAGGCCAAAAGAAATGGAGGCTAGTTATCGATTATCGAAAGTTGAATGATAAAACTATCGATGATCGATATCCTATCCCTAATATTACTGAC
ATTTTAGACAAGCTAGGAAGATGTCAGTACTTCTCAACTATCGATTTGGCTTCTTGATTTCACCAGATCGAAGTTGATAAGAA

>c177_g1_i1_len=240
CGATGGTTTGTACTGAAGCCACTTTGTTATAATCACTATCCTGCATTGCCGGTTTCACTGCAGTTTTGATCATAAAGTTGAAAAGATTAGTTAGTC
GCTCGTTAATACGTAAATCGGACTATGACAGTTTGCGCACGACGTCGGGATTGTCTTGCGTGCGCAACCAGAGGGAAAGAGCATGTCGTAATATAT
TTGGAGGTTACCGACGGAACCATGGACGGCGGCAGGCGAGAAAGGGAG

>c8631_g1_i2_len=333
CTGCCCTATATTCATAAAGTTGAAACGCCGTTGGTTGTCCGTTATGCGGTCGAAGTATGTCTACCTCACCGACCTATCCCTCGACGAATTTGAAGA
ACTACTAAACAAACGATTCTCTTCGATTGAATACCTCAATTCGACGAGAATCGAAAACGACATATCGAAATATGACAAGTCTATGCGAGCTTGGAC
GCTTGTCACTGACGCGGTAGTTTTATAATTTACAGATTCAATTATTGGCAAATTAGCAAATTGAAATTGATTCCGATCGACCTCCAGTCGGTCAAT
AACCTAGGAAACAGAAAACAAACCCGACCCAACATATGGGCAACG

>c8631_g1_i3_len=203
GCCGCTTTGAAATCCGTCAATGCACTTTACTGCCCTATTTTCATAAAGTTGAAACGCCGTTGGTTGTCCGTTATGCGGTCGAAGTATGTCTACCTC
ACCGACCTATCCCTCGACGAATTTGAAGAACTACTAAACAAACCTCAATTCGACGAGAATCGAAAACGACATATCGAAATATGACAAGTCTATGCG
AGCTTGGACGC

>c9229_g1_i1_len=1131
CCATGTGTTCCGATTATGCTACCACATTCTTGATAGTGTTTCTTGTGGAGATAGGGGAGATCCTCCCACTTTTCGACCCATTCTTTATGTTTCATC
CATGGTCAACGAGGTAGAGTCAATGAAACAGTGCAATGGGCGGAAAGCGACGACGAATTCCTCATAGGGCATTGGGGACACAGGAACGGATAGTAT
CTTAGGAAACCATCCGTGGAGTGAATACGGAAGTGAGAGATTATGGAGTGCGCGTGGAATAACCATACCGAATGCGTCGGGACAGTAAAACACACA
CAGGTCGTGTGGTGTGGGAAAAGGTGGGTTCCTCGGCTTACACTTCAAGGGACGGGAGGGACGGATCGAGAAGGAGACGATCGTCAGGCAAAGAAT
AATACAGCTTCGAAAATTCATGTTGTGATGAGACTACGAAATTAATGGAGTGAAATAAGCCGGAAAATTCGGTGATTGGAGTGTTGTAACGTTCGG
AAATGGCTTCGGACAGTTGACGGTATATGTAGGCATTCATTAAGGGCTTCATGTTGTCTTTGCACGAGATTTGATACTGCGCCACGTGTTCCCAGT
TACGAAGATCAATCTACCTAGTTTCATCAGTAACTTAATGGGGCCAGGAACAACAGTGAAGCCGTCAATGGAGGCGAGGATGAATTTACCGCAAAA
ATAAGAAGTTTCAAAGTGCGTTAAGGTTTTTGCTTCAAAGTTAAAAATGTGCGAGTAAATGGGTGCAACGTTGTCCAGAAGAGAACTTAGACCCCA
CAGGTGATTGTCATCACCGGCAAAAACACCGCCGAGCAATTCGTAGTCGGCGTAAATTGCCATTGCGACTGCATGATTGACGAGGGTGTTTCCAAA
AAAGGTCGAAGCATCACCGGAACGGCGTTGGTAGGCGACGGTAGTCGAAAAACCACTAACAGAATCGAGATACCGAGATAGGACATGACTCTGATA
CCAGATCAAGGCTAAATGATCTGGTAAGCCTAGAGCCTTATAAATGCGCGTTTCAAACAATAGCGTTGTCGCGCACTGAAGCCTAAGCTTCCGACC
CGAAGCACACCGAGTTAATCCGTAACCCGATGTTACGTTCCGCGAAGGTTTGAACTCTTCGGTGCGACAGTGTCG

>c14987_g1_i1_len=548
GAGGATATTATTGCAAATGATTTCCTTAACAACCTTTCTTTCACTAATTTTGTTCAGTTAGCTACGCTTAAAGCCAGCGCTAGGGATCATTCTCAG
CTGATCTCTACCCCAGAAGACATAGATACCAATAGACGCTCCATTCTTAAATCATTGAATGAACGGAATAAAGGAGAGAGTAATAAGAGACCAAAG
GTGCTTGAAGCCCTGATGAAACTAGTTGATGAGTATAAGAGATCTGAAGACAGACCTGTTAGGCACGTTATGCAGTTGATTCCCTGGTGCTTGAGG
AAGCTGCAAAATAAGGGGTATTTTGACAGTGATATTTTCCCTAAGCCTCAGCACGGAGGAGATAGAGAAATACATGTTTTAGAGATATCTGCCCGA
CTTGTTCAGTATTGCCTTGAATTAATATCCAGAACAATTTGCAAGTTGTTCCCATCAGAGACAATAACTCATCCTGAAACTAAAGAAAGATTTGTA
AAGTATCACTATAAGCTAACATCTGATACATACAAAAAAAGTTTTGTTTTGTCAAAGTCTGCTGATGC

>c32637_g1_i1_len=214
CGCTATCAGCATCATATACTCCGCTTTGCATTAAGTTTGGTAGATGTGTATATGACCAAGTCGTTAATAAATCGGTTGTGAGTTTTAACGGTGAGA
TGGCTTCTCTGATACGACCATCAGTTGTATACCATCTTCCACCTATTTGAAATTTGGCTGGTAGAAGGGGTGTGCAATCAATTTGCGTACCTCTTT
TTTGTAAAATATGAGTTACTGG

>c7547_g1_i1_len=544
GTTCAACTTTGCCGACTTTCTCGAACATACATTCCAAAATACGTTCGGCTTCTTTCATAATTTTACCTGTAGGCTTACGCTTCCAGAACCGATCAA
AAAGTGCCATGTGTTGCATAACACGATCGGGCGTGCAAGGATCCCGGATGTACGTGTCTTCGAGATGTCTTGTCATATTAGGGTCTCTCTCTTTAA
GAGCAGTTCTAAACCAGGGGTCGAACTTTGGAATGGAGCTATTGAAAGCATATCCATCCATTTTTCCCAGATACAACATCGACTTTTCAGGACGAA
TACAGTAATCTGTTTTGGTGTATTGTGCCAAGGGATACTTTGCATCAAAAGACGTAGTGACTTTCGGAGCCACTGACGCGCTAGGTCCGTGGGGGG
TAGCTACCTGCCCATCAAACTCTAACTTCTTGAGATAAGGAACTACTCCTTCTGGAAATAGCTCTCTCAAGCGGATACGTCTCTCTAACGCGAGAG
CGCGTTGATAAACGTTTTGAATCGATAATAATTGGTCGCTCATGTGCTGGAATGTGTAATATTG

>c27686_g1_i1_len=615
```

GGGTCCCAGTGAAGACGTTGACCATATGCACCGGCGTCAGCAGTGATGCTTTCGTCTGTAATGCGATATTGGCTGATAGTTGATATAACATCGAAC
CGGGGGGAGATGAGTTCCTCTTTCTTGGCACGAGCACGTGTGTACATAGACTGAAGTTCTTTTTCGAGCTGGTCGACAGTCTTGTACGTATCACCT
TTCAAGATCTCTTCATCAGATGGACGGGAGCCATCAGACTTTGACTTAACATGGTATCGGTTACCATTATGTTCGATCTGTGTGTTAGCGGCAATA
GCACGTTTGGCAGAGGAGATAAAACCGCGGTGGTCTGCAATCTTCTTGATCAGTTCAAGTTTGGTTTCAGAACCTTCGTTGGTTACCGGGGCTTCA
TCTACACCAATATGTGGCACGTAGGGTTTGCGCGTCTTGCGTTGTGCTTCAACGGCTTCCCAACTAGCGGGCCAGTCATAAAGTGTGCAACTGAGG
AGGGCATCAACATCTCGAGGGTCGGACAGGTCATCGGTGTAAGGGAGAATACCATCTGGAATGTATGTTACTCTGAGTTCATCATCATCGACGCGA
CCTATGTTGGCAAGAGCCGACCATAGAGGTTGAAATACG

>c28765_g1_i1_len=401
GACAAGCCTCACTTTCATTTCCTAATGAGAGAAAAGTTCCTTAAAATGGAATGTGTTGGCAGGCAAAGGCCTAGTACGTACCGTACTGTTCTGTGG
GAGCGCCACGTTTTATTGCAGCGTGTATATACCGGACACGAAGTCCAGCCACGTTCGACAGTGTATCGGTCTCGACATAGAAAGTCGACCGGCGGT
CGTAGGGGAGGGTGGATCCTTGGAGCAGTAGAGCTAGAATCCAGGTGACAGGAGCTATCGAAGACTTTGGCATTTTACAAAAAACGTCAGATTGAT
TGACAGTCTCGACAGGAAGAACCCAGGCATCGGTACCTGTAGTCTCGACGGGCATGGAAAGACTGAACATAGCGACGGGAGAGAGAATCTCTAACA
TCTGTTCATACTCTAAC

>c35315_g1_i1_len=396
ATGTATGTTCGAGAAAGTCGGCAAGGTTGAACCTATTGATTTCAACTTTGAAGGTTGGCACAAGATTCTACCCCATCTTGACATGTCATCATCCCC
CGGCTTGCCACTTCGTAGGGAGTACCCTACACAAGGCGAATGTCTTGGTCACATCTATGACAAAGCAAAAAGATTGAACCACTTCGCTAAATTCCT
AGACCCGCCAAAGGTGCGTGCTCCTCCTTGTATGATCGGTCTCCGTCCAGGTCTACTGCGACGTGATGAACTAGATCAGAAGATCAAGGCTCGAGG
AGTCTGGGCATACCCAGCAGAAGTCAAAGTTATTGAGATGCGTTATTGCATCCCGCTTTTAGAACGTCTGTCTCACTGCTTCGGCAAGATACCTTA
CCCTGTTGGAAG

>c43767_g1_i1_len=338
ACACATTTCTGTCACAACCATGACCAACGTTAACAACAAGAAAACCGATGTTGAGGATAGCGCCTCAGCCCCCGCTTCCGATGTACAGGTAGCTAA
GGATAATGCTAAGCCCAAGGCTCCGCAGAAATCCAACAAAGGTGGAAAGAATAGTGGCCCCAAGCCCAACACCAGTAACGCTTCTGGCGAACAGAA
ACGTTCAAATTCGCAAACCCCCGGCTTTGCCGAAGGTAATTCAGTATTGAATGCCTACGGGTTAAGTGTCTCTGGAAGAAAGGAACGTGTTTTGAC
GCCGTTTAAGACGGAAAAATACTTTCATCTTGTCAGCGATTCGTGGCGTG

>c3606_g1_i1_len=492
CTGAATACAAGATCTCGAGCCATCGTTTCTGGTCCTTGGAAAACCACCCTTCCTCAGGACATGGGTAACAACGTTGGTAGAACTCCATGAATTCAC
AGAAAACACGGTCCCACATGGCCCCACCTAGCCACAGGCCGATCAGTCGGGTAAAAGAAACGTCCAACGTAAACACGCTGGACTCTGGGTACAGAA
CAAGCTTGAACCACTCCACCGTGTCACGGTGAGGTCTATCATCACGGTACTTGGTGCCGAGGAGTTTGAATTCACTCGGGTCTTTTGTCTTTTCGC
ACTTTTCAGGGTGCATCACCATTCCCGTTGGTTCCGCGTCTCGCTGCGCTTGCAGTAGATCAAACTGCTCATCGGAGCGGAAAGCACTGTCATCAC
CCAACACCTTCAGGTTTCGGATCTCTACAGCCTGGCAGTTCGCAAAATAATCTATCAGAATGAAGTTGATCACTGAGTCAATCATTTGCGTCCACC
AGGATCCGGAAG

>c17358_g1_i1_len=983
CTGAGGTTTTGGAGGAGTACCTAGAACCTTCTGCCAATGAGAGCAAACTAGCGCTCGAAGCTAACCCGTATGCGGTGCTTCACGACGAGTGCGAGG
ATGGTTGTTCTCTCAGCGGCCGGCTACGCGGTCGTGCAAAGAAACTGGTAAAATTTTACGAGGAGTTGGGAATGACTCGTTCAGCTGAAGAAGTTC
CCCAGCACATAGAGTGCGGTGGTCTCCGGGCTGCCGTACGACAATGTTTCGTCGCGCAGTTAAGCCCTGTCGACGAATTGAGCTTCAAGACAATAC
AGAAACTTGAGAAATCTTGTTGCAAGTTTTGTTTGCCTCGTTTCGAACAGAAGCTCGACCAGTGGAAACAGGCTAGGTTCCGACCAGTTGCTGTCG
ATGTGGAGCATTTGGGTAAGTTCAGACGAGCCCTTAGGCAAAATATCGAAAAGGGATGGGACCGACAACGTGCCCCTTTTATTCCAAACGGAAATG
CTACCCGGCGTTACCGGAGGAAAGAGGGTGGTAATTGGAACGTGGAAGAATTTTCCGGCGAATGCCGTTACGAGCTAGTGTTTTCATCGGGCAAAC
CCAGAGTTGTTACTCTTTACTCTGCCGAGAACACACGAAGACTCGCTCCGTTGCATTACTCGTTATACGACATGTTGAAGAGGCGAGGGTGGCTGT
TGGTAGGCGAACCGACCGACCAGCACGTTCAAGGCCTCACAGGCGCTAACTTCTTGAGTTTTGATTATTCTTCCGCGACCGATAATATTAAGCGGG
AGTACGTGAAGGTAGCAGTTGAGGTACTAGAAGAACAGGCGGACCATCTTTCGGATGATGAGATCCAGGCACTACGGGTGCTATCGAATCTGGTGA
TTGATGGCAGGGAGACATTTTCGGGCCAACCCATGGGGTCAGTTATGTCTTTTCCACTGCTTTGCGTGATCAACAAGACCGTAGTTGACATGGCAT
TGGCCGCAATGTTAGACAGGAAG

>c9501_g1_i1_len=6086
CCGCACCTAAGTGCTGCTCTTTAGGCGGGTGCATTTATCCCCGTGCACCGCTTACCGTTCGGGGTCGAAGGTTTGGCCGGACATCGTCTCACTGGG
TGGCCGTGTCCAGTGAGACTCACGCACTATGCAATACTGTACGATAACACAAGTACTCCCTAGCGACTGACATGGCAAGTGATTTAGTGCGTGACA
AGGTCAACCGTCTGCACGATGTCAGAAGAGCCCGTGTGTATCTAACCATGTATCTTGTTAAGGATGCACTATACCTGTTGTCCAAAAATAAACTAT
TTGGCACAGATATGTTATCCGGAAACAAGATGTGCTTAATTCTACTCATAAAATTACGCGTCTTCCTCATATACATTGGATACATACCATCGTTGA
ACACGCTCTTATCGACTAGCCCCACTGAGTTATCGTATACACGTCTGACCACTTCAGAGTTACGGTAGTGCAGAAACAAAGCAAGAATTGTCTTGA
ATGCATTGCAATCCTTCCTAATGGTTAAACCTGATACTGTCGATAAGCCGGTGAAACGCGCACTAGCCGCATTATCCAAGAATGTTACATACGATG
TTTTCACATCTAAAACCACGCGTTTGGGGGCTTCAGCAGGAACAACACCCAGTAGTGAAAGAAACTGGCGTTCATATGGCATGCCGTGTTCAGGCT
CGCGTATGCTGGTGATTTCGCGGTCAGAGGACACATCCATGAATTCGACATATCCTAGACCCCCGGCATTTGTCGGTGTTTTTAACAACCTGTCGT
ACTGTTTCTTCCTGAGGCTGCCTCCACTCCAACTGACCGAGTCGTCTCGTGCATAATCTATGCACATTTGCACGATGGAGGTGTCATTCAAACACA
TAGCCATTCTGCTCGCCAGAGCCATCCACTTCGAGTGTACTGTTGCGATGGAACTCTTCGAACTGCTGTCAAGCCAAGGATTAGCTAGATATATTG
ATCTCACTGTTCTAGCGGGATATGCTGATATTATACGCGGACCGTATCTGTATTTCAAAAACTCGCCCACATCCGAAACTGTTGTCTTGAGAGGGT
TTGTGGTTATCCCAAATTCTTCACATATGCTACATATCGTTGCGACATTATAGTACCTATTCGACATCATGATTATATCATCTCCTTGTACAATCT
TTTTCATGTCGACATTCAGCCTCTGATTTATGTATGCACACAGAAGATCTGATTTCATCGACCCTATTAACGACGTCCATTTCCATCCACTTAGTA
GTCCACCTTCATACTTAAGTGTTTTGTTATCGAATTCAACCACAAGTGACTCGATCTCTTCAATCTCGGCATCAATCAAGCGATCAAGCTCGAGAT
CAAGGTGATAAGGTGCCAAACTGTTCTTCAGTGTTCTCAACAAGAACAGTATGAAACGTTTAGAAACACAATGGTCAAATTTGGAAGCATCAACAC
ATATGTAGTATTCGTATCTGTATGGAGCCAGTGTTTGTACGTGTTCAATGGATCCAATGGTACTATCTAAGAATATAGGGTCACCCAAGACATACA
ACAAGTAACATTGACGCAGGTAGCTCGCCATCGGAGTAGTGATGACTGTCCGCGTCTTGGCTTCTTCTTTTAGCGCCACCCTCGCTATCCGTGGCA
TTTCACACGCCTCTTCATACACATCACGACCATTCAACAACTGTTCAAATCCCCACGCCCATTTTGTCTTCAAGTTGAACTCCTCACCGCCGACTT
CAAAGTTGCTACGCGGCCCACCACCAGAGGTGGCCCACTTGTTCAAGTCCGCACAAAAAACCTTGAAGCTCACCCTAGTTGTGCCGAGCTTGGCCG
AACACCAAGCGCCAACCCATTTTACAACTAACTCCTCCTCTAATCTCATCGCATCAGGATGTCTGTTTGTTTCGTCTTTCTCAGAAACCCACTCGC
TTATACTATCATACAGAGTCCCGGTACTCCAGGAGCGTGGTCCTCCGATGGTTGAGAGTCCGTGGAAACAGGATAACACGTAACATATCGGGGTCG
ACCCGCTACAAGACGTGAACACCACTTTGTCCACAGCCGACAGGTACTGGCGGATAGCGTCATCGACAAAAAGGGCAGGGACCCTTGATGTAATGT
GTGAAATGCGATCGTTTATTAGAGAGGATAAGTGTTGTGCTAATGGGTCTGTCATCAACGAGAAGTACGCGGAGAGGCCATACGTGAAGACTGATC
CTATATCCGCATCCCCTGTGAACATTCTGCATCTACGTAAAGACAGCAATGAATGATACCTATAATTTGGCATCAGCCCATGCTCACTTGCGTAGT
CGCTCACTTTTTGAACGAGAGCCTTCTTGGCGCTTGAACATAAGGTGCAAATGTTGAGGATGCAACCACCCAACATTGGGTGTGCTGGAAATTTTG
AACCGCACTCAAACGCGCCACAACTTACCACACATCTAGCGTTCATTATTTCGCTCAAATCGATGCCGTTGCAATCTGAAGACGTCTTAACATCTA
CTCCCTTACTGTAAATATATTTTTTGAGCACGCTCATGGCCTCAGTTAACATATCGACGGGGCTGCTAGTACACCACTGACGATTGCAACGTCGGA

ATTTTCGAATTTTTTCTTCCACCTTAACTACATTAGACCTAGGACCACAAAGTCCGATAATACTACGCCATCCCTCTAGACTTGAGCGGAATGTGT
CTAATACTAAAGGTGTCGCCGACACCACACTAAGATATTTATGATCTATTTCTACGCGTTCCACGCCAACATTCTCACACTCAGATTGTATCACAG
ACACCACATTCATAACCCGCTCGACATCAGCTGGTGTATACTCACACGCAGGATAGTACACACTCGCATCACCAGACATCTTGTACGCAGTGTATC
TTGGAATGCTGTCAGCCAGCCTGCGTGTAATACTACTTAAGAAGAACAAGCGCGTTTTTTTGCTCTCCTGAAAGCGCACAGATGACAAAATTCAAT
CTCTTGCCTCCGTTTCACCAGCCACGTGTCGAAGCTGGTCCCCATCCGTCAGACCGGCAACGAATTTCCGCTTGCCGCTACCTTCGAGCTTATCTG
AAAGCGCAGACACGGCTTTAACCACAGTGTCGATGCCCACAGATCCAATGGCTCCGAGCACATCACCACGTAGTAAGTGCGGTGTGGCAACGCGCG
CCTCACTTTTCAAGTACTCCCAAACATCACGCATCCAACTTGGAGGAGGATCAGGGTAATCCATGCTCACCGAGACATTTAATACTGATGCGCCAG
GTATGTTGGTCAATCCGACAATGTATGTCTCGATGGTCCTGTCTGGGTTGTGTACTTGTCGAAGACGAGGCATTGGTAAAGCTTGTGGCTCACACA
TCACCCAACTCCACGGACCCGGAGCGCCATTGTGTTTCATGTCGTCTAAGAACACATACGGCATCCAGCCGATCCTTCCCGTTTTAGGGAAACCAT
TCCATCTTCCGGGTAGGTATATTTGAGGATGTGCTTTATCCCAACCGGCAGCTGCCACATTATAAGCTCTCATTCCGCAAGCGGCGGGAAACTCAC
GCCAGTCAGCATCCTTAGAGGTGAACTTAAACGCTAGAGCACAGCCCATAGTGTCAATGTCTTCTGCGGCATCCCTATCAGTGATATCATACAATG
AATAAGGGCGTAGTGGCTTCTCTTTAAGCGGCGTCCACTGGCCAAACTTTTCCACGCACATGTGTATGAACCACCAGGGCACATGAACTGTGGGCC
ACATCTTCACCTCTGCGTCAGTAACCATCCCCGTGTATGATTTAACTGCGGAAATAAAACCTGTGGAGAGACTGTACCTATTTGTGCGTCCGCTAG
TATTACTAATGAACCCAGATGTTATTGAGTTCAACAAGATGGCACTATAGTTATCTACCACACCACGACCGGATGACGCATACAATCCATTCAAAT
CCATGATGTTATAACCAATCTCACACATCATCCATCCACTCACACCAGCCATCAAAGACGCTCCCGACTGAAGCGCGTTGAACAGATCAGTGTAAG
TGTTCCACTCGTACTCACACAATACTCGATCTCTGAAGCAACCACACTCTCGTAGCACACGCATTGGACCCGAGGTTTGCGTTACACCATAACCAG
TCACACGCTTAACAATACTATACAATGCAAACCTCGAGTTCTTCTCCTCTGTCGCCTCCGTCCATTTGTTATCGCACTGGAAGAGTGCGTCCATAA
GCTTACCTGTGGGTGTAACACACCAGTCACCGTACCCAGTCAGCCACCCGGACATTGCGTCCCACTCCTTTGCGAGATCCGTCGTTCTTGTTTTAT
CAAGCAAATAGTCGGCCGCATTAATGTCAAATCTTCCCACACCAGACGCCCCAAACACACCGTCTGTCCTCACCGCGCCACCCAATGGGACAGCGA
AAGCTAATTCTTGAACCATCGATAGCACGGTTCCCATGTCTCCTGACAGAGCAGTTCGCTTGCACATTTCTCTTACTGCCATCAGCATTGTGTGAG
GGTCTGGTGCCAATCCGTTCTCAGTGAGCCCCAGATATGTAAACACCTTCTTTGTAAAATCAGCGATCTTGGTCTCGAATTTGATCGCATCTCCAA
GGTGAGGCACACTGAATCTGGTTGACATACTATAGTCCTGGTCGACAACAACTATGGCGATCTTCTTCCATTCGCCGGGAACATAGACACAGCTGG
CACGTGTCATGGTTTTGATGACAAATTTGGATTTTGATTCCGAAGTGGACTTCGGGTCTGAAGAGAAATCGTACTCTTCCGTAGCCGCGCAATTCC
ACCAAATGGTGGAAGTGTGGGCGATCAAAAAAGCCATAAACCAATTCTGTTCAAGGTTGTGACTCTCGATGGCGACGATCTCAGCCTCAAGGATGT
TGAACCCAGTTAACCTGTCATTGTTATTGGGTTTAGCACATAACGCATTTACGTACGTGCTGAGGGGCACGATGCGCACGTCACATTCGGCGCAGC
CAGCGGGAATCTGGATGGTATTGTCCAATACTCCTCTCTGGTCATACGGGTGAAGTGTCTTTGTGCACTTTCCCTGGTAAGCTCCTAGCCTGAGCA
TCTTGATTGTGTTCGATGCGTACCCGGGTGATCCAGCGGGCAAGAAGGTCAGGAACATTAATAACATTCGAAGCAGGGGTGCTTCATCTCTGGCTA
AAGTATGCAGATCACCGTGGTATTTTGATACAAGTTGGGTCATCGCACTTAGATTGACGCTTCCCCCTGTAGGTATCAAATTACGTATCTGCATAG
AGGCATCCACCGGCGTAAGTACTGCGTTTGACCACAGTTTTGCTCCTCTGTCTGACAGCTCGGGAGGGTTCGTCCCCGCTCGCATCGTTCCAGATA
CCGATACAATATGCCCGGCCACCTGTATACGCTCGACGTTGTGTTCTACGTCAGCGAGCGTCCACTTGTATCCTATGACATATAAGGGGTCAGCGA
TATACTGCGGACTGAGCATATCACGCATGAGAGCGTAAGTACGCGCAGCACCATGACTCCTCCAATGCCCCGCACCTCTAGGTGCAACTTCAGGTA
TCGGACGGTTCCAAATGGACGCCAGTGCCAGGGCGCTCCCTCCATAACCACTATCTGACGAACTCGCTGGTGTATCCTGACGCCGGGTAGCGCCAC
CCGGTACAACTTCTTCGCTTGCAGACATGTTCGAGGAAAGTGTTATGTGTACGTCTGAGTTAGCTATTTGTTCTACCTGGTCGTGCGCTGTTATGA
CTGTTCCTGAAGCCGAGTTTTGCCTCACTCCTCTCACGTCACGTTCTGGTTTTTCAGTGTTAGTGGGGTGCATAAAAGGATCAGACTTCTCAACTA
GTGTGAAGCCCGTGTCTTCTGTTCCAATATAAGCACCCTCAGAAACACTCATTGATCCTTCGAAAGAAATCCGGAAGAAACAATATTGCGTTCACA
ATGTCACGTCACGTATGTAGACAGGTAACGTGTCGCCCTGAACCAATCTTGTATCTGTTTTTATGTCTTGTATTTATTTCTCTCGTAAATTCGTCG
CTTTGATCTCACTTACCGGTATTTTAATTGTGGGCCGG

>c51916_g1_i1_len=482
GCCAATGTTCGGTTTCTCGAGAACGATTTCTCCAAGTATGATAAATCTCAGACGGAGATGTGTGTACGCTTGGAGCAATTTGTTAATGAGAAGCTT
GGTTTTGACCTGGACTTGTTGGCAAAGTGGTATGCTGGTCACGAGCAAAGCACTATTCATAGTGTTCAACAAGCCTTGAGGATGAAGATTGACTGG
CAGCGCCGCTCGGGTGGTGGCAACACCGCGTTTGGCAACGTCACGGTTAATTTGCTCACGACGGCTTTCGCGTATCGTGGGACGCAGGTGAGAGCA
GCAGCGTACATCGGTGACGACTCTCTAATCATGACCGATTTGGTCTCTGCCTCGCAGAAATCAATTGGAATGTTAAGTGAGATGTACAATTTGCAG
GCTAAATTTTATTTATCTGATGATCCCTATTTCGCGTCGAACTTCCTGTTGTTCGATAAGGTTCAGAGGCAAGCTATATTTGCCCCTGATCCAATT
AA
>c9499_g1_i1_len=10684
AGCGCATCTATGGTATAGAAAAGATAATCAAAACTCCCTAACGGTGGGAGGTTAAGATGAGTTCTTTCCCCATACTTTTTCACATTAACGGAGGAG
GTTCGCTACCCCGGGATTTCTCCCTGTTGCCCCGTCCAGTTTTGAAAGCTCTGGATCGCTTTGGCTGCCATTTTATAAGAGCACTGGTGCCATCCA
GTTTTGTTTACAGGCTTCTCTTACCCATTCCATGGGGGGGATGCTTAAGACCATCAGCTAGCTAAATGATAAGATGGGCCGCCACCACCATCTTAG
ATCAGGTACCTATGATCAGAGGTTTTTGTAGGACGGAAGCGGGCACCCTCCTACGGGGGTTACAAGTGCCCTCCCTGTTACACAGGGGACTGAGGG
GAGCGGGTGCGTCACGCCCGACACAAATGTGCCCTGCTCGAACGTGCTCCATAAAGGAACACACCCTGTTGCCAGGGGACTCGGGGGAGCGGGTTC
GTCATGCCCGACATTTTCATGCCCTGCTCCTGTGGCCGGGGGGATGCTGGTTCCTCACGGCTCTATTCGATTACGTCTAGATGACATCATTTCCTC
TTCGATATCGACTCATAACTGAGAGCTCGTCCTTGTAGGTGTTCAGGACCCCAGTTTCTGCGTCGATTATCCTCCGAACGCTGATGACACTATTTT
CAATCTCTCGTGCCCACGTTGCGCGCTCTTTGACCCCAATCAGGCTTCCGCAGGTTATGTCCTGTTTCTTGTGCAGGTAGGGGATGTCATCCCAGG
ATTCAATTGACTCCTTGTTTAGCATCCACGGGTTGTTGTGCACCCACACGTCATTCCAAACCCGAAGCATGTCATCAGTGGTCATCCACTCGTGAT
ACTGGTGGACTGACCACGAGGTACGTCCTAGAGGGAACCAATTGGTTGGCACAGCCGACGAGATAGCAATGAAACCTAGGCGTAAATCACGCCTGT
GGAAAAAATACAATGCCCACATCTGGGCATAGGCCTTTGCCATGCAAGCACTCTCCGATATGCTGACTACCCCTCCCTTTTGCAATCGGCTACGAC
CAAGCACTTCGTTCTCATGTCTGCAGGGTGCTATGAGGATTCTGCCGTCTTTCAGGCACATTTCATGGTAATGGTGAGAACAAAACTCCACCTTTT
CCCAATCGCTCTCCATCCTGGAGGGAGCTCCCTGTGGGACGTTCTTCCGGGTTTTTCCCGTCAGCTCGAGATATTCGAGAGCAGATGAGAAATCGC
GATTGTCTGTCGACACAACAACGTCGTCTCCAGCAATGACCATGTTTCCGAGAGCTTCTTCAAGGTGTTTTTCCAACCACGCATCCAACACCGATT
CATCCGCGTGCAGTAGTCCTTCACTTTCTAAAACCCTCGCTATCTGAACCTTACCGTTTGTTATGGTGTTCAGCGCATATGTGACCACTTGCCCTG
ACCCACGTTGGTCTCGACGGCCAACGACGTCCATCACCGTGCCGCTGCCGTATTTTGAGTGTGTTCGTGGGAACATGGCCACAATGTTCTGATATG
CCAGTCGCATGGTAGCCGCCATCAACGCTCTGTGGTGGGGGTCTTCCGTGAGGGCTGTTAGAAGAGCTTCCTCATCCTCCAGATCCTCCTCACTAA
TTTTTGTGTCCCATCCAGCAATGTCGTCCGCGAAAAGGTATTTTCCTCTGCTGGCAATGTCCTTCAAGTAGTACCCGAAATAATTGACACCAACGC
CTCCAACTCCCCCAGGGAAGTGCTCCCTACTGACCCAGTGATCCGCATTAAGGAAGCCTAGAGCCTCAAATTCAAGGAACCGGCTCCCAAGCCACA
TATACCAGATGGTCCTTGAGCCCTTGGCCTCACCAGCTAGGCTGGGCTTTTTCTCCTTCTTGCCCATGGTGTTGTAGATACACATTTCACACTTCC
CTTGCAGATGGAGGGCCCGTTCCTTTCCAATGAGATCCCAAAAGCACTGGTCCTGAATGGCTTCTTGTACATCTCGCCATGGCACATCCCTGCTCC
ACGATCCCACAGCCGCATCATTTCGAACGTTGGTGATGAATTCTTCTTTTGATAAAATACGCGGTTGTAAGTTGCGCTTTTTGAAGAGCCGGATAA
GATGCTTTGTGATCGTCCTGTTGACCAGCCGTATATGCTGTGGGTGGGGGGGGATGACAGTGTCCACCTTCTCCCTCAGCACCTTCTGCTGTGAGT
AAGTTGACACGTCAGTCATGGAAAACCCCACCACACTCGTGAGGGTCTCCCATGGCCACATGATCTTCCTAACGAGGGGGTTCACCGCTTGCCCTC
CCACCTTCACATCATCGGTGACAAACGATCCAAAATACTTGAAACTCTTGTAGGGGTGTTGTTGATCTTCAAACCATGTTCCACGGTTCTCGTTCA
TTAAGCGTTCAACACGGTCCATCATGTCGCTAGTGTCCATGGATGCCACACTAGCACCCGGGTCAGCCCGTGTGCCTTTGGGCAACACAGGACTCG
GGAGGACAACGCTGTCTAAGTGTCGGGAGAAGCGGGCCATTAAAGCGGCCAGCGTCATGTATGTGGTGCGCACCACGTTAGACCTAGCCCCGGAGA
TATAGTACAGCTCAGCCGAGCTATTCCGACTGTGGGAGAGACGCACCAACCTGCCATTGTACAGATGTTGGAGGGACTCGATTTTCCGGAGGACCT
CAAGAGAATATGGTGATAGAATCTTGCACACAAAATGGGCTCCCGGGTTCGCCAAGAGCCAATTTTCTAACAATTGCAAGACTTTAAGAGTTCTGG
TCTTCTCCTTGATTGGGCTGGGATCACTCTCTCCGATGTCACACATAATGGTGTCACACCGGAATGGGGTGAGCCTATGCACATCCACTCCAGTTT
TTAGCGTAGCCAGGTTATATCCCTTCGTGACGAATCGCTGAGGATTCTCTTTTTCCTCCCCACCTATTGTGAACCCGAGAGCGGAGGACACCGTTT

CCTCCATCACAGCTCGCTGGCTCCAGCCACCGCGCCCACATCCCAAATCCACCACTCGACCACTCGGTCTCCATTCGTATTTTGAGATGATTTCAT
TCAATTTGAGGCCGCCACGAGAGACATAATCACCCCGCTCCGTCTCATTTACCCCGCGTGATTTGTATTTTGTGAATGCATCTCCATCAAGAGCAT
TCAGAGTCATCTTCCATTTCATTCCCAGACCGATCGTTGTTGACTTTTCCAGAGCTCGAAACTGGTTGAAGGGGGCTACCGCGCCCAAGGCTCTAG
CGGCAATGTGCATGAACCTATAAAAAAGGTAGTTCATTTCTAGCAACCCTATGGCAGACACCATGGAGCCAAAATCCATGACATCATGGATCTTTG
ACCGGGGATGGAGAATTTGCCACATGCTAGCCCCGACCACCAATGACCCTCGGAGGAAATCTCCAGGAGACCGTGTGCAGAAAGCCCAAAACAGGC
TGAAGATCAAGCAGAAGGCATACATGATGGACCGAGTGTTTTCGAGTGGCACTCTACTAGTGATTTCAGTGGGACGATCATGTCTCTGGTCGCGAG
CGTCCAATGCTGTCAAGATCTTTGGGGTGGCATTCCACTTGTTCTGACAGTACATGATGGCCATGAACACACCAGCTGCCAGAAGCCCCACCACCA
CACTTGAGAGGCTAGCAATCGCGACAATGGCAAAACCAGAGGGAAGGACAGCCCTGAAGGGAATTTCATCAAATCTGAATCCACCAATCGAGCTAA
CCATCTCCGGATGTTTCATGGCGTACATTCGTGCTGTCCAGTTCTCCTGGAACCAGTACAAGAATTTTGTTGACAACACTGTTATGAAGAAGAATA
CCTGCAGAGAGTCCAACATTGTAGCTCCCTTTCCCCGCGCTATTTCCCAGCTTGGGAAAAATGGCCCGGAATTCACGACCTCCTCGGGCTCCTTAA
CAGCTTTCACGAGCTCCATGAGGTCGCCACGTATGTTCGGGAACATTCGCATCTCCCAAGCCAGCACTCCGCAGATCAGACATGATCCTAAGATGA
CCCACTTTACCAAATCGCTGTCCATGTAACTGCGATTTCCAATTGACTTGTACAATACTGGGTACACCAAGAACATAGCCAGTGATATGACGACGC
AGAACCCCAAGGGAACTCCCATATAATAAAGGAGGCTAGGGGTCGACACCAAGACACCACCGCTAAGAGGGTCACTAGATGGCATTTGTTCATAAG
TTACCTTTCGCGGCCTAAACAAGGAGAATAGGAAACACAATGCCCAGACAGCGATGAAACAGGAGAGCACAATTGAGACGCCCAAGACAAAGGCCA
TTAGCACGGTAAGGGAGCGCTCAGTTGTGGACCGGTCACGCAGTTCTCCATTCATGACGTCACTCAAATCCCAGAGAGCAGATGTCGTTCTCCTCC
ATATATCGGTCCAATCAATCAATGTGAAAAACTCATAGAGGCTAGGCCCCCCGCGGCAGTTCATGTAGAAATCAATGGACACCTTTCGCGTCTCCC
ACTCAATGCTCTCAAAGCGGTCATCAACGTATTGTGGTCGATACTCTTGTTTGCCAAATCGGTTTTCGATGATCTTTCCGGTTCCTTCGCCTTGAA
ACAGGGCTGAGTGCCGTCGGTCTCCAGCTTCTGCCCAGTGCCACGCCAACCAGATGGGAACGTCATCCCGGTCCATAAGCTTTGAAAATCGCATTC
TATCCTCTCCCACTAGGGTGAATCTCCCCGGGGGCTGGCTGTATGCGGCTTCCTCTAGCATAAAAGTCATGCCGAGCTGGTCCAGGATCATTTGGG
CTTCAGGCCAACACACCCATGACACTGGATTTTCCTCCGTTTGGCTATCAATAGGATACACATACGTTCCTGGTTCCCGTCTACCCGTCCTCCCTC
TCCTCTGGATCATGGATGATGTGGTTATGTTCGTTTCAACCAGCTTCACACGCGTGGCAGAATCTACAAGGGGTCGCAACGCTCTTCGAGTGTCTA
TAACCAGATCGACGCCCAAATTCGCTCCCATTTCTGAGATGTCGGTGGAGATTACCAGGGCGGTGGCTGCATCACCTGCTTGTGCATAGTTAGTGG
AAAAGTTTGCTCGATGCAATGGAACGGATCCAGGAATAGCACTAGCCAGGGTGTTAGCTTGGTTGTGCGATGGGACGAATAAAATGGTCTTCTTCG
CCCCCACGGATTTTTGAACGTTTCCAATCCACGCTGGACTCAACCAAGTTGGGAAGCTAATGGATTGGTCACTTATTGCATAATTCGAGCCTGTAT
CAGGCGCGTGGCCGGGTGGTGTTGCACTAAGGTAAATCAATTTTGTTCCTTGGCTGTGGAGATGTTCCATGATCCCGCGCGCAGCAATGGACATTG
GATCCATGAAATGGCACTCATCCATGATGATCATGGCAACCCGCACTTTCTTGGCGCCATGGCTAAGAACAAATTTCGTGAACGTTGCGTGACATG
CAACCGTTATCAGATTCCTGGTGCAGTACGATAGGTTCTTATCACTCCTCATGCCTGAGTTCTTCAAGGCCTCAACGACCTCAGCCATGACCACCC
GGGTGGGTGTTAAGATCACGGTCCGTTGGTTGGAGTCATAGTTCTTCTTGGTTTCTTCGACAATAACCTTGCGCGTCTTTCCTTTTCCTGGATGCC
ATGTCACGAACCGCCTGGTCGAATCACAGTTCAAGTTCTCCGTGACATCGGTTGGTTTTCCACCTGCTACTGTTGAGCGATAAATTCCGTTCACAT
AGAACCCAAAACCATAGAACCCGACGGGCTCACCATTAATGAAGAACGGTGAGCCAGAAGATCCTTTTCCGAAATCATCACTAATGAACATGACAG
GTTCACCGTCAATACTCATGACTCCCGGCCTGTACCGATGGTACTCAATGGTCTTGTCCGGTTTGCAAGCCATCAGCACGAGGTCTTCCGTAGTAG
TGGGGGTGTCCAACTGCCAAGGTCCACCATACGATACCACGTCGCGCAGGGCACTACCAGAGTGAGGAACGACAATTCGCCCACGCCATTTAACTG
GTTCACCTCGGGTGACGTGCATGAGAGTGTGGAACACCCCATTTTTCGAAAAGCCAGCCCCACGTTGCCGGTCGCCAAATAAGCTGCGAACAACGA
TGCGATACACTCCATTGGGAATGTCTTGAAAGCTGGTCTGGAGATCAGTTTCAATGGGTCCGCCGAGTCCCAAGAGATCATCAGATCGGTTGTTAG
CAGCGAGCTTCATACGCAGCATCCGTATTATGGTCACGGGAAGGTTAGTGCTGGTTGCGATGAGCAGGTAGAGTGCCCCCAAAAAGTATGAAGTGG
ACGTTACGGCAAAGCCTAGGAGGAACACAAATACGAGCTCGGGTAGGTAGCCAGACGATGAGGCCGCCCCCACGACGATCCCATCCTCCCCATAGC
GCCCTTTTAAGTCAGGAATATTTATCCCGTCATCATTTTCGGTTGACACTCCTTCGGGAGTCTCTCCAGCTGAGATGAACTCAAGCTCCATACTGA
CGTTCGTCTGTTCCATGTAAAGAAAGAGCACTATCATTGCAAAGGCTAGGACAGCTGCCACCGTTGGTTGGTGAGCTTTCTCCGCCGCCCAGATTC
CCCCAGCGCTCGCCAAGGATACAGTCCAAAACCATAAATTCGCGCCAGAGTCTCTGTAGCCGTTCCTCGGATTGTGATATTGACATGCGTACCTGT
ACATAGCGTTCAAACTGAAGATCAGAAACGCAACGGTAGCGAGAGACAGGGCCGTCTTCCACCCCATTATCAGGCTGAAACATAGAACCTCTGGGG
GGAGAGCCTGCAGGACGCTCTTGTAGGCGCTCAGCAGGACCACCGAAGCCATGAGTTCTAGGAGACCGTAGAGATATTTCTGATAGCGATCGACGG
CGGAGAACAGGAACTGTACGCACATCAGAGTCACCAGGTGGTGGACCCCTAGCACTATTGAGAGTTTCTTCCTGATCATGTACCCCAACAGCAGGA
CTGCTGAGCTCCCAGTCTGTAGTATGATCCAGAGATTCATCAATAGATCTTGCGGTTGCGATGATGAGGACGCCACTAGCAACACCCAAGCAGACA
GCCCCACAGATGAAAACAAATTGGGGAGTCCAAAGAGCGCCATTGCTAGAATCGTCAGAAATAGACGAGTTAGTGTCGATGTTCTGGTTTTTGCCC
CGATAAGCTGCAATGAGATGGCGAGGCACAGGAGATTTAGGAGGGAGCGGTCGAACCCAACTCCTGCACGTGGGCTCCCCCTCCGGAAATCCGATT
GAGCGTTCGCCTTCCCGTATCTCTTCAATATGTCGTCAACTGTTGGTTCTGGTTCCTCGCCGATCGGTTCCTCAAATATTTTGGAATTCGGCTCTG
ACGCCTCAGACATGGTTCCAAGTTGGATCTCCATGGGATAATATAGGAGGTCACCGACTCGAAAGTGGACTATTCTTGTACATGACTGGCAACACC
AAGATTCCGTGTCATTCGGTCCAATTACCTGTGCGTGCATTCGCCCATGACATCTAGAGTCCACCTTGACCTTTGTGCCAGGAGCTGGGCCCTCTA
TGAGAGTGATGTCACTAACGTTCCACGGAAAGTCAATTTGTGTCTTGTACCCGATTATGTGGTTCACTCGCGAGATTGGTCCCCCCCAAGCAGGTG
GAATAAATAAAGCTTGGTCTGAAGGGTCATTGAGCGTGTCTGGGGTGTATTCCCTGGGCCACACACAGCGGTGTGATTGACGCATCTCGAGTGATG
TAATCTGGGCAACTCCATCCAACACTGTGCTTTCCATCCAAAACATGCCGTCAGTGATGACAGTTCGGCCATTCTTCACGACAACGCCCGCCAGGT
ACGTGGGACATTCGGTCGATGAGTTCTTTGAGACCTCAACCTGAACGTTTGAGCCATACAGGCCTCGCCGGAAGCCCGTAAACTTGAATTGGAAAG
AGACGGCTTTTCCGCAGAGTCGGCGAATATCGGGCCGCGATGCGACAATCCGGATAACCTTATCAGCCTCTTTTTCGGGATAGCTTCCAAACATTG
TTGGTAGATGCCCCAGATCACCCTCAGCGCGTCCATGTGCAACGTAAGATGAGAACTTGTACTGAATTAGGTTAATGGTGACGGAAATGGTTCTCT
TTGGTGTTTCGGAGAACTTGAATGTACGTGCCAATGTGTCATTTACATACACCTGTTCAGGACCCATCGACCCAACAGCAGAGAAAGCAGCTCTGC
GCAGGGCCACGCACTGCAAAGCATCCTCACAAATCAAGCAGGGTTTCGTGGTCCAGCTAAACATATCCTTTATGTACAAGTCAGTGAACGAGTAAT
CTTCAAGTTCCACAGCATGATCATTGGAAATGCCAACGCCCAAGTGTTTCCACACAAAAGCGCCGCTACCGCACGAGATGGTTTTTCTCGTGGTGT
CAATTCCACACCCCAGATCAGCCCGCACATAGTAAAAATACCCCCCTAAAACCAATAGAAAGGCTATGCGTTTGTCAATCAAGGCAGCGAGGACGA
AAATGACCAGGAGGGTTGTGTTGGAGAAGAAGGCACCAACCGAAAACTTTCCAACCAGATCCTGCCACACCCCCGCAACACCAGACACGCCAAACC
GTCGGTACCGATCTACCACGTGTCGAGCCAGGCGCCATCCTTGCGAGATAGGGTTAAGAGCACACTCAATAGAAACTGAAGTGCTCCCAACGAGCA
AGGTGCTTGTATGATTCCCGCACTTGACAAATGCCCGAGAAGTGGTCTTCCCTGCGAGAAAGATCATTTTTGTGGATTGCAACAGACAACCGTGGC
ACGTTAAGCTAAGCGAACAGGTCTCATTAGAAGAAGCGGTGAGGGTAGTTATCACCACTCCACTATACCCGAAGCCGTCTTGGGAAAATGTGGATG
TTTTTGGGCCTGGACAGTCTCGAAAAGCTCCACTGACTAGTTTGTCGAGCATAATCTGACAAACAAAGGAGATGTCACGGAATCCATCATGGGTGG
CGATCGCGTCTTCCCAGGACAACGAAGGTTCAATTACGTTTTTTACTAGAATCTCATTGGACCTAGCATCTCCCCACACAACAACTTTCTCCATGC
CGTGCCGACCACTAGCGGTAGAGTGCACGCCAGGCCAACTGTCAACTTGAGACCGCAAAAACAGCCCCTCGTGAGAGCTTCCCGTGACGTGATAGT
AATCAAGCAGCAACTGCTCGGACTCCAACCTGCAGGTCATCGCCGCGTTTCCGAGTTTCGCAAACCTAAATGTAAGCGGTATGTCGCCAACCATCT
GTTGTGTGTCGCTAACAGAGTGGAAGCTAGCCGTAACGTTCATAACAATGGCTGACCTAGCGATAGAAGAAACGTTGAATCCTCTGTCGCAATGGA
GTTCGACGCACGTTCCAACGAATCCAATCCCCCACTTGAAGCACCCAGTGCCCCAACCGCGGTTGTATGGTTGGGTCGAGCATACTCGTTCCTTGG
CCATGATGTCAGCCATGTTGAGCTGCGATCCACCGGGACACACGTCAGTCGAATAGGATGCGTTGACGTGGCAGTCTGCGAGCAACTCGCGCAGCC
ATTGCCCCCCATAGATCTCCGCCGGTCCTGTTTTAAACTCGAGAAGACCGTTTGGCGTGGCCACGACGTACCCTTCCTCCGGTCTCACAATGGTCT
GTAGCATGGTCATCTGTTCCGCTTTCAGCGTGTATAACGGTTCAACAAACTCCCCCTTTACTGTGGTCCAAGTGCCAATTGCCAGAAGAATCACAA
CCCAGGTGGGCCAACGCTTCGCGATTGCCACACACAGTAGGGCCACGATGAAAATTGTCTTATTTTCGCGAATGGTCTTGAACGCAATGATCTCCA
TTTCAGCTAGCAGATCCGCTTTCGGGGGTTCACGTGATCGCTTTTCACGTTTCTTCACGACGCATCTCCGATACCGCAATGTGAACTCAGTCCACG
TTGATCCGCAATCCGTCGACTCGAGGTCCTTTGCCAAGTCAGTCACTTTTGGGCACTTCTTTGTGACAAGAATGCCACTCCAGCAACCATCAGCGG
GTAACTTGAACAGATGCAAGTAGTCTGTCCGGTTCGTCTTTCCTTCAAAAATTTCCGTGCCCTTAGCGGTAGAAACTTGCATGTCAATCACCACGC
ATCCACATAACACTGTGAGAAAGCCCAAGATGAAAGCTAGCGTCATAGGATTTCCTGTGGTTCGTCGAGATTCAAGAGCGGTAACACGTCGAGATA
ATGAGCGAATTCGCCGGTCGATGGAGGCAAACAGGGCATGAAGCACCAATGCCATGTGAATGAGGGCTTGGACGATGTCCTCCTGCAAGAAGGATC

CCCATCCAACTCCCAGCCTTCCTAACAGGCCAACACGCTGTCTTTTCCCTGCCCTACGCCCACTCTGGATGGGTGTTTTCAGGGTCACCTGCCGAT
TTGAGGGAGACTCCCCTCCCTTTTCCTTGTCCTTTCTCCTACCTTCTCTTCCCCAGAACGGGGTGGAAGAATCTCTCCCTGGCGCTTTTCCTCGCG
CCTCTAAATCCTTCCTCTTCATTCCTTGCCGGGTTCAACCTCACGGTCTCTCGGCAAAAATTAAAAACAACTTAAGCTTTGAAAATATAAGCGTAA
CCAAAACTGTGGTGTAGCTAAGCCGAAG

>c11413_g1_i1_len=693
GATGACTGTGGCGAGGTATTACTCTCGACATGCAACGGCGCGAAGGTCCGGTTCCGCCTTGAGCTTCGTGCTGGCCTAAAAGATGTCATACAGACC
GCTTATCAAGCGTTCCGTGCCTCCCACCCTTGGCACCCACCCACTGGTACCCTGGAGCCCTTGAGCTTCCAGGAGTGGGTTAAGCGATATGACCGG
CCAAAGCGCAAATTGCTGGCTACTGCTCATGCTGATCTTGTTCATTCCAACATGTTAACGAAACGCGATATGAACATTGGAAACTTCGGCAAACTG
GAGACCACCACCAACGCAACCACCCCACGCAATATTTCACCACGTAAGGACCATGTCTTGTGCCTGTTGGGCCCTTACGTTAGCGCTATCGAACAG
GCGGCTAAGCACGCACCGTATCTCGTCAAAGGCCTCAGCCCTAAGCAAAGGGATGAGCGTATGACTCCGTTGACATACTACGGCCAGGTCTTGGAA
CTGGATCACAGCCGCTTCGACAAGCACATTTCCGCCCCAATTCTCCGCGCCGTGGAGCGCACCCTGCTTACAGACCCATTCACTGGGTCTCACTAT
TTATACCATCAGGCAGTGGATATGCTAACACGCACTAGAGGATTGAGCCGGTACGGCACTAGGTATGCCATGCCAGGCACGCGCGCTTCGGGCGAC
GCGCATACATCCATATCTAAC
>c40769_g1_i1_len=1259
GCACTCATGTGTCTGGAGGGAAACGGGTCACCATCGGCTATGCGTTGGGATTAAGCAATTCCACAGCGTTTTGGACCAACACAAATCAGCGCAGAC
CGCCTAAAATCCAATACATCGGTCCAAATACGCTAAACATGACCTACACATACTACCCACAATCAGCTGACAATGTCGAGGCTGCCTGGATGGGTA
GACATAATCCGGTCATCCTGAAGGAATATAACGCCTCATTATTTAGCTTCATAAAAACCAATAACCACCTTAAGAGTTTGTGTTCTCAAGGTGAAG
TAATAAATAAACTATCACGTCAGGAATTCATCAATAATCAACCTCAGGATAAGCGCTGTAAATACATAAAACACATAAACAACCACACCAAACCTA
GCAACATCATCACCTCCTTTATTAAAATAGAAAAAGTCAAATGCAACAGCTACCGAGCACCAAGACTCATACAAGCTCGGAATCCCAGGTATACAA
TGGACACCGGTTGTTACACACTACCGATAGAGAAGGCTATCTATCGTAAGAACTACAACAAGATCATGAAATGTTACAACCAGTATGAAATGGCCA
CCATCGTTATAGACAATGCCAGCAGATATAACGATCCATACTACCTTTGCCTGGACCATACAGCATTTGACGCCCATGTTACGAATGACATGCTAA
TGTTGTGCCACAAGATCTTAGCTACTTATAATGGTGATCCTGAATTGCAAAGATTGTTGCGTATGCAATTGAAGAACAGGAGTTACACCCGTGACG
GACAGTATTATAAGTGGAAAGGCACAATATGCAGCGGTGACATCACCACCAGTCTGTACGGCTGCTTACTCAATTATTACATCCTGAACCATGCCT
TAGATAGACTCCATGTAGGGAGGAGGAATGTAATGGTGAATGGAGATGATAGCATAGTCATTATAGAATCGCGAGATAGGCGTAAGTTCACAATCA
GGAAACTACTCGCTCTGCTGAAGGAATACAACATGGAAACGAAACTAGACATCGAAACAAGCGAACTATCTGATGTCGAGTTTTGTAGGAGCAAGC
TAAGAACTAATGATGATGGTATAGCAATGTTCGCAATCGACAAAACAAGGCAGGAGCAGGTATACGGTATGACGCATAAGGTAGCGTCCATTGACT
ACAATGCTTACCAGCGGGATATAGCATATGCCAATATGACCCTATACCAGAATACACAAAGAGAACAGTGTTTCAGAACCTTGTATGAACACTATA
GTCGATTAACA

>c18294_g1_i1_len=339
CGGCCCTCGCGCGTCCTTCCTCCGGCGGCCACCCGGGGTCCCGGCCCTCGCGCGTCCTTCCTCCTCGCTCCTCCGCACGGGTCGACCTGCAGCCCA
AGCTTGTATTCTATAGTGTCACCTAAATCGTATGTGTATGATACATAAGGTTATGTATTAATTGTAGCCGCGTTCTAACGACAATATGTACAAGCC
TAATTGTGTAGCATCTGGCTTACTGAAGCAGACCCTATCATCTCTCTCGTAAACTGCCGTCAGAGTCGGTTTGGTTGGACGAACCTTCTGAGTTTC
TGGTAACGCCGTCCCGCACCCGGAAATGGTCAGCGAACCAATCAGCAGGGT

>c38830_g1_i1_len=286
GCAAATATTATATTGTTATTACGGTATTTGCTATTATAGGAGGATTGGTTCAAATTGTATTTATATATAATTTTTTTCATAGTATTTTTTATGGAA
AAAAAGCAGTACAAAATCCATGGAAAGCCAATACATTGGAGTGGACTACTCCTGTAGAGCATATTCATGGGAATTGGCCGGGTGAAATTCCAGAGG
TACATAGATGGCCATATGATTATAGTAAACCGGGACATGATGAGGATTTTGTTTTACAAACAGTTCCAATGAAAGAGGGTGAGGAAGAACTTCA

>c9445_g1_i1_len=1618
ACTTCTTGGACGGGGATTGGAGGGCCCGTACTCCCGCTAGTTGAGTACTAAAGTACGGGGGTTTACTGTTTTCCTTCAGTCGCCTTTTGTTGGTTT
CATTTGGACTCAGGGGCCCATTTCTGGGACACAACAGGCGCCGGTGACGACGAAACTTCTTGGACGGGGATTGGAGGAGGGAGAACAAAGAAAGGA
TGCAGAATGGTTCCTCTATCCGAGATGCCAGTCAAAACACGCAAAACTTTGCTCTGCATTTCCAAAAGTTTCGGATCCCTGAAGACGGTCACTGGC
GCTGGGAAAAATCCTTGTCCCACTTGCTAAAGCAGGCACGCGGTCTCACTCCCGCCAGCACACCCAATCCATCAGCTTCTTGATGGATATCCTCTA
TGGTGTCCGATTCCTCTTGGCCACCAACCGCTCCTTCTTCCTCTAAGGACGCTGTTGAAGCTTGGTTTGACCCAAGATTCAACCGCGAGAGAGCTC
GCACAAAAGCCTCCCACTCCGGCAATTCCCGAGCTCGCAGCGGAATCATAGATTCCAAGGTTGCTGGAAGCATCCTAGCTACGAACCGGGGTTTTG
CGGGGGTTGCCACCGCTGAGGCAGCGGTGTACTTTATCGCCTTGGTGACATCACCAGAGCGCACGTACGCCACCGCATCATCGGACGTGGCGAACA
GAAGAAGCCCTGGCGTGGCATACCCCACCACTTTGGCGAAGAACTGACACGTAGCGGTCTTCTTACTTTCGTCCACGTACCGAACGACGGTACCTC
CCGACGGTATATAGTACCTGTCGCTCCGAGCCGAGCTAAAAGGCACCATATCACCACCGGAGTGCATCTTAAAAGTCAAACGCTCCGAATTGAAAG
AGCCGTCCGAGGTAGTAAAGATATTACTCCAGCCAGCATCACAGGTTATGGTATCCTGCAATACCGAAGCTGGAGTCTCTGCTCCCTCGAATTCAA
CAGCCCATTCAAGGGTAACGTTGACCGTGATGGTACCAGTGAAACCTCCGGCTGCCGCTCCGACCACTCCCACCAAACTTCCATGGGTGGAAAGGC
CGACCTCGCCGTCGCAGAGGTACCACTTGGTAAGCGTTTCAGTGGGTATTGAGAACCTTTTGCTCTCATAAAGCCTCATCTGAACGCTAGGCTTAA
ACGCAGAAACCCTCATAAAGTCTGCGGAAGTGTTATTTGCACTCCAGTGCTCATCTGGAGTCCAACCCACAACCAACGACCCATAAGTGGTCGCAC
ATCCGGAAGCGGTAACGTTAACCACTAAACCTCGAGGCCGCCATCTTGACCAGAGCTCTGACTCAGCTTTGAGCCTAGAGTTGGGCAAATCAGACG
GGGCGAGAGGTTGTATAAATGCAACCTCCCCGACCTTTGCGGTAGCCTTAACACCAGCCGTAGCTAGCAAGTCGACTCCCCGGCTGACTATGACGG
GCATCGTAGCACGTGGTTGACGCACCACTTGCCTGGCCGGCCGTGCCCTAGTGGATTGCACGGCCTGGCTATGAAAATCTCTCCTGTGCTGCGCCA
GAGCAGCATTAGTCTTGAACTGGCGATTGCACTGTTGACACTTCATCTTGTGTGGCGCTTGATAATCTAACACTTTGTAGAT

>c9477_g1_i1_len=2761
AAATCAAGTGAGAAAGCGTTTAATTGTTCTATAAATTATTAAATCTTTTGCAACTATGTATTCAGACTTCGGGGAATGCATGGGCTTGACTTTCAA
GCTAGCGAGCGGATTTTTCCATGAATACTACAGCGTGGTTCCCACGGTGTTGGAGTCTTGTCGTAATCTGTTCGTAGAGGAGATCGTAAAACCACA
GTTATTGTCAACGACCGTGGAGGTTCGGCCAACCAAATTGACCATAGGAGTGGCTGTAGCCGCTGCGGCGTATAGTGCATTGGCGATTGGTAGGAA
GTTAAAGAGAGCTAAGCCGCCACGTTATGAGAACAAGCCGCCTAACTACTTTAAGGAAAGTTCCTACCCTGGTTCGCCCATGATGGATGCTCCAAA
CATTCCACCAGGGCAAGTGAAATTGGCGATGATAGGGAAAGACAATGTCATTCGGCTTATAGGCAGTGGTATACGCGTTGGTGAGTATTTGCTCAC
TCCTACCCACAATCTTATGGACGGTCAGGACATCCATATGATAGGAGGGGCCAAGGATGCGGTTCACGTAAAACTGGATATGGAGCATGTAATAGA
CATAGCTCCAGATGTTTCGGCAATCAAGTTTACTGAGGCTACTTGGTCGCAGTTGGGCATATCTCGCGCGCGTTTGGGCTGTGTCTCAGGGAAGAC
CACGGCTCAGTGTGTGAGTTCTGTGGACATGCGAATGTCAATTGGAACTCTGAACATTGAGGCCGATGGCTGCCTTGGACGTCTGTTTTATCAGGG
ATCAACGATCGCTGGTTATAGCGGATCAGCCTATACCACTGGCAATAAGGTGTTGGGCATGCATACGCATGGTAAGGAAGTTGGCGGTGGAGGCTA
CGAGATCATGTATTTGTACATGAAATTGAAGTTAGCCACCCGCCAATGGGATGAGCGTGATGATAATTACGATTACACAGATGCTCAATATGCGAC
TATGCAGTACGAGGATGGTGGTGAGGACCTAGGCAAAGCTATCGTACGTGAAACGAGTGGCAAATATCAGCTCGTTAAATCAGAGTTGATTTCGAG
AATTGAACAATGCCGCCGTGATTACGTTCGAGGAAGCTGGGCCGATGAGAGTGAGCTATACGACTATGAGCAGCGGTTGGCTAGAGGCGAGTACAG
AGAAGAATGTGCTCTGCCTCATTACTCCGGAAACGTTCCTTCGTTTTCGGGGGAAGGCCAGTCTCCAGCTCACGGGGCAGAGTATGGAGAGGTAGC
ACCGAGGCCCCTGCGGGTTTCAGGTGCATCTCGTCCATCCCCGGTACAGCAACTCACAAGGGAAGCTTTGTATCACGCCGCTTGCAACACGCTCTC
GAAGAAAGAGTTGCGAGCTGCCCTGAATTACAAAGAAGTTGGACCACGGCCATCAACGAGTACAGCCCAGCCGACACGAGCCAAGAAGCGGAGTGG
AGCTCCTTCTGCATCCAATTAAATGAGGCAGCGGAGCGACGCAAAACGATCGAGGCCCCGAATGAAGCTGAAATAGAGGCGGTCACCACAGTGTTA

GAAGATTTCTATAAAACTGCCATGTGTAAGCCTGTCAGCTCCCTTTGGAATTCTCGTCAAAATTTCGAACAATTGCTAATGACACTGGACAATTCT
TCGGCGCCGGGTTACCCTTATCAACGAGAAGCTCCAACCATAGGTGATTGGTTAAAGATCGTTGATGGAATGCCCAATATAGTTCAAGTCGAGCGC
CTATGGTATGACGTGCAATTAGTCATGCGAGGCGAATACGACCACATATTTAGGGCTTTCGTCAAAGATGAACCACACAAAGCGTCAAAAGTCAAA
GCGGGTAAGTGGAGGTTGATTATAGCATCGTCTTTGCCAGTTCAAATGGTATGGCGAATGCTTTACTTTAGCCAGAACGAGGCTTTAAATGCCAGT
TCTAGCACAATTCCATCAAAACATGGTTTTGTGTTTTGCTATGGTGGGTGGAGGAGATTCCTCGCCATGGCTAAAACTAAAGGAATTACAATCTCG
CGTGATATCTCTGCTTGGGACGTGAACGCTCCTGGTTGGGTCTTCAAGGTGGTGGGTAGGTTACGGGCCAGATGGTCTGGAGTTGATGATGAATGG
AGGCGGGTGCACAACATGTTGTACACCGATGCCTATGAGGATTCCCGAATCCTTTTCTCTAATGGATTGATTGTTAAACAGTTATTCCAAGGGTTC
ATGAAGTCGGGTCTCTTTAATACCATCACAGACAACTCCATGGCCATGGTGGCGATGCACATTCTAGCTTGTTTAAGATCTAACTCAATTATTGGC
CAAATTTTCGCCACAGGCGATGACGTCGTGCAGGAAGTTTGTAGTGATGAATATTTGAGCGTTCTTGAGAAGTTAGGGTGTCGGGTGAAGGAGTTG
ATCAATCATATAGAGTTCATGGGACTTTCTTTTAGGACAGGAAAGCCAGAGCCCCTTTACGTGGCCAAACATATGGTTAATTTCATGTGTAAGGAG
GGTCTAGAAAAGGAGGTTTTAGACAGCTACTGTCGCAATTATGCTGAGTCGGAGTATTTCGATTTCTGGCACGGGTTAGCGAAAAGTTTGGGCATG
CAATTGCCCTCTCGTTCCTATTACCGTTTCTGGATGTCGAGTCCCCTGGCACAGCACGTTGCGGCAGCTAGATTGATGATGTGATCTAGCCCGGAT
CTCTGGAGGTGTAAATAGTTAATATAGGCGTTTCGCCGCGTCTATATTAGGTTGCATCTCCAATTTTGTTGGT

>c2174_g1_i1_len=953
TGACACCGCTGTTCACAATGGTCCTCTAGTTATTAATGGTCTGCTTCGTTCACTGAAGTCGCCCATGGATTTTGAGTTGGGTGTCTATTTAGGCGG
TCCAGGAGCAGGCAAAACCTATGAGATCATAAATCGGTATACCAAAGGTCAGCAGATCACTTCGCCATTGGTTAAGAGTGTTGACGATGCAAGGTC
CGATCTTGTCAAGAAACATGGGTTGCGCCTGCTTGAGTCCCAGAGAGTTGTGAGGACGATCGATTCGTGGCTGTGTATTGCCGGGCAGGGGCGTGC
CTTGCCGTATTGTGGTGAGATTTTGGCAGATGAAGTGTATAACGGACGACCGTCGCGTCAATTTGCAGTGTGGGGAATGATGCGTGCGAAACGCGT
CGTGGGCTACGGTGACGATCGTCAGATCCCACCTGTAGAACCATCTCGAACTGCTCGGTTGTATCCGGAGTTGGCGCCTGCGCATATTGTGAGGAT
GTTTAAGATCTGGCGTTACGGACCTGAAGTCCTTGCAATGATTGCGAAACACTATGATTTCAAATTGCGTTCTATGCGACCTCGCGGTTATACTGA
GTGCATCAATCTAGGGAAGAACGTCAATGATTACAAACCGATGTGTGACGATTATGTTATTATCGGGGCGTATCAGGCACACAAGTCCCTGGCCAA
GAAACGTTATCCTTGGAGTAAGGGACACGTTCACACCATTCATGAGGTACAAGGAAAGACTGTCGAGGAGGTGCATGTGACCGAACTAGAGGTCAA
GAAAAGAACCTCCTACAATTTGTATCAGGATCCGGCGTACATCAACGTGGCGCTGTCGCGTGTCACGAAGCGCCTTGTCGTGAGTGATGTGACGGG
GGCCACGCTTCTTGGCGAGTGGTTCAACTATTCGTTGGACCCTCGCCGCGTTGCCGCTTGTGCGGATGTTGCTAGTGCAGGAACTTCCG

>c11895_g1_i1_len=515
GGCGGACCCTTTCGAGTGGGAGCCCGTGCATTAACAGGTAGAGCACGAATTGTCCTACGACGTAGTGCGTCACGTGGGACGGAAAGAGCGCACGCC
CCTTGAGCTTAGCAATCTCGTTTTTAATAAAATATCTGGTGAAGCTGTTGGGTACATATTCAGCTATTACCCTCTTAACCAACTCAGGGAACTCGG
CGAACTCAAAGGTAGCGGCTTTGTTCAGGCGGACCCGCCGCAGTACGTATGTCCCCATGGCTGCGAGGTCATCCGCAAGCTCAGCGACCTGTATCT
CACGCTCCCCGACCGCTTGTAGGTACACATCTGCCTTTGGCCCACCTGTCACGGACCCTGGGCGGACCCACTTCTTCCTGTAATCGAGGAAGGTGT
CGAAGTCCTCTGTTATAGCACTTGCCGATCTCATAGCTGCACCCCCGTCGTTGATCGCTGTAAATAAGAGTTGGCGCACTTCTTCCGCAAACACCA
TCTCGAAACGACCTGCCGAGGCTGTTCCATCGGCG

>c17275_g1_i1_len=561
CGCCTCACCACCGGCCCGGAAGACGTAATGAACGACCACCGCATATGGGCTGCAGCGCGCGAATTCACCGTGGGCCTTATGCTGGACTACGATAAC
TTCAATGAGAAGCATGAGTTTGCAGACATGCAGATGATCATCAACGAGCTCAAGGGATTGTACCGTGTGGCGGGGGTGATGAACAGCGATTTGTCT
GCAATGATCGACTGGGTCGTCGAAGCGTACGATCGCACTGTCCTGGAGTACGAGGACCAGCTCTTCGTCTTCGGACATGGCATGTTATCTGGCCAG
GCGCCAACATCGGCTATCAATAACATCATCAACGGCGCTAATAAACGTGTGATCAGGCGCCAGGTAGCTGAGCTGACAGGCCTATCAGTCATGACC
AACCGCACGTCGGGTGGTGATGACGTAGCCGCGGAAACCACTGACTTGTACGAGGCGGCAGTTATTGTGGCCGTCGGGCAAAAAATGCGCTTCGCT
TTTAGCACCCACAAGCAACTAGTTAGCTCGGGGTTTTATGAGTTCTTCCGGCTCATGACGGACGCTGACGGTGTGTACGGG

>c18610_g1_i1_len=1073
GGCTTCTTCTTTTTGCAGCACAGTATAAGTGTATACCTACTTGCTCAACACGCAACGCTTGCTCCGCCGCTCCGCGTTAAGTTATCGTAGCTAGCA
GACGACATTTCAGCAAACTTCAACATGGCTCAAGCAGCATTTCAAAGCCAACTCAGCATCCATGCTGGGCTCGGACTCCCCGACGACTTTAAGCGC
AATGGACCGCTTCCGGACGCGCTATTCGCCGCTTTGGCCGACGAAGCGGGGAACGTCGCCGGTCAGCGGCCCGTAAAAACACAGCCAACAGGGCTA
CCGTTGGACCAGTCTCGGGCGTCCGTCAAGCCGGTTGCACTGAAGGAGATGGCCGGCGGTCAGCTGTACTCGACTTTCCTAATGTTGGGTGACAGT
GATTTCGATGTTGGACCAATGGATGAAGCGGACGCAGATCACTCCATCCACGTGGCGTACGCGTTAGAGCCGCCGGGCAGCTCGCTGCTCCGCTCA
TCGACCATAGAGACACGGGCTCGCTACCCACGCTTTGTATCTCATCGTCACGCATGCAGGGTCAGCTACACCATGAAACGGGACTTCTACGCGCGT
AGTTCTGCAAGCGCAGCCGCGGACGGCCTGGCGTCTCACGCGGCCTCAGGCAACAACTCGCCCGTCACATTGGGCGTTAGCAGAGAGATGGTGCAC
GCAAGAGAAGTCCGGTATGGAGGGGAGATGGAGTCGGTGTTTCTACGTATGTGGCTAGTGCACTTTAGCGTCCTGGCACAGAAAGGCGTAACTGTT
CGCGTAGACAGGCCATCCTTAAGGGGTAGCTTAGCCGGTGTTGGAGTGGCTCGCGAGAGCGATCAAGCGCGTGTGCTGTCTGGGGTGCGAGTCAAC
GCTCGTGGTTTGACGCCTCAAGCGCTCGGCCTGCTAGTGGTGGGTTGTGGTAACGTGAACGGCCTTAACGGGCTGCACCAGAGAGCGGACCGCTAC
CGTTTCCGCTCCACACCCCTCACTCTATATGGCACTACGGACACCAGCGTGAGCAACGTTGAACTGCGCGACCCTCACAAGACCGGTGCCGCCATA
GTGGCACTTGCCAGCCG

>c54876_g1_i1_len=513
CGGGGAGTTCTTCTCATACCAAGACCTCGCAGATAAAGTTTACACCACGCTGCAGGGAGTTGGGGTACTTCGGTACGACCAACGGTCCTATGGAGC
GTGGCGTCAACTGGCTGCCGCTAGCAGCAAGCGTGAAGAGTTGAACTTCGCGTATGACGTGTGTCGCGATCGGTCGTACGTCCCTTGGGTGCGCGC
TGTGGCTTCGAGAGTGATTGAACGTTACATGGGTGCTGCACTGCCGGAGGAGGCGGCTGCTTGCAATAGCTACGCCATGTGGGCACGCATTATCCT
CGCTGCTGAGCAGCATCGCCTGCCTGAATGCCCGCTCGGCGACACGGAGCGCAGCTGGAGCGACCAGTTTCCATACGGACCCGGCAACTCGAGGTT
TGCCTTGGTACGAATTGCGGATTGGTTACGTGACGGCGATAGGTCACCCGACCAATATTTGTGGTTCCGCCAGCTGCTGAACCGTAAGGACGCATC
TTACGAGCGGGCGGCGTGCACTGCTATGATGGG

>c10259_g1_i1_len=1503
CATCTAATACGTCAGCTTGCGGGTCCACAACCCACTTCCTTTGTGACCTGCTAGCCTCGGTGTCTGGTGCGGCGCTCGAGCCTTAGTGGCTGGCCT
GCGGTTCTGGCAACGCCACTCCAATGGCCGCCCCGATTGTGCACGACTACCGGTTTGGCATGCGATGCGCCGGAACCCACACGATAAGCTCTTGTC
CCGTCCGCGGTGTCATGCACCGCCACCAGGCTGTAGTCACCTAAACTTTGTAGCCCGGCCGCCAGCTGGTCCGCTGTAAAATTGTTGTCCTCTAGC
TTTACACCGGCGCTCTCTGCCAAAGCTATGCTTTCGGGGCTAAGGGCTGATTTTATCGCGCTAGTGATGTGGTCCAGTTCTAAACTTACGCCAGCC
TGGGACTTTAGGGACGCAGCCAGTGCATAAGCGCCGCAGAGCAGCCCGCTCCCACTGGTCGGGACTACATTGTAATGCTGCTCCTCTAGCTCGCGG
ATGATGTCGTCCTCCGAGCCGGCAGCCACTAACACTGGCCCTTCACCACCAGCCCCGGTGTAGGCGTTTGTTGTCTCCAGCCCTCGGACCCGGCGC
ATGATCGACCCGCGCGGTAGGGCGGTCTCCATAAGTTCATCGACGGGCACCCTGTGTACGTTAGGCTCCGTATCTTCCCTGGTCAAGCTCTCCGAC
AGGTAATGTGGCCTAACAGCCGCGGAGTCTAGCGTCGTCTTACCCTCAGCCTGTGGGGCCCACTCCACTGCTGTGTCGTTCAACTGGACACTCAGC
CCGCTGCCGACGCCCAGCAAAGTGAACTGCACACTACCACCACCTAATTGCACCACCTGGTTGCTAAACTGAGTCACCGCAGTCGCCCGTCTCACG
CGGCCACCTGTGACCCAACGAGGTCCTCACCTGCCCCCTCAAGAAGGGACTGGACCAGCAGAAGGGTGGCTACGTCAGCAGCCCGCTGTTTTCGA
ACAGCCGGATCGTCGGTGGATGTGAGCGAGGAAGCGTAAGGAACTGCAATCGTTTTGGTTGCTTCGTCAGCCGTTGACCCAGCAACAAGGACCCCG
TAAGCCAGGCTGTGCACTATGCCTGGTTCTGACCACAGTGATCCGTAGTTGTTCAACACAAACTCTTCGCTATCTATGAGCGTACTCATGTCGGCG

AAGAACTCCCCGTGCTGCCGCCCGACCTCGCGGAGGAGGGTGCGCTGATTTGGAAGGAGCATCTGTCCTGCCGCGTCGACATCGGTTGCGTGCATG
GAAGACATGGATGCTCGCAGCATGTGTCCACCCACTTGTCGCCACGTCCTACCCATGAACAGCGACAGGGATAAGAGGCGAGCATCGCCAAAGTCT
GAGTTGCTCGTGAGGGGCCCGCCTGCGAACCCAAGAGACACCACAGCGTCATCATGGAGGTCAGGTTCGGCACAAGCCAGGGTCAACCCGCGCCCA
GCATCTGTGACACCAAACATGAGCAGTGCAGTCTTCAGCGCGTTCGCGCACTGCGATTGTGAG

>c18623_g1_i1_len=378
GTGTGGCGTACGGACCACTTTTCGCATGCACCTAATAGCTGGCATCCGGCTGCCATTAGGATACCCGTGGTCTGCGCGTAGGAGTCAGCATGATCG
CTCATGCCCTGGCCGACCATGGAGTTGGCCATGTCTACCCCGCCGTCGAATAACTGCGTCAGCCCGCCAATCGGGCACCTGTTGAACATTGCCAAC
AAGTACCCATAGTTCCTATAAGCGTCGACGCAAGCGGCGTAGCCTGACTGGACATCGTAGCTGAACGGGCTAAACCACTCCATGCCCACCCCCCCC
GTAAACAAGAGGGAAGTTATAGACGCCATCAGCGGCCGACGCGCTTCAGGGTCCGTGAGCCTCTCAATGTACTGTTCAACGACAAGTGAG

>c18803_g1_i1_len=293
TAACCCGTGGGCATGCACCACCGGCTGCAAGGACCCCAGGCGGGTGGTGCCTGCAACTATGTGGTCTTTCATTATGAAACGGGCCAATCCAGACAT
CCAGTAGTCAAGCGCAAGTTCTGACGGGTACTCCGTGAAGCCGTCATCCCGTTCCGGCGACAGGTCTACCCACTCGGATTCACTCAAATACTCCAA
CCAACGTGGCCCAGTGGTGGGCCCGTACTCCAGGATCAGCTTGACCTCCTTCCCATCTCCAGCCACTGCCTTGAGTTCGAAGGTGTTGCTGCCGTG
GCTCG

>c28520_g1_i1_len=232
TGTAGGACATGAGTGCTCCCCCAATTGCCGCCTTATATGCCAGCCCGAGGATTGCCGAGCTGTGAACTACTGTGGCGATATCATCGATCACCACGT
GAGCAACGTCCTGATCCGGCAGCAACACGTAGGTACAGGCGTCCAGCCGTGGCTGCCCGGTGCGGTAGGGGCTTATGGCATGGTCCCCCCAGATGT
GGGGCGCTAGAGCAGCCTGTATGAGTACCGCGGCGGCTTG

>c31892_g1_i1_len=286
GGACCCTCGGCTCACATACCTCTACGTACACACCGACACCTCTGCTGACTATCGTGCGTTCCTGACCATGGGCGTACGCGGTGTGCAGCATTACTC
TGGAGCGATCGACAGCGCTTATTCGGCGTGCGTGTCTGAGGAGGAGGCTAGGGCTGAAGACCAGCTGGTGTTTGTGCGTGTGGGCGGTGACGCGCC
CTCACCCGCGTCCCCGGGGGCTGACTCCTACACGCGCGTGCTCGGGAACCCGGGTGCATGCCTCGCGTACTACTTCGCTTATGCTCAATCCATG

>c35229_g1_i1_len=493
GTTGACGCCGCGCAGCACCTCCTCGTCACTGGGAGGTGGACGCCGTGCAACGCTGTCTACGTGCCAACGGCACCTAACGTAAGCACTATGCGTACC
GCCAGGGAGTGGCCAGAGATCGCATGCCACGCTAACGTATTCTCCAGCCACCTCGGTGCGCCACCGGTAGAACAGCTGACGGCCAAGACCATGGTG
ATCCAATACGATGTACGTTCAGGAGTCTCGCACGGAATCATTGCCGCGTGTGACGTCTCGCCTAGTCTGCTAGTGATAGGTGGGTCCCCCAACGGT
AAGGGAGTCCATTACTCGATCTGCCGAGTACTGGTGGACGGATTACGCGGACATTGCGCTACTGTAGCCACTCAACAACCAGAACAGCCCCTCCTG
TACTATTCAGCAGTGCGGCGGCAACCGTGCCGCAAGGACGGGTTACTGTCTCCTGCTCTAGTGAACCTCATGACCGGGTACGAAGACGGCAGGGTG
GGGATTGACTTTG

>c45206_g1_i1_len=581
CAATAACTGAGGAAGTCGCTCGCAACGGTATGATGACTGGCGCTGTTAACGCTGACTTAGAATTCTCATCAGCGACCGGGGCCTCTGCGGACGATG
CGACGTGCCGCTTAGCCACGCTACCTTACATGTACACTGGGGCCAATAGTACGCGAGTAGAATACACGTTTGCTGGTAAATGTTTTGTTGTGCGAC
CAGCCGTTAATGAAACAGTTGTGAATGCCATGTACTCGGCGCCCACCGCCGATAAGTGGGCACAAGCAACAGGCATCAAACTGCCGCGAGTCACCG
CCCAGTCTCAATCGCCTGGCGCCGTTCCAGGGATGCCCACGTCTGGCCTGGCCAACCCGATGCCCCAACAGGGGTTCGACAGCTTGCGGAAACACG
TTGTAAGTTGCGCACGCGGGGATGACTACGGAGATCGTTTGTTCCAGATGGCGCGGGTAGTCCTCCACGCACAAGTGCTGCATACTCTTCGGCACA
AGCCAGTTGTCACGCCAGCTATGCAGTCTACCAACGCCGCATTTTCTATAGCAGCGGCGGACACGCCCAACCTACTACCCTCGCAGCTGATGGACC
CTCGG
>c55497_g1_i1_len=239
ACCGGACAACGTGGGCAAAGGAAGTTGAAAACACTGTCGTCCAAGTTCGACAGATACTCGACAAGGAAACAGGAAGACAGAACAGATACTTCGACG
ATATCAGACAGCGCGTGTCTAGCCAAAGCATACAGCCAGATGTGGGCTTTGTACTTCTTCCACAGAAGAGACTTGAGGTTAGGATTCGCGGCCATC
GCTTCTAGTGTTCCTGTGGAATGGTTCCCTGAAGGGCGAACGTCGTG

>c40408_g1_i1_len=317
ATTAAATAACTTTAATTTTTATATTGGACACACGACTGATATTGTTGAAAGATTATATAATCATTATTGTTATTCAGAAAAATCATCAAAATGGGT
CATAAAGAATAAGCCTATTATTACAATTTATGAAATAATAAAGAACTCTAAAAAAGAAGATGAAAAGTATAAAACTCTTGAAATTATGAAAAAGTT
TGGATGGAAGAATGTTAGAGGATATGCATGGTGTCAAAATAAAATTAAAAAAGAACCATTACCTCTTCAATATTTTAAATATGATCGTGCAGATTT
AGAATATATGAATTCTAATGAAATTAGAG

>c9510_g1_i1_len=536
GCCCGTCCTTACCGTACTCCATTTTGCCGCGCATTACAATATCGGCACATCCTATCCCCCCGTCGTGCCGAGATAGGTGCGTGTAGCCTTCCAAGT
CTTTGCACAGCCTGTCGTTCAAGTGCCGCCCAAGCCAAGCTTTGCAAAACCTCCCCAGTACGGCCTGTAGGCTCTGGACATCGACACCTTTAGTCT
CCCTCTTTAACACCCAATTAGACAGTAGGGCTATCTGTTGCTTCCGGTTCATTTCACCGAGCCACGTGCCTGAGTTGACAATTTCTAGCACCAAGC
CGACCCCTGACCTGCTCCAATCAACAAGCTCGGCAATCGTCGAGAGCACCGAGTCCTCACACCCAGAGGAATTAATGTATTTCGGAGCTCCTAACT
TCATGATCTCGCGTACGTACTCTGACTCTGTGTTCCCCAGCACCACTGGTGACACTCCCTCTGGCAGCCGCTGCAATGCCACCTTTTGCCCTAACT
GTGCACCGCACCACCCCACGACTATCAGGCGCTGTAAACAGTCAGCAACTTTGTTG

>c16117_g1_i1_len=424
GTGTGACACAGTTCACCTCAGTCGGGCATATGGTGTATCCATGGTTTCGTGTCTGCCAGGCAGAATGGTCTAACAAGATGAAGACGCTCCATTCAC
TGATACGTTGCAGCCGATATTTTATGAATACAAAGATCAGCGAGCGCGACCGGCAGGCACTAATGTACATGGACCTGTTGGTTGGGCGAGGTGAAT
ACACCATTGACGAAATGGCTGAAGTGGAATTGCGTACCGATGACACCTACGACACGAGACACCTGTCGTATGACCCGCATAGGCAGGTGTTTACAA
ATCAACAATACAAGAAGGATTTTATCACATCGGTAGAGGAGGCGTATATTCGACTTAAGATTGAGCCTAAACCAGTCAATGTTGACAGTTTCAAGG
ACTTTTACACGCGGAGGGCATCCTGGCTAACCAAGGGGGG

>c16636_g1_i1_len=213
CACAGAAGGAACAGCAGCGCCATACCTCTACACCAAGTGTTCAGGTCGCCACCGAGAGGTAGTGAAACGCACTCGTCTGAGTCGACCAGGTGGAGC
CATCACGACGGGGTGCAGCTAACCAAACGCGAGGAGCCACCGGCATACGAGTCGGGAAGGAGCTCGTCACCAGTGACTTCCAGCATCGAGGACTCA
GTGAGCCAGCATGAAGGAGAG

>c18833_g1_i1_len=466
GTTGCGGCGAAACGTGACGAAGGCCTAAGGCTTAAGCGGCTGGCTAGGACGAAGCACCGCGTGGAGATGGTGAAAGAGTGTGGGTACAGCCCCGAC
CTGAACCTCAAGGGACATGGGCAAAAGAAAAGAATAACTAGCCGTAGCTCATACAGGAGGAACAGTAGTGCCATACCACTCCACCAGGTCTTCCGA

TCGCCGCCACGAGGGAGTGAGACTCACTCGTCTGAATCCACGAGGTGGAGCCACCATGACGGTATTCAGCTCACTAAACGTGATGAGCCACCGGCA
TACGAATCTGGGAGGAGCTCGTCCCCTGTAACATCAAGTATAGAAGATTCCGTCAGCCAACATGAAGGTGAGCAAGGGCTTTTTGACGACGAGCCT
GAGCTGCCAAGGCAGCAGACGGTTTTAGCTACGGCAGCAAGACGGCGGTTAGGACGGGCAACACTTGATAAGATAATGGAGG

>c19064_g1_i1_len=553
TAATTCCTAGGGGCCTGAGGAGTAATCGAAACGCCTGGTGCGTAGCCCCCGGGTATTACAGTGACTATAGTTATATTAACAGAATAAACAACAACA
ACACACAATTAGAACACAATACAATCCAAGCAAAGGACAACAACCAATACAACATCAACAATGGCAGGAATTTATTATGGCGGTAGTGGGGGTAGT
AGTCTGGGGAACACGGACATGCGCCGTGCACAGATGAATGAAAAGAAAACCGTCTTTGCTCAACTGCGGAAGGAGGCCCAGGCCTTGGCTGTTGCA
AAGCAGGCAAGATCCAAGCTTACCACCTGGAATCCTGGCACTCGAGAAGGGGCAAAGGGGTACAGGGAGAAAGTAGTCAAGTTTGGCCGACAAGTG
GGTGGCCTTCTGGATTATTTTGATAACATGCATTCGCCCGCGCTTGACATAATAGCAGGCGACAGGTTCTTGCTGAAGTATCAGATCTATGGTGAC
ATTGACCGCGAGCCGGCATTTGGTCAGAATACGATGACGGCTGAAGTACCGGTGAACTGGGATAAATGCGAAG

>c19508_g1_i1_len=293
CGGCCCAGTGCCAGCTCTGGGGCATGGGTATGGATCTGATAGACAAAGGTAGCCAATCAGTGACGCGCTATGGAACAGCATCCGATGACTATCGGG
TTACGGTCGTGAGGGATGGGGCCCATTTCAAGGCCGCACAAATCGGGAAGGGGCCGAACAAACTGAAAGTAGCAAGGCTCGAGGAGCAGACTGAGG
CACCAGAGGACTTCGTAAAACAGGTTAAGGAGTACGGTTCACTATTTGGTGGTTCTCCAATTCTGCAGTAAGTGATGGAACGTGAGTTTGAAGGGT
TGGAG

>c27061_g1_i1_len=719
GGATCGATTGCGATAGTTTGATCAGCCCAGCTATGAAGAAAGAGCTGACTCCGGCAGCAATGGCATGCCTGGCAGAAGGAGGCGACTACGAAGAGG
CATTCTGCCAGCTCAGTGCTGTTATGGCAAAGAGCCTGGAAATACTATCTCCGTCAAAGCCCACGTTAGTATTGACAAATAACTTATCATTGCTGG
AGCGATGTGGGGTACCATGTTTAGCGATCATGTCTCTCGAAGATACGGCCTTTGAATACAACCTGAGGTTGAGAGATGATGTCGAAAAGTACACTG
CTAGGATAAGTAAACGTTGCCTCGAATCATATGAAACGGGGTCACGGCCGCTTATAATGGCACAGGATAACCATACCATGAGGCTAATGCTGTACC
AGATAGCTGAGGGGATGGGCATTGAACTCGGTGCCCCACGTGAACTCGACCCAAATTGCCCGCTGCCACAAGGGGTTGGGTGGTCAAGGTCTTATA
GCCTTAGCCGCCTGGCCGAAATGCATGACATAGGGCAGATAAGTACCGCTATATTCAACTACCAGATCAACCTCCAGGGGTTGAAGAGCTACCACG
GATGCGGGTTTACAATGAATGATTGGGCTTCCGTCGCTAGCCAGCTAGTAGACACCACACCTCTGCATGACCCTGCCATACCGGGGTTGGGCAAGT
GGCCAATCACTTTAGAGGCGATTGGTAAACATTTCGAATTGTCTGAG

>c27836_g1_i1_len=245
CTGGGGGCCGCCGCCTAACCATAAGCATAAGCTTGAGGCCAGTCTGGAACACATGAAGAGCGTGAGTCGGGGGAATGCGATAGTGCCAACACGGGA
GCCCAAGCACGTCCGGGTGAATTCGCAGAGTATAGCCACGGTACCAAGATATGTCCGAGATAATGAAGAGACAGAGGTCTACCTGCACTACACAAA
ACCTGAGATAGCAGAGGGTGACGAAATACAGTACAGCGAAATCGATGTTCCGG

>c29121_g1_i1_len=631
CGCACCCAGAAGAGTTAATATACTTCGGGGCTCCCAACTTCATGATTTCGCGGACATACTCCGATTCAGTGTTCCCTAGGACGACAGGTGACACAC
CCTCCGGGAGCCTCTGCAGTGCAACCTTTTGCCCTAGCTGAGCCCCACACCATCCGACAACAATCAGCCGCTGCAGACAATCGGCCACTTTGTTCC
CCCAGGATGCACCTGACAGTCCCTGGCAGCTGCTTGTTGCCACCATAGCGACCACCATCTGGCTCAGCTCATCTCCTGCTTCACATTCGTCGTCTA
ATACCTGACCTATGAAGTCAAACCACTCATCGCGTATCCGACCGACCCGGAACCAGTTCGTAGCAACCTGCTTCATCGCCTCGCGTATGATACGTG
GCGCACCTTGCTCACCTTCCCAATTCTTGGCTAGTGTAACTCCCAGGTTGTAGCAGATAACATGTTTCCTCTTCAGCATGGCCTCCCCACCCAGTG
CCGAAGCGCAGTCTGTCAATGCTTCAAGACTGGATTCTCTCAGGTAGCTCCAGGCCTGCATCCGGAATTCTTCGCCAGGATCGGAAGCTCAATCAG
CAGGCACTTTAGCATCTTGGAGTACCCTGACATCAGCGATCTGCTCGGCCCCTTG

>c29554_g1_i1_len=383
TCAAAGACTGACTGCCGCGAGGTCAATGCGATGATACCGCTGAGTTACTCTATGAACACGGCATTTGGGCAAGTGAGGGAGCGCCAGGTCGTGACC
AGGATGCCAGGATTCTTTTCTACTAGCTTAGCATTAGTGTCAGACCTGATGTATGGTATGGTCTTCAAGGGTGTTGCGTCATGTGTGTCAGAAGCA
CTTGGTGCTATGGGCACAGTCGTGTCGTCATCTACACCTAAAACAAACCCTACCATTAATGGTCTAATGCGAGACTACGGGCTGCAACACACGGAT
GCCAGTGACAATTTTATGCTGCGCAATTTTGAGATGTTCACTAAGAAACCAACACAGTGGGATATCGGCCAGCATATGAAAGAATATGCGCTGAG

>c31457_g1_i1_len=393
CGACCGCTTCACTTGTCTGGTCTGCCCGATGCTAACCATACTACACAGCTTGAAACCGTCTCTGAACCTGTTCATCGTGAACTTATCTTGCAGGTT
CAGGTGTCGGATTATTTCCTTCTCCCCATCGGAAATAAGTTTCTTTTCTACTTCAGTTGCGTCTATCTCCTCAACAAGCCTTTCACTATTCTGCTC
CAGAAACTGAGTGAAGTAAGTCTCCCTATGTTGGTATGCCTGTGGCAATCCCTTCATGTAGTCTATAACTCTGGCTTCGGCTGCCATATTAATCGC
ACTATCTTGTTGTTGTTCAGTTGTGTTTGTTTGTTGTTGTGTTGTGATTGTAATGTAATGTTGTTGTTGTAGCAATGTCAATAATACCCGGGGGCT
ACGCACCAG

>c32979_g1_i1_len=449
CACAAACACTAAATTTGACACTAGCTGTACTATGGGTAAACTACAGGATTCGCTAATTCGTAGCGTAACCGGGCTATCGCAGAGAGAGCTAGAGCG
GGTGAGGGCTGGTGGTAGGTCCGAAAAGGCACGCCATGGTAGCCACAGGAAGAGGCTGGAAGACGTGGAAAGTGGTCGGCGCGATACCAAGCGTGA
GGTCAAGAAGCATGAGCACAAGATTAAGAAGAGGTCTGAGCAGATGAGTGAAAAGGTGATGTTTGATCGTCTTTCCCTACCACGGCAACCAAACTC
GCTGACAAAGTTGCCGGTTTACGGTATCTTGATGCCTATGGCCAATGGCAAAACCACACTGGCACAGAGTGAAGGGTGGATCGACTGTGACAGCTT
GATTAGTCCCGCAATGAAGAAAGAGCTGACGCCGGCTGCTATGGCGTGTCTAGCAGAAGGGGGGG

>c33782_g1_i1_len=204
GCGGCCATTTAGCCAGGAAGATGGGCGGTGGTTAGTAGCAGCAGCAAACGCGGAAGGCAGTTACTTTAATGTAAGGGAATTAAAGAAGCTGTGCAA
GGAGTTCAATGTGCCCAAGGAGCTGAGAGAAGCACTGAGAGAGAAATACGGCCTTTTCGATGGCAAGAAGAGCACAATGTAAGGCAGCAGGGGAAC
TATGAAGATCAG

>c34127_g1_i1_len=361
CCTCGCTTCCTTGAAGTAATGGACGCCATGTTGTTCAAGGCTAATAAGTGGAAGCAGATGTTCGGGACGCGCTCTGAGTTTTTCAGAAACACAGTA
TGCGAAGGTCGGGTGCATGCCAGTCCAACGCGGGCACTGGCCAGTTTCATTGCTGGTGATTGGGAAGGTGCTGGCCGGGCAACAGTGAAGGAGCGA
GTGGTAAGCCTACTCGACCAGATAGCAAAGCTGAGGCGTCGGGGGTGCAGTGAGGAACTTTGCCAAGGGCTGACAGTATCGACTATCAGCCACTGG
TGCCGCATACGTGATGGTGAAGATTGGCTGTCGCTGCCTGCTACGATCATTCACGGCCATCCGGATGATGGGG

>c35317_g1_i1_len=276
CTCCGATATTTCTAGCAACAATAGGAACCCTTTCACTAACCGCGTCAGCTTAGATTGCCCCGTCTGCATTTGTTCCAACAATGAGACTAGCGATTC
CATCCGATTGTCGGGAAACGCACGGACCGCATCCTGACTCCACCCCGTGCTTTTGGCCACATCGTTGCGGTTTGGTATCCCTTCTCTGGCACTACC
TGTCAGCTTGGCCCGTGACATTAGTTTGTCGAGTGTTCCCGCGTACAGCCGGACATCAACCTCGCACTTATCCCAGTTGACCGG

>c37624_g1_i1_len=614
CAATATTATTGACTGTTCCATCGCTCACTGCTGTTAACACAGCATTTGGCCTAACCCGTGGTTGGTACGGTGACGGCGACACGCTCAAGATGTCGA
AGAAAGAACGGCGGGACATGACAGACGCCCTATGCGCTGTCGGGTGGATGTGTGGGCTGAGGAAATGTAGGCCGCAGGTATTTCGGAATAGGGCAG
GTAAGAAGCAGGTGATGGTGAGCGCTAGTGAACGGAAACTGCGAGCAGAAGCGGGGGAGGACTGCCGAGTACGAGATGTAGAGTTCTGGCTGGAGG
ACACCCCAGGTGGCAGAGTAGATGAAAATGAAGAGGCCGGCAACAATTTGTACAGGACAGAGTTCAGTGGGACAAAGTGCGCCATGGTGTTCAATT
ATGAGATGGGGATGTGGATTGAGGCTAGGCAGACAGACTATGATCGAATGAAGCGAGAGACTATGAGTGGGGACCTGACTAAAAAGGAACGGGCCA
CCATGAGCAAGGTCAGCGCAATGCCTGTTAACTGGGGCCCGCCACCAAACCACAAGCATAAGCTGGAGGCTAGTTTAGAACATATGAAGAGTGTGA
GCCGGGGGAATGCAATAGTGCCAACACGGGAGCCCAAG

>c39328_g1_i1_len=266
CGAAGCGTTCACTCTAGGACTGCTACTACATTGGAAATTGTACGGCCTCAGGAATGACACTACCGGTAGGCTGCGCCTACTATACCATGTTAGGCG
GGCAAAATGGGATACAGTGATGCGGAAGGTGAGGCAAGGAGTGTTAGCTAGTAACACCTTCATGGGCGAACCATTGACATTACTGGAGCGGGACAT
ATTATTGTCGTTGCACATGCTTTCGAGCACAACATCAGCATCACTCATTAGCAAATGGCGGGAGGAGAAGATGG

>c43319_g1_i1_len=505
GTTTGATTTCGGCGTCACGATGTTCGAGTGGTCCCAGGCATTGGGTCAGCTGCCCGCCTGCTATAACGAGCCGCGCCAATTTTCGACACTTGCTGA
CTTGATGCAGGTTTTCCCACCACAGTCCGCTAAGGAGCTAACCAGGGCAAACGTCACACTGCGGCAGCTCTGCCACACATTTGACATATTATCCAT
GCCTGATGCACGAGAGATAGCTGAGTACCATGTTGGGGAGAGTCAGACTTTCGTAACGTGTTTGCTGGCCGAATGGAAGGGTGTCACCCAGTTCAC
CTCAGTGGGGCACATGGTATACCCGTGGTTTCGCGTGTGCCAGGCAGAGTGGTCAAACAAAATGAAAACACTACATTCGCTAATACGCTGCAGTCG
GTACTTCATGAACACAAAGATTACGGAACGAGACCGTCAGGCACTTATGTACATGGACCTGTTGGTAGGCCGTGGCGAGTACACTATCGACGAGAT
GGCTGAAGTCGAGCTTCGAACTGAC

>c45192_g1_i1_len=938
ATGTTATTTCAGCATCAACCCACAAACTGCCACAACTTGATTACATAGTACTCTTCTTCCCGTCAAACAGTCCGTACTTTTCCCTGAGACTGTCTC
TTAACTCCTTCGGTACGTTAAATTCCTTACACAACTTCTTCAATTCTCTCACATTAAAATGATTCCCTTCAGCGTTGGCTGCTGCAACCAGCCAGC
GACCATCCTCCTGACTGAAAGGCCTCTCCCCCAGACTGGATGGCGCTGATCGCCACTCCAGATCATGTGTGGGTAGTCTCTCCAGGTCACTTGCCT
GGAATTGCGCCGGCATTAGGTTCATAGTCATCCCATCTACCATACCTGCTAGCTTACGTATTTGTTTCAGTGAACAGTTTCCCCTAGATGCCGCTT
CAGCCAAAAACCTACCTAGCTCCTCGCCGCCGAACACTTCATCTTCGGGGTCCAACCCCAGATCGGTAAGCCACAGTCTCCACACCTCACTACCTT
GGACAAACTTCAGGCCTTCGGCAGGAGCCTCTTCTATACTCCCTCTCCAGTTGCTGCGTTCGCGCATCATAGACCCTAATAGACTCTCGAACTTGC
CCGGGTTGCCATGCTTAATTGCATTATTCACTGTAGTTGTATACCTGGAGTTACCACTTAGTGTTGAGTTGACTCCATTTAGCCTGCCGTCCTCAC
CAGCTGTTACAAGCCTATGGACTAACGATGGCATATCACTCGAATCCTTAGCGGACCAGCACCCTATCGTCTTGTTGATTAAGTCCAGCCACTCTT
TTCGCTCACGCCCACCGAGCCCGCCGGCCTTAAGCAGCCTATCTACCTGCTCCTCTGCAGTGAATTGGCTGTCCTTAGGGCTTAACATCCGCTCTA
TCTGCTTATTCTTCTCGGCTGTGGCAATGCCTTTGGCCTCCTGAGCTTCAGAATGTGCCTCCATTATCTTATCA

>c45358_g1_i1_len=208
CACGGGAGCCCAAGCATGTGCGGGTGAACTCACAAAGTATTGCTACAGTACCTCGGTATGTGCGGGATAATGAGGAGAGAGAGAAGTGTACCTGCACT
ACACTAAGCCCGAGATAGCAGAAGGTGATGAAATACAGTACAGTGAGATTGATGTCCCTGGTGACGGGTTGTGCGGAATACATGCAATGGTAAAGG
ATCTAACGGTGCACGG

>c45834_g1_i1_len=494
GTAGCCCCCGGGTATTACAGTGACAACATTTATATTAACACACAACAACAACAAACAACACAACAACAACACAACACACCACAACACAACAAGCTA
CAAGAGCAATCATGGCTGCGGTATACTATGGCGGAAGCGGCGGTAGTAGTATGGGGAATACAGACATGCGCCGCGCGCAGATGAACGAAAAGAAGA
CTGTCTTTGCCCAGTTAAGGAAGGAGGCTCAGGCGTTAGCGGTGGCAAAACAAGCCAGGTCCAAGCTTACTACTTGGAACCCGGGAACCCGTGAGG
GGGCGAAGGGTTACAGGGAGAAGGTGGTGAAATTTGGCCGTCAGGTCGGGGGTCTGCTAGATTATTTTGATAACATGCACTCACCTGCTCTTGATA
TAATAGCGGGAGACAGGTTCCTGCTGAAGTACCAAATCTATGGTGACATTGATCGGGAGCCAGCATTCGGCCAGAATACTATGACGGCTGAGGTGC
CGGTCAACTGGGAT

>c48065_g1_i1_len=289
GTGACTCAAATGGCAAGTAAGCTGACACATATCGGATTGTTCGACGCTACACCGTCGATGCGATTATTTTCTGAAATGGACGACCGCGACTATGCT
GATATGCTGAACTTGTCCATATTCGAAGGGCTCTGGCTGGTTTCGGATGCTAGCGCATCAGTAGACAATGGTCCTATTAGTTTCTTGCTCAATGGG
GACAAGCTGATGTCTGGTGATAAGGCAGGCTACGATGTGCTCGTGGAAGAATTAACGTTAGCAAATATCACAATCGAGCACCATAAAATGCCTACG
G

>c48709_g1_i1_len=393
GTCCAAGGACAGCAGTATGTACAAAGCCGAAGGCATGAGCTTTGATCTACGTGCTAACCTGCCAGCTGATGCCCGACTAGCTACAGTTATCAACCA
TGATATAGTTATAGATGCAGACCTGTTTACACCAGAACAGATATCACTGCTATGTCTTGCGGGGCAGCAGTATCCCAGTGTATGGTACGGTGGGGA
CAACAACATATACAACAGTGTGAACATGATGGCGGATGATTTGGTAGTAGTCAGTGACGGTCGTATCCACACAGATACTTCCTTCACCTGGGGCTC
CCCCGATCGACTATACCACATGATTTGGACTACCGCCCAACGGCTTAATGCCGTAAGTTGCCTAATGTACGCATTGGAGACTATGCGCGGTAAGTG
CAAGATGAT

>c49342_g1_i1_len=1296
ATTCCTTTGGGCCTGAGGAGCGTGCGAAACGGCTGGTCCGTAGCCCATAGGGTATTATTGACAACGATAACATATTACAACAACAACGATTCAAAA
CACACACAACAACAATACAAACAGCAACTGTAGTTGACAATGACTATCACAGGTCGGAACAGCTGGCAGTCGGGAAAAGTTACAAATGCAGAACGG
GCCAGCCGGATGGCCACTGAGCGGGACAAGGCATCAGCTAATTACGGTTCGAAAGTGGTTGAGAAGGCTAGGTACCAGAAGTCGCTCGGTAACTTG
AACCGCGGCCGGTATCTTGGGATGAACAAGTGGACAGCTGAGCAGTTGAAACATTCTATGGCCAGGTACGAGGACCTAGCTGATCGGAGACACAAT
CTATTCGCCGTGATCATGCCTGCTGGTTGCGGGAAGACAGCCTTGGCTGAAAGGTACGGACTGGTTGACGTGGACGAGCTGGTCTCACGTGCTGAG
CATGATGAGTATGTGGAAATGAGGAATGAAATAGTCTCAGGCCGCGGCGATTGGCATGACCACAATACTCTTTGGTTTAGGAGATTGAACATGACA
TTGGATCTTCTCGACTATAGCATGCCGGTGATAATTTTTGTGCATACTGAGGAGACTGCACTTGAAATAGGTGCAAAGCCAATGGCATGCTTGAAA
CTCACAGAGCATGCACACGAGATGAATATCAAGAACAGGGAAGCGCGATTCAGGCAGTTTTCGCGTGAGGGGCTGGAATCATGCAGGCCGTCAGAT
AGGGTGCCCAACCAGCAGACCTTCAGTAGCAACAGAGATCTGGAAGCTTTTTTTCTTGAAGTTATGAATGTCTCGGGCCTGCCAGTTGGGGCGCCG
TTCAAGTACAGCAGCTCAGTGTGGAACAATTCATACGCTAGAGATACACCAGGGTGGGTGATGCGCGGTGAGCGGGCGGGTGATCCCACGGTTAGT
GTTAATGAGTTGAGGCTGCTGTTCGAGAATGGTAAGGTGCCTAAGGAATGTGTCGATTACTATGTCAGAGCGAGCTATGTTCCTACACAGTTTGAC
TTCGGTGTTACCATGTTTGAGTGGTCTCAAGCATTGGGCCAGCTGCCTGCATGCTACAACGAACCACGTCAATTTTCTACACTAGCTGACCTGATG
CAAGTGTTTCCGCCACAGTCTGCTAAAGAGCTAACTCGGGCAAATGTCACACTGAGGCAACTGTGCCATACATTTGACATCCTATCGATGCCGGAC
GCTAAGGAGATAGCCGAATATCACGTGGGCGAGAGCCAAACGTTTGTG

>c51680_g1_i1_len=860
CTGGGATAAATGCGAAGTGGACGTACGACTGTATGCAGGCACCCTGGATAAGTTGATGTCGCGAGCAAAATTGACTGGTAGTGCACGCGAAGGTAT
ACCTAACCGGAACGACGTAGCCAAGAGTACTGGGTGGAGTCAGGACGCTGTGCGTGCGTTCCCAGACAACCGGATGGAGTCGCTGGTTTCGCTGCT
AGAGCAGATGCAGACAGGGCAGTCCAAACTGACGCGCTTGGTCAAGGGCTTCTTGCTTTTGCTTGAAATATCAGAAAGGAGGGAGGTTGACTTCCA
CATTGGTGACCACATCCACGTGACCTACGGTATACAGGAAGTCTGTAACAGCTACGATTTAGGGGAGAGATGCTATGTCTTCACATCCAAACCAAC
ATCCGCTGCGCACAGTGCAGTGCTACTAGCGATGTGTCGTGAATACCCACCACCGCAGTTTGATTCACACGTGCATGTTCCCGCAGATGCTAGAGA
TATATGCCTGGTCTCCCAAGGCCGGCAGTTGCAGCCTGGGACAGCTGTGACACTCAACCCCGGGTTGGTTTATTCAAGCATACTAACCTATGCCAT
GGACACCAGTTGCACGGGGCTACTACAGGAAGCACAGATAATAGCCTGCTCATTGCAGGAGAATAGATACTTCAGCCGGGTGGGCCTGCCCAATGT
GGTGTCACTTTACGACCTGATGGTACCAGCCTTTATTGCTCAGAGCAGTCCACTAGAGACGGCACGGTTGTCAGGGGACCTGTCTAAGGCTGTTGG
CCGGGTTCACCAGATGCTAGGCCTGGTAGCTGCTAAGGACATAGTTTCGGCAACGTATATGCAGAGTTCGCGGGGGTTCGACCCAAGTGGGG

>c53047_g1_i1_len=243
CCGAAAACGTAATGCTGGGGTACGACATAGAGTTGCCAGCAATCCTGCTGACGGTCCCGTCGCTGACAGCTGTCAACACTGCATTCGGTTTGACCC
GCGGCTGGTATGGTGACGGGGACACACTCAAGATGTCCAAGAAAGAGCGGCGGGATATGACTGATGCTTTGTGCGCCGTTGGATGGATGTGCGGGC
TTAGGAAGTGTAGACCCCAGGTGTTCCGGAATAGGGCGGGGAAGAAGCAGG

>c53928_g1_i1_len=458
GTCGGCTTCTTTGTGAACATCTCAAAGTTACGCAGCATGAAGTTATCACTGGCATCTGTATGCTGCAGCCCGTAGTCTCGCATCAACCCGTTGATT
GTTAGATTCGTCTTGGGTGTGGATGATGATACGACAGTACCCATGGCACCAAGTGCCTCAGAGACACAAGAGGCAACACCCTTAAAGACCATCCCG
TACATCAGGTCAGACACCAGCGCCAGGCTAGTTGAAAAGAAACCCGGCATCCTGGTTATAACCTGCCGCTCTCTAACCTGCCCAAAGGCCGTGTTC
ATAGAGTAGCTAAGAGGTATCATAGCATTAACTTCCCCGGCAATCAGTCTTAGATACTATATCGCTCATCATCTTGCATTTTCCGCGCATCGTCTCC
AGTGCATACATGAGGCAACTGACAGCATTCAACCTCTGAGCAGTCGTCCAGATCATGTGGTATAGCCGGTCTGG

>c9421_g1_i1_len=1339
ACACAAAGACCAGCCCCAAATTTGGAAAATAAAAACAATAAAGCAATACTAATTTTCAACCTTTTACAATTTCCTTATAATTTTGCAAACCTGATC
ACTTGTCAATTTCAAATTCTTACTAATTCAAAGGGCTGGTCTTCTGATTGATGATGAGGTGCCAATTCAAAGCTGTAGATAAATTTAAGCCATAAA
TGACAAATCAGCAGACGGAAATCGTTGTCCAAATATGTTAGATGGCAAAACTATCTCTGTAGCCAACTGATTCTGATCATTTAACATTCTCAAAGC
TTGACAGGCTTGGAACCTATTGCTCTGAGGGACTATACTCGAAACGAATGACGCTCTTGCAAAGGGTATTACTTTGGCTAGAATGTCAGCATCCGT
TGAGCCAGATGTTGTGCTCAGGAGCTTATTCTCTTCAATTAAATATAGAAGTAGAGCTTTCATTAGTTGTGTTCCTTGGGTGCCTCTTGGGAAAAC
TGCCGCTGCAATCACAGTTTGCATCTGACGTGGGAAGATTGTCCCAAAATCATTCACATTAAGTGTTACAGCCCTTGGTACTGCTACAGTTTCGTT
GGCAAGGATAGTTAAGGTGCCAACAGGAAACACTGCAGCAACTCTTGACAGTGTAACTACAGTGTTCCCTCCTCGTCCCACACTATTCGCAAGATT
ATAGATGGTCCTGAGCGCAGTAATTCTGGTCTTCCCTGTTTCATTAGTTCGAGTTAGCATTTTCTCCACATTATTGCCTCTGCTTAAGAAGAGAAT
GATCATAGTGCTCACGTCATTCTGAATGTCAGGTCTTGATCGGCCAGCTGCGTTTCCCCTTCGGAAAATTTCCCTAACAATACCGAGAACATCAAG
TCCTTGGTATTCAAACTCAGCCAAAGTACTACGTAGAACGGTTAAGAAGGAGTCTTCGGAGACCCGTGCCACTAATGCGTTCATCTCCATACGCAC
ATCATTCAGATCCTTGATTTCTGCCATTTTGAATTCACTAGCAGTCGTATCTATTGCTCTGTAAATTGGTCTCTGAATTTTGGTGGAGTCAATCTT
GTCAATTTATCAGTGCTCCTATTATTAAACTTAGTCTTACTATAGGATATTTGTGCTTATAACTGGGGTATTTGAATATTTATAATATTTGTAGGA
ACTATAAATAGGAAATTAATTAAAACTTAAAGTCAACTTATTAATTGGTGGTAAATGGAGCCTCTATCTTAGCAACTAAAGCTTATCATACGATTA
AATGCTTTAACTGAAATTCAGTTATAAAATACAATGCAACTAAATCCTACACAACACAATGAAGGTTAAAATTGAAATTGTCTATTAAAGG

>c9498_g1_i1_len=3842
CACAAAGACCAGCCCCACCATTGGAAAACAAAGAAAACTAACACACATTTAGTCTCACTTGTTTCCCAATTGTTTTGAGTCTTTGTGTTTAAACAA
ATTTAGTATTTTTTACCCTTTCTCTGTCCACTGAACTTTCTACTACATACAACTCTGGCTACACACTTAATTAATCCTATGTATAAAATGTAGATC
ACTGGTATAACCAGCAGAACACCAATAACCAAGTAAAAATACCTTACGAAGCCAAAGTTTCCCCAGGGTATGCTAGCAAAGAAGTCAGATCCTCTT
CCAAGCAGGTTAGTTTGATTTTGGTTCTGAATTTTAATGTTGTTAATCAATTCTCCCCTTACATCTATTTCCACAGCATTACCAGAGCAGCTAATA
ACGCATTTTTCTTCTATTTCTGATACTGAGAATGAAAGCTTAAATTTAGTCAGAGGGTTAGCTGATGTCACTAATTCTGACCCAGTGTTTAAAGCA
GATGGGCACTTAACAGTAACTGGACTCCTTACTGAATTAGTTAGTATAACTTCTACTTCTAATGTTGCTCCTGTACCACAATTAGCACAGCCTCTC
AGTTTCCTAAAGTGAGCCTCGCATGTTGCCCTAACAACTGTGTACGAGATCTTATAATCTTCCATCCTTATCCTAACATTGTAAGAAGCTTGTCCA
GTGTGATGTAGATAAATGATTCCTGAGGCATCTTCTGATACCACTGACATGGTCAGATTACTAGGCAAAAGGTTGTTTTTCCATTCTTCACTAGCA
TTGCTGAACCTATTGACAAAAACGACATTGTCATCCTGTGCAACAACATGAATGGAATTGTAGTCAATCAAGCAAGATTTAGATGCCCTCTCAGCC
AAGGTTGGAGTTGCACATTGAACGCCACCAATTTTACCAATTATTGGGTGATTCCTGTCAGAGCAGTCCACTATTGCAGTTAGCATTGATCCTGAC
AACTTTCTCAAAAAACATTTGCCAGTGATGGTTATAACAGGCGTATTGACAGATAATACTTGTATTTGCCCTACAATGGTTTTAGTAGGCAAAGTG
TTATCCAAGATCAATGTTTCATTTCTCACTCCATCCTTGCTTGACACAGTTATGTTCACTTTGACTTCCCAGAACCATTCAGAACACCTAGAAACC
TCATAAGATTTCCTTTCGGTGTTTTGTAATTCAATTTTATAAAACTGACAAGCTTGGCTAGCATAAAAACAACCCTTACCCCAGAAACCTGTGACT
CTTGCGCAACCATGAAACCCTGCTTTATCCTTATTCGAAGATGGGACAGGCACCGGGCCTTCGGTCTTAATCTTCTCACAATTGTCATCTTCGCAT
TCCCCGGCATTTCTGCATCTGAAAACATTCAGTAGCTTAAAATCTGCATCGTTAGTGTAGTAGAGCACACTCTTTGAACACTTTTGTCTAATAGCA
CTAGTTCTAAGCTTTATAATGTGAACTACATTTCCAGACTGATCATTAACATTTATGCAAGACTCCTCCCCAAGAGAAGATAGAGGCACTTCAATG
ATCGTATTAACAGCGCATTTGATCTCACCAGCGGTTGATGGTAAGCAACTCAACACTTTACTATCAACTGTTATCACATTTTCACAGGTCAAGGCA
CATGGTATCAAGATCAAAATTACGACAAGACTGACACTAAATGGCTTATTCATTGTTCTAACTCTAACTATTTCATCCTGTTCATCTGCAAATTGC
GCTAGTCTTTCACTCGTTGTGTTCACCATGGATCTGGACTTCCTGGTGCATTTCTTACATAGTGCTCTAGTTATCTTATAGGCTAACTTGGCAGGG
CTTAGTAAGACATAAAGAGCTAATTTTGCGATTTTATAAATAATGCACGCTAAAGGTATAACCAAGATTAGAGAACTAATAGCTAACACTAAAATG
ACCCAGTTCACAGCTTTGTAGCAATGAGGGTTGGCAAACCTAGGTAAACATAAGTCACAATGCAAGGCTAAGCATGTGTGTGAATCATAACACGAA
ACTGATAAGACCGTTTTCCCCTTACCATCACTTCTGAAGTACATTACTCTAAAGTCAGTGACATGTAAGAATTCTGATGGCAATACATAATCAAAA
TCTGTTTTCTGTTCGTTTATCAGTCTGCAGTTATCGTGCACACACAAATGAATAATGTCAAATTTTATGTCTCTTTCAATCCTCAGAGATCTTCCT
CTGCAAGAAACTTTACATCCACTACACTGTACTGATTCTTCTTCTCTTACTGGAATAGTAACATCCCAGTTAGCATGAGCAACAGCATCAATTCCT
ACTTTTGCTGATCCAATTTGCATGTACAGGTCAGAGCAATCTTCACAGTATCTGCAAGAACAGTTTGCATTTGGCCATTTACCAAACACACTTTCA
TATGCTTCATCCCCTTTACATTGATGACAATCTCTAGGACAGCCACCGTCGCAATTGAAGAAGCATAGGTTTGTCACATCTTGTCCATGAGCAACT
AAAACATCCTTGTCCGTGAATAGCCATTTTCCACCAATAGAATACATTGTGAATCTTTCAGTCGACTTCACTGGCTTTTCACATTGTTTACCGTTA
GTAACTGTTTTCCCCTGGATGGAGCAGTAAGTTTTAGACAACTCTTTTAATTCATCAGGTAGCTCGTTCAATGGAACCCTATTCCTGATTACGCTT
AATTCTAGATCCTCAATGTTGCTAAACTCTCCAATTGGCTTAGTGTTATCACAGTCAACACCGTTGAAAGAGTGATCTGAGGGGCAAACAGCTACT
CTCACACAAGCTTCCACTCCTGATTTATAACTAGCACTGATTACATTTCTATAATGTGACTTTTCCACAACACCAGACCAAGCAGTTTGAGCATCA
GAACACACTCCAAGTGCGGTTAAAGAGCCCGATTTTAATGTTGATCTAAAAGGAACCTTTGACATAAATTTTACTTCAGCCAATGGTCCGTTTACT
CCTGGTCCAAAGTTCCTTGTGCATGCCCCTGCGCTGATCATTGCTTGCATGACATTTCTCTTCTTTCTCCGATGATCTAGATTTAACCCACACTTT
TCTTTATGTGCCTTGAAGGCGTCTTCTAAGAAGTAAGCATCACTCACCAGTGTTGTAAACTCACCTAGTATCTTGGCTAAACCCATGACTTTAACT
CGTTCATCATAATTGTTTATGACATAGTCAAAATAAAATTTTGCCAAACCTGAAATAGTATGAGGACCTGTAAAGCCGTAATGAGAGTGAGCTAAC
TTTAAGACTTCATGTCTTCTGGTTTTACTCTGAGCAATCAAATCCTTAACCACTGCATAGGTCTTACATAACTCCGGGTCTATGGTTTCTTCTCTG
TATCCCATGGCTAGATACTCTGCTTCATCATGATTCGTTATTGCAGGATAGACTTTATGATCAGCCTCCGATTTTACAATTGTTTTCTTTGGAATG
GTTTCTGTCTCAGTGGTCAATCTATGCCTGAATACTAAATCTAAAGTGCCTTCTTTCTCACTCAAATTCTCATTTCTCCTTAGCATTTCATTCAGT

AAGCCATTCATTGATGCATGCATCTGCCCTATGGGATCCATCAATCTAGATATTTTTGAGTCCAGGTCTTCATGCCTTTTCAAATTACAACTCGCC
GCTGCATATATCATTTCAACTGCTCTACTGTTCATCCCATTACTCAGCAGATCATTCTGCAGTGCTGAAAGATCACTTATCGATACAGTAACTTCT
CTTCTTTCCGCTTTGACTCCATTCATCATCAACATGAGTACCAAACTCACTCTTATAGAAATCTCCATTTCTTCGTTAAAATTAGATGTGTTTACT
TA

>c9500_g1_i1_len=6697
TATAGACTCCTTGTCAGGAATTCAGGGGGATGTTGTCACTGGAGATGAAATTCTCTATCACGATAGTGTTGCAGGCATTCCTATTCCAGAGGTTCT
GGTTGATGTGCGTGGTGACTTAATCGGGGTTTCATTCAGACCACTGGATGATGATGATGTGGTTTCTACTGTCGGTAGTAGCGTTTATGGTCAAAG
CTTGAGTTATAACTTGGGTGATTGGAAGCTAAACAATCTTGTGCATGATTTTACCTTTGGAGAACTAATTGAGAACACTGATAGGAAGTTAAGTGA
GGTTTTCCAAAGATCAGGTGCAGAGATCACTCGATTTACTCCTGATCATGTTAGTGAAGAACCAGATTTCAACATAATTGTTGAAGGAGGCACAAA
TCAAGGAAGTTTGAGATCAGTTAAAAACTATTACGAAGAAAAGAAACAGAAATATGATGCTTTGTTGAGAACCATTCATCAAAGTGAGCTTGCAAA
TAAAGGGAATGTAAAAAAGATCCTGTTCGGGATCATCATTGTTGGGCATGAAGCAGTGGCAACAAACTTAGCTCTTTCAAAAGAGCAAGTAATTGA
GTTGCAGATGAGATTTAGAGTAGGTCTAAGAATTCAAAAAGAATTAGCGGAGTTAGGCTTGGTTGCTAAACTACCAGAAGATGCAACTGAAATAGA
TAAGAGATTAGAGCAATATTTCCTGAAACTTGATTTACTCAGTGATAAGGAAATAGAGAACTGGAATAAATCAATAAAACGGCAGATAGACATCAA
CAACCCTGATAAAGACTTTCTTGAGCTACACTTAATGAGGTGGGATGAAGATAGGATAAAAGAGCTCATAAACCATCCAGATAAGGAAGCAGCTGG
CATCAAAACTGCAAAAATTCTTCAGGAATCCCTGAGTTTTGTTGCGGAACAGACCACAAAGTATGAGTTTGACGAAGAAGCTAGAAGAGTTAAGAG
CCAATCTGCACATGCTGATTTTGAGAAAGAGTACATATCAAGAAATGAGGGAAACATGTTCACTGCAGAGAAGGCATTCATTAACTTTCCAATGAT
TGTTATTAAACCATCCGAGCCTTCCAGGGTGCCCAGTAGAGGATTAATGCTTAGCGAAGCAAAATTTGAGACTGTCTATGACACCCTATATTATGC
TGGGATCCTGAAAGCAGTAGCTGATCCCTTGTACTTCCCTGATCATGAAGCTGACATATTAGAGCAGTACAAATGGGAAACTCCGCTATTGACAAG
CAAAGAGGAATTGCAGCAGTTCAAAGATGAAAATTTACCAACGCTTTTCCAAATTGGTGGGACCTTTAGCCTGAACAACATATCTAGTAGTTTTCT
GGATTCAGCGCTATCACTTGTAACTAGTGATGTGATTAAGCAGTCTGAACTGGCGAAAGACTTTAAAGGTAAAACCTCTAGATTTAACGCTTATTT
GACTGACGCAAATAGGTTCGATTTAGCTCTAAAAGGTGTTCAGGGTAAAGGATTAACATCAAAGTTCATAAAGGGCAAGATAAATGTATCATACAA
TGCATCAATTGATGAAGCCAGAGAAAGCAAACCAGATTGGTTCCACATAACAGACACCAACACTGATGACATGGATCTATTGATGAATGATACTGA
GCTGTTTATGGAACTCACATCTACTCTTCACGAGACAACTACAAGTACAACTTTATGGCAAAGTCTAATCAACCAAGCAAGAGACTATCATGGTGA
CAACTACTTAAGGTTAAAAGAGGAACAGCATCAAGCGTGGTTGTCAAAAACTAAACTTTTTAATTGGTGCAAACTAATCACAGACATAGGGACTGA
AGTGGACATTAGCATGAAACAAAACTGCAAGAAGGATGAATTCATATTTAAGAAGTTGCAAGATTTCAATGTCTGGTTACTTATAAAACCAACTAA
AAAAGAGGAAAAAATATTCTTTTCAGTCTTATTTCGAAGAGAGGACATTGATTATCTTTATGAAAGCACAGTTTTTAAGAGATTAAACAAGCTGTT
TGGAGGAGAGAATTACTTATACACAAACTTATAAGTTAACAGAATCAAAGTTGCTAAACTGGGTGTTAGTGTTACCTAGAATGATTGGTTTATT
TAGGTTTTGGAGCAATTTTTCTAACATCGAACCCTACAGCGAACAGAACATCAGAGATTTACCAGAAACGCATCCGCACTTCATTGGGTTTTACAA
GAATGCAATGCCAATGTTGATTCTGAGCATAATTATTGGGTTGAACGATAAAACTGAGGTTGAAGAAGAGATAACGAGAACAAGATACATGGTAAT
GGAAAGCTTAACAGAGTGGCCAATTGAACCCAAACCTTACAAAATGGCTAGTAAAGCAAGCATGGCAATGAGAAGCAGGTTAACATTATGGCTGTA
CAGAAAACATAAAGACCTGTGCCACAGGTACACTGCTACACCGCCTAGAGCAAGAAATGAAGATGTTACTGGTCAAGAACAAACTGAGACTAGTAA
CCAACTGTTCTGGGAAGGATTTGTTAACCCCTACACAAATTTCAAACTAACAACAGGTCAGCAGGTAATAAATTTATTCTATTTAGGGTACATAAA
GAATAAAGACGAGACAGCACAAGCAAATAAAGCAAGCAAACTTTATGATAAAATATTGACCTATGAAGTCCAGTATAACCTTGAAACTGCTAAGAT
ATTGGGCTTGACGTCTCCTCAGAAGCCAAGACCACATTGCTTTGATTTGGATCTTCTTATTGATTGTGGGAGGAACCTAAACCATAGAATAAATGA
GTCATATCCTGAGTTTAACTACAACTTTATGAAGGAACTGCATAACTTCATGTTTTTGCGCTCAGTGGAAGAAGAATTTTCAACATTAAAAGCTTC
TAGCAACTTTAATGAGGAGCTGTATGATGAGGAAGAAAGTTAGCAGCAAATACCAAAGAGGCAAGGTCATTGAGAAGCTAGCACTAGAAGGAGAAGG
GGCAACAAGCATTGCTGAGCTGTTTGAGAAAAAAATACCTGAACGCTTTGAAGTCAAGAGGCCTTAACATAGATATATTCAGAAAGCCGCAGCATGG
AGGAGATAGAGAGATCTATGTCTTAGGTTTTCAAGAGAGAGTTGTTCAGAGACTTATAGAGCAAATCTCTAGAATAATCTGCAGGTTCATACCTGA
GGAAACAATGACTCATCCAGGAAATAAAACTGCTATCCCAGAAAATAGCTCAAGAGAGGCAAGAACTAAATATAAAATGGGACACTTAACTTTTAA
CAGCTCAGCAGACGCCTCGAAGTGGAGCCAGAACAATAGCAGCTTTAAGATGATGATTTGCTTACTAATTATTTGTCCTCCTTATTTGCACAAAAC
TATCATCAGGTGCTTACGGTTGTGGGAATTCAAGCGGATCCTAATCAATCCTCAAATTTTGGAATTGTTTGACAAATATAGAAACCTAAAACTGTA
TGACAAAACCTTACAATGCATGTTTGATGGATATAAAGGACTTAAAACATTTAGATGGATACGAGCAGGGAGTCCTTATATTGAGGTGACCACAGG
AATGATGCAGGGAATCTTACATTATACATCATCTCTATATCATTCAGCCATAATATCCAGAGTAAAGGAAGTGGTTCAACAAAGCAGCCATGCAAT
TCAGGAGACTTTGAAATATCCAAGGCCATTCAAACTTATAATGTGTCATTTGCAGTCTTCAGATGATTCTTACTTTAGCGTTAGTGCACCTTTAAC
AGGAGATGCAAGTATTGCCACAAAATCACGTATTTTGGCTACTGCAATACTGCAATTCAAGGTTAATTACTCATCGCATTGTGGCGTCGTGAATTC
GATTAAGACTGTTTTAAACAGCAATCATGTTTTTGAGTTTAACTCAAACTTCGAGTTTGGATTCAATCATTACAAGCCTGATATAAAGGCTATATT
CTCAGGTTTCCTAGTGTCTGAGCAAGAATTGTTGCTAACTAGGCAAGAAGAGTTGAGTGTGCTTTTGACCAGCTATTTGGAAAATGGAGGGACTAA
TTACGTTGCTAATGGCTTACAACTTGGCCAAAGCTACCTACACTATCATCTGATGGGTCTCACAACAACAAAGTACTTCAGAACATATGAGGTTCT
GCAAACTATTCTACCAGATCCTAGCATTGGTTTTTTTGTCATGGATAACCCAATGTGCCCTGGATTATTGGGTTTTAAGTATGGACTATGGAACTT
GGTGAAATCCTCCAATCTAGGGAAACTGTATAAACATAAATTAAGACCCATAACCCTGACAGATACTAAACTAGAAGAGAATGTCAAACTATCAAT
TGAATTAAGTGCACATGGTTCCCTATCAGCATCATACAAATTGATACATGGGAATAGGAGAAAGTGGTTAGCTTTGCTGGAGAGGATGAATGTTGA
CCAAGAATGGCGAGAAAAAAATACAGATGAATCCAGAAATATTATTCAGGAGGGCCCTAAATCCAGAGGAGGTTCGAGTTAAGATTTCACAGAAAAT
GCACTCCCCAGGTGTTTCTGCTTCCTTAAGCAAATTAAACACAATCCCACGCATTCTAGCTGAGAGCGCATATGTCCTAAAATTGCAAGCTGTTAC
ATCATTGAGTAACTGGATTGATCCCAGACGCACTGAGTTTTCCAAGGTTACATTAATAGAGGCTATTTTAAAAGAAATGAAGGAAGTGCTAGCTGA
CGATGACATAAATGAACAAGAGATGCGGATCCTATTCCCTTTCCATGAGGACTTCACTAGAAATCAACAACTGCTAAGTGGTGTTCACGTCGTTCA
GATAGGTCAGTCATTTAGAGAATATAAAAGGAAGGTCACATCAGTTGAAATAGCCACCAACAATGAATATAGTTTATTAAGTCTTCGGGGAATCTT
GTTATCATTCTGGTTTGAGAATAACACTGATATCATGACTCCTCGGTTGAGTAAAGAACATCGAGACTACATATTCACACAGCATCAGAAAGTGAT
TCCTTGGATAAGGAAAGACATAAACGAATCATTAGCAAATTCACCATTCAATGAGATGGTTGAAATGCTACTATGGCTCAATACTTTTTCAGGAAG
GAGAAGGATAGTGAGACTGCTAGGAACTCAAATTATCTCAAAACATGGTCATAGCAGGTTGTTATCAGTGGTCATTAACAACTTATCATCTTCCCA
TAGACTAAATCTCGATAGAGTAGAACTGGAGAGCCCAGTTGATTTAATCTCCACTGTAAGATCCAAGTTCACACTAGCTAGCTCGCTACCTGGGGA
TCATATTGATATCATTAAAAGAATATTTTCATCTGCAGGAAACCAGGTCACTTTCGTTGAAGGAGCTGTTAGGTCAAAGCAAAATGACGTTGTGCT
AATGTGTAAGTTGGCCAGGGTCATTGATAAAGGCTGGAAGGAATCAAACTCTGATGAGGTCTTAAGCATTATTAATGATATGAAAAGAAATAAGCA
TGGGACATTAGGGTTGTTTACGACAAGACAGAGAGTATCACTGGCAGAAGATGGAAGTCCACGATATCATGGTTTGGGAGTGTGGGAAGGAATCAT
TGACGGCTATGACATAATGATTAGGATTAGGAACACCATTAATAAGCCTGTAACAGTTCAGAATGTCACAGTAAACACACCACTAGCATCAGTTGA
GTCAATAGCTGTTAGAAAGTTCATTGAGAACATGGGCTGGGTTATTCCGTCTGATATCCACTCAGGAAGGTCCACCTTGTTCTTAAATGATAAAGG
CTTGAATTCCAATTCAGGGGTGCCAATAATAGTGGATGAAGGCTACAATGTAAACATAGCCGAGTTTTATAAACTACAACTAACCTTAGAGTGGAC
AGGGAGGAACCTTAGATGCGTTGCAAAGTTACGGAAAGAAACAGGTCGAGGGGTAAACATCACAGTCCTAAGTCTGAGCCCTAAGATTCAACACTA
TGAATATATGGCAAAATATGAGAAAATAATGGGTAAGGGAACACTGGGAAACCACTGGCTCGAGAATAAACCTCTTAGCCAGTCTGCAGCAGTGAG
GATTATTCGGTCTTTCAATGAGCACTCTAATGATGAGAAGAATTTAATTAGAGCTAATCTCAAAGCTAAGCTGCAAAGCCAAGGAATTTCGTTTAG
GAGAAAGATAGATCTAATAGACTTAGCTTTCGTATCAAAAGAAGAGCAGGATGAACTTTTGGATGATTACATGCTATTCCAAATGCCACTGGACCT
TGGTGATTTAAACATAGTCGATTTCGAAGAAGTAGGTGCATCTCCTGAAATTGTTGAGTTTGAAGACTTTGAGCTGAGGAATGTTGAAGAACTAGC
TGATGATATTGCTCTAGAGTTCGAGGAACGCACATCACCAATAGAAGTTATTAAAAGAGAAATTTGGAGACTCCACCCACTGTTCAAAGACTTCTC
TAGCCTGATTATTTCTCAGATAGGAAGGGATGCAATAAATAAGCTGATAACTAGAAGAGAATATAACAGAGAAATAGAAAGTTATCTACCAGTTTT
TGAAATAATACTAGAAGAAGATAGCTCCTCTTGGACTTTAATAACACCCATCAACATGTATGAACTAGCTGGGGACGACAATAGCGAGGACTCTGA
GTAAATCGAATTTGCATTAGATTTGGAAACTTAAGGTTTTGAATTAACGTAGAAGAGTATTGTTTAGCATTTA

>c25711_g1_i1_len=2417
CGGTATAAACACATCCTTTCATCAAACAATGACTAGCGAAAACCAAGGAGGATTTGACAAGAAGGGCCTTCTAGCATTGGCGGAGAAGATCATTTC
AGCTAATCAAGGCACCATTGATATTCTCAAGGATACCCCGGTCTGCAACTTAAAGACCGTCCAGAGAAATGCTAAGAATCTAAAGGATCCAGCGCC
TCTGCAGAGCATGATTGCAGTAATAGGAACAAAGTATCCGCTTTCAGTCGATTCTGCGAAAGCAGCAAAGCATGGCATCCCCTTGCATTTCCTGGA
TAGAGATGATGCACATCAGTTTGGGAGGAGGCGTTGCAAACTCGAGGCGGTGCCGTGGTTCTTAGAGAACATGGCAACCCCAGATGAGGGGATCAA
GGAAGCCATAGACATCTTGTTCAGGGCAAAGAAGGCAGAAGTGCAGGCGCACTACTCGGTACCTTGGAGAGAGTCACGAGTAAGATTCGGGAAGAC
CTGCCTGCAGAGACAGCTTGTGCCAACCCAGGAGGTGGTATTCAAGGTCCCCCATAACCAGCGTGTCGAATACCTCATGACAGCGTTAATGCCTCA
CCTCTCGATCATGTACCCGAGTTTGGATCCCACCATCGTGAAGGAGCTCAAGGTGCTTAGTAACTTGAGACTCGAGTCTAAATTGACTATTCAGTC
CCAATTACGGATTTTGCTCGACCTGATGGATCCAAAAATGCGCTACATGCCTGCACTTCCAGAGTACCCCCACTCAGTCAACAGGGTAAGGTATGT
GTATGCCTCTGGGAACCACTTGATTCTGGTTCCAGTGACTGCCAGTCGAGATAAAGGCATCTACACGGAGATCTTGACACTGGTTGGCAAAGTATG
TTGGTATTTGTTGAACAACAAAAGGAGTCCAGAGGAGATAAGAGCAGTTTTACAATGCTGCACATTCAGAGGTGTGAGCCTTCTCGTAATCATCCA
TGAAGAGGTTGACACTATCAACATGTACTTGAAGCTGCTGTTCGCTGTGATGGGGCTTCCCATTTACGTGGAGAAGGTCGTTAGAGGATACACTTT
CACGCCCATCAGTGGGAACATGAGGCAGGAGCTCACACGGAACAGTGCAGGGACAAGATATGCAAGTTATATCGGGACAGAAAAGGTGCAAGTAAG
GGGTGAAGGTTTTATAGCTAACATCACGCGCCACGAAGATGTAATTACGCGAATCGTGTTACAGAGATGTGATTTCAACATATTCCTGCGTGCCTT
GGCAGCCATCAGCGTGTACATAAAGATCGGATTTCACACCTCGAAGGCTAGGAGCATCAGAGCGATGGAGGAAGAAACATTCGAAGGTGCCACTAA
GGACCCAGCAAAGTATTTGAGGATGTCAAGAGAAGATATAAAGACAGCGTGGAAAAGTGATACAGGATACCGCAAGATAGCATTTGAGGATTACAT
CGAGCCTACCCCTAGGGCGCAGAGGTATGTCACTGTAGAGGGGACCACAATCCACGTTAAGGGTGGTCAAGACTTTAACGTCCAGAAGCCGCGAAT
ATATCCCCTGCTTCCCATTGAGTTTAGATGCCAGAGGAGCCTCCTAAATGATTTCATGTCTCCCCCTGTGATGTTAAGGTCCAAAATTGCTTACTA
TGTCCATGAAGGGAATTTCGCTGAGATGATGCGGCGGGCTCAAGCTGACGATTGGAACTGGCTCAATGAGTGCATAATGGAAGGCGTCGAACCCAC
ACAGCGTATCCAGGTGGCAGTGAACGCGCGTTATGCATTCAGAGTGGTCATGGAGCAACAGGGATTCCGACACTATTTGGCATATTTGTATTGTCT
CTGCCACTACAGGGTGCCGATAGCAGGTGAAGTTGCTCAAGACATTGAGTTTAGATTCTACAACAGTTTGCGGTTGTCACTGTACCTAACTACCCC
TGACGCCACGATGGTGGATGGGGAATTCCGCATCGCAGGGAGTGTGATCGCCAGGTACGAGGAAGGTGTGTTCACTAGCTTAATACCAATATTCTT
AAGAGGATATCGCCTCCTGGAGAACTATGATGGACCCTTCAAGATTACCAGCGTACCTCGTGCCGCGGAACTAGCGCCAAACCTCAAGCCAGGATC
CGTTTTGAAAGTGTTCTTGTTCGGCAGGGAATATTTCCTCGAGAGAGATTTGTTCTTCGAGCTGTCTGTCCAGAGAACCATCGCAGTGCGGACCAG
TAACATCAATTTACCGTCTGCCTTGCTGAACTTGATCTCGTTGAATAAGAGGGTTCACGATGAGGCCGACATTTCAACAGTCCCTGTAAAAAAGCC
AAGGTGTGAGGAGGTTGCCGGGTCAGACTCCGACGTTGAGGACGTGGGTTATGACATACAGGACATGGATGAAGATATAGAGCTGGACGACCTTTA
AAGTTTTTAGAAAAAGG

>c45853_g1_i1_len=203
GTCTGCTGATGCTTCCAAATGGTGCCAACGCCATCATGTCTCACATTTTGCATGCATGTTGGTAGGTATAACCCCACCATTTCTACATAATTTTAT
ACTGCAGAGCTTAAAGCTGTGGACAACAAAAAGAATCAGTTTCCCTCTTCAGTTGGTAGCAACATTATTGGCAAACAAAGAGACATACACAACCAA
TGAGACATTTG

>c2560_g1_i1_len=2481
AAACCACTTAGCGATCAGCAAGGAAGTGAAATCCCCCGTCGACCGAGCGCAGAGCGCCTTGGGTAAGTCGATCACAGCCTGGGTGGCTACAACAAA
CCCGAACCCCATTTGAAGACCATCGCCTGGAGCCGGTTTTCTGTCTCGTAGCAGGACGAGTTAAAAGCTTCCGTGCATCCTCTCACTAACCGTGAA
GACAACCACCATCCTCACTACCGACCTGTTACGCGATCCGCCTAACGGGACGGGCGCAAAGGGTAAGGAGTGCCAGGGGATAATTCAACCCTTGGG
GACCACTCCTAACTTTTCCACGGGGGGAGTCGAGGTCCCACAACCCCCCTATCCCAATTAAACGCCCCGTATCCGACGTGCTTCGCAGGTTACTCA
TGAGGGAGGGTGCTGTCCGAGCGCCGCCCCCAGGATGAAATTCTCATACGTGGCCCCGCCAAAACGGGTCGACCACGGAGTTACATCCGTCCATTC
ACTCGGGTCCTCCGTCTCTAGAGCCGTAGGCACCCAAACCTTTTCGTGGCGAGCCACGTAACCCAAACCCGAACGGTCCAACTCCGACGGGATTCC
GTGGGAAATCCATCGACGCACTATGGGTGCACCCTTGGTACAGGGTACAACATCGTGGTCCACCCGCTTAAGAGCCATCCGATAAATCGGAAGGCG
CCTGATGCGCTTGCGATCAGACACCCAAGCCCGAAAGTTGGTCTCCCAACCAGAGACATCGACGGCACGGAAATATTCCTTCTTTTGCACCCCGCT
GGTAGGGGCGCCCCCTCGCCAGGCACGGTCCACGAGCTCGCGAACAAACGCGGACTGCAGCTCGTCCTTGCGGAGACACTCGGCCCTGCGGAGCTT
CCTTCCTTTCTTAGGAATCAGGAACTCCCTTGTCCAACCATCCGGGACGCCGGCCCATTGCAACCGGGAAGGATCCACTGGCAGTGGATTCTCCCG
AGGCAATTCGGCGTACCAGCACTCTCTCCTCCACAAACCGGCCTTTTGAAGGGCTTCAACACCAACCGCCATCCCCAATCCTCGGATACACGAACG
ACCCGTGGAACGGATAGCATACGCTCTTTTCTTCAAGAACATAGCCTCCGCTTGAATCCGTTGCTGGCCAACAAAGCCAAGGCAAAAACGCCGGAG
TGCCGAACCAAACCCGGAGAACGACTCAACGGGCTTCGACAAACCTCCTACCCGCAAAACGGGTACCAGGCTAGGTTTACGTCGCCGATGAGAGCG
AAAGAAGCTCGAATTCACGGAAAAGAACCGTGACGAGACCATCGTCTTACCTCTCGACAACTTCAACCCGACTTGGGCAACGAACCGTGACCACTC
GTCGAACTTCTCCTTTGTCGTGCGGAACACAATGTCATCCCCGTTTATACGAACGGGGACCTCTGGGCCAAAGACCCATCGGAAGGCAGCGTAGTT
CTGGACGCACAATAGGGGGAACGACAGAAGGTTACCCATAAGTTGGCCGACCACTTGTTCGAAGGGCTGGGAACAATCGTCATAATGGATCCGCGC
CCGCAATGAAGCGAGCGCAGCATCGCCGAGACGACGCGGAATCCAAGACGAGTTCGCCAGCGCGACCTCCAACACGGCCCTTGCCACCTCAATTGG
CAAGAAATCCGTCGCGGCCTCATAATCGCCGGACACGAAGATCTCTCCTTTCTCGGGTGTGAACTCCCGAAACTTGGTAGCAGTGGCCTCTCCTCG
AAGGAGCCAAGGCAGTCGGCTGAGCCGATTGTAGAGGGCCTTATGAAGGGGTCTAAGCACCCCGGTGCTCGCATCAGAGCGAGTTACCATCCTGTC
CTTCCCGTTCTCAGTAACAACCAAGAAATCAACGTCCCGACCACAGTCGAGATCGTCCCTCCCTTCACGTAGACAATCCCGAAGGAAAGTCCCACG
ATCCAGGCCGAGAGACCGAGGGCCGCCTTTCGAGCGCGGCGCTTGGCGACAGGACTTGACCGAAGGGGTAGAACCCCATGCAAGGTCCTGATACTG
GGAATCCCACCCAGGCGGGAAGCACTCGCGTGCGATCCGTCGACAATGGGCGAGATAACCAGCCGGCAGGGTTACGTGAGCAGGTGAGGTGCACAG
TCTTTCTCTGTGCTGGGACCTCAGCTCATCAGCTTTACCAGTCGCAGGCAGGCACTTCCGGAAGAGGAAGAGACTACCCGCGATAGACATCCGTTG
CGAGGCGGACAAACCTCGAACTTCAGAATACCACGGGTGAGACTCCGGTTCTTCAAGAAGACCCGAGCAAAAACTCTTGGCCAATGGGGGCGAGAA
CGTCTTCTCCAACAAACACTCAGGAACCTCGAAGCGAATGCCATCAGGCAAGCCCGCCAAGAGGACGCTGCGTGTCTCGGAGAAAAGAGACGCCAC
TCGTCCAGCACCGCAAAACGCAGGCGATTCACTTGCGGTGACCATTAAACGTTGATTACAACGTGAACTCCTCGGATTTCA

>c6975_g1_i1_len=1806
AAAATCCTTGCATCATCTAGCTTTCGTAACTAGATACGGGTATCAGTGAGTAACATCGAGATGACGACGTCACAGTTGTCAGCAAATCGTTTCGCT
GGTTCACTTTCACCAACCATCTCCCGCTTATGGGAACAGTATGTACTAGTGTTAGAGGACATTTATGGTGTCCGTCTTCCACGCGTGCTGTTCACG
GGTGACGATCAACTTCGCTCGTTTTGCGAAGGTTTAATTTTAGGAGATGTTTCACATCAATGGCAGTCTGTAGTTCGTCGACTGTCTGCGCGATGC
CGGTTTGGATTCGCGCATTCACTTTTTCTATTTCGTAAAGTGATTCCAAAGGAGAAACCTCCTGTCAAGGAATACGTCGAGCGCTTGGCGACACCT
CAGTCGCCCCCGGACCCGAAATTCAAGGCCTTCGCTTTGAATTTGGCGGGTAAGTTGTTCCCCTGCGGGTGGGACAAGAGCTACCTTCGGCATTCG
TTGACAAGTTCCTTACCCTTGACCTCTTGTTCGGAAGCAGGTCGTAAGGATGGTGGGTCTAGAGGATGGAGCGTACATGAACGTGAGGAACGTGAG
GAGTTTTGTTCCTACGTGATTGACTCGGTCGCTCCAAGACCTAGGGGTGTTTCGAAAGTACAGGCCATTTTGACTTCTGGCAAATGGCGGATCATA
TCTATCCCTCCTCGGGTCGATAATGCTCTCCGCCCTTTGCACAAGGCTATGTACTCTCACCTTTCCCGTTTTAAGTGGCTTCTTCGCGGGGACGCG
AAGGCTGAGGCGTTCAAAGAATTCTCTCGTAAGGACGGGGAAATCTTTGTAAGTGGCGACTACGAAAGTGCCACGGATAATTTAAACGCCTCCCTG
CAGGAAGCCATCTTAGATAAGTTGCTGTGCAACGCTTCCTTGGTGCCTCCGGGCATCGCGGAGCACGCCGTTTCCACATACCGTAGTCGTCTGGTA
TGCGACGGTCGCGAGTACGTCCAAGCGCGCGGGCAACTTATGGGACAGCTTACGTCTTTTCCCCTCTTGTGTCTTATAAACTATATTACGTTTCGG
TATTCAATCCGGCGTCGGGACGTTCCCGTCAGGATTAATGGCGACGATATTGTTTTTCGTGCAACGCCCGAGGAAACGTTAACTTGGGAACGTAAC
GTAGCGAAAGGAGGACTTACCTTGAGTGTAGGGAAAACCTTGAAGAACCGAAAGTTCTTTACCCTAAACTCAACACCCTTCGAAGGACTAGCTTCG
GGAGCTAGGCGGGTGGGATTCGTGAGGGCTTCGGCCCTTTTCGGATCAGGAAGTCTTTCCGATCGGATCGTGTCGCTGAATGGTAGGTTCTATTCA
GCACAAGCTGGGTACGGTCGTGCCCGAAAGCTCGTAGTTCGGCGGGCGTTCGTCGACATCAACCAGCAAGCGATCCATGCCTCACGGCGGTCGGTT

ACAAGAGGGTTGGGATTGGCTGTGGAGAGGAATGTGCTGCATGACTTGCGTTTGTGGCACCGGGAGCTGTTCTACCTTGAACAAGTAGAAGAGCGA
ACCGTCCCCGCCTTCGCGAGGGGGGAGCTTCCTCAAGGATGGTGCCAGGTCAGCCGCAACTGGATACCGGAAACGGAACGCAAGTTTATCCAGGAG
GCCTGGGGCTGGGCGTGTGTCGAACATGCCTGGTTTTCATCCTTTTCACGTGAGAACTTCTCGGAGCGGAGCAAGTTAGATCAAATCTGGGAGGGG
TGCTCTCCCTACGGGCTTGGAAGCTTGGCTTCGGCCAAGGTCCGTAGGATGCTGAGACTCTCCCGCAGGGAAATGTGG

>c13763_g1_i1_len=747
CTGCCATTGGTTGGGCCCCTGTGGTCCCAATCTTGAGGTACGTGTACGTAACATCGCATTGCGTTGTACGCGTTCCACAAGCTTTACTGTTGCTAG
TCGTTTGAAACGATTCGCGGCTGTCCTGTCTGAGCTCTTCGGCTTAGATTTGCAGGTGCCTTCGTTTACTAGCAATCAGCAGATTAAGGAGTTCTC
ATCGGACCTCCTTGAGGGCAATAATCACCCCTGGAGGGAGACACTTCGTGGTCTCCCTATTCGTCACCGACACTCGGTGGCCTTTTCTTTGTTTCT
CTTTCGAAAGACCATTCCAAGTGAGGAGCCCGACGTGGGCGCCTACGTGAGGAAGATGTGTTCATCCCAGGAGGATCCTGACCCGGTCTTCCTCGA
CTTCTGTCGGACTGAGGTTCGTCGGATGTTTAGAAAGGGGTGGGACGCTTCTTATGAGCGTCGGGCCCAGACTACCACCCTGCCTTTGAGCGCTTG
CGCTGAGAGTCCCAGGTCCTCCGGTGGTTGTCGAGGATTGGAGGCTTCGGAGCGGATAGATCGTCAGGAGTTCGTCTCCTACATCTTGAACTCTGT
GGTTCCATCCTCTCGTCGGACTTCTAAGGTGTGTTCGGTAGAGACGGGGGGCAAGTGGCGTATCGTTTCGACTCCACCGCGTGTCGACAACGCGTT
ACGCCCGCTTCACACGTGTCTCTACGATTACATCTCGCGTTTCCCATGGTTGTTGCGCGGTGACGCCAAGCCCGG

>c45380_g1_i1_len=320
GGACGGGGACGTCCCGCGGGACGCAGAACTTAAAGGTGAGGTAGTTGACAAGACACAACAGAGGAAAGGACAGAAGGTTCCCCATGAGCTGACCAC
GACGCTGAGTCCCCGACTCACCGCCACCCTCCAACGTTGTCTCCAACGTAGAGAGGGCATGAAGAATGACGCCGCGAGGGACGTTCGCAGTCTTCT
GCAATAGCTCAAAAAGGATTGTGCGCTGAACCTCAGAATTGAGGTTGTCAGTAGCGGACTCGTAATCGCCACTGACAAACACCTCGCCCGACTTCT
TCTTAAAGGACAAGAAGCGGCCGGGCTTGGCG

>c17450_g1_i1_len=431
TGTCCCAAAAGATCTTAGATCTGTAAAAATAAGATTCGGAAAGTTATATTTATGGAAAAGTATGGTTGATCGTTTAAATATAGGTACATTAGAGTC
TGCTATCGAAATGATTGAAAAAGATCCTTTAATATTATTTGGTCGACATACCAGTTGGGATGAAGAGCTACCTAACTTGATTATCAAAGTGTTTAG
TCCTGGAGTTAAAGAATCTATAAACAATGTTTCTTCTTCATTGCGTCTAATGGCTGCATCTGCATACATGATAAATCGACCGTGCATCACAATATT
CGAAGGAAATGCAGTTAGTAAAAAGTCACTGTACTCTGCTTTAAAAGATAAACTAAATGCATCTAATCATCTATTTAATTCTGTTGAAACTTTGTC
AAGAGCATTTCCCCTACATGCAGAATATGAACCTTTTATCAGGGACA

>c50950_g1_i1_len=313
AAAAAACATCCAAAGCATAAGTGAAATTGAACTGTTTTAATTTGAATTACCAGATGTCACGCTGAAAAATGTATATCTATTATATAGTTAGCGTGG
GATTCCCCTAGGAGGTTGCATGAGTTTTCTAACGTTTGCAGGACGTAACCCTTTCAACACGCGCTCAAGCTTGATGAAGGCATTGAAAGAAAACCG
TGGCTCTTACACCATTAGCCGCGACGGCCAAGTTTCACTTGATCTAAACAGTGCCACTGTTAAGAAAAGCATTGCTAAACAGATTTCAAAGCTGGG
CGGTATTAAGGAAGATAGTTCGAAG

>c24059_g1_i1_len=313
CGATGCTCGTCCAGTCGAGCTATCAAACAATGTAGCGTTCAGACCTCCCAGTCTGAATAGAGACATTGCTTTATTAGCATCGTCAGAAGACACAGT
CGTTAGTTCGGCAGTCAATTGGTTGTAGTGCAACGTGGTTGTGTACTCAAGCGAGTTATTGAAATAACGGTCATCATTAATACCCATTGAAAGAAT
GGGGAATAGAGCCGGTTTCAGTTCGCGACACACGAGATGTGTGGTACTTTCTCCGCCATGGTGTGTTTCATACACTTCATAACCATCAATAACATT
GTCAACAACTTCAGCAATGACGTCG

>c28122_g1_i1_len=369
CAGAGTGTCATTTATCACGCACGTGATGTATGCACTCGTTGCTCCGATTTTTGCAGTATATCATTTGCTATGGATGTTGCGGGTAACTTCGAGTTA
CCAAGAATATATGGACATGCTAAAGAATGAGAGCGGTGCGGCAAAACAAAGTCAAACAGTATTTCGCAATGATCTTGCTGTGCTATATGAGCTTCA
AGTTCTGTTCAACAGAGTAAGTGCGGAAGTTGACTGGGAGAAAGAAAGGAATAACCGAGTTAAATCGAATACAGTCCCTATTCCTGCAGAAGTGGT
TTACCAAAACGCAACCTGGATTTTCAAATCAGGGTTGAGTGAAGGTAGAAAGCCATTGAACCTGAAGTGGGATGATTACTG

>c31620_g1_i1_len=321
CTTACTGTGGTTAATGCAATGTATGCTCCCTTGCCTCGTAGTGCTGAAGCACATGGCTGGATTTCTCCTATTGGTCAAATCTTTATGCCTAAAGTT
TCAAGTATTCGTGGTTTTATGGGTCAGCTGACACAGGGCTCGCCCTACGAACCTCATCCTGACAGGGCGTTGACTTGGGCTGGTTATGCTAACCAA
CCCGACCGAATGTTGATCCATGCTATTGCTGCATGCGAGGCTTTCTACACCGGCTTGTACGAAATCCTTACTAGTAATCCTTATGGTGTTGAACAC
TCTTTGTACCAACTAGGTATTACTAGCTTCGAG

>c32294_g1_i1_len=309
CGTAATTAACGCCATGTTTGGTATTGGCGGGCAGGTACAAAGTATTAACGAGTGACTGAGAGCCAAAAGCAATACGCAAAGGGTAAGAACATTCAA
TTTCGCCGATAAGAGTACAAAAGATGTTGAATTCACGGTTGGTAAAATTTTCAAGGTTAGGACAGTAAAAGTCAGGGTGGATAGATTCGTTTTCGT
CATATCGCACAATGATGAAAGATTGGTTGAAGTTAAGGCCTTTCCAAGGTTCGAAACTACGCTTAAGGAAATTTCCATAACTGATGTTGAGATGTC
CATCGTCATAGAAGAGATCAG

>c33879_g1_i1_len=403
AAAGTTCTCAGCTGTTTGGCCACTATCAGACCCATGGACCTAGCTTTGGCTACTGGCGCTTCAAAGCCTGATCCTTTCCATGCACCGTACAAACCT
CTATAAATGGCGATACGCACATCAGTTTCGAATCCGGTCTCGTACCTGATGAGGTCACGCTCAAGAGACGTGTAAGCCTTCAACAACAACTGCTTA
CGATCCAGTTCCCCTTCTCGAAGTTTAAGCTTCTCCTTTACTGCAGTGATATAGGAGTTAATGCCAGACTCGAGGAAAGAATATTTCTGGAGGTAA
TAGCTATCATCACGATGTACACGTGTTAAACGTTTGATGTTCTTCTCGCGAATTGGTGCATCTTTACAGATCCCTCCTTGCACAGGATGCGTTGTG
AGAATGCACTCAACAATTT

>c42138_g1_i1_len=440
AGCGTTGAAATTAACGCTCATGCATTCAAGACTATCATGAGTCCATTCCGCTGAAAGTAACTGCTCCTTGCTCAAATATGGCATGTAGCAATCAAT
CCAGGCCCTCATAACTGCCTGCATGCTTGCAATTGAATGTTGACTATTAAAGTCGTCATAATCGAAGCAAACAGGCACTGTGTCTTTAAATTTGTG
AACACTTGCCATCACGATTCGGTCAGTAGCGAATTTGCCAACTGGGAAGCAAGAAGGTAAAGTGTCTTCACAGTTGCTCATTGCAAAGTCAGCATG
CAAGAAAGAAGTGACGTCACAACCATACAAGGCACGTACTTTACCCCATTCATACTTTGTGCTAGTCGTAGCAAAAATTTCAGGGTGGCGTGAGTG
CCAGTAAGAGTGATCTCTATCTCGCTTGCATGCAAACCAACATGATTTAAGCTTTG

>c45664_g1_i1_len=401
GGAATTGTGTAATTTTTGTTTTAGGTAGAGAAGATGCGAAATCGAACAGCAGTTGAGGATCAACAATTGGCCTATATTCATCAACTTGTGGTACAA
AGTTGTAGAAATCTTGATGGTTTTCGAAAGTGATACCGGAACGTAGGTTTCCAGAAATCATACCTCCGGCGATTGGCATAGTTGACGGTGTGTAGT
TAGAAATCGACTTAGTATTTACATACCAACACACAATGTTGCGTCTCTTAATGCGCAAGCCCCATACTTTTTCATCTGTGATGGGTAGTACAAAAG

GATAATCAACGTTGTGTGTTGGACAATCATCATTAGAAACAAACATCAAAATATATTTACAGTTTTCAGGCAACGAGTTATCTAGGTTCTCATCTA
GGTACATAGGAACTGGG

>c50237_g1_i1_len=391
GTTCTACTTGACCAGCTCACCGGTCCTATGGCCAACATTCAAGCCAATTTCAAAACGCTTCATAAGAGCGTTAAATATGGAATTAAGGCAGATGTT
GACCTTAGTCTTACTGGTGAGTATACTCATCAATTCAACCGGCGTGGTGCCCTCTTCTCCAACATGCTGAGTCCTTTTGGAATCCTTAAGGCTCAC
GTGGTGGAAGATGTGCCCAACTTTTCAGGAACCAATGTGAGGTTTATCAATGACGACGGTCGTATCGACGAAAAGTGTATCTTTGATGCTCTTAAG
CTTAACAGCTACTTTCGTACGAACACGATCGCCGCAATCTCTCCTCTCATTGCCACCCTTGGTAGTGAGAACCACGTATCGGTTCTCATCAACATG
CTCCGCT

>c25217_g1_i1_len=311
ACCGGATCGCTGCATAATTAATGGTCCGTTTGATGCTGGATTGTTGTAAGATAATGCATTCGTTAAAGTTGACAGACAATAGATTAGCCAGATTCG
GTCTGTAGTTCGGCATGGTAGTTGTGCTGTAGTGGTTCGTGGGAATCCTAGGATGAAATTGCGATCGGTTGTGAACGTGGTCCGGATTGTTCTTCG
TTAGCACTGTCATTAGCTTGTTTATAAGACTGTCTACTTTAGTGTCGATGTCGCTCATTGAAGATGCAATAATCACCAGCTTGTGCAATCTCAGGG
ATGGTTCGGTTTCGAGCTCCCGG

>c18669_g1_i1_len=952
ATAGTAAGTGAACATCCCAAATTGCAGTGGTGCAGCACTGACTTAATTGAGAACTCTGCACAGTAAGGGATGTCCATTTATGTAGCAACAGCAGTT
ATGAACTCAACCTTTGACATTATCTCAAAATTTGGATCCACCTTCTTCTTGTAGATCTCAAGAACATTTGCGATCTTCTGGTAGTCATTTGATAGA
GGCAAAGTCTTGAAAAGAGCAACTCTAACATCTTCACTAGGAACAGGAGAATTGTGCCCCACCATGGTGTAATTACCTTGATCATCAGCCAGTGCT
TTTAAGTCAGGGTCTTTCAATTCAGAGATCTGGATGGACTGATCTAGAGAATAGCAAAGGCTTGCCATCAATAGTGTTCTTTTGAGATTCATGTTG
ATATTCTTGGGGATCGTAGCAGGGAACACTTGAACTTGGAGATAGTATGGCAGTTGAGTTGATAGCATAGGTCCACCAGGAAACTCCTTTGGTCCA
AGAATATTTGCTGTCTTCACTGCAAGATCAGGGAACGTTGCCATCACTCTAGGGAAAGTGATCACCTCTGACTTCTGACCTTTACCACCTCCCATC
TTGATTCCATACTTGCCACAAAGATTTTCAATGTCTTGCTTACCTTGATCAGACATCTTGTTGATGTTCTTCTCAGTCACACTACCCTTGATTAAA
CCAATAGCAACCATTTTCACAACATCTGCGATGAAATCAGCATCTGAGATGCTCTTATTCATTTTCACTTTGTACAAAGCAAGAGCTAGAGCAATC
GGATCAAATCCTTGGTATTTGAAGAGATCAAAGTCCTCAGACTTGAGAGCAGAAATATCATGGTTACTGATGAGAGTGAGGACTCTTTCAACGTCA
GCGCGGGAAGCAGTCATTTTGAAATAGAAATTGTTTTTTTATTTTGAAATGTAAGTTTTTTTGAGGCAATTTTTGTTGTATTTAAAGG

>c9415_g1_i1_len=2203
TTAACACTCTCTTTCAATAAACATACAAATGGCTGAACCAAATGACGTGGAAACATTGTGCAACTCAGGGGTGTTGTACAGCCCGGAGTTCATCAA
AACGTTTGGATCATCTGGACCGCACTGGGGAAGCGATACGTGGCTCCAGCGTGAAGCTAGTTTGAGACATGACATGGTGTGCCTGCTATTGTGCAA
TCTGGAAGTCGATGAAAGCCTCAGGCCTCTCGATCTCGAAGAAGATTTGAACCCCCCACCGTTGAAAAGGAGAAGATGTGATGAAAGTGAAGCGAG
TACCTCCAGTTCAAGCGCATCGGACAGAGGGGTCAGTTCCCATGAGCTGCCCAGAGACCCAGATGTGGTTGCAGAAGTAAAAGGTGATGAGGACGT
GTATCGTTATCTGCTTCTGGAGGGAAGGTCTAATGTAGCTCAGCTCCAACATAAAATATGCACTGCTTTAGGCATAGAACCCATGAGGCGTCAGTA
CGATATATATGATAAGGTCGAAAGAAAATTCATTGAGGTTAAAGTCACTCTAAACTACCAGGCTTCCCTAGATGAGTTTGACTATTACATGGATCC
AGATCAGTATATTGCACTGGTCCACCTTCATCCGGTGTCAGCAGATTGCAGAACCAAAGGGAAACGCGATCCAATGCCTGGGTTGGGAAAGGCAAA
GCAGTTTCTGTTGGAAAGAGCATCGAAGATGGGTGCCCAATTCAATCACATGGAGTCTGACCTCGTCACATCGAAAGACTTGGTTAAGTCGATCTT
TGTCTGTGAGAGATTCAACAAGTGCGTGGCAGATTGGACAAAACTGTTCTGGGATCACAAGGACAATGCAGCTGTGGGTTATGAGAAGATAATGAA
CGCAGAGAAAGTGGACTGGGTTCTAGAGAAGGATTTGAAAAGCTTCATAGAGGATACAAGCAAGAGGGACGCAGAATTCATGAAATTCCCTGGAAA
AGTCCTCCCAGATCCATTGGTTCACCATGTCATGACACAGCTTGATAAGGATTCAGAAATGATCAGCTTGTTGTTTGAGGATCTACAGTTTGCTGC
AACCGAGGAAAAGTCCCATGTGTTGAACTGGATAGTGAACAAGTGGCAATCATCTGAAGGAAATACATTTTGCCTCTCCACAATCAAAGATAGGAC
TGGGATGCCTATAGAGCTGCAAAGAGACTTAGGCATAGGGTGCAAAGGGACCGTGCGCGATGATGGCAATGTTAATCTAAAACAGAAGGAGTTTAA
ACCTATCCAACCAGCGCGCTATTCGGCTTGGATGTCAAACCTGTTAAAGGAGTTGTCAGAACCCAACAAACTGGGAATGAATTTCTTCTTAGAGTT
GCAGCAGCAAGAAATGCAAGATGACCATCCAATGAACGTGATAATTGAACCCATCGCCAACAAGTATTTCAATCTGTTTGGCCACTCAACTATTGG
GTCGTACTGCAGCAAATTGAAGAGCGTGTACTCCCGCTTTAGTGGGGCATATTCCGGAAAATTTACCTCCTCTAAAGACCGTCCAGACGCTGTGGT
CTTCCCAATATACAGTGTAGGTGAGAAGGATGGGGAAACTTGGAGAGCGTTATCTGGTTTTATCATTCGGGGTCCCCAACACGCAAGAAGTGCCAC
TGACAAGATCCCCATTATCACAGTGGAAATGTTGAACGGAGATGATGGCGTGAGGTATAGGAAATTCATCAAGAATGCTCATGTGGTGAGAGACAC
CAGGGGAAGAGCATGGTGCTACCGCGTGAATTCTGTGATGAAGGGGGCCCTTTCATATGTGACCTTTGTAATCAACTCATCTTACCTCCCGGCGAA
CATGTTAGGGGAGTTGACCTTGAACAATCCGCACAATAGAACGGACTACAATGCAGGCCTGGCGATGGAAACCTACTTGTTAGAGCATGGCGAATG
GCTCATGGAGAGGTGCGCTGAAAGTGTCATGATGGCTGCGATAGGGGGCTCTCAAGAGGAGGGTGCTATGGCTATCATCAGGAAGATCTTCATGAT
GAAACTTGAGTGGAGCAGAGGGCAAAAAGTGATTGGATGTGATGTCAAAGGTTTAGCTGATGCTCTGAATGAATGTCTGATAAATTCCCCGTTTGC
TCTGTACATGGGTAAGCAGCTCAGAGACTCATTGGTTGGATAGTTCGGATCTCCTTGGGGTTGCCCTATTTTTGGTTTAAGAAAAAGAGAG

>c16223_g1_i1_len=254
GGGGAGTTTGCCACGAGCATTGGGTAGCATCTGCTCGGGCCAGTGGTCAAACAGCATTAAAGCGAAGTTCGTCGACCCTGCATCTAAACTCAGCTC
TATCGTAGAGATGGCGCGCAAGTTAGCCAGGCGCGCCGACTGCAACGCAACCTTCTTAGACAAGATCTGCCGAGTCGCCTTCGAGAAGTGGGCTAC
TTACGGGGAAAAGAGGCTCGTTGCTGGCTACGTCCATGGCAGGCGCATTGACGGGGGCCTCG

>c26707_g1_i1_len=500
GGGCGCTGGCGCAGCACAGGTCATAGGCGGCTGGGCGGGACCTGCAGACGTCAAGATTCTGGCGCGGAAGAACGTACCCCTGGTGCCGAGGCCTAA
ACGGCGGACAGGAGTGGAAGCTTTCGTTACGCCCTACGAACGCCACCGCGACAAAGTCAGGCAGATGGAACGCGCTGGGACAAAGTATGACGCCTT
GAGTAGTGCGGTCAAGAAGAGTGGACGCAAACGACTAGCTGAGCGAGTGGCACTGGACTGTGGAGTGGAGGCCAGTAGATTGTATCACTGGAAAGA
GGAACTTACGCTGTATGGGTGCGGGACGTACCTGTTGACGGAGGATTACTACTCGCTCGCGCAGGTACTAGCTGTCGTAACCGCGGGGGAGCAGAC
CGACGATGATGTGAGCCGACACCTGGCCGTTTACGCTATGGCACTGGATAGCGCGGGAGTGCTGAGTTACTAAGACCGTGGTTATCACTCAACGTC
GGCCAATATCAGATTATGCT

>c35080_g1_i1_len=257
CTTGTCGAAGAAGAAACCCTCTTCGCGCTTGTCTATCATTAAATTCCATTCCTCGTCACACCCGAGTGGCTCGAACCCGACGACCATCTCGTCAAT
GTCGTAGCCGCTGAATCGTTCCGCCAGACTAGTTTTTCCACCTAACATAGGGACAACTACGGCGTGCAAGTCCCACCTCGCCTTAATCTTGTCCTT
CTCGTACCGCAGTAGAGTACGTTTGCCTGACTCCGGTCCAGTGTAGGACGCAGCTAAAGTTGGAG
>c1129_g1_i1_len=292
CTCCATGCTCTCGGGTCGTACAATATAGGGTATCTCATACCCCCAGATGTCTCGCCAACCTTCCGTCAGAGGTAGGTTGGGCCTGTGGTTAAAGTT
GCTGCACCAAGCTAGGCAGACAAAGGTGGACATGTGGATAGAGAGGCCGGTCATTGCCTCGATGATGTTGATTGCCATGGTGCTAATGGCATTTAG
ATTTGTCCCGGTCTGGCTGTCAACAGCCCGTCCAGGAATGATGTCACCAACTGGACCTCCCGTCGCGCCCATGAGTTCAGGTTCCGCCCTATGAT
GTTG

>c2748_g1_i1_len=373

GCTCCTAAGTGAGGAGGCGTTGTCATATGACAACGTCACCCTTTTCGCATCGGCCTACATCCTCCTGGACTATTTAGCCTACTTGGAGAGGAACGA
CATTGGGTGGGCTCCTGCAGCCGTACCCGCTGATCGCGTCACCCTGTGCAACATAGGGTGTGCTGACGCACAAGTGGATGCCGAGTACGTGACCCT
CGTCGAAGCCATCAACGCCGGCCGAGTATGCCTTCATCGGTCTGAGCTGACCACTGCTGACGTGAACTGCATGATGATGATGGCACAGGGGACTGG
GTACGTTCAACTAGCTGCCCGCCGCATGATGCCCATCCATGCATACATAGATCTGCCCCGCATACGCTGGTGTATCTGGGACACG

>c7017_g1_i1_len=238
GTGGCTCGACCGCTTGAAACTACCGCCGGTCACGCGCACCCACGCCGAATACGAGATGATGAAAGGGGGTGAGACGGCTAATCCGCTGTTCGAGGT
GTGGCTAACCAGACTTAGCTCTGGAGACATGGATAAGGTGACACCCGTGGTTAAGTCGAGGATGGGCGTCGTTATGGCCGCAATCGGGCTCAGTCC
ATTCCGAAGCCTGAGCAACACGATGACGGCAACAGGGTTGGACGCG

>c8022_g1_i1_len=265
GCTCATCCATTCGTCGAACATCTCTTTAGGCATAGTCTTGACGTCGTGCAGATGTTGCACTTCCTTGAGTGTGGTGAGTAGGCACAGATGTGGTTT
AGTGAGCTTCTTGTGGACGATGGGGGATGTTGGGTCTCCAGGGTCTATACGTACGTACATCTTGGGTGTTCTGTCTTGAGATTCGGAAATGGCTAT
ACATGTCTTACACTGGAGCAGCCCCCGTTCTAGGTTGAAATGGTTTTGATGGGATGGGTGGATGGACACGTTG

>c8271_g1_i1_len=892
GATGTTCGACGAATGGATGAGCATTAACGGTGGCAATTACCAACTTGGCGTTAGCGCCCAATGTATGCGTCTCTGGAAGACGGTCACACAGTACTC
ATGTAAGCGGACGCCTCCAGGCCATGTGGACCAAGTCATGGCCAAATACAACCAACATGTCGTGCGAATCAAACAATGCAAACGCCACCGAGGTAA
ATTCGTGATGATCAATTGGAGCACACTGCGCAATAAGCGGGACCTTGCCATACTCAACGAACTGTGGCCATGGTCCAGTGAAGCCGACCACACCAA
GACGAATGTATCTGCTGCCGACCTGGTTTGGCACTTAAGCCGCCACCCTACCTACCGTGAGAAGTACGACCGTTGGAATCACCTATTCTCCGCCGG
CCTCTACAACATAACGATCTGCGCACTGCTGGTGCATATGATCGGTAACGAGCAGGCCGACAAGATGTTCAGTCTCCTAGAGAGGTACAAATTTTG
TTGCCTCGACATGGATGACTACGCGGCCCTGTTTAAACACATCAGCACCGCCATTCGGCGGACGGGGCGCTGGGAGGACGGCAGTCCAGCCAGTCT
GGAAGAGCGGACAGGGTGTGCTGGCTGGGAGCTCGCTATTGGGCGGTCAGGTAACAAATCGGATTGGGCGGATGAAAAGCGGAAACGGACGCAGAT
GGTGGTCCCCTTAGGAGCTCCCACTGCGCAGGATAAAAGCGCGACAACTAATGCCGAGTACATAGTGGCCCTCCGGAGAGAACTGGACCTGATTAC
GAAACAATTAGTGCGACCACCGGGTCGCAAGGAGAGTTGGGTGGACTTTGTGGAGAACCGACAAAGGTGGGTGGCCTCCGGTAGTACAGGAGGGGA
GCGTCTAGTCATGCCCGATGGGGAGCGG

>c8605_g1_i1_len=410
CCGCCCTATGATGTTGAAATGGTTCAAGCAGGTCGAGTACCCTAAGGAGACTACGCCTGCGACTGCCGCTCCCAGACGTACGTGTTCGTCGGCAGT
TAGCTGTTGGAGCGTATCCCACTCCCTGGCGAACAATCTTTCGTCCACACTGGTTACCCAGAGGCTAGTTAGCAGTGTCCAGACGACGTTGCGGCC
GTGGACTCGCGGCAGGCGCGTCCGCTCTGTCTCGATCGTGCTCATGACCATGATAGCCGGGTCTCGGTCTGGGTATGCTACTGACACGTTCACCAG
GGTCTGGCCTATGGTGCAACCCCTCACCGCAGCGTCCATAGTCCCCACAAGCCGTACCAGCTTGATGATGAAAGCGCGCAATTGTGCGTAGGTGAC
AACCTCCGCCCCGACAGGCGGGATGG

>c9075_g1_i1_len=619
TGACTTCAACTACCAACACACTCTGGAAGCACAGGGCGCAGTGTTCGAAAGTCTAGCTGACGCTTTGGCACAACAGCAACACCACCCTGATAAGGT
GCGGGCTTCACGCTGGGTAGCGGCAGCACTGTTGGAACAATACTGCAAGTTCCCGCAGGACAAAGGCAAAATGACGAAGGTAACACAAGGAATGTT
CTCGGGCATACGGGCGACCAACTTCATCAACACGCTCTTGAATCTAGGGTATTTCAAAGTCGCTGAGACTTGGCTCCAACATGAACTGCACCTGCA
ATCTGTGGAATTGCATAACATACACCAAGGAGATGACGTCTGGATATCGAACCAGTCTCGCCTATGGGCCATCGGCCTTTACAGGGTCTTGGAGAG
CTCCGGGCTCGTGTTCCAGCCCGCGAAACAACTGTTCGACGTGAACCTGGGCGAATTCTTACGCGTAGTGTACGTCTCCGGTGCCTGCAGGGGCTA
TCTGGCCCGTGCAATAGGCACGCTGATAATGAAGCCTATCCAGAACACGGATGTCGTTAGCCCAGCTGAGCGGGCCATCGCCCTCAATAGCCAGAT
CAACCACCTAGTCAGAAGAGGTTTCACCAGCGCAGGCGCTGAT

>c9470_g1_i1_len=6772
CCAGTCACTGACCCCCGACAAACTAAGAAAGGGGCCCGATATAGAGGGGGATGACGTAGAAGTCCTGTACCTACGTACAGACCGATTAGATATCAG
CCATGAGCAGATACAACCCTCAGACGAACCAACAGACACACACGAACGTGTGTTGAGACCCGAAGAAGTCGAACTAGACGCAGCTACCCAGGAGAG
GATAGCCATCTTGACGTCCGAAGAGGAGGCGGGTGCAGTTGGGGGTGAATTACTACCGCCTATCCCTGAAGAATCGCTGATAGCCAACACCACTAG
TCCACCCTTGTGTGGAGAGGAAGACTCTTTCCGGTCGACCGTATCCACTGAAGTGGCGCTAGTAACACAAGAACTGGTCGGCGCAAGCAAACCTGT
GGCTACATCCACGCCGTTAATCACGCCAACAACGTCACAGCCAGCTGTGGGACATGGAGTCCCACTGATCTCATCCACGCCGTCCATCCCCACCAG
AGGGCGGGGACGGGGTTTGCCCCGCTCCGCCTCAGACCCTGTCAACACCACGCAGCCCGTAGAAAGCATCGACGAAGAACTGCCTTATGCAAGTGC
CAGTAACCGTCCACCCACCCCTCCTGCTGCCGATGTTGAAAACGCAGCCGATCCAGACCCACCGGCCAGCGTCCGTAGGACGGTGGCCCGGCGTGG
TGTGCAGCCGATAGCAGCAGTAGATCCGGCACCTCAGCCGATAAGGCGCGATGCGCCTACCGGCGCACTCAAACAGCTAGACGAACCGAACAAAGT
TGGGAAAGCTTCGGTCGAACTAGTCGATGTAGTTGATGTGCCACTCAAGATGCCATCTGAAAGAGAGCAGACTATGTTCTGCGGCCTGTACGGGAA
GATCGCGAGCCGCAATGAGCTAGTCCGCATCATTGACGATCAGGTAGTGCTCACTGGAGTACTATCGTTACCTGCGTTCCAGGGGCGTGCCCCCAC
AAGCACGTTTACCACACATTTCGACCGAGCCGTCGAACGCAAGCAAGCGCCACGTCGGTTCGTAATCGAAGAAGATAGAGCCATCATTAACGGCCA
CGTTGACTCGGCTTTCCAAGATCGGATGAGACCGGGAGGTCAGACAAGCGCGAAGGAGGTAGCCTCTATCCTGCGTGGCGAGCATCGATTGGAGAA
TTCGATGGCCAACAGGATAGTGCCGCTCCTAAGTGAGGAGGCGTTGTCGTACGATAACGTCACCCTATTCGCGTCTGCCTACATCCTCCTGGACTA
TCTAGCCTACCTCGAGAGAAATGACATTGGATGGGCGCCCGCAGCCGTACCCGCTGACCGCGTCACCCTATGCAACATCGGGTGTGCTGACGCACA
AGTGGACGCTGAGTATGTGACTCTCGTCGAAGCCATCAACGCTGGTCGAGTATGTCTACATCGGTCTGAGCTGACCACTGCCGACGTGAATTGTAT
GATGATGATAGCTCAAGGTACCGGGTATGTCCAATTGGCAGCCCGTCGTATGATGCCCATCCATACATACATAGATCTACCCCGTATACGCTGGTG
TATCTGGGACACGAACCCAATCCCGCCTGTCGGGGCAGAAGTCGTCACCTACGCACAACTACGAGCCTTCATCATCAAGCTCGTACGGCTAGTGGG
TACTATGGACGCCGCTGTGAGAGGCTGCACCATAGGCCAGACCTTGGTGAACGTGTCAGTGGCCTACCCAGATCGAGATCCGGCTATCATGGTTAT
GAGTACGATCGAGACTGAACGGACACGCTTACCGCGGGTCCACGGTCGCAACGTCGTTTGGACGTTACTAACCAGCCTCTGGGTAACCAATATGGA
CGAGAGACTGTTCGCCAGAGAGTGGGACACGCTCCAACAGCTAACTGCCGATGAACATGTGCGCCTAGGAGCAGCGGTTGCAGGCGTCGTGTCTTT
AGGATACTCGACTTGCTTCAACCATTTCAACATCATAGGGCGGAACCTGAATGCGTGGGCGCGACGGGAAGTCCAGTTGGTGACATCGTTCTTGGA
TGGATTACTAACTGCGAGACCAGGCCATACTCTGAACGCCATAAGCACTATGGCAATTAACATCATCGAGGCGATGACCGGCCTCTCCATCCACAT
GTCGACTTTTGTCTGCTTAGCCTGGTGCAGCAACTTCAACCACAGGCCAAATCTACCCTTAAATGAGGGTTGGCGAGACATCTGGGGGTACGAGAT
ACCCTACATTGTACGACCCGAGAGCATGGAGTGGGCCATTACAACATGGATCGCGGAATGGGGCCTGAACGGGCCCAACCCTAAGTTCAACTTGAG
CCATGAGACGATGGTTTATGGCAGTCGCGGGTCTAGAGGCATGTACATCTACTTCGGGAGTAAGAAGTATTACGACACACGCCAATCCAACTCTCC
GTATCTTTACGTGCTGTACGGTGGGCTAGCAGCTAACCTAATAACACAGATAGTCCGGGCACCCCGGGCTATATTAATGACCTTTAAGTTGCTGTC
TCGCAGTACGGCCCGTGAGGTGATGGAGGAGCCGGGTTGGCGTCGGGATGCGGTGTGGCAACCGACATACAACCCTGTCACCTTCCACATTACTCC
GGGCACTCTCATCACCTACGACTGGGAGCAGGACGTCGTTATGGCACCTGTTTACCTACTAGCTGACATTCCACGGCTCTTCTGGCCGCAGATGAC
CCGGATCGTCCCTACCAACGAGCCCAGTTATACCGGCTTCGTGTCAAGCAACGCATGCCCACAGGCGGACATGATCGTTGACAGCTGCGCCGTCTG
GAGCCTAATCACCGGTGGAACCGCTGATTTGGCTGCCGACCAGCATGCTGCCAATGACGCGCTCGCCAACGAGGAAAACTAGTTTGGGCCCTCGTG
ACCAACGTGTTAGAGGGCCGACTAGGTGAGCGGCAAACGAGGGAGCTTAACAACGTGTCCATCCACCCATCCCACCAAAATCATTTCAACCTAGAA
CGGGGACAACTCCAATGTAAAACCTGTTTAGCCATTTCTAGATCTCGAGATAAATCACCTAAGACGTACGTGCGCATAGACCCTGACGACCCGACG
TCCCCTATCGTCCACAAAATTCTCACCAAGCCACACTTATGTCTGCTCACAACACTCAAGGAGGTGCAGCACCTGCACGACGTCAAAACCATGCCC

AAGGAGATGTTCGACGAATGGATGAGCATCAACGGCGGCAACTACCAGCTTGGTGTCAGCGCCCAATGTATGCTTCTCTGGAAAACGGTCACACAA
TACCCATGTAAACGGACACCCCCGGGCCATGTGGACCAAGTTATGACCAAGTACAACCAACATGTCGTGCAGATCAAGCAGTGCAAACGCCACCGA
GGCAAATTCGTGATGATCAACTGGAGCATACTGCGCAATAAACGGGACCTAGCTATACTCAATGAGCTGTGGCCGTGGTCCAGTGAAGCCGACCAC
ACAAAAACGAACGTATCCGCTGCCGACCTAGTTTGGCACTTGAGCCGCCACCCGACGTTCCGCGAGAAGTACGATCGCTGGAAGCACCTCTTCTCC
ACCGGACTCTACAACATAACGATTTGTGCACTACTAGTGCATATGATCGGCAACGAGCAGGCTGACAAGATGTTCAACCTCTTAGAGAAGTACAGG
TTCTGTTGCCTCGACGTGGATGATTACGCAGCCTTGTTCAAACACATTAGCACTGCCATTCGACGGACGGGACGCTGGGAAGACGGCAGTCCAGCC
AGCCTGGAAGAGCGGACGGGGTGTGCCGGTTGGGAACTCGCCATCGGACGATCAGGCAATAAGTCGGACTGGGCGGAGGAGAAGCGGAAGCGGACC
CAGATGGTAGTCCCCTTGGGGGCCCCAACTGCGCAGGACAAGAGTGCGACGACTAACGCCGAATATATAGTCGCCTTACGGAGGGAATTGAACCTG
ATCACGAAGCAGCTGGTGCGACCGCCGGGTCGTAAAGAGAGCTGGGAGGACTTCGTCGAGAACCGGCAAAGATGGGTAGCTTCTGGTAGCACGGGA
GGGGAGCGCCTAGTCATGCCCGATGGGGAGCGGATACGCATGAACAAGCAGGCCTATTTCGACTCAGTTTCGAAAGAAGAAATGGTATCATGGCTT
GAGGATGAGCCCAAGATAATCGGCACGGCTAGCGAGAAGTTTGAGATGGGCAAATCACGAGCGATCTACGGCACCGGTGTCCGTGATTACGCCATA
GTCGCCTATGTGATGGATGGCGTCGAGGAGCATCTGTATAACATCGATGGAGTGGAAAGTGGGCTGACGGGATTTGACGCTATCGCAACCATGGTG
AGACGTTTGCAGGTAGTGAGTGAACCAAGAGTCGAGTGTACGATGGTTGATTATGCTGACTTCAACTACCAACACACTCTAGAAGCACAGGGAGCA
GTTTTTGAGAGTCTAGCCGACGCCTTGGCACAGCAGCAACATCACCCAGACAAGGTACGGGCTTCGCGTTGGGTAGCTGCAGCCCTATTGGAACAG
TATTGCAAGTTCCCACAAGATAAGGGCAAAATGACTAAAGTGACACAAGGGATGTTTTCTGGCATACGAGCGACCAATTTCATCAACACCCTTTTG
AATCTAGGGTATTTTAAAGTAGCTGAGGCCTGGCTCCAAAAAGAGCTGCGTTTACAAGCCGTGGGTTTGCACAATATACACCAAGGTGATGACGTT
TGGATATCCAACCAGTCCCGCCTGTGGGCTATCGGTCTCTATAGGGTCTTGGAAAGCTCTGGGCTCGTATTCCAACCCGCAAAACAGCTGTTCGAT
GTGAACTTAGGCGAGTTCCTGCGCGTAGTATACGTCTCCGGTGCCTGTAGGGGCTACCTAGCTCGTGCAATAGGCACATTGATTATGAAACCTATC
CAGAACACGGACGTGGTTAGCCCAGCCGAGCGAGCCATCGCCCTCAATAGCCAAATCAACCATCTTGTCAGACGTGGTTTCACCAGCGCAGGCGCT
GATCTGATCTGGCACGCCATCGTGCCCTACGCAGCCCGAGCTAAGTTAGTGCACGGAGCCATAACCATTCCGGTGGCATACCTCATGCTACCCTTC
CTGGATCACGGGCTGGATCTAGGACCGCCTATGACGGCCGCAAAACGCGCAACACCAGTGACCCCCATCCCCACGATGGCTCTGTGCAGTCGAGCC
TTGGAGGAGTGTGTACCGAGCCGTATGGCCCACGACTACGTGGGTGTGATAAGCAGGGGCCTAAGACGGTCTCTACGAAGTGAAGAGCTAGAAGCA
ACGTTGCATGGCACTAACGTAATCGGGTCAATGCGACAGATAGATCGCACTCAGTGTTTAAGGGCTCACGAGCGTGATCTCAGGAAGTGGCTCGAC
CGCTTGAAACTACCACCAGTCACGCGCACCCACGCTGAATATGAGACGATAAAGGAGGGAGAGACGGCTAACCCCTTGTTTGAAGTGTGGCTCACC
AGGCTTAGCTCTGGTGACATGGAGAAGGTGACACCCGTGGTCAAGTCAAGGATGGGCGTCGTCATGGCCGCAATCGGGCTTAGCCCGTTCCGAAGC
CTGAGCAATACAATGACGGCAACAGGGTTGGACGCGCTGTCAGCTGCAGAGGTAGCGATTACATCGTGCCCGAAGATTCAGCTGAGCGGCGCGGCC
GCTGCCCTGTTGGCCGCATTGAAGCATAAGGTGGGTGAATGCGGGGCCCGATTGGTGCTAGACGGCGTCAGAACTGGCTCCACTAAGTACGAAGGA
GAATTCCATCCCACGATTCTCTCTTGGGTTCAAGATCAGGCGTTAGACTGTGTCATTGGTCAGATGACCGCGAAGGAACGTTGGAGGGACGCCGAT
ATCAGAGAAGTTGTTGTCCAAGAGATGGACAAATACGTCAGGTCGTTGAGGAAGTTCCCGATTTTTCATATGATCAGCCACTATTAGAGTTGAGGG
ACACGTTGTGGACGGCGTAACCCCACCGCGGGTACATATGCTGCGCCCTAGAGCAAGGCTCGGGCGTAGCAGTTATCCTAGGGCACCCGCTTGAAT
TCGAATAAGGGTGTCCTAAACGGCGACGATACCGCCAAGCCTGTATATAGTCTTTTGTACATAAATCTTCCACTCTTTCCTCCTCTTTCAATTTTA
ACTCTTTCATTTTGACCAGGCCCACGGGTGGGGCCGAATGCTGCGTCCTAGAAGCCACGCTCGGACGTAGCAGCTTAAGCTAGGCACTCGCCTGAA
TTTTATAAGAGTACCGGCAGCCGCGGAAGAATCGCGTTTTATTTTCTGTACATACTTTTCTTTCACCCCTTTTCCTACTTGCTCCCTCTGAATTCA
GCAAGTACCAAGACTCACCAACACTAATACACACGATACTTCTTCTTATCACACACGCAAGCAATACATACACTACACACACACACACTACACTAA
CACACATTACACAACACAAAAAAACAAAAAACAAAAATAAAAAATAAAAAATACAAAAATACAAAAAAATATACAAAAAACAAAAAATCCAAAAAT
AAAAGGGGTCCTTCGGGGCCCCGCCAAAAAAAACATTTTAAATGTTAAGTCATACATGCGACTAGAGCGCCACAAGCACACGGCAACTTCATAAAC
CGTGCGAAACCTTGTGAACCCGTCTAGTGGCACTACCACTGCCCAAGAAGGCGTGCTGGTAGGATGAGACCTAATGTAACCAATATGCAGAGTCTC
GTCAGTGTGTGGCTAGAATTACGCAAGCGCCAGACACCGACCCCGGTGACTG

>c37343_g1_i1_len=253
TTTCACCAGCGCAGGCGCTGATCTAATTTGGCACGCCGTCGTGCCTTACGCAGCTCGAGCTAAGTTGGTACATGGAGCCCTAACCATCCCGGTAGC
GTACCTTATGCTACCCTTTCTGGACCATGGACTGGACTTAGGACCGCCTATGACGGCTGCAAAGCGCGCAGCACCAGTCACTCCCATTCCGACGAT
GGATCTGTGTAGCCGAGCCTTAGAGGAGTGCGTGCCCAGCCGTATGGCCCACGACTACGTG

>c48383_g1_i1_len=229
ACGACCCGAGAGCATGGAGTGGGCTATTACGACCTGGATCGCAGAGTGGGGCCTGAATGGGCCCAACCCCAAGTTCAACTTGAGCCACGAGACGAT
GGTGCACGGCAGCCGCGGATCCCGGGGCATGTACATCTACTTCGGAAGCAAGAAGTACTACGACACGCGCCAATCCAATTCTCCGTACCTTTACGT
GTTGTACGGCGGACTAGCAGCTAACCTAATAACACAG

>c8342_g1_i1_len=1509
GGCGTGGGTTCAGTACCCACATTAAGGCTTCGCCACCGATAGTTACGGACTACTCTTCATATGCACAATAGCCGGTGCACCAGAACCTCAGGGAGA
GGTAGAATGGGTGAGCAATCAGTAATCCTTATGCCGAGATTCCGTAACATGCGAGCTGTGTCTCGAGGGAGATTTATCGTAAACGCACGAACTCCT
ACAATGTTACGGCATATCTGGGAGAACTGCCCATTCACTCCCGCGTTCGCATAGAACAACCCTAAGGCCCGACTGGCGAACATGTCCCAATCCTCG
TCTGGCCATTCTGGATGGTACAGAGCCTCCATCCACCTTTCGAGGCTACGACAGGGAAAACCACCTTCTATCCTAAATCCGAGAAACTCCACCTCA
TCGACGCAGGTTGTGTAAATGGATTTATCAACGTTTACGGTCATGCCAGCTTCCGACAGTTGGCCACTTATCTCCTCCAGCGAGAAGTGGCTGTTG
CTGGACACTACCGAATCATCGCCAAAGACCAGGCAATCTTCTGGCATCCTTCCGAATCTCTTGAGGAATGCATAGTTGATCAAAATCCAATTAACG
ATGGAATCGACAAGCTGGGTGAAGTAGGATCCACTTGGAACACCAGCTTTCTTCTTCCACCTTTCGCCGTTACACAACCGGAGCGGAGTCTCCAGA
AAATACTTCACCAGGTAGTCCCATGCTCGGTACAGTCTACTTGCGTCTGGTATACCATGCTTTTGGTATCGTCGTATGTCCACGTTCTGTTCGAGT
ATGGAAAATGCTATCTTTATCAACTGCGCCGATACTGTCTTATCGAAGCTGGAAAAGTCGATACATCCGAAATATCGATACTTCTTGATACGAGAG
CGGAGTTTGTAGGCTCCACCAACCGCCATATCGTATCCGTAAGCTAGCGGCGTCTTTCCTTTCTTGTACTCGTCGATCAAAGGGAGTGCAAAGCAT
GCCTCCATCAATGTGATAGCAGTCGGATATCCATACACTGCTCGGATCTTCGGTTCATCTCTCTGGATATGTGCGCGATACAACACCTTGCAATCA
TCAGGTGTCAGGTCTTCTCCTGCCTTAATGCGGTGCCAGTAGCGTCGGATCGATGTCCTAGCGTCTGGACAGTCCATAACTTCGCCGCGCGTTTTG
TACTGTGGCTGCCACGGGAGTCCGGGACTGGACTTGCGAATGTCCAAATCCATATCAAAGATATCGTTCAGGTGGATTGGGTTCACTGGAGTCACA
CTGAACTTATCCCAGGCTGCACGTACTACATATCCAAATTCGTCATCACGAATAATTGTCGGATTGGATCCCATCTTCAACATGTCATTCTTGATT
AAACTAAACCTCAATTCCGATCTACGAGCAGTAGAGAACTTCTCTCCAAACAGAGCTTGTGCATATGGGGAGGGTCGGGGTACGTTTGGGCGCATT
CCAAAACCTCTAGAACGATTAACGTAATACATCTTGCCCGTATTGAATAACACGAGTAAAATCGCGCGG

>c9239_g1_i1_len=4038
CTTCGATAAAGTCACACCAATGTTATGTCAGAATATTATCGTCCTAAGCTTTATGCCGTAAGATCCGATAGTAGAGCAACAGCAGCGTTAGCCACA
TGTATATCACTTTTTAGCTGCCGAACAGTAACGTTAAAATTCGGAAGAGCACAGCTATAAAGAAAACCTTCATTGCTTTTCGCATTAATCAGTTTC
GAAGGCGCACGAGAATTAACTAAATCAACCAAAGTACGATTTCTTTCAGCCGATTCAACTATCCTCAGCGTTCCCGTTCTTAGGAAATCAAGTAAC
ATAATCTGCCGCGAGAATATGAAACATTGAAATCTCATTCCACTATTAATTATTCCTGCAGTCATAAAATCATTAAACGATGATTGAGTAATCATT
CCAGTCATGGATATTAAATCATCGCGAAGGGCTCCCATTGAATCATCTTGGATTTTCACAGCTGCATTGGAGAATCTGTGATTCAATATCTCTTCG
CACACACTGTTCTCAAAGCCCATACGCCATAGGATAACACGTCCTTTATCGGCGTCAAGCATCGTATTCACATCGTATTTATCTAAAACACTTGTC
ACTTCTGCTGGAGAACAATTTGATCTCCTAGCAGGATCTTTTGAAATTATGATACGTAATCTCTCAGTCTGAGTGAATCGCGTACTCATTTCATTA
TTAATGCCAAACGCTCTAATAATAAGAGTTGTCTTGGCAGACATTCCATTAATAAACGTTAAACGATTATTGAGTGGAATGGGATTTCCATATGTA
TATACGAATCCCAATAACCACTCATCATGGTTAAATTTCAGATCGGCCCGAGTCTGTTTTAAGTTCTTTATTATATGAAGAAGCAACATTTCTCGG

GCGCAATGTCGAACAGAGGTCATGAACGATTCTAGAGCTAAACCGTCCTTAAACATTTTTCTCGGAACGTTTTCCGCATTATGGGTACCGAGAGTA
AAGGCTCCTTTTAGAGCGTTTATCCTCTCATTAACCCTACGGCTATATAGAGTTTCACAATCAAGATTAGGAGACTCGGATATTCGAATATCCATA
TTCTCATCCGGATTGAATCCCTCTATAAGCTTAATCAGATAAGCATAGAGCACTAGAAATTTATTCTTCTCGATCTTCCCCATCTTGCTCAGTTCA
CGCAGGAATAACGCGTCAGTATGTACGACTGATAACGCTATCAAAGATAAACCGATCCCTCCGCAAGATCTAGGGAATAGGCATAGCATAGAAGAC
CAAGTAAATTCCCAGAAAGCATTTTTATCTTTTTTATCCTTCTTCTTCTTGTCAGAATTCTCTTTCTTATTCTTAAGAATATCAACGTTTCTTATA
CGCATCTCAAAACCTTGCGTTTCCCCAGATAATATCCTTTCGGGAGCACCGATTTTCAGATTCAAGAAGCGTGAATCGAGTCTGCTTATCCACCTT
GATATATATAAAAGAATCGCGATGGCGGCAGATTCCTCATATCCTCGAGAGACTTTTGTGCAAAGTAGTCTCCTCAAACTTTCCATAAATTGAACA
GAATCAGTAATAGATCTAGGGCGTTCACTAGCGATCAGCATAATGTGATCTTTCGGAATATAAACCCCAAGCGTAGCGTGAGTTTGAATATATTCA
CTCGAATGTATCGTGATATGCGTCTTAGGGACAGACATCTCCAGACCGCAATTTCTTACAATTGAACTAACTTCCATGAGAAAATCGTCAATGTCC
TCGGAAGTAAAGTCATCGGAGACGATCTTCATGGCGATCGTAACGTCATCGCCAATCGCACGTTTAGTAATAGGTTTGAGACATTCACGAAATTTT
AGATTCTTTTCATGCGTCCGCGCCAATATTATATCAATCATGGCTAATGATGCGGCAGAATTAAAAGCTAACGTCGTTAGTTCACCTGATGCTTCA
CTAGTCAAAAATATCAAGTCCTCCCCATCCATCCTGAAACTTACCTTAAATTTGGACTCGTCTTTAGGATGTATTGGATCACAGGTCATATAGTAT
GTACCCGCCTTCAGAGATTTTAGGCCGGGTATTCGTTTCAGCTTATATTCAATGTCGGAAATCACGATTTCCTCAAACTTATATCGAGATGATAAT
TCTTTCAGATCCGAGTCGTTATCTAGATACACGATTGGAACTCTACCCGCATCCCAATATGTACCATAAACAGATCCCTCTCCAAAAGCATAATCA
ATCATTTCATCTTTATCAATCTTGTCAGGACCAAAAACGGCCCCGTCCCAGCTCATGCGTCTTAGGGCACGGAGGATAGGCTTCCGAAAATTTCCC
CAGACTAAGCTCGAGTCAAAAGAGGTAGCGTCGATATCGATCGTTATGACCTTATCCGTACCAGAATTCAATATTGTATAACCAGTATCCACTACC
CGCACTCCACTTGTTGATGACGATCCAGCACTGATATGATCACAATGGGGGCCATCAGTTGCAAGCGGAGAACGGTTATCGGTGCTTGATACATAA
ACAGCGAGGTGATGTGCGACCGCTACTTGAGCATGAAGCGTAGGCAGCGGAATAACGTACACAGCTCTCGTAGCTTTATATGGTACATCCCGAGCT
CCAACGTTCCCAGGATTTTCACGAGTTAGTTTTCTCGACAAGGTGTCTCGGGTAAATAAATCTTTACCACAAACGACTCCCACTCCAAGTTTCTTT
CCAACCCGTACATTCTTGTATTCCCTATCATTAACAGATACGTCTACCTTGCTTGGTTTGACCCCAGCAGAGGTGCTTTTCCAGTAACTTATACAA
CGAGGATAGAATGTTTCAGTCGTCGTTGGCATAATTCCTTTCTCAATTGCCATTCTATGGAATGACATCAGTATCTCTTCAATTTTATATGGTAAA
TCTCCATAATCGCTTTTCGCCTCGAAACTTCTAGGTGCGGCTATTGAGAGCTGAGCTCTACCGTAACCAGCGACTTTTACGAGATTTAGAATGAAC
GATCCCACAAAAGCGAAATCGTCCACACTTGTATTCGACTCATCACCAAAATTACAGTGAAGATAGTCATTCGCAAGATCATGTAGTTTGTATAAC
TTATGATCCTTCATCGTTTTCAATCCATCTTCAATCGATCTTTTAGCAAACATCGAATCCGAGAATTTCTTATAGAAATCATGATCAATTTTCTGG
ATGACGAGATCAGAAACCAATCTGGATTCGAGTGGATTTCCGCTCATATGACGAACGTAATGCGGATTATCACTTAGCGAACCGGCTCGTCGCACA
ACCATTGTGCACGCGGTTCTAATATTTTTAACGACGATTTTTGGTGAGACCCCTATTCTCAAGGCCACTTCGTCAAGTATAGAATACAACGAATGA
TATTTGCCGTTAACCCGAATATTGTTTCGGGACAGGCTCATTCTAATCTCAGATATATTAAGCATACATTCGATAATGTTATTCATGTTGTAGAAA
CCTGACACGTATTTCAGTTCAGAAAAATCTCTTTTCCGAATGACTACAGGCGGAACGCCTTCCTGCTTTTTCACCCATGAAACAATACCAGAAATA
TATCGTAATTCAATCGGATAACCGCCAGTTCTCCCCTTCGACAAAGAAGAGAAGAAGAGGAACCACATATATCCCGGCATCGTTCCATATGTCAGC
GTTTCAGTTTTCGCACGTTCAAGTAGAGAGATATGAAAAGGAGATTCCTTATTTATTCTCCACGTATCTAAAAATGTCGAGCGAACATCAATCGGA
GTGTTCTTGCTCCGCTCGTCGCAATTGCGTATCAACGTATAATCGGCAGGTATCTTAAGTGATTCACATATTCTAAGCCATATGTTCTCATTCTCT
GCAACCTGTCTAGCATAAAGTATTGGTGTACCGCATGATATTACCTCCATCATTTGACGATAGAGGACGGATTCATGTTCTATCCAGAAATTTTCG
AGGGCGTCAAATGAAGAGAAGGTGTCGTTCTCTCCATCTTCCCCAAATTTTAAATTTCCGAAAAGACGCGCTGCTCTGGGACCCAGATAATCATGC
ATGATG

>c5575_g1_i1_len=446
GTGGCGGAATGTCAACGATAGACGATCCGAAAACGCGGCTTGTGTGGATTTACCCCGCAGAGATGTTGGTTGTGGAGGGCTTCTACGCTCCTCTTA
TGTATCGTGACTTTATGAACGATCCCAATTCACCAATGTTGAACGGGAAGAGCGCGCAGCGTTTGTACACCGAATGGTGCTGCGGCTTGAGGGAAG
GGGAGACACTATATGGCATTGATTTCAGTGGTTTCGACACGAAGATACCTGCTTGGTTGATTCGAGTAGCTTTTGACATTTTGAAACAGAACGTAA
ATTTCGAGACGTTTCAGGGTAAGGTTGTTGATCCCAAAACCCGTCAAAAGTGGGCGAATGTCTGGGACGCCATGGTGTGGTACTTTATTAACACTC
CCATTCTTATGCCGGACGGACGTATGTTCCGAAAACTCCGGGGTGTGCCTTCCGGATCCTGG

>c5783_g1_i1_len=733
CTTTGCACTGTTACTCGCCTCAATTTGTTTGGCGGACCAAACAAGGTTGTTCACCGTTGCCTCGTAATCTGTGAAGAGAAAACGAGTTCCTAAAGC
AGGCACGGAAAACTCACCGATCTGCGCTAAAAACGCAGACATTGAGGCCGGGACCCTGACATCCGTAGAGGCCACAGGTGCAAAATCGCCTTGAGG
CAGGCCTAAATTGACATGGCTGTGAACCACTTGCTGGGCTGCCCGCAGCAAGCAGGCGGCTCCGACGTCCTTAGCGAACTGGGCGTCATCAATCTC
AGCATGGGCTTTGAACTCAGCATATTTGGCGTTCATGGTAAGCCGGGAAGGGAACTGGGCTAACGAATTCGCGAATACACGCGGTTCGACCATGAA
CGTCTGATCTCTCGACGGCTCTCCGGCACCCGTCGTAAAAACGACTGGGAATTTACCCGGTTGAGGCTTCGGGACGGCTGAACCCGTGGAAAACAT
GGCGGCTTTTGCAGCTGAAGCGGACTGTCCAGGTTGTGCACCAACGGCAGAACGCGCGGCAGCTCGCTCACGTCTTCCAGGCTTGGACTTCTTGCC
AGTAGCGGAAGCGGAGTCGCTGGGGGCGACGGTTGAGGTAATTTGCGAGGGCGTGTCAGGCATGCTCGCAAGGTGAAGGAGGCGTAACGAAATAGT
CCTAGGTCACAAGTCTAGGCTAAAACATGAAAACTCCCCGTATACACAGGGCCAAAAGAGG

>c6217_g1_i1_len=886
CGGAACGTTAGTCCACCGTTTTGTTAAAACAATAGACACCTGTAACACAGGATTCACAGCGTTGGGCACACTGTTAAAACACACGAAGAAAGGACA
TGAACATGTTTTTCAAGATTTTCTCTTTTCGACAACGACAAAAGGAAACAATCGAGCACACGCGATGAACTACCGCCAGTCCATCTGAACGAACTC
AGTGGCGCGTTGGGACACCGAAAGTGGTGTGGTAACTACCACGCGCCGCTGTAGATCGCCCGTAAACACACCAGTCACGGGAAAGCACGCGACAAG
TGAAAAGCTCGGGGCGTCGAGAGCGAGATGAGTCTTCACGACAGTAACACTGTCTGTCTCAGTCACAATGGCCATCTGGGCTTGGGAGCCCGTCGC
AGCCATGCGATTTGTCTGACTAGAAGACAACTCGAAAAACTGAGCGTAAGTAGCGGACTTACGTGCCCAGTTGTCAGCCATGCGTGTGAACTGACT
CTTCACGTTAAAGGACCAATCTAAATGGCCCGCGCTTGGGTTTTGCCACTCAATACCCAGCTCTCTGAGCACAGCGGCCGCCGCCGCCGTAGTAAA
CGCAACGGTGAATGCAGGGGCATCAGCGTACGATTTAAACAGGAAATCAAACCTGTCACGTGCCGCATCATCATCACCGAGCGCTGGCTTTATGTT
CTCCCACGCACTAGGAGTCTCTCCTGATAACACGCCCCTCTCCAGGGACGAGGCGAGAATTTGAAGATCCAGAGGTTGTAAAAACATGGACAGGCG
ATGTGCGATATACGTTTTCGTCCGACCATCGTTTGCACGGGTCGGCAACCACGCTCGCATCAGTACGTTCTTTGCACTGTTACTCGCCTCGATCTG
TTTGGCGGACCAAACAAGGTTG

>c9031_g1_i1_len=699
CTATCGTTGACATTCCGCCACGCTGGCCCGACAAGCACGGGGGAAACCGCATCTTGGTAGGATCGAACGACGATTTGCCGTCCTGTTTCATTCTGT
GCCCTAACCACCGAGCTGCATGATAAATCTCTTCCATTACATCACCTTTCTTGCATCCCATGAAGGATGCTCCGGCAGACGTATCACGGCGCAAGA
ACTGGCCAACTTCGTGCCAATCTAGCGGTTCCCGTTTGTAAGGAAGTTTGAACGCCTTCTTCGCCTTGGAAATGGCGCGGCGCATTGATGCTTGCT
GCGAGATGGAGAGGTCATCAAAACGGTTCTTCTCGCCTGAGAATTTCAACAAGGACTTGTACATTCCAGGTGTCCCTTGAGGACGGCGAGTGAACC
CACGGACTTCTTCGTAGATCTCACGAGAATAAAGTTTTAAACTCTCACGCACCCAAGGATCTGTGTTTGAATTGGATGAGTACGTTGAGTAACCGC
CATACCGAGCTATCTCGTTCAAGGTAGGCGAGGAAAAGTTTGGTGGAAGCACGTCTTCGCTAGTGGTTGACCCCGTTTTTCCAGGACGGAGGTGGA
GTGCGTCAACTAAATGATACCCCTTAGCTAGGACGTAGTGTGAAGTTGGATCCTGAGAATAATCTTCCATTACGCTCAGGGAGTGAAGGAGGCGAA
AACTCCTCGTGAACACGAGGCCAAAAC

>c25256_g1_i1_len=1070
AATAAACCTAAAGATAATGGAGGAGTAATGCTTGGTGCGGGTATCTTCGGTGGTACTTGGCGTAGAATTTGGAAACTTTTCGAAGGTGATTATGAA
TATTATTGCCTTTACTTTTCCGGCTTCGATTCGTCATTGCCATCATGGATTATCCAAAAAGCATTTAAGTATATTCGTTCACACTTTAGGGAAGTT

CCTGGTAGTGATGCATATTGGCAACATGAGTATGATCGCCTAGTTAATACATTAATTGCTGACCCTAGCGGTCATTTGTATAGGAAGAAGGCAGGT
ATTGGTTCGGGAGATCCTTGGACGTCAATTGTTGGCTCATACTGTAACTGGATGATACTAGAAGCAATTCTTTTGTCATTCAACATAGAAAACGGA
AAGGTGTTTACTTTTGGAGATGATAGCGTTATCTGTCTACCCAAGTACCGTAAATGCCCAACCATTAATGAATTCAGTGAACGTGCAAAGAGTCTA
TTTGGAATGGATATCCATCCAGGAAAAAGTTTCAAAACGTATCAATTATACCTTAAATCATCAATCCCGGTAGAGTCTCAAACAGCCGTCAACTTC
TTATCATACTATTGTAATGATAATCGTCTCCCCGTGAGAAGTCACCATGATGTCACACGAATTATGATGCATCCTGAGTGGAATGAGTCAGACGAT
CCAAAATATGAGCTTGCTCGCACATGGTCATTATATATGTCAACATATTGGAATGACAAGAGTGAGAACTTATTAAGATTGTTCTACTTCTATCTT
AGACCACAAGTTACAAGACAGGAAGTACATTCTCGTGATGACATACGATGGTTAATGAAACACGGAATGCCTTACGATTTAATCATGAAGACTGTA
TCTGACGACCTACCTGATGCTATGGAGATCATACGTCTGTATTCGATATCTTGTCATGACCCACTTTATGCGGAATGGCCAGATTGGGGTGGTGGC
AATTGGTTTGAATAGTAGCGGTGAGAATCCAATTGCCACCACAGCCATTTACTCAATTTAGTCATTAGCGACACCATATGTACATAAAAGGGAGAT
ATGGTGTATAAGGT

>c51155_g1_i1_len=357
CTATTATTGATGGATGGTTGAATCATGATAATCTGTCAAGAGTAAGTGTACCATCGAAGACCAGTCCGGGTCTTCGCTATCGTCGAGCTGGGTTTC
GCACAAAGAAAGATGCCTTGCTTCATGCGATCAAGGATGCTTATTTGGATCTTAGCCGAATGATAAGTTATGGAGAACAGCCGATGGAAATTCCAT
GCTCAATGGCTGGTCGTGGTAAGTTAGTTGGTGGTGAGGGAATTACCCCTGGTAAATCCGCCGGAAGATTGATTATTGTGCCTGATCTTGCTAGAC
ATTTGATAGGATGTATTGCTAGTCAACCATTTGCAAAGATCACACGGAATAAACCTAAAGATAATGGAG

>c37453_g1_i1_len=594
GTCGATTTTCGTATGAGCATGCGACCTGTCGTAATTAGACAAATCACTCCACTTGACACAAGTTGCCGCCCACTGCGTGACAGAGGAGACCCTGAT
GCGCGCTGAGATAGCTTCCGGACTGTGCATGACCTTAGCTACAGGGTCGTTAGTCGGATCGTCAAGGCAGCCGTCGCGTTCACTAGGCAACAAATT
GTTTTCGATGAGTTCGTTTTGGTCAGCGATGCTGCCCGAGTCAGAACCTTCGATATTAGACTCTGATGGGAGTCGGTCAGTCTGCAACTCCGAGCC
CTCCGGCTCGGCGGTGTTACTGTCACGTGGAAGTGCAGACATTATAGAATTTACGTGCAATGTTTTTGATGTGGTTCTGTCTTACTAATTTTTTGT
TTTTATTTGTTTAAATTTGTTTTCGAATGTGAGAAGAGTATACTAACTAAAAGGCTAGCTATAGAAATATGCGTATTGCTCGTCTCACGCTAAGAC
AGACTGGTCAGCTCCGGGCACCACCCCGTATATGTTACAGGCAGTCGTTACACCTACTATGAAATCGGGGCGGCTACTATTAAAAGTGGTAATGCA
AGGCCACAGGCTGCTGCG

>c42393_g1_i1_len=205
CGGGGTTCCCACTGTAGACGCGATGGGCAGTTGCAAAGCACAGTAAAGAGCGTCGTTGACCTGCTCGTCCAACCCGAACTTCATAGCATACATCTC
AATGAAGCTTAGGATGAGATTGGGATTGGTCCACCAGCTAGGGGCAGTGGACGGAAAGACCAAAGTGTGTTCGGAGATAACGCACATCCTGGTACG
CACTTTGCGCCTG

>c43689_g1_i1_len=402
CGACACGACACTCATCAATACACTACTCAACATCGCATACGCCACCGTGGTCGATGAGTCTTTGCGGGCTAAGTACACCAACCCGGGTGTTGTTGA
GAGGTTGTGTCACGGCGATGACATCATCACAGTGCATACATCGCTCTCGAGAGCGCTGCTTTGGAACGATGAAGCCGCAGCTTGTAACCTAGAGGG
ACAGGAGAGTAAGTTGATGATAGACCACAAACATCATGAGTACTTGCGTATTATGGGGTGTTCGGACAACAAGCTCCGCGGGTCATTGGCCAGGTG
CGTGGCTACCTTCTGTAACGGGAATTGGGAGACAGACAAGATAATCGGAGTGTGGGCTAAGCTGCAGGAAAGTGCTAACTCGCTTGCAACATGGGT
GAGACGGGGAGCGGCCAG

>c51753_g1_i1_len=404
GGCTCTTTGACTTTATGCCCAAACTGCCGCTGTCGTCCGACGACGTCCACTCCGTAGTTTGGCGTGAGGCGGGTTGGGCCGGCAAGCCGTCCCTAG
CCATGCAGGTTGAACGGATCCTATATGTGGCAATCGACGATGCTTCCGCTACTATGTACGACCGTTACTTTACCGACAAGGCGTTTATAGAGGTGG
CGTGGCCAGAAGTGTCGAACGCTGGTTCTCCCTATAGATCCAGTGAAATTAGGGGCCGTCCATCCCTCAAACACTACAATGCTTCGGTCAGCCCTC
AGATACTCAACAAGATAACCCAGACCCTTGACCCGACGACTGCTAAGTTCTGGCAAACCAGCTTCCAACTGAGGAACGAGGCCGATGCACTTTGGA
TCTTGCGGTATAGAGCTAAG

>c2871_g1_i1_len=2961
GTTAATTCTTAAAAAAAATGGAAGATTATGCGGTTTAATAGTGAGATAATGCTGTCATTCAAGTAATATCTTAGTGGCTTCAAAACATATTAACAGC
TCGGATTCATTCTACTTGAATAAGCTCAGCTGAGGACAGAAATCGAGTCATCTGAGGACACAACGGCTGGTTTCTTCCAGTCAAATCTTCTTTTCC
CTCTGGCAAACCAACAACGGCCTGAACATTGACACCGACCATCTTCGCAATCCCGATCATCTTATGAACAAATTTCATATGTTTGGACTGGAGTAG
ATTGGACAAGAGCCAGACTTTGACGCGGGACGACTTCAGAGACTCTTCGTGTACAGTGCTTAATGCCAACCCCCACCCACCTTTCACAGTTTTGTA
CCAGTAGAATGTCATGCTTCCCCAGTTTCGTAGGAGGAGGGTCATATGTCGATCATCAGCTATTCCAAATGCGATCACCCATTGCATGGCCCACCT
AATGATGATACGAGTATCCAAGGTGTCTGTCCATATGGTCACCCTTTTGAGTGATGGATTGCCTCTTACTTTCATGCTAGTTTGTTTGGCCCATGT
AATCAAATCTTTGGGATACTGAATAGTTTCTCCTTGTAGATAAAAGGGTAGAATGAGTCGAAGCTCCTCGGCAGTGGTAACTTCCCAATTTGGATC
TAGTATGTTGTTATAAAGCTGAACTATGTTAACCATTTCTCTAGGGTCTGGAATAGACCAATTGGTTTTCAGTGAGGACTCCAGCTTGGTTAGGTC
ATGAATGTAGCCCTCAACCCGATCCGCTTCGATCACAAATGAAGTAGCGTTCTTTCTAAATTCAGCTAGCAAGTAAACTTCTGTGTTGGACTTATT
GGAAAATCCACATCTCAGCACTTTTACAGAGCTATAATAAGATAAAAAGCTAGATATTTGGATTCAGAATTGAGGAGACAGGGCGTAAGTCTTAAA
TATAACCGTATTTGCACTCAATTCATGTCCGAGGTGAGCAACCTTGGAAGCCAGTCTTAGAGGCTGACCGCCGCTCTAATCCACACATTCGGCATC
GCACACTACAAGGTCAATAGATGCAGAGAATTTTACCTTAAACATGCTTGGGTACATTGGCGAAGTGATGTCAGTGATATTATCATTTGTGAGATC
AAGATGTTTCAACCGCACCTCCAAATGCGGAAATCCAGCTAGGCAGGGCAGATAGGGAATAGGAGCAGTCTGCTGGATCTGATCACTGGATTGGAT
CAGGGTGTTGAAGAACACATCTGATTCTGGAAAGGTTAGCAAGGCACTTAAGGTAAACCCGCCAGCTCCATCCCCGCAGCATAGGATTTTTGAAGG
CATTAGAGGTTGAACATGTTTCATCAAGGCGAGACCTTTATAAGGGCCAGTGGTAGGCATACTGACCGTTTTTATTTGATGTCCTGAGTAATCTCG
CTCGGAGGAGCCTAAGCAACTAGGAATCATAATTTGTTGGAAAATAAGAGACCTTCTAGTGCAAGTTGCTACAATTTCGCATACTGGCGGGAGATC
AACAAGAGGAGAGGAGTCAGGGATCTCAAGGGTAAGAATCGGGACCTCTTTGCAGAGAATGTCAATTTCGTCCGGCACCACAAGATTCCCTCCATC
CTTAAGTAATCTCTCCGCCACCATGGATTGATAACCTGACGGACGACCAAGAATAACCCTATTAATAGTTGCTACAATTCCGTGACGGATTGCTAC
GGGGTCATCCTTCCGGCTTCCTCCCACCCAGCTCTGCATCAAAAGGAGAATTGACGGGTGAAGCATCGCGCCGCAGTCTGGCCCTGATCTCACTTC
AATGTGATTGATAGGATGGTTCTCAAGATTCTTGTTGGACCAGTGGAGAAGAATATGTTCCACACACTTCTTGATATTCAGAGACAGGGTGTGCTC
AGACATCTCCGGATCTCCTGCCAATACGGACTTGTAGGGAAGTGACACCAATTCATGATGCAGATTAGGGGTGTGTACCAAATTACCCAGACCTTT
CCAATGCTCGATACTAGATCGGGTCACACGTTCGAGTACTCGAGATACAAATTTTGAGGGGGAACATTCAAAAGCTTGGGAGGAGTAGTATGCAAG
AAACAATAGGGCAACGAATTCCAAAGTTTTTAGCAGAGGGAGCCGAAGGGCCCAATTGATCATAATTCCTGGTTTTGAAAAGTTGGTAATCTTATG
TCCCCAGGAAGGAACCTCCAAAATATCAAAAATTCGAAAAGCCATCAAGGCTATGAGCCTATGTTGGAGTTCTTCCGGAGCTGAAGATCTGAGCGC
CGTGGGTTTAAAAAAGGTAATGTTGGAAGTAACTGGAATTGCTTTCGCCTTGGGCACAAACAAAAAAGGATTGTTCGGGCGAGACACAAATCGAAC
TGGGAGATCTTCACCTTCCATTTTTGTCTCATCTACTGACTTGATACAGCAAGAATTTTTTATATGCAGGTGGTGAGGATGCAATATGGCTGTAGG
AGGGGCATATCTCATTGCAGTCCCTAGAATGCTTACTCCATATGAGATGGGGGACTGAAACATGAGAGTTACGTTTTTGGAGCCTTTGCTATAAGC
TACCAATGGGAATGTGTCAATCCTCATTTTTGTTCCGTAGTTTGGGAGAGTGCTGACTGCTCCACCTCGGTCCACGTAGTCACTTTCCAATCGGTG
GTGAACGGATCCAGAGGTCTGAGACTCGAGAGGAATGAGATCCTCTAACGGAACGTCTGTAAGAGATTGCAAAAGATCCATGATAACACGGTGCAT
ATTCCCCCCGTAGTGAGTCGCCCATCCAATTAAGGCTCCCAACCTTGCTGCTTTGCTTAGCAGGGGAGCAGAATAAGCAGCTACACAATGGCCAGA
AGTAATCACTTTCTGCTTTGTGGAAGAACCTCTATAGGGCATATAGGGTCCGGAAACCAGGGGATCCCTGAGAGACTCTTG

>c2871_g1_i2_len=2057
GATTCAGAATTTGAGGAGACGATTCAGAATTGAGGAGACAGGGCGTAAGTCTTAAATATAACCGTATTTGCACTCAATTCATGTCCGAGGTGAGCA
ACCTTGGAAGCCAGTCTTAGAGGCTGACCGCCGCTCTAATCCACACATTCGGCATCGCACACTACAAGGTCAATAGATGCAGAGAATTTTACCTTA
AACATGCTTGGGTACATTGGCGAAGTGATGTCAGTGATATTATCATTTGTGAGATCAAGATGTTTCAACCGCACCTCCAAATGCGGAAATCCAGCT
AGGCAGGGCAGATAGGGAATAGGAGCAGTCTGCTGGATCTGATCACTGGATTGGATCAGGGTGTTGAAGAACACATCTGATTCTGGAAAGGTTAGC
AAGGCACTTAAGGTAAACCCGCCAGCTCCATCCCCGCAGCATAGGATTTTTGAAGGCATTAGAGGTTGAACATGTTTCATCAAGGCGAGACCTTTA
TAAGGGCCAGTGGTAGGCATACTGACCGTTTTTATTTGATGTCCTGAGTAATCTCGCTCGGAGGAGCCTAAGCAACTAGGAATCATAATTTGTTGG
AAAATAAGAGACCTTCTAGTGCAAGTTGCTACAATTTCGCATACTGGCGGGAGATCAACAAGAGGAGAGGAGTCAGGGATCTCAAGGGTAAGAATC
GGGACCTCTTTGCAGAGAATGTCAATTTCGTCCGGCACCACAAGATTCCCTCCATCCTTAAGTAATCTCTCCGCCACCATGGATTGATAACCTGAC
GGACGACCAAGAATAACCCTATTAATAGTTGCTACAATTCCGTGACGGATTGCTACGGGGTCATCCTTCCGGCTTCCTCCCACCCAGCTCTGCATC
AAAAGGAGAATTGACGGGTGAAGCATCGCGCCGCAGTCTGGCCCTGATCTCACTTCAATGTGATTGATAGGATGGTTCTCAAGATTCTTGTTGGAC
CAGTGGAGAAGAATATGTTCCACACACTTCTTGATATTCAGAGACAGGGTGTGCTCAGACATCTCCGGATCTCCTGCCAATACGGACTTGTAGGGA
AGTGACACCAATTCATGATGCAGATTAGGGGTGTGTACCAAATTACCCAGACCTTTCCAATGCTCGATACTAGATCGGGTCACACGTTCGAGTACT
CGAGATACAAATTTTGAGGGGGAACATTCAAAAGCTTGGGAGGAGTAGTATGCAAGAAACAATAGGGCAACGAATTCCAAAGTTTTTAGCAGAGGG
AGCCGAAGGGCCCAATTGATCATAATTCCTGGTTTTGAAAAGTTGGTAATCTTATGTCCCCAGGAAGGAACCTCCAAAATATCAAAAATTCGAAAA
GCCATCAAGGCTATGAGCCTATGTTGGAGTTCTTCCGGAGCTGAAGATCTGAGCGCCGTGGGTTTAAAAAAGGTAATGTTGGAAGTAACTGGAATT
GCTTTCGCCTTGGGCACAAACAAAAAAGGATTGTTCGGGCGAGACACAAATCGAACTGGGAGATCTTCACCTTCCATTTTTGTCTCATCTACTGAC
TTGATACAGCAAGAATTTTTTATATGCAGGTGGTGAGGATGCAATATGGCTGTAGGAGGGGCATATCTCATTGCAGTCCCTAGAATGCTTACTCCA
TATGAGATGGGGGACTGAAACATGAGAGTTACGTTTTGGAGCCTTTGCTATAAGCTACCAATGGGAATGTGTCAATCCTCATTTTTGTTCCGTAG
TTTGGGAGAGTGCTGACTGCTCCACCTCGGTCCACGTAGTCACTTTCCAATCGGTGGTGAACGGATCCAGAGGTCTGAGACTCGAGAGGAATGAGA
TCCTCTAACGGAACGTCTGTAAGAGATTGCAAAAGATCCATGATAACACGGTGCATATTCCCCCCGTAGTGAGTCGCCCATCCAATTAAGGCTCCC
AACCTTGCTGCTTTGCTTAGCAGGGGAGCAGAATAAGCAGCTACACAATGGCCAGAAGTAATCACTTTCTGCTTTGTGGAAGAACCTCTATAGGGC
ATATAGGGTCCGGAAACCAGGGGATCCCTGAGAGACTCTTG

>c32974_g1_i1_len=2318
GATTGGCCGAGCCAATGGCCAGACCTACAAAGAACGTGTGTCCGAAGATCTGATCAAAAAAGCTACCGAGTGTGATGTTGTAGATCTACACTTCCA
ACGAGAAGCAATAATTGATTCCTTCTCTCATGACGCCAATATGGTCATTCAATGTTACGGTCTTTACCGAATTTGGGGTCACCCGACACTAGAACC
TCTGAGAGGGGCAACTGCTTTGAAGGCAATCACTACCAATGTAAGACTTCTCAGATATCTCGAAGCAGAGGCCATATCAAATAAGTTCAAGGAAGA
ATTTGTGGTTAGGTACTTTTCGAAGAAAAGGACCTGGCCTGGTCTTGACTTGTCAAAACTCAGCCCTCATAATGTCATTCGTGTAGCATATGAAAA
TCTAGCTCCTCCCCCCTTACACCATCCAGATTATCGTCGGAATCACTGGACTCTCATATCCTTCTTGCCCTGTTTCCCAATTGATCCAAAGTTTGA
GATACTCGAGATGCTGTCGGACAAGGCCTTGTCCCTTAACACACCGGACTTGAAGGAGAGCCTAAAATCTGGAAAGGGTCCCGGATCATCAATCTC
TAGGTCTGTTATCGTACAGTGGCTCCAAACTCACCTGGGAGATCCAGAGAAGTTCTTGAGAACTGTGGATGTAATAGGGTTCACCTCTATGGAACG
ATCTTTTGGGCTCAGAGAGAAGGAAAGAGAGGGGAAAATAGACGCGAGAATGTTTGGTCTAGCTACCTTGGTAAAACGTATGTATATAGTACTCAC
TGAAGCCTTATTGGCCGAGCACATTCTTCCTCTATTCCCTGAGATCACTATGACAGATGACGAAATGGTACTCGACAAAAAGAGAATCTCTTTCAA
TAAAAAACTTAAGAATCATTTTCACTTGTTCACCAGCCTAGACTTCTCCAAGTGGAACTCAAATATGCGAGAAGAAGAGACAATAATGGTCTTTAA
ATCCTTTGATGAGCTATTTGGTTTAAAAACATTTTCCAGAGATCTCATGAATTGTTCAATCCCGGGTTCATGTACCTGTTAAACGGCTCTTACAC
TCCCATGCTTGAAAATGATGAGTTTGTTCCCATGATTGGAAGCTGGTTCGGCCATCTCGGAGGCATAGAAGGACTCAGACAAAAGGGTTGGACAAT
ATGGACAGTTGTATTGATTCTGCTTTGTGCGGAGAATTCTCCTATAACTCTTCGCATGATGGGACAAGGGGATAACCAAATTTTAAAGCAAATATT
TCCAAAAAATATGGATGAATCGACTTGTCTTGACTATCACTATCAATTCATTGGGAAACTGAATGACATGCTATCAAAGATTGGACCCCCCATTGAA
ATTAGAGGAGACCTGGACTTCCAAAGATCTCTTCATATATGGAAAATATGTTGTTTATAAAGGCACTGCAATAGAGACATCTTACAAGAGAATCTT
GCGGATGTTTAAAATGTCCAACGAGGATTTTCCAACTGTAGAATCCGCGATATCATCAATGACAGCGAATGTATCTTCCGCTCTCTCGTGTAGTAC
ATCCATCAATTCTGAGTACTACATTTACACTTCAGAATTAGTAGGACTTTTCCAACTGTTCCTCACCAAGCCCTTTCTCCAGAAAAGAGCTCCGAT
TAGAGACCTGACAAAACCTGGAAGAATAGTGATTCCCAACACTCCCAATATCCCGACCGGTGCGGTGATAAAAAAAGAAGACATCCAGTCTGAAGC
ATTGTTCATCAAGCTTTTGCTCCTCCCTAGAAATCTGGGGGGATTTCCTGTGTCTTATTTACCGTCCCTTTTAATTCGTGGATTTCCTGATGACTT
AGAACTAAACATATCCTTTCTGAGAATTGCTTACTTTCTGGTGCGCCCGTCAATACAACCGTTCATACGAAATATCCTCAGTCCGTTATTCTCTCC
CCACATCTCCTTTGAGCTTCTCCTTGAAAATCCAACATCCTTGAATTTGTATGTTCCCTCCGCCCCTGGAGAAGCCAGACGAAGTTCCATCATAAA
ATTCCTTCAGACTGGTTACGAAATTGAGAATCCTAGTTTCAAAGAGTTTCTAGACATACTAGGAGATGAAGAAGATAGAGACTTAATAGAATTCCT
GAAGACGTGTAAGCCTATGAATCCAATAGTCTTATCAGCTATCTATGACGCTACTGTATTGGCTCGAGCCCGCCATGTCGCTGGGAAGCTTCAGAA
AACCCGAACCCTCTCAAAAATTGCTGTTCAAGACGGGGGAGTGAATCTGTATGATATTATTAGAGATTCTGAGATGAATCACTTAAGAAGCGTTTT
TCATATAGTTCAGG

>c51091_g1_i1_len=640
TGAGCCGCGAGATCTGAAAATACAACTAAATGATCCCTAGATAGAAGAGATATGCCTCGGTCCGACCAGACGAAGAACAAGCTCTTGCCTGCGTAG
AAACGAACTCCTGCTACGTCTGCTGACCACAATCCTTTACACTTAGTTGGCTTCCACCAGTGATCTCCGATCGAATGGTCACTGCCAGATGCCAAG
ACACCCCCCTCCAAAAAATTCCTCAATGCATATTTTTTGAGCAGCATTACTCTTTGTCTCTGGGAGTAAGGAGGGAATTGATCGGGGAGATTGGTG
TTTCCTGTTCGATGAACTACTCCTAGATTAATCCTCCACATTTCTGTCGCCACTGTCTGTTGGCGCGTCCAAGCATCTAATAAAGCATCAGTGATC
GGGACCTGTAAATTCATTAACTCCTTGTGATGAAAAGAGGAATCAAATCCCTTTACTCTCTTAACTGCGACTTGAGATAGAATGTATTCCCACTCT
TCTCTAGCTCTATTGTGCCGGGGAACATATTGGCCTATTTGTGGCCTTCGGAGAGCAGCTCGAGCGGCGGTATACTTTAACGGTGAATTCCCATGG
GTAGGAAGAGGGACCTCTTCATTGAAATGAGGTAAAAAACGTTCTCGGAAAGACTCCATGATTG

>c7211_g1_i1_len=447
GAACAATTCATGGCCCCTATGGTCACTGGTATACTTACTGGCATGCTCGCATTCTTGCTGACTGCATATATATGGAGGAAATGGCTTTCATCAGCT
ATCACATCCGGGTGGGCCAAAATCCAAAGTAGGTACTATAGAAGGAAGTTTAGGAACGAAGAGAAAAAAACTGACAGAATAGCTATGAGGTTGAGA
CACATGAGGGCACCATCTCTAACTACCATGGCCATCCTTACTTTCATATTTTCGACGGCATTAGGATGTGACAATACATTGTACATTGGTTCCAAA
GAAGTAATCTGCGACAACACGCACTGCATTGAGTCCAGCATGTTCGACCTAACAATAGAAACAGGATCCACATTGTGCTTCAGAGATCAAAAAGGT
GACCTGATCACATTATCTACGACAACATCATATAGGGTTGTGCGAAGCATGCTTATTTACCAC

>c8461_g1_i1_len=308
CGGACTATGAACTGAGCACTGAGCAGTTCTGGCAATGCAAAGGTGCAGGCATTTGCTGGAAGGGTGGTTGCCACCAGTACTCCATGCATCCCAGCC
TTAAGAAGAACCCCAAAGCAGGGTCTATTGCAAGATACGGGTGTGACATTGACGTAATTGGCTGTGACACATACTGTCATTATGGGGTGTCTTGTG
TATATTATAGATGGCAGGTTGACCCAATTGGTGCACCAGTCCCGGTATACAAGGCAGTAACAAACTACTGGGGGCTGGATCTTTCAGTAACCTATG
GAAACCAGACTAGAGTGTAT

>c8461_g2_i1_len=809
AGTCGCAAATGGGAAAGTGTACAACGTTGATGCTTCTAGACAAAACATGCCAGAATGGGGTAAACTTGGTGACTATCAATTAGATACTAGAAACAA
CACTGTGGCAATGAACACACAAGATGTCAGATGCACCACACATTCATGCCGGACAGTCTGTGTCATGCCAGAACCAAAATTAGAAGGTTCCTACAT

GGCAACAATCATGTTGACAGCATTCAGTCAAAATTCGTCGGGAGCAATGCTATAATGCTAACAACACAGAAGGTAAATGATATAGCCAAGTTGTTA
ATAGGCAATGTTGAGATCAATTCTCTGAGAGTTGCACCTGCCAAGTGTGCACTAGATGTTATTGGTACATTTTCATGCAGCGGCTGTGCAGTCAAG
CCATACATCATAGTACAAGCATCTGAAATATCTGAGCCTGGACTAGTCCCGTTCACCTCAAACTGCACATTTAGCACTTCGAGCATTGGCTGTTCG
ATGGAACCAATGAAGCTTGAATTAAATGACATGAATAACAATTGTAAAATATTCTTCCCAAGTCTTAACAGAAGTATCACAGTAGAAATGAAATAC
AATTATCTAGGCCAGCTTGATCCAACCAGGGAGATTGTGTCACGTGGAATAGATGTGTATGTATGATTTGTATAGCTTGACCATGAACCAAAATAC
TGGAACCATATGTCTTTGTTTACCTCGACGATATCGTCGTGGTAACGGAGACATTTGAGCATCACGTCCAGCTACTTCAGGAAATTGCACGCAGAT
TAAACAGAGCCAACCTAATGATAAACCTGGAGAAGTCTCAG

>c9280_g1_i1_len=2438
CTCCTTTTTCTTGAACATCTAAACGTGTAAACCAGATTTGCTCTAAAGATCGTCATCAAGTTGAATTTCCTCCATGTCATCATCATCATGCGCCTT
GCCAGTAGTGAGGACTGCGATTGCTTGCTCAATTAACCTTCTCTCGTTCTCACTCCATTCAAGACCAGAGGCTGCCGTTTGAAATATCCCGGAAAC
ATGAGAAAGCATGTTGTGGGAGCTCCCAGCTGTTCTTAAATGCATGGTGGGATCCGCAGTTGTCAGTATTCCTAGAGCTCTTGCATTATCTTTCTC
CACATGGGAGTGGTTAGGGGTCCTCACATTGGAGATTGTGGCGTTGGCACGTTTGATGTAGTGGAAGATGGTGCGCGGTGTATCAACTCTCTCCAC
AGTCAGGCACCCAGTGTTTCTGTCCCACATAACCTCCTCAGTTGGCTCACCTGTGAATGGGTTGTCCGGATTTCTGATCTTCAGGAAATACTCCAC
CTCATTGTCCTGCACTGCAAATCTCTCTTTGAGACTGGTCTCTTCCAAGTGACAATTGGTGCTATTCCACGGGTTGCACCCTCCATCAGCGAGCAC
TCTAATTTCCTTCCGTATGCCTCCTTCCACGTCTCCCCTTTTGTTCACACGGTACAAGCTCCTGACATTTGTGATTCCAATGCGGATCTTTGCTTC
TGCTCCGAGGTGGTTGCTTTCGATGTTCATAAGGCTTACAGAAGTTGACTTTGCAACGTTGTAAAAGTCATCAGGCGGATTTATCCCCATTGGTCG
GATCCGCGTGGCCTCTGCACCATATTGACTCACCAACACGCCATCTTGATACCAGGAAGTGTATTCCCCTACACCAAGCCGGAAGTACTGGGTTTT
CTTGTCGCTCAAATTTATCCCACACATCTTCAGATTAATCTTCTCTCCAATTACCAGTGTCTTGATCACATCCATGTCTGGTCCAGTGTATATTGT
CATACTGTCGTCGCTTGACCTGAGAGTGTAAATGTTGCCTAGCATCGGTTTGTTCCATCCCACATTGACCAAGCCGTACGTGGTAGACAAAGCATT
CATCATGCCCATCAGCATCCCGCCAGGTGCACGGATGTAATTGTTGCCATAACGCAGGGGAATCGCCTCTTTGAGCCATGACTGAGTTTGGGTGTT
GAATTGGTGAACCTTGTCAGCATCAAACTGGACAGTGCCATGAGTGTGAGGAGTGTGACACTGCAGCCCATCACCCAGAGTGATCATTTTCGTTGC
TAGGAGCCAGTGCGAGGCCATGCCAATTTCACGGAGGAGTTCTTCATATGGGGTTGGTATTAGCAATCTCTGCTCGAACCTGACCTCCTTATCAAA
GAGCGTCTTAGTGAGCATTCCGAATGACATCGCGGAGAGACACTCATTCCACTTAGTAGCGTCCTGCGTGGCTTGAAGAGCATGTACGGCATTCAG
ATCACCTTTACATTCGCTTGTGATCGACGAAATCTTTATCTTCTTCAATTCCCCACCAATGGAGATCGTGCTTCCCCGTATAACTTTGCCCAGGGC
AAGGTGGAATTCTTCAAGCACTAAAAACAATGCTCTCTGGAATATTCCCGTTGAAGCAATTGCTCTTCTATTCAAATGAGCTCTTTCTCCGTGCTT
CAAGTAGGAACAGAAAGATCGCCCTGCATCCATGAGTTTGCGGTACACCTGCTCACCCCTCCATGTTACCTTGACAACCCTCTTCTTCCTTATCTT
TGTGGCAATTAACTGTCCATCCACGTATTTCATCTTCTCGGCGTAGTAGTATTCAGCCTCGTGGCCTACCACTTCTGGCATGCTCATCAAGCGGAA
GAAGCCTTGAATGAGCTCCATAACCGTATGGTGGTCGGGTAACCCGTTGTCCTTGAATAGCTTGTTGACGTTGATGTAAGCTTGGGGGCAAGGAAC
ACTTCTTTCTTCAAAGGCGTCCCATGTTTGACGACCCTTCGTAAGCGTATCCGAGTTCAAAACGCACATCTTGCCTATTACGTCCTCTGCCGTTTT
CCGTATGGCCACGAAGTTCTCTTGCAGGAAAGCACAGCACATTCTATGGAGGCTGCCCGGGCAGAAGTTGCTGGATATCTCGTCAAACGGGAAGCC
GTTCTGCTGTGTCCACACTGGATTGTCAATCACGTAACCGTCTATGACAGCCTCCCTGTCTTCCGTCTTCCGATTGAAGTTGATGGCCCTCAATAC
GGTTTCCGCCACCTTTGGTGCTGGAGTGCCCGTGCCATTTGGAGGGGGGTTCGTATACAAATACAACGCACTAACCATGCCTAACGTGTCCATGTC
ACGCTGGTCGGGTAGTTTGATGTCAGTCCTCCTCGTTTGGTCGAAGACTGCCGCATAGAAGGAACTTGGGATTTCCGTGAAAGGATCTGGTTCTGC
CATTTCGATGTTTTGTTGAAAGGAGGTGTTAATACGAC

>c33645_g1_i1_len=930
GTGGGAGTGTCTTTGTTGATTGAAGATGCGTATTGTTCCCAAGATTTTTGTCAGATCGTCTAGGGAAGGAGAAAAGGAGGGGGCAAGAGACCCAGT
ACATAGGTCGATACCTCCATGTTTTAGTAAAGGAAGATAACAGTTTATCACATCTGCAGTTTTCTCTGGACATTTGCCAAGCATAATTCCAGATCC
GCAGAGTAGACACCCCTTGGAATAACTCTTGTCATCCAGGGAGTACTCAATTTCAATTGAACCGTCGATAGGACCAGAGTGATTAAAGACGAGTAC
CTTCACTGGAAAAGCAAGTTTATAGTAAAGAACTCCTTTCTTTTGAGTAGAAGAAAATAATTGGTGGCCTAAATACTGAGTTAAGTAGGGTCTTTC
ATTGATAGGGATAAGGAATCTTGAAAGTTGTAGGAGCTGTTGGCGCTTCTTACAAGCCTCAGCCTCCAGATTAACTAATTCCTTTGTTGTCACTTC
TTTAAAATCATTCAAATTCCCCAAATAGTTCGGAAGTAGTCCATGATGCTTGTTCTGCGAGTAGAAGGAGCTTCAATTCTCCTGGAAAATCATACA
TAGATCTAGAGGTAATTGTCGCCAAACTTCTGGTGGGTCGAGCTCTTCGAGGGTTTTTGAAATAGACCCTAGGGGGATCCCAGGAGTTCTTCTTCC
ATAATATCTGCCGGAGTATTGGGAATGCACCATGGTTGAGGATCGATCTTCTTCGCCTTTGAGAAAACAATCCTCCACTTAAAGAAGTGACAGGAG
ATCCGTTAACCATGACAAAAGGGGGGGGGTAGAGAAAGGTCGTCCTCGAGCTTTTGGTTAATTCTTCCAGGGTTTTTGATTTTGATGATAAGTTTT
CTTTTACGGAGACAAACGTATAAAAATATTCCAATTATTGTTAGTGTTGTAGTTGAGAGTCCTCCG

>c50692_g1_i1_len=383
GTTGGCTTGCATATTCAGACCCAGACTCGAGAACGATTTGTCAACCTACTAGGTGCCCCCTCAAGCGCGCTCCCTAAGTGCTCCACTTTCGCCCCA
CCCCATGCGGAGTTTTTGCAGAGGTTCAAGGAGTCCATCCCCAAAGCAACTCCGTTAGCTCGTCAACTGGCCATATTAATGATTGAGGGGTACTCT
ATTGAGACTTCGGAACACGTCCGCGGTTATTGGAAAGCAGGCTGCGCTTTGTCCCTATCGTATACCGGACTGGGCGCTATTGGATGGTTTGAGAAA
GCACGCGATCGGCTCAAACTTTCTGGATATGACTTGTGCAAGCAGCTTGCAATCCCTGCCATGCTAGACTTTATCGAGGAGTATCTCCAGCTCCG

>c8881_g1_i1_len=6498
CACCCCCTAAAATCAATCTAGATCCTTTTGTAAATACAAAATGTCGAACATCGTCGTTGGTGCGCAAAGCGTAAGCAGGACTCTCAGCACTAGGTT
AGCGGCACCTAATGACCTGGATGCTTTCTCTAGAGGTCTAATGGAGAGTTACCTTCCCAAAAATCAAAATGATTACCAGACTTACAGGAGATATAT
GCATGACATTTTGTGTCTTGCTTTGGTTGAGATGGGAGTTTATCATGAAGAGGAACTGACTCTAAGCAAGTTAGGCATCCATATGAATGATCGGAG
ATTGAACATGCAGAAACCTGATCTTTACACCAAAGAGTTGACCAACCTGGAAATTGGGGAAGTAACATTATCCTACAATGCGCAGGCTGACTCTGA
AAGGAAAAGAGCCACCTATAAGGAATTCTTAGCCACCATCACAGAAAAATTGGGTATTCAGGTTAACTTTAATGTCATCGCGGTGGATCTTGCTGA
TACTGAGTGGGAAGAAAGCTTACCACAACTACCAGCTATTCATGCAAAAATGCTACGTTCTTTCATCAAGAATCTCACCTACATTCACTCACAGAC
CAAATTTTCTAGTCATAGGAGAGATTTCTCGGAATTTTATGCACTAGACAAGTTTAGGTTTGAGCTATCTCCTGATATTGGGGATATTATGACAAA
GGCTACAAGAGGGCCAATTACTCTTGATGAAACCATGAAGAGGCTTGAATTTCCTGGGTATGAACAGCTTTCTGATGCGGAGTACATAGCTCTTCT
TGCTAAAGGGATACTAACAGGGCCAATAAGCAAGAGGCCTGCACCACATCCAGAGCCACAGCTCGGAGATGAGTTAGATAGGAGCTGGGCCACTTT
CTGTTCCTCTAAGAAAAACACTGACAAAATCCCAAGAATTCTACAGCTAGGAGGACCTCTTGAGTTCATTGAGACCTCCTACAATTTTGATGATGT
CGTGAGACAACTCAGGTCTACAAACAGAACAGGGGGCTATCTTGATATGATTAAGTCTTATTTGGCCAGTAATGATCCTGATGAGTCCCACATCAT
AACACTATCTCTGTCTGATTCACAACTACAGAAAGAGCAGATGGAGGGACCTGGCAGGAAAGCCTACCTTCGGAGAAACGGTCTGAAGACAGAAAG
GAAGGCACCTACACACATAGGAATTTCTCGAGAACACGAGGTCTTACTGGAGGACCTGGTCCAGCAAGTCGAGGAGCTCACCATCAGAAATGAGTT
GCATAGCCCAGCACTACCTCCGTTGAGCTCCATTGGTTCTTCAATGCACACAGTTTTAGAGAAGGTTCTTGAAACACACATGGCGAGCCCCTTTTC
TGGCATTTCTAGATTTTATCAGAGAGCTTCACAGGAGATCATCATAAACAGTATGAGAAGAAGGAAGAATAGACAGTATGTTTTGTGCACTAGTGG
GTTTCAGGATATATACATCCTAATTGCACCAGGACCTCAGTTAAGAACTGAATCAAACACAGAGTTTGTTAAGTTCATATCATTCTGTAGACCCAT
AAGTCACATACTATCTGCTCCTTGGCTGCCCACCGGAGATCATTGGGAGTCCGAGTGGTTATCAGTAGACACTGATAGGCTAAAGCATTGGTCAAG
GAGTTTTGATAGAACTATTACAGCTTCTGTGGCATGTAGCGAGAGACTGGTTGAATTAGATAAAACTATCACCAAAGCTTGTGTGGAAGAGGTCAA
AAATGGTAATTATCAGTTAATGATACTGACTTACCTCGAGGATAAACAACTCACATCATTAACAAATCAGACTCTCCGATACTTGTGGATGAAGTC
CTTAGGAGACAAGCAGTTCTCTGGGATCATGAGCAAATTCCCTTCAAGGATTGGTTCAGTGATTCAATCAGTCATGCTTCAAAGGGCAATGAGAAC
AACAATGCACATCAACAGGAGTAACTTATCTGACTTTATTAAAGTGCCAATGTCTCGCCAGGATGACCAGACCGGTATGTACGATGAAACCACAAC
AGGAATTTTGGGCAAGCTGCCAAGACTATTCACAAAGGGAGATATGGTGCCACTGTCTTACAACCTCAATGAAATATATTGGTGCATGATGTACAA

TAAAGATAGACAAAACCCTGCTCAAGATGCATTGAGCATATTAAACAAAGTCCTTAAAGAGGAGCAGAAATATGATGAGGAGATAAACAAAAGGCA
AACTCATAAAGAGAAAGCAGACTACACATTTGGTAACACGACCCTATCACAGGATCTTGAACACATACATGCTTCGAAACCAGAAAGTCATTTCTA
CAGCAGAAAAGCAGTGCAGGTTGGCATTAGGTTGCAATCTGTACATGAAGACAATGTAGCTGCAGATTACAGTTGGCTAACTCCAGCCAAGGTAAA
TGCTATCCTAAGCAAGAACTTGTCTGAATATGCAACTTTCAAGGCTTCAGTTAAGGACCTGTGTCGGCATGTGGATGTAAATGACCTCAAAGACAT
TGAGAATGTAGGTAAAAGAACAAAGGCGGTCGAGCTTGTTGCTGAGTTGGTTTCTAATGAAGGGCTCACACAGGCATTCGAAGTGGCAATGCAATT
TTCTGGTGACAATAACAAGGCTTTTGATGTGATGATTCAGATCTTTAAAAAAGGTCAAATAGGTGGAGTTCGTGAAATCATGATTCTCTTTATAAA
GGCTAGAGTACTCTTCAATATAGTCGAAGAGCTGTGCAGATTGCTATCCAAGTCAGACAAGAGAGAAATCCTCACCAAAGGCAAAGACAAGAGGTT
GATGATGAGAGGAGACTATGAAGAGGTGATGTCTCAGTTTCCAAAAGGGACACCAGTACAGATGATTAAGAATTCATATGACATGACTACATGGGC
ACAAAAATTTATACCAACCATTTTCTGCGCAATATATACAGACATGCTACAAGAATATCCGGACTTAAAAAACCTAGCTTACTTTGTGTTCCTCAA
ACACACCAACAAAATGATTGAATACCCAAGAAAACTTGTTGAGATGTGGTCCAGACACAAAGAGGAGAAACACCCAGAGCCGTGGTTGCAAGCAGC
AAAAGAAAAATACCTAAAAGATGGCATTCCATATTTCATCAACCATTCAAACATGTGTCAGGGGATACCACATTATAACTCAACTGTGCTGGCCTT
GTCGTGTCAAAGCCTAAGGGACTCCTTGTTTCGAGAATGTTTAGATCAGCTGGGGCAAGATTGCCACATTAGATGGAAGACCAGAGTTGGATCTGA
TGACAAAGGAGACATGATTGGAGTCGACATGAGTGCCAAACATTCATACAGACAATACCTTTTGTTTGAACAATGTGCGCACGCAGCTGAAAGGTT
GCACTCTATGGAGTTATCGGTCAAATCAGCATCAGGGAACATCATATACGAGCTGAATTCAGCTTTCATGGCAAACCTTGAAACCATGTCTCCAAC
TATCAAGTTCGCTCTAGCATCATGTGACATGATCCAAACATCCTCCTGTGCCACCTTTGTGAATGAATCCTACGGCCGAGTTCGCCAATTGAGGGA
AAATGGGGGGTCATCTGTCCTTTGTGGGTTGGCCCATATGGTAAATGCTGACCACTTTTACATGCTATTCAGGACCTCATCAGGAATGAAAAATGA
CATATCAGCCATTCTAGGCGTGCCTAAAGAAATGGTGCCTTATGACTTTGGTGTCTACCCTTTTTATGATATAGACTTACAAGAGATCGTTGGCCC
AGAATTCCACAACTACATGATCATGACAAATAAGGACGTGCCTGTCGAGATAAAGCAACTTCTGTACACCCCTCTGTCAAAGGATGACATAGGAGA
GGCGTTCCCAGACCCAGAGGAGCCTTCCTTGCTCAAAAAAGACCACTTTGGGATAAAGCAGGGGCTTATAAAGCAATTAACAGCAATGAGAAGGAG
GCTGTCAATTGAACCAAATGAAGTTGACAAATTCTTCTCTGACAACCCGTTTCTGATGGTTAGAGGGCCTGAGAACAGAGAAGAAACAATAAAGGT
TATCCACTCAAAACTATTAACTAAAGGTGCAGCTGAGGCCTTGAGAAGGACCTCACCGGCAATATACTTAGGTAGACTATCTGCCTTTGAGTCAGC
AAGAGCATGGAAAATTAGGAAACCCAACGGAACACAGCTAGTAGACATCGATGAAGGAATAGCAATGGAGTTAGAAGATGATGTGCCTGCGACTTA
TTCAGAATTCATGTCTTGGGGTATTAAAGTTGCAAGGGCACAGAACTTTCCTGTTGATAAGATGATGAACGTCATTTTCCCCCAGGCAAACTCTTT
TGAAGTCATAAGACAATTTGTGGGGCAATTTGGACTCAAGAGAGAGTCAGCTAAGAAGTATTCACAGGCTGTCCGCACTTGGGTTGTCAACAATTA
CAACTACAACTTTAACAATTCTCTAAAATCCATCTTAGAAACCTCTTTTGGAATGTCACAGTTGTCCAGCAAGGAAGATGTAAATGAATTCAGGAA
ACTTCTCTCTTTTAGACTGGACAGCTATGATGGATTCATAACTGAGTGTAAGGCCAAAGGATTGAGGCCTTTGGACATCTTTTTCTACATGACTAA
GATCTATAAATCCAGTAGGAGCACCAAGGTGCAGGCATTTGCTAATGGACCGTCAACTAGCTCTTTAAATACTACCCTAATCTCAATCAAGAGATA
CAGCCACATTCCACACACAGAAATGGTTATGGACCTAGGTCTTGACCCTGAAGATTTCCTGGAAACAAACAGGCAAGATGTGAAAATGGAAACACT
GAAATTTTGCTGCAATCTACTAATTATGTCTTGCCAGGAGACTGTTAGCGGCAATGGGAATATCCTCGATGAGTGCAAGATTGCTGGTGTGACCAT
GACAGACTGGTGTCTTACCACCTTAAGATCATTGAAGAGCATCTCAGGATTTGACCATCAAACTAGGAAGGTTGCTGTGTTCGTTGCCTCACAGGT
CATGAGCACAGGAGAATTCAAGGAGAAGTTGCTAACTTGGAATAGTCTTAATTTCACCTACTTGAAGAGACAAAAGAAAGTGATTTCTGGAAACGG
TAATGTTTCCTGGGAAGGGGACCTTCAGTTGCTAGTGAACTCAGGGAAAAATACTTTTGTGTTAAATGTGGTGAATGATGTGCACTTCATAGTCTG
CAAGAGGGTTGAGGATGTTGAAACCTTGCTGGTGACTTTGAATGAGTTATGTAGGATGCTTGGATTTGACCCGAGATCTTTCTTTACCAGGAAACC
ACTCAAGCCCAACCTGTACTATCTCTCTGATAATTCTAAGTCATTGATGAGATCAAGCAGTCGCGGACCAGATCACAACTGTCTGAATCTAACTGA
GCAAAGAACATTTAAACACATGAGGTTGCAGGATTTTGACAATTTCAGGATAATCACTAGCCTAGATAGGAAAACTCAAGCTGTCTCTGTGTGTTT
AGAAGAAAATGGAGAGAGAAGCGCAACACTATGCCATTTCCCAGGGAATTTTTATCCAGCTAGCTCCCCACGTGGGCTGACAATTTCAGAGAGCTT
CTGGTTCAAAGGCCTGAGACTGTCAACTATTTTGCGCAATCCAGACTGGTTTTATAATTATAGGCTGCCATCTCTGACTGATAGCGACACCTTAGA
ATTCTTCCAGAATGATGTTAATTTTGATGCCATATTACAGCAAACAACAGAAGAGAAAGGTAGAATCTTGGAATACCTCCAAGTTATGGATGAGAT
AGACGAGGAAGTGTTCAATCTCAATGTTGGAGGAACTGTTATGCAGGGTAACACAGTTGTGCTTGAGACAGACTTTGAGAGCGATGTTGAGAATGT
TGAGGACCTATTCCTAGATGCCATGACGAACATTAAAAGGGAAGGAGCCTTTGAGTTTGAGCCGCTGGAGCCTGAAGGATTTGATTGGGCTGAGGA
GATGGAAGATGTGGAGAGAGAGGGTGACATGCCTCAAATGATGGAAGAAGATGGCATCAGATTCGTGCGGTCTATGGGATATAAGAGAAAAGCAAG
GAAAGCCAATATGCATGTTGTGAATCAAATGCAGTTAGGACACATTCTGAAAGAGAGAGTCTTGAATATGTTTTTTAAAAATGCTGCAGTAACCTC
CGAAGACAGAAGGTTGCTGCCAAATTACTACATTTGGATCAACTCAAACAAAGACTCAATTGGCACAAAACTGTCCGCAGAGCTTCAGAGGGTGAT
TCTGGTGGAGCTGCATATTCACATGGGAGCCACTTTACCTGAGCTGAGAAACTCCCTAGATAGAGCCAACCCCAGCCTGGCAACAGCACCTGCAAA
ATTGATGAACTACACACAAGGACAGCTAACAGATCTCTACACCCAACTAGCACAGAATGTTGGATTTTCTCCGGATCCATATGAAGAGAGCGAGGG
TTCAGAACAAGAATACTGATTTAGACCTAATCACATCGTCTTGTCTCTTTCAATCTTCAGTGTTAG

>c4670_g1_i1_len=251
ATATCTCTTCAAGCTCTGTCCCGTCAGTGGGGGCATTTCCCTTCCATATCCTCCCGTGGAAGACCAAGTTACAAAGAACATCGAAAAGGCAGATCG
GGATCGCCCACAGGACTCCAACATCATGTCTCACATGCATGAAATTGATCACTGACGTTAGGACATACAGGCAGAATTTGTAAATACCAAAAACAA
TTAGGATGAAGCCAAACCATCCCCCTGCCCTTACAGCTGAGTCTGTAAATCTGTCCCAG

>c4670_g1_i2_len=212
ATATCTCTTCAAGCTCTGTCCCGTCAGTGGGGGCATTTCCCTTCCATATCCTCCCGTGGAAGACCAAGTTACAAAGAACATCGAAAAGGCAGATCG
GGATCGCCCACAGGACTCCAACATCATGTCTCACATGCATGCAATTGATCACTGACGTTAGGATGAAGCCAAACCATCCCCCTGCCCTTACAGCTG
AGTCTGTAAATCTGTCCCAG

>c7681_g1_i1_len=734
GGAGTCTAGAAGTGTAATGGGGAGATGTGGAAAGTCTATTGATACATTTCATCATGCCGGATTGTTCTCCGAGATTGTCCGAATCGGAAGACACGA
GTGTGACACTATGCATAAGAAGAAGGTGTTCCGTATGGCGCCCTTAGCGACATCCAGCTGAGTTCACAGGGACAGACTAGGTATGCATATGTAAGC
CGTGGATCAGTAACCTCTGATGGATCCTGCACCCCCGGAGCCGACATGGTGAGAAATGGCCGAGAGTACGATCGCCCCCTTATCAACACAACACTA
ACTATAACAATCTCGTCAGGGACCGCCATCGTCTCAGTTGAGGAGAGTCTAATAAAGTTCCCCAACGGAAATAACTGTAAACTCCAAGACGAGACA
TGTTTTGACGCAGATTACGGCGATGTTTTCTAGACGGTTTCAACTCCATCCTGTGAGGGATCGGAGTCTGAGAAAAGCCTTGTGTACGAGGGCCCC
GGGACGCTGGTGCGAGATGTCGCAGGAAACACCTCTATTGAATACGTTCATGTACACTATGCGGGGTACGATTTCCAAGTGCTCTTAAAGAACAGA
CAAGAGATAATTTGCGGATACAGGTCATTTTATAACGAGCATCCGAGTCTGTTTGTTACCTTATTATCTTCTAACGGTCCCAAGTTCCCACTGCGT
AGGAAGGTAACCGCAGTCGACGTGCGTCTTCTCAACTTCGTGAATTCTAAGCTGGTTTATTC

>c23353_g1_i1_len=208
ATGGAACATCTGTTGTAGCTCGGCCGCCTGGTACTTTTGGATGATATCGCCTCGATAAAGCCCTCCTCCAGATATTCCAGACAACTCTCTAAATTC
GTACTCAACAGCGTTCGGAGCAATGGAATGCGGAGGCCGGGTCTCAATCAGTCCGTGGTCAGTTTTCACGTACCAATTTTCATTCAAAAAATATTT
AGGTAGTAAGTCAGGG

>c49221_g1_i1_len=303
GTGACATACAGCTCAACTCGCACAGCCAAACTCGGTATTCTTACGTGAGTCGGGGATCTGTGACATCAGACGGATCATGCACTCCTGGAGCAGATA
TAGTACGTAATGGCATGGAATACGATCGTCCTCTGATCAACACCGAATTGATAATAACTATCTCTTCAGGGACAGCCATAGTCTCTATCGAGGATA
GCCTGATTAAGTTTCCTAACGGGAACAATTGCAAGCTACAAGATGAGAACTGCTTCGACGCTGATTACGGCGATGTGTTTTGGTCCGTTCCCACTT
CCTCCTGTGAATGAT

>c9237_g1_i1_len=382
GGCTAAGCAGGATGTGAAGGACGGAGGCGCATGCCTCGCAGCCGAGTGGAGCGGTTTTCCGCGGCCTCGGGGTGGAGGTGAGGAAGGGCTACCTCG
AGGACCACATCGTGAACTTCCTAGGTTTAATGCTGTGTCTCCAGGTGATTTCAAACGCCCGGCCACGCCAACGGCCATTGGCCTACTGGGTGTTAT
CCTGGTTGGCTTTTGCTGTTTTTGGATTGCCGTCGGTCTTCTCATGGTTGGTTCTGTTGCACGCTTCAACGTCGTCGATCCGCTTGTCCCAGATGA
TGATTCCTCTGCGGATGCTACTTCACGTAGGGCATTGCTCGATGTTCTTCCTAGGGTATCTACTGGAACACAAACTGAGGGCAATTGGGATCGG

>c25880_g1_i1_len=513
CGGAAATACATTGTTGAAGAGACACTGAAAAACGTGGAGCTCAAGCGGCGCACCCTCATTTTGACCCCCACCCGAGTTGTTATGGAGGAAGTAAGA
AAGGCCTTCGGTGAAACGGAATTCAAGATAGCAACAGCGAAAGCAGCCCATCACAACATCATCTATGGTGCAGCGACGAGGGCGAACTGGGCGAAA
TGCCCCAGGTACATATGTTTATCCAGTTGACGCTGCTACCAAGGAGGATCCATATGACTGGGTTTGTTGGCTGGAAGCACAAATGGTGTTAGACCA
GCTAAGCATGCCACTAATGAAGGAAGAGTATGCTTATGGACAACCCCCTGGAGCTTTTCGTCTGATCCAGTTGACGCTGCTACCTAGGAGGATCCA
TATGACTGGGTTTGTTGGCTGGAAGCACAAATGGTGTTAGACCAGCTAAGCATGCCACTAATGAAGGAAGAGTATGCTTATGGACAACCCCCTGGA
GCTTTTCGTCTGATCCAGTTGACGCTGCTACCT

>c47288_g1_i1_len=272
ATCAAAAGTAAGTAAAGTAAAGTAACAATGATGAAAGAGACCAGAGCTACTCTGGCGAATTACTTCTACAACCTAAATGTTCAAACCATGCGTTCT
ATGAGAGAAGCCAGAACCTTGTGGACATCTAGGACCATATTAGATCTTTTGCCTGAAAGGTAATACATAGCTATGGAGTTCCTGCTGAGGGACAGG
CGCACCTGTCTCCCTCCGTGCCGGTGCTGTAGTGATTCGAGCTTTTTCAACACCTCAACCGGCGCTTGCGACCTTGTCCG

Bangkok contigs

>c39646_g1_i1_len=1084
ATTAATTGGTCGTAAGATCATTTTAATCAGATCATTTGCATCAGATGAGCTGGAGTGGACAGTCCCACTATCGCTGTCATCATTGTCATCATTGTC
TGGTTCGCTTTCGTCGCTAGATGATGGTGATGGTGAAGATGGAGGTGAAGGTGCTGGAAGGGTTGAAGGTGCCGAAATTGAAGGTTCAGCTTCATT
GGCATTGATTTGACAATTTGTGTTGGTTTCTTGTACAACCCGAGATAGACTATCGGCGACAACATTCTGTTTGCCGATACTTTATGTCGTAATTAT
ATTGTTCTAATCTTAACTTCATCTTAACGAGCCTGTCATTTGAAGTTTTCAAATTCAAGGCGAACGTTTGTGGTCGGTGTAAAGAGTAAACTTACG
ACCGAACAGATAAGGTCTAAAGTATTTGCACGCCCAATCTATGGCTAGGAGTTCTTTCTCTATGGCAGAGTAGTTCTCTTCTGTCCGAGTTAGGGT
GCGTGATGCAAAGGCTATAGGTTTGTCACTCCCAATGGGGCCTTGCGACAAAACCGCACCTAAAGCGTAATTTGAAGCATCTGTGGTTAGCACAAA
TGGCTTCGAGAAATCTGGGTACTGTAGCACGTGGCTACTGGTTATAAGATTTCTACAGCGCTTGAAAGCAGAGATAAATTCAGGTGTATGCTGAAT
TGTCTCTCCCTTCCGCAACTGTTGTGTTAGAGGTTTCGCGATTTTGGCGAAATCTCTAATAAATTTCCGGTAATATCCAATCATTCCAAGGAACCC
TTTTAGTTCCTTTTCATTATTGGGTATTGGCCATTCCTGGATAACCCGAATTTTCTCGGGATTGGGTTTTACTCCTTCGGGAGTAACCACGTGACC
AAGAAATGCCACTTCCTTCTGAAGGAATTCGCACTTATCAAGTTGGATCTTGAAATTGTGCTTTTTTAGCGTCTCTAGAACCCTTTTTAGATTCAC
GAGGTGCTCTTGTAAGCTGGTAGAATAGATGATCTGTAATATGGGGGATGGGATATCGATCATCGGTAGTCTTTAGGTTGAGCTTCCGATAGTCGA
TGACAAGTCGCTATTTTCTTGTGCCAGA

>c55859_g1_i1_len=395
AATAATTAAATATGGGGGAACATGCGCCGCTACAACACTCGTCGTTATGAACGCAGACGGCGCCGGTCTCGGCGCAAGCGGCTAAAACGTGTTGCG
CGTTCAACGCGGCAAACATCGCAAAAGCCAATAGTACAGTTTTGATTTGCATATTAACGGCGATTTTTTAAATTATCTTATTTAATAAATAGTTAT
GACGCCTACAACTCCCCGCCCGCGTTGACTCGCTGCACCTCGAGCAGTTCGTTGACGCCTTCCTCCGTGTGGCCGAACACGTCGAGCGGGTGGTCG
ATGACCAGCGGCGTGCCGCACGCGACGCACAAGTATCTGTACACCGAATGATCGTCGGGCGAAGGCACGTCGGCCTCCAAGTGGCAATATTGGCAA
ATTCGAGAATT

>c76297_g1_i1_len=529
AATACCTTTCGCAGATTGATTAAATGTTCTTGAAGCGATGTTGAGTATACGATAATATCGTCCATGTATACTAAACATACTTTTCCAATTAAGTCC
CTTAGGACATTGTCCATCACCCTTTGAAAAGTGGATGGCGCATTTTTAAGCCCAAAGGGCATTTTGAGAAATTCGTATTTCCCATGCTCAACCGTG
AAGGCGGTTTTACAAACGTCTCTGGGATGCACCTCAATTTGGTGGAATCCCGAGGCAAGATCAATCGTTGAGAAATAATTGCATCTTCCTAGTTTA
TCTAAAACTTCGGTGATGTTCGGTATTGGATATTTATCGTCGATTGTCTTTTCATTAAGTTTGCGGTAGTCGACAACTAACCGCCACTTCTTTTTG
CCAGAAGCGTCCATTTTCTTGGGTACTACCCAGATAGGGGACGACCATGGAGAGTTGGATGGCCGAATAATGCCTTGTCGAAGCAGGTCTCGTATT
TGAGATTGTACTTCTTCTTTCAGGCAATATGGATACCTGTAACTCTTGG

>c82294_g1_i1_len=206
TAGTTTTAACGTCACCGACATACTCGGTAATTTTGTAGTCGGCAGTTCTTTTGGGCGTGATCGTTGTCACTACGGGGGGCATGTCATCGCTTCGTA
AAAACTTATTACAATCAAAGCTCGTGCCGGAACGCGTGCACAGATCGTGCACGTTGCGTCGCAAGCTATTTAATTCGCGCTTTATTTGATCTCTAT
CATAGTCGTGTTGA

>c118388_g1_i1_len=208
TTTTTCCTTCTAACCATCTTTGGTACCTCTGAATAACTTCGATTTTGGAAGGGTTTGGGCTAATGCCGTCAGATCAAATTATGTATCCTCGGACTT
GTCCAGCTGAACTTTAAGGTTAGCATTGTTCAATGCCTGGATTATGGTATCAAGGTTTTTCAATGCTTCCTCGAAGGTTTTGCCAAATACGATTAC
ATTATCCATGTAAACG
>c70842_g1_i1_len=1639
GGCACAACCAGGCGGTCTTTCTCAAGAACACATGGTTGGCGCGCTTAGTTACTGTCGAGCACTTAACGACACGTTGGCAAACTAATAGTGGTCCAC
AACTTGAAACTCATCACTTGAAATACTTGAGGTTGGGGATTCCTCACCACTTTCAAGCGACTCGCTACTATTCACTGCGGAACTCTCACCCTCCCC
CTCCCTAAAGTGCAACAGAGAAGTAACAGAGTTATAGAACGTAATTATTGGCGTAATGTCCAACACCATATCACCATCCCATCTCCAATCCATATA
GGTTTGGTTGATGACGATGTTGACATTTATCACCGCTGTTGGATCCAAGATCTCTATCAACTGACAAAACTTTATCATAAACCAATTATCGCTCCG
AGGAGCTTGACTATTGAAAAGATTCATTACAGGACCAATGATCTTGATCTTCGCGCTCTCAGAAATAGCAACGTTTACTTCTTTAAGAGACTTGGG
AACAAAAAATGTTCCTGTTAGACACGAAGTGAACCGTAAAGTCGAACATTGTTATCTTCGGAAAAATAAGGACTTTTCAAAGATAGACCAATGTGCAC
CAATCCTATTGGTTCAACATTCCACGTCGGGGGTCTCGAAGCACATTGTATTTCACTCACGTTGTCCACTTTTGAATCATCGTGAGATACGCTGTA
ATTATCTTCAATAGTTAAATCGGTTCCATTGACTAAAACCTCGGCTGCCGCTGCATACGTTATCATATCCCTGTTAGCCATCTGAATTTCAAAAGG
CTTGTAAAATCCAGAGGTCTCTTGATGTTGGGGCACATATTTAGTTCTTATAATGGCCCGCACTTCATATAGGGTAACCTCCCTAACCATGTGGTA
ATCAAACACACTAGGTCCCGGAGGCGGTGGGGGGGGCGTCTCGTGTCCCTATTTGGGCCCCTGCAGGGTAACCTTATAGTGGACCCAGATGTCTCC
AAAGTACTGCTCCTCATCCTTATTAGAGGAACAAGCCAAATAGGCCAAAACCTCGCAAGGGGCTTTGTCTATCATAGGTATGTCCCCCGAATCTGA
GAGGGCATAGTACTTACGACTCTGTAACCGGGACGCAGGGAGAGTCAACTCCTTCTTCTGCCATACTGGGACCTGAAAGTTAGGAGTACAACTTTG
GACTTTGCTCTTACTAGGCACAGTCGTTGTGGGGTCCCAGTCGACTCCGACGACTAGAGAGCCATCTTTCATCGCCCCCACAGCGGGGCGATACTC

TAGTCTCACGGAATGCCAAATTATCTGAGAGAAACTACCGCTCAGAGTCTTTAGCCACGTCATTCGGGCCGACGTCGGCTGCAAGGCCTCATAATA
GGACGTGGAGGAACTTCCTTTGGACACTTTAATCTCGAACAGGAGCTCAGCTCTCTGGACGGTAATACGCCCATCAGCCGAAACCCCGCTCGGTCC
AGCGGCTCCTCCGCGTTTCTTATTACGCCTATTGCGCCGTTTCTTGGCAGAAGCGTTAGGTTGCGACTTAGCAACCTTCATCGCAGCTACTTGAGC
AGTTAAAGATGCAAGCTGTTTCTTCAACTGTTTACTCATTGTGTTAAAATAAAGCAATTAAACTCAGTTATATAAGGGCTAACCAAGGAAGCATAA
ATCACTT

>c28983_g2_i1_len=4421
CATCAGTAGTACCTCTGTGCTACAAAACCCACAATTCTAGGCGCTTTCGCATAGACCATACTCTTTAGCACTAAACCGCACCCACGACAGCATTTT
CAACAATGCACACCTACGAACTCTATAGTTCAAATCTAGAACTCCTCGAAGTTCGCCAACGGTTGGTCTCCGACCAACACCCATTTGCTCGTTTTG
GTACTAACCATCATGAGCTTTACTCCCCACCCGACTTCCTATCCCGATCATTTTGTCAAGCTTTCTGTCTTGACTTCAAATCCGACCCTAATCCTA
AGTTCGTGACTCTGACAAAAGCCGTAATCGCCAACGTTCCCGGTTTGTGTTACGTCGCCTCGCTTAACACCAATATCCCACAAACAATTGACGTCG
TTAAAAAATTAGGACTTTTCCCTCACACCGACTCCGCTAAAAATTTTAAGTTGCATGGAAAAATTACAGACTACGGATCACACCTCCATATGGAAC
GTCATCACGCTCCCCTTGCCGGTGTTGGTTACGTGGGATGGAAGTTTTCCTTGTACTCTTTCCTCCGCAGTACAATTCTCGGCACGGTGTGTTATT
TCACCGGAGCTTTCGTAGCATCGCAACCCGAATTTCGTGCTGCCGTCGATGCTCACGTTAACCCTCCGACTGTCGCCAGCAACCTCCCGCCGGTAC
CGCTTCAGACCCACGCTATGGAGGATTTGGTGAGACTTGCCTCCATCGAAGCCATACCGACCGTCGTTGCCCCGTCATTAGCCACCGCTGTTGACT
CCGTCGCCCCCGTCACCGACACTGTCGCCGACGTTGTTTCCACCGTCTCTTCCGCTCACGTCGTACCACCCATTTCGACCATCTCCAGTATTTCCC
CTGACTATCTTGACGTTAGTTCCGCCGCCGTCTCCGTTGTCGCCGCCGCCGAAATCGACCCCACTCCATCGACCGATCTGCTATCATATGCTTCTT
CAGCAATCAGCAGTTTGTATCGCCCTGTGGCTAGATTCGTCAACGGCCTCACCCGAGACCACCTCCCGTCTGCAACCTCCACTACTTTTGAGTCGG
TGCAAACAGGGCTATCCTCTTTCTCCGACTCTGTACGCAGTTCGTGCGACGGTTTGTATCGCCGAGCCGTTAACTCAGTCATTGGTGCTTATCATA
ACGTCGATGACTACCTGCGTACGTCCCCGTTCTTTACCGTTAAGTACCTTTTTTATTTATTTAAGTCTGCCGTTTTTTCGTTCGTTCGCGTGACGT
TTAATAACCCTGTGTCCACCCTGTTGGTTGCATACGTGTCCACCCCATTTATTCGTGCGTACACCAAGAAACATGGCTGGCCACACTTCCCCGTAG
TGAACGTCATGACCGTTTTGTCTGTGCCTGCTGTCGTTGGCCTTGTTTGCAAGGTTTTCGACACATTTTATTCGTCTTTACGTTTTACCACGATCA
CTTTTTGTGAAGCCGCCGGAATACCGCCACCATCAGCTCTCGCCGTTACCACGACAGGGCTTACGGCGACCGTGGGTACTTATGTACTCCGCTATA
CTCCTACCGGACTCGCAATAGGTGGTCGCATTCGCCGTTGGTTCATGCGGCGACCCACTTATTCTTGTGTTTTGCTTCCCCCTTCACACGGACTCA
CCACTATCTCGTCCCATCATCCGACCCTTTTCCTCGACTTGTCCACGATCGTAACTGAGAAGGAACTTGACGAGCAACCGGAGGAGACTCGCACGG
CTTTTGTCCAAAATAGACTTTATGCCTATCTTGGTCAATCGTCTAGAGTCGTCGTACTTAACCCCTTCTTCCATTCGTTCGGCATGAAAGTGCTGG
GTTCATATTGTTTGGAACCCTCTGCCGTGAACTCGACTGTACCCGATCGACTCACTTTCTGCCGTTCACTCACCGAACGTTTTGCGAAAATTGCTG
TCACCTTCCCAGATTACGCTTCCCTTCATGCCCGTTTGATGACTCACACCTGACTCCGCATAGGACGACGGCAGGACTATCATCAAAAGGGTGACT
ATACCCTCCCTACTGCTAAAACGTTTAATGCGTACGTTCACTTTCCTATCGATCCCGTCAACCGACCGCCTGCCAAACCACCAAAGTTACCGATTT
ACAGCACTAATGAAATCTTTTCTGCTGTATATCATGTTGACGAGGCGGCGGTTGACCCTGTGCCCGCCGAGACGATTGAACCTGAACCACCGCCTT
TACCGTTTTCGCCTGCCGCCGTCCATGGATCTGTTGGAAATCTACAGATCTACTATGACATGCTTTTCCCATCCACTCCCCTAACACTTTACGAGC
ACGAGGCTTACCTTGTGCAACTCTCTACCTTTGACATTTCCCTGCCTCGATGTACGTTAGTTCCCGGTAAAAACGCTTTTAACTTCCCACAATACG
ATTGCCTCCGCCCTAACTTAAAAACGCAAATGCCGCCGAAAAAACCTAATACTCAATTGGAAACTCTCCTCGGGTTCCAAAAGCGTAACGGCAACG
TGGCTCGACTACATTCCGGATTGAATCCTGAGATCTTGGCTAAACAAACCTATGATCACTTTATTCAGTCCGCAGTCGAACCTAGTAGCCTAAAGG
TTTTTGAACAATTTCATCGCTCCCCAATAGACATATCGAACCAAGACTTTGATGAGTGGTTACGGACTCAGGACAATCCTGATGTAGTTCGCAAGT
TGTCGCAGTCCGACTCACCCATCAACGAACGTTTAACCAACCTCTTTAACTTTATGATAAAAACCGCCGTCAAACCTGCTATGCAAGACAGCGACT
ACAATAAAGTGGCCTCCGTACAAACTATTGCCGCTGCTCTTAAGTCTGTCAACGCCCTCTATTGTCCGATTTTTATAAAGTTAAAACGACGTTGGT
TATCTGTAATGCGTTCTAAATATGTCTATTTAACCGACCTTTCCCTCGACGAGTTCGAGGAACTGCTGAATAAGAGATTCTCATCTATCGAGTATC
TGAATTCGACTCGGATTGAGAATGACATATCTAAGTACGATAAGTCGATGCGCGCTTGGACTTTGTTGTTCGAGACACGTATCTACAAGGCTCTGG
GTTTGCCCGATCATCTTGCTCTGATCTGGTACCAGAGTCACGTCTTATCTAGATATCTCGACTCGGTCAGCGGTTTCTCGACCACCGTCGCATATC
AACGTCGTTCCGGAGATGCATCAACCTTTTTCGGCAACACCCTCGTAAATCACGCTGTAGCCATGGCTATATATGCTGATTACGAACTACTCGGGG
GTGTTTTCGCAGGTGATGATAATCACCTTTGGGGTTTGAGCACTCTCCGCGACAACGTTGCTCCCATTTATTCAGATATATTTAATTTCGAAGCTA
AGACCCTCACCCATTTTCACACTTCGTATTTCTGCGGTAAATTTATTTTGGCGTCCGTCGACGGTTTTACAATTGTCCCTGATCCCATCAAATTGC
TAATGAAACTTGGTAGGCGCGACCTGCGTAATTGGGATCACGTAGCTCAGTACCAAGTTTCCTGCAAGGACAATATGAAACCCTTGCTAAACGCCT
TTATATACCGACAGTTATCCGAAGCTATATCAGAGCGTTATAATAGTTCTATCACTGATTTTTCCGGACTCTTCCATTCAATTAATTTTGTCGTTT
CTACTCAACATGAATTCTCTAAACTTTATTATACTCTTCCTGACGACCGTCTTTTACTCGATCCATCTCTCCCTTCCCTCGAAGTGTAAACCCCAC
AACAGACCCTTCTCGGCCCCTAAAGACGTTTGCGTGTTCTACTGTCCTGATCCTCATGGGATGGTGATACCGCAAGCGTTATATAACCTTTCTCTT
CCTTACTCTCTTCACGGTTGGTTTCCTGAGAAACTTTCCGCCCCCTTGGTCCCTATCCCTTACGAGGCTTTCGTCTACAGTTTTCGCCCTCTGCAC
ACTTTCATTGACTCTACAACCCTGACCGACACAATTGGGTGCGGACAGTACCGTGAGTATGTGAAACATGGTGCTAAATCAGGTTATTCTTATGAC
CTGTGGGCGAACGCTGTCGACGACTGCCCCGTCCCCATTTACCCTCACCAATGCCGGTATAACGGTTCCCATAATATCGTCCAATCCCTCTCCACC
TTTCCCTTAGGCTCCGTCGGTTGTCGCACTGCTCCTAACATTTCTTACTGTTTCTTTTAGTTTAAGTTATCTTAGAATAAGTCATCTTAGAATAAG
TTTGGTCGCCTCCCGACTTAACGGTCGACTTTGTCCACCGTAGGTGTGCCTGCACCAGTGTATCACGTGACTCGCCTTCAGCGTGTTCACCTCGCG
ATCCCCGTTTTGAACGTCCACGGGTTGTTTTGTTCACATTTGCGATGAGTTTCTTGTTCGTTCTTCTCATCTTCAACGTTTTGCAGTATCAACACT
TCACA

>c34693_g2_i2_len=880
CTCGTCCCTAACCTAAACACTCGTCTTTGAAGTGCACCGTGATAAGCCAACGTGCATTCGTTTTTGATTATTCCTTTCTTCGCGCGGGTGCTGCTT
TTGTCTAAGACTGGCGTACTTACGGTGTCAAATAGCTAGTTCGGAAAATTTTGAGATGGAACCTTTTCCTGCCTCTGTCTTTCTATCTTTCTCCAG
AGTCAGGTTTGATGTCAATGCACTTCTATCGTTGCCCATATGTTGGGTCGGGTTTGTTTTCTGTTTCCTAGGTTATTGACCGACTGGAGGTCGATC
GGAATCAATTTCAATTTGCTAATTTGCCAATAATTGAATCTGTAAATTATAAAACTACCGCGTCAGTGACAAGCGTCCAAGCTCGCATAGACTTGT
CATATTTCGATATGTCGTTTTCGATTCTCGTCGAATTGAGGTATTCAATCGAAGAGAATCGTTTGTTTAGTAGTTCTTCAAATTCGTCGAGGGATA
GGTCGGTGAGGTAGACATACTTCGACCGCATAACGGACAACCAACGGCGTTTCAACTTTATGAATATAGGGCAGTAAAGTGCATTGACGGATTTCA
AAGCGGCGGCGATGGTTTGTACTGAAGCCACTTTGTTATAATCACTATCCTGCATTGCCGGTTTCACTGCAGTTTTAATCATAAAGTTGAAAAGAT
TAGTTAGTCGCTCGTTAATACGTAAATCGGACTATGACAGTTTGCGCACGACGTCGGGATTGTCTTGCGTGCGCAACCAGAGGGAAAGAGCATGTC
GTAATATATTTGGAGGTTACCGACGGAACCATGGACGGCGGCAGGCGAGAAAGGGAGTGGAGGTGGCTCAGGTTCAATTTCCGGAATTGGAGTGGG
ATCATCGACAGCCTCG

>c34693_g2_i3_len=853
CTCGTCCCTAACCTAAACACTCGTCTTTGAAGTGCACCGTGATAAGCCAACGTGCATTCGTTTTTGATTATTCCTTTCTTCGCGCGGGTGCTGCTT
TTGTCTAAGACTGGCGTACTTACGGTGTCAAATAGCTAGTTCGGAAAATTTTGAGATGGAACCTTTTCCTGCCTCTGTCTTTCTATCTTTCTCCAG
AGTCAGGTTTGATGTCAATGCACTTCTATCGTTGCCCATATGTTGGGTCGGGTTTGTTTTCTGTTTCCTAGGTTATTGACCGACTGGAGGTCGATC
GGAATCAATTTCAATTTGCTAATTTGCCAATAATTGAATCTGTAAATTATAAAACTACCGCGTCAGTGACAAGCGTCCAAGCTCGCATAGACTTGT
CATATTTCGATATGTCGTTTTCGATTCTCGTCGAATTGAGGTATTCAATCGAAGAGAATCGTTTGTTTAGTAGTTCTTCAAATTCGTCGAGGGATA
GGTCGGTGAGGTAGACATACTTCGACCGCATAACGGACAACCAACGGCGTTTCAACTTTATGAATATAGGGCAGTAAAGTGCATTGACGGATTTCA
AAGCGGCGGCGATGGTTTGTACTGAAGCCACTTTGTTATAATCACTATCCTGCATTGCCGGTTTCACTGCAGTTTTAATCATAAAGTTGAAAAGAT
TAGTTAGTCGCTCGTTAATACGTAAATCGGACTATCGTCCAGATTGCAACCAGAGGGAAAGAGCATGTCGTAATATATTTGGAGGTTACCGACGGA
ACCATGGACGGCGGCAGGCGAGAAAGGGAGTGGAGGTGGCTCAGGTTCAATTTCCGGAATTGGAGTGGGATCATCGACAGCCTCG

>c36305_g1_i2_len=1763
GATCGATGCGTGGCCACATATGGTTGCAATTAAATGCTGTTCTATATTTTGTTTACATTGCATTTTTGAGGCAGATGGCCTTTACTGCAGTTTGGT
TACGTTAGGCGTGAGTTGATATGGATTAACAGTGGTTCTTAAATGGAATACCATTACAACTGTCATGCAGGTTGCAATAACATGCATAGGTCCGAT
TCCTGAGTCTGTTAATGGCTGTTGTTGCTGCTGCTGTTGTTGAATCACTATCTTCTATTTTCTTGCTGTAGGTATTCACTTATTCACTTTTAATCA
CTTAGCTGGTTAATTAGTGATGCACTTCTTGTGATTGCCTTTAGTTCAACCAGTAGTTGATAATAAATATACACTATCAGGCAAAATCCTATCAGA
GAAACTATTAACACAATTATCACGTTATTTGGTACTTTCATTGTCTTTTCTGTAGGGGTCACCATGTGTTCCGATTATGCTACCACATTCTTGATA
GTGTTTCTTGTGGAGATAGGGGAGATCCTCCCACTTTTCGACCCATTCTTTATGTTTCATCCATGGTCAACGAGGTAGAGTCAATGAAACAGTGCA
ATGGGCGGAAAGCGACGACGAATTCCTCATAGGGCATTGGGGACACAGGAACGGATAGTATCTTAGGAAACCATCCGTGGAGTGAATACGGAAGTG
AGAGATTATGGAGTGCGCGTGGAATAACCATACCGAATGCGTCGGGACAGTAAAACACACACAGGTCGTGTGGTGCGGGAAAAGGTGGGTTCCTCG
GCTTACACTTCAAGGGACGGGAGGGACGGATCGAGAAGGAGACGATCGTCAGGCAAAGAATAATACAGCTTCGAAAATTCATGTTGTGATGAGACT
ACGAAATTAATGGAGTGAAATAAGCCGGGAAATTCGGTGATTGGAGTGTTATAACGTTCGGAAATGGCTTCGGACAGTTGACGGTATATGTAGGCA
TTCATTAAGGGCTTCATGTTGTCTTTGCACGAGATTTGATACTGCGCCACGTGTTCCCAGTTACGAAGATCGCGTCTACCTAGTTTCATCAGTAAC
TTAATGGGGCCAGGAACAACAGTGAAGCCGTCAATGGAGGCGAGGATGAATTTACCGCAAAAATAAGAAGTTTCAAAGTGCGTTAAGGTTTTTGCT
TCAAAGTTAAAAATGTGCGAGTAAATGGGTGCAACGTTGTCCAGAAGAGAACTTAGACCCCACAGGTGATTGTCATCACCGGCAAAAACACCGCCG
AGCAATTCGTAGTCGGCGTAAATTGCCATTGCGACTGCATGATTGACGAGGGTGTTTCCAAAAAAGGTCGAAGCATCACCGGAACGGCGTTGGTAG
GCGACGGTAGTCGAAAAACCACTAACAGAATCGAGATACCGAGATAGGACATGACTCTGATACCAGATCAAGGCTAAATGATCTGGTAAGCCTAGA
GCCTTATAAATGCGCGTTTCAAACAATAGCGTTGTCGCGCACTGAAGCCTAAGCTTCCGACCCGAAGCACACCGAGTTAATCCGTAACCCGATGTT
ACGTTCCGCGAAGGTTTGAACTCTTCGGTGCGACAGTGTCGATGTGAAATTGTGTAGTGAATCTATCATCAGCCAAACTTGTCTAGCTGATGTTGT
GATCTCTCACTTGAATCAAACTTGGGAAGGGCCTAGTACCGCTATGATTAGGGGAGCGGCCTATGTTCCGTCTTCAGAAGATTGATTCAGGGTTGA
GATCCGATTTAAGCGTTACGTAATAAATGGACGCT

>c36305_g1_i3_len=1757
GTGCAAAGTGCTTATGCGCGCTGCACCGAGGCCATCGTTTGCGCCTATGCGGACCTGACCTATTCTGTTCTGGAAAATGTTATGTGCGTTTTATTG
CTTATGCTGATAGATGAGGGAAAGATGTTTTGGGTGAATGCGATTATAGCACACTGTCATGCAGGTTGCAATAACATGCATAGGTCCGATTCCTGA
GTCTGTTAATGGCTGTTGTTGCTGCTGCTGTTGTTGAATCACTATCTTCTATTTTCTTGCTGTAGGTATTCACTTATTCACTTTTAATCACTTAGC
TGGTTAATTAGTGATGCACTTCTTGTGATTGCCTTTAGTTCAACCAGTAGTTGATAATAAATATACACTATCAGGCAAAATCCTATCAGAGAAACT
ATTAACACAATTATCACGTTATTTGGTACTTTCATTGTCTTTACTGTCGGGGTCACCATGTGTTCCGATTATGCTACCACATTCTTGATCTTGTTT
CTTGTGGAGATAGGGGAGATCCTCCCACTTTTCGACCCATTCTTTATGTTTCATCCATGGTCAACGAGGTAGAGTCAATGAAACAGTGCAATGGGC
GGAAAGCGACGACGAATTCCTCATAGGGCATTGGGGACACAGGAACGGATAGTATCTTAGGAAACCATCCGTGGAGTGAATACGGAAGTGAGAGAT
TATGGAGTGCGCGTGGAATAACCATACCGAATGCGTCGGGACAGTAAAACACACACAGGTCGTGTGGTGCGGGAAAAGGTGGGTTCCTCGGCTTAC
ACTTCAAGGGACGGGAGGGACGGATCGAGAAGGAGACGATCGTCAGGCAAAGAATAATACAGCTTCGAAAATTCATGTTGTGATGAGACTACGAAA
TTAATGGAGTGAAATAAGCCGGGAAATTCGGTGATTGGAGTGTTATAACGTTCGGAAATGGCTTCGGACAGTTGACGGTATATGTAGGCATTCATT
AAGGGCTTCATGTTGTCTTTGCACGAGATTTGATACTGCGCCACGTGTTCCCAGTTACGAAGATCGCGTCTACCTAGTTTCATCAGTAACTTAATG
GGGCCAGGAACAACAGTGAAGCCGTCAATGGAGGCGAGGATGAATTTACCGCAAAAATAAGAAGTTTCAAAGTGCGTTAAGGTTTTTGCTTCAAAG
TTAAAAATGTGCGAGTAAATGGGTGCAACGTTGTCCAGAAGAGAACTTAGACCCCACAGGTGATTGTCATCACCGGCAAAAACACCGCCGAGCAAT
TCGTAGTCGGCGTAAATTGCCATTGCGACTGCATGATTGACGAGGGTGTTTCCAAAAAAGGTCGAAGCATCACCGGAACGGCGTTGGTAGGCGACG
GTAGTCGAAAAACCACTAACAGAATCGAGATACCGAGATAGGACATGACTCTGATACCAGATCAAGGCTAAATGATCTGGTAAGCCTAGAGCCTTA
TAAATGCGCGTTTCAAACAATAGCGTTGTCGCGCACTGAAGCCTAAGCTTCCGACCCGAAGCACACCGAGTTAATCCGTAACCCGATGTTACGTTC
CGCGAAGGTTTGAACTCTTCGGTGCGACAGTGTCGATGTGAAATTGTGTAGTGAATCTATCATCAGCCAAACTTGTCTAGCTGATGTTGTGATCTC
TCACTTGAATCAAACTTGGGAAGGGCCTAGTACCGCTATGATTAGGGGAGCGGCCTATGTTCCGTCTTCAGAAGATTGATTCAGGGTTGAGATCCG
ATTTAAGCGTTACGTAATAAATGGACGCT

>c36305_g1_i5_len=1256
TGTTTCTTGTGGAGATAGGGGAGATCCTCCCACTTTTCGACCCATTCTTTATGTTTCATCCATGGTCAACGAGGTAGAGTCAATGAACGAATTCCT
CATAGGGCATTGGGGACACAGGAACGGATAGTATCTTAGGAAACCATCCGTGGAGTGAATACGGAAGTGAGAGATTATGGAGTGCGCGTGGAATAA
CCATACCGAATGCGTCGGGACAGTAAAACACACACAGGTCGTGTGGTGCGGGAAAAGGTGGGTTCCTCGGCTTACACTTCAAGGGACGGGAGGGAC
GGATCGAGAAGGAGACGATCGTCAGGCAAAGAATAATACAGCTTCGAAAATTCATGTTGTGATGAGACTACGAAATTAATGGAGTGAAATAAGCCG
GGAAATTCGGTGATTGGAGTGTTATAACGTTCGGAAATGGCTTCGGACAGTTGACGGTATATGTAGGCATTCATTAAGGGCTTCATGTTGTCTTTG
CACGAGATTTGATACTGCGCCACGTGTTCCCAGTTACGAAGATCGCGTCTACCTAGTTTCATCAGTAACTTAATGGGGCCAGGAACAACAGTGAAG
CCGTCAATGGAGGCGAGGATGAATTTACCGCAAAAATAAGAAGTTTCAAAGTGCGTTAAGGTTTTTGCTTCAAAGTTAAAAATGTGCGAGTAAATG
GGTGCAACGTTGTCCAGAAGAGAACTTAGACCCCACAGGTGATTGTCATCACCGGCAAAAACACCGCCGAGCAATTCGTAGTCGGCGTAAATTGCC
ATTGCGACTGCATGATTGACGAGGGTGTTTCCAAAAAAGGTCGAAGCATCACCGGAACGGCGTTGGTAGGCGACGGTAGTCGAAAAACCACTAACA
GAATCGAGATACCGAGATAGGACATGACTCTGATACCAGATCAAGGCTAAATGATCTGGTAAGCCTAGAGCCTTATAAATGCGCGTTTCAAACAAT
AGCGTTGTCGCGCACTGAAGCCTAAGCTTCCGACCCGAAGCACACCGAGTTAATCCGTAACCCGATGTTACGTTCCGCGAAGGTTTGAACTCTTCG
GTGCGACAGTGTCGATGTGAAATTGTGTAGTGAATCTATCATCAGCCAAACTTGTCTAGCTGATGTTGTGATCTCTCACTTGAATCAAACTTGGGA
AGGGCCTAGTACCGCTATGATTAGGGGAGCGGCCTATGTTCCGTCTTCAGAAGATTGATTCAGGGTTGAGATCCGATTTAAGCGTTACGTAATAAA
TGGACGCT

>c46271_g1_i1_len=538
TGATTTGTGCTAGCCGAGGTGGTCATAAGCTTTCCTTCTGCTTAGACCCAACTCCACAACAACGCCCCTAACTCTTTACGAACACGAAGCATTCCT
TGTCTAACTCGACGTTCGACATATCCATGCCGAAATGTACCCTTGTTCCTGGCTAGGGTGCATAGGTACTCTTTTCTCCAGTATGATTGCCTCCGA
CCCAACCCAAAAAAAAAACCCAAATGCCACCGAAAAAGCCGAACACCCAACTGGAGACTTTACTTGGTTTTCAAAAACGTAATGGGAACGTGGCCG
CACTGCACTCCGGAATGAATCCAGAAATGTTAGCTAAGCAAACTTACGAACATTTTATTCATTCCGCAGTCGAGCCCCGTAACCTAAACGTTTTCA
ATGAATTCCGCAAGTCGCCGATAGACATATCCGTTGAAGACTTCGACGAGTGGGTGCGTACTCAGGACAATCCGGACATCGTCCGCAAATTTTCGC
AGTTTGACGTTCCGATTAACTAGCGGCTAACAAACCTATTCAACTTACAATCCTTGAG

>c36387_g1_i1_len=6125
ATGTTAACTTTCACAAGTTAGGGCGAAAGTTTTTCGGACCGGCCCACAATTAAAATACCGGTAAGTGAGATCAAAGCGACGAATTTACGAGAGAAA
TAAAAACAAAATACAAAAACAGACACAAGATTGGTTCAGGGCGACACGTTACCTGTCTACATACGTGACGTGACATTGTGAACGCAATATTGTTTC
TTCCGGATTTCTTTCGAAGGATCAATGAGTGCTTCTGAGGGTGCTTATATTGGAACAGAAGACACGGGCTTCACACTAGTTGAGAAGTCTGATCCT
TTTATGCATCCCACTAGCACTGAAAAATCAGAACGTGACGTGAGAGGAGTGAGGCAAAACTCGGCTTCGGGAACAGTCATAACAGCGCACGACCAG
GTGAAAGAAATAGTTAGCTCGGAAGTACACATAACACCTTCCGCGAACATGTCTGCAAGTGAAGAAGTTGCACCGGGTGGCGCTACCCGGCGTCAG
GATACACCAGCGAGTTCGTCAGATAGTGGTTATGGAGGAAGCGCTCTAGCATTGGCGTCCATTTGGAACCGTCCGATACCTGAAGTCGCACCCAGA
GGCGCAGGGCATTGGAGGAGCCATGGCGCTGCGCGTACTTACGCTCTTATGCGTGACATGCTCAGTCCGCAGTATATCGCTGACCCCCTATATGTC
ATAGGGTACAAGTGGACGCTCGCTGACGTAGAACATAACGTCGAACGTATACAGGTGGCCGGGCATATTGTATCGGTATCTGGAACTATGCGAGCG
GGAACGAACCCCCCTGAGCTGTCAGACAGAGGAGCAAAACTGTGGTCGAATGCAGTACTTACGCCAGTAGATGCCTCTATGCAGATACGTAATTTG

ATACCTACAGGGGGAAGCGTCAATCTAAGTGCGATGACCCAACTTGTATCAAAATATCACGGTGATCTGCATACTTTAGCCAGAGATGAAGCACCC
CTGCTTCGAATGTTGTTAATGTTCCTGACCTTTTTGCCCGCTGGATCACCCGGGTACGCATCGAACACAATCAAGATGCTCAGATTAGGCGCTTAC
CAGGGAAAGTGCACAAAGACACTTCACCCCTATGACCAGAGAGGCGTTTTGGACAATACCATCCAGATTCCCGCCGGCTGTGCCGAATGCGACGTG
CGCATCGTGCCCCTCAGCACTTACGTAAATGCGTTATGTGCCAAACCCAGTAACAACGACAGGTTAGCTGGGTTCAACATCCTTGAGGCTGAGATC
ATCGCTATCGAGAGTCACAACCTTGAACAGAATTGGTTTATGGCTTTTTTGATCGCCCACACTTCCACCATTTGGTGGAATTGCGCGGCTACGGAA
GAGTACGATTTCTCTTCAGATCCGAAGTCCACCTCGGAATCAAAATCCAAATTTGTCATCAAAACCATGACACGTGCCAGCTGTGTCTATGTTCCC
GGCGAATGGAAAAAGATCGCCATAGTCGTTGTCGACCAGGACTATAGTATGTCAACCAGATTCAGTGTGCCGCACCTTGGAGATGCGATCAAATTC
GAGACCAAGATCGCTGATTTTACAAAGAAGGTGTTTACGTATCTGGGGCTCACTGAGAACGGAGTGGCACCAGACCCCCATACAATGCTGATGGCA
GTAAGAGAAATGTGCAAGCGAACTGCTCTGTCAGGAGACATGGGAACCGTGCTATCGATGGTTCAAGAACTAGCTTTCGCTGTCCCATTAGGTGGC
GCGGTGAGGACAGACGGTGTGTTCGGAGCGTCCGGTGTAGGAAGATTTGACATTAATGCGGCCGACTATTTGCTTGATAAAACAAGAACGACGGAT
CTTGCAAAGGAGTGGGACGCTATGTCCGGATGGTTAACTGGGTACGGTGACTGGTGTGTCACACCCACAGGTAAGCTTATGGACGCACTCTTCCAG
TGCGATAACAAATGGACGGAGGCGACAGAGGAGAAGAACTCGAGGTTTGCATTGTATAGTATTGTTAAGCGTGTGACTGGTTATGGTGTAACGCAA
ACCTCGGGTCCAATGCGTGTGCTGCGAGAGTGTGGTTGTTTCAGAGATCGAGTATTGTGCGAGTACGAATGGAACACTTATACCGATCTATTCAAC
GCGCTTCAGTCGGGAGCGTCTTTGATGGCTGGTGTGAGTGGATGGATGATGTGTGAGATTGGTTACAATATCATGGATTTGAACGGATTGTATGCG
TCATCAGGTCGTGGTGTGGTAGATAACTACAGTGCCATCTTGTTGAACTCGATAACATCCGGGTTCATTAGTAACACTAGCGGACGCACAAATAGG
TACAGTCTCTCTACAGGTTTTATCTCCGCAGTTAAATCATACACAGGGATGGCTACTGACGCAGAGGTGAAGATGTGGCCTACAGTTCATGTGCCC
TGGTGGTTCATACACATGTGTGTGGAAAAGTTTGGCCAGTGGACGCCGCTCAAAGAGAAGCCACTACGTCCTTATTCATTGTATGATATCACCGAT
AGGGATGCCGCAGAAGACATTGACACTATGGGCTGTGCTCTGGCGTTTAAGTTCACCTCTAAGGATGTTGATTGGCGTGAGTTTCCCGCCGCTTGT
GGAATGAGAGCTTATAATGTGGCAGCTGCCGGTTGGGATAAAGCGCATCCTCAAATATACCTGCCGGGAAGATGGAATGGTTTCCCTAAAACGGGA
AGGATCGGCTGGATGCCGTACGTGTTCTTAGACGACATGAAACACAATGGCGCTCCAGGTCCGTGGAGTTGGGTAATGTGTGAACCACAAGCCTTA
CCGATGCCTCGTCTTCGACAGGTACACAACCCAGACAGGATCATCGAGACATACATCGTCGGATTGACCAACATACCTGGTGCATCAGTGTTAAAT
GTCTCGGTGAGCATGGATTACCCCGATCCTCCTCCGGGTTGGATGCGCGATGTTTGGGAATACTTGAAAAGTGAGGCGCGCGTTGCCACACCGCAT
TTACTGCGTGGTGATGTGCTCGGAGCCATTGGATCTGTGGGCATTGACACTGTGGTTAAAGCCGTGTCTGCGCTTTCGGACAAGCTCGAAGGTAGT
GGCAAGCGGAAATTCGTTGCCGGTCTGACGGATGGGGACCAGCTCCGACATGTGGCTGGCGAAGCGGAGGCTAGAGATTGAATTTTGTCATCTGTG
CGCTTCCAGGAGAGCAAAAGAACGCGCTTGTTCTTCTTAAGCAGTGTTACACGCAGGCTGGCTGACAGCATTCCAAGATACACTGCGTACAAGATG
TCTGGTGATGCAAGTGTGTACTATCCTGCGTGTGAGTATACACTGGCTGATGTTGAGAGGGTTATGAATGTGGTGTCTGTGATACAATCTGAGTGT
GAGAGTGTTAGCGTGACACGCGTAGAAATAGATCATAAATATTTTAGTGTGGTGTCAGCGACACCTTTAGTATTAGACACATTCCGTTCAAGTCTA
GAGGGATGGCGTAGTATTATCGGACTTTGCGGTCCTAGATCTAATGTAGTTAAGGTGGAAGAAAAAATTCAAAAATTCCGACGTTGCAATCGCCAG
TGGTGTACTAGCAGCCCTGTCGATATGTTGACTGAGGCCATGAGCATGCTCAAAAAATATATTTACAGTAAGGGAGTAGATAATAAGACGTCTTCA
GATTGCAATGGTATCGATTTGAGTGAAATAATGAATGCTAGATGTGTGGTAAGTTGTGATGCGTTTGAGTGTGGTTCAAAGTTTCCAGCACACCCA
ATGTTGGGTGGTTGCATCCTCAACATTTGCACCTTATGTTCAAGCACCAAGAAGGCTCTCGTTCAAAAGGTGAGTGACTATGCAAGTGACCACGGG
CTGATGCCAAATTATAAGTATCACTCATTGTTGTCTTTGCGTAGATGCAGAATGTTCACAGGGGATGCGGATATAGGATCAGTCTTCACGTATGGC
CTCTCCGCATACTTCTCGTTGATGACAGACCCATTGGCACAACACTTATCTTCTCTAATAAATGATCGCATTTCACATATTATATCAAGAGTCCCT
GTCCTTTTTGTCGATGACGCTATCCGCCAGTATTTGTCAGCTGTGGACAAAGTGGTGTTCACGTCTTGTAGCGGGTCAACCCCGATATGTTACGTG
TTATCCTGTTTCCACGGACTTTCAACCATCGGAGGACCGCGCTCCTGGAGTACCGGGGCTCTATATGATAGTATAAGCGAGTGGGTTTCTGAGAAA
GATGAAACAAACAGACATCCGGATGCGATGAAATTAGAGGAGGAATTGGTTGTAAAATGGGTTGGCGCTTGGTGTTCGGCCAAGCTCGGCACAACT
AGGGTGAGCTTCAAAGTTTTCTGTGCGGACTTGAACAAGTGGGCCACCTCTGGTGGTGGGCCACGTAGCAACTTTGAAGTCGGTGGTGAGGAGTTC
AACTTAAAGACGAAATGGGCGTGGGGATTTGAACAGTTGTTGAACGGTCGTGATGTGTATGAGGAGGCGTGTGAGATGCCACGAATAGCGAGGGTG
GCGCTAAAAGAAGAAGCCAAGACGCGGACAGTCATCACCACTCCGATGGCGAGCTACCTGCGTCAGTGTTACTTGTTGTACGTTTTGGGTGACCCC
ATATTCTTAGACAGCACCATTGGATCCACTGAACATGTACAAACACTGGCTCCATACAGATATGAATACTACATATGTGTTGATGCCTCCAAATTT
GACCATTGTGTTTCTAAACGTTTCATACTGTTCTTGTTGAGAACACTGAAGAACAGTTTGGCACCTTACCATCTTGATCTCGAACTTGATCGCCTG
ATTGATGCCGAGATTGAAGAGATCGAGTCACTTGTGGTTGAATTTGATAACAAAACACTTAAGTATGAAGGTGGACTATTAAGTGGGTGGAAATGG
ACGTCGTTAATAGGATCGATGAAGTCAGATCTTTTGTGTGCATACATAAATCAGAGGCTGAATGTCGACATGAAAAAGATTGTACAAGGAGATGAT
ATAATCATGATGTCGAATAGGTACTATAACGTCGCGACGATATGCAGCATATGTGAAGAATTTGGGATAACCACAAACCCTCTCAAGACAACAGTT
TCGGATGTGGGCGAGTTTCTGAAATACAGATATGGTCCGCGTATAATATCAGCCTATCCCGCTAGAACAGTAAGATCAATATATCTAGCTAATCCT
TGGCTCGACAGCAGTTCGAAGAGTTCCATCGCAACAGTACACTCGAAGTGGATGGCTCTGGCGAGCAGAATGGCTATGTGTTTGAATGACACCTCT
ATCGTGCAAACGTGCATAGAGTATGCACGGGATGATTCGGTCAGTTGGAGTGGAGGTAGCCTCAGAAAAAAACAGTATGACAGGTTGCTGAAAACG
CCGACAAATGCCGGAGGTCTGGGCTACGTCGAATTCATGGATGTGTCCTCGGACCGCGAAATCACCAGCATACGTGAACCTGAACATGGCATGCCA
TATGAACGCCAATTTCTTTCACTACTGGGTGTTGTTCCTGCTGAAGCCCCCAAACGCGTGGTTTTAGATGTGAAAACATCGTATGTAACATTCTTG
GATAATGCGGCTAGTGCGCGTTTCACCGGCTTATCAACAGTATCGGGTTTAACCATTAGGAAGGATTGCAATGCATTCAAGACGATTCTTGCTTTG
TTTCTGCACTACCGCAACTCCGAAGTGGTCAGACGTGTATACGATAATTCAGTGGGGCTAGTCGATAAAAGCGTGTTTAACGATGGTATGTATCCG
ATGTATATGAGGAAGACGCGTAATTTTATGAGTAGGATTAAGCACATCTTGTTTCCGGATAACATATCTGTGCCAAATAGCCTATTTTTGGACAAC
AGGTACAGTACATCCTTAACAAGATACATGGTTAGATACACACGGGCTCTTCTGACATCGTGCAGGCGATTGACCTTGTCACGCACTAAATCACTT
GCCATGTCAGTTGCTAGGGAGTACTTGTGTTATCGTACAGTATTGCATAGTGCGTGAGTCTCACTGGACACGGCCACCCAGTGAGACGATGTCCGG
CCAAACCTTCGACCCCGAACGGTAAGTGGTGCACGGGGATAAATGCACCCGCCTAAAGAGCAGCACTTAGGTGCGGC

>c36400_g1_i1_len=10659
CTTGGCTACACCACAGTTTTGGTTACGCTTATACTTTCAAAGCTTAAGTTGTTTTTAATTTTTGCCGAGAGACCGTGAGGTTAAATCCGGCAAGGA
ATGAAGAGGAAGGATTTGGAGGCGCGAGGAAAGGCGCCTGGAAGAGATTCTTCCACCCCGTTCGGGGGAAGAGAAGGTAGGAGAAAGGACAAGGAT
AAGGGGGGGGAGTCTCCCTCTAATCGGCAGGTGACCCTGAAAACACCCACCCAGAGTGGGCGTAGGACAGGGAAAAGACAGCGTGTTGGTCTGCTA
GGAAGGCTGGGAGTTGGATGGGGATCCTTCTTACAGGAGGACATCGTCCAAGCCCTCATTCACATGGCATTAGTGCTTCATGCCCTGTTTGCCTCC
ATCGACCGGCGAATTCGCTCATTATCTCGACGTGTCACTGCTCTTGAATCTCGACGAACCACAGGAAATCCTATGACGCTAGCTTTCATCTTGGGC
TTTCTTACAGTATTATGTGGATGCGTGGTGATTGACATGCAAGTATCCACCGCTAAGGGCACGGAAATTTTTGAAGGAAAGACGAACCGGACAGAC
TACTTGCATCTGTTCAAGTTACCCGCTGATGGTTGCTGGAACGGCATTCTTGTCACAAAGAAGTGCCCAAAAGTGACTGACTTGGCAAAGGACCTT
GAGTCGACGGATTGCGGATCAACGTGGACTGAGTTCACATTGCGGTATCGGAGATGCGTCGTGAAGAAACGGGAAAAGCGATCCCGTGAACCCCCG
AAAGCGGATCTGCTAGCTGAGATGGAGATCATCGCGTTCAAGACCATTCGCGAAAATAAGACAATTTTCATCGTGGCCCTACTGTGTGTGGCAATC
GCGAAGCGTTGGCCCACCTGGGTTGTGATTCTTCTGGCAGTTGGCACTTGGACCACAGTAAAGGGAGAGTTCGTTGAACCGTTATACACGCTGAAA
GCTGAACAGATGACCATGCTACAGACCATCGTGAGACCGGAAGAAGGGTACGTCGTGGCCACGCCAAACGGTCTTCTCGAGTTTAAAACAGGACCG
GCGGAGATCTATGGGGGGCAATGGCTGCGCGAGTTACTCGCAGATTGCCACGTCAACGCATCCTATTCGACTGACGTGTGCCCCGGTGGATCACAG
CTCAACATGGCTGACATTATGGCCAAGGAACGCGTATGCTCGACTCAACCATACAACCGCGGTTGGGGCACTGGGTGCTTCAAGTGGGGGATTGGA
TTCGTTGGAACGTGCGTCGAACTCCATTGCGACAGAGGATTCAACGTTTCTTCTATAGCTAGGTCAGCCATTGTTATGAACGTTACGGCTAGCTTC
CACTCCGTTAGCGACACGCAACAGATGGTCGGCGACATCCCGCTCACATTTAGATTTGCGAAACTCGGAAACGCGGCGATGACCTGCAGGTTGGAG
TCTGAGCAGTTGCTGCTTGATTACTATCACGTTACGGGGAGCTCTCACGAGGGGCTGTTTTTGCGGTCTCAAGTTGACAGTTGGCCTGGCGTGCAC
TCTACCGCTAGTGGTCGGCACGGCATGGAGAAAGTTGTTGTGTGGGGAGATGCCAGGTCCAATGAGATTCTAGTAAAAAACGTAATTGAACCTTCG
TTGTCCTGGGAGGACGCGATCGCCACCCATGATGGATTCCGTGACATCTCTTTTGTTTGTCAGATCATGCTCGACAAACTAGTCAGTGGAGCTTTT
CGAGACTGTCCAGGCCCAAAAACATCCACATTTTCCCAAGACGGCTTCGGGTATAGTGGAGTGGTAATAACTACCCTCACCGCCTCTTCCAACGAG
ACCTGTTCGCTTAGTTTAACATGCCACGGTTGTCTGCTGCAATCCACAAAAATGGTTTTTCTCGCAGGGAAGACCACTTCTCGGGCATTTGTCAAG

TGCGGGAACCATACGAGCACCTTGCTCGTTGGGAGCACTTCAGTTTCCATTGAGTGCGCCCTTAACCCAATCTCGCAGGGATGGCGCCTGGCTCGA
CACGTGGTGGATCGGTATCGACGGTTTGGTGTGTCTGGTGTTGCGGGAGTGTGGCAGGATTTGGTTGGAAAGTTTTCGGTTGGTGCCTTCTTCTCC
AACACAACCCTTCTGGTCATTTTCGTCTTCGCTGCCTTGATTGACAAACGCATAGCCTTTCTATTGGTTTTAGGGGGGTGCTTTTACTATGTGCGG
GCTGATCTGGGGTGTGGAATTGACACCACGAGAAAAACCATCTCGTGTGGCAGCGGCGCTTTTGTATGGAAACACTTGGGCGTTGGCATTTCCAAT
GATCATGCTGTGGAACTTGAAGATTACTCGTTCACTGACTTGTACATAAAGGATATGTTTAGTTGGACCACGAAACCCTGCTTGATTTGCGAGGAT
GCTTTGCAGTGCGTAGCCCTGCGCAGGGCTGCTTTCTCTGCTGTTGGGTCGATGGGTCCTGAACAGGTGTATGTGAATGACACGTTGGCGCGCACA
TTCACGTTCTCCGAAACACCAAAGAGAACCATTTCCGTCACCATTAACCTGATTCAGTACAAGTTCTCATCTTATGTTGCACATGGACGCGCTGAG
GGTGATCTGGGGCATCTACCAACAATGTTCGGAAGCTATCCCGAAAAAGAGGCTGACAAGGTTATCCGGATTGTTGCATCGCGGCCCGATATTCGC
AGACTTTGCGGAAAAGCTGTCTCCTTCCAATTCAAGTTCACTGGCTTTCGGCGAGGCCTGTATGGTTCAAACGTTCAGGTTGAAGTCTCAAAGAAC
TCATCGACCGAGTGTCCCACGTACCTGGCGGGCGTCGTTGTGAAGAATGGCCGAACTGTCATCACTGACGGCATGTTTTGGATGGAAAGCACAGTG
TTGGATGGAGTTGCCCAGATCACATCACTCGAGATGCGGCAATCACACCGCTGTGTTTGGCCCAGGGAATACACCCCAGACACGCTCAATGACCCT
TCAGACCAAGCTTTATTTATTCCACCTGCTTGGGGGGGGCCAATTTCGCGAGTGAACCACATAATTGGGTACAAGACACAAACTGACTTTCCGTGG
AACGTTAGTGACATCACCCTCATAGAGGGCCCAGCTCCTGGCACAAAGGTCAAGGTGGATTCTAGATGTCATGGGCGAATGCACGCACATGTAATT
GGACCGAACGACACGGAATCTTGGTGTTGCCAGTCATGCACAAGAATAGTCCACTTTCGAGTCGGTGACCTCCTATATTATCCCATGGAGATCCAA
CTTGGAACCATGTCTGAAGCGTCAGAGCCGAATTCTAAAATATTTGAGGAACCGATCGGCGAGGAACCAGAACCAACGGTTGACGACATATTGAAG
AGATACGGGAAGGCAAACGCGCAATCGGATTTCCGGAAGGCGAGCCCACGTGCAGGAGTTGGGTTCGATTGTTCCCTCCTAAATCTCCTGTGCCTC
GCTATCTCATTGCAGCTTATCGGGGCAAAAACCAGAACATCGACGCTAACTCGCCTATTTCTGACGATTCTAGCTATGGCGGTCTTTGGACTCCCC
AATTTGTTTTCATCTGTGGGGCTATCTGCTTGGGTGTTGCTAGTGGCGTCCTCATCATCGCAACCGCAAGATCTATTGATGAATCTCTGGATCATA
CTACAGACTGGGAGCTCAGCAGTCCTGCTGTTGGGGTACATGATCAGGAAGAAACTCTCAATAGTGCTAGGGGTCCACCATCTGGTGACTCTGATG
TGCGTACAGTTCCTGTTCTCCGCCGTCGATCGCTATCAGAAATATCTCTACGGTCTCCTAGAGCTCATGGCTTCGGTGGTCCTGCTGAGCGCCTAC
AAGAGCGTCTTGCAGGCTCTCCCTCCAGAGGTTCTATGTTTCAGCCTGATAATGGGATGGAAGACGGCCCTGTCTCTCGCTACCGTTGTGTTTCTG
ATCTTCAGTTTGAACGCTATGTACAAGTACGCATGTCAATATCACAATCCGAGGAACGGCTACAGAGACTCTGGCGCGAATTTGTGGTTTTGGACT
GTATCCTTGGCGAGCGCTGGGGGAATCTGGGCGGCGGAGAAAGCTCATCAACCAACGGTGGCAGCTGTCCTAGCCTTTACAATGATAGTGCTCTTT
CTCTACATGGAACAGACGAACGTCAGTATGGAGCTTGAGTTCATCTCAGCTGGAGAAACCCCAGAAGGAGTGTCAACCGAAAATGATGACGGAATA
AATATTCCTGATTTAAGAGGGCGCTATGGAGAGGATGGGATCGTCGTGGGGGCGGCTTCATCGTCTGGCCACCTACCTGAGCTAGTATTCGTGTTC
CTCCTAGGCTTTGCCGTGACGTCCACTTCATACTTTTTAGGGGCACTCTACCTGCTCATCGCAACCAGCACTAACCTTCCCGTGACCATAATACGG
ATGCTGCGCATGAAGCTCACAGCTAACAACCGATCTGATGATCTCTTGGGACTCGGCGGACCTATTGAAACTGATCTCCAGACCAGCTTTCAAGAC
ATTCCCAATGGAGTTTATCGCATCGTTGTTCGCAGCTTGTTTGGCGACCGGCAGCGGGGGGCTGGCTTTTCGAAAAATGGGGTATTTCACACTCTC
ATGCACGTCACTCGAGGTGAACCAGTAAAATGGCGTGGGCGAGCTGTTGTTCCTCACTCCGGTAGTGCCCTGCGCGACGTGGTGTCGTATGGTGGA
CCTTGGCAGTTGGACACCCCCACTACCACGGATGACCTCGTGCTGATGGCTTGCAAACCGGACAAGACCATTGAGTACCATCGGTACAGGCCTGGA
GTCATGAGTATTGACGGTGAACCTGTCATGTTCATTAGTGATGATTTCGGAAAAGGATCTTCTGGCTCACCGTTCTTCATTAATGGCGAGCCCGTT
GGGTTCTATGGTTTTGGGTTCTATGTGAACGGTATTTATCGCTCAACAGTGGCAGGTGGTAAACCATCCGATGTCACGGAAAACCTGGACTGTGAT
TCGACCAGGCGGTTCGTGACATGGCATCCAGGAAAAGGAAAGACGCGCAAGGTTATTGTCGAAGAAACCAAGAAGAACTATGATTCCAACCAACGG
ACTGTGATCTTAACACCCACCCGGGTGGTCATGGCTGAGGTCGTTGAGGCCTTGAAGAACTCAGGTATGAAGAGTGACAAGAACCTGTCGTACTGC
ACCAGGAACCTAATAACGGTTGCATGTCACGCAACGTTCACGAAATTTGTCCTTAGCCATGGCGCCAAGAAAGTGCGGGTTGCCATGATCATCATG
GATGAGTGCCATTTCATGGATCCTATGTCCATTGCCGCGCGCGGGGTCATGGAATATCTCCACAGCCAAGGAACAAAATTGATTTACCTTAGTGCA
ACACCACCCGGCCACGCGCCTGATGTAGGCTCGAATTACGCAATAAGTGACCAATCCATTAGTTTCCCAACCTGGTTGAGTCCATCGTGGATTGGA
AACGTTCAAAAATCTGTGGGGGCGAAGAAAACCATCTTATTCGTCCCATCGCACAACCAAGCTAACACCCTGGCTAGTGCTATTCCTGGATCTGTT
CCATTGCATCGAGCAAACTTTTCCACCAACTATGCACAAGCAGGTGATGCTGCCACCGCCCTGGTAATCTCCACCGACATCTCAGAAATGGGAGCG
AATTTGGGCGTCGATCTGGTTATAGACACTCGAAGAGCTTTGCGACCCCTTGTGGATTCTGCCACACGAGTGAAGCTGGTTGAAACGAACATAACC
ACATCATCCATGATCCAGAGGAGAGGGAGGACGGGTAGACGGGAACCAGGAACGTATGTGTATCCCATTGATAGTCAAACGGAAGAAAATCCAGTG
TCATGGGTGTGTTGGCCTGAAGCCCAAATGATCCTGGACCAGCTCGGCATGACTTTTATGCTAGAGGAAGCCGCATACAGCCAACCTCCGGGGAGA
TTCACCCTGGTGGGAGAGGATAGAATGCGATTTTCAAAGCTTATGGATCGGGATGACATTCCCATCTGGTTAGCGTGGCACTGGGCAGAAGCTGGA
GACCGACGGCACTCAGCCCTGTTTCAGGGCGAAGGAACCGGAAAGATCATCGAAAACCGATTTGGCAAACAAGAGTACCGACCACAATACGTTGAT
GACCGCTTTGAGAGCATTGAGTGGGAGACGCGAAAGGTATCCATTGATTTCTATATGAACTGCCGTGGAGGGCCTAGCCTCTATGAGTTTTTCACA
TTGATTGATTGGACCGATATATGGAGGAGAACGACATCTGCTCTCTGGGATTTGAGTGACGTCATGAATGGAGAGCTGCGTGACCGGTCCACAACT
GAGCGCTCTCTTACCGTGCTAATGGCCTTTGTCTTGGGCGTCTCAATTGTGCTCTCCTGTTTCATCGCCGTCTGGGCATTGTGTTTCCTATTCTCC
TTGTTTAGGCCGCGAAAGGTTACTTATGAACAAATGCCATCTAGTGACCCACTTGGCGGTGGTGTCTTGGTGTCAACCCCTAGCCTCCTTTATTAT
ATGGGAGTCCCCTTGGGGTTCTGCGTCGTCATATCACTGGCTATGTTCTTGGTGTACCCAGTACTGTACAAGTCAATTGGAAATCGCAGTTACATG
GACAGCGACTTGGTAAAGTGGGTCGTCTTAGGGTCATGTCTGATCTGCGGAGTGCTGGCTTGGGAGATGCGAATGTTCCCGAACATACGTGGCGAC
CTCATGGAGCTCGTGAAAGCTGCTAAGGAGCCCGAGGTGGTCGTGAATTCCGGGCCATTTTTCCCAGGTTGGGAAATGGCGCGGGGAAGGGGAGCG
ACAATGTTGGACTCTCTGCAGGTATTCTTCTTCATAACAGTGTTGTCGACAAAATTCTTGTACTGGTTCCAGGAGAACTGGACTGCACGAATGTAC
GCCATGAAACATCCGGAGATGGTTAGCTCGATTGGTGGATTCAGATTTGATGAAATTCCTTTCAGGGCTGTCCTTCCCTCTGGCTTTGCCATTGTT
GCGATTGCTAGCCTCTCAAGCGTGGTGGTGGGTTTTCTGGCAGCTAGTGTGTTCATGGCCATCATGTACTGTCAAAACAAGTGGAATGCCACCCCA
AAGATCTTGACAGCATTGGACGCCCGCGACCAGAGACATGATCGTCCCACTGAAATCACTAGTAGAGTGCCACTCGAAAACACTCGGTCCATCATG
TATGCCTTTTGCCTGATCTTTAGCCTGTTTTGGGCTTTCTGCACACGGTCCCCCGGAGATTTTCTCCGAGGGTCATTGGTGGTCGGGGCTAGCATG
TGGCAAATTCTCCATCCCCGGTCAAAGATCCATGATGTCATGGATTTTGGCTCCATGGTGTCTGCCATAGGGTTGCTAGAAATGAACTACCTTTTT
TATAGGTTCATGCACATTGCCGCTAGAGCCTTGGGCGCGGTAGCCCCCTTCAACCAGTTTCGAGCTCTAGAAAAGTCAACAACGATTGGTCTGGGA
ATGAAATGGAAGATGACCCTGAATGCTCTTGATGGAGATGCATTCACAAAATACAAATCACGCGGGGTAAATGAGACGGAGCGGGGTGATTATGTC
TCTCGTGGCGGCCTCAAATTGAATGAAATCATTTCAAAGTACGAATGGAGACCGAGTGGTCGAGTGGTGGATTTGGGATGTGGGCGCGGTGGCTGG
AGCCAGCGAGCTGTGATGGAGGAAACGGTGTCCTCCGCTCTCGGGTTCACAATAGGTGGGGCGGAAAAAGAGAATCCTCAGCGATTCGTCACGAAG
GGATATAACCTGGCCACGCTAAAGACTGGAGTGGATGTGCATAGGCTCACCCCATTCCGGTGTGACACCATTATGTGTGACATCGGAGAGAGTGAT
CCCAGCCCAATCAAGGAAAAGACCAGAACTCTTAAAGTCTTGCAATTGTTGGAAAATTGGCTCTTGGTGAACCCGGGAGCCCATTTTGTGTGCAAG
ATTTTATCACCATATTCTCTTGAAGTCCTCCGGAAAATCGAGTCCCTCCAACACCTGTACAATGGCAGGTTGGTGCGTCTCTCCCACAGTCGGAAT
AGCTCAGCTGAGATGTACTTCATTTCCGGGGCTAGGTCTAACGTGGTGCGCACCACGTACATGACGCTGGCTGCCTTAATGGCCCGATTCTCCCGA
CACTTAGACAGCGTTGTCCTCCCGAGTCCCGTGTTGCCCAAAGGCACACGGGCTGACCCGGCTGCCAGTGTGGCATCCATGGATACTAGCGACATG
ATGGATCGTGTTGAACGCTTGATGAACGAGAACCGTGGGACTTGGTTTGAAGATCAACAACACCCCTACAAGAGTTTCAAGTACTTTGGATCGTTT
GTCACCGATGATGTGAAGGTTGGAGGGCAGGCGGTGAACCCCCTCGTTAGAAAGATCATGTGGCCATGGGAGACTCTCACGAGTGTGGTGGGGTTT
TCCATGACTGACGTGTCTACCTACTCACAGCAAAAGGTGCTGAGGGAGAAGGTGGACACTGTCATCCCCCCCCACCCACAGCATATACGACGGGTT
AACAGGACGATCACAAAGCATTTTATCCGGTTCTTCAAGAGTCGTAACTTACGACCGCGTATCTTATCAAAAGAAGAATTCATCGCCAACGTTCGA
AATGATGCGGCTGTAGGATCGTGGAGCAGGGATGTGCCGTGGCGAGATGTGCAAGAAGCCATTCAGGACCAGTGCTTCTGGGATCTCATCGGAAAA
GAACGGGCCCTCCACCTGCAAGGGAAGTGTGAAATGTGTATCTACAATACTATGGGCAAGAAGGAGAAAAAGCCTAGCCTAGCTGGTGAGGCCAAA
GGCTCAAGGACCATTTGGTACATGTGGCTTGGGAGCCGGTTCCTTGAGTTTGAGGCTCTAGGGTTTCTTAATGCGGATCACTGGGTCAGTAGGGAG
CACTTCCCTGGGGGAGTTGGAGGCGTTGGTGTCAACTATTTCGGGTACTACTTGAAGGATATTGCCAGCAGGGGAAAATACCTTTTCGCGGACGAT
ATTGCTGGATGGGACACGAAAATTAGTGAGGAGGATCTGGAGGATGAGGAAGCTCTTCTAACAGCCCTCACGGAAGACCCTCACCACAGAGCGTTG
ATGGCGGCTACCATGCGACTGGCATATCAGAACATTGTGGCCATGTTCCCACGAACACACTCAAAATACGGTAGCGGCACGGTGATGGACGTCGTT
GGTCGTCGAGACCAACGTGGGTCAGGGCAAGTAGTCACATATGCGCTGAACACCATAACGAATGGTAAGGTCCAGGTGGCGAGAGTTTTAGAAAGT

GAAGGACTACTGCACGCGGATGAATCAGTGTTGGATGCGTGGTTGGAAAAACACCTTGAAGAAGCTCTCGGAAACATGGTTATCGCTGGAGACGAC
GTTGTTGTGTCGACAGACAATCGCGATTTCTCATCAGCTCTCGAATATCTCGAGCTGACGGGAAAAACCCGGAAGAACGTCCCACAGGGAGCTCCC
TCCAGGATGGAGAGCGACTGGGAAAAGGTGGAGTTTTGTTCTCACCATTACCATGAGATGTGCCTGAAAGATGGCAGAATCCTCATAGTACCTTGC
CGACACGAGAATGAAGTGCTTGGTCGTAGCCGACTGCAAAAGGGAGGGGTAGTCAGCATTTCAGAGAGTGCTTGCATGGCCAAGGCCTATGCCCAG
ATGTGGGCATTGTATTTTTTTCACCGGCGTGATTTACGCCTAGGTTTCATTGCTGTCTCGTCGGCTGTGCCAACCAATTGGTTCCCTCTAGGACGT
ACCTCGTGGTCAGTCCATCAGTATCACGAATGGATGACCACTGATGACATGCTTCGGGTTTGGAATGATGTGTGGGTACACAACAACCCGTGGATG
CCAAACAAGGAGTCAGTTGAATCCTGGGATGACATCCCCTACCTGCACAAGAAGCAGGACATAACCTGCGGAAGTCTGATCGGGGCCAAGGAGCGC
GCAACGTGGGCACGAGAAATTGAAAATAGTGTCATCAGCGTCCGGAGGATAATCGACGCAGAAACTGGGGTCCTGAACACCTACAAGGACGAGCTC
TCAGTTATGAGTCGATACCGAAGAGGAAATGATGTCATTTAGACGTAATCGAATAGAGCCGTGAGGAACCAGCCTCCTCCCGGCTATATGAGCAGG
GCATGAAAATGTCGGGCATGACGAACCCGCTCCCCCGAGTCCCCTGGCAACAGGGTGTGTTCCCACACGGAGCACGTTCGAGCAGGGCACATTAGT
GTCGGGCGTGACGCACCCGCTCCCCTCAGTCCCCTGTGTAACAGGGAGGGCACTTGCAACCCCCGTAGGAGGGTGCCCGCTTCCGTCCTACAAAAA
CCTCTGATCACAGGTACCTGATCTAAGATGGTGGTGGCGGCCCATCTTATCATTTAGCTAGCTGATGGTCTTAAGCATCCCACCCATGGAATGGGT
AAGAGAAGCCTGTAAACAAAACTGGATGGCACCAGTGCTCTTATAAAATGGCAGCCAAAGCGATCCAGAGCTTTCAAAACTGGACGGGGCAACAGG
GAGAAATCCCGGGGTAGCGAACCTCCTCCGTTAATGTGAAAAAGTATGGGGAAAGAACTCATCTTAACCTCCCACCGTTAGGGAGTTTTGATAATC
TTT

>c27006_g1_i1_len=1437
CTGCGATTATCAAATTCGGGTCCCTTTTTAAAACTTTTTTTAATAAGGTTCAAAGGTCATTGCCTTCTGTAGGGAACAGTAAATTCCCATACGTGT
AGGAAAGTATACCCATCTTACTACACTAGTCTGCGATTATCAAATTCGGGTAAGTTGAACTATTTCATTAAGAGATTTTAAAGCTTGATTCTAGAA
TCAATTCCTGGAGAGGGCCCTAACGGGTAAATACTTAAGTTTTAACTTCTCTGATTTAGTTTGCCGACACTCGGGGTAGTCCAGGCCTTGAAACTA
ACCATTTCAAGCCGTTTGAAACGTAGCGGAGCTACTTTCAAGGTCATTCGGAATCTATTGAATGTACATCAATTGTACCACCCGCGAAACACTGCA
CGCTAAGCAATTAATTTGCGAAACTTCTGACATTCACATTGATCAGTCCTCGTTTTGGTTGATTGTTATCGCCCTCGTATTGTTAACTAGGTCTAT
AGTTCATCTCTATCAGCATGTCCGGAAACCGCAGATTTCGGCCCAAGAAATCCCACTCCTCCGCCGTTACACGGCAAGAACTGAGCGGAATGAAGA
TGAAAGTGCCTGAGAATCCACCTGAAGTGACCTATCAACCATGGAACCAGGCCACTATTGTTGACAATTTTAACAATAAAGTTGTATGTACGGTCC
ACAGCCTAACCAGGCAGATTTGGGATCAATTGGACCCTAACCAACATGGGTTTAAACCATGCGACGAGGACCATACGAAGAGTGAATTCCGTATAC
TGATCAAAATTCAGACCATCCGTGCGTGGGCATTAGCGGGGAAGATGATAGCTCTTTCTGTTGAAGATTTCCTTGTTCCTAACAAGGATAAAGCCG
CCGTGGATCAGCTGTGTGGGATAGTCGACACGGGTAACGCTAATCATACTCCAGCTCTTGGTTATAGATTACCCATAGCCCATCAAAATGCTGTAC
ACCGCGATTCAAAGGATGAGTTTGGTTTGCAACATCTTTTTCAAATTCAATGCGATGGTCCAGCTATATGCTACATAGGCATATTATGGCGATTTG
ATGGACCGTCAAAACTACCGACATTTAATAGATGCGTCGAGCAAATGGTGAGAGACATTAGATTTACTAGTACTAACACAAATAGAAAGGTACACT
CGTTAGATGAACGACTGAAGAAAGTGAATGAACAGTTAGATGATCTAATCGATAAACAACCTTCTATAATTAGTGAACTCTCCAATGAGATCCCAA
TTGGAGCCGCAGCTGTAACCTTTGCAGCTAAGGATACTATAGTAGATCTATTTTCTGAACTTAAGGCCCTCAGAACAACTCTCTCTCCTGCTTCTA
GTTTTGACTTTGTCGCCGATTCCGAATAACTTATATATAATCGTAGCTCAAGAAACCATATCCCAACCTCGTAAAGTTTCTAGAGTGATTATA

>c123170_g1_i1_len=521
GTTGGTCCTTTGACAGGGCCATCGAATCTCCACTCCAATCGTACGTAGTTCATGATGCTTGAGGAACTAGCGGCGGAGGTTGTAAATAGCTTCTTC
TTTCCAGTCTTGTCATCGTTACGGATCACAGTGCTTCGATGCGAAAGAGGGAGCTGGTATCCAAGGCGTGGTGCCCCGGTCGATCCGGCATCCATC
ATACCGCAGAGCTGGTCACTATCATCCAAATCATGAAAATCATACACAGTAAGGGCGATCGTCTTGTCGGTTAAATTCCAGACTTTGACGCTGTGG
ATCTTCATTTGGACGGCTGGGCTCTGAGCAAGTCCGCTGCCCTTTGGGTCCAACTGGGCTTTGATCTTGTTAACAAGATCGCCAAAGTTGGTATCG
CCTGGCTTGGAAATCCATGCTAAAGTCAAATGGTTCCAAGGCTGATAGGCAACCATAGGGGGGTCCTGTGGTGGTTGGGCACTGCCTCCGAGGAAC
AAACGGTTCTCGGTACTCTTCGTGCCTCTGTGCCTCTGGGG

>c36375_g1_i1_len=6510
CTTCACGAATGATTTCTCATTCGACTATTAATGAAAACAAAACAAAAACACTAACACGCAATTCAATGCGCTCACTAACTCATTATAGTCGAATGT
TGAAACTGAATTTGTTAGCGTCGGTTAGGGGTGAAGACCGACTCGGTAAACATTTCAACATCAGATGGCTCAATTGCTTGAATCAACTCCTCCTTA
TCCTGTGCCGAAAGCTTCCTATACCAAATATGATTAGGAGCTTCGAACAGATGAATAAATTTTTCTTTATCCTCAAGTAGTTCTTTCAAAAAGTTC
AAAATGTAATCAATTACGAACGTTATATTTTTCATTCTTTCTTTATATCGACTGATAATACTTTCTTTAACAGCTAAACATATACTATCATTTAAT
ATCAATTTTAAATTATCACTGAATGACACGTGATAAAGTTCAATATGTTCTTTACAGCGCATATCACTTCTTCCTAACCGCACTATTGCCTTTAAA
GGATCTGGAACGAAATACCAATGGCCTTCCGCTTGTACCAGAAATTTAGATGCAAAGTACGGAGCATTTGGGTGGTTTTCCACCTTTGCATATAAA
TTAAACGTTTCAGAAATAAAACTGGTACGATCAAACAGTCTAACATCTTTGGAAAAGACAACTAGACTATCATCTCCTCCAAAAATACCTCCTATT
GCTCGTTTAAAATCATATGTAAAGGCTAAAACCATCATCGTCACTAAAGTATTACCCAAAAAGGTAAACACATCACCGCTTCTACGCTGAAACTGA
GTAGTTATGGTAACTCCTATATTCATAAACAATAATTTGTTTATAGTATGGCTTTCAAACCATTCCTGTAAATAATCTTCACTAACTCCCAGTCTT
CTCATCAATTCTAACTGTGCCAAAAGTAATACTTCGCCTTGACTTTTATCAAACTTACTAAAATCTATTTCTTGATATTTTATTTCTGTCTGATCT
TGTAAAATACTATGCATATGTCCGCTCAGTTGCATCAGATTAAATCCATCATTGATGAGCCATTTATCATAGATAGATTCTTTCAAAACACAACCA
ACGTATTTTGCTAAAGTTGTTAAACTAGCGGTAATAAGTGGATGATGAGCTGTTATAACTTGACCGCTAGTTATTTGATCATTATGAGTATTGTCT
AAAGGTGGTTTAACATCTGGTTTAACATGACAATAATACACATCTGACCTTTTGGTACCGTATTTTTCTAAATTCAAGAAACTACGTTGTAATTTG
GTACGTGTAGAATACCAATCATATAAATAAACTGCATTTTTTAGACTATGTTTTTTCATTGAGTCTTTCATTTTATCAAAATCTAAAAACGTTTCC
ATAAATACATCAACTACCTGTTTTATAACCTCAGAAGAAACTGTTAAATGTAGAATTGGTGGGTTTAAATTACGACGTTGAATAGCAAGTAGAATT
TCCGCAACTGTGTTTCTAAGCCGCGTTGGCTGTGGTGTTTGAAGTTTAGGAGTACAATACTCTTCAACTTTATACTGTTCAGAAAATATACTACTT
ACAAATTTCGATAACTCTACGTTTATATGCTTATCCAAAGGATAAATTATCATAGGTATTTTACGTGTCGATCTGTCATAGATCGAATCCAAGCAA
TACTGAAGCATCGCTATAGACGTATCACTATTGACTTTACTTAATGTTGGATAATCCAATTTTAACAAGTTGATCTGTATTGTTACAGATTCATTA
AAATTTAGACCTCGATCTAATGATATCATCTCCAAAACATCATCATCAAAATGTTTAGATTTTATTAAAGCTTGATATATTGATTTTTCCTTCCTA
CACGATAAATTTATCTTAGTACTTTGGCTAACTTCAGTTTTCAGATCTTTAAATTCAATTGATTTATTTAAATTCAAAAATTGCGACGGCACATCT
CCCTTTACTACCGTACGATAAACTAATGTTTCTCTGTGGCGACTAAATGCCACAACTATATGAGGTCTGCTCGCATAAATGATATTATCTTGAACT
ATAAGTCTGATGACTTCTACATGATCGTAAGTTTGACCTTGACTTTCATGTATTGTCATCACTTTATACCCAGAATTAAATCTCAACAAAGTTTCT
TTTTCAAACTGAGTAAATGTTAAATAACACTTTCCAATATCATTCAGTTGAGGTTCCCTTTCTCCTTTCACGACTTTCACACTACCAAAAATTTGT
GAAGCAGTTTCAATATTGGGATAAACACGATCAAATATATTGATCATGATTCTTGGTAGTCTATAAGTCAACTTCATAGTTGTAATTTTATCAAAA
TCTATAACACTCAAATGTTTCAATTCTAATTGATTACATCTATTTATAAACGGTATTTGGTTTGTATCTCCGTACATTCTAACTTCTCTACACTTA
GCTAGAAAACATACTATTAACATCTCACCTGGATGTATCATTAAAGCTTCATCTGCAAAAACACGACTTACCTCAGGAGCTCCGTTTAACATTATT
GAATCGTAAGTGCGATAATACTCACTACGACCAAAATTTTTGATATAGTCTCCCAAAGCAGCTCGCGTTGCTGTACAAACTAAATCTTCATTTTTA
CTGTGATAATGACTTATGGTATGTGTTTTACCACAACCCGGTACACCATCAACTAATATAAACTTTATGTTCTCATATTGATAACTTAAAATTTCG
TCTAACCTTTCATACAATCTCTTAGTTAGACTTCTACTTCGGATGTACTCTACATCCTGATTTAAATAAACAAACCTTCTATTTTTTAAATACAAA
TCATATTTGAATTTATGTATCAGAAATCTTAAAAGTACAAAATCTTTGCCGTCCCACGCATAAAGTAACTTTGAATCAGGTTGTCCATTTATGTAT
TTTTTGTCATTCAAATCAAAAATATAAGTACTAGTTACTGCTCTCATATAATGATCCTCCTGACAACTGTGAATTAATTCAAAATTACTTATTACA
TTAAGTTCTAGCATGATATTGTACTTAAAAACCTCAAGTATAGCCAATGACCGTTTCATGTCTGGCATTGTTTCAAACATTCGTAATTCAACATTT
AAAAGCATACTCTCCAACACTTGATATCTCCTTACAGTGGGTTTGGTAAGTTTAGGTACCAACCCCAAGGTTATGCACCATTGTTGTAAATCTCTT
CTTTCGAAGAGTGAAATATCAGCGAGTCGAATATTCTTTAACGGATATACGTGTTGATCAATAACTAGACACCACTTCTCAGGCAGGTAATCACCA
TAATAAACAGAGTTATTATGTATCATACCTATCGCTTTATTATTGCAAACCTCAATCACATGGGATGGAATCGTCACAAAAGACGGCTCTTCACCC

ATAAGTTTGCTTTTATATTCGTAAGGAAACCGTTCGTTAAATATGCAAAACTTAAAAAACAATAATCGCATTTGATAAATCGCTCTAAGTTGCCT
ATCAAAATTAACACATCTTGAAAATTATGAACGTTAACAGTTGTGAATTTACTCCAATATTTGTCACTTAAAGGCACCTCAAATTTCATGACAATT
CTTCTCCAGACATTTGTTAATTCCATTATTAAATTATCAGTAATTAATCTAGATTCGCTCAAATCATTAAAGTAAAGCATCTTACCATGAATTATT
ATCATTGGGATGGATTTCCACAACGTTGCCATAATCCTAATGGTAAGGTTTTGTCTATCAACCGTGCGTAACTGTGCACAGTCTGTCAAAATGTCA
TAAAGTATATACCTATGACCATATCCTCTCACGATATTTCCCACTACATTATATTTTAACTCATTATCTTTAAAATTTACTTTAATGGTTGAACCA
AAACATTGATTTCTACTGAAAATATTACGTCTTCGCGCCAGATATCTTAAAGCATCTGGGCTGTCTTCACCTAATTCCGGAATGAACATTACACCG
GATAAATCCAAAATCTTACTTTTTAACCACAGTCTATCCTTAACGGATGTAACCAAATCGGCAATATCTGGCAACTGATAAGAAATTTTTTCCAAC
ACACTTTCACTAATTAAAGAAGCACTGTCGCCACACGTACAGGTGATAAACTTTTTAAGCTGAGCAGTAATACATCTGCTTTGCTTAAATAGTCTC
AAGCTTACAAGTGTTACATATAATTTTTTCATAAATTGATCATCCCTCAGCACAATAGATGCCACAGGATGATATTCCTCATAAGCTTCTGGATGA
AAAGTATGCATAGCCACATTCCAGAATGTCTCAACATCTTTGTACTCTTTAACTCTATCTAGTATACCAGAAATCATTTTCACCAAGACATTAGGT
AAAAAGTATTCATCATAAATTATTTCAACATACTCAATATGTTGCACTATCCTCACAATCAATTTAAAATATCTAAATTTATGCAATTCCTTATTC
AGCAATTGCATTGTGTAAGGTTTTAATTCCTTACCATCATTTATGTCATATTTCAACATATGCGTATCTGGGTGTTCCATCACTATACCACCACTA
TAATAATATTGATTGCTATATTTTACATCACGAGTTAGATACAACTTCTTCAAGTTATCTAAATCGTGCTGATAAATTACTTCGGACTCATGCAAC
TGCACGAACTTAACTTGTCCTGATTTAACTTCTTTCTCGTCTAAGAATGAATTTTTCACAAATTTTTGACCCACACTAGGTAATATACCACTATTT
CTAAGAAACAACTCAGTAGAAAAATGCAGCAATATGTACACAGTACTCGCTCCTAACCGCTTCGCATTATAAAATATTTCCTCAACTCTGATATCA
TAAGCGCTATGAACAAATATAAGTATATCAAATTTTCCACAAATTTCCTGCAATCTTTTTCCAGTCCATTCAATAACAACTCCTTTTTCGCGATGT
AATTTCCTAATATACTCCAATTGTTTCGATCTACGTATCGGATCTCTGATATCAAGCATAGGTATAGAGTGAACATGACCTTCAGCTGGCCAATGT
TTCACTTCTGACCCGACATGTAAAACTCGACAACCAGCAGGTATCTGCCGTCGAACATATTCGATGGCAGCATTCCTCAAGGCTGAAGCAACAGGA
TGAGGTGAAGTTGTATGAATAGATTTCTCAAGACCAATCGACGAACTGATCACTTGACGTAACCAATTAAATTCATGATCTTGAAGATCTGCAGAT
ACCTTTACTGAGTCACTTTGAGGCTCGAGCGTCAACGAAACTTCTTGTGACGGGTCCAACGCTTCATAATACAAGATGTCTCTTGCATCATCAGCA
CCAAACCAAAAAGGTGCTGGCTCAATCTCGCCGTACACCCACTCTCCAGTATCTATCCTTTGGTATTCAAGATAGGACTGCTCAATAGTGACAAAA
GGTGTTAAGTCTAAAGTATTATAATCAACATCGATATCTAAAAATATTTCTTTTTCTTCTTCATTTATAAGATCCTGTGTCTTATATTGTTTATTT
AAAAATCTTGGCGCTCTAATCAGTTTGATCACATAGAATACAAACCACAGCAGAAGTATGGACTGCAACAACAATCCAATAACGCAAAAACCGAAA
TACAACAAAGCTATCATTACAATAACTATGTTAACTGTAAAAAACCTTAAATAGCGCTTCTTCGTCTGAAGATTGTCATTCAAGTAAACAACTGAA
CATGATGAAACATAGTAATCACATACTCCGATTACTTTATCCAAATTATTTTTTAGAATATCCAAAGTTAACTCATTAACATCAGACTTAAAAACG
TGTAGTGAGTTACCACGAATGTAATAACTCATGCCAATAAACTTCAAACGTCTAGTATTCTTCAAAAACTGTTCAATTGTTGGGCAGTATCCCAAC
CTCTCCTTACTCTTAAGAACTTCAGAAAAACATTCATCTATTAGATTCAGATAACAATATCCGTCACCAAATATTAAACTTATCAAATCCACGTCC
ACACAATGGCGCCAAAGATTGGTCAAGCCATTGTGACCCGCTAAGCGTCTCAAAAGAGACACGTAACCCAATAAGTTCCGGTCCACGAATGCGTAT
GGTACCGTAATGTTGGGAACCCATGACATATACCATCGGTGTCCAATAAAGCGTACTCTATCTTTTTCCTTAGGCAAATCATGGTATGACCAGCAT
ATTCCATAAGAGATGTAAAAACGCATAGCAACCCTTAAAACCATCTCCAAACCAATTCGGTATGTATGGAACACCATACCGGTGGTGCCCGATTCT
GTACGACGATAGGCAAATAGTCGATACAGCCTTGGTGAAGTGCGATGTAGGTCAGTACTCGACACCAAATAGACCGCCTCAAGGACGAACAGGGTA
ACCGGACCTGCGGCAAGGGAACCGGGGCAGTCCGGGCCTACCACGTAACCCCTCTCAGGTGATACGCTCTCGGCAGCGACGGGCGACGGGTGTTCC
TTCCCCACTGGGTTGGCCGTCGAGGCAAACCCGCCTAAAACTCTACCAACAAAAAGCATCGATAATGCGACAGCGAAG

>c30446_g1_i1_len=3790
GGATTATGGACTTATATCAAATTCCTATATGGATCACTGGAGGTGGAAAATAAGAGAAAAACACAAGGTGGAAAATAAGCTTATTCTCCACACACA
AATACACCCTTAATTTCCACTACCACATGGTCCACCCCTATATAAGGAGTACAAAAGGAGAGGCGAATCGAGTGATGAATTCAGTCTGCAGCGAAC
ATTCGCCGTGTGAACACGGTAACTTATATTGTGAGTGCATATATTGTTGGGAGCATGACGGACAGTGCAGGAGGAAAAAACTGGATTTGGGAGAAT
CAATTGGAGTCGAAGGAAGATTGGCCGACGATAACCAACAACCAGGGCTCTCAGATTTATATTGCACCGAGACAATACATCTTACAACTGCAGTTC
AACCGGAAAGAAGAGCCATCAATCGAGAAGATTACATCAAGGATTTCGCTGATCAAACCGTTGGTGACCTCTACCCACAATTACAAGGCAGCACCG
GAGCCTCTGAACCAATTGATTTCGCATTTCCAACTGTTGGCTCAGGAAGCTGGGAAATACTTGTACGTGAATCTCACAAACATTTCGAGCCAAATA
ATTCGGAAGAAGCTTATCAATCACATATTAGAAGTGTACGAAGAAGACTATTCCCCGAAGAAACTATGGATAATAACGGGTCACAGGCAAGCACGA
CCGAAATGCTACGAGACGCTGTCAAAAGATGCGGTTTTGAAGGCCCTCCTAACAGCCCAAGCGAAAATAACGGAGATGGAGTTGATGGAACGTGTA
TATCAACCGTGGAATTACACAGCAATTGTATTGTTAACGCACATTGCCCAAAACAAGGACCAAGCAATCAAACCAATAAGAGAAAGAAATCAACCG
ATACAACAGAATCAAGCGGATCCAAAAAAAAATAAAAGCAGCAATAATCAACAAAATATTCAAGAAAAAAGCAGTTCCAGCATCGCCGACGCAGTC
GATCTCATCGACGGAGAGTTGGATGGATCAACTGGATCGAATCGAGAAACAGCATACTACACATTCGTCCTCCACAAAGACAACGTTAAAGAGGAC
TGGAGATACATCGCCACACAACAAGGGCCAAGCAAGCGCCGAGTTTCATCACATTCGATCACGGAGACCACATCCATATCCTCTTCTCCTCGTCCAAT
ACAGGAGGAAACAGCACCAGAGTCAGAACCAGAATCACCAAGTTTCTTAGTGCGACAAGCGCAGGAAGTGCAGAAGCGACTATCACTTTTTCCAAA
GTTAAATTTCTCAGGAACTTCATTCTCTATTGCATCCGTTACGGTATCGAAACAGTCAATATCTATGGAAATAAAATCCAACAACAATTAACCGAA
GCAATGGATACATTTAAAATATTATTTGAAAATAGAGACCCGAATGACGTAATATTAGAAGCCGGTTGCAAACTATATCATGAAGAAAAAAAGGAT
AATAAACAAAAAAGATGCGGACAACGTAAACAACAAAATTTAACGGACATTATATTGGAAAAAATTAAAGAAAGAAAATAACAACGGCACAGCAA
TGGGAAAATCTAATTGAACCTGAATTCAAAATACAATTAATGAAAGAGTTTGGATTAAATGTAGACAGTTATGTAACAAGAATAGTGCGCATTGAA
AGAACACGTATACAACAGTTGATAAAAGCAAAAACGCTTACGGAAATAATGCTTGAAATATTAAATGACGAATATATTAAACATTTCACACCAGGA
GAGGACAACAGCAAAACAACAAAATGTATAGAATGGATTGAATACCTATTCAAAGAAAACAACATCAATATAATCCACTTTTTGGCGTGGAATGAA
ATTATAAAAACAAAAAGGTATAAAAAAATAAATGGAATGGTATTAGAGGGGATCACAAATGCAGGAAAATCACTAATATTGGATAACTTGTTGGCC
ATGGTAAAACCAGAAGAAATCCCAAGAGAAAGAGACAACAGTGGATTCCACCTTGACCAAGTACCAGGAGCAGGATCGATTCTATTTGAAGAACCA
ATGATCACACCAGTAAATGTCGGAACGTGGAAGTTATTACTAGAAGGAAAAACCATAAAAACAGATGTGAAAAATAAAGACAAAGAGCCGATAGAA
CGCACACCAACGTGGATCACTACAGCAACACCAATTACAAATAACGTTGATATGAATGAGACATCACAAATACTACAAAGAATAAAACTATATGTA
TTCAAAAAGAGTATCCAACACAGAGACGACAAATATACTATAAATGCGCAAATTCAAAATAAATTGATCAGTCGTCCTCCAACTCTCATTGAGCCA
ATACATATGGCCATAGTGTTTATAAAAAATTTCACAAAAATATATAATCTAATCGAAGAAGAAGACAAAGCACACACAGTGAATGAAAAGGCAATA
CAATTAAGCAACGAAGTGAAAGAAGAAGCAGAGTCATGGCAGACAGCACTTCAATGGACCATGATGGAGAACAACGAGGAACAAAACGAAAACGAG
GCGCAGAAGGATCAGGAGCAGGAATTGGAAAAGGAAATAGCAATTCCGTAAAAGAAGGATATGGACCAAATATCATCGAAATGATCCCAAGAAATA
TTATGAACAAAGGAAATCATAGTGTATTACATGTAGTAAAGCAACAAAAATACTTGGACTTCAATTTCGTATCGAATCAAAACCCATACATTATAC
CATATCAAACGGCAGGATTCTGGGCATCCATGTGGGACCAAGGAGAAATCGGATCAAATAATACCATCAATATCATGAAAGCACTAAATAAAGTAG
CTCTAGGAGTAACATGGATCAGAGGAGAAATCACATTCGAAGTATATACAGTAACCAGACAACGTTTGCTAACGGGAACGACGAACCAAACTACAT
GGGACTTTGAAACAAGTCAAAACTTGTTCATCGCAGATGCAGACAGAGAACCAGAAAACTTCGGATTAGCATCAGCAGCAGCAACTGGTCCACTTG
CTCAACAAACAACACAAACACTACTATTCAATAAAAACAACGACAGATACACAAAATATGAATTACCACAAAGAAATCAATATACAAGGGAAATCG
ACTTCCAAAAACTAACAAACAACTATATGTGGAAACCAACGGACATAAACGAAGAAGTAAACTTTAGAAGGTTAATCCCAATGTCGGAAGGAGTAT
TTACAACAACAAACGCAACAAGCAAAGTGTCAGAACTAACACAACAAAATTCAGCATGGGCTACATCAGGAAAAACTACCCAAGGAACATTATTCA
GAAATAGAACATCATATCCAAGAATGCATGTAGCACAACCACAAGTTCCAGATGAAACCGGATACATGAAGTTCAGATATCAAGTACGAATGAGCA
CCAAATTGTACCTAGAATTTCATCTCTACCCAGATTATGGATCATCAACAAACTTGGAATATATGCACAGACAAGTACTGGAATTACCAGAAGTAA
CAGCCACAGGAGGAGTAGTAACATGTATGCCGTATGAAATCAAAACTTAAATATTTTAATCAACATGTATCAACTATAACACATATATAATCAATA
AAGCATTCAAAATATATATACAAGACAATTTAATATATATCACAAAAATCCAAATTAAAATATAAGTTAAATTTCCACCTCCGTAATCCACATAAG
AGTGTTGACTTAATTTCCACCTCCGTAGTCCACCTAAGAATATTGGCTTAAATTCCACCTCCAATGATCCAGTTAAGAAGCCAACATTAGTCCGGG
ATCCCCGTGTGAGCCGATAGGCGAGGATCGAAAGCCCAAATTTTGC

>c27654_g1_i1_len=526

GTTGTCTTGGATCCATACGCGATTCCATACAGTGAGCATGTCCTCTGTTGTCATCCATTCACAGTGTTGATGGATGGACCACGACGTTCGGCCTTC
AGGGAACCATTCCACAGGAACACTAGAAGAGATGGCCGCGAATCCTAACCTCAAGTCTCTTCTGTGGAAGAAGTACAAAGCCCACATCTGGCTGTA
TGCTTTGGCTAGACACGCGCTGTCTGATATCGTCGAAGTATCTGTTCTGTCTTCCTGTTTCCTTGTCGAGTATCTGTCGAACTTGGACGACAGTGT
TTTCAACTTCCTTTGCCCACGTTGTCCGGTCATGGTCGCCATGTGTCGTTCGGACATTTGGCATTGAAACACCCGAACGGTTTCAGGTTCAAAATC
AGAGTACTACGTTTATTGCATTCCATCTTACAGCTAACCTATTTCTTAAAGTCTACAAAACTAATACATTGATAGTCATTCTGACTGGAGGAGGTG
CTTAGCTTCGATCGGGCCAATCTGTTATGTCCCAATGACTGGGTGA

>c27654_g1_i3_len=368
GTTGTCTTGGATCCATACGCGATTCCATACAGTGAGCATGTCCTCTGTTGTCATCCATTCACAGTGTTGATGGATGGACCACGACGTTCGGCCTTC
AGGGAACCATTCCACAGGAACACTAGAAGAGATGGCCGCGAATCCTAACCTCAAGTCTCTTCTGTGGAAGAAGTACAAAGCCCACATCTGGCTGTA
TGCTTTGGCTAGACACGCGCTGTCTGATATCGTCGAAGTATCTGTTCTGTCTTCCTGTTTCCTTGTCGAGTATCTGTCGAACTTGAGTATCTGTTT
GTTGAGTGCCAGTAGTGACAGTAGTGCACATATAGAAAGGTCGTGTAGGTGCTATCGAGTCACTACCACTGCATCACGTG

>c36290_g1_i1_len=1668
CTACACATTTATTCTCGTTATTGTATAAAAAAAGGAATAGCAGTAGGCAAGAGCTTCGCTTTTCGTTTCTACACAGCCCGTACTCCCGCTAGTTGA
GTACTAAAGTACGGGGGTTTACTGTTTTCCTTCAGTCGCCTTTTGTTGGTTTCATTTGGACTCAGGGGCCCATTTCTGGGACACAACAGGCGCCGG
TGACGACGAAACTTCTTGGACGGGGATTGGAGGAGGGAGAACAAAGAAGGGATGCAGAATGGTTCCTCTATCCGAGATGCCAGTCAAAACACGCAA
AACTTTGCTCTGCATTTCCAAGAGTTTCGGATCCCTGAAGACGGTCACTGGCGCGGGGAAAAATCCTTGTCCCACTTGCTAAAGCAGGCACGCGGT
CTCACTCCCGCCAGCACACCCACTCCATCAGTTTCTTGATGGATATCCTCTATGGTGTCCGATTCCTCTTGGCCACCAGCCGCTCCTTCTTCCTCT
AAGGACGCTGTTGAAGCTTGGTTGACCCAAGATTCAACCGCGAGAGAGCTCGCACAAAAGCCTCCCACTCCGGCAATTCCCGAGCTCGCTGCGGA
ATCATAGATTCCAAGGTTGCTGGAAGCATCCTAGCTACGAACCGGGGTTTTGCGGGGGTTGCCACCGCTGAGGCGGCGGTGTACTTTATCGCCTTG
GTGACATCACCAGAGCGCACGTACGCCACCGCATCATCGGACGTGGCGAACAGGAGAAGCCCTGGCGTGGCATACCCCACCACTTTGGCGAAGAAC
AGACACGTAGCAGTCTTCTTGCTTTCGTCCACGTACCGAACGACGGTACCTCCCGACGGTATATAGTACCTGTCACTCCGAGCCGAGCTGAAAGGC
ACCATATCACCACCGGAGTGCATCTTAAAAGTCAAACGCTCCGAATTGAAAGAGCCGTCCGAGGTAGTAAAGATATTACTCCAGCCAGCATCGCAG
GTTATGGTATCCTGCAATACCGAAGCTGGAGTCTCTGCTCCCTCAAATTCAACAGCCCATTCAAGGGTAACGTTGACCGTGATGGTACCAGTGAAA
CCTCCGGCTGCCGCTCCGACCACTCCCACCAAACTTCCATGGGTGGAAAGGCCGACCTCGCCGTCGCAGAGGTACCACTTGGTAAGCGTTTCAGTG
GGTATTGAGAACCTTTTGCTCTCATAAAGCCTCATCTGAACGCTAGGCTTAAACGCAGAAACCCTCATGAAGTCTGCGGAGGTGTTATTTGCACTC
CAGTGCTCATCTGGAGTCCAACCCACAACCAACGACCCATAAGTGGTCGCACATCCGGAAGCGGTAACGTTAACCACTAGACCTCGAGGCCGCCAT
CTTGACCAGAGCTCTGACTCAGCTTTGAGCCTAGAGTTGGGCAAATCAGACGGGGCGAGAGGTTGTATAAATGCAACCTCCCCGACCTTTGCGGTA
GCCTTAACACCAGCCGTAGCTAGCAAGTCGACTCCCCGGCTGACTATGACGGGCATCGTAGCACGTGGTTGACGCACCACTTGCCTGGCCGGCCGT
GCCCTAGTGGATTGCACGGCCTGGCTATGAAAATCTCTCCTGTGCTGCGCCAGAGCAGCATTAGTCTTGAACTGGCGATTGCACTGTTGACACTTC
ATCTTGTGTGGCGCTTGATAATCTAATACTTCATAG

>c36361_g1_i1_len=2839
CAAGTGAGAAAGTGTTTTAAGTGTTCTATAAACCATTAAATCTTTTGCAACTATGTATTCAGACTTCGGGGAATGCATGGGCTTGACTTTCAAGTT
AGCGAGCGGATTTTTCCGCGAGTACTCCAGCGTGGTTCCCACGGTGTTGGAGTCTTGTCGTAATCTGTTCGTAGAGGAGATCGTAAAACCACAGTT
ATTGTCAACGACCGTGGAGGTTCGGCCAACCAAATTGACCATAGGGTTGGCTGCAGCCGCTGCGGCGTATAGTGCATTGGCAATTGGTAGGAAGTT
AAAGAGAGCTAAGCCGCCACGTTATGAGAACAAGCCGCCTAACTACTTTAAGGAAAGTTCCTACCCTGGTTCGCCCATGATGGATGCTCCAAACAT
TCCACCAGGGCAAGTGAAATTGGCGATGATAGGGAAAGACAATGTCATTCGGCTTATAGGCAGTGGTATACGCGTTGGTGAGTACTTGCTCACTCC
AACCCATAATTTAATGGATGGTCAGGACATCCATATGGTAGGAGGGGCCAAGGATGCGGTTCATGTAAAACTGGATATGGAGCATGTAATAGACAT
AGCTCCAGATGTTTCGGCAATTAAGTTTACTGAGGCTACTTGGTCGCAGTTGGGCATAGCTCGCGCGCGTTTAGGCTGTGTCTCAGGGAAGACCAC
GGCTCAGTGTGTGAGTTCTGTGGACATGCGAATGTCAATTGGAACTCTGAACATTGAGGCCGATGGCTGCCTTGGACGTCTGTTTTATCAGGGATC
AACGATCGCTGGTTATAGCGGATCAGCCTATACTACTGGCAATAAGGTGTTGGGTATGCATACGCATGGCAAGGAAGTTGGCGGTGGAGGCTATGA
GATCATGTATTTGTACATGAAATTGAAGTTAGCCACCCGCCAATGGGATGAGCGTGATGATAATTACGATTATACAGATGCTCAATATGCGACCAT
GCAGTACGAGGATGGTGGTGAGGACCTAGGCAAAGCTATCGTACGTGAAGCGAGTGGCAAATATCAGCTCGTTAAATCAGAGTTGATTGCGAAAAT
CGAACAATGCCGTCGTGATTACGTTCGAGGAAGCTGGGCCGATGAGAGTGAGCTATACGATTATGAGCAGCGACTGGCTAGAGGCGAGTATAGAGA
AGAATGTGCTCTGCCTCATTACTCCGGAAACGTTCCTTCGTTTTCGGGGGAAGGCCAGTCTCCAGCTCACGGGGCAGAGTATGGAGAGGTAGCACC
GAGGCCCCTGCGGGTTTCAGGTGTATCTCGTCCATCCCCGGTACAGCAACTCACAAGGGAAGCTTTGTATCACGCCGCTTGCAACACGCTCTCGAA
GAAAGAGTTGCGAGCTGCCCTGAATTACAAAGAAGTTGGATCACGGCCATCAATGAGTACAGCCCAGCCGACACGAGCCAAGAAGCGGAGTGGAGC
TCCTTCTGCATCCAATTAAATGAGGCAGCGGAGCGACGCAAAACGATTGAGGCCCCGAATGAAGCTGAAATAGAGGCGGTCACTACAGTGTTAGAA
GATTTCTATAAAACTGCTATGTGTAAGCCTGTCAGCTCCCTTTGGAATTCTCGTCAGAATTTCGAACAATTGCTAATGACACTGGACAATTCTTCG
GCGCCGGGTTACCCTTATCAACGAGAAGCTCCAACCATAGGTGATTGGTTAAAGATCGTTGATGGAATGCCCAATATAGTTCAAGTCGAGCGCCTA
TGGTATGACGTGCAATTAGTCATGCGAGGCGAATACGACCACATATTTAGGGCTTTCGTCAAAGATGAACCACATAAAGCGTCAAAAGTCAAAGCG
GGTAAGTGGAGGTTGATCATAGCATCGTCTTTGCCAGTTCAAATGGTATGGCGAATGCTGTACTTTAGTCAGAACGAGGCTTTAAATGCCAGTTCT
AGCACAATCCCATCAAAACATGGTTTTGTGTTTTGCTATGGTGGGTGGAGGAGATTCCTCGCCATGGCTAAAACTAAAGGAATTACAATCTCGCGT
GATATCTCAGCTTGGGACGTTAACGCTCCTGGTTGGGTCTTCAAGGTGGTGGGTAGGTTACGGGCCAGATGGTCTGGAGTTGATGATGAATGGAGG
CGGGTGCACAATATGTTGTACACAGACGCCTATGAGGATTCCCGGATCCTTTTCTCTAATGGATTGATTGTTAAACAGTTATTCCAAGGGTTCATG
AAGTCGGGTCTCTTCAATACCATCACAGACAACTCCATGGCTATGGTGGCGATGCACATTCTAGCTTGTTTAAGATCTAACTCAATTATTGGCCAA
ATTTTCGCCACAGGCGATGACGTCGTGCAGGAAGTTTGTAGTGATGAATATTTGAGCGTTCTTGAGAAGCTAGGGTGTCGGGTGAAGGAGTTGATC
AATCATATAGAGTTTATGGGACTTTCTTTTAGAACAGGAAAGCCTGAGCCCCTTTATGTGGCCAAACACATGGTCAATTTCATGTGTAAGGAGGGT
CTAGAAAAGGAGGTTTTAGACAGCTACTGTCGTAATTATGCTGAGTCGGAGTATTTTGATTTCTGGCACGGGTTAGCGAAAAGTTTGGGTATACAA
TTGCCCTCTCGTTCCTATTACCGTTTCTGGATGTCGAGTCCCCTGGCACAGCACGTTGCGGCAGCTAGATTGATGATGTGATCTAGCCCGGATCTC
TGGAGGTGTAAATAGTTAATATAGGCGTTTCGCCGCGTCTATATTAGGTTGCATCTCCAATTTTGTTGGTTTCATATATGCACTCGGGGGGGCTCC
CCCCCCCACCCCCCCCCCCCTCCACCCCACAACACCATCCAACCTTCACTTCTTC

>c32738_g1_i1_len=529
AACCGACCACCCGCGCATACTCATAGTCGAAGTGATAGGTTTCAATTCCCCTCTCAGAAGGAAGGCAGCAACAGGGAAAAACTTCGATTTGTTTAC
CTATTTCAACTCGAAGATAACCTTAGTTGAAAATTATATCGGACAAAGCATGACAGAGCTTTATAACACCGTTATGACGGAAATGTGCAAAGTAGA
TAAGGCGCTAATGGAGTGTAGAGTATTTAGTTACATCATATCATAAATCACCTGACTTCTTCAGCACATGTTCTGTGATTGATCTCTCTCTCTCCT
TCTTCATTATTTTATCCTTGTTGCAAACCTCTTATTGATCCCTGATTGGTTTGCCTAAATGAAAAGTAACTCCAAGCACCTCCCACTAGGATAAGC
ATTGTGTAGAGAACAAATCTATGTATGTAGAATTAAGTGATTTGTAAACTTGAAGATTGAAATAAAGACAGATTTTATTCAACAGTAGACTCGAGC
AGTTGATATCCGGAAAGATATCACACTATTGACAAACATCCAAGAAGAT

>c32738_g1_i3_len=664
AACCGACCACCCGCGCATACTCATAGTCGAAGTGATAGGTTTCAATTCCCCTCTCAGAAGGAAGGCAGCAACAGGGAAAAACTTCGATTTGTTTAC
CTATTTCAACTCGAAGATAACCTTAGTTGAAAATTATATCGGACAAAGCATGACAGAGCTTTATAACACCGTTATGACGGAAATGTGCAAAGTAGA
TAAGGCGCTAATGGAGTGTAGAGTATTTAGTTACATCATATCATAAATCACCTGACTTCTTCACCACATGTTCTGTGATTGTAACTTTCCCACAGT

CACAGAACCCGACTTGACAAAAACGTACAACTTTCATATAATCAGCTAATTGTTGCTTGGTTACAAAACGACAATCAGCACCCTAAAATAATTTAT
AGCTTATACTCATACTCAAATAACATAGAACGACGACCCACGTCGTTGTCATGTATTCTCAAATGTCACATAGCAATGACCCATGTCATTGGCACT
TCAGAAATCACTTCAAATGCAAAACCCACATGAACCGTAACTCGACTCCTTCAGCATAGCACTCCGTTCCCAGGCAATAATACTTGCATACACATA
AATGACTTAGACTGCTGCCAAGTGCATCACGCAACAAGCGTTGATGCCTTATTCGCGTTTACTCTTTCTCTCTCTCTCTCTCTCTC

>c54150_g1_i1_len=3099
GCCCATCCCACTATTGTTTGCCAACTCATCCCGTTTCACTCACCATTAGATAGAAGAGAACTTGACATAGAATCAATTTGGTTGATTCATTATGTC
TAGTGCTTCTATCTTAGGCAAGACGAAACGAGGACCTAACTCACGAGCCACTCTACACACAAATTCGTACATCACAGGCTCATTGACGTACAAATA
CTGATAACTACGCAAAGTCTCCCCAAGATTTTGCGTATACTTAAGGTTAAAAAGATGTTTTTGCCAGTATGCCGGATAACAAGTTTTCCCGTCAAA
ACAAAACCCCGCGAATTCAACCCAATGCTGGACCTTAGCTCCTTTGACCGTGATTCCCAATATCTCGAGGTGCTTAACATAGTCTAGAAGCCAATC
CATGGCTTCCTGTACCGTGTCATCACCTATCGAGTATGGTTGTTTCATGGTCATGGGCTTCCCACATCTACTGTTGACTACGTAATGTAAGAGACT
TTGGCTAAGACTATTTAACAAAATAGTTAAATAGCAACCGCTCTTCATTACTCCACGCGTTGCCTGCTTTACTCTTGTTCCGTCTTCGAACTGAAA
CCAAACCTCTTCAAACAGTAGCTTAAAACGTAATTTCATCATCCTAAGCCACCAGTCTGGAGGATTCACTGGTAAATTTTCCAAAAACAATATCCA
AAGATCAACTAAATAGCCTTGTACAGTCCAATCCCAAGCCGATCTATCTAAACAATTTACAGCTTGGTTACCGAACCTGTTCACTATCTTGCGCCA
TCCGCCTCTGACTGGACTCCACCCGATGAGACATGGCGTCTCTCCAACGGAATCCATCTGAGCTCTGGCTATCCAAGCAAACAAAATTCTGTCAAT
AAAGGTATCAATTAAACTGACAGCGCTTATTAAGCGATAAGTACCTTCATGAATTTTCTTCATTTTATGCGGCTCTTGTTTAACAAAAACCTTGAT
ACAATCGCTCACAGCCTTATCTGCCAACAACTCATTGAACCTCAATTTGACGTGCTCTCGTACCATACTTACTCTATCCGGATCACAGCATATACC
ATTCCAACCAAAGATTTGCGCATTCGTAGAGCCTAAAAGAGTCAAAATACAGTAGCCAGGAGTGCTATCCATATCTATCTCCTCTAGTGCTTTATT
GAATTCCCTATCAAACCATTTGTGGGGATCTGCTGCGGATGTGAGGTCGCACCTCCATGCGGAATAGGTCCGTAGAGCTGCTTGAACTGCTCTAGT
TCTTTCTTCTGCTGTTGGCGGCGCGTCCGTCTGTTTTGTTTCTGACAGTGCGTGCGGAAGCTTCTTTTGGCGGAGCACGCGTCAATGTACGGGAT
TCCATAAGGCAGGTCTCCAAAGGTTTCTGGCACATGCAATCCATTTTCTTGCATCTTGGCAAGTCGTGGTTCTGTGCATTTTCCCTCTGATATTCC
ATACCACGGGCCCCAACAACTACAGCTGGAGCCCTGATCAGGTTTTCCGAATCATTAAACGTCATAGCATTTCTCGGCGCTAAAGGTAAATCTTCA
GCTTTCTTGGCATCCGCAGTAGGCGTTTCAGGAATATCTCTAGACTCCCCCCAATATATATAGGCGCCACAAGACTCACAACTAGTGCATGTCCCT
TGGTCTTTACTCTTAGGCTCTGGAGGAACGTACACTGAGGTTTTGCCAAACTCTGACAATAGCCCTTTAGTCATGGCTATTATCGGATCCGTCTCG
GTAGAAAGCTCCTGATGCTGTTTTTGTCCACTCTGTTGAGACCTAGTAAGGTCTCCTTTAGTGGTTACAGCTTCGAGATCAATAAGGGGCTCAACT
TCGGCTTGTTCAGCCAAATATTGAATATCCCCCTGTGCTCGCTTAGCGGATCTTCTCTTTAAGACGGTACTCAACACTTCCTCATCTACAATATGA
TACATCCCTCCAATCCTAACCTTATATTCATCAGGATTATACGGAGATCTAGAATACTCTACTTCTTCATAACGCTCTAACTGCTGCTCTAGAAAA
CTTGCACTATCTTCAGTTCTTAAATTTAACTGGTTTCTTATAACCCGACTAGGCTTGAGTTCAGTTTTTAAGAATGCTCCATCATAACCAAGGTTC
TTAGAGTCCGACCCCAGATGCATTCCAAATATGGTCCTGCCAAAATAGTAAGGTGCTCCACTAAATCCTTTAATGGTGCTCCCACTATAACGAACA
TATCCAAATTGTGGATGTCTATCTAGGAATCCTATTGTTCTCTTTCCCATAGCTGTGATGTTGACACTCAAGCCGGCATCTTTTTCGACCGCCAGC
CTTGCAAACTTTGCTTTGCTCAAACCCAATTTTTGCGTTATCAGCGTTGGATTGCGACATACTGCGTAATCCCCATTGCCGATCTCGAATGTTTTT
GGTCCTACTTCGATGCGTTTCTCTTCATCCTTGTAGATACACAAATATTCAAAACCCTCAATCACGTGGGCTGCTGTCACTAAGCCTTCATCAACC
CAGAAACATTGGCCCATAACGTGATAAGTAATCCCATCCAACGATGCCATTACCATAGCCTGGAAAGTGGGCAGAACTTTAGCTACCTCAAAAGAA
CTTCCCGGCATAGCTTTCTCACTGACATAAGAGCCAGCTCCAGCCTGGCCACTAGGCACCCACAAACACACGGTGGTCTTTTGCCCACCAATTTCTC
ACCTCAACATATATATAACTCGAAAGAAGGATGACAAAACCGCAAGCTATCGCCATTAAATAGACTTTATAGTCCAATAAAAACGTTTCCAAAGTC
TCCATATGCACTTTAGAATACGTCTCTGACAACCGCTCGTGGATTATCCCTTCAATCGATGGCGCAAGGCTCCATATGTATTTTAGGAGAAATCCG
GTTTGCACGTAGAAGGATACCGCCAACATGGCTTTATTCAACGGGTTCACCACAAATGACATCATTATAGCTGACATCATTAAGGCGACTACCTGC
AGTGGTATGGAAATATATCCTACCGCGTCACTCACAGTGGTGTAAATAACATTCACAGTTTCCATCTCGTTTTACAGTTAGGAATTCGGTATTTAA
ATGAAAAGTTGGGATATCAAATCATTT

>c35604_g1_i3_len=633
AAAAACGTGGAGCTCAAGCGGCGCACCCTCATTTTGACCCCCACCCGAGTTGTTATGGAGGAAGTAAGAAAGGCCTTCGGTGAAACGGAATTCAAG
ATAGCAACAGCGAAAGCAGCCCATCACAACATCATCTATGGTGCAGCGACGAGGGCGAACTGGGCGAAATGCCCCAGGTACATATGTTTATCCAGT
TGACGCTGCTACCAAGGAGGATCCATATGACTGGGTTTGTTGGCTGGAAGCACAAATGGTGTTAGACCAGCTAAGCAGGATGTGGAGGACGGAGGC
GCATGCCTCGCAGCCGAGTGGAGCGGTTTTCCGCGGCCTCGGGGTGGAGGTGAGGAAGGGCTACCTCGAGGACCACATCGTGAACTTCCTAGGTTT
AATGCTGTGTCTGCAGGTGATTTCAAACGCCCGGCTGTGCTATACACGCATCGACCCAAAACATCCTTCCAGCAGCTATCAGCCAAGCAATAAATA
CAACACAGAATGTTTTGTCACAAATTGACTAGGTCAGGTCAGCATGGGTGCGATGGCCAACATGCGTAAGCAATTTGCATCAAGGCACTCGAGTGC
GCTACATGTTATGCTGACTGCATGTTTACTCGTTGGTGGTCGTCCGTTTGGACGATG

>c35604_g1_i6_len=804
AAAAACGTGGAGCTCAAGCGGCGCACCCTCATTTTGACCCCCACCCGAGTTGTTATGGAGGAAGTAAGAAAGGCCTTCGGTGAAACGGAATTCAAG
ATAGCAACAGCGAAAGCAGCCCATCACAACATCATCTATGGTGCAGCGACGAGGGCGAACTGGGCGAAATGCCCCAGGTACATATGTTTATCCAGT
TGACGCTGCTACCAAGGAGGATCCATATGACTGGGTTTGTTGGCTGGAAGCACAAATGGTGTTAGACCAGCTAAGCAGGATGTGGAGGACGGAGGC
GCATGCCTCGCAGCCGAGTGGAGCGGTTTTCCGCGGCCTCGGGGTGGAGGTGAGGAAGGGCTACCTCGAGGACCACATCGTGAACTTCCTAGGTTT
AATGCTGTGTCTCCAGGTGATTTCAAACGCCCGGCCACGCCAACGGCCATTGGCCTACTGGGTGTTATCCTGGTTGGCTTTTGCTGTTTTTGGATT
GCCGTCGGTCTTCTCATGCGTTGGCTTTGGTTCGGACTAACTTTTCGGAGTGGAAACCCATTGGGATCCTGGGAGAACGCGTGTGTCCCTGACGTG
CAAAATCGATTTTCCAGCATGCCAGACGGAGTCTACCGTGTAGCATATTTGATGAGCCATTAAGATGAAAGTTAACAGATATCCCTATAAAGTCAA
AACCATTCTCATTTACACTGAACCCATCTCAAGTTCAGTCATTATCCTCAATAGCGTTAAAAACATGAAACATTCCCATGAACATTCCCATGTTCA
AATACTTATAGCTTTCCCACAGACAGAAACCCTGCG
>c54922_g1_i1_len=1698
GCCGGCAGTGCTTTCGCCCAAATCACTTCGGTTTCGGTGGTCACCGCCGCACCCGCTTTACGCTGACCTTCCTGCAGCAGAGAGCTACCGCCGGTA
TACCAGGTATGATCCGCATCCGGCAGCGGCTGATCGGTCAGATCCGGACGGGTGCCATGCGCTTCGGCCAGGATATCCAGGCAGTTATGCTGCAGG
CCTTCTTCTGGCAGCGGCAGCAGGGTCGCCGGATTCAGCGCCACCACCGGGCCAAACTGCACACGATCGGTATCCAGCAGCAGTGCTTGATAATGG
GTCATACGCGCGTTAGACAGCCAACGATCCGGCGGCTGTTTCACCAGCGCTTCAACTGCGTGCGGTGCCAGGATAACCAGCGGCTGGCCCATGGTC
AGTTTGCCCGCATCTTTGGTCAGCACCGCAATCGCCGCAACCATGCGCAGACACGGCGGCCAACCCGCCGCAACCGGGTCCAGTTTTTTGCTCAGA
TACGCAACCGGACGACGCCACGGGCCCAGTTTCTGGGTCAGCACGCCTTTCGCATAGCCCTGTTTTTCATCCACGAACAGTTCAAACGGTTTGGTC
AGATCCGGCAGACCCAGCGCCGGCGCGGTCAGCAGCGCCTGTTTAATTTCCTGATACGCTTTCTGCTGATCCGGACCCCAGTTAAACAGGGTGCCG
GTTTTGGTCAGCGGATACAGCGGCGCCGCCATTTCCGCAAAGCCCGGAATCCACAGACGGCAAAAGCCCGCGGTGCCCAGAAATTCACGCAGCTGA
CGCGGGGTTTTCGGGGTCGGCTGGCCCATCACGGTTTCTTTACGCGCTTCGGTCAGCCAACGCTGGCCTTCTTTCAGCAGATAGCCCAGATATTTC
ACCTGTTTCTGGCAAATCTGCGCTTTTTTCGCGCTCGCACGATAGCCCAGGTTGCCCAGGGTCTGCAGCAGCGCACGGGTGCCCTGCTGGCAATCC
AGTTCGCTGGTCGCGGCCAGCAGCAGATCATCCACATACTGCAGCAGAATCAGATCCGGATGCTGAATACGAAAATCGGCCAGATCACGATGCAGC
GCTTCATCAAACAGGGTCGGGCTGTTTTTAAAGCCCTGCGGCAGACGGGTCCAGGTCAGCTGGCCGCTAATGCCCATTTCCGGATCACGCCATTCA
AACGCAAACAGCGGCTGGCTGGTCGGATGCAGACGCAGGCAAAAAAACGCATCTTTCAGATCCAGCACGGTATACCACTGATGGCTCGGCGGCAGG
CCAGACAGCAGGTTATACGGATTCGGCACGGTCGGATGAATATCTTCCACACGTTTGTTCACTTCACGCAGATCCTGCACCGGACGATAATCGTTG
GTGCCCGGTTTTTTCACCGGCAGCAGCGGGGTGTTCCACGGGCTCTGACACGGCACCAGAATGCCCTGATCCAGCAGACGCTGAATATGCGGTTTA
ATGCCCAGACGCGCTTCCTGGCTCATCGGATACTGTTTAATGCTCACCGGGGTGCTGGTCGCTTTCAGCGGAATAATCAGCGGCGCCTGACGAACG

GCCAGACCCATACCGCCGGTTTCCGCCCACGCCTGCGGAAAATCAGACAGCCAGGTGCTGCCCAGGCTCACATCCGGTTCTTTGCTGGTTTCATGC
AGACGATGTTCATCTTCGATGTTCAGGGTCATATGATATCTCCTTAGAATTCATCGCTGTTTCCTG

>c33969_g1_i1_len=1727
AAGGACTTCCCCTTTTAGAATATTCTATTAGGAAAAGGCTGGAAGCCGCCAACCGTATTTAAATGAGTTACGAGGCTAAGTATATGAAAACCCTTC
CCTCGAGGGAGGGATACCCAGTTGACCTAAGAAAGGCCGGTACCGATCGGATTGCCGAAGCCTTCACAAGGAAACACTTTGGTGACAAAGTAGTAG
ATGATGTGACAGGAAAGTTTCACAGGGCGACCTTAACATCAGCTGAGATGAACGACGACTTTATGAAGTTTGATCGTCGTCACGTTCCAATGATAA
AACAAGACCCTGTGTACTTTTCCATCCTCCAGACACTCCGTGAAGAGTTAGTACCTGAGAAGAAGATAATCCCCTTCACCACCGGTGGAGCGTGGG
CAGCGCCAGAATTCCCAAAGGACAAAAGTCCTGGTTTACCTTATCGTGAACTAGGATACAAGACAAAAGGGGAAGTCCTTGACCATAAGAGTAATC
GAAACGAAATCATGGCCACGTGGTATGCAGTAGGAGCAGGTAACGTGCACCACTTACCAGACACGATGATTTTCTACCGAGCACAGATCTGTAAAA
CAGATGCTAACAAAATCCGTGCAGTGTTTGGTTACCCACTAACTGTTTTTAGTGAGGAGGTGCGTTGGGTGTATCCACTCATGCGTTATCTGAAGA
CGTGTACAAGCGATTTCCCTATAGCGTACGGCCTAGAAATGGCGAATGGTGGAATGCAGTATATCAATGAAATGTGTAATCGTTCACCCCCCGGAT
CAACCTTCGTGATCAACGATTATCGCATGTTTGATAAGACCGTTCCACCCTGGCTCATACGTGATGCATTCTCTATTTTAGCAGACTGTTTCGATA
TGGGTCGTGTCGAAAGCTCAGATGGTTTAATATGGGAAGTTGATGCGGAGAAGAGCTTGCTACGCTTTAAACGCATGATGCGCTACTTCATAAATA
CCCCTGTTAGATTGAATAGTGGTGAGAGATTCATGAAGCAAGGCGGTATTCCCTCAGGTTCTGGTTGGACTAACATCGTCGGCAGCATACTAAACG
CGATCATTACAAGATATGCTGTTTACAACACAACTGGATCCCTACCCTTCGCTGACATTTACATGGGCGATGACTCCTTCGTTGTAGTTGAAGGAC
CCGTTAACCTCGAAGATTTAGCAACAGTAGTTGAAGAAAATTTCGGGGTGATATTGAATACAGATAAGAGTTGGGTGACCACATCTTCTGTTAACG
TCCACTTTCTCGGGTATTACAATCTTGACGGTGTCGCGTTTAAACCTCAAGATACTATTATTGCGTCCTTCATATACCCTGAAAGAAAGCCGGACG
ACGTTCAGACTACTTGTGCTCGTGGTCTGGGACAGTTGTGGTCATCGTTGGGAAGTAGAAGTGCTCAAAAGTGGTTGGAATTGGTCCAAGATATTA
TGGAAACACACAGCATTTCCTCCCAGTGGATTGAAGACTATCTCCACAGATACCCAAATCGATTCAGATTTTTGAAGACCTTAGGCTTAAATCCTG
TCGAGTTAGGGCTCCCGGAGCCTAGTTTGTTGGGGGTTGTTACCATTGTAGAACCCTCCCCAATCGCGCTTAGGCAGTTCAAGTCGAGGTACAGAA
ATTTGAAATATCTGTATTCACGCTACAACGAAGAACCTCTACCACAGTTGGAATTGGAGGAATAATAAATACGCGCTGGTAACGTACTACCCCCC

>c33559_g1_i2_len=374
AAATTGTAAGTCATCATTACTCAACTACAACACTGAGAAATAAATCTTAAAGCCCCAGTTTATAAGATGGCTGTTTTTTACACTTTGAATGATGTT
CGGGAGGAGATGAATTCCCTTGTCTCTAGATTATCAGATGAAGGCTTCTGGACAATCCCAAGGAACACGCTAGCCGAGTTTGAGTACCAAGGCTTA
GATGTCCTTGGCATTGTTCGTGACATTGCCAGAAGGGGGGCAGCAGCTGGAAAGTCTCGAACCGATGTCCAAGGAGACGTCAGTTAACACCACCCT
AAATAGTAACACACCAGTCAAGCACCAGCACATTCACAGTACATTCACTGCACCGGCGCGGCATCGATGATCCCAGAACCGGCAGC

>c36351_g1_i2_len=1327
CCCTTTAATAAACAATTTCAATTTTAACCTTCATTGTGTTGTGTAAAATTAAGTTGCATTGTATTTTATAACCGAATTTCAGTTAAAGCATTTAAT
CGTATGATAAGCTTTAGTCTCTAAGATAGAGGCTCCATTTACCACCAATTAATAAGTTGACTTTAAGTTTTAATTAATTTCTTATCTTTAGTTGCT
ATAAATATTATAAATATTCAAATACCCCAGTTATAAGCATAAATATCCTATAGTAAGACTAAGTTTAATAATAGAAGTGCTGATAAATTGACAAGA
TTGACTCCACCAAAATTCAGAAACCAATTTACAGAGCAATAGATACGACTGCTAGTGAATTCAAAATGGCAGAAATCAAGGATCTGAATGATGTGC
GTATGGAGATGAATGCATTAGTGGCACGGGTCTCCGAAGACTCCTTCTTAACTGTTCTACGTAGTACATTGGCTGAGTTTGAATACCAAGGACTTG
ATGTTCTCGGTATTGTTAGGGAAATTTTCCGAAGGGGAAACGCAGCTGGCCGATCAAGACCTGACATTCAGAATGACGTGAGCACTATGATCATTC
TCTTCTTAAGCAGAGGCAACAATGTGGAGAAAATGCTAACTAGAACCAATGAAACAGGGAAGACCAGAATTACTGCGCTCAGAACCATCTATAATC
TTGCAAATAGTGTGGGACGAGGAGGGAACACTGTAGTTACACTGTCAAGAGTTGCTGCAGTGTTTCCTGTTGGTACCTTGACTATCCTTGCCAACG
AAACTGTAGCAGTGCCAAGGGCTGTAACACTTAGTGTGAATGATTTTGGGACAAACTTCCCACGTCAGATGCAAACTGTGATTGCAGCGGCAGTTT
TCCCGAGAGGCACCCAAGGAACACAACTAATGAAAGCTCTACTTCTATATTTAATCGAAGAGAATAAGCTCCTGAGCACAACATCTGGCTCAACGG
ATGCTGACATTCTAGCCAAAGTAATACCCTTTGCGAGAGCGTCATTCGTTTCGAGTATAGTCCCTCAGAGCAATAGGTTCCAAGCCTGTCTAGCTT
TGAGAATGTTAAATGATCAGAATCAGTTAGCTACAGAGATAGTTTTGCCATCTAACATATTTGGACAACGATTTCCGTCTGCTGATTTGTCATTTA
TGGCTTAAATGTATCTACAGCTTTGAATTGGCTCCTCATTATCAGTCAGAAGACCAACCCTTTGTATTAGTAAGAATTTGAAATTGACAAGTGATC
AGTGTTTTAAGTTTGCAAAATTATAGGGAATTTATTAAAAGTTGAAAATCAGTATTGCTTTATTGTTTTTATTTTCCAA

>c36369_g1_i1_len=1239
AAAGTTTAGTTTTTGACAACCACACACACACAAAGACAGCCCCTTAAGTAAACACATCTAATTTTAACGAAGAAATGGCGATTTCTATAAGAGTAA
GTTTGGTACTCATGTTGATGATGAATGGAGTTAGAGCGGAAAGGAGAGAAGTCACTGTATCGATAAGTGATCTTTCAGCACTGCAGAATGATCTGC
TGAGTAATGGGATGAACAGTAGAGCAGTTGAGATGATATATGCAGCAGCGAGTTGTAATTTGAGAAGGCATGAAGACCTGGACTCAAAATTATCTA
GACTGATGGATCCCATAGAGCAGATGCATGCATCAATGAATGGTTTACTGAATGAAATGCTAAGGCGGAATGAGAATTTGAGTGAGAAAGAAGGTA
CTTTAGATTTAGTATTCAGGCATAGACTGATCACTGAGACAGAAACCATTCCACAGAAAACAATTGTAAAATCGGAGGCTGATCATAAAGTCTATC
CTGCAATAACGAATCATGATGAAGCAGAGTATCTAGCCATGGGATACAGAGAAGAAACAATAGACCCGGAGTTATGCAAGACCTATGCAGTGGTTA
AGGATTTGATTGCTCAGAGTAAAACCAGAAGACATGAAGTCTTAAAGTTAGCTCACTCCCATTACGGCTTTACAGGTCCTCATACTATTTCAGGGT
TGGCAAAATTTTATTTTGATTATGTCACAAACAATTATGATGAACGAATTAAAGTCATGGGTTTAGCCAAGATACTAGGTGAGTTTACAACACTGG
TGAGTGATGCTTACTTCTTAGAAGACGCCTTCAAGGCACATAAAGAAAAGTGTGGGTTGAATCTAGAGCATCAGAGAAAGAAAAGAAATGTCATGC
AATCAATGATCAGCGCAGGGGTATGCACAAGAAACTTTGGACCAGGTATGATCGGACCATTGGCTGAAGTCAAATTCATGTCAAAGGTTCCTTTTA
GATCAACACTAAAATCGGGCTCTTTAACCGCGCTTGGAGTGTGTTCTGAGGCTCAAACTGCTTGGTCTGGAGTTGTGGAAAAGTCACATTATAGAA
ATGCAATCAGTGCTAGTTATAAATCAGGAGTGGAAGCTTGTGTTAGAGTAGCTGTTTGTCCCTCAAATCACTCTTTCAACGGCGTTGACTGTGATA
ACACTAAGCCAATTGGAGAATTCAGCAACATTGAAAATCTAGAATTAAGCGTGATCAGGAATAGGGTCCCATTGAGTGAGCTGCCGG

>c36405_g1_i1_len=2738
AAATTTGTTTAAACACAAAGACCAGCCCCACCATTGGAAAACAAAGAAAACTAACACATATTTAGTCTCACTTGTTTTCCAATAGTTTTGGGTCTT
TGTGTTTAAACAAATTTAGTATTTTTTACCCTTCCTCTGTCTACTGAACTTTCTACTACATACAACTCTGGTTACACATTTGATTAATCCTATGTA
TAAAATGTAGACCACTGGTATGACTAGCAGAACGCCAATAACCAAGTAAAAGTACCTTAGGAAGCCAAAGTTCCCCCAGGGTATGCTAGCAAAGAA
GTCAGATCCTCTTCCAAGCAGGTTGGTTTGATTTTGGTTCTGAATTTTAATGTTGTTAATCAACTCTCCCCTCACATCTATTTCCACAACATTACC
AGAACAGCTAATAACGCATTTTTCTTCTATTTCTGATACTGAGAATGAAAGCTTAAATTTAGTCAGAGGGTTAGCTGATGTCACTAATTCTGATCC
AGTATTTAAAGCAGATGGGCACTTGACAGTAACTGGGCTCCTTACTGAATTAGTTAGTATAACTTCTACTTCTAATGTTGCTCCTGTACCACAATT
AGCACAGCCTCTCAATTTCCTAAAGTGAGCCTCGCATGTTGCCCTAATAACTGTGTACGAGATCTTATAATCTTCCATCCTTATCCTGACATTGTA
AGAAGCTTGTCCTGTGTGATGCAGATAGATGACTCCTGAGGCATCTTCTGATACCACTGACATGGTCAGATTACTAGGCAAAAGGTTATTTTTCCA
TTCTTCACTAGCATTGCTGAATCGATTGACAAAAACGACATTATCATCCTGTGCAACAACATGAATGGAATTGTAGTCAATCAAGCAAGATTTAGA
TGCCCTCTCAGCCAAGGTTGGAGTTGCACATTGAACGCCACCAATTTTACCAATTATTGGGTGATTCCTGTTAGAGCAGTCCACTATTGCAGTTAG
CATTGATCCTGACAGCTTTCTCAAAAAACATTTGTCAGTGATGGTTATAACAGGCGTATTGACAGATAATACCTGTACTTGTCCTATAATGGTTTT
AGTAGGCAAAGTGTTATCCAAGATCAATGTTTCATTTCTCACCCCATCCTTGCTTGACACAGTTATGTTCACTTTGACTTCCCAGAACCATTCAGA
ACACCTAGAAACCTCATAAGATTTCCTTTCGGTGTTTTGTAACTCAATTTTGTAAAACTGACAAGCTTGGCTAGCATAAAAACAACCCTTGCCCCA
GAAACCTGTGACTCTTGTGCAACCATGAAACCCTGCTTTATCCTTATTCGCAGATGGGACAGGCACCGGGCCTTCGGTCTTAATCTTTTCACAATT
GTCATCTTCGCATTCCCCGGCATTTCTGCATCTGAAAACATTTAGTAGCTTGAAATCTGCGTCGTTAGTGTAATAGAGCACACTCTTTGAGCACTT
TTGTCTAATAGCACTAGTTCTAAGCTTTATAATATGAACTACATTTCCAGATTGATCATTAACATTTATGCAAGACTCCTCCCCAAGAGAAGATAG

AGGCACTTCAATGATCGTATTAATAGCGCATTTGATCTCACCAGCGGTTGATGGTAAGCAACTCAACACTTTACTATCAACTGTTATCACATTTTC
ACAGGTCAAGGCACATGGTATCAAGATCAAAATGACGACAAGACTGACACTAAATGGCTTATTCATTGTTCTAACTCTAACTATTTCATCCTGTTC
ATCTGCAAATTGCGTAAGTCTTTCACTCGTTGTGCTCACCATGGATCTGGACTTCCTGGTGCATTTCTTACATAGTGCTCTAGTTATCTTGTAGGC
TAACTTGGCAGGGCTTAGTAAGACATAAAGAGCTAATTTTGCGATTCTATAAATAATGCACGCTAAAGGTATAACCAAGATTAGAGAACTAATAGC
CAGCACTAATATAACCCAGTTCACAGCCTTGTAGCAATGAGGATTGGCAAACCTAGGTAAACACAAGTCACAATGCAAGGCTAAGCATGTGTGTGA
ATCATAACACGAAACTGATAAGACCGTTTTCCCTTTGCCATCGCTTCTGAAGAACATTACTCTAAAGTCAGTGACATGTAAGAATTCTGATGGCAA
TACATAATCAAAATCTGTTTTCTGTTCGCTTATCAGCCTGCAGTTATCATGCACACACAAATGAATAATATCAAATTTTATGTCTCTTTCAACCCT
CAGAGATCTTCCTCTGCAAGAAACTTTACATCCACTACACTGTACTGATTCCTCTTCTCTAACTGGAATAGTGACATCCCAGTTAGCATGAGCAAC
AGCATCAATTCCTACTTTTGCTGATCCAATTTGCATGTATAGGTCAGAGCAATCTTCGCAGTATTTGCAAGAACAGTTTGCATTTGGCCATTTACC
AAACACACTTTCATATGCTTCATCTCCTTTACATTGATGACAATCTCTAGGACAGCCACCGTCGCAATTGAAGAAGCATAGATTTGTCACATCTTG
TCCATGAGCAACTAGAACATCCTTGTCCGTGAATAGCCATTTTCCACCAATAGAATACATCGTGAATCTTTCAGTTGACTTCACTGGCTTTTCACA
TTGCTTACCGTTAGTAACTGTTTTCCCCTGGATAGAGCAGTAAGTTTTAGACAACTCTTTTAATTCATCAGGCAGCTCGCTCAATGGAACCCTATT
CCTGATTACGCTTAATTCTAGATCTTCAATGTTGCTGAACTCTCCAATTG

>c36406_g1_i1_len=6400
AACACACACAAAGACGACGCATAAAATAAGCAAAGTTAAATTAAATCAAGATGTCCACTTTACTCAATGATATTGTTATAGACTCCTTGTCAGGAA
TTCAGGGGGATGTTGTCACTGGAGATGAAATTCTCTACCACGATAGTGTTGCAGGTATTCCTATTCCAGAAGTTCTGGTTGATGTACGTGGTGACT
TAATCGGAGTTTCATTCAGACCACTGGATGATGATGATGTGGTTTCTACGGTCGGTAGTAGCGTTTATGGTCAAAGCCTGAGTTATAACTTGGGTG
ATTGGAAGCTGAACAATCTTGTGCATGATTTTACCTTTGGAGAACTAATTGAGAACACTGATAGGAAGTTAAGTGAGATTTTCCAAAGATCAGGTG
CAGAGATCACTCGATTTACTCCAGATCATGTTAGTGAAGAACCAGATTTCAACATAATTGTTGAAGGGGGCACAAATCAAGGGAGTTTGAGATCAG
TAAAAAACTATTACGAAGAAAAGAAACAGAAATATGATGCTTTGTTGAGAACCATTCATCAAAGTGAGCTCGCAAATGAAGGGAATGTAAAAAAGA
TCCTATTCGGGATCATCATTGTTGGACATGAAGCAGTGGCTACAAACTTAGCTCTTTCAAAAGAGCAAGTAATTGAGCTGCAGATGAGATTTAGAA
TAGGTCTAAGAATTCAAAAAGAATTGGCGGAGTTAGGCTTGGTTGCTAAACTACCAGAAGATGCAACTGAAATAGATAAGAGATTAGAGCAATATT
TCCTGAAACTTGATTTACTCAGCGATAAGGAAATAGAGAACTGGAATAAATCAATAAAACGGCAAATAGACATTAACAACCCTGATAAAGATTTTC
TTGAGCTACACTTAATGAGGTGGGATGAAGATAGAATAAGAGAGCTTATAAACCATCCAGATAAGGAAGCAGCTGGCATTAAAACTGCGAAAATTC
TTCAGGAATCCCTGAGTTTTGTTGCAGAACAAACCACAAAGTATGAGTTTGACGAAGAGGCTAGAAGAGTTAAGAGCCAGTCTGCACATGCTGATT
TTGAGAAAGAGTACATATCAAGAAATGAGGGGAATATGTTCACAGCAGAGAAGGCATTCATTAACTTTCCAATGATTGTTATTAAACCATCTGAGC
CTTCCAGGGTACCCAGTAGAGGATTAAAGCTTAGCGAAGCAAAATTTGAGACTGTCTATGATACTCTATATTATGCGGGAATCCTGAAAGCAGTGG
CTGATCCCTTGTACTTCCCTGATCATGAAGCTGACATATTAGAGCAGTACAAATGGGAAACTCCGCTATTGACAAGCAAAGAGGAATTGCAGCAGT
TTAAAGATGAAAATTTACCAACGCTTTTCCAAATTGGTGGGACCTTTAGCCTTAACAACATATCTAGTAGTTTCCTGGATTCAGCGCTATCACTTG
TAACTAGTGATGTGAATAAGCAGTCTGAACTGGCGAAAGACTTTAAAGGTAAAACCTCTAGATTTAACGCTTATTTGACTGACGCAAATAGGTTCG
ATTTAGCTCTAAAAGGTGTTCAGGGTAAAGGATTAACATCAAAGTTCATAAAAGGCAAGATAAATGTATCATACAATGCATCAATTGATGAAGCCA
GAGAAAGCAAACCAGATTGGTTCCACATAACAGACACCAACACTGATGACATGGATCTATTAATGAGTGATACTGAGCTGTTTATGGAACTCACAT
CTACTCTTCATGAGACAACTACAAGCACAACTTTATGGCAAAGTCTAATCAATCAAGCAAGAGACTATCATGGTGACAACTACTTAAGGTTAAAAG
AGGAACAGCATCAAGCGTGGTTGTCAAAAACTAAACTTTTTAATTGGTGCAAACTAATCACAGACATAGGGACTGAAGTGGACATTAGCATAAAAC
AAAACTGCAAGAAGGATGAGTTCATATTTAAGAAGTTGCAAGATTTCAATGTCTGGTTACTTATAAAACCAACTAAAAAGGAGGAAAAAAATATTCT
TTTCAGTCTTATTTCGAAGAGAGGACATTGATTATCTTTATGAAAGCACAGTTTTTAAGAGATTAAACAAGCTGTTTGGAGGAGAGAATTACTTAT
ACACAAACTTTATAAGTTTAACAGAATCAAAGTTGCTAAACTGGGTGTTAGTGTTACCTAGAATGATTGGTTTATTTAGGTTTTGGAGTAATTTTT
CTAACATCGAACCCTACAGTGAACAGAACATCAGAGATTTACCAGAAACGCATCCGCACTTCATTGGGTTTTACAAGAATGCAATGCCAATGTTGA
TTCTGAGCATAATTATTGGGTTGAACGATAAAACAGAGGTTGAAGAAGAGATAACGAGAACAAGATACATGGTGATGGAAAGCTTAACAGAGTGGC
CAATTGAACCCAAACCTTACAAAATGGCTAGTAAAGCAAGCATGGCAATGAGAAGCAGGTTAACATTATGGCTGTACAGAAAACATAAAGACCTGT
GCCACAGGTACACTGCTACACCGCCTAGAGCAAGAAATGAGGATGTTACTGGTCAAGAACAAACTGAAACTAGTAACCAACTGTTCTGGGAAGGAT
TTGTTAACCCCTACACAAATTTCAAACTAACAACAGGTCAGCAGGTCATAAATTTATTCTATTTAGGGTACATAAAGAATAAAGACGAGACAGCAC
AAGCAAATAAAGCAAGCAAACTTTATGATAAAATATTGACCTATGAAGTCCAGTATGACCTTGAAACTGCTAAGATATTGGGCTTAACGTCTCCTC
AGAAGCCTAGACCACATTGCTTTGATTTGGATCTTCTCATTGATTGTGGGAGGAACCTAAACCATAGAATAAATGAGTCATATCCTGAGTTTAACT
ACAACTTTATGAAGGAACTGCATAACTTCATGTTTTTGCGCTCAGTTGAAGAAGAATTTTCAACATTAAAAGCTTCTAGCAACTTTAATGAGGAGC
TGTATGATAGGAAGAAAGTTAGCAGCAAATACCAAAGAGGTAAGGTCATTGAGAAGCTAGCACTAGAAGGAGAGGGAGCAACAAGCATTGCTGAGT
TATTTGAGAAAAAATACCTAAACGCTTTGAAGTCAAGAGGCCTTAACATAGATATATTCAGAAAGCCGCAGCATGGAGGAGATAGAGAGATCTATG
TTTTGGGGTTTCAAGAGAGAGTTGTTCAGAGACTCATAGAGCAAATCTCTAGAATAATCTGCAGGTTCATACCTGAGGAAACAATGACTCATCCAG
GAAATAAAACTGCTATCCCAGAAAATAGCTCAAGAGAGGCAAGAACTAAATATAAAATGGGACATTTAACTCTTAACAGTTCAGCAGACGCCTCGA
AGTGGAGCCAGAACAATAGCAGCTTTAAGATGATGATTTGCTTACTAATTATTTGTCCCCCTTATCTGCACAAAACTATAATCAGGTGCTTACGGT
TGTGGGAGTTCAAGCGGATCCTAATCAATCCTCAAATTTTGGAATTGTTTGATAAATATAGAAACCTAAAACTGTATGACAAAACTTTACAATGCA
TGTTTGATGGATACAAAGGACTAAAAACATTTAGATGGATACGAGCAGGGAGCCCTTATATTGAGGTAACCACAGGAATGATGCAGGGAATCTTAC
ATTATACATCATCCCTGTATCATTCAGCCATAATATCCAGAGTAAAGGAAGTGGTTCAACAAAGCAGCCATGCAATTCAGGAGACTTTGAAATATC
CAAGGCCATTCAAGCTTATAATGTGTCATTTGCAGTCTTCAGATGATTCTTACTTTAGCGTTAGTGCACCTTTAACAGGAGATGCAAGTATTGCCA
CAAAATCACGTATTTTGGCTACTGCAATACTGCAATTCAAGGTTAATTACTCATCGCATTGTGGCGTTGTGAATTCGATTAAGACTGTTTTAAACA
GCAATCATGTATTTGAGTTCAACTCAAACTTCGAGTTTGGATTCAATCATTACAAGCCTGATATAAAGGCAATATTTTCAGGTTTCCTAGTGTCTG
AGCAAGAATTGTTGCTAACTAGACAAGAAGAGTTGAGTGTGCTTTTGACCAGCTATTTGGAAAATGGAGGGACTAATTACGTTGCTAATGGCTTAC
AACTTGGCCAAAGCTACCTACACTATCATCTGATGGGTCTCACAACAACAAAGTACTTCAGAACATATGAAGTTCTGCAAACTATTCTACCAGATC
CTAGCATTGGTTTTTTTGTCATGGATAACCCAATGTGCCCTGGATTATTAGGTTTTAAGTATGGATTATGGAACTTGGTGAAGTCCTCCAATCTAG
GGAAACTGTATAAACATAAATTAAGACCCATAACCCTAACAGATGCTAAACTAGAAGAGAATGTCAAGCTTTCAATTGAATTAAGCGCACATGGTT
CCCTATCAGCATCATACAAATTGATACATGGGAATAGGAGAAAGTGGTTAGCTTTGCTGGAGAGGATGAATGTTGACCAAGAATGGCGAGAAAAAA
TACAGATGAATCCAGAAATATTATTCAGGAGGGCCCTAAATCCAGAGGAGGTTCGAGTTAAGATTTCACAGAAAATGCACTCCCCAGGTGTTTCTG
CTTCCTTAAGCAAATTAAACACAATTCCACGCATTCTTGCTGAGAGCGCATATGTCCTAAAATTGCAAGCTGTTACATCATTGAGTAACTGGATTG
ATCCCAGGCGCACTGAGTTTTCAAAGGTTACATTAATAGAGGCTATTTTAAAAGAAATGAAGGAAGTGCTAGCTGACGATGACATAAATGAACAAG
AGATGAGGATCCTATTTCCTTTCCATGAGGACTTCACTAGAAATCAACAACTGCTAAGTGGTGTTCACGTCGTTCAGATAGGTCAGTCATTTAGAG
AATATAAAAGGAAGGTCACATCAGTTGAAATAGCCACAAACAATGAATATAGCTTATTAAGTCTTCGGGGAATCTTGTTATCATTCTGGTTTGAGA
ATAACACTGATATCATGACTCCTCGATTGAGTAAAGAACATCGAGACTACATATTCACACAGCATCAGAAAGTGATTCCTTGGATAAGGAAAGACA
TAAACGAATCATTAGCAAATTCACCATTCAATGAGATGGTTGAAATGCTACTATGGCTCAATACCTTTTCAGGGAGGAGAAGGATAGTGAGACTGC
TAGGAACTCAAATTATCTCAAAGCATGGTCATAGCAGATTATTATCAGTAGTTATTAACAACTTATCATCTTCCCATAGACTAAATCTCGATAGAG
TAGAACTAGAGAGCCCAGTTGATTTAATCTCCACTGTAAGATCCAAGTTCACATTAGCTAGCTCGCTACCTGGGGATCATATTGACATCATTAAAA
GAATATTTTCATCTGCAGGAAACCAGGTCACTTTTGTTGAAGGAGCTGTTAGGTCAAAGCAAAATGACGTTGTGCTAATGTGTAAGTTGGCTAGGG
TCATTGATAAAGGCTGGAAGGAATCAAACTCTGATGAGGTCTTAAGTATTATTAATGATATGAAAAGAAATAAGCATGGGACATTAGGGTTGTTTA
CGACAAGACAGAGAGTATCACTGGCAGAAGATGGAAGTCCACGATATCATGGTTTGGGTGTGTGGGAAGGAATCATTGACGGCTATGACATAATGA
TTAGGATTAGGAACGCCATTAATAAGCCTGTAACAGTCCAGAATGTCACAGTAAACACACCACTAGCATCAGTTGAATCAATAGCTGTTAGAAAGT
TCATTGAGAACATGGGCTGGGTCATTCCATCTGATATCCATTCAGGAAGGTCCACCTTGTTCTTAAATGATAAAGGCTTGAATTCCAATTCAGGGG
TGCCAATAATAGTGGATGAAGGCTTCAATGTGAACATAGCCGAGTTTTATAAACTACAACTAACCTTAGAGTGGACAGGGAGGAACCTTAGATGCG

TTGCAAAATTACGGAAAGAAACAGGTCGAGGAGTAAATATCACAGTCCTGAGTCTGAGCCCTAAGATTCAACACTATGAATATATGGCAAAATATG
AGAAAATAATGGGTAAGGGAACACTGGGAAACCACTGGCTCGAGAATAAACCTCTTAGCCAGTCTGCAGCAGTGAGGATCATACGGTCTTTCAATG
AGCACTCTAATGATGAGAAGAATTTAATTAGAGCTAATCTCAAAGCTAAGCTGCAAAGCCAGGGAATTTCGTTTAGGAGAAAGATAGATCTAATAG
ACTTAGCTTTCATATCAAAAGAAGAGCAGGATGAACTTTTGGATGATTACATGCTATTCCAAATGCCGCTGGACCTTGGTGATTTAAACATAGTTG
ATTTCGAAGAAGTAGGTGCATCTCCTGAAATTGTTGAGTTTGAAGACTTTGAGCTGAGGAATGT

>c38870_g1_i1_len=246
ATTCTCTTCTTAAGCAGAGTTAACAATATTGATAAAATGCTTGTCAGGACAAATGAAACTGGCAAGTTGCGAATTACCAATTTGAAGACAGTCTAC
AATCTGTCCAATAGCGTGGGCCGCGGTGGAAATAACGTGATAACTCTATCAATAGTGGCGGCAGTCTTCCCTATGGCAACTCTCTCCATATTAGCC
ATAAATGGAACTTCTCTAATGAAAGCACTACTCCTTTATTTGATTGAGGAGAAT

>c125955_g1_i1_len=320
ATTGGTTCTCGTTATCTTATCAATGTTGTTTCCTCTGCTTAGAAACAGAATTATCACTGTGCTCACATCGTTTTGTATATCAATTCTGGACTTCCT
TGCGTTAGCACCTCGTCGGAAAATTTCTCTAACGATTCCGAGTACATCCAGCCCCTGATACTCGAACTCAGATAATGTACGCCACTAAAGCGTTCA
TTTCTTGTCTAACATCATTAAGATTGTTGATTTCAGCCATCTTGTATTTTGTGGCCAAAGGGTTAATAGTTCTGTAAGTTGGTCTTTGAATTTTGA
CTATCTCTAACTTATGATCAAGACGATTAAGA

>c32973_g1_i1_len=2402
TTTTTTTCTAAAAACTTTAAAGGTCGTCCAGCTCTATATCTTCATCCATGTCCTGTATGTCATAACCCACGTCCTCAACGTCGGAGTCTGACCCGG
CAACCTCCTCACACCTTGGCTTTTTACAGGGACTGTTGAAATGTCGGCCTCATCGTGAACCCTCTTATTCAACGAGATCAAGTTCAGCAAGGCAG
ACGGTAAATTGATGTTACTGGTCCGCACTGCGATGGTTCTCTGGACAGACAGCTCGAAGAACAAATCTCTCTCGAGGAAATATTCCCTGCCGAACA
AGAACACTTTCAAAACGGATCCTGGCTTGAGGTTTGGCGCTAGTTCCGCGGCACGAGGTACGCTGGTAATCTTGAAGGGTCCATCATAGTTCTCCA
GGAGGCGATATCCTCTTAAGAATATTGGTATTAAGCTGGTGAACACACCTTCCTCATACCTGGCGATCACACTCCCTGCGATGCGGAATTCCCCAT
CCACCATCGTGGCGTCAGGGGTAGTTAGGTACAGTGACAACCGCAAACTGTTGTAGAATCTAAACTCAATGTCTTGAGCAACTTCACCTGCTATCG
GCACCCTGTAGTGGCAGAGACAATACAAATATGCCAAATAGTGTCGAAATCCCTGTTGCTCCATGACCACTCTGAATGCATAACGCGCGTTCACTG
CCACTTGGATACGCTGTGTGGGTTCGACGCCTTCCATTATGCACTCATTGAGCCAGTTCCAATCGTCAGCTTGAGCTCGCCGCATCATCTCAGCGA
AATTCCCTTCATGGACATAGTAAGCAATTTTGGACCTTAACATTACAGGGGGAGACATGAAATCATTTAAGAGGCTCCTCTGGCATCTAAACTCAA
TGGGAAGCAGGGGATATATTCGCGGCTTCTGGACATTAAAGTCTTGACCACCCTTAACGTGGATTGTGGTCCCCTCTACAGTGACATACCTCTGCG
CCCTAGGAGTAGGCTCGATGTAATCCTCAAATGCTATCTTGCGGTATCCTGTATCACTCTTCCACGCTGTCTTTATATCTTCTCTTGACATCCTCA
AATACTTTGCTGGGTCCTTAGTGGCACCTTCGAATGTTTCTTCCTCCATCGCTCTGATGCTCCTAGCCTTCGAGGTGTGAAATCCGATCTTTATGT
ACACGCTGATGGCTGCCAAGGCACGCAGGAATATGTTGAAATCACATCTCTGCAACACGATTCGCGTAATTACATCTTCGTGGCGCGTGATGTTAG
CTATAAAACCTTCACCCCTTACTTGCACCTTTTCTGTCCCGATATAACTTGCATATCTTGTCCCTGCACTGTTCCGTGTGAGCTCCTGCCTCATGT
TCCCACTGATGGGCGTGAAAGTGTATCCTCTGACGACCTTTTCCACATAAATGGGAAGCCCCATCACAGCGAACAGCAGCTTCAAGTACATGTTGA
TAGTGTCAACCTCTTCATGGATGATTACGAGGAGGCTCACACCCCTGAATGTGCAGCATTGTAAAACTGCTCTTATCTCCTCTGGACTCCTTTTAT
TGTTCAACAAATACCAACATACTTTGCCAACCAGTGTCAAGATCTCCGTGTAGATGCCTTTATCTCGACTGGCAGTCACTGGAACCAGAATCAAGT
GGTTCCCAGAGGCATACACATACCTCACCCTGTTGACTGAGTGGGGGTACTCTGGAAGTGCAGGCATGTAGCGCATTTTTGGATCCATCAGGTCGA
GCAAAATCCGTAATTGGGACTGAATAGTCAATTTAGACTCGAGTCTCAAGTTACTAAGTACCTTGAGCTCCTTCACGATGGTGGGATCCAAACTCG
GGTACATGATCGAAAGGTGAGGCATTAACGCTGTCATGAGGTATTCGACACGCTGGTTATGGGGGACCTTGAATACTACCTCCTGGGTTGGCACAA
GCTGTCTCTGCAGGCAGGTCTTCCCGAATCTTACTCGTGACTCTCTCCAAGGTACCGAGTAGTGCGCCTGCACTTCTGCCTTCTTTGCCCTGAACA
AAATGTCTATGGCTTCCTTGATCCCCTCATCTGGGGTGGCCATGTTCTCTAAGAACCACGGTACCGCCTCGAGTTTGCAACGCCTCCTCCCAAACT
GATGTGCATCATCTCTATCCAGGAAATGCAAGGGGATGCCATGCTTTGCTGCTTTCGCAGAATCGACTGAAAGCGGATATTTTGTTCCTATTACCG
CAATCATGCTCTGCAGAGGCGCTGGATCCTTTAGATTCTTAGCATTTCTCTGGACGGTCTTCAAGTTGCAAACCGGGGTATCCTTGAGAATATCAA
TGGTGCCTTGATTAGCTGAAATGATCTTCTCCGCCAACGCTAAAAGGCCCTTCTTGTCAAATCCTCCTTGGTTTTCGCTAGTCATTGTTTGATGAA
AG

>c31623_g1_i1_len=3076
TGTCATACTACTTATTGATAATATTTTCTTTAATGACGGTTCACATCTTATCCCTTTATAATAGTGTATTCTTAATAATGTAGAACGATGTTTTGA
TATTATACTTTGACTCGGTTTGACTGTAAAGCCGAAATTCTGATAATGTGAGACTATATCTCTATATAATTCATTAATAGTTAATTGATTTAAATC
ATTATGCCTGAATATAATATTAATATCATCGGAATATACTAAGATCTCTTTTACATCTATAGTTGTTTCTTCTTGTAACAATCTAGTATATATTAA
GGTAATTAAAGTCCATAATGGATTCAACCATCCCTCTATTCCACCTAATTGACCATGACTATAGAATCCTTTATTTTGATAATGATAACTATAATA
TACATCTAGATTGGAGAAGTAACTTGCTATATGGACTAATTCTCTGATACCAAGTAATTTACTCATCTCATTTAATATAGGATACATATTTTTATA
ATGTATAGATTGATTGTGTCCCTCAATATCAAGCATTAAACTATAATAATTGTGTTTTAATAAACTTTGAGCCGCGGTATGCATTGTATCCTTTTT
TTCGTCATTTTCTATATTAATGAATTGACCTTTAAAATAACCCACTATATCATTCATTCTATTGCTATAATATGATAATTCGTGTTTATATGTTAA
GGTAGCACAACCAAATAGACGCCCTTCTTCCTTCTGTTCCCTTTCCTTCATACATAATCTGGTTGCTTTCTCTAGTAATAAAGGTTCACTTACTTC
TTTAGTATTTACTATTGCTAAAGACCTATTATCTACATATTGAAGAAAATGATGACTTGGATTATATTTCTCTTTTTGTATTATAGATAATAATTC
AGATTCACCATCAAAAGGATTAAAACATACATTCTTTTTAGAACAACTCTTATCTTTTGCATTAAATAGTGGATTATTATTATGACTAGTCAAATA
ATTTACCCCAAATTCTATATTTTCAAAGAAACTTAATGGTAAATTTTGATATCCTAATTCTTTCTCCTCTAATAATGCTAAAATAGTATTTCTCGT
GTGTGCTTGCATATTAGAGAATATTGGTAATGTTTTATGTTTCTATAAAAACTCTCTATAAACATCTTTCTAAACATTAATGTAGCTCTATGTAT
AGCATCATTATCAGTATTTCTTTCACTATGTACTCTTTTTAAGTATTTTTCACAACCTTTATCTTCATTGATCTCACTATAATATAAGAATTTATG
TATAGCTGATAATTCAATTAATTGAGGACCATCTAATTTATTTAATTCAGATATAATTTGTATAAAGATATTTTTAGAAATAATCTTTATACCTAG
GTATGAACAGATAATTAAATCTATTTCAATCTTACTTAAGAAATCTAATTCATATATCACTTTAATTAATGGCTCCCAATTTATAATAGCACTTTC
TTTAAAATCTGACTTCATAAGGCATAAAGTTTCAAAAGTTTTCATAAATTTAACTTTAACCTTATAGTTTATATCTCTATCTATTACTAAATTAAT
TATCAAATGAAGGATAGGATTTAGATAAGTATATTCACCGGTGAAGATTATAGATTTTATATTTACCAAAATTTCACAAGCACTTATTAAATAATC
AAAATGGGCATTATCCATAAAATAAAGTTTATCATTGTCAATCATTGTAAAATAGCCATCACTATTTATTATTAATCGATTATCATGTACTTTCAA
TAGAAAATTAACACCATTTCTCAAACTAACTACTTCAAATGCTGAATTGTGAATTAAAAAATTATCTATGTTAGGTTTATCTAATGGTTTCTTCTC
TTTATGTATCATATTCCTATATCTTCTAAAATCACTTACTAATTTAGAAGATCTAAATATGTCTTTACTACCTGTATTATCTGGTAATATTAGTTT
TTCATGACTTTTACTAACTGTATTAATATCAATATTCATTAATGTTTCTGTCATTTTAATATGAGATTTTAGTTCAAATAGATTTGTATTAGGATC
AGAACGTATAATCTCTGTAAATGAATCTATTGTAATGTGTGGTATACTACATAAAATTTTACAAAAATTAGTTCGCTGTTGCTTATCTATTTTTTG
ACAAATTTCTAAAACATATCCTACATGCATAGTGACATCTTTTACATGACAAGGTCTTATATTTTCAAAGTTTATATTATGTCTACCAGCTCTCAT
TAAATAACTTGAATAAATATTCCTTAAATCAATACTCATTGGAAATAAGGGACTATTTAATCGACTAGGTTTATGTAAATTTATTCTTGATCGATA
TTTATTTGTTTCATAAAAGTCTATTAAATCATTTAATGATTTAAACTCAGTTATTAATTTATATTCAGCTATATGATTAGATACATCTATATATTT
CTGAGTATAACAGGCTTTTGGTATACTATAATTAACTTCATTCGATTCAATTTTATTGTTTGTAATATAATCAATAATTGTTATTATTTTATACAA
CAATTCTATTGTTTCTGGTTCAAATAATTTTTGATATTTCTTTATCAAATATTTGCAGATTCTTATTTGTATTAAATCAGATTCATTTATATCTTG
ATTAGCATTCATTAATTTAGAAAAAGAATTTATTAAATCTAAATTAGTTATATCTTTAATTATTGTAATACCTAAATATAAATTCTTAATGCCTCT
CAATAGTTTCTCATCTCTATCTTTTGCATTACTAATTTGATAATCTTTTATTTTATTAAGTATTGAATTCATATAATTATTTATATATAATTTAAGAG
TTCTCTTTGATTCATTGTCAAAGTTTTTTAATTTATTAAATTGAAATTGATATTCACTACATAAATAACACTTTAATATATATTAAAAATATCGG
TAAATTCTAATAAATATGATAATATTATATTAGTATTATAATTTAATAAAATAATTTATTATCAGCTAATATACCACTAATTAATATATTAATAAT

TTAATATTTTTTAACATTAATGAACCATGTATTGTAAGTTTATAGTCTAGTTGTTTATTTTTCTATTCCTTTTTATTCTTTTGCCTTTATGAGTTT
TATG

>c12670_g1_i1_len=1492
AATAAGTAGTATGACAGATAATTACTTTGTAAATGATGAGATTGACAGCATAGCAATAGATTCTTCAACTAGCTCAGCATTAGAAGAGACCAATAA
AATAAAGACTATATTATTTATAAAATTCTTTAAATTGATAGAAATTAATATTTATAGTTTTTTGTTATTTTTAAAGACAGAAAAAAGAAATAATAT
ATTGAGACCTTACTATCCAGATGATTTAATGACATTAGTAAATGAAGAGAATTTTATCTTAGAAAATATTCACAATGAAAAATTCTTGAGAGAAAC
ACTAACATATATGCAAACTAGATTTCCATATTATCAAATAGATGAAATGTTTAAACCATCCCATCATATACATTTCAGATTTGCTGATTATTTAAG
ATCTATATCATCAAATATGATAATAATGGTAGCATTCTACTTATCGTTAAGCTTGCCTAGTCAACTTGGTGGTTATAATATAAGTACATTATCTTC
AGTACTCTTAACCGGACATTCATCTAGTTTTATAAGAAGATTAGATGATATATTGTTTATGCTTAAATATACAGACATTAGCTTCGATGAAAGAAC
CTTATTTATGAACATAATATTCGGTAATATAACTAAAGAAAAATTAACTAATATAGATGACGAAATTAGAGTCATAAATTCTGAATATCCTATATT
TACTGATTATGATCATGCTTTAAATATATTAAAAGACAACATTTTAAACAAGTTAAAAACTAAAAGTGTAAATTTAGAATTTAATAAATACCTAGG
TACAAAAGATAGTAAGCAAAAATATTTAAAGGAATGTTTACAATTGACAAGAGAAAATTTTTCACATCGTATTACTAATTATTATATAGAACACTC
TATATTTATGATAGGTGATATGTTATACAATAAAATAGATAATTCAAGAAGTTTTATTAAGAAATTGAAATTATATTATGGTATGTTTTATAAATT
ATTTAGTAACAATATAGTTAGCCATTATAGATTATTTAATGTACAAGTTCGTCAAGATTTAAAATATGAAATCGAATCTTTAATTTTAGATATGGC
AGATGTACAAACTGTATTTGACAATTTACTAATGAATAAGTATCCAAATATTAAATTCAATAATATTAAGGAACCATTATTTAATAATAGGCTTAG
AGATTATAAAATAGAAAATAACTTATTTACTATTAAGAAAGTAAGTTCTACAATATGGAAAAATGATATGAAGGTATTTAAGACTATGAATATCCC
TTTAGGATTAAGAATAAAATATTCGTCAGAAGAATTAGTCAATAAACAAGTAAATATAGAGAATAGATTAGTATTAAATAGTTGTTATATAACTAA
GTGGATATTACAAAATGATGAAGTTTTTTATCACGAAGATAATAATTATGTTAAATTATGTAATTTGACTATTAGTTCAATGACTAAATTTAAATA
TAATGAACTTATGGATTACTTAACTATACCAATAGGTGGTGAAATATTTCAT

>c35704_g1_i1_len=7947
AAAAAATTAAAAAAATAAGAAATATTCGTAGACTAATAAACAGTAAATATAGATCAACTGTTATACTAAAATATTTCAAAAATATTTAGTAAATAA
ATATTCATTAATTTATTGATTTATATCTTATAATATTATAGTTTTTTTAATAATAAATTAATGAATTTATAATAACATAAATTCTAATTTATAATT
AATATTTCATAATTTATTATAATATACTATTCAATAATCTACTTCCGCAACATTATTGACTATTAATTATTTATATATTAATAGGTATATTATGAT
AAAATATAAAATAAACATTACATTAAATCTTCATCATCTTCTACATATTGATCCAATGGTTCATCATCACTATAATCAATTGGTTCAGTATTTTCC
ATTACTGACATTGCTTTGCTTAAAATATTTTTAGTTTGATTGACTATTCCTGCAGTTACTTTATTCGGTATTATATTAATCGGTAATCTAATTGTA
CCATCATTTGTTAAAACATTACGTTCTTCTGAAAATGCACTTAACATTGATGTAATTAATAAATTACCAATACCATTGACTGTCTCATCTTCAGGA
TCTGGAGTCTCTAATATTTTCTTCTTTCTCTGTTTTAATGATGCAATATTATTAAATTTTTGCTTTTCAATTAATCTTTCATATATTTCAGATATT
TTTAATAATTGTTCATTATTTGGATTGAATAATTCTTTTGCTTTATCTATTAAATTACTAGAGAACCTTATTAATTTAATAAATCCTAAAAATTTT
GCTCTATAAATAGCTCTTTGAGATAATAATTTATAATGAAATATTAATTCACTCAGAACATCATCATACGAGCTAACAAAAGCACACTGTAGTATT
AATATTATATTCTCATATCCTGAAGCAGACATGATTCTTGTATAATAATTCTCATCATCTATGAATCTATTATAGTTCTCTATTATATCAAAAGTG
AATGTCTTAATATTATTTGATAGGCAATACTCCAAATAAACATTCTTGAACTTATTCATTAATTCAGTTAATAATGGGTATTTACAAAATTGAATA
AATTGATCTAATTTATCTAATTTTAACTCCCTTAATTTACATACTCTTACAAATTTGCATTCATTTAATAAAACAGAAATATACTTTTCTTTAGTT
TTATCTTCTAATATAAAATCACTATGAACAACCATATCTATATATGTAATATAATCTTCATCCATATTATGTGTATCTTCAAAACATTCCATAATC
AATTTATTATTAGTTGGTATATATTCATAATATTTAATATTTTCTGCAAAAAAGTTAACTGAATTAATATACTCATAATTGGTTATGTAACCTTCT
ATATTATTTATTAATCTATTAAATTCTATTTCTTCTAAAGATATTTTAGAATTTACTGAATTGTAACTTTCATAAACCATTTTTGCTTTATCAAAT
TTATAAAATGACTTCATAGTAAAAAAAGTGATTATATTTACTAATCTTTGATTTCTTATATTTAGAACTGGCATCATTCTTATAGATTTGTATAAT
TCTATATAGATATGGTAAGGTTGTAAACTTGATTTAGTAGGTATTAGAAAATTCATTTCAGTAAATCCCTTCAATTTATTTATTATTCTATTAAAT
ACTTTATTACTAAAACTGTTAATTCTTAATATTACTATCTTATCTCTTGCTATAAGATTATCTAAAGTGTCTATGATATTTAAGGGATCACCTTTA
ATAAAAGATATATCTAAATAAATAATATCATACATCTCTGTAAAGCTTAACGTTTCATAAGATAAAATATCATAATCCTCTGTAATTATTCCCTCT
ATCTTAAATATATCTGTATAAAAACTCCTTATATTATAACTTGTGCAATTCATATTTAATTTCTTAAAAGCTAAATGGCCATCTCCTCTGCCAGCA
GTCAAGTTACATATTGATTTATTATTATAATATTTATTTATGAATTTACCTATTCCTAAATTTGCAGTTATACTATCAGATCCTGTTAATGAATAA
AAAGACTCTATCTTAGAATTTAAGTATGTTATTGGCCTAAAATCACTTATCATCTTATATATACTAGACATATATTCTCTAACAGAATGATCTATA
GGGTACCTAATATATTCTATAGAATTCACTAACTCATTTGGTATCATATCTTCATTAATAAATTTATCTATCTTAATTTTATAATTCTGTCTATCA
TCCCTATAATTTTTCATGATTATACCATTCATTTCTACAATTTTAGATCTAATATAAATCATTTCTTTTTCTATAAAATTTCTTAATTCATGAACT
CCAATTATATTTATCAAATTATAAATAGTATCATTACTAAATTGTATCATTTCACCATTCAAAAATGCTCTCAATAATCTAATTGTCCTATCTATC
ATTATTTTAATTCCGAATTTTGCTCTTTGATCAAAACATAGGCAAAACTTTATTATTATTTCTAATACTATAGCTTTATATAATTGATGTCTAACT
GAATATTCTGTTGATCCTAAATGCTGTCTAATTTCTCGTGTTATATAGCCTTCTTTTTGGTTTATCATTAACTTATCTAATTTACTTAATTGTTTA
TTTTTAGATTGTCTCCTTAGATTTTCTATCTTTTTGGTATTTGTTTTCATAATATCTAATACTTGTTTCATTCTTTTCATTACTTGATCTTCTGTT
GAACTTAAATTTAATATTTCAAAGATGGATTCCATATTAACTAATCTCAAATTTCTTAATTCTTTATAATAATCTCTTATAGTATAATTGACTTTC
TCAATGAATTCATTAAAATTCAAATCTATTAAATTACTGTAATTATATTGCATTGTTTCAAAGACTTTCTTCCAAACTTCATTGCTTGATATATTG
AAATCTAACCAAACTTGATTTTGAGTTAAATTATTATATACATTAATTACATCTTTAGTGAATCGTCTATATAAATAATCTTCAGTTGTTTCTTTT
AATCTAATCAATACTTCATTATTTGATAGTATTTTATTTATTGTAGAAAATTGTTCCATAGACTCAATTCTATATATGCTCATAAGAATTCCTTCA
TATTTAAAGTTACTTATATAAGTTGTATTATATGACAGTTTAATTCTAACATTTTTCACTATATTTTGAAAATGCCTAAAAGAATATTCTTTCATT
ATTATACTTTTATTAAAGTAATATTCATCTATAACGGCTCTTAATCTGATCATATCTTTAACAAATGAAAAGTTTATATTACTATCAGTATAATTC
TTTATAATACCAGTCTGAGTCAAATTGCAAGTGTAAGTAACTAATAAATGATGTAATGATTTTATATAACTCTTATTTTTAAAAGCAAAATTATCC
AATCTATGAAATATTTCACCACCTATTGGAATTGTTAGATAATCCATTAATTCAACATATTTAAAAGTAGTCATTGACGATATGGTTAAATCACAT
AATTTTATATAATTATTATTCGAATCATAATGTAATTCATCATTTTGAAGAATCCATTTTGTTATATAACAATTATTTAAAACTAATCTGTTTTCA
ATATTTACTTGCTTATTAACTAATTCTTCTGTAGCATATTTTATTCTCAAACCTATAGGTACATTCATAGTCTTATATACTTCTATATTATCTTTC
CATATAGTTGGATTTACTCTCTTTAACTTGAATAAAGGATTCTCTATATTGTAATCTCTTAATCTTGTATTATAAATAGGTTCTTTGATTTCATTG
AAAGTTACTTTATAATATTTCTTTGCTAAAATATTATTAAATACTGTCTGTATATCACACATATCTAATATTAAACCTTCTATCAATTTTATATTA
TCTTTCCTTGGTATTATATTGAATAATCTATAATGACTAACTATATTATAACTAAATAATTTATAAAATAAACTATAATGTAATTTTAATTTCTTT
ACGAAAGTTCTAGAATTATCTATTTTATTATATAACATGTCTCCAATCATGAATATTGAATGTTCTATATAATAATTAGTTATCCTATGAGAGAAA
TTTTTCCTAGTTAATTCCATACATTCATTTAAATATAATTGCTTACTTTTTCTAGTACTTAAGTATTTATTGAATTCTAAATTAACACTCTTATTT
CTTAATTTAGTTAGAATATTATCTTTAATAACAGTACTTGTTGTATCATAGACACTCATGATAGGATATTCATTTGTTATTAATCTAGTTTCTTCT
TCAATTCCATATACTATATCACTATTTATATTACCGAATAAGATATGAAGAAATAGTATTCTATCATCTAATTTTAATTCAATATGTTTTAACATA
TAAATTATATCATCTAATTTCCTTATGAAACTAGAAGAATGTCCAGTTAATAATGATGATGATAAAGTACTAATATTATAACCTCCAAATTGGTTC
GGTAAAGTTAAAGATAAATAAAATGCAACCATCATTGATTTATTATTAGCTATTGATCTTATATAATCATGGTATCTATATTGTATTTGATGGGAA
GGTTTAAATAATTCTTCTAATCTATAATATGGATATCTGAATTTCATATATTCATATGATTGTCTAACTACTTCTTTATCATTTGCATGTTGTAAT
AAATAATTTTCATCATTGATTAAGGCCACCAATTCATCTGGATATAAACTTCTCAGAGCAGTTCTACTCTTTTCTGTATATAAATATAATAAAAAT
CCATATAAGTTAATTTCTGTTAATTTAAAGAATTTTATGAAATTAATTGTCTTAATTTTATTGCTTTCTTCTAAAGCGGAATTAGTAGCTGAATCC
ACTGATGTACTATCTATTTCATCATTAACAAAATAATTATCTGTCATACTACTTATTGATAATATCTTCTTTAAAGAAGGCTCACAACGTAGACCT
TTATAATAATGAATTCTTAATAGAGTGGATCTATTTTTAGATATTATACTCTGACTTGGTTTTACTGTAAATCCAAATTTTTGATAATGAGCTACT
ACATCTCTATACAATTCATTTATAGTAATCTGATTTAAATCATTATGTCTAAATATTATATTTATATCATCAGAATATACTAATATATCCTTTACA
TCTATTGTAGTTTCTTCTTGTAACATTTTCGTATATATTAAAGTTATTAATGTCCATAATGGATTTAGCCATCCTTCGATACCACCTAATTGACCA
TGACTATAAAATCCTTTATTTTCATAATTATAATTATAATAAACATCCAAATTTGAAAAATAACTAGCAATATTTACTATCTCCTTTATACCTAAT

AATTTACCAATTTCATTCAATATAGGATACATATTTTTGTAGTGTATAGACTGATTATGACCTTCTATATCTAACATTAAACTATAGTGATCTCTC
TTCAGTAGACTTTGAGCTGCATTATGCATACTATCCTTTTTATCACTATTATCTATATTAATGAATTGTCCTTTAAAATAACCAACTATATCATTC
ATTCTCTCACTATAATATGATAATTCATGTTTGTAATTCAAAGTTGCACAACCAAATAATCTGCCTTCCTCTTTCTGTTCTCTCTCTTTCATACAT
AATTTTGTAGCTTTCTCTAAATTAATTGGTACACTAACTGATTTTGTTTCTAATATTGCTAAGAATCTATCATCTATATATCTTAGGAAATTAGTA
CTTGGTGTATAAGTTTCTTTATGAATAATAGACATTAATTCTGATTCACCATCAAATGAACAGAACCTAACATCACTTTTAGAACAACTTTTATCT
TTAGCATTAAATAATGGATTGTTATTATGACTGGTTAAATAATTAATTGAAAAATCTATATCATTAAAAAAATTCAAAGGCAAATTTTGATATCCT
AGTTCTTTTTCATCTAATAATGCTAACATAGTATTCTTAATTGAAGGATCTAAATTATTGAATAAAGGTAAAACTTTATGTTTCCTATAAAAACTC
TCTATAAATAATTTACGAAACATTAGTGTAGATCTATGAATAGCATCATCATCACACTTTCTTTTACTATGTACTCTTTTTAGATATTTTTCACAA
CCTTTCTTTTCATTTATTTCACTATAATATAGAAACTTATGTATAGCTGATAATTCTATTAATTGAGGACCTGTTAAAAGGTTTAATTGAGCAATT
ATTTTCACAAATATATTATTACTTACTACATCCATACCTAGATAAGTGCAAATTACTAAATTCATATCTTTATTATTTAAAAAATCTAATCCTTGT
ATGACTTTAATTAATGGTTCCCAATTAATTATCATACTATCATTAAAATCTGATTTCATTAAACATAAAGTCTCAAAAGTTTTCATGAAATTGACT
TTATGTTTATATTGTATTTTTTGATCATTTACTAAATCTATAATTAAAGCTAATATTGGATTTAAATATGGGTATTCGTTCATAAATATTATAGAT
TTAATATTTACTAAAATTTCACAAGCACTTACTAAATAATCAAAATGACTATTATCCATAAAATATAATTTATTATTTATTATTAATGTAAAAAAA
CCATCGCTATTAATGAATAACTGATTACTTTGGGCTTTTAATAAGAAATTAACTCCATTCTTTAAACTAACTACTTCAAAATTAGAGTTTCCAATC
ATAAATGTATCTATATCTGGTTTGTCAATTATTTTCTTCTCTTTATGTATCATATTTCTAAATCGTCTACAATCATTCAATAACTTAGATGTTCTA
AATATATTTTTATATCCTGCATTATCAGGAAGGTGTATAGTTTCATGACTTTTACTAACAGTCTTGATATCAATATTCATTAAACTTTCAGTAGTT
TTAATTAAGGATTTAAGTGTAAATAAATCCTCAGATGGATCTTTATTAATGATATCACTAAAAGAGTCTATATTCGTATTAGGATAATTACTTAAT
ATATTAGTAAATTTTGTTCTCTGTTGTTTATCTATTGAATTGCATATATCTAAGACATATCCAGCATGCATAGCCACGTTCTTAATATGATTTTCT
CTTACAGCATTGAAATTTATATTTACACCACTAGATCTTATATGATATTTTATGTAGATTGATTTTAAGTTTTGATCCATCGGATATAATGGACTA
TTTAATCGACTGGGTTTATGTAAGTTAATAATTGATCTATATTTATTTTTCTCATAATATTCTATTACATCGTTAATAGTTTTAAAATCTATAATT
TCTTTATATGTATTTATATGTTCACTTATTTCTATATATTTATTGCTAATAGTTAATTGAGGAATTTCATAAATAACTTCGTTAGGTGTTATATCA
TTATTTTTGATATAATCAGTTATTGTAATAATCTTATATAATTTATCAATAATATCATTTTCTAATATTTTAGATCTTGATCTTATCAATTCTTTA
GCTAATTTAGCTTGAGGTAATAGGCTATATTTTTCATCAATAACTTCCAATATTAAGTCTTTTGATTCCTTGATTAATTCATCAACATTAGGGTAC
TTCAATTGATGACTATTTATCTTTAAATTATTAATATATCTATGAAATTTCTCTAATTTCAAAGGATCATCATCTTTATCATAAACATTTAATTTC
TTAATTATTTGTATTAAATATTCATTAATATAACTAATTAGGCCCTCACAATTCATTTTACAATGGTTTTTTTAATTTAAATAAATAATGTAAATA
TATTATAATTAAATTGATTTTTAATCAAATAATTTATATAATAAAATTCAATAATTAATAATCTAATAATTCAATATAATGATAATAATATCTCAT
CCTGATTTATATTTTATCTTATATTATATTAATAGATTTTTATTCTAAAAATTTATTTTTTATGGCCTTTTGATTAAAATATTTTAATTTATTTAT
ATTTTGCGTGTGTTGTTTATCAACATACGTATTATCAAAAATAAAAAACACATTTGAAAAAATTAAAAAAATAAG

>c26658_g1_i1_len=2239
AAATAAAACAATTATTAAATAATTAATAAAATATTAATCAATAAATCATGATTTATAATATCATGAATTTTGATATTCTGAATATCAGAAAACAAG
TTTTTTTAAATACGTCATTAATGGAAAAATGAATGCCTAAAATCTAATATCTTAAATAAATTATTGACTAAATTGATCCTTATCTTAAAATTGTTT
TCACAAATTTTAAGACTATTAAATAATTAAAATTTAATAGGTTCAAAACGTTAATAATATATTAATGATATATTTATTTTAGTATTAGAAATCTTC
TTCTTCGTAACAATCCAATTCTTCATCATCTTCATAAAATTCTGGTGGAAGATTAATTGTGAATCCCATATCAAAGAAACTTTCAGATTGTTGAGT
AACTTGTTTTAATATTGGTGCTTGACTAATAGACACTGGTGTTTTATTAGGTTTTATTGGAATCATAGATTGGTTATTATCTCCAAACATATTTGA
AAAAGCACTTCTCATTGAATCAAATATCATCCTTTCTATATTATCCAATATAGAATTAGAATTATCTGGTGTCGATTGAATTGCTTTCTTCCTTTC
TCTTAAATTAGCTATATTATTAAATCTATGTTTTTCTATTAATCTATGATAAATATTATTGATTTTTTCCTTTTCTATTGATTTAGGTACAAATAA
TTCTTTTGATTTATTTATTAAATTACTTGCTAATCTAAAAGACTTTATAAATCCTAAGAATTTTGCTCGAAATATTGCTCTTTGGGATAATAACTT
GTAATGGTATATAAGATCAGTCAATACATCATCATAACTCGTAACGAATGCTGCTTGGAATATCAATATTATTGTTTCATAACCACTAGCAGACAT
GATTCTAGTATAATAATTTTCATCATCAATTAATTTGTTAAAGTCTTCTATTATATCATAAGAGAATATTCTTATATTATTTGCTAAACAATATTC
AATATATACATCTCTGAACTTCATCATTAATTCTTTCAAAAGTGGATAACTACATATTTGAATAAAATCATCCAATTTATCTAACTTTAATTCACT
CAAATAACATATTTTAACATATTGTCTTTCATTAATTAATACACTTTGATATAACTCTTTAGTTTTATTGATCATAATGAAATCTACTTCTGTAAC
CATTTCTATACAACTTATTAATTGATCGTCATAACGATGATTGGCGATAAAGCACTCCATAAATTTTGGATTATTTGTTGGGATTTTATCATAATA
TTTTATGTATTCCGAAAAGAAGTTGACTGCATTTATGTGTTCATAATTGTCCATATAGCCTTCCATATTAAATAATAATTTGTCAAAGCTTATTTC
TGTTAATGACAATTTGCTATTAACTGAATTATTACTTTCGTATGTCATTATAGACTTATCAAATTTATAAAAAGATTTAGAAGTGAAAAAGGTTAT
TAAATTGAGTAATCGTTGATTTTTAACTGCTGGTAGCATATCTATTTTCAAACATTTATATAATTCTATATAAATATGATAAGGTTGTAAGCTTAA
TTTGGTTGGTAATAAAAAATTCATTTCAGTAAATCCTTTTAGTTTGTTTACTATCCTTTCGAATGATCCTTTATTAAAACTATTTATTCTTAATAT
AACTATTTTATCCTTTGCAATGAAATTATCTAATGTATTAATTAATTTATCTTGATCACCTTTGATGAAAGATAAATCCATGAATATAATATCATG
AAATTCAGTAAAAGTCAATGTTTCATAATTTAATACGTCGTAATCCTCTGTAATAATTCCATTTATTTTATATATATCAGTATAAAAGCTCTTTAT
ATTATAACTAACAAAATTTAGGTTGACTTTTTGAAAAGCTAGGTGACCATCACCTCGGCCTGCAGTTAAATTACAAACACTCATATGAGGATAATA
TTTATTTATGAAATTACCGATTCCTATATTAGCTGTTATACTATCTGAACCAGTTAGAGAATAAAATGATTCTAATTTTGAAGTCAAATAAGTTAT
CGGACGATAATTTGATATCATGTCTTGTATAACATATAATGATTGCCTCACTGAGTTGTCTATAGGATATCTAATATATTCTATGGAAGTTATTAA
TTCATCAGGTATAATTCTATAGTCTATGTCTTTTTCTATATTAATTTTATAATTTTGCCTATCATCTTTATAATTCTTCATTATAATTGTATTCAT
TTCAACTATTTTAGATCTAATATAAATCATT

>c40444_g1_i1_len=345
GTCAAAAGGTATCGGTTTAAAATTTTTTGAAGAAAACTGATAAACAATTTATAAATATGATTACATACCTACCGAAAACAGAAACTTCAAACCTCC
TAAATATTATGACAGAAAACTTAAAGAAATCAATCCAGAACTATTAAAAGAAATAAAAAAACATAGAAGAGAAATTTTCACACAAAATTTAGAAAA
TGAAATAATTTCCCAACAAATATTATTAACAGAAATCATGGGAAATAAACAAAGACGGCTTCATAACAACTTAAAAAAAAATTCTCAACCATTGAG
AAACGAAAACTAAACATTTGAAACCAAATATAATTCTGATGGCTGTTTTTATGTTGC

>c35196_g1_i1_len=2187
CTTAAACCAAAAATAGGGCAACCCCAAGGAGATCCGAACTATCCAACCAATGAGTCTCTGAGCTGCTTACCCATGTACAGAGCAAACGGGGAATTT
ATCAGGCATTCATTAAGAGCATCAGCTAAACCTTTGACATCACATCCAATCACTTTTTGCCCTCTGCTCCACTCAAGTTTCATCATGAAGATCTTC
CTGATGATAGCCATGGCACCCTCCTCTTGAGAGCCCCCTATCGCAGCCATCATGACACTCTCGGCGCACCTCTCCATGAGCCATTCGCCATGCTCT
AACAAGTAGGTTTCCATCGCCAGGCCAGCATTGTAGTCCGTTCTATTGTGCGGATTGTTCAAGGTCAACTCCCCTAACATGTTCGCCGGGAGGTAA
GATGAGTTGATTACAAAGGTCACATATGAGAGGGCTCCCTTCATCACAGAATTCACGCGGTAGCACCATGCTCTTCCCCTGGTGTCTCTCACCACA
TGAGCATTCTTGATGAATTTCCTATACCTCACGCCATCATCTCCGTTCAACATTTCCACGGTGATAATGGGGATCTTGTCAGTGGCACTTCTTGCG
TGTTGGGGGCCCCGAATGATAAAACCAGATAACGCTCTCCAAGTTTCCCCATCCTTCTCACCTACGCTGTATATTGGGAAAACCACAGCGTCTGGA
CGGTCTTTAGAGGAGGTAAATTTTCCGGAATATGCCCCACTAAAGCGGGAGTACACGCTCTTCAATTTACTGCAGTACGACCCAATAGTTGAGTGG
CCAAACAGATTGAAATATTTGTTGGCGATGGGTTCAATTATCAAGTTCATTGGATGGTCATCTTGCATTTCTTGCTGCTGCAACTCTAAGAAGAAA
TTCATTCCCAGTTTGTTGGGTTCTGACAATTCCTTTAACAGGTTTGACATCCAAGCCGAATAGCGCGCTGGTTGGATAGGTTTAAACTCCTTCTGC
TTTAGGTTAACATTGCCATCATCGCGCACGGTCCCTTTGCACCCTATGCCCAAGTCTCTTTGCAGCTCTATAGGCATCCCAGTCCTATCTTTGATT
GTGGAGAGGCAAAATGTATTCCCTTCAGATGATTGCCACTTATTCACTATCCAGTTCAGCACATGGGACTTTTCCTCGGTTGCAGCAAACTGTAGA
TCCTCAAACAACAGGCTGATCATTTCTGAGTCCTTGTCAAGCTGTGTCATGACATGGTGAACCAATGGATCTGGGAGGACTTTTCCAGGGAATTTC
ATAAATTCTGCGTCCCTCTTGCTTGTGTCCTCTATGAAGCCTTTCAAATCCTTCTCCAGAACCCAGTCCACTTTCTCTGCGTTCATTATCTTCTCA
TACCCCACAGCTGCATTGTCCTTGTGATCCCAGAACAGTTTTGTCCAGTCTGCCACGCACTTGTTGAATCTCTCACAGACAAAGATCGACTTAACC

AAGTCTTTCGATGTGACGAGGTCAGACTCCATGTGATTGAATTGGGCACCCATCTTCGATGCTCTTTCCAACAGAAACTGCTTTGCCTTTCCCAAC
CCAGGCATTGGATCGCGTTTCCCTTTGGTTCTGCAATCTGCTGACACCGGATGAAGGTGGACCAGTGCAATATACTGATCTGGATCCATGTAATAG
TCAAACTCATCTCGGGAAGCCTGGTAGTTTAGAGTGACTTTGACCTCAATGAATTTTCTTTCGACCTTATCATATATATCGTATTGACGCCTCATG
GGTTCTATGCCTAAAGCAGTGCATATTTTGTGTTGGAGCTGAGCTACATTAGACCTTCCCTCCAGAAGCAGATAACGGTACACGTCCTCATCACCT
TTCACTTCTGCAACCACATCTGGGTCTCTGGGCAGCTCACGGGAACTGACACCTCTGTCCGATGCGCTTGAACTGGAGGTACTCGCTTCACTTTCA
TCACATCTTCTCCTTTTCAACGGTGGGGGGTTCAAATCTTCTTCGAGATCGAGAGGCCTGAGGCTTTCATCGACTTCCAGATTGCACAATAGCAGG
CACACCATGTCATGTCTCAAACTAGCTTCACGCTGGAGCCACGTATCGCTTCCCCAGTGCGGTCCAGATGATCCAAACGTTTTGATGAACTCCGGG
CTGTACAACACCCCTGAGTTGCACAATGTTTCCACGTCATTTGGTTCAGCCATTTGTATGTTTATTGAAAGAGAG

>c124296_g1_i1_len=280
ATGTTGGACTCTCCGGGTATGTTTGAATTTCCGGATGTCAGCCAAATCCTCAACAAATGGAAGCCTACGGATGGTCCGTGGTTTAAGGAATACAAC
CACCTGGAAATTCCGGTCGGAGATTGGGATAGAGTGAGCCATGCCGAGAGATCTCGTCACATAGGCATAGCGCAAGGATTAATGTTCGGGTTGCTC
CATCACAGTTACCATGACACATTTGTGCTGAAGGGGTTATTCCCGATCGCTCTCCGGAACAAAGTGCAAGGTCCTGCGTATCTTTTGG

>c28080_g1_i1_len=284
AGCTGAGCGAGCCATCGCCCTCAATAGCCAGATCAACCATCTAGTCAGAAGAGGTTTCACCAGCGCAGGCGCTGATCTGATTTGGCACGCCGTCGT
GCCTTACGCAGCTCGAGCTAAGTTGGTACATGGAGCCCTAACCATCCCGGTGGCGTACCTTATGCTACCCTTTCTGGACCATGGACTGGACTTAGG
ACCGCCTATGACGGCTGCAAAGCGCGCAACACCAGTCACTCCCATTCCAACGATGGATCTGTGTAGCCGAGCCTTAGAGGAGTGCGTGCCCA

>c29461_g1_i1_len=238
ATGTTGCTGCTGTGCCAAGGCGTCAGCTAGACTTTCGAACACTGCGCCCTGTGCTTCCAGAGTGTGCTGGTAGTTGAAGTCAGCATAGTCAACCAT
TGTACATTCAACTCCTGGCTTACTCACCACCTGTAGACGCCTCACCATGGTCGCGATGGCATCGAACCCAGTTAACCCGCTCTCAACTCCATCGAT
GTTGTAAAGGTGCTCTTCGACACCATCCATCACATAGGCGACTATG

>c33519_g1_i1_len=502
GAGGGCGATGGCTCGCTCAGCTGGACTAACGACATCCGTGTTCTGGATAGGCTTCATTATCAGCGTGCCTATCGCACGGGCCAGATAGCCCCTGCA
GGCACCGGAGACGTACACTACGCGTAAGAATTCGCCCAGATTCACGTCGAACAGTTGTTTTGCGGGCTGGAACACGAGCCCGGAGCTCTCTAAGAC
CCTGTAAAGGCCGATAGCCCATAGGCGAGACTGGTTCGATATCCAGACGTCATCTCCTTGGTGTATGTTGTGCAATTCCACAGATTGCAGGTGCAG
TTCATGTTGGAGCCAAGTCTCAGCGACTTTGAAATACCCTAGATTCAGGAGCGTGTTGATGAAGTTGGTCGCCCGTATGCCCGAGAACATTCCTTG
TGTCACCTTCGTCATTTTGCCTTTGTCCTGCGGGAACTTGCAGTATTGTTCCAGCAGTGCTGCCGCTACCCAGCGCGAAGCCCGCACCTTATCTGG
GTGATGTTGCTGCTGTGCCAAG

>c34550_g1_i1_len=820
CACGAACCCCATCCCGCCTGTCGGGGCGGAGGTCGTCACCTACGCGCAATTACGCGCCTTCATCATCAAGCTGGTACGGCTTGTGGGGACTATGGA
CGCTGCGGTGAGGGGTTGCACCATAGGCCAGACCCTGGTGAACGTGTCAGTAGCATACCCGGACCGAGATCCGGCTATCATGGTCATGAGCACGAT
CGAGACAGAGCGGACGCGCCTGCCGCGAGTCCACGGCCGCAACGTCGTCTGGACACTGCTAACCAGCCTCTGGGTGACCAGTGTGGACGAAAGACT
GTTCGCCAGGGAGTGGGATACGCTCCAACAGCTAACAGCCGACGAACACGTGCGTCTGGGAGCGGCAGTCGCAGGCGTAGTTTCCTTAGGGTACTC
GACCTGCTTGAACCATTTCAACATCATTGGGCGGAACCTGAACTCATGGGCGCGACGGGAGGTCCAGTTGGTGACATCATTCCTGGACGGGCTGTT
GACAGCCAGACCGGGACAAAATCTAAATGCCATTAGCACAATGGCAATTAACATCATCGAGGCAATGACCGGCCTCTCTATCCACATGTCCACCTT
TGTCTGCCTAGCTTGGTGTAGCAATTTTAACCACAGACCCAATCTACCCCTGACGGAAGGTTGGCGAGACATCTGGGGGTATGAGATACCCTATAT
TGTACGACCTGAGAGCATGGAGTGGGCTATCACAACCTGGATCGCAGAGTGGGGCCTGAACGGGCCCAACCCCAAGTTCAACTTGAGCCACGAGAC
GATGGTGTACGGCAGCCGCGGATCCCGGGGCATGTACATCTACTTCGGAAGC

>c36064_g1_i1_len=887
CCAGCCAGCACACCCTGTCCGCTCCTCCAGGCTGGCTGGACTACCGTCCTCCCAGCGCCCCGTCCGCCGAATGGCGGTGCTGATGTGTTTAAACAG
GGCCGCATAGTCATCCATGTCGAGGCAACAAAATTTATACCTCTCCAGGAGGCTGAACATCTTGTCGGCCTGCTCGTTACCAATCATATGCACTAG
CAGTGCGCAGATCGTGATGTTGTAAAGGCCGGTGGAGAATAGGTGGTTCCAACGGTCGTACTTCTCACGGTAGGTAGGGTGGCGGCTTAGGTGCCA
AACCAGGTCGGCAGCAGATACATTCGTCTTGGTGTGATCGGCCTCACTGGACCATGGCCACAGTTCGTTGAGTATGGCAAGGTCCCGCCTATTGCG
TAGTGTGCTCCAATTGATCATCACGAATTTACCTCGGTGGCGTTTGCATTGTTTGATTCGCACGACATGTTGGTTATATTTGGCCATGACTTGGTC
CACGTGATCTGGGGGCGTCCGCTTACATGAGTACTGTGTGACCGTCTTCCAGAGACGCATACATTGGGCGCTGACGCCAAGTTGGTAGTTGCCACC
GTTAATGCTCATCCATTCGTCGAACATCTCTTTGGGCATAGTCTTGACGTCGTGCAGATGTTGCACTTCCTTGAGTGTTGTGAGCAGGCACAGATG
TGGTTTGGTGAGCTTCTTATGGACGATGGGGGATGTTGGGTCTTCAGGGTCTATACGTACGTACATCTTGGGTGTTCTGTCTTGAGATTTGGAAAT
GGCTATACACGTCTTGCACTGGAGCAGCCCCCGTTCTAGGTTGAAATGGTTTTGATGGGATGGGTGGACGGACACGTTGTCAAGCTCCCTCGTTGG
CCGCTCACCTAGTCGGCCCTCTA

>c36389_g2_i1_len=882
CGCGGTCAGTAATCCATCCAAGAATGATGTCACCAACTGGACTTCCCGTCGCGCCCACGCATTTAGGTTCCGCCCTATGATGTTGAAATGGTTGAA
GCAGGTCGAGTATCCTAAAGATACGACGCCTGCAACCGCTGCTCCTAGGCGCACATGTTCGTCGGCAGTTAGCTGTTGGAGCGTGTCCCACTCCCT
GGCGAAAAGTCTCTCGTCCATATTGGTTACCCAGAGGCTGGTTAGCAACGTCCAAACGACGTTACGACCGTGGACCCGCGGTAAGCGCGTCCGTTC
AGTCTCGATCGTGCTCATAACCATAATGGCTGGGTCTCGATCTGGGTAAGCCACTGACACGTTCACCAAGGTCTGGCCTATGGTGCAGCCTCTCAC
AGCGGCATCCATAGTGCCCACAAGCCGAACGAGCTTGATGATGAAGGCCCGTAATTGTGCGTAGGTGACGACTTCTGCTCCGACAGGCGGGATGGG
GTTCGTGTCCCAAATGCACCAGCGTATGCGGGGTAGGTCTATGTATGTGTGGATAGGCATCATACGGCGGGCAGCTAGTTGGACATAACCGGTACC
CTGAGCTATCATCATCATACAGTTCACGTCGGCAGTGGTCAGTTCTGATCGATGTAGACATACTCGACCAGCGTTGATGGCTTCGACGAGAGTTAC
GTACTCGGCATCCACTTGTGCGTCAGCACACCCGATGTTGCATAGGGTGACGCGGTCAGCGGGTACAGCTGCGGGCGCCCACCCAATGTCGTTTCT
CTCGAGGTAGGCTAAATAGTCCAGGAGGATATAGGCAGACGCGAATAGGGTGACGTTGTCATACGACAACGCCTCCTCACTTAGGAGCGGCACTAT
CCTGTTGGCCATCGAGTT

>c36389_g2_i2_len=456
ATCGCTCGTGATTTGCCCATCTCAAACTTTTCGCTAGCCGTGCCGATTATCTTGGGCTCGTCCTCAAGCCATGATACCATTTCTTCTTTCGAAACC
GAGTCGAAATAGGCCTGCTTGTTCATGCGTATCCGCTCCCCATCGGGCATGACTAGACGCTCCCCTCCTGTACTACCAGAGGCCACCCACCTTTGT
CGGTTCTCCACAAAGTCCACCCAACTCTCCTTGCGACCCGGCGGTCGTACCAATTGTTTCGTAATCAGGTCTAGTTCCCTCCGGAGGGCCACTATG
TACTCGGCGTTAGTCGTCGCGCTTTTATCCTGCGCAGTGGGGGCTCCTAAGGGAACCACCATCTGCGTCCGTTTCCGCTTTTCATCCGCCCAATCC
GATTTGTTGCCTGACCGCCCAATAGCGAGCTCCCAGCCAGCACACCCTGTCCGCTCTTCCAGGCTGGCTGGA

>c36389_g2_i3_len=677
TTAGGACACCCTTATTCGAATTCAAGCGGGTGCCCTCGAACAACTGCTACGCCCGAGCCTTGCTCTAGGGCGCAGCATATGTACCCGCGATGGGGT
TAAGTCGTCCACAACGTGTCCCTCAACCCTAATAGTGGCTGATCATATGAAAAATCGGGAACTTTCTCAACGACCTGACGTATTTGTCCATCTCTT

GGACAACAACTTCTCTGATATCGGCGTTTCTCCAGCGCTCCTTTGCGGTCATCTGGCCAATGACGTAGTCTAACGCCTGATCTTGAACCCAAGATA
AAATCGTGGGATGGAATTCACCCTCGTACTTGGTGGAGCCGGTTCTGACGCCGTCTAGCACCAATCGGGCCCCGCATTCACCAACCTTATGCTTCA
ATGCGGCCAACAGGGCAGCGGCCGCGCTGCTCAGCTGAATCTTCGGGCACGATGTAATCGCTACCTCTGCAGCTGACAGCGCGTCCAACCCTGTTG
CCGTCATCGTGTTGCTCAGGCTTCGGAATGGACTGAGCCCAATTGCGGCCATAACAACGCCCATCCTCGACTTAACCACGGGTGTCACCTTATCCA
TGTCTCCAGAGCTAAGTCTGGTTAGCCACACCTCGAACAGCGGATTAGCCGTCTCACCCCCTTTCATCATCTCGTATTCGGCGTGAGTGCGCGTGA
CCGGC

>c914_g1_i1_len=836
ATGGCAGTTCGACTACCATCCAATAATGTATGGCTCAACGGAATCAGCTTCATCTCCGGTATCCTACTCGCCATATGTCGTCGAGGAAACTCCACA
GATGAGCTCAGAAACCGTGTGGAGCAAGGAGTTCGCGACGCCACCTCAGCTGCGTCTGTCAACCTCGGACCTGCAACCCTTAAGAGATTCTCCACG
ATCTATTGCTCGCATATTGATGCCACCAAAGCCGGACAACTATTCGCTCACTTCCAAGCGCTCATCCCCGACAACATCATCGCTCTGCGCAACTTG
GTTATTCAAGCTGCTGGTTCTGGCCTTACCACGTACATGGTCATTTTGCGCGCCATGACCGATTTCCCTGACTTCAACTGGGCACGGCTGATGAAC
ATCGTAGGTCCAGAGTTCCGAAAGTTGGATCTGGCTGTCACCGCGGTAGCATTATTTCGGACTTAATCATCAAATGGCCGCAGCATCTGCCAAGAA
TTTCCCGAACTTGGCTTACGCAAGCTTCCAGCTTTGTATCAGAGCGGATGGTGATAATCCACTTAGAAGATACGGAGGAATGCCAACAGTCATCCC
ATCGAAATTGATCATCGATCAAATGCTCACTGAATATCTCGAATTGCGAGCCGGCGCAGCAGCTGCAGCTGAAGTACCAGCTGAGATCCAGGATGA
ATGGGCAGATTTGGCTAACAGAGTAGCCGGAGCACACCAAGGTTTAATCGGAGGTGCTTAAAAGACTCCGCAGAGCGCCAGAGCAATGGCTCCATA
TGACCCTTAGGCGCTAGGGTTACATACTACAGTACCAAGCAGTTTAGTGAACAGTCATGCAGCTACCA

>c15947_g1_i1_len=1115
AAAGTAGATAAGGCGCTAATGGAGACTAAACTCACATTAGCACGCCTTAACCCAACCGAATTTGTAACTAGCATTATTAAAAGATCGGGATATACT
GCCGTAGTAGCAGGAGAAGTGCTGCACATCTTGGAATGCAAACCAGTTTACGTAACACCTCGATCTGATGATACTTGTTATCAAGAAATACCTGTA
AATTATAATAATCAAACTATGTTCATTGCCCCAGTAACTCATATTTTACAAAAAAGAGGTACGCAAATTGATTGCACACCCCTTCTACCAGCCAAA
TTTCAAATAGTTGGAAGATGGTATACAACTGATGGTCGTATCAGAGAAGCCATCTCACCGTTGAAACTCACAACCGATTTATTAACGACTTGGTCA
TATACACATCTACCAAACTTAATGCAAAGCGGAGTATATGATGCTGATAGCGTAGAGAAAATGAGAAACATGATTTACGAGCAAGGAGATAGACGA
GTTGCATCGAATGTGTTGCACAAAATATTAATTGGACAACAACCTGAATTTCAAGGCTTCCGTTTCGAAGCACTAGTTTCAAATAAAGTAATCGAA
AGCATAATAGAAAAATATTGGAATAAGCTAATGTCATGGTCAACCCGTTTAGGCAATATAACCTCAACATTTATAGGATTGTACATGATTGGTAGA
ATCATTAAATTTCTGATTGATACCATAATGCATGGACGCATATTGTACGACATATACGGTCTAGGATGGCAACTACTTGCGTCATTCTGGGATTCT
TTGACTAACTTTCTGTCTCACAGAAACGCTATGAAAAGAACAGCCGGACAAGAAAATAATTTACAACCCGAAAAACAAGAGGATGAAGTACTTCAA
GTAGATGAAGAAGAGATTTATACTCAAATCGCACGTCCACACCCAACTCCATACTCGCGGACTAGTACCCAGGTATGAGTAAATCTAGATCGATTT
ACGGGCTCCGGAATATCGCCGCGAGTAATTAATCAACAAGAAAAATAACCCCAGCCATAGAAACCAAATTCCCGACAGCCCATCGAAGCATAGTCG
ATGTCGATACCAAAATGAGAAAATGAAATAAAAAGCAGAGCGCAAAACTAGGACCTCCG

>c35613_g1_i1_len=2049
CTTGTTTAGTTGTCGAGCTCCAGCTAAGAGGTTGTCACTCCTTCGAAAATCTACCCTTATACACCTGTACCTTCCATTCTTCCAGACTTTGGGCGT
GAGTTCTCTTGTCACTTCTCACCGAACTATAGAAGTGCCAACCCTCACGCAGTCCTGGATTAAAGGATTTCCCAACTCCCCAAGTCTATTAAACTT
GAAAGGAAAATAGGAAAGATTCCTTATTTACGATTCACTCTCTAGCGGAAAACGAAAAGAAAAACGGAGAAGAACACCGGCGTCGACCGTCAAAAC
CTTCGCGAGTAATAAGTAAAACGGTCGTCACAGGTCGGACTTCGACTTAGACAATGCGGCTTATACTGCAGTGAGAATGAGCTAATCGAGGGCTGT
GAACAATTCATGGCCCCTATGGTCACTGGTATACTTACTGGCATGCTCGCATTCTTGCTGACTGCATATATATGGAGGAAATGGCTTTCATCAGCT
ATCACATCCGGGTGGGCCAAAATCCAAAGTAGGTACTATAGAAGGAGGTTTAGGAACGAAGAGAAAAAACTGACAGAATAGCTATGAGGTTGAGAC
ACATGAGGGCACCATCTCTAACTATCATGGCCATCCTTACTTTCATCTTTTCGACGGCATTAGGATGTGACAATACATTGTACATTGGTTCCAAAG
GAGTAATCTGCGACAACACACACTGCATTGAGTCCAGCATGTTCGACCTAACAATAGAAACAGGATCCACATTGTGCTTCAGAGATCAAAAAGGTG
ACCTGATCACATTATCTACGACAACATCATATAGGGTTGTGCGAAGCATGCTTATTTACCACACTTCGGACTATGAACTGAGCACTGAGCAGCTCT
GGCAATGCAAAGGTGCAGGCATTTGCTGGAAGGGTGGTTGCCACCAGTACTCCATGCATCCCAGCCTTAAGAAGAACCCCAAAGCAGGGTCTATTG
CAAGATACGGGTGTGACATTGACGTAATTGGCTGTGACACATACTGTCATTATGGGGTGTCTTGTGTATATTATAGATGGCAGGTTGACCCAATTG
GTGCACCAGTCCCGGTATACAAGGCAGTAACAAAATACTGGGGGCTGGATCTTACAGTAACTTATGGAAATCAGACTAGAGTGTACCAAGTGAATG
TCAACAATCCCAAATTTAACCTTGGAGAAGTGGACAAAACCGCATTTCCCATGTTAGTAACAGGGTTCAAGGCCCAAGAGGATGACAATGAAAAAT
TCTACATAGTCGCAAATGGGAAAGTGTACAACGTTGATGCTTCTAGACAAAACATGCCAGAATGGGGTAAACTTGGTGACTATCAATTAGATACTA
GAAACAACACTGTGGCAATGAACACACAAGATGTCAGATGCACCACACATTCATGCCGGACAGTCTGTGTCATGCCAGAACCAAAATTAGAAGGTT
CCTACATGGCAACAATCATGTTGACAGCATTCAGTCAAAATTCGTCGGGAGCAATGCTATAATGCTAACAACACAGAAGGTAAATGATATAGCCAA
GTTGTTAATAGGCAATGTTGAGATCAATTCTCTGAGAGTTGCACCTGCCAAGTGTGCACTAGATGTTATTGGTACATTTTCATGCAGCGGCTGTGC
AGTCAAGCCATACATCATAGTACAAGCATCTGAAATATCTGAGCCTGGACTAGTCCCGTTCACCTCAAACTGCACATTTAGCACTTCGAGCATTGG
CTGTTCGATGGAACCAATGAAGCTTGAATTAAATGACATGAATAACAATTGTAAAATATTCTTCCCAAGTCTTAACAGAAGTATCACAGTAGAAAT
GAAATACAATTATCTAGGCCAGCTTGATCCAACCAGGGAGATTGTGTCACGTGGAATAGATGTGTATGTATGATTTGTATAGCTTGACCATGAACC
AAAATACTGGAACCTTATGTCTTTGTTTACCTCGACGATATCGTCGTGGTAACGGAGACATTTGAGCATCACGTCCAGCTACTTCATGAAACTGCT
CGCAGACTAAACAGAGCCAACCTTACAATAAAT

>c35946_g1_i1_len=2434
AACACCTCCTTTCAACAAAACATCGAAATGGCAGAACCAGATCCTTTCACGGAAATCCCAAGTTCCTTCTATGCGGCAGTCTTTGACCAAACGAGG
AGGACTGACATCAAACTACCCGACCAGCGTGACATGGACACGTTAGGCATGGTTAGTGCGTTGTATTTGTATACGAACCCCCCTCCAAATGGCACG
GGCACTCCAGCACCAAAGGTGGCGGAAACCGTATTGAGGGCCATCAACTTCAATCGGAAGGCGGAAGACAGGGAGGCTGTCATAGACGGTTACGTG
ATTGACAATCCAGTGTGGACAAAGCAGAACGGCTTCCCGTTTGACGAGATATCCAGCAACTTCTGCCCGGGCAGCCTCCATAGAATGTGCTGTGCT
TTCCTGCAAGAGAACTTCGTGGCCATACGGAAAACGGCAGAGGACGTGATAGGCAAGATGTGCGTTTTGAACTCGGATACGCTTACGAAGGGTCGT
CAAACATGGGACGCCTTTGAAGAAAGAAGTGTTCCTTGCCCCCAAGCTTACATCAACGTCAACAAGCTATTCAAGGACAACGGGTTACCCGACCAC
CATACGGTTATGGAGCTCATTCAAGGCTTCTTCCGCTTGATGAGCATGCCAGAAGTGGTAGGCCACGAAGCTGAATACTACTACGCCGAGAAGATG
AAATACGTGGATGGACAGTTAATTGCTACAAAGATAAGGAAGAAGAGGGTTGTCAAGGTGACATGGAGGGGTGAGCAGGTGTACCGCAAAATCATG
GATGCAGGGCGATCTTTCTGTTCTTACTTGAAGCACGGAGAAAGAGCTCATTTGAATAGAAGAGCAATTGCTTCAACGGGAATATTCCAGAGAGCA
TTGTTTCTAGTGCTTGAAGAATTCCATCTTGCTTTGGGCAAAGTTATACGGGGAAGCACGATCTCCATAGGTGGGGAATTGAAGAAGATAAAGATC
TCGTCGATCACAAGCGAATGTAAAGGTGATCTGAATGCCGTACATGCTCTTCAAGCCACGCAGGACGCTACTAAGTGGAATGAGTGTCTCTCCGCG
ATGTCATTCGGAATGCTCACTAAGACGCTCTTTGATAAGGAGGTCAGGTTCGAGCAGAGATTGCTAATACCAACCCCATATGAAGAACTCCTCCGT
GAAGTTGGCATGGCTTCGCACTGGCTCCTAGCAACGAAAATGATCACTCTAGGTGATGGGCTGCAGTGTCACACTCCTCACACTCATGGCACTGTC
CAGTTTGATGCTGACAAGGTTCACCAATTCAACACCCAAACTCAGTCATGGCTCAAAGAGGCGATCCCCCTGCGTTATGGCAACAATTACATCCGT
GCGCCTGGCGGGATGCTGATGGGTATGATGAATGCTTTGTCTACCACGTACGGATTGGTCAATGTGGGATGGAACAAACCGATGCTAGGCAACATT
TACACTCTCAGGTCAAGCGACGACAGTATGACAATATACACTGGACCAGACATGGATGTGATCAAGACACTGGTAATTGGAGAGAAGATTAATCTG
AAGATGTGTGGGATAAATTTAAGCGACAAGAAAACCCAGTACTTCCGGCTTGGTGTAGGGGAATACACTTCTTGGTATCAAGATGGCGTGTTGGTG
AGTCAATATGGTGCAGAGGCCACGCGGATCCGACCAATGGGGATAAATCCGCCTGATGACTTTTACAATGTTGCAAAGTCAACTTCTGTAAGCCTT
ATGAACATCGAAAGCAATCACCTCGGAGCAGAAGCAAAGATCCGCATTGGAATCACAAATGTCAGGAGCTTGTACCGTGTGAACAAAAGGGGAGAC
GTGGAAGGAGGCATACGGAAAGAAATCAGAGTGCTCGCTGATGGAGGGTGCAACCCGTGGAATAGCACCAATTGTCACTTGGAAGAGACCAGTCTC

AAAGAGAGATTTGCAGTACAGGACAATGAGGTGGAGTATTTCCTGAAGATCAGAAATCCGGACAACCCGTTCACAGGTGAGCCAACTGAGGAGGTC
ATGTGGGACAGAAACACTGGGTGCCTGACTGTGGAGAGAGTTGATACACCGCGCACCATCTTCCACTACATCAAACGTGCCAACGCCACAATCTCC
AATGTGAGGACCCCTAACCACTCTCATGTGGAGAAAGATAACGCAAGAGCTCTAGGAATACTGACAACTGCGGACCCCACCATGCATTTGAGAACA
GCTGGGAGCTCCCACAACATGCTTTCTCATGTTTCCGGGATCTTTCAAACGGCAGCCTCTGGTCTTGAATGGGGTGAGAACGAGAGAAGGTTAATT
GAGCAAGCAATCGCAGTCCTCACTACTGGCAAGGCACATGATGATGATGACATGGAGGAAATTCAACTTGATGACGATCTTTAGAGCAAATCTGGT
TTACACGTTTAGATGTTCAAGAAAAAGGAGGTTG

>c1882_g1_i2_len=472
GTCTTCGACTGTCTGGGTATGACCTCTGCAAGCAGCTGGCGATCCCGGCTATGCTTGAGTTTATCGAAGAGTATCTCCAGTTGCGCCGTTTCTCTG
ATTCCAAGTGGATATTCTGCAGACTTCTTAGTGAGACTGCCTTACTCGGTTTATCAGAAAATAAGAGGCCAGTCCCCACCTGCACATTCGTGGTCT
TGTCCACGTCTTCTCTGAGTTTGGAGGAAGAAATGTGGGAGATTATCTCCCTTTCTCGGATAACTAAGATGGATCTTGCAATGGGGTTCACAATCG
CCAGCGCATACCTCTCTACATATCAGAAGAGGATGTGGGACCAACCCATCCTTGAAGAGGCCCAGACCCTGACTGCAACCCTCCAAGAAACAGGAA
ATGAAACTCTTCAGTGGTCAACCAAGAAACTAATCCGGAAGCAAGGACAAGGTTCAAGGACCTTTGGAATCTATGACAAAAGGTCGAA

>c64714_g1_i1_len=477
ATTTTCCAGGTGAATTGCAGCTCCCTCTACCCGCAGAACAAGCATCATGGCCTGCTTCCGCACTTTTGGGGAATTTGAATGATTTTAAAGAAGTGA
CAATAAAGGAATTAGTTAATCTGGAGGCTGAGGCTTGTAAGAAGCGCCCTATCAATGAAAGACCCTACTTAACTCAGTATTTAGGCCACCAATTAT
TTTCTTCTACTCAAAAGAAAGGAGTTCTTTACTATAAACTTGCTTTTCCAGTGAAACTACTCGTCTTTAATCACTCTGGTCCTATCGACGGTTCAA
TTGAAAATTGAGTACTCCCTGGATGACAAGAGTTATTCCAAGGGGTGGCTACTCTGCGGATCTGGAATTATTCTTGACAAATGTCCAGAGAAAACT
GCAGATGTGATAAACTGTTATCTTCCTTTACTAAAACATGGAGGTATCGACCTATGTACTGGGTCTCTTGCCCTCTCCTTTCCTCCTTCCCTA

>c18481_g1_i1_len=324
TGTAGCAAAGGCAATGACCACAAATGGTATCATACCAGACTCCGTCTTGTGAAGAGTCCGAATCAGAAAAGAGCCTTGTTTATGAGGGCCCGGGGA
CACTAGTCAAGGATGTCAACGGGAATACATCGGTAGAGTATATCCATGTTCACTATTCCGGGTATGACTTCCAAGTCCTGTTGAAATCGAGACAAG
AACTTATCTGCGGATATCGGTCTTTTTATACAGAGCACCCAAGTCTCTTCGTCACTCTTCTCTCCACTAGCGGGCCCAAATTTCCGCTCAAAAGGA
AGGTTACAGCAGTGGATGTCAGTCTTTTGAATTTCG

>c25987_g1_i1_len=1033
CAGACAAGCAAGCTCAAACATTAGCAAAAGCATTTGTTGAGTACTTTATTTTCGACTGCAAGTCCAAGACAGTTAACTTGACTGTTCTCTCATTGG
TAACCAAACCACCCCAAATTGCGAAGTAGCACACAAGAACATAACCTACGAGCAAGTAAACCTAGCAGTGACGCAAACAACTGAGCGATACGAGCT
CCCATTCTAACGCTGTCATGTGGAGTCTAGAAGTGTAATGGGGAGATGTGGAAAGTCTATTGATACATTTCATCATGCCGGATTGTTCTCCGAGAT
TGTCCGAATCGGAAGACACGAGTGTGACACTATGCATAAGAAGAAGGTGTTCCGTATAGTTAAGGATGGCGCCCTTTGCGACATCCAGCTGAGTTC
ACAGGGACAGACTAGGTATGCATATGTAAGCCGTGGATCAGTGACCTCTGATGGATCCTGCACCCCCGAAGCCGACATGATGAGGAATGGCCGAGA
GTACGATCGCCCCCTTATCAACACAACACTAACTATAACAATCTCGTCAGGGACCGCCATCGTCTCAGTTGAGGAGAGTCTAATAAAGTTCCCCAA
CGGAAATAACTGTAAACTCCAAGACGAGACATGTTTTGACGCAGATTACGGCGATGTTTTCTGGACGGTTTCAACTCCATCCTGTGAGGGATCGGA
GTCTGAGAAAAGCCTTGTGTACGAGGGCCCCGGGACGCTGGTGCGAGATGTCGCAGGAAACACCTCTATTGAATACGTTCATGTACACTATGCGGG
GTACGATTTCCAAGTGCTCTTAAAGAACAGACAAGAGATAATTTGCGGATACAGGTCATTTTATAACGAGCATCCGAGTCTGTTTGTTACCTTATT
ATCTTCTAACGGTCCCAAGTTCCCACTGCGTAGGAAGGTAACCGCAGTCGACGTGCGTCTTCTCAACTTCGTGAATTCTAAGCTGGTTTATTCATT
CCGCCATGTACGACAAGAAGTAAGCAAACTTTTTGACCTGTTCAATCACGATCGGTGTCAAGCACATAACCGG

>c32243_g1_i1_len=318
GTTACCACTATACATCAGCATTCACCCCTCTAGATTACGATAGCATTAGGTCCAAAGTCGGAGGCTTCTGGGACAGATTTACAGACTCAGCTGTAA
GGGCAGGGGGATGGTTTGGCTTCATCCTAACGTCAGTGATCAATTGCATGCATGTGAGACATGATGTTGGAGTCCTGTGGGCGATACCGATCTGCC
TTTTCGATGTTCTTTGTAACTTGGTCTTCCACGGGAGGATATGGAAGGGAAATGTCCCCACTGACGGGACAGAGCTTGAAGAGATATATCTCCCTA
ATGAATAAATTCGGCCATAGCCAATGCTTT

>c32243_g1_i2_len=356
GTTACCACTATACATCAGCATTCACCCCTCTAGATTACGATAGCATTAGGTCCAAAGTCGGAGGCTTCTGGGACAGATTTACAGACTCAGCTGTAA
GGGCAGGGGGATGGTTTGGCTTCATCCTAATTGTTTTTGATATTTACAAATTCTGCCTGTATGTCCTAACGTCAGTGATCAATTTCATGCATGTGA
GACATGATGTTGGAGTCCTGTGGGCGATACCGATCTGCCTTTTCGATGTTCTTTGTAACTTTGTAACCACGGGAGGATATGGAAGGGAAATGTCCC
CACTGACGGGACAGAGCTTGAAGAGATATATCTCCCTAATGAATAAATTCGGCCATAGCCAATGCTTT

>c36378_g1_i1_len=12872
TGCACCGCAAAAACATAAAAACCATAGGTTCTGAATTAGTAGTGGCGGGCTTAGTATCACTTATATACTTGAACCTAGTACGGTGTCAACTTGAAT
AAACCCGCTAGTTAGAAATCATACGATATTTCGCAGATTTCGAACATCTTAAACTGTGACACAAGATGGCAAAGGTTGTAGACGAGCGATATCTCC
AAGAGATAAGCCTAATTGCACCGATCAGGGAAGACAACTTTACAAATTACGAGTTCACGGAGGTTATCTACCGAAAGGGAGCGGCATCATACTTGC
TGAACATTGGGACTCATAAGGAGACAGAAGAAAGGGCTGTGCCAGGATACGATCACCAATCCCTTTGCGCGGCACTCGGGAAAATCTGCCCATTCA
AGGGAGAGCGATCTATCCCTAAGGGTGTATCTGATGCATCAGTTACTATCCTGAACCCGCAGAGAATTAGAGAACTATCGCAAGTTCTTGCTGCAT
TGCCTAAGATAAAGCTCTTTGATGAAAATGGAGGGATCAACATCTCTATATTGCTGGCACTCCTGCGTTGGATTCCCGGCATGGATGGTGTCCACC
TCTCATATCTGGCTGCGCGAGATGAGGGGGTAGCCATGGTCGAAAGCGATCTGGTCTTCAGGGTCATGGTTGCTGTGCATCCCGAATTTGATGGAA
CGAGCCCCACCTCTTCGTACGCGAGGCATACAACTGCCACAATGATCTGCGCCGCATATGCTGTGCTTTTGATGGTTATCAAGACCATTAACGCCA
ATGCAGTGAATCAGCTCGACATCTTTATTGATCGCAGAATACGGGCTCTCTGTCATACTCTCGGTCTCTCAGGTAAGTCCATCGACTGGGAGAACA
AGATCAAGCCCTTGCTTGACGAAGAAGAACTGCGCAAATTAAACGAGGAGTTAGGGTTCTTCCCACGCTTGAAGAAGGTCATCTTTGTTCCTGTCA
TAGATATGCTGACTCCTGAACTGCAGCATATGAATTTAATATTTCAGGAAACATCGATGACCATATTCTCGCTAATAGCCGAGTTTTGGATGACGT
CTGACATAACAAGACTACATGTCTCTCCGCTCGTGCTTGTGGAGTTGCCGAAATGGCAGAAAACCATGCATCAACTTGTGGCCTTGTACGGTACAT
CGTGGAGGTTTTACAAATTGATTGACCCAAGAGGGACACTCACTGCCCAGAGCAACTTCCGTACGTTGGGGTGCGCTGCTCTATCATGGAAGAGAC
TCAACACTATCCAGTCCGGCCAAACGTCCTTGGCTCAATTACAGGGGGTCAAAATCAACCCTGAATTCGAGAAACTGGCATCCATAAAGTTGAAGC
CGGAGTTTGTGGGCACCAATGCAAAATCGTACTTGGAAATTGTCAAGGGTCTCCTGCAGGACCAGTCCCCGTACATCAATATGAATTGGTCCAGGA
TCGTGAAGGCTATTTCAGATGGGGAGTGGGTAGAAGGCAGAGACTTCTAAGATTGTCCACAGCCTAGGGTCCACTTCCATATCAGGAAATGCACAG
AAGAGGCCATATACATTAGCCACCTGAGGTATATATCCATACCTCCACACCGACCTATCGGCGAAATTTCATAATAATAAAAAACCCGCTAGTTAC
AAATCATGTCAATCATTGCAGCATTATGGGCATCTGGCAGCGTGGCCTTCTACAGGTTCCTAGACCACCTGATCAAGAGCCTTAACGGGGGTATAC
TGTTCGACCCTCAGCCGGACGGGTCCCTCTTCGGCCCACCCAGAATCCACTTGATGAGTGTGTCGTCAGAAGGGCTCGCCATTCTTGGGACAATTG
CATGTGCTGCATGTGTAGGTTTTCTCATAGACAACTGGCATGAATATGTGACTAGACAGAGGTTTCGCGAGAGAGTAAGAAGCCGATTGTCTCGGG
TGGACTACTACACCCCCAATGAGTGAGTAATAGCTCACCCCAAAATCCTCAGTGCTCAAATTGTGACCCACTGGGTATGTGTGATACACGAGAGTG
TCGCCTGTGGGTGCGCTTTATTTTCTAGCTGTGATATTCTTATGTTTTTATTAACAAAACCCGCTAGTTACAAATCAATAAGAGGAGTAACATAGG
AATGATGGAACGTGTAGATTATAAAGGCCTTGATTATGTAGGAATGCTCAGTGCTGGCCTCATACCACCACCGATTGGAGTCTTGAGAGGCAAGAA
AAAGACGTTTAAGAAGATGGCAGCCCTCTGGTCTCTAGCCTGTTGCATAAGATCCACTGAGGGCCTGGAGCAGCGTGCCTATATTGTCCGCGGAGT

AACCTTAACCTGTGGGGCTGAGTCAGAGGATTTTCAACTGGAAACTATGGTAGCGGTGGTATCAAAGGATGTTGGTCTTAGTGAGGATGACACATA
CCTTTTATCTGACTTGGTTGCAAGCGGGGAGAGGAAGGACGCTATATCAGCCTGCTATAGGTGGACAGCAGATAGTCGGAAGTTTGATGTGAATCT
GCCGGACATCCCGATCACTGGATCTGTTATGGCACAGAGATGGCTCAGAGAGGTATCTGAGCACATGGCAAGTGTCCTCTATCCTGACGATGCTAC
AACCGACCCACTATCTGGTGCTCATTTACCCCCTGATAAGTGGGATGAGGATCTGTCCACCCGGAGGTCTGTCTCAGGTGACGACGAAAGAAGTAT
GTCGTCTGATAACCTGGAGGAGTGTGCATCTGCTGGGAGTATCGCATCACCGGCAAAGGCGACCTCCAAGGCCAACACAAGTCTGCTTGGCCAAGA
CAGCTCCGAGGATTGTCCAATACTCCGTGAAGGACCTGAGATCCGGAGGCCTACTGACCCGGGTGAAAGAGAGGAATTCCAGAGGCAGGTTATTGG
GTGTCTAGGGGCTTACAGTGCGTCTTTAGTCCGTCAAGCCATTCAAGAGCAAGCAGACGAAAACCAGCGCATTGTTCAGATGGATACAGATATCCT
AATGAGAATGATAAAGGAGGAAGTGGATATGATAAACGATTGCACTCGAGCTAGGCAGTATCTATGCCAAGTTTATGACGCCCTGCACGATCAGCG
AATCCCGTATATGAAGAATATTGCCTCCTACCGTGAAGACATGGCTATGAGATGTCATGTGCTTGAGAATGCTCTCTGGGAGATCGTCACAGTCAG
AGGGAATGGATCGGGGGTTCCCCATATCTGGAATAAGGTGCACTTGGGTGATATGTATGCCAGATACTACCCCGCACATGCAGAGCTTATGAGGAG
TGCAAACTACGTTGCGGACATCGATCAAGTTTGGAGGAATGTCAACAATATGGTCATAAGTGAAATCAAAAATACAGGAAACTATGACCACAAGGA
TGTCCGAGACAATATACCACTGACAAAGGATGAGGTATTAACTGCCATTAAGAAGGCAGCAGAAGTCCCTGACTCCCTCAAGTCAATCCTCACCGC
AGGAGTAGACAAGATCCAGCTCATAGCAGACAAGTTTGTCTCAGGGTCCCGTCCCCTAGAAAAGGAGACAGTCGAGAGCTTGCGGGTGGACCGTCG
ATCCAGCGTCTGTGCCCCATTAGACTCCGACCCTGTAACCAAAAGTGAAGCTCCAGCTGCAGGACGATCGAAGAGAACTCCGGAAGAAGAGGCTGA
GCTTATCAGGGCATTCAATGCGAAGATGGATCGGATGTGAGGCATCAGTTTGCTAGTTCCTCCCTTTAATAAAACCCGCCAGTTAGAAATCATAAG
ATTTCACACTTGTTGTTTATGCAATTATTCAGTTCTTCCAAAAAGAGCTCCAGAACATTCGTCATGCTCAGCTTGATTACCCTCGCTGTGCTTGTG
GCCATTCGTGCTACGGTAGTCACCTGCTTAACAGCATTCGATTGCAATTCCAAATCAATAAATCTAACATCAATCTCTTTGGTTACAACCCCAAGT
TGTGATGTCTCTCATAAGAACATCACATACGAACAGATTAACATTGCAGTCACTCAATCAACTGAGCGTTATGAGCTCCCATTTTTCAGATGCCAT
GTTGAATCTAGAAGTGTGATGGGCAGGTGCGGACGTACCATTGATACCTTCCATAACGCAGGTCTCTTCTCGGAGATTATCCGCGTTGATAGGCAT
GAGTGTGACGTCATGCACAAGAAAAAAGTCTTCAGGATCGTGAAGGAAGGTTCCTTCAGCGATATACAGCTAAGTCCGGATGGGCAAACTCGCTAT
GCATACGTTAGTAGAGGCTATGTATCCCCGGATGGATCTTGTACCCCAGGTGCGGACCTTGTAAAGAATGGAAGAGAATATGACAGGCCCCTAATA
AATACAGAGCTCACCATCACGATATCAACGGGAACGTCTATCGTCTCTGTCGAAGAGAGTCAGATCAGGTTCCCGAATGGAAAGAGTTGTAAGCTC
CAGGAAGAGTCTTGCTTCGACGCAGACTACGGGGATGTGTTCTGGTCGGTCCCAACCCCCTCCTGTGATGAATCTGAGTCCGAGAAGAGTCTCGTC
TATGAGGGTCCGGGAATGCTAGTACGCGATGTAAATGGAAACTCGACTGTCGAGTACATCCATGTCCACTATTCTGGCTACGACTTTCAAGTTTTA
CTCCAATCTCGACAAGAGCTCATATGCGGGTACCGATCATTCTACACGGAACACCCTAGGTTGTTCGTGACACTTCTCTCTGTGAATGGACCAAAG
TTTCCTCTAAAGAGGCGGGTAACTGCGGTGGATGTCAGTTTGCTCAATTTTGTTAATTCTAAACTTGTGTATTCATTCAGGCATATCAAGCAAGAG
GTTAGTAGACTGTTCGACCTATTTAACCAGGATAGGTGTCAATCGCATAACAGAGTCACCCAAAACCTCCTGTCTATTGCCCTTCTATCCCCCCAG
GAATTCGCCTATACATATGGAGGTCCGGGACATACTGCCGTTTCTCGAGGCGAGGTTATTTACCTCGCTCAATGTCGTCCTGTGGCTGTAGTTCCC
GACCTCAATGTTACCGGATGTTATAACGAATTGCCAGTTGTTTATAATAACGAAACAATGTTTATGACACCTAGGAGTCGCATCCTTATAAACGTT
GGAACCCCGCTAAAGTGCCTGCCCGACCTATTGCCAAAATACTATTTGAATGAAAATTGGTATGTTAAAACAGACCACGGTCTGATAGAAACAAAA
CCCCCTCAGACCATCGCACCTAACACCCTAGCTTATGAGTTTAGAGAACTGTCAGGAATATCTGGAGGAGGGCTTTATAGGGCGGACATCATCCAA
AAGTATCAGGCAGCACTGATATCCCCAATGGTCGAAGCTGTCATCTCGACTCGAGTTGTAGATTCTCTAAGAGGAGAAGTGCAACTCCCCTCCGGG
TACCACTACACATCTGCCTTCTCCCCCTTAGATTATGAGAGCATCAAATCGAAGGTGGGGGGATTCTGGGACCGCTTCACAGAATCCGCCGTCCGT
GCAGGGGGGTGGTTTGGTTTTGTCCTTATTGTGTTTGGAATATATAAGTTTTGCTTATATGTGGTAACAACCATAATCAACTTCATGCATGTCAGG
CACGATGTAGGGACACTCTGGGCAATCCCTATTTGTCTGTTCGATGTATTATGTAATTTGGTGTTCCATGGGAGGATCTGGAAGGGCGAGCCTCCA
GCTGAGAGCGTTGAGCTCGAAGAAATTCTTCCAAATGAGTGATCGTCTGTAGGAGCTGCATCTTAGCAAGACAATGGTCATAATAATATTACAGTA
CTGATTTAATTCTTCGGGGTGAGTCAGAATAATCACCTATCTACCTAAGCGTATATAGTGCGATCTATGTGTAAGTTAACCTTTAGAAGTTTTATG
ATAATAGGCATCGGAAGTAAGTGTGGGACCATTATAAAAACCCGCCAGTTACAAATCATGCCTGTTTCAATAGCTTGTGCACATTGCCACTATCCC
ATTGACATCCAGTCAGTGAGTGCTCCCAGTGATGTTGGGATAAACTGCAAGATGTGTGGAAAAACATCATACGTAATCAATGCCCCATCTTAGTAT
GGGCGGCTAGATCCTCAACACCAAGTTCATATTTCTATTTTGCTCAAGCACAATGCAACCTAGCAAATGCACTTATATATCGATATATTTAGACAA
TTATACATATTGTGCAGCTCCATTTTCATCGCTGTGAGGCTCCTTTTCTTTATTTATATCTGGCTAATTTATTCAGTGTACTGGTTCTTTTGCTTA
TAAAAAACCCGCCAGTTACAGATCACTTATTGTGTATTTGGAGCATTAAAAATCGAAAATATGGCGAGCGAAGTGTGGTGTTGCGAAGATGAGGAT
TGGGAGATTAACTGTGAGGAGATGAGATATTTGGCAAACGATGTCACTTATCAGGAGGAAGCGACTGGTCAGAGGCGTATGACGGCTTGTATTCCT
GGCCACCTGGCATCCCCCATTTACGGAGAAGAAGTCGATATGCTGATGTCTGGCAAGAAGATTAGAAGGAGCTGTTATCGTCATAGGAAAGTGCGA
GCGCACATCGAACAGTGTACTTCATTCCGAGAACTTACTGACCCCATGGGAAGTCTGGAATCGTGGATTATGGGGAGGCCCATATCTAAGTCAACT
GTTGATATGAGGATCAAATGTATGATGCAAGGCGCTGTCACCTGGAAGATTCAAAAGACATCTTTCCTTAAACCTTTGGAACATCTGGTCTCTTCA
AAGGAAACACTGGATCACTGTGACTCAGTTATAAGTAAAATCAGGTCATCGATTAATAGTCTTAGAGCTGACTTAGAAAACATGATCAGGGTGGAG
GCGATAAAGGATGCAATATGCTTCAAGGAGATAGAAATGGGTTCTTTCATCCAGCATATGGGCGGAAAATTTGAAAGGGAATTGTCCATAATCCCG
ATGAGAGGAAAGCTATACTTGTTGCCCACAAACCTGCTCCTTTGTGCCTTGGATAAGATGCAAGCAATGTTTCCGTTGAAGCTGAACTGGCTCATA
TCAGATATCCTAGAACGATATCCAGGGTTAAGTATCCTCAAAACCGGATCTGAAATACTCGGAAAGTTCAAGAAGCTCCGGAGTATCAGACAGGAG
GGGTACCACTCCTTCCTGGCAGCATGGGAACCGCTGGTGGTGGGGGATACAATACGGCAGCCAGGAGATCTAGGAAATCTGGCCCTGTACGTCCAC
CAGCGTTCCAGTATGGAGGACCACCTTAGGGTGACCAAGTCGCCACTTAGGATAGAGGACTTGTTGCCCCGAGCTGAGGGAGGAGAGAATGAGAGG
AGAATGTTCCTAGAGTTAACTGGAATAGCTAAGATGACGGGGTACCCCATACTGAAAGCAGATAAGCTTCTTGATCAGCTCAGGGAGCATGGAACT
GGATACCCTGAGACTATTGAGCCAACAGTCGTCGATACAGTTGTTGCTATATGTCGGAGAGACTGGATGCTGAGTTACTTTAGAAAGAAGGGTTGC
TATCCGAAGTTGGTATCTTATCCGGTCGAATTCAAGGGTTTCATAGACAAGAACAAGGAACCCCAAGAGAAGTATATGACGATGTACGACTTATGG
GGCTCTGTAACATTCGGGAAGACGCTTAACTTCAATTACAGCCCTGATCTAGCGGAGATAACTAAGGACTCGGCTTGCGCAGTTGAGTTCCAGGAA
TGGCCGAGCACCTTTGACAGGTGTGCGTTCCAGTATTTGTACGATAAAGACCCACCCCGAACTCCGAGGGTTAAGTCACATCGCCGAGTAATAGAT
GCCTTTATAACGGCTGAGCCTGACCTGCTCAAAAAGGAGATGAACAAGAGAGAGAAGGGGGCAATGGATATGAAGGACAAGATAATCATCCAGTGT
GGGAAGGAGCTGGAACTGAAGGAGGACACAGGGAGGGCGTTTACGAAACAGACTGCCACTCAAAGATACTTTCAAGTCTCGTTAGAATCTAACACT
GCAGACGGGATCTTCCCATATGTAGACGAACAGTCAATGACAGAGAGTGAGACGGCTATTGCAAACAAACACATGGCTCAGGTCAAGGCCATGGGA
GGGACATCTGAGTTCGTCAGTTTAGATCTTACCAAGTGGTGCTTGAGGCATAGGCAAGTCATGATCCGTCCAATCGGAAAAATGTACGACGAGATC
TTTGGTCTCAACGGCATATATGAAAATAGTCACCTCTTCTTTTGTGGTGTTCCCACCTTTTGCAACAACAGATTCTCTCCTCCTGACTATGATAGT
AAAGGCCCCATACCTGGGCCGTTCTACTTGGAGGACTTCTGCGGAGGGTGTGAAGGAATGCATCAGAAGAAGTGGACTCACTTCACCATAGGCGTC
ATAAAGCTTGCGTTAGAGAGGTGTAAAATGAAAGGCACAATCATGGGCCAAGGGGACAACCAGGTCATCCTCCTGCATTACAGTATGGAGGATCTT
CCCAATAAAGATGTAATGAGGGCTGCTTTTCTTCGAACTTGCGAGACTTACTTTGAGGCAGTTGGGCACAGGTTAAAGCCTCAAGAGACGTGGTAT
TCAAGCAAGCTGCACGAATATGGGAAACAGCGGATTTACAACGGAATGTCTATTACCATGGCCACAAAGAAAGCAACCAAATGTATTCCTGATATA
AATGACGGGCTATTCTCAATACCATCGTCTATATCAACATTAAACACTACAACAGAATCTATCGCCAAAGGTAGCATAGACCCCGATGCAGCCTTC
CTAATGAACCAAATGCTGGTGGCAAATTACCTGTGCAGAAGGAAATTAAACTTTGATTGCTCTATGAACCCCGAAGATGCCGTTGCAACATCGCTC
CTGTTCCCTGCCGATTTTGGAGGTCTACCACTGTCAACTTACTCCTCTCACGCTGTAAGGGGCCACGACGACCCGGTAACTACGTGGCTAGGGATA
GCAATGACACTCAAGGAGCATTTCCCAGCCCGATACCGGACTGTCATGAACGTGTGGAGGATGAGGCCCAACTCCCCTGCATCATCTGCTTTGGAG
AGAACAAGGCTCTATGAGGATCCCTTTAGCCTAAACGTAAGGAGCCTCCCGTCAGCCGAATCTGAGATACGTCAGCACACTCTCTCGTTCCTCAAG
TCCGCAGCAGTTACCAACCCCTGTATTAAGAAGCTGTACTCCACAGATGTATCGGTCAGCTACTCAGAGCTCATACGCATACTTGATACGATGGAG
CCATGCTACCCCCAGTTTGCAAATGCCATTCTGAAAGATAGCAATGCCGGGATAGGGAAGATGCTCCAGGCAAAGCTCACCAACTCAAAAACAATT
GAGAAAGCAACTGTATCTTACACCCAGGTATCTCTCATTGATTTAATAAAAGAGAAAAATGCAGAGCTGTCGCGAGAATTGTCTAGGAGGTTAAGC
TCCAACAACTTCTCAATGAACGCCCGATATTTCTCTGTGATCTGCCCCTATGAACTGGCAGAGAAACTCAGGAAAGATAGCTGGGAAAGAATTTT
ATAGGACTGTCCAAAGCCCCCTACTACCACCAAGTTGTGCTCAAAGACATCGACGCTGCCACCGAGGATGAACGGGATAGATCAATCGTAGTTAAG
CTGTCTGACCAGGCTCGTGAAGGGCACCCTTACTTCTCTACCGGGTTCGGACCCTACAAACCATATGTCGGGAGCAGGACAAAGGAGAAGGTGACC

AAACCGACCATCGACCTAACAGAGAAGACAAGCTACACGAGGGCCTTCAAGCGCTTAGGAAAGCTCAAAACCTGGCTACAGATCATCTCAGCCCCT
AACCTGACATCACTCATAGATAGATTGCTGTGTGAAAAGCTTGAGTTGATAGTTCTCCCTGACTGTGCTGATGACGTCAGTGGTGTATTTGCTAAG
ATTAGATCAGGGAATATCTTTCATCGACTCTCGACCGATGTAGAAAGGTCTAGCGCTCAGATCAACTCCCTTGTTACCATCCCAAGTCATTTCAGC
CAGTCCAGCAACCTGCTCCAAGGCATGTCTCAAGATGGGGAAGATTACTCTATATTCTTCCAAAAGATATACGCTGAGAATATGGCAGCTCTGTCT
CTAGGATCGAGAATAAAACCAGGTAGTCTGCCTCCGGTCTGCGTCGCGGTACTCCAGTGCAGAAGCTGCACTCATATCCTTCCAGACCCTGACTTT
GACATCAAGCCTCAGAATGTCACTGTTGAAACTCCCATAATGCTCGACTCCAGATCTACTCCTGTTCTTCCTGCTTCTAATCTTGATACGTCCTTG
CTGTTCAATATTAGCATTGGCCTTGACTTAGCCCAGAATGTAGACGACAACTTCAAAGCGAATCATCAGGGTCACACATCGTTCTCATCAACAGTA
GTAGTTAAGAAGGGCAGAGTCTCAGTAAATGACTTCAAGAGATGTGACCTTCTGGTAATACTGTTGTCTATGATCTATTATAGTACGCATTGTCAT
AGAATCTTGTTCGGTCGGGATGAGCTTCTGAGGAAGTCGTCCAACGATCTTTCATTTTCGTATGTAGCAGATCTCATCACGGAGTCAGATCGCAGG
GAGGCCTTATTTGAGCTACTAGGCAGAGAGGTATCCGAGCATACAATGGTAACAAAGTCGGAGAGGATGTCTGCATACATAGCACGAACAATTCTT
CCATTTTACAAGACAAACATTGAAATAGTAGTGCAGAAGATACTCCCGATCCAGTTCTTGGAGGACATGCGTTCTTGGAGATTGAAACATGCAGTC
CTGGCCTTGATTGGTTTTTGTAGGCAGAATAGACTCTACCCTAAAGTGGATGAAGGGACAAGACAGAACGCCTTAATGTGGAACATGTATCAGGTC
AAAAAGGCCTTCGGTGTTAAACACACTGTAGTGCGAATAGCCAGAGAGTCCATATTGACAGAGTGGCGGGCTGCCCCTACAATAACGTATCGACCA
CCAACAGCAGTTCGGAGAGAGATGTTTTTACCAGACACTGCTTACCCCTTGGCTGAGGCCGATGCTTACCATCGGCTACGATTTTTGAATCAGCAG
GGTCCACTATGCTACCCCTTCTTATCCTTTCTGAGCCGGTCCATAGTATTTATTTCGTCCGCAGCATCAAAGTACGTGGAATGCCTTGCTGCGGTC
AGGTTCTTCGACAGCCACAAGTATAAGGATGGAGTTGTTCTGTCACTCGCGGAGGGATCTGGGGGGGTTCAGAATACACTCCTCACTCTGCTCCCG
AATAGTGAGGGGATGTACAACACGTGGATGAATCCAAGCATTGATAACAGAGACTGTGCAACTGACGTCAAGGCGCCTGCATGCGTGGTATCAGGG
CTCACTGGAGTTGCTAGATCGCACGAACTCGCATTCGGTGAGACCAATATTCTATCTCACTCCTTTCTAGAGAAACTGGGACGGTGCTGTAAGGAT
AAGAACATCCTTGTTGTCACGATGGATGCTGAGAGTATGATGCACAGCACGAACATCGAGTTCGTAGATAAGTTGTTTCCAGTCGTACTGGGTTTC
CATCCGATAATAGTCATCTTCAAGATGTTCAACTATGGCTCTCTAAGTGATCAGATTAAAGGAGTAAAAGGCATGTATCCTGATTATGATTTTGTA
TTTTTTAAACCGATAAGCAGTAACCCTGTGGGAAGAGAAATATTCCTTATTGGGGCGAGGTTAGGAGAGGGAATGGCCCTTGACCCGCTAAAGAGA
AATGAATATACACAATTGTCTTACACCGGCAATCTAAGTGGTATGGATAGCTTCTCATTCGAATCGTATGTCGGTGTTTCCTTGCGTATGAGAAGC
TACTTCTCGAGCTTCTTTGTCATGTCTTCAGCCTCCTACGGGGATAGACCTCTCGTGGAGGGGGCATGTGGGCTAATCTGTAGGGTACAACTAGCA
GCACTCAGCGCATCCATTGACCGGGTTCATAATAATGAGGATGATCCGGATGTCCACACAGTAATTCGGGCATGCGGGACCAACGAATGGCTCTCA
AACAAACTCCCGGATTATGCTTTTCTGCAGATCATGTTCTCATACCCATCAATGACACTCAAACAGATGCTAGCCACCTTGGAGAAATGCAGCCTA
ACGGGAAGGATGAGGGAGTTCAGGGCTGCCATGGGGAATACAGAGGTTCAGATTCCTCTATTGAAAATCAACATTAGTGGGGGGGACTTCTTGGAG
AAATGGAAGGACGTTAAATTCTACATGAGGAATTGGGGGGGTACCTGCCGATGCACATTCGACGTTAGGGTAATCCCACATGGAGTCCGAAGCCTC
TCCTTTCACATAGACAAATCACTGGAAGAATCAGGAGTACTTCCAATAGGGCAAAGGGTAAGATTGACACAGCGATCAAAGGAGCGCTTCAACCCA
CCAGTTCTTTCGCTAGAATAGGTGTACAAGTTCTCCCAAATAGAGAAAGCTTACTTTTAAAGGGCCCGCCTAATCAAGTTCTTTGTTTGTGTTTAG
ATACATCCTGATCCCATCCCAGGACCTGGGAGAGAACCCAGATGCCTCAGCACATGTGTGCACAATGGCCATGTTGGCCTGAATGTATTGCTATGT
TTGGCACTCCATGTCTGTTGGCTTAAGGGTATCAGGAGTATTACTCACGCTCTGCCCCCCTGTGAGAGGCATGCTTGACAAATAATACTTTTGTGC
ATCCAACAATAAGTAGAGGGTGCTCCCTTTGCGCCCCGGATAAAAAACGGAAAAATAGAGTCTACTGCCTTTGGCATACCCCCCTACAGCAGAGAA
ACTCTGAACAAGTTGATCCATGCACTTGGAAAGGCTTGAGTGTATAATTCCCGCACGAATAAATGGGCAGCTAGGCAGTGTATAGAGTTGGTTGTT
TATTGCGG

>c36378_g1_i2_len=10372
TGCACCGCAAAAACATAAAAACCATAGGTTCTGAATTAGTAGTGGCGGGCTTAGTATCACTTATATACTTGAACCTAGTACGGTGTCAACTTGAAT
AAACCCGCTAGTTAGAAATCATACGATATTTCGCAGATTTCGAACATCTTAAACTGTGACACAAGATGGCAAAGGTTGTAGACGAGCGATATCTCC
AAGAGATAAGCCTAATTGCACCGATCAGGGAAGACAACTTTACAAATTACGAGTTCACGGAGGTTATCTACCGAAAGGGAGCGGCATCATACTTGC
TGAACATTGGGACTCATAAGGAGACAGAAGAAAGGGCTGTGCCAGGATACGATCACCAATCCCTTTGCGCGGCACTCGGGAAAATCTGCCCATTCA
AGGGAGAGCGATCTATCCCTAAGGGTGTATCTGATGCATCAGTTACTATCCTGAACCCGCAGAGAATTAGAGAACTATCGCAAGTTCTTGCTGCAT
TGCCTAAGATAAAGCTCTTTGATGAAAATGGAGGGATCAACATCTCTATATTGCTGGCACTCCTGCGTTGGATTCCCGGCATGGATGGTGTCCACC
TCTCATATCTGGCTGCGCGAGATGAGGGGGTAGCCATGGTCGAAAGCGATCTGGTCTTCAGGGTCATGGTTGCTGTGCATCCCGAATTTGATGGAA
CGAGCCCCACCTCTTCGTACGCGAGGCATACAACTGCCACAATGATCTGCGCCGCATATGCTGTGCTTTTGATGGTTATCAAGACCATTAACGCCA
ATGCAGTGAATCAGCTCGACATCTTTATTGATCGCAGAATACGGGCTCTCTGTCATACTCTCGGTCTCTCAGGTAAGTCCATCGACTGGGAGAACA
AGATCAAGCCCTTGCTTGACGAAGAAGAACTGCGCAAATTAAACGAGGAGTTAGGGTTCTTCCCACGCTTGAAGAAGGTCATCTTTGTTCCTGTCA
TAGATATGCTGACTCCTGAACTGCAGCATATGAATTTAATATTTCAGGAAACATCGATGACCATATTCTCGCTAATAGCCGAGTTTTGGATGACGT
CTGACATAACAAGACTACATGTCTCTCCGCTCGTGCTTGTGGAGTTGCCGAAATGGCAGAAAACCATGCATCAACTTGTGGCCTTGTACGGTACAT
CGTGGAGGTTTTACAAATTGATTGACCCAAGAGGGACACTCACTGCCCAGAGCAACTTCCGTACGTTGGGGTGCGCTGCTCTATCATGGAAGAGAC
TCAACACTATCCAGTCCGGCCAAACGTCCTTGGCTCAATTACAGGGGGTCAAAATCAACCCTGAATTCGAGAAACTGGCATCCATAAAGTTGAAGC
CGGAGTTTGTGGGCACCAATGCAAAATCGTACTTGGAAATTGTCAAGGGTCTCCTGCAGGACCAGTCCCCGTACATCAATATGAATTGGTCCAGGA
TCGTGAAGGCTATTTCAGATGGGGAGTGGGTAGAAGGCAGAGACTTCTAAGATTGTCCACAGCCTAGGGTCCACTTCCATATCAGGAAATGCACAG
AAGAGGCCATATACATTAGCCACCTGAGGTATATATCCATACCTCCACACCGACCTATCGGCGAAATTTCATAATAATAAAAAACCCGCTAGTTAC
AAATCATGTCAATCATTGCAGCATTATGGGCATCTGGCAGCGTGGCCTTCTACAGGTTCCTAGACCACCTGATCAAGAGCCTTAACGGGGGTATAC
TGTTCGACCCTCAGCCGGACGGGTCCCTCTTCGGCCCACCCAGAATCCACTTGATGAGTGTGTCGTCAGAAGGGCTCGCCATTCTTGGGACAATTG
CATGTGCTGCATGTGTAGGTTTTCTCATAGACAACTGGCATGAATATGTGACTAGACAGAGGTTTCGCGAGAGAGTAAGAAGCCGATTGTCTCGGG
TGGACTACTACACCCCCAATGAGTGAGTAATAGCTCACCCCAAAATCCTCAGTGCTCAAATTGTGACCCACTGGGTATGTGTGATACACGAGAGTG
TCGCCTGTGGGTGCGCTTTATTTTCTAGCTGTGATATTCTTATGTTTTTATTAACAAAACCCGCTAGTTACAAATCAATAAGAGGAGTAACATAGG
AATGATGGAACGTGTAGATTATAAAGGCCTTGATTATGTAGGAATGCTCAGTGCTGGCCTCATACCACCACCGATTGGAGTCTTGAGAGGCAAGAA
AAAGACGTTTAAGAAGATGGCAGCCCTCTGGTCTCTAGCCTGTTGCATAAGATCCACTGAGGGCCTGGAGCAGCGTGCCTATATTGTCCGCGGAGT
AACCTTAACCTGTGGGGCTGAGTCAGAGGATTTTCAACTGGAAACTATGGTAGCGGTGGTATCAAAGGATGTTGGTCTTAGTGAGGATGACACATA
CCTTTTATCTGACTTGGTTGCAAGCGGGGAGAGGAAGGACGCTATATCAGCCTGCTATAGGTGGACAGCAGATAGTCGGAAGTTTGATGTGAATCT
GCCGGACATCCCGATCACTGGATCTGTTATGGCACAGAGATGGCTCAGAGAGGTATCTGAGCACATGGCAAGTGTCCTCTATCCTGACGATGCTAC
AACCGACCCACTATCTGGTGCTCATTTACCCCCTGATAAGTGGGATGAGGATCTGTCCACCCGGAGGTCTGTCTCAGGTGACGACGAAAGAAGTAT
GTCGTCTGATAACCTGGAGGAGTGTGCATCTGCTGGGAGTATCGCATCACCGGCAAAGGCGACCTCCAAGGCCAACACAAGTCTGCTTGGCCAAGA
CAGCTCCGAGGATTGTCCAATACTCCGTGAAGGACCTGAGATCCGGAGGCCTACTGACCCGGGTGAAAGAGAGGAATTCCAGAGGCAGGTTATTGG
GTGTCTAGGGGCTTACAGTGCGTCTTTAGTCCGTCAAGCCATTCAAGAGCAAGCAGACGAAAACCAGCGCATTGTTCAGATGGATACAGATATCCT
AATGAGAATGATAAAGGAGGAAGTGGATATGATAAACGATTGCACTCGAGCTAGGCAGTATCTATGCCAAGTTTATGACGCCCTGCACGATCAGCG
AATCCCGTATATGAAGAATATTGCCTCCTACCGTGAAGACATGGCTATGAGATGTCATGTGCTTGAGAATGCTCTCTGGGAGATCGTCACAGTCAG
AGGGAATGGATCGGGGGTTCCCCATATCTGGAATAAGGTGCACTTGGGTGATATGTATGCCAGATACTACCCCGCACATGCAGAGCTTATGAGGAG
TGCAAACTACGTTGCGGACATCGATCAAGTTTGGAGGAATGTCAACAATATGGTCATAAGTGAAATCAAAAATACAGGAAACTATGACCACAAGGA
TGTCCGAGACAATATACCACTGACAAAGGATGAGGTATTAACTGCCATTAAGAAGGCAGCAGAAGTCCCTGACTCCCTCAAGTCAATCCTCACCGC
AGGAGTAGACAAGATCCAGCTCATAGCAGACAAGTTTGTCTCAGGGTCCCGTCCCCTAGAAAAGGAGACAGTCGAGAGCTTGCGGGTGGACCGTCG
ATCCAGCGTCTGTGCCCCATTAGACTCCGACCCTGTAACCAAAAGTGAAGCTCCAGCTGCAGGACGATCGAAGAGAACTCCGGAAGAAGAGGCTGA
GCTTATCAGGGCATTCAATGCGAAGATGGATCGGATGTGAGGCATCAGTTTGCTAGTTCCTCCCTTTAATAAAACCCGCCAGTTAGAAATCATAAG
ATTTCACACTTGTTGTTTATGCAATTATTCAGTTCTTCCAAAAAGAGCTCCAGAACATTCGTCATGCTCAGCTTGATTACCCTCGCTGTGCTTGTG
GCCATTCGTGCTACGGTAGTCACCTGCTTAACAGCATTCGATTGCAATTCCAAATCAATAAATCTAACATCAATCTCTTTGGTTACAACCCCAAGT

TGTGATGTCTCTCATAAGAACATCACATACGAACAGATTAACATTGCAGTCACTCAATCAACTGAGCGTTATGAGCTCCCATTTTTCAGATGCCAT
GTTGAATCTAGAAGTGTGATGGGCAGGTGCGGACGTACCATTGATACCTTCCATAACGCAGGTCTCTTCTCGGAGATTATCCGCGTTGATAGGCAT
GAGTGTGACGTCATGCACAAGAAAAAAGTCTTCAGGATCGTGAAGGAAGGTTCCTTCAGCGATATACAGCTAAGTCCGGATGGGCAAACTCGCTAT
GCATACGTTAGTAGAGGCTATGTATCCCCGGATGGATCTTGTACCCCAGGTGCGGACCTTGTAAAGAATGGAAGAGAATATGACAGGCCCCTAATA
AATACAGAGCTCACCATCACGATATCAACGGGAACGTCTATCGTCTCTGTCGAAGAGAGTCAGATCAGGTTCCCGAATGGAAAGAGTTGTAAGCTC
CAGGAAGAGTCTTGCTTCGACGCAGACTACGGGGATGTGTTCTGGTCGGTCCCAACCCCCTCCTGTGATGAATCTGAGTCCGAGAAGAGTCTCGTC
TATGAGGGTCCGGGAATGCTAGTACGCGATGTAAATGGAAACTCGACTGTCGAGTACATCCATGTCCACTATTCTGGCTACGACTTTCAAGTTTTA
CTCCAATCTCGACAAGAGCTCATATGCGGGTACCGATCATTCTACACGGAACACCCTAGGTTGTTCGTGACACTTCTCTCTGTGAATGGACCAAAG
TTTCCTCTAAAGAGGCGGGTAACTGCGGTGGATGTCAGTTTGCTCAATTTTGTTAATTCTAAACTTGTGTATTCATTCAGGCATATCAAGCAAGAG
GTTAGTAGACTGTTCGACCTATTTAACCAGGATAGGTGTCAATCGCATAACAGAGTCACCCAAAACCTCCTGTCTATTGCCCTTCTATCCCCCCAG
GAATTCGCCTATACATATGGAGGTCCGGGACATACTGCCGTTTCTCGAGGCGAGGTTATTTACCTCGCTCAATGTCGTCCTGTGGCTGTAGTTCCC
GACCTCAATGTTACCGGATGTTATAACGAATTGCCAGTTGTTTATAATAACGAAACAATGTTTATGACACCTAGGAGTCGCATCCTTATAAACGTT
GGAACCCCGCTAAAGTGCCTGCCCGACCTATTGCCAAAATACTATTTGAATGAAAATTGGTATGTTAAAACAGACCACGGTCTGATAGAAACAAAA
CCCCCTCAGACCATCGCACCTAACACCCTAGCTTATGAGTTTAGAGAACTGTCAGGAATATCTGGAGGAGGGCTTTATAGGGCGGACATCATCCAA
AAGTATCAGGCAGCACTGATATCCCCAATGGTCGAAGCTGTCATCTCGACTCGAGTTGTAGATTCTCTAAGAGGAGAAGTGCAACTCCCCTCCGGG
TACCACTACACATCTGCCTTCTCCCCCTTAGATTATGAGAGCATCAAATCGAAGGTGGGGGGATTCTGGGACCGCTTCACAGAATCCGCCGTCCGT
GCAGGGGGGTGGTTTGGTTTTGTCCTTATTGTGTTTGGAATATATAAGTTTTGCTTATATGTGGTAACAACCATAATCAACTTCATGCATGTCAGG
CACGATGTAGGGACACTCTGGGCAATCCCTATTTGTCTGTTCGATGTATTATGTAATTTGGTGTTCCATGGGAGGATCTGGAAGGGCGAGCCTCCA
GCTGAGAGCGTTGAGCTCGAAGAAATTCTTCCAAATGAGTGATCGTCTGTAGGAGCTGCATCTTAGCAAGACAATGGTCATAATAATATTACAGTA
CTGATTTAATTCTTCGGGGTGAGTCAGAATAATCACCTATCTACCTAAGCGTATATAGTGCGATCTATGTGTAAGTTAACCTTTAGAAGTTTTATG
ATAATAGGCATCGGAAGTAAGTGTGGGACCATTATAAAAACCCGCCAGTTACAAATCATGCCTGTTTCAATAGCTTGTGCACATTGCCACTATCCC
ATTGACATCCAGTCAGTGAGTGCTCCCAGTGATGTTGGGATAAACTGCAAGATGTGTGGAAAAACATCATACGTAATCAATGCCCCATCTTAGTAT
GGGCGGCTAGATCCTCAACACCAAGTTCATATTTCTATTTTGCTCAAGCACAATGCAACCTAGCAAATGCACTTATATATCGATATATTTAGAAAA
TTATACATATTGTGCAGCTCCATTTTCATCGCTGTGAGGCTCCTTTTCTTTATTTATATCTGGCTAATTTATTCAGTGTACTGGTTCTTTTGCTTA
TAAAAAACCCGCCAGTTACAGATCACTTATTGTGTATTTGGAGCATTAAAAATCGAAAATATGGCGAGCGAAGTGTGGTGTTGCGAAGATGAGGAT
TGGGAGATTAACTGTGAGGAGATGAGATATTTGGCAAACGATGTCACTTATCAGGAGGAAGCGACTGGTCAGAGGCGTATGACGGCTTGTATTCCT
GGCCACCTGGCATCCCCCATTTACGGAGAAGAAGTCGATATGCTGATGTCTGGCAAGAAGATTAGAAGGAGCTGTTATCGTCATAGGAAAGTGCGA
GCGCACATCGAACAGTGTACTTCATTCCGAGAACTTACTGACCCCATGGGAAGTCTGGAATCGTGGATTATGGGGAGGCCCATATCTAAGTCAACT
GTTGATATGAGGATCAAATGTATGATGCAAGGCGCTGTCACCTGGAAGATTCAAAAGACATCTTTCCTTAAACCTTTGGAACATCTGGTCTCTTCA
AAGGAAACACTGGATCACTGTGACTCAGTTATAAGTAAAATCAGGTCATCGATTAATAGTCTTAGAGCTGACTTAGAAAACATGATCAGGGTGGAG
GCGATAAAGGATGCAATATGCTTCAAGGAGATAGAAATGGGTTCTTTCATCCAGCATATGGGCGGAAAATTTGAAAGGGAATTGTCCATAATCCCG
ATGAGAGGAAAGCTATACTTGTTGCCCACAAACCTGCTCCTTTGTGCCTTGGATAAGATGCAAGCAATGTTTCCGTTGAAGCTGAACTGGCTCATA
TCAGATATCCTAGAACGATATCCAGGGTTAAGTATCCTCAAAACCGGATCTGAAATACTCGGAAAGTTCAAGAAGCTCCGGAGTATCAGACAGGAG
GGGTACCACTCCTTCCTGGCAGCATGGGAACCGCTGGTGGTGGGGGATACAATACGGCAGCCAGGAGATCTAGGAAATCTGGCCCTGTACGTCCAC
CAGCGTTCCAGTATGGAGGACCACCTTAGGGTGACCAAGTCGCCACTTAGGATAGAGGACTTGTTGCCCCGAGCTGAGGGAGGAGAGAATGAGAGG
AGAATGTTCCTAGAGTTAACTGGAATAGCTAAGATGACGGGGTACCCCATACTGAAAGCAGATAAGCTTCTTGATCAGCTCAGGGAGCATGGAACT
GGATACCCTGAGACTATTGAGCCAACAGTCGTCGATACAGTTGTTGCTATATGTCGGAGAGACTGGATGCTGAGTTACTTTAGAAAGAAGGGTTGC
TATCCGAAGTTGGTATCTTATCCGGTCGAATTCAAGGGTTTCATAGACAAGAACAAGGAACCCCAAGAGAAGTATATGACGATGTACGACTTATGG
GGCTCTGTAACATTCGGGAAGACGCTTAACTTCAATTACAGCCCTGATCTAGCGGAGATAACTAAGGACTCGGCTTGCGCAGTTGAGTTCCAGGAA
TGGCCGAGCACCTTTGACAGGTGTGCGTTCCAGTATTTGTACGATAAAGACCCACCCCGAACTCCGAGGGTTAAGTCACATCGCCGAGTAATAGAT
GCCTTTATAACGGCTGAGCCTGACCTGCTCAAAAAGGAGATGAACAAGAGAGAGAAGGGGGCAATGGATATGAAGGACAAGATAATCATCCAGTGT
GGGAAGGAGCTGGAACTGAAGGAGGACACAGGGAGGGCGTTTACGAAACAGACTGCCACTCAAAGATACTTTCAAGTCTCGTTAGAATCTAACACT
GCAGACGGGATCTTCCCATATGTAGACGAACAGTCAATGACAGAGAGTGAGACGGCTATTGCAAACAAACACATGGCTCAGGTCAAGGCCATGGGA
GGGACATCTGAGTTCGTCAGTTTAGATCTTACCAAGTGGTGCTTGAGGCATAGGCAAGTCATGATCCGTCCAATCGGAAAAATGTACGACGAGATC
TTTGGTCTCAACGGCATATATGAAAATAGTCACCTCTTCTTTTGTGGTGTTCCCACCTTTTGCAACAACAGATTCTCTCCTCCTGACTATGATAGT
AAAGGCCCCATACCTGGGCCGTTCTACTTGGAGGACTTCTGCGGAGGGTGTGAAGGAATGCATCAGAAGAAGTGGACTCACTTCACCATAGGCGTC
ATAAAGCTTGCGTTAGAGAGGTGTAAAATGAAAGGCACAATCATGGGCCAAGGGGACAACCAGGTCATCCTCCTGCATTACAGTATGGAGGATCTT
CCCAATAAAGATGTAATGAGGGCTGCTTTTCTTCGAACTTGCGAGACTTACTTTGAGGCAGTTGGGCACAGGTTAAAGCCTCAAGAGACGTGGTAT
TCAAGCAAGCTGCACGAATATGGGAAACAGCGGATTTACAACGGAATGTCTATTACCATGGCCACAAAGAAAGCAACCAAATGTATTCCTGATATA
AATGACGGGCTATTCTCAATACCATCGTCTATATCAACATTAAACACTACAACAGAATCTATCGCCAAAGGTAGCATAGACCCCGATGCAGCCTTC
CTAATGAACCAAATGCTGGTGGCAAATTACCTGTGCAGAAGGAAATTAAACTTTGATTGCTCTATGAACCCCGAAGATGCCGTTGCAACATCGCTC
CTGTTCCCTGCCGATTTTGGAGGTCTACCACTGTCAACTTACTCCTCTCACGCTGTAAGGGGCCACGACGACCCGGTAACTACGTGGCTAGGGATA
GCAATGACACTCAAGGAGCATTTCCCAGCCCGATACCGGACTGTCATGAACGTGTGGAGGATGAGGCCCAACTCCCCTGCATCATCTGCTTTGGAG
AGAACAAGGCTCTATGAGGATCCCTTTAGCCTAAACGTAAGGAGCCTCCCGTCAGCCGAATCTGAGATACGTCAGCACACTCTCTCGTTCCTCAAG
TCCGCAGCAGTTACCAACCCCTGTATTAAGAAGCTGTACTCCACAGATGTATCGGTCAGCTACTCAGAGCTCATACGCATACTTGATACGATGGAG
CCATGCTACCCCCAGTTTGCAAATGCCATTCTGAAAGATAGCAATGCCGGGATAGGGAAGATGCTCCAGGCAAAGCTCACCAACTCAAAAACAATT
GAGAAAGCAACTGTATCTTACACCCAGGTATCTCTCATTGATTTAATAAAAGAGAAAAATGCAGAGCTGTCGCGAGAATTGTCTAGGAGGTTAAGC
TCCAACAACTTCTCAATGAACGCCCGATATTTCTCTGTGATCTGCCCCTATGAACTGGCAGAGAAACTCAGGAAAGATAGCTGGGAAAGAATTTT
ATAGGACTGTCCAAAGCCCCCTACTACCACCAAGTTGTGCTCAAAGACATCGACGCTGCCACCGAGGATGAACGGGATAGATCAATCGTAGTTAAG
CTGTCTGACCAGGCTCGTGAAGGGCACCCTTACTTCTCTACCGGGTTCGGACCCTACAAACCATATGTCGGGAGCAGGACAAAGGAGAAGGTGACC
AAACCGACCATCGACCTAACAGAGAAGACAAGCTACACGAGGGCCTTCAAGCGCTTAGGAAAGCTCAAAACCTGGCTACAGATCATCTCAGCCCCT
AACCTGACATCACTCATAGATAGATTGCTGTGTGAAAAGCTTGAGTTGATAGTTCTCCCTGACTGTGCTGATGACGTCAGTGGTGTATTTGCTAAG
ATTAGATCAGGGAATATCTTTCATCGACTCTCGACCGATGTAGAAAGGTCTAGCGCTCAGATCAACTCCCTTGTTACCATCCCAAGTCATTTCAGC
CAGTCCAGCAACCTGCTCCAAGGCATGTCTCAAGATGGGGAAGATTACTCTATATTCTTCCAAAAGATATACGCTGAGAATATGGCAGCTCTGTCT
CTAGGATCGAGAATACTTTCGCTAGAATAGGTGTACAAGTTCTCCCAAATAGAGAAAGCTTACTTTTAAAGGGCCCGCCTAATCAAGTTCTTTGTT
TGTGTTTAGATACATCCTGATCCCATCCCAGGACCTGGGAGAGAACCCAGATGCCTCAGCACATGTGTGCACAATGGCCATGTTGGCCTGAATGTA
TTGCTATGTTTGGCACTCCATGTCTGTTGGCTTAAGGGTATCAGGAGTATTACTCACGCTCTGCCCCCCTGTGAGAGGCATGCTTGACAAATAATA
CTTT

>c70967_g1_i1_len=511
GTTATTTTTGTGCATAATCTAATTGTAGTGTGTAACCAATGCGCACATAGTCATCATTTACGTCCAATTGCCGCTCCATTTAATGTATGTGCCTAT
TGGTTACCCGATAATAATGGATTAACTCCTTCCAAAATTGTATATAATCATAATTTAACTTGGAGTTTACACGGATGGTGGCCACAAGTTTTATTG
CCACAACAATCTCCGATACCCTATCAGCAATTTTATTCTCATTTTATTGCTTTGAATTTAACTAATCACTTTTTAGATATGACTCCTTCTCAACAT
TGTTGTTGTTCGGCCTATCATGAATATAAAAAACATGGAGCTCATAGTGGATATAATATGACCACATGGATTAATCAAATCAAATGGTGTTTAGCT
GTGAAGACTCAGTTATCTCATTGTTACGTACACCAATGCCGATATAATCATGTTTTAAAGTGCAATATTCCACAATCCTGTACGTTACCTAAACCA
CTTTCTCCTACTATTGGATGTCGTATGAATT

>c14012_g1_i1_len=320
GGCTAACCACATACCAGGATACAAAATGCAAACGGATTTTCCGTGGCATGTGTCTCCAATTCGCCTCATCCCCGGTCCGGTGCCCGGAACCCACGT
CAAGGTTGACCCAAAGTGTGAAGGACGTTCAGCTGCGTCTGTGGTAAAACCCGGTGAGCAATCCAATTGGTGTTGCAAAAATTGTTCCCACCCAGT
ACATTTTCAAGTGGGGCCGGCCTTGTATTACCCCATGGAGATACAAAAAGCAGTCACAATCACAGATAGCAATCCAAAAGTCTCAAAGCTGAAATT
GACGGAAACGCCTTTGTCTGATGATCCTGAAC

>c15103_g1_i1_len=433
GTGCATCGATCGTAACCGAGTCCAAAGGTCTTGGCCCATATGGTCTAGGCAAATTCGTAAATGTTTCATTGTTGATGATAGTAATGACTGGTGACA
ATGTGTGAATAAAAGCGCCTTCTGCACCAGCAGGAGGCCAGTTCCATCAGGTCTTCGGACCAATGCCAACTAAATGGGTTTTTAATATATATCACT
GCCAGTGTTCGGTTCGTAAGTAATGCCCAAAGTGGTCCGTGGGTTCCGGCTTCTTCATCAGTGGTTCCAGCATATTTCAAGTTGTTTACTGTAGAA
GCAATACCGGAGGAACACCAACTAACAAATTAAGGTCCACACCATCATGATGGACGAATGCCACTTTTTGGACCCGATGTCAATAGCGGCCCGGGG
AGTGATGGATTACTTAAATTTACTATTGTCGTGGGTTGAGAACCACTGA

>c35778_g3_i1_len=473
CTTGTCTGTCAAAAGCCCACGGACAAATGTGGGCTCTGTACTTTTTCCATCGGCGTGATCTGCGGGTGGGGTACGCAGCCATACATGCATCCGTTC
CAACAGATTGGGTCCCAGAGGGGAGGACATCATGGTCCATCCACCAGCAATACGAGTGGATGACCACTGGTGTGCCAAAAACATTGGCCACCAGTG
TAGCGATCGAACGATCGCCATCAACCGATGAAGCATGATGTGATCGAGCTAGGTGAGGCTAGTTCGAAACTAATTAATTTCGATTAACGATCGTCG
GTGGAAGCCCTAGCGGGAGCGGGGCCGATCGAAGGTCGTTGCCAGAACATTGGTGACCCCGACGTGATAAGATTCTTATCACTCCTAAATCGCGTT
CGTGTGAACAAGAGAAAAGTGTTCCATGTCTTGCCGGTGGTGTCAAAAAATTGCATGGCTTTAGTGCTAACCCAAGTGATTTTCCGAAT

>c36162_g1_i2_len=667
GAAACTTAGCCAAATCGTCAATTGATTTCGGTGTTAGTCCAAAATAAGTGATCGTACCTAAAGTGATAAATCAAAAGTGCGAGCAACGAAACGAAA
AATGGGTTCTAGTGATTCAAAGGAAAAGCCCATAGTGGACGACAGTCATGTCATTAATATTCTTAATGCAAATAATAAACTAGCTCAGCACGCCGG
CGAGTCAGCTAAAAACTCTATTCAAATCGCAAACGCACTGACAGTGTTGGCATACATAGCTGTACTAGTGATTGGAGCGCTGTTTATTTACAAATT
GTACAAGGTAGTGACAAAGTACGAACGAATGCGCACAGCGGTGGATAGCAGTAAACCAGATATAGTGCCGCATGTAATTTGAACGAGACCCGCGGA
ACAAAACGAAAAACAAAAAGGTGCGCGTGAAACTGTGAACATTGGGACTGGCATCACCACCCGCATTAGCAAAATGCGTGAAACAAATGCGATTTT
CATTACAAGCCTTGCACTATTAGACAAAAATCATCTATTCGACGGAAATTTCTGAGATGGGAGCCAATTACAACGTTGACCTCGTGATTGACTTGA
GAACAGCTGTGAAACCAGTCATACAAAGTGAGGATGAGGTCATCTTGCGAAAGCAGCCCATCACAAAATCATCTATGGTGCAGCGACGAGG

>c36237_g2_i3_len=240
CAGACCTCCTCCACATACAAGTGTCTTTCTGTCGGTGTCAATACCACACCCCACATCACCATACACAAACCAAGCTGATCCGGGCACGATGAGAAG
CATGAACATGATACTTGATCACTTTTCTGTCGAATGCTCTCATAGTTGTTTTCATGGCGTTCGCATCAATAAAATCAAATGGTGACTGATATATTT
CATCATTCGCTGCTACCGAGCTATGGGGAAGTTTAAAGTGTTTCTTAG

Table S2a. Contig based virus identification of viruses in Cairns *Ae. aegypti*. Contigs were assembled from reads obtained from sequencing Cairns *Ae. aegypti* and were used for virus identification using the NCBI protein and nucleic data bases. Percentage amino acid identity is shown with the number in brackets the percentage of the contig length aligned in the BLAST hit; this was added as the reference sequence was often only a partial sequence. Underlining highlights viruses (or phage) or nearest relatives (italics), which appear to be unique to Cairns mosquitoes, with no reads from Bangkok aligning with the indicated Cairns contig(s) and/or no Cairns contigs overlapping with Bangkok contigs. Grey shading in the **Contig id** column represents contigs that overlap with contigs identified from Bangkok *Ae. aegypti*. [1]Similar match to *Ochlerotatus caspius* flavivirus, Hanko virus, and Parramatta River virus. [2]c50692_g1_i1 is possibly derived from an EVE (see Table S2). [3] Similar match to Blacklegged tick phlebovirus.

Classification	Virus or *nearest match*	Contig id	Amino acid identity	Gene	Length (nucleo-tides)	Mean read depth	E Data ncbi protein	Host	Refs
**Known viruses of *Ae aegypti***									
Genus;<br>*Phlebovirus*<br>ssRNA(-)	Phasi Charoen-like virus	c9421_g1_i1<br>c9498_g1_i1<br>c9500_g1_i1	98.5(60)<br>97(97)<br>99(99)	Nucleocapsid, glycoprotein, RNA polymerase	1339<br>3842<br>6697	62661<br>28142<br>18605	6.49E-175<br>0<br>0	*Ae aegypti*	1,2
Genus;<br>*Flavivirus*<br>ssRNA(+)	Cell fusing agent virus	c9499_g1_i1	99(94)	Polyprotein	10684	664	0	*Ae aegypti*	3
Unclassified<br>ssRNA(+)	Humaita-Tubiacanga virus	c9445_g1_i1<br>c9477_g1_i1	98(80)<br>97(51)	Capsid<br>Replicase	1618<br>2761	16949<br>19494	0<br>0	*Ae aegypti*	2
Unclassified dsRNA (probably a Totivirus)	Unnamed	c7017_g1_i1<br>c8022_g1_i1<br>c8271_g1_i1<br>c9075_g1_i1<br>c9470_g1_i1<br>c37343_g1_i1	96(99)<br>90(99)<br>95(99)<br>97(99)<br>98(41)<br>95(99)	PArp-RdRp, partial<br>(Contig 5425)	238<br>265<br>892<br>619<br>6772<br>253	140<br>437<br>465<br>389<br>1081<br>298	4.85E-43<br>3.79E-50<br>0<br>1.14E-138<br>0<br>1.14E-47	*Ae aegypti*<br>Thailand	4<br>AJT39589.1, KP642128.1
**Mosquito-associated viruses**									
Genus;<br>*Flavivirus*	*Xishuangbanna flavivirus*	c55497_g1_i1[1]	60(79)	RNA polymerase<br>Polyprotein	239	27	3.29E-22	*Ae albopictus*	5
Genus:<br>*Orthomyxovirus*<br>ssRNA(-)	*Whidbey virus*	c9280_g1_i1<br>c9415_g1_i1<br>c25711_g1_i1	85(92)<br>79(90)<br>76(92)	PB1<br>PA<br>PB2	2438<br>2203<br>2417	285<br>299<br>288	0<br>0<br>0	*Aedes dorsalis*	AQU42764.1
Unclassified. Order; *Mononegavirales*<br>ssRNA(-)	*Xincheng mosquito virus*	c4670_g1_i2<br>c4670_g1_i1	37(77)<br>59(81)	Glycoprotein	212<br>251	2<br>17	2.34E-9<br>6E-17	*Anopheles sinensis*	6
Family;<br>*Bunyaviridae*<br>Phasmavirus like.<br>ssRNA(-)	*Wuhan mosquito virus 2*	c7211_g1_i1<br>c8461_g1_i1<br>c8461_g2_i1	34(96)<br>44(96)<br>39(82)	Glycoprotein precursor	447<br>308<br>809	18<br>23<br>12	4.05E-21<br>7.49E-24<br>7.87E-47	*Culex, Anopheles, Aedes sp*	6
Unclassified;<br>*Rhabdoviridae*<br>ssRNA(-)	*Wuhan nosquito virus 9*	c33645_g1_i1<br>c50692_g1_i1 [2]	28(60)<br>37(83)	Glycoprotein<br>ORF1	930<br>383	8.59<br>8.7	1.5E-015<br>1.28E-12	*Culex, Aedes sp*	6
Genus;<br>*Totivirus*<br>dsRNA	*Anopheles totivirus*	c9501_g1_i1<br>c9501_g1_i1	45(37)<br>27(42)	RdRp<br>Putative capsid	6086<br>6086	1106<br>1106	0<br>4.00E-82	*Anopheles gambiae*	7
Unclassified	*Kaiowa virus*	c32637_g1_i1	75(99)	Putative glycoprotein	214	11	3.9e-192	*Brazilian mosquitoes*	8
Unclassified	*Hubei toti-like virus10*	c8605_g1_i1<br>c48383_g1_i1	39(96)<br>46(93)	Hypothetical protein 1	410<br>229	268<br>232	1.50E-16<br>2.8E-15	Mosquito	9
Unclassified	*dsRNA virus environmental sample*	c2748_g1_i1	37(98)	Proline-alanine-rich protein	373	235	1.3E-13	*Ochlero-tatus sierrensis*	4
Oribivirus	*Unnamed*	c9239_g1_i1	87(60)	RdRp<br>AGW51764.1	4038	3543	0	*Ochlerotatus caspius & detritus*	10
**Other insect-associated viruses**									
Unclassified	*Blackford virus*	c177_g1_i1<br>c8631_g1_i2	45(75)<br>44(64)	Putative polyprotein	240<br>333	19<br>37	3.6E-6<br>1.1E-12<br>6.5E-7	*D. tristis*	11

		c8631_g1_i3<br>c9229_g1_i1	47(93)<br>48(55)		203<br>1131	12<br>67	3.9E-4		
Unclassified RNA virus	*Hubei tombus-like virus 40*	c54631_g1_i1<br>c11413_g1_i1	51(96)<br>51(91)	Hypothetical proteins	221<br>693	5.5<br>5.6	2.3E-13<br>1.2E-51	*Coleoptera*	9
Unclassified	*Diaphorina citri associated C virus*	c40769_g1_i1	34(86)	RdRp	1259	7.4	6.07E-48	Psyllid	12
Family: *Rhabdoviridae* ssRNA(-)	*Wuhan ant virus*	c2871_g1_i1<br>c2871_g1_i2<br>c32974_g1_i1<br>c51091_g1_i1	32(92)<br>35(98)<br>53(99)<br>32(91)	RdRp	2961<br>2057<br>2318<br>640	7.9<br>4.7<br>10.6<br>11	9.5E-140<br>5.9E-125<br>0<br>1E-23	*Camponotus japonicus*	6
Family; *Baculoviridae* dsDNA	*Autographa californica nucleo-polyhedrovirus*	c35321_g1_i1	69(72)	ORF B	563	6.6	5.59E-46	*Autographa californica*	13
**Plant viruses**									
Unclassified phlebovirus-like	*Citrus concave gum-associated virus*	c14987_g1_i1	45(99)	RNA-dependent RNA polymerase	548	5.8	8.1E-46	*Citrus sinensis*	14
Genus: *Closterovirus*	*Carrot closterovirus*	c51916_g1_i1	36(95)	ORF2	482	6.6	2.71E-18	Carrot	15
Phenuiviridae ssRNA(-)	*Watermelon crinkle leaf-associated virus 2*	c18669_g1_i1<br>c45853_g1_i1	35(82)<br>49(99)	Nucleocapsid<br>Polymerase	952<br>203	9.8<br>12	7.5E-31<br>5.8E-13	*Citrullus lanatus*	16
**Phage**									
	Enterobacteria phage SP6	c18294_g1_i1	100(53)	Protein 45	339	158	7.4E-36	Entero-bacteria	AAR90038

Table S2b. Contig based virus identification of viruses in Bangkok *Ae aegypti*. Contigs were assembled from reads obtained from sequencing of Bangkok *Ae. aegypti* and were used for virus identification as in Table 1. Underlining as for Table 1 except for Bangkok contigs. Grey shading in the **Contig id** column represents contigs that overlap with contigs identified from Cairns *Ae. aegypti*. [1] Similar match to *Culex pipiens pallens* densovirus. [2] Similar match to Menghai flavivirus. [3] Similar match to Hubei virga-like virus 17.

Classification	Virus or *nearest match*	Contig id	Amino acid identity	Gene	Length (nucleo-tide)	Mean reac depth	E Data ncbi protein	Host	Refs
**Known viruses of *Ae aegypti***									
Genus; *Phlebovirus* ssRNA(-)	Phasi Charoen-like virus	c125955_g1_i1	81(54)	Nucleocapsid	320	4.0	1.22E-22	*Ae aegypti*	1,2
		c33559_g1_i2	69(56)		374	461	3.8E-23		
		c36351_g1_i2	99.6(61)		1327	96046	7.1E-178		
		c38870_g1_i1	54(100)	Glycoprotein	246	5.0	2.1E-28		
		c36369_g1_i1	99(94)		1239	48052	0		
		c36405_g1_i1	99(96)		2738	51100	0		
		c36406_g1_i1	99.6(99)	RdRp	6400	28145	0		
Genus; *Flavivirus* ssRNA(+)	Cell fusing agent virus	c36400_g1_i1	98(94)	Polyprotein	10659	4396	0	*Ae aegypti*	17
Unclassified ssRNA(+)	Humaita-Tubiacanga virus	c36290_g1_i1	98(78)	Capsid	1668	19454	0	*Ae aegypti*	2
		c36361_g1_i1	97(49)	Replicase	2839	18407	0		
Unclassified dsRNA	Unnamed	c36064_g1_i1	91(99)	PArp-RdRp, partial (Contig 5425)	887	266	0	*Ae aegypti Thailand*	[4] AJT39589.1, KP642128.1
		c36389_g2_i2	99(99)		456	305	2E-97		
		c29461_g1_i1	96(100)		238	238	5E-45		
		c33519_g1_i1	97(99)		502	261	2.3E-108		
		c28080_g1_i1	97(37)		284	319	8E-55		
		c36389_g2_i3	96(44)		677	276	1.2E-42		
Subfamily: *Densovirinae* ssDNA	*Aedes aegypti densovirus 2*	c30446_g1_i1[1]	98(61)	Non-structural protein 1 & capsid	3790	106	0	*Ae aegypti*	18
**Mosquito-associated viruses**									
Genus; *Flavivirus*	*Xishuangbanna flavivirus*	c27654_g1_i3	67(57)	RdRp	368	38	4.94E-35	*Ae albopictus*	5,19
		c14012_g1_i1[2]	59(73)	Polyprotein	320	18	7.42E-40		
Genus: *Orthomyxovirus* ssRNA(-)	*Whidbey virus*	c35946_g1_i1	85(92)	PB1	2434	104	0	*Ae dorsalis*	20
		c35196_g1_i1	79(90)	PA, partial	2187	115	0		
		c32973_g1_i1	76(93)	PB2	2402	131	0		
Unclassified. Order; *Mononegavirales* ssRNA(-)	*Xincheng mosquito virus*	c32243_g1_i1	39(72)	Glycoprotein	318	12	3.4E-15	*Anopheles sinensis*	6
		c32243_g1_i2	42(74)		356	43	1.7E-19		
Family; *Bunyaviridae* Phasmavirus like. ssRNA(-)	*Wuhan mosquito virus 2*	c35613_g1_i1	42(78)	Glycoprotein precursor	2049	80	4.2E-118	*Culex, Anopheles, Aedes sp*	6
Family *Rhabdoviridae* ssRNA(-)	*Tongilchon virus 1*	c124296_g1_i1	57(98)	Putative RdRp	280	4.2	2E-26	*Culex bitaenio-rhynchus*	21
Unclassified; *Rhabdoviridae* ssRNA(-)	*Wuhan mosquito virus 9*	c64714_g1_i1	30(80)	Glycoprotein	477	3.3	6E-7	*Culex, Aedes sp*	6
		c1882_g1_i2	37(79)	ORF1	472	10.8	2E-11		
Genus; *Totivirus* dsRNA	*Anopheles totivirus*	c36387_g1_i1	45(36)	RdRp &	6125	1509	0	*Anopheles gambiae*	7
			28(41)	Putative capsid			2.E-107		
Unclassified	*Kaiowa virus*	c15947_g1_i1	73(72)	Putative glycoprotein	1115	5.6	1e-146	*Brazilian mosquitoes*	ASV45863.1
Unclassified virus	*Croada virus*	c32738_g1_i1	68(36)	Putative glycoprotein	529	52	5E-23	*Psorophora pools*	ASV45861.1
		c32738_g1_i3	68(29)		664	31	1.8E-22		
Unclassified RNA virus	*Wenzhou sobemo-like virus 4*	c54150_g1_i1	84(43)	Hypothetical protein	3099	1137	1.6E-48	Mosquito	9
Unclassified virus	*Hubei mosquito virus 2*	c70842_g1_i1	48(83)	Hypothetical protein	1639	1491	8E-142	Mosquito	22
Family; *Chuviridae* ssRNA(-)	*Chuvirus Mos8Chu0*	c914_g1_i1	63(86)	Putative nucleoprotein	836	9.6	6.9E-75	*Culiseta minnesotae*	API61889.1
**Other insect-associated viruses**									
Unclassified	*Blackford virus*	c34693_g2_i2	46(46)	Putative polyprotein	880	44	6.9E-30	*D. tristis*	11
		c34693_g2_i3	46(39)		853	26	5.1E-23		

		c36305_g1_i2	47(36)		1763	45	1.2E-49		
		c36305_g1_i3	47(36)		1757	56	1.1E-49		
		c36305_g1_i5	47(50)		1256	40	2.4E-51		
		c46271_g1_i1	45(52)		538	9.0	2.3E-14		
		c28983_g2_i1	46(33)		4421	409	1.1E-124		
Unclassified	*Chaq virus*	c123170_g1_i1	38(97)	Orf1	521	7.6	9.7E-32	Pachypsylla psyllid	23
		c27006_g1_i1	59(61)		1437	8068	2.8E-111		
Unclassified RNA	*Hubei virga-like virus 12*	c70967_g1_i1	31(88)	Hypothetical protein	511	4.8	1.4E-07	*Dipteria*	24
Family*;* *Baculoviridae* dsDNA	*Autographa californica nucleo-polyhedrovirus*	c39646_g1_i1	56(70)	ORF B	1084	7.0	2E-91	*Spodoptera frugiperda*	25
		c76297_g1_i1	75(99)	ORF B	529	3.7	2E-88		
		c55859_g1_i1	100(48)	AcOrf-4 peptide	395	2.8	3.1E-38		
		c82294_g1_i1	99(99)	AcOrf-5 peptide	206	3	1.10E-41		
**Other**									
Unclassified RNA viruses	*Beihai barnacle virus 12*	c33969_g1_i1	44(91)	RdRp	1727	4527	4.4-163	*Barnacle*	7
**Plant virus**									
Genus *Tobamovirus* ssRNA(+)	*Cucumber fruit mottle mosaic virus*	c36375_g1_i1[3]	28(47)	Unnamed protein	6510	108	5.41E-58	Cucumber	NP_072162.1
**Phage**									
	Spiroplasma phage 4	c40444_g1_i1	36(84)	Hypothetical protein Sp-4p1	345	5.1	4.9E-6		26

References

1. Chandler, J. A. *et al.* Metagenomic shotgun sequencing of a Bunyavirus in wild-caught Aedes aegypti from Thailand informs the evolutionary and genomic history of the Phleboviruses. *Virology* **464-465**, 312-319 (2014).
2. Aguiar, E. R. *et al.* Sequence-independent characterization of viruses based on the pattern of viral small RNAs produced by the host. *Nucleic Acids Res* **43**, 6191-6206 (2015).
3. Yamanaka, A., Thongrungkiat, S., Ramasoota, P. & Konishi, E. Genetic and evolutionary analysis of cell-fusing agent virus based on Thai strains isolated in 2008 and 2012. *Infect Genet Evol* **19**, 188-194 (2013).
4. Chandler, J. A., Liu, R. M. & Bennett, S. N. RNA shotgun metagenomic sequencing of northern California (USA) mosquitoes uncovers viruses, bacteria, and fungi. *Front Microbiol* **6**, 185 (2015).
5. Fan, H. *et al.* Complete genome sequence of Xishuangbanna flavivirus, a novel mosquito-specific flavivirus from China. *Arch Virol* **161**, 1723-1727 (2016).
6. Li, C. X. *et al.* Unprecedented genomic diversity of RNA viruses in arthropods reveals the ancestry of negative-sense RNA viruses. *Elife* **4**, e05378 (2015).
7. Fauver, J. R. *et al.* West African Anopheles gambiae mosquitoes harbor a taxonomically diverse virome including new insect-specific flaviviruses, mononegaviruses, and totiviruses. *Virology* **498**, 288-299 (2016).
8. Pauvolid-Correa, A. *et al.* Novel Viruses Isolated from Mosquitoes in Pantanal, Brazil. *Genome Announc* **4**, e01195-01116 (2016).
9. Shi, M. *et al.* Redefining the invertebrate RNA virosphere. *Nature* **540**, 539–543 (2016).
10. Cook, S. *et al.* Novel virus discovery and genome reconstruction from field RNA samples reveals highly divergent viruses in dipteran hosts. *PLoS One* **8**, e80720 (2013).
11. Webster, C. L., Longdon, B., Lewis, S. H. & Obbard, D. J. Twenty-Five New Viruses Associated with the Drosophilidae (Diptera). *Evol Bioinform Online* **12**, 13-25 (2016).
12. Nouri, S., Salem, N. & Falk, B. W. Complete Genome Sequence of Diaphorina citri-associated C virus, a Novel Putative RNA Virus of the Asian Citrus Psyllid, Diaphorina citri. *Genome Announc* **4**, e00639-00616 (2016).
13. Friesen, P. D. & Nissen, M. S. Gene organization and transcription of TED, a lepidopteran retrotransposon integrated within the baculovirus genome. *Mol Cell Biol* **10**, 3067-3077 (1990).
14. Navarro, B. *et al.* The first phlebo-like virus infecting plants: a case study on the adaptation of negative-stranded RNA viruses to new hosts. *Mol Plant Pathol* (2017).
15. Menzel, W., Goetz, R., Lesemann, D. E. & Vetten, H. J. Molecular characterization of a closterovirus from carrot and its identification as a German isolate of Carrot yellow leaf virus. *Arch Virol* **154**, 1343-1347 (2009).
16. Xin, M. *et al.* Two Negative-Strand RNA Viruses Identified in Watermelon Represent a Novel Clade in the Order Bunyavirales. *Front Microbiol* **8**, 1514 (2017).
17. Bolling, B. G. *et al.* Insect-specific viruses detected in laboratory mosquito colonies and their potential implications for experiments evaluating arbovirus vector competence. *Am J Trop Med Hyg* **92**, 422-428 (2015).
18. Sivaram, A. *et al.* Isolation and characterization of densonucleosis virus from Aedes aegypti mosquitoes and its distribution in India. *Intervirology* **52**, 1-7 (2009).
19. Blitvich, B. J. & Firth, A. E. Insect-specific flaviviruses: a systematic review of their discovery, host range, mode of transmission, superinfection exclusion potential and genomic organization. *Viruses* **7**, 1927-1959 (2015).
20. Makhsous, N. *et al.* Isolation and characterization of the fall Chinook aquareovirus. *Virol J* **14**, 170 (2017).
21. Hang, J. *et al.* Genome Sequences of Five Arboviruses in Field-Captured Mosquitoes in a Unique Rural Environment of South Korea. *Genome Announc* **4**, e01644-01615 (2016).
22. Shi, M. *et al.* High-Resolution Metatranscriptomics Reveals the Ecological Dynamics of Mosquito-Associated RNA Viruses in Western Australia. *J Virol* **91**, e00680-00617 (2017).
23. Webster, C. L. *et al.* The Discovery, Distribution, and Evolution of Viruses Associated with Drosophila melanogaster. *PLoS Biol* **13**, e1002210 (2015).
24. Shi, M. *et al.* Divergent Viruses Discovered in Arthropods and Vertebrates Revise the Evolutionary History of the Flaviviridae and Related Viruses. *J Virol* **90**, 659-669 (2015).
25. Ayres, M. D., Howard, S. C., Kuzio, J., Lopez-Ferber, M. & Possee, R. D. The complete DNA sequence of Autographa californica nuclear polyhedrosis virus. *Virology* **202**, 586-605 (1994).
26. Renaudin, J., Pascarel, M. C. & Bove, J. M. Spiroplasma virus 4: nucleotide sequence of the viral DNA, regulatory signals, and proposed genome organization. *J Bacteriol* **169**, 4950-4961 (1987).

Table S3. Contigs showing homology to EVEs. *c50692 might be derived from a relative of Wuhan mosquito virus 9 (Table 1). ** Closest match was to a reverse transcriptase-like enzyme in a transposable genetic element in *Drosophila melanogaster*. ***EVE based on presence of multiple stop codons.

Contig id from Cairns	Nucleotide identity	Gene	Length (nucleo-tides)	Mean read depth	E Data ncbi protein	Refs
c9237_g1_i1	95	PREDICTED: *Aedes aegypti* uncharacterized LOC110677206	382	16	1.1E-26	1
c25880_g1_i1	99	Uncharacterized protein LOC110677205	513	5.9	2.2E-30	1
c50692_g1_i1*	88	PREDICTED: uncharacterized protein LOC109422677 [*Aedes albopictus*]	383	8.7	2E-40	2
c7681_g1_i1	85	Uncharacterized protein LOC110677235	734	7.9	8.3E-102	1
c49221_g1_i1	84	Uncharacterized protein LOC110677235	303	4.5	2.7E-49	1
c7681_g1_i1	85	Uncharacterized protein LOC110677235	734	7.9	8.3E-102	1
c23353_g1_i1	88	Uncharacterized protein LOC110677235	208	3.2	4.2E-07	1
c118388_g1_i1**	58	Retrovirus-related pol polyprotein	208	2.7	4.3E-06	3
c47288_g1_i1***	69	Polyprotein	272	5.1	1.21E-14	4

Contig id from Bangkok	Nucleotide identity	Gene	Length (nucleo-tides)	Mean read depth	E Data ncbi protein	Refs
c35604_g1_i3	89	uncharacterized protein LOC110677205	633	35	3.5E-67	1
c35604_g1_i6	89	uncharacterized protein LOC110677205	804	16	6.3E-68	1
c15103_g1_i1	97	uncharacterized protein LOC110677205	433	15	7.5E-11	1
c36162_g1_i2	98	uncharacterized protein LOC110677205	677	195	7.6E-24	1
c18481_g1_i1	98	uncharacterized protein LOC110677235	324	7.1	5.1E-59	1
c25987_g1_i1	83	uncharacterized protein LOC110677235	1033	17	5.7E-165	1
c36378_g1_i1	89	uncharacterized protein LOC110677235	12872	665	0	1
c36378_g1_i2	89	uncharacterized protein LOC110677235	10372	599	0	1
c36378_g1_i1	89	uncharacterized protein LOC110677235	12872	665	0	1
c54922_g1_i1	99	Subfamily of reverse transcriptases	1698	26.3	0	5
c36237_g2_i1^	74	Polyprotein	240	91	3.8E-8	4
c27654_g1_i1^	59	Polyprotein	368	38	1.1E-39	6
c35778_g3_i1^	83	Polyprotein	473	27	1.68E-24	4

References

1 Matthews, B. J., McBride, C. S., DeGennaro, M., Despo, O. & Vosshall, L. B. The neurotranscriptome of the Aedes aegypti mosquito. *BMC Genomics* **17**, 32 (2016).

2 Chuang, C. K., Yang, T. H., Chen, T. H., Yang, C. F. & Chen, W. J. Heat shock cognate protein 70 isoform D is required for clathrin-dependent endocytosis of Japanese encephalitis virus in C6/36 cells. *J Gen Virol* **96**, 793-803 (2015).

3 Saigo, K. *et al.* Identification of the coding sequence for a reverse transcriptase-like enzyme in a transposable genetic element in Drosophila melanogaster. *Nature* **312**, 659-661 (1984).

4 Fan, H. *et al.* Complete genome sequence of Xishuangbanna flavivirus, a novel mosquito-specific flavivirus from China. *Arch Virol* **161**, 1723-1727 (2016).
5 Shinnick, T. M., Lerner, R. A. & Sutcliffe, J. G. Nucleotide sequence of Moloney murine leukaemia virus. *Nature* **293**, 543-548 (1981).
6 Cholleti, H. *et al.* Discovery of Novel Viruses in Mosquitoes from the Zambezi Valley of Mozambique. *PLoS One* **11**, e0162751 (2016).

Table S4. Contig based virus identification of mycoviruses. Contigs assembled from *Ae aegypti* reads were used for mycovirus identification using the NCBI protein data base. Underlining in the **Classification** column indicates viruses or nearest relatives that appear to be unique to Cairns or Bangkok *Ae. aegypti*.

Mycoviruses from Cairns *Ae. aegypti*

Classification	Virus or *nearest match*	Contig id	Amino acid identity	Gene	Length (nucleo-tide)	Mean read depth	E Data ncbi protein	Host	Refs
**Mycoviruses**									
Family: Partitiviridae dsRNA	*Botryosphaeria dothidea partitivirus 1*	c7547_g1_i1<br>c35315_g1_i1<br>c27686_g1_i1<br>c28765_g1_i1<br>c43767_g1_i1	62<br>85<br>41<br>66<br>69	RdRp<br>Coat protein	544<br>396<br>615<br>401<br>338	10<br>5.7<br>3.8<br>5.6<br>7.3	5E-54<br>2E-72<br>2E-39<br>3E-33<br>7.9E-13	*Botryosphaeria dothidea*	1
Family: *Partitiviridae*	*Ustilaginoidea virens partitivirus*	c9031_g1_i1<br>c5575_g1_i1<br>c5783_g1_i1<br>c6217_g1_i1	79<br>88<br>70<br>61	RdRp<br>Coat protein	699<br>446<br>733<br>886	14<br>14<br>16<br>15	3.30E-110<br>9.32E-92<br>1.99E-95<br>5.46E-82	*Ustilaginoidea virens*	2
Family: *Partitiviridae*	*Botryotinia fuckeliana partitivirus 1*	c3606_g1_i1	78	RdRp	492	8.9	3.59E-88	*Botryotinia fuckeliana*	NC_010349.1
Family: *Partitiviridae*	Unnamed	c8342_g1_i1	45	RdRp<br>AGW51759.1	1509	55341	1.38E-109	*Fusarium solani*	3
Family: *Chrysoviridae* (clade I) dsRNA	*Tolypocladium cylindrosporum virus 2*	c16223_g1_i1<br>c26707_g1_i1<br>c35080_g1_i1	74<br>42<br>49	RdRp	254<br>500<br>257	10<br>17<br>7.9	6.02E-35<br>2.98E-15<br>3.53E-16	*Tolypocladium cylindrosporum*	4
Genus: *Chrysovirus* (clade II) dsRNA	*Magnaporthe oryzae chrysovirus 3*	c10259_g1_i1<br>c18623_g1_i1<br>c18803_g1_i1<br>c28520_g1_i1<br>c31892_g1_i1<br>c35229_g1_i1<br>c45206_g1_i1	35<br>45<br>63<br>52<br>51<br>36<br>47	Hypothetical proteins	1503<br>378<br>293<br>232<br>286<br>493<br>581	9.5<br>4.4<br>7.5<br>9.3<br>5.9<br>5.4<br>6.7	4.47E-62<br>3.31E-26<br>2.83E-18<br>1.62E-13<br>2.36E-15<br>9.70E-18<br>6.35E-33	*Magnaporthe oryzae*	US201400 37586 A1
Genus: *Chrysovirus*	*Magnaporthe oryzae chrysovirus 1*	c11895_g1_i1<br>c17275_g1_i1<br>c54876_g1_i1<br>c18610_g1_i1	69<br>66<br>39<br>40	RdRp.<br>Hypothetical protein	515<br>561<br>513<br>1073	3.9<br>5.2<br>5.2<br>11	8.42E-77<br>5.98E-81<br>2.69E-35<br>5.12E-30	*Magnaporthe oryzae*	5
Genus: *Chrysovirus*	*Penicillium chrysogenum virus*	c16117_g1_i1<br>c34127_g1_i1<br>c43319_g1_i1<br>c49342_g1_i1	83<br>93<br>79<br>78	RdRp	424<br>361<br>505<br>1296	4.3<br>4.9<br>3.6<br>7.9	2.77E-79<br>1.06E-72<br>2.43E-92<br>0	*Penicillium*	6
		c16636_g1_i1<br>c18833_g1_i1<br>c19064_g1_i1<br>c33782_g1_i1<br>c35317_g1_i1<br>c45192_g1_i1<br>c45834_g1_i1<br>c48065_g1_i1<br>c51680_g1_i1<br>c45395_g1_i1	60<br>59<br>73<br>68<br>83<br>44<br>72<br>82<br>84<br>35	Major capsid protein	213<br>466<br>553<br>204<br>276<br>938<br>494<br>289<br>860<br>228	6.0<br>5.5<br>12<br>6<br>8.7<br>5.4<br>5.2<br>5.3<br>5.6<br>4	7.35E-9<br>6.04E-30<br>1.18E-56<br>1.62E-16<br>4.18E-45<br>2.70E-73<br>1.70E-53<br>1.94E-47<br>1.30E-173<br>0.00002		
		c9510_g1_i1<br>c27061_g1_i1<br>c29121_g1_i1<br>c32979_g1_i1<br>c39328_g1_i1	54<br>54<br>59<br>40<br>80	Hypothetical proteins	536<br>719<br>631<br>449<br>266	5.3<br>3.8<br>4.9<br>12<br>4.3	1.73E-56<br>4.59E-82<br>1.97E-66<br>2.44E-22<br>2.71E-21		
		c19508_g1_i1<br>c27836_g1_i1<br>c29554_g1_i1<br>c31457_g1_i1<br>c37624_g1_i1<br>c45358_g1_i1<br>c48709_g1_i1<br>c53047_g1_i1<br>c53928_g1_i1	66<br>77<br>80<br>81<br>86<br>74<br>77<br>81<br>78	Hypothetical protein	293<br>245<br>383<br>393<br>614<br>208<br>393<br>243<br>458	5.5<br>4.8<br>4.7<br>11<br>7.3<br>8.0<br>6.0<br>4.0<br>4.2	5.11E-33<br>2.11E-36<br>1.34E-69<br>2.26E-43<br>2.63E-127<br>3.77E-28<br>1.08E-65<br>4.49E-41<br>2.66E-82		

Genus; *Totivirus* dsRNA	*Scheffersomyces segobiensis virus L*	c24059_g1_i1	52	Capsid	313	6.0	2.53E-27	*Scheffersomyces segobiensis*	7
		c50237_g1_i1	59		391	4.4	1.73E-45		
		c42138_g1_i1	61	Polyprotein	440	4.2	4.50E-62		
		c45664_g1_i1	51		401	4.4	2.66E-36		
		c28122_g1_i1	53	Polyprotein	369	5.0	3.30E-32		
		c31620_g1_i1	52		321	4.9	7.69E-34		
		c32294_g1_i1	52		309	4.9	2.54E-28		
		c33879_g1_i1	42		403	4.8	1.34E-26		
Genus; *Totivirus* dsRNA	*Ustilago maydis virus H1*	c37453_g1_i1	33	Cap-pol fusion protein	594	5.1	3.31E-6	*Ustilago maydis*	8
		c42393_g1_i1	72		205	6.0	3.62E-29		
		c43689_g1_i1	80		402	4.7	3.25E-69		
		c51753_g1_i1	57	Polyprotein	404	6.8	4.79E-50		
Unclassified ourmia-like virus	*Rhizoctonia solani ourmia-like virus 1 RNA 1*	c6975_g1_i1	36	RdRp	1806	23	1.18E-53	*Rhizoctonia solani*	KP900922.1KP900921
		c13763_g1_i1	33		747	23	2.04E-14		
		c2560_g1_i1	38		2481	189	1.21E-84		
		c45380_g1_i1	56		320	10	1.20E-18		
Unclassified ourmia-like virus	*Botrytis ourmia-like virus*	c17358_g1_i1	30	RdRp	983	8.8	2.82E-16	Botrytis	9
Family *Bunyaviridae* ssRNA(-)	*Fusarium poae negative-stranded virus 2*	c8881_g1_i1	46	RdRp	6498	37	8e-58	*Fusarium poae*	10
Genus: *Victorivirus* dsRNA	*Ustilaginoidea virens RNA virus M*	c25256_g1_i1	35	RdRp	1070	7.1	2.69E-49	*Ustilaginoidea virens*	11
		c51155_g1_i1	44		357	12	3.80E-23		

Mycoviruses from Bangkok *Ae aegypti*

Classification	Virus or *nearest match*	Contig id	Amino acid identity	Gene	Length (nucleo-tide)	Mean read depth	E Data ncbi protein	Host	Refs
**Mycoiruses**									
Family: Ophioviridae ssRNA(-)	*Rhizoctonia solani negative-stranded virus 1*	c31623_g1_i1	31	RdRp	3076	55	9.88E-76	*Rhizoctonia solani*	12
Family: Ophioviridae	*Rhizoctonia solani negative-stranded virus 3*	c26658_g1_i1	29	RdRp	2239	54	8.66E-11	*Rhizoctonia solani*	12
Family *Bunyaviridae* ssRNA(-)	*Fusarium poae negative-stranded virus 2*	c35704_g1_i1	26	RdRp	7947	103	3.35E-118	*Fusarium poae*	10
		c12670_g1_i1	25		1492	39	2.47E-13		

References

1 Wang, L. *et al.* Hypovirulence of the phytopathogenic fungus Botryosphaeria dothidea: association with a coinfecting chrysovirus and a partitivirus. *J Virol* **88**, 7517-7527 (2014).

2 Zhong, J. *et al.* Complete genome sequence and organization of a novel virus from the rice false smut fungus Ustilaginoidea virens. *Virus Genes* **48**, 329-333 (2014).

3 Cook, S. *et al.* Novel virus discovery and genome reconstruction from field RNA samples reveals highly divergent viruses in dipteran hosts. *PLoS One* **8**, e80720 (2013).

4 Herrero, N. & Zabalgogeazcoa, I. Mycoviruses infecting the endophytic and entomopathogenic fungus Tolypocladium cylindrosporum. *Virus Res* **160**, 409-413 (2011).

5 Urayama, S. *et al.* A dsRNA mycovirus, Magnaporthe oryzae chrysovirus 1-B, suppresses vegetative growth and development of the rice blast fungus. *Virology* **448**, 265-273 (2014).

6 Jiang, D. & Ghabrial, S. A. Molecular characterization of Penicillium chrysogenum virus: reconsideration of the taxonomy of the genus Chrysovirus. *J Gen Virol* **85**, 2111-2121 (2004).

7 Taylor, D. J., Ballinger, M. J., Bowman, S. M. & Bruenn, J. A. Virus-host co-evolution under a modified nuclear genetic code. *PeerJ* **1**, e50 (2013).

8 Voth, P. D., Mairura, L., Lockhart, B. E. & May, G. Phylogeography of Ustilago maydis virus H1 in the USA and Mexico. *J Gen Virol* **87**, 3433-3441 (2006).

9 Donaire, L., Rozas, J. & Ayllon, M. A. Molecular characterization of Botrytis ourmia-like virus, a mycovirus close to the plant pathogenic genus Ourmiavirus. *Virology* **489**, 158-164 (2016).

10 Osaki, H., Sasaki, A., Nomiyama, K. & Tomioka, K. Multiple virus infection in a single strain of Fusarium poae shown by deep sequencing. *Virus Genes* (2016).

11 Zhang, T., Jiang, Y., Huang, J. & Dong, W. Complete genome sequence of a putative novel victorivirus from Ustilaginoidea virens. *Arch Virol* **158**, 1403-1406 (2013).

12 Marzano, S. Y. *et al.* Identification of Diverse Mycoviruses through Metatranscriptomics Characterization of the Viromes of Five Major Fungal Plant Pathogens. *J Virol* **90**, 6846-6863 (2016).

Table S5. Mapping of reads to the human genome. All trimmed and quality-controlled reads (before ribosomal filter) were mapped to the human reference genome (ensemble release 75) using STAR v2.5.2a. Genes were derived based on the genome coordinates. In total, 8646 reads from the Cairns and 8744 reads from the Bangkok dataset matched a human gene. Below is a table listing the number of reads matching human genes. Only genes that have at least 5 reads in both datasets (63 genes) are listed. In total, 327 genes were identified that had at least 5 reads in either of the two datasets.

Gene	Bangkok	Cairns
ENSG00000266658 RNA28S5:"RNA, 28S ribosomal 5 [Source:HGNC Symbol;Acc:37659]"	1646	4109
ENSG00000210082 MT-RNR2:mitochondrially encoded 16S RNA [Source:HGNC Symbol;Acc:7471]	118	986
ENSG00000211459 MT-RNR1:mitochondrially encoded 12S RNA [Source:HGNC Symbol;Acc:7470]	34	600
ENSG00000244734 HBB:"hemoglobin, beta [Source:HGNC Symbol;Acc:4827]"	417	126
ENSG00000188536 HBA2:"hemoglobin, alpha 2 [Source:HGNC Symbol;Acc:4824]"	394	85
ENSG00000206172 HBA1:"hemoglobin, alpha 1 [Source:HGNC Symbol;Acc:4823]"	351	87
ENSG00000258486 RN7SL1:"RNA, 7SL, cytoplasmic 1 [Source:HGNC Symbol;Acc:10038]"	280	60
ENSG00000150991 UBC:ubiquitin C [Source:HGNC Symbol;Acc:12468]	57	234
ENSG00000170315 UBB:ubiquitin B [Source:HGNC Symbol;Acc:12463]	111	173
ENSG00000265150 RN7SL2:"RNA, 7SL, cytoplasmic 2 [Source:HGNC Symbol;Acc:23134]"	128	18
ENSG00000226958 CTD-2328D6.1:	16	87
ENSG00000184009 ACTG1:"actin, gamma 1 [Source:HGNC Symbol;Acc:144]"	62	41
ENSG00000143632 ACTA1:"actin, alpha 1, skeletal muscle [Source:HGNC Symbol;Acc:129]"	49	52
ENSG00000263563 UBBP4:ubiquitin B pseudogene 4 [Source:HGNC Symbol;Acc:12467]	29	63
ENSG00000258947 TUBB3:Tubulin beta-3 chain [Source:UniProtKB/Swiss-Prot;Acc:Q13509]	16	60
ENSG00000263740 RN7SL4P:"RNA, 7SL, cytoplasmic 4, pseudogene [Source:HGNC Symbol;Acc:10039]"	54	17
ENSG00000265735 RN7SL5P:"RNA, 7SL, cytoplasmic 5, pseudogene [Source:HGNC Symbol;Acc:10040]"	46	17
ENSG00000147454 SLC25A37:"solute carrier family 25 (mitochondrial iron transporter), member 37 [Source:HGNC Symbol;Acc:29786]"	55	8
ENSG00000107796 ACTA2:"actin, alpha 2, smooth muscle, aorta [Source:HGNC Symbol;Acc:130]"	22	40
ENSG00000234289 H2BFS:"H2B histone family, member S (pseudogene) [Source:HGNC Symbol;Acc:4762]"	22	34
ENSG00000265401 RP11-138I1.4:	20	33
ENSG00000132475 H3F3B:"H3 histone, family 3B (H3.3B) [Source:HGNC Symbol;Acc:4765]"	13	35
ENSG00000197409 HIST1H3D:"histone cluster 1, H3d [Source:HGNC Symbol;Acc:4767]"	12	34
ENSG00000221983 UBA52:ubiquitin A-52 residue ribosomal protein fusion product 1 [Source:HGNC Symbol;Acc:12458]	38	7
ENSG00000152234 ATP5A1:"ATP synthase, H+ transporting, mitochondrial F1 complex, alpha subunit 1, cardiac muscle [Source:HGNC Symbol;Acc:823]"	26	18
ENSG00000075624 ACTB:"actin, beta [Source:HGNC Symbol;Acc:132]"	18	25
ENSG00000202198 RN7SK:"RNA, 7SK small nuclear [Source:HGNC Symbol;Acc:10037]"	37	5
ENSG00000241838 LA16c-3G11.7:	11	27
ENSG00000203852 HIST2H3A:"histone cluster 2, H3a [Source:HGNC Symbol;Acc:20505]"	9	25
ENSG00000204434 POTEKP:"POTE ankyrin domain family, member K, pseudogene [Source:HGNC Symbol;Acc:30182]"	19	15
ENSG00000180139 ACTA2-AS1:ACTA2 antisense RNA 1 [Source:HGNC Symbol;Acc:45169]	11	20
ENSG00000147403 RPL10:ribosomal protein L10 [Source:HGNC Symbol;Acc:10298]	18	13
ENSG00000197903 HIST1H2BK:"histone cluster 1, H2bk [Source:HGNC Symbol;Acc:13954]"	13	17
ENSG00000104765 BNIP3L:BCL2/adenovirus E1B 19kDa interacting protein 3-like [Source:HGNC Symbol;Acc:1085]	23	7
ENSG00000180596 HIST1H2BC:"histone cluster 1, H2bc [Source:HGNC Symbol;Acc:4757]"	8	18

ENSG00000170889 RPS9:ribosomal protein S9 [Source:HGNC Symbol;Acc:10442]	10	15
ENSG00000203811 HIST2H3C:"histone cluster 2, H3c [Source:HGNC Symbol;Acc:20503]"	8	17
ENSG00000196176 HIST1H4A:"histone cluster 1, H4a [Source:HGNC Symbol;Acc:4781]"	10	15
ENSG00000197616 MYH6:"myosin, heavy chain 6, cardiac muscle, alpha [Source:HGNC Symbol;Acc:7576]"	14	10
ENSG00000207507 RNU6-9:"RNA, U6 small nuclear 9 [Source:HGNC Symbol;Acc:34269]"	11	13
ENSG00000230037 UBBP1:ubiquitin B pseudogene 1 [Source:HGNC Symbol;Acc:12464]	6	17
ENSG00000137285 TUBB2B:"tubulin, beta 2B class IIb [Source:HGNC Symbol;Acc:30829]"	12	10
ENSG00000188229 TUBB4B:"tubulin, beta 4B class IVb [Source:HGNC Symbol;Acc:20771]"	10	12
ENSG00000109971 HSPA8:heat shock 70kDa protein 8 [Source:HGNC Symbol;Acc:5241]	8	14
ENSG00000184779 RPS17:ribosomal protein S17 [Source:HGNC Symbol;Acc:10397]	16	5
ENSG00000104904 OAZ1:ornithine decarboxylase antizyme 1 [Source:HGNC Symbol;Acc:8095]	14	6
ENSG00000187537 POTEM:"POTE ankyrin domain family, member M [Source:HGNC Symbol;Acc:37096]"	6	14
ENSG00000083845 RPS5:ribosomal protein S5 [Source:HGNC Symbol;Acc:10426]	6	12
ENSG00000126803 HSPA2:heat shock 70kDa protein 2 [Source:HGNC Symbol;Acc:5235]	10	8
ENSG00000272055 RNU6-6P:"RNA, U6 small nuclear 6, pseudogene [Source:HGNC Symbol;Acc:10228]"	10	8
ENSG00000142534 RPS11:ribosomal protein S11 [Source:HGNC Symbol;Acc:10384]	12	5
ENSG00000206932 RNU6-4P:"RNA, U6 small nuclear 4, pseudogene [Source:HGNC Symbol;Acc:34253]"	7	10
ENSG00000110955 ATP5B:"ATP synthase, H+ transporting, mitochondrial F1 complex, beta polypeptide [Source:HGNC Symbol;Acc:830]"	6	11
ENSG00000127824 TUBA4A:"tubulin, alpha 4a [Source:HGNC Symbol;Acc:12407]"	9	8
ENSG00000156508 EEF1A1:eukaryotic translation elongation factor 1 alpha 1 [Source:HGNC Symbol;Acc:3189]	9	8
ENSG00000196374 HIST1H2BM:"histone cluster 1, H2bm [Source:HGNC Symbol;Acc:4750]"	6	9
ENSG00000100316 RPL3:ribosomal protein L3 [Source:HGNC Symbol;Acc:10332]	9	6
ENSG00000143947 RPS27A:ribosomal protein S27a [Source:HGNC Symbol;Acc:10417]	8	7
ENSG00000123091 RNF11:ring finger protein 11 [Source:HGNC Symbol;Acc:10056]	8	6
ENSG00000207357 RNU6-2:"RNA, U6 small nuclear 2 [Source:HGNC Symbol;Acc:34270]"	7	7
ENSG00000197697 HIST1H2BE:"histone cluster 1, H2be [Source:HGNC Symbol;Acc:4753]"	5	8
ENSG00000206899 RNU6-36P:"RNA, U6 small nuclear 36, pseudogene [Source:HGNC Symbol;Acc:34280]"	5	7
ENSG00000137267 TUBB2A:"tubulin, beta 2A class IIa [Source:HGNC Symbol;Acc:12412]"	5	5

Table S6. Mapping of reads to other chordates. Chordate reads that did not match human sequences (see Fig. 1) were used to search for matches in the nucleotide database. Non *homo sapiens* matches with at least 95% identity and 95% coverage are included. For Bangkok 190 reads mapped to mice/rat genes, for Cairns 79 reads mapped to mice/rat genes. For Bangkok 5 reads mapped to dog genes. For Bangkok 1 read mapped to cow, for Cairns 1 read mapped to cow. The closest *Homo sapiens* genes are included for a random selection of reads and are highlighted in grey.

Bangkok (Mouse/rat)

CTCCAAAGCTATCAAAGTACCGCTGGGTCCAAGGGTAGACAACCAGCAGCCTGCCCAGGGCCTCACCACCA
ACTTCATCGGCGTTCACCTTTCCCCACAGGCCAGAGACAGCAGCCTTCTCAGCATCAGTCAGGTGCACCAT
GATGTCTG

Mus musculus hemoglobin, beta adult t chain (Hbb-bt), mRNA
Sequence ID: NM_008220.5
Identities: 150/150(100%) Gaps: 0/150(0%)

```
Query  1    CTCCAAAGCTATCAAAGTACCGCTGGGTCCAAGGGTAGACAACCAGCAGCCTGCCCAGGG  60
            ||||||||||||||||||||||||||||||||||||||||||||||||||||||||||||
Sbjct  196  CTCCAAAGCTATCAAAGTACCGCTGGGTCCAAGGGTAGACAACCAGCAGCCTGCCCAGGG  137

Query  61   CCTCACCACCAACTTCATCGGCGTTCACCTTTCCCCACAGGCCAGAGACAGCAGCCTTCT  120
            ||||||||||||||||||||||||||||||||||||||||||||||||||||||||||||
Sbjct  136  CCTCACCACCAACTTCATCGGCGTTCACCTTTCCCCACAGGCCAGAGACAGCAGCCTTCT  77

Query  121  CAGCATCAGTCAGGTGCACCATGATGTCTG  150
            ||||||||||||||||||||||||||||||
Sbjct  76   CAGCATCAGTCAGGTGCACCATGATGTCTG  47
```

Homo sapiens hemoglobin subunit beta (HBB), mRNA
Sequence ID: NM_000518.4
Identities: 128/148(86%) Gaps0/148(0%)

```
Query  3    CCAAAGCTATCAAAGTACCGCTGGGTCCAAGGGTAGACAACCAGCAGCCTGCCCAGGGCC  62
            ||||||   |||||| ||| |||||||||||||||||| |||||||||||||||||||||
Sbjct  190  CCAAAGGACTCAAAGAACCTCTGGGTCCAAGGGTAGACCACCAGCAGCCTGCCCAGGGCC  131

Query  63   TCACCACCAACTTCATCGGCGTTCACCTTTCCCCACAGGCCAGAGACAGCAGCCTTCTCA  122
            |||||||||||||||||  |||||||||| ||||||||| |||  || |||| ||||||
Sbjct  130  TCACCACCAACTTCATCCACGTTCACCTTGCCCCACAGGGCAGTAACGGCAGACTTCTCC  71

Query  123  GCATCAGTCAGGTGCACCATGATGTCTG  150
             ||  |||||| ||||||||| ||||||
Sbjct  70   TCAGGAGTCAGATGCACCATGGTGTCTG  43
```

GGGGCCAGCTCCAAGGTGTGCTTCTCATTAGTATACTCCACAGTGCCACCCTTGGCCAGGTCCCTTTGGAA
ATTCCAGGTAAAGCGCAGTGTAATGTCGGCTGTGCCGTTGTACAGCTCCTGCTTCATCTGGGCTCGGCTGG
GTGGGCTG

PREDICTED: Mus musculus piezo-type mechanosensitive ion channel component 1 (Piezo1), transcript variant X6, mRNA
Identities: 150/150(100%) Gaps: 0/150(0%)

```
Query  1     GGGGCCAGCTCCAAGGTGTGCTTCTCATTAGTATACTCCACAGTGCCACCCTTGGCCAGG  60
             ||||||||||||||||||||||||||||||||||||||||||||||||||||||||||||
Sbjct  5458  GGGGCCAGCTCCAAGGTGTGCTTCTCATTAGTATACTCCACAGTGCCACCCTTGGCCAGG  5399

Query  61    TCCCTTTGGAAATTCCAGGTAAAGCGCAGTGTAATGTCGGCTGTGCCGTTGTACAGCTCC  120
             ||||||||||||||||||||||||||||||||||||||||||||||||||||||||||||
Sbjct  5398  TCCCTTTGGAAATTCCAGGTAAAGCGCAGTGTAATGTCGGCTGTGCCGTTGTACAGCTCC  5339

Query  121   TGCTTCATCTGGGCTCGGCTGGGTGGGCTG  150
             ||||||||||||||||||||||||||||||
Sbjct  5338  TGCTTCATCTGGGCTCGGCTGGGTGGGCTG  5309
```

Homo sapiens piezo type mechanosensitive ion channel component 1 (PIEZO1), mRNA
Sequence ID: NM_001142864.3
Identities: 129/150(86%) Gaps: 0/150(0%)

```
Query  1      GGGGCCAGCTCCAAGGTGTGCTTCTCATTAGTATACTCCACAGTGCCACCCTTGGCCAGG  60
              ||||||||  |||   |||||||||| || | ||||||||||||||| ||||| ||||||
Sbjct  7245   GGGGCCAGGGCCAGCATGTGCTTCTCGTTGGCATACTCCACAGTGCCTCCCTTCGCCAGG  7186

Query  61     TCCCTTTGGAAATTCCAGGTAAAGCGCAGTGTAATGTCGGCTGTGCCGTTGTACAGCTCC  120
              ||||| ||||| |||||||| |||||||| || |||||||| ||||||||||| ||||||
Sbjct  7185   TCCCTCTGGAAGTTCCAGGTGAAGCGCAGGGTGATGTCGGCCGTGCCGTTGTAGAGCTCC  7126

Query  121    TGCTTCATCTGGGCTCGGCTGGGTGGGCTG  150
               ||||||||||||| |||||||| || |||
Sbjct  7125   CGCTTCATCTGGGCACGGCTGGGGGGACTG  7096
```

CTCAGACTCAAATATCTTGGCCATATATCTAGGCTCTACTCTGACTANATCATGACTCACANAACCCAATT
ATTTTAACCTACACTCNGCCCCGTGGCGGGTTCCCTGTGCTCATG

Mus musculus targeted non-conditional, lacZ-tagged mutant allele
Siah2:tm1e(EUCOMM)Hmgu; transgenic
Sequence ID: JN948870.1
Identities: 113/116(97%) Gaps: 0/116(0%)

```
Query  1      CTCAGACTCAAATATCTTGGCCATATATCTAGGCTCTACTCTGACTANATCATGACTCAC  60
              ||||||||||||||||||||||||||||||||||||||||||||||| ||||||||||||
Sbjct  5625   CTCAGACTCAAATATCTTGGCCATATATCTAGGCTCTACTCTGACTAGATCATGACTCAC  5566

Query  61     ANAACCCAATTATTTTAACCTACACTCNGCCCCGTGGCGGGTTCCCTGTGCTCATG  116
              | ||||||||||||||||||||||||| ||||||||||||||||||||||||||||
Sbjct  5565   ACAACCCAATTATTTTAACCTACACTCTGCCCCGTGGCGGGTTCCCTGTGCTCATG  5510
```

Homo sapiens chromosome 2, alternate assembly CHM1_1.1
Sequence ID: NC_018913.2
Identities: 24/26(92%) Gaps: 0/26(0%)

```
Query  8          TCAAATATCTTGGCCATATATCTAGG  33
                  |||| |||||||||||||||||| ||
Sbjct  84754503   TCAATTATCTTGGCCATATATCTGGG  84754478
```

GGCAATAACTGACAGATGCTCTCTTGGGAACAATTAACCATTGTTCACAGGCAAGAGCAGGAAAGGGGGTT
TAGTGGTACTTGTGAGCCAGGGCAGCGGCCACTCCAGCCACCACCTTCTGGAAGGCAGCCTGTGCAGCGGG
GGTGAAAT

Mus musculus hemoglobin, beta adult s chain (Hbb-bs), mRNA
Sequence ID: NM_001201391.1
Identities: 147/148(99%) Gaps: 0/148(0%)

```
Query  3    CAATAACTGACAGATGCTCTCTTGGGAACAATTAACCATTGTTCACAGGCAAGAGCAGGA  62
            ||| ||||||||||||||||||||||||||||||||||||||||||||||||||||||||
Sbjct  565  CAACAACTGACAGATGCTCTCTTGGGAACAATTAACCATTGTTCACAGGCAAGAGCAGGA  506

Query  63   AAGGGGGTTTAGTGGTACTTGTGAGCCAGGGCAGCGGCCACTCCAGCCACCACCTTCTGG  122
            ||||||||||||||||||||||||||||||||||||||||||||||||||||||||||||
Sbjct  505  AAGGGGGTTTAGTGGTACTTGTGAGCCAGGGCAGCGGCCACTCCAGCCACCACCTTCTGG  446

Query  123  AAGGCAGCCTGTGCAGCGGGGGTGAAAT  150
            ||||||||||||||||||||||||||||
Sbjct  445  AAGGCAGCCTGTGCAGCGGGGGTGAAAT  418
```

Homo sapiens hemoglobin subunit beta (HBB), mRNA
Sequence ID: NM_000518.4
Identities: 75/93(81%) Gaps: 0/93(0%)

```
Query  56   AGCAGGAAAGGGGGTTTAGTGGTACTTGTGAGCCAGGGCAGCGGCCACTCCAGCCACCAC  115
            |||| ||||| | | |||||| |||||||| |||||||||   ||||| |||||||||||
Sbjct  509  AGCAAGAAAGCGAGCTTAGTGATACTTGTGGGCCAGGGCATTAGCCACACCAGCCACCAC  450

Query  116  CTTCTGGAAGGCAGCCTGTGCAGCGGGGGTGAA  148
             |||||  ||||||||||  | |  ||||||||
Sbjct  449  TTTCTGATAGGCAGCCTGCACTGGTGGGGTGAA  417
```

GCCAGGGCAGCGGCCACTCCAGCCACCACCTTCTGGAAGGCAGCCTGTGCAGCGGGGGTGAAATCCTTGCC
CAGGTGGTGGCCCAGCACAATCACGATCATATTGCCCAGGAGCCTGAAGTTCTCAGGATCCACATGCAGCT
TGTCACAG

TPA_inf: Mus musculus Glna1 gene for globin a1
Sequence ID: LT548150.1

Identities: 150/150(100%) Gaps: 0/150(0%)

```
Query  1    GCCAGGGCAGCGGCCACTCCAGCCACCACCTTCTGGAAGGCAGCCTGTGCAGCGGGGGTG  60
            ||||||||||||||||||||||||||||||||||||||||||||||||||||||||||||
Sbjct  428  GCCAGGGCAGCGGCCACTCCAGCCACCACCTTCTGGAAGGCAGCCTGTGCAGCGGGGGTG  369

Query  61   AAATCCTTGCCCAGGTGGTGGCCCAGCACAATCACGATCATATTGCCCAGGAGCCTGAAG  120
            ||||||||||||||||||||||||||||||||||||||||||||||||||||||||||||
Sbjct  368  AAATCCTTGCCCAGGTGGTGGCCCAGCACAATCACGATCATATTGCCCAGGAGCCTGAAG  309

Query  121  TTCTCAGGATCCACATGCAGCTTGTCACAG  150
            ||||||||||||||||||||||||||||||
Sbjct  308  TTCTCAGGATCCACATGCAGCTTGTCACAG  279
```

Homo sapiens hemoglobin subunit delta (HBD), mRNA
Sequence ID: NM_000519.3
Identities: 124/150(83%) Gaps: 0/150(0%)

```
Query  1    GCCAGGGCAGCGGCCACTCCAGCCACCACCTTCTGGAAGGCAGCCTGTGCAGCGGGGGTG  60
            |||||||||   ||||| |||||||||||||||||  ||||||||||       ||||||
Sbjct  623  GCCAGGGCATTAGCCACACCAGCCACCACCTTCTGATAGGCAGCCTGCATTTGTGGGGTG  564

Query  61   AAATCCTTGCCCAGGTGGTGGCCCAGCACAATCACGATCATATTGCCCAGGAGCCTGAAG  120
            || |||||||| | || | || ||||||||  ||| | || |||||||| ||||||||||
Sbjct  563  AATTCCTTGCCAAAGTTGCGGGCCAGCACACACACCAGCACATTGCCCAAGAGCCTGAAG  504

Query  121  TTCTCAGGATCCACATGCAGCTTGTCACAG  150
            |||||||||||||| |||||||||||||||
Sbjct  503  TTCTCAGGATCCACGTGCAGCTTGTCACAG  474
```

GGTTTTTCTTTTTGTTGTTGTTGTTGTAGTGGTGGTGGTGAGAAATCCTTTTTTTGTTGAAAACGTAGTAG
TGAAAAATTTAATTCTATGGCTTAAAAATGTATCTGTTACCTAAAAATTTGAAGTTTTACAATTCTGTCTC
AACTCCTT

Mus musculus targeted KO-first, conditional ready, lacZ-tagged mutant allele Bco2:tm1a(KOMP)Wtsi; transgenic
Sequence ID: JN954538.1
Identities: 149/150(99%) Gaps: 0/150(0%)

```
Query  1     GGTTTTTCTTTTTGTTGTTGTTGTTGTAGTGGTGGTGGTGAGAAATCCtttttttGTTGA  60
             |||||||||||||||||||||||| |||||||||||||||||||||||||||||||||||
Sbjct  1836  GGTTTTTCTTTTTGTTGTTGTTGTGGTAGTGGTGGTGGTGAGAAATCCTTTTTTTGTTGA  1895

Query  61    AAACGTAGTAGTGAAAAATTTAATTCTATGGCTTAAAAATGTATCTGTTACCTAAAAATT  120
             ||||||||||||||||||||||||||||||||||||||||||||||||||||||||||||
Sbjct  1896  AAACGTAGTAGTGAAAAATTTAATTCTATGGCTTAAAAATGTATCTGTTACCTAAAAATT  1955

Query  121   TGAAGTTTTACAATTCTGTCTCAACTCCTT  150
             ||||||||||||||||||||||||||||||
Sbjct  1956  TGAAGTTTTACAATTCTGTCTCAACTCCTT  1985
```

Homo sapiens chromosome 1 genomic patch of type NOVEL, GRCh38.p7 PATCHES HSCHR1_4_CTG3
Sequence ID: NW_014040926.1
Identities: 35/41(85%) Gaps: 0/41(0%)

```
Query  3       TTTTTCTTTTTGTTGTTGTTGTTGTAGTGGTGGTGGTGAGA  43
               ||||| || |||||||||||||||| || || || ||||||
Sbjct  294088  TTTTTGTTGTTGTTGTTGTTGTTGTTGTTGTTGTTGTGAGA  294048
```

ATTGTCCAAGTAGTCCCTTCTATTATTCACTCTAAAGCCAGAGCCACATGGCTGAAACTGCAGAGTTCTAC
TGCTTGCAGGAACTAGAACATGACCCGCTTGTACTATTACATTATCACCAGCTTTTTGTTCTCCAACTCCT
TCACTACC

Mus musculus BAC clone RP24-408G4 from 14, complete sequence
Sequence ID: AC154759.2
Identities: 148/150(99%) Gaps: 0/150(0%)

```
Query  1    ATTGTCCAAGTAGTCCCTTCTATTATTCACTCTAAAGCCAGAGCCACATGGCTGAAACTG  60
            ||||||||||||||||||||||||||||||||||||||||||||||||||||||||||||
Sbjct  252  ATTGTCCAAGTAGTCCCTTCTATTATTCACTCTAAAGCCAGAGCCACATGGCTGAAACTG  193

Query  61   CAGAGTTCTACTGCTTGCAGGAACTAGAACATGACCCGCTTGTACTATTACATTATCACC  120
            ||||||||| ||||||||||||||||||||||||||||||||||||||||||||||||||
Sbjct  192  CAGAGTTCTGCTGCTTGCAGGAACTAGAACATGACCCGCTTGTACTATTACATTATCACC  133
```

```
Query  121  AGCTTTTTGTTCTCCAACTCCTTCACTACC  150
            |||||| |||||||||||||||||||||||
Sbjct  132  AGCTTTCTGTTCTCCAACTCCTTCACTACC  103
```

Homo sapiens chromosome 1, alternate assembly CHM1_1.1
Sequence ID: NC_018912.2
Identities: 31/36(86%) Gaps: 3/36(8%)

```
Query  14        TCCCTT---CTATTATTCACTCTAAAGCCAGAGCCA  46
                 ||||||   || |||||||||||||||||||| |||
Sbjct  26182260  TCCCTTTACCTCTTATTCACTCTAAAGCCAGATCCA  26182225
```

GGGGCAGCGGCCACTCCAGCCACCACTTCTGGAAGGCAGCCTGTGCAGCGGGGGTGAAATCCTTGCCCAGG
TGGTGGCCCAGCACAATCACGATCATATTGCCCAGGAGCCTGAAGTTCTCAGGATCCACATGCAGCTTGTC
ACAGTGGA

TPA_inf: Mus musculus Glna1 gene for globin a1
Sequence ID: LT548150.1
Identities: 149/150(99%) Gaps: 1/150(0%)

```
Query  2    GGGCAGCGGCCACTCCAGCCACCAC-TTCTGGAAGGCAGCCTGTGCAGCGGGGGTGAAAT  60
            ||||||||||||||||||||||||| ||||||||||||||||||||||||||||||||||
Sbjct  424  GGGCAGCGGCCACTCCAGCCACCACCTTCTGGAAGGCAGCCTGTGCAGCGGGGGTGAAAT  365

Query  61   CCTTGCCCAGGTGGTGGCCCAGCACAATCACGATCATATTGCCCAGGAGCCTGAAGTTCT  120
            ||||||||||||||||||||||||||||||||||||||||||||||||||||||||||||
Sbjct  364  CCTTGCCCAGGTGGTGGCCCAGCACAATCACGATCATATTGCCCAGGAGCCTGAAGTTCT  305

Query  121  CAGGATCCACATGCAGCTTGTCACAGTGGA  150
            ||||||||||||||||||||||||||||||
Sbjct  304  CAGGATCCACATGCAGCTTGTCACAGTGGA  275
```

Homo sapiens hemoglobin subunit beta (HBB), mRNA
Sequence ID: NM_000518.4
Identities: 122/148(82%) Gaps: 1/148(0%)

```
Query  2    GGGCAGCGGCCACTCCAGCCACCACTT-CTGGAAGGCAGCCTGTGCAGCGGGGGTGAAAT  60
            |||||   ||||| ||||||||||||| |||  ||||||||||  | |  |||||||| |
Sbjct  474  GGGCATTAGCCACACCAGCCACCACTTTCTGATAGGCAGCCTGCACTGGTGGGGTGAATT  415

Query  61   CCTTGCCCAGGTGGTGGCCCAGCACAATCACGATCATATTGCCCAGGAGCCTGAAGTTCT  120
            | ||||| | ||| ||| ||||||||   || | ||  ||||||||||||||||||||||
Sbjct  414  CTTTGCCAAAGTGATGGGCCAGCACACAGACCAGCACGTTGCCCAGGAGCCTGAAGTTCT  355

Query  121  CAGGATCCACATGCAGCTTGTCACAGTG  148
            |||||||||| |||||||||||||||||
Sbjct  354  CAGGATCCACGTGCAGCTTGTCACAGTG  327
```

GTTTTCTATATTTCATACAGATATCTCTTTGGGAATGGTATTATTTGGTGAAATTAATGGAGTTAGAATAA
AAATAAATATTTCATAGATTTATATTTTATCACTATATATTTGAAGGCTGAATCTAAATATTGATCTGATA
ATATGAAG

Mus musculus targeted KO-first, conditional ready, lacZ-tagged mutant allele
Bco2:tm1a(KOMP)Wtsi; transgenic
Sequence ID: JN954538.1
Identities: 149/150(99%) Gaps: 0/150(0%)

```
Query  1     GTTTTCTATATTTCATACAGATATCTCTTTGGGAATGGTATTATTTGGTGAAATTAATGG  60
             ||||||||||||||||||||||||||||||||||||||||||||||||||||||||||||
Sbjct  2100  GTTTTCTATATTTCATACAGATATCTCTTTGGGAATGGTATTATTTGGTGAAATTAATGG  2159

Query  61    AGTTAGAATAAAAATAAATATTTCATAGATTTATATTTTATCACTATATATTTGAAGGCT  120
             ||||||||||||||||||||||||||| ||||||||||||||||||||||||||||||||
Sbjct  2160  AGTTAGAATAAAAATAAATATTTCATATATTTATATTTTATCACTATATATTTGAAGGCT  2219

Query  121   GAATCTAAATATTGATCTGATAATATGAAG  150
             ||||||||||||||||||||||||||||||
Sbjct  2220  GAATCTAAATATTGATCTGATAATATGAAG  2249
```

Homo sapiens chromosome 1 genomic patch of type NOVEL, GRCh38.p7 PATCHES
HSCHR1_5_CTG32_1
Sequence ID: NW_014040927.1
Identities: 33/40(83%) Gaps: 2/40(5%)

```
Query  64    TAGAATAAAAATAAATATTTCATAGATTT--ATATTTTAT  101
             ||||| ||||||| ||||||  || ||||  |||||||||
Sbjct  3095  TAGAAAAAAAATATATATTTTTTATATTTTTATATTTTAT  3056
```

GTTTTCTATATTTCATACAGATATCTCTTTGGGAATGGTATTATTTGGTGAAATTAATGGAGTTAGAATAA
AAATAAATATTTCATAGATTTATATTTTATCACTATATATTTGAAGGCTGAATCTAAATATTGATCTGATA
ATATGAAG

Mus musculus targeted KO-first, conditional ready, lacZ-tagged mutant allele Bco2:tm1a(KOMP)Wtsi; transgenic

Identities: 149/150(99%) Gaps: 0/150(0%)

```
Query  1     GTTTTCTATATTTCATACAGATATCTCTTTGGGAATGGTATTATTTGGTGAAATTAATGG  60
             ||||||||||||||||||||||||||||||||||||||||||||||||||||||||||||
Sbjct  2100  GTTTTCTATATTTCATACAGATATCTCTTTGGGAATGGTATTATTTGGTGAAATTAATGG  2159

Query  61    AGTTAGAATAAAAATAAATATTTCATAGATTTATATTTTATCACTATATATTTGAAGGCT  120
             ||||||||||||||||||||||||||| ||||||||||||||||||||||||||||||||
Sbjct  2160  AGTTAGAATAAAAATAAATATTTCATATATTTATATTTTATCACTATATATTTGAAGGCT  2219

Query  121   GAATCTAAATATTGATCTGATAATATGAAG  150
             ||||||||||||||||||||||||||||||
Sbjct  2220  GAATCTAAATATTGATCTGATAATATGAAG  2249
```

PREDICTED: Homo sapiens ST6 beta-galactoside alpha-2,6-sialyltransferase 1 (ST6GAL1), transcript variant X9, mRNA

Sequence ID: NC_018912.2

Identities: 54/69(78%) Gaps:2/69(2%)

```
Query  68         ATAAAAATAAATATTTCATAGATTTATATTTTATCACTATATATTTGAAGGCTGAATCTA  127
                  ||||| |||||||||| ||| |||||    | ||| | ||||||||| |     ||||| ||
Sbjct  216475256  ATAAATATAAATATTTGATATATTTAA--TATATTAATATATATTTAATATATGAATATA  216475313

Query  128        AATATTGAT  136
                  |||||||||
Sbjct  216475314  AATATTGAT  216475322
```

>DB7DT8Q1:335:H7TWNBCXX:1:2116:8111:46127_2:N:0:ACTGAT/1
ATATCCTCCAGATACATCCATTTGTCCAAGAATTTCATAAATCCATTGTTTTTAATAGCTGAGTAGTACTC
CATTGTGTAAATGTACCACATTTTCTGTATCCATTCTTCTGTTGAGGGACATCTGGGTTCTTTCTAGCTTC
TGGCTATT
>DB7DT8Q1:335:H7TWNBCXX:1:1102:12394:3470_1:N:0:ACTGAT/1
GGTTTTTCTTTTTGTTGTTGTTGTTGTAGTGGTGGTGGTGAGAAATCCTTTTTTTGTTGAAAACGTAGTAG
TGAAAAATTTAATTCTATGGCTTAAAAATGTATCTGTTACCTAAAAATTTGAAGTTTTACAATTCTGTCTC
AACTCCTT
>DB7DT8Q1:335:H7TWNBCXX:1:2211:1529:93710/1
ATACATTCTAGCATCCCAGAATGTCAGGATGGATGATCCTCCAGCACCATGTCCTCGCTGATTATCATGGT
TGTCCACAAACACAAGGGCTCTGTAAGAAGGCACCAAACCC
>DB7DT8Q1:335:H7TWNBCXX:1:1110:6771:65395/1
CATTGTTATTGGGTGATCCAATCCAGTCATTCTGATCTTTTCCATTCTGGAAATTTCTATTCCAACGGTAA
CTTGACATTACTCTTGTGAATCCATAAGGATGAG
>DB7DT8Q1:335:H7TWNBCXX:1:1114:4497:35153/1
CTCTTTTCCGGCATCTCCGCAGACTGTGGCGGGCCATGCACCTCGGCTAGCTGCTCCAGTGTGCCCACTCT
CTGGCTGAGATGGTCTCTCTCCTGCATGAGTTGCTT
>DB7DT8Q1:335:H7TWNBCXX:1:1203:8315:17038/1
GAGCAGGGACTCCGAGAAGCTCCCACTTCACACCAGCCAGCATCAAAATTTCTGTTGGGGTCTACACCAAA
GCAGGAACTTCCAGCCGTAGTAGAGCGGGTTTTTCTCCACATTCGGT
>DB7DT8Q1:335:H7TWNBCXX:1:1205:19273:49897/1
GTGTGTGCGTACTTCATTGCTCAATTCAATTAAGCTCTCTATTCTTGATTTACTACTAAATCCTCCTTAGT
CATTTAGTTTCATAAAGGGTATAGTAATGTTCTTT
>DB7DT8Q1:335:H7TWNBCXX:1:1207:15077:37007/1
CTCGGTTATGCTTGCAGACAGGAGTCTAGCATGGCTGTCCTCTGGGAGGCTCCACCTAGAAGCTGACTAAG
ACAGAGGCAGACACCCACAGTCAATCAGTACATGGAGCTTGGGGACTCTTATGAAAAAACAGGAGGAAGGA
CTGCGGGCCCTTAAG
>DB7DT8Q1:335:H7TWNBCXX:1:1216:7154:71942/1

GGAAATTTCTATTCCAACGGTAACTTGACATTACTCTTGTGAATCCATAAGGATGAGCCAACATAAATCCA
ACAGCCATTTTATACATTCTAGCATCCCAGAATGTCAGGATGGATGATCCTCCAGCACCATGTCCTCGCTG
ATTATC
>DB7DT8Q1:335:H7TWNBCXX:1:2102:20267:92440/1
CTATCAGTTCAATTCAAGTCACGAAGGTAAATGATTTTTGTATCCTAAACATCCACACTTTCCCCGTCCTT
GAGAGAACATTTATGCTCTCCTGCCTTGCTTCTTAC
>DB7DT8Q1:335:H7TWNBCXX:1:2103:13894:36646/1
CTCCGCTGTCCGCTCTCCGTGGACCTTATTGTCTCTCGTGCGGATGTTCACCGTGCCACTGGCTTTCTCTT
TTTCACAACAACAAGGATGAAGTTATACTGTGCTAACTGTGCGTTTCTGATCTTCTTATTCAAGGTACAGC
CTGGATCCAGG
>DB7DT8Q1:335:H7TWNBCXX:1:2105:14331:20671/1
CAGAATGATATTTTAATTAAAGAAAAAACTTCACATAGTGAATTAATTCTCAAAGTTGTGACTATATCATT
TCACTAAAACCACTGACAGCATGTGAAGAAC
>DB7DT8Q1:335:H7TWNBCXX:1:2113:6309:69723/1
TGATGACCTCCGCATCCCATCCGTGAGTGTCGGCTGCTTCCCAATCACAGCATAGCCATTCCAGATGTACA
TCATTTCCAGAGCAGGGATCGGCAAGGAGATGGGGTTGGGGGTGAGTTATCGCCTGGACTTCCGGATAGCA
AACTTCTCTTTGGGTAGAGATTTCCCAGCAATCTTGAGCTTCAAG
>DB7DT8Q1:335:H7TWNBCXX:1:2114:20249:12454/1
TGACATTTTGCTGGGAGCAGACGGCCTGGACATCTGCCAAGGGCTCATGCACGAAGGTGTTCACGGGCTTG
CATGACCCATTTGTCATAACCCGGCGTTTCATCATTTGGTTGCAGTAGGTGGG
>DB7DT8Q1:335:H7TWNBCXX:1:2213:12022:7981/1
CACAAGTAGTGTCTGCATTAATGGTCACTTCTTTTGTTAATCCATTGTTATTGGGTGGTCCAATCCAGTCA
TTCTGATCTTTTCCATTCTGGAAATTTCTATTCCAACGGTAACTTGACATTACTCTTGTGAATCCATAAGG
ATGAG
>DB7DT8Q1:335:H7TWNBCXX:1:1103:14461:85528/1
GGCAATGGTCTCCTCAATCCAATCAATGAAGGCTGAGACACGGGTGAACACTGTGGGCTTCCTCAGGGTGT
TGCAGCCCAAGGAGGACACAAAGCTGGTCACAC
>DB7DT8Q1:335:H7TWNBCXX:1:1106:18271:50581/1
ATCTCTGGTCAGCTTCCACATGATGATGGTCTTGTCTCGAGACGCAGACAGGATCATGTCCGGGAACTGCG
GTGTGGTGGCGATCTGTGTTACCCATCCATTGTGGCACTTAAGGGTCCCGCGAAGGGTCATCTGCTCGGTC
ATGGCTGCA
>DB7DT8Q1:335:H7TWNBCXX:1:1205:9059:22981/1
AAAATTATTTATACAGCCAAAAGAAATGTAGACAATTAATTTGGGAGGTGGCTTGTAAGAGAACAGCAAAG
AGTAAGACTGAAGGCTTTGCTTGGTATTTATAATCAGTGTATCAATTTTGAAGAAG
>DB7DT8Q1:335:H7TWNBCXX:1:1205:8228:84018/1
AAAAGACACAGTGTGGTCCTCGGGGTCCTGAGTTATATTTTCCTTGGCTAGATAACGAACTCTGTATGTGT
AAGGACCTCTTTGTTTAACCTTGATTTTGCTGCTGTTCTTTGCCACGTCATCTGGGTTTTGCACATCAAAG
>DB7DT8Q1:335:H7TWNBCXX:1:1210:5910:31619/1
ATCAACTTGCTGCTATGAGCAAGAATCTCAATATCAGACTGCCAGGTACAAATCCCAGCTTGCCTCAGCTC
TGTGTGATCTCAAGCAAGCAACTTTCCTGGGCCTCAGTTTCCGCTCTGAAAAAAA
>DB7DT8Q1:335:H7TWNBCXX:1:1211:2112:27274/1
CTAAAAGCTACTTGGTTGCTGTTATTATCCCACCAGTTTGAAAAAGGCTGACCATTGACCACATTCCTGAA
GGCAACCATGTTCCTGATTTGACGCCATCTGTGTTCACAGACCCAGTCATTGCCACAAGTAGTGTCTGC
>DB7DT8Q1:335:H7TWNBCXX:1:1214:15277:12688/1
GGGGAAGCTAGCAAACATCCTTTCCAGGGCTTCAGCTCCATATTCAGCACCATGGCCACCAATCTTCCCCC
AGGCAGCCTTGATGTTGCTTTTGTCTTCCCCAGAGAGCACCATGGTTT
>DB7DT8Q1:335:H7TWNBCXX:1:1214:15119:99237/1
CATTGACCACATTCCTGAAGGCAACCATATTCCTGATTTGACGCCATCTGTGTTCACAGACCCAGTCATTG
CCACAAGTAGTGTCTGCATTAATG
>DB7DT8Q1:335:H7TWNBCXX:1:1215:3017:49689/1
GGGAAGATAATGCAAGATAATGATTGATACAGATAGACAGACAGACAGATATGTAGATGATAGACTGATAG
ATGATAAATGGATAATAAATAAAAGATAATTGATTGATAGACATCTGATATGCATACAGAGATGAGAGAAA
GAGATGATGGAT
>DB7DT8Q1:335:H7TWNBCXX:1:2109:19484:78572/1
CTTGTCACCATGTCTCTGAATTCATCTTCATTTCCAGACCTTGTGCAGATTTTATAGCTGATTGGTTGGTA
TCTTTCCCACCAAGGTCTTGATGGGTTATGAACTACAACGTTTT
>DB7DT8Q1:335:H7TWNBCXX:1:2113:9421:74485/1

CTTGGTGCCATGCAGAGTGGAAAGCTCCTTTGCTGCACCTTTCACCAGGGCATTCAGCTCCTCATAGTTCT
CGGGCAGCTTATAGTCGTAGGAGTAAGGATAGAGCATCATCTGTGAGTAGGAGTGGACGGGCAGATAGGCC
TTGATGGAGGAGAG
>DB7DT8Q1:335:H7TWNBCXX:1:2115:3674:64300/1
AGGGAAGTAGGTCTTGGTGGTGGGGAAGCTAGCAAACATCCTTTCCAGGGCTTCAGCTCCATATTCAGCAC
CATGGCCACCAATCTTCCCCCAGGCAGCCTTGATGTTGCTT
>DB7DT8Q1:335:H7TWNBCXX:1:2203:17211:64363/1
CACGACATATAGCAAGAAAAACACGAGGGAAAACCTTGTGGCAGGAAAGAGAAAGCCTGACAAAATGCTTA
AAAAGATATCCTGACAAGGAATGCAATCCTGGCAGGAAGCCTGGTAAAGGTGAG
>DB7DT8Q1:335:H7TWNBCXX:1:2214:4980:60420/1
GTTACCAAGAGATCACATTGTGTCTGAGGTTTTACAAGGCAAGAGAGTGAGCAGGATGTCTTCTCTCACGG
TCTCTTCACTACAGAAGTTGTGCTCTGCCGCTCCTCCCCCTTTTGCACAGTGATCT
>DB7DT8Q1:335:H7TWNBCXX:1:1107:8750:72066/1
CATACCTTTAATCCCAGCACTTGGGAAGCAGAAACAGGTGGATCTGGGTGAGTTCAAGGCCAGCCTGCTCT
ACAAAGTGATTTCCAGATCTCATAACAGAGATCCTGTCTCAAAAAATAAAAATAAAAAACAAACTAAGAGT
GTAAACAGAGTAGAAGGGCTGGGGGAAGGAAGAGGGTCACACAGGCAAGTAATTAATACCCCAGGAGAGCA
GTCC
>DB7DT8Q1:335:H7TWNBCXX:1:1115:8712:79654/1
CGAAAATCTAAGTATTTGCTCAGATCCAGACAGCTGGGGCAGTCTGCCGCCAGAGTATAATGGCAAAGCAG
CTGTAGGTTACAGAGGCAGACATCCTCCTGAGAGGTCGGCCAGCGGAAATATTGGAGACCAAGGGTTCCCC
AACTCCAGCTCGGAACAGCTTGACCCACCACTGGAGTCCCG
>DB7DT8Q1:335:H7TWNBCXX:1:1111:2379:40745/1
CCGCCTTCTCCAATCTCCTTTGAGCCCCACTCAACCAATGAGCTTCAGGACAAGCCTGCTGGCACAGCCAG
ACATGAACTTGACACAGTTTTCACAAGATGGATCCTCACCTCACTTGGCACCAACACAGAACCACAGCGGC
CTGTCACCATGCATGGAACGGTG
>DB7DT8Q1:335:H7TWNBCXX:1:1202:2314:49980/1
CTCTGTGTGTAGCTGTCAAGAAGGAAGTTTCTCTTTGTTTTAGGTCTTACCTCACTGTACCATTACCCTGC
CCTGCGTCTGTGTCTGCTGGCTGTAAAGTGGTCATCATAATGCATTTCAC
>DB7DT8Q1:335:H7TWNBCXX:1:1209:17766:100451/1
ATACATTCTAGCATCCCAGAATGTCAGGATGGATGATCCTCCAGCACCATGTCCTCCTGATTATCATGGTT
GTCCACAAACACAAGGGCTCTGTCAGAAGGCACCAAACCCCAACCTTCTCCCCAGTTCTTTAAATAGGACA
TCTTCTCGCCATTCCACTTGCG
>DB7DT8Q1:335:H7TWNBCXX:1:2104:19436:92690/1
GTGTGTGCGTACTTCATTGCTCAATTCAATTAAGCTCTCTATTCTTGATTTACTACTAAATCCTCCTTAGT
CATTTAGTTTCATAAAGGGTATAGTAATGTTCTTT
>DB7DT8Q1:335:H7TWNBCXX:1:2110:18323:6437/1
CTTTAATTGCCTCACCACCCAGATCAATGACCTCTTGGAAAATGAAAGGTCTGCTTCCTTGGGAGAACCAT
TTTGTATTGAGATTATGCAGTTTGTCCAAAACTGCCTTTATGTCTCCAGGCCACATGTGCTTAG
>DB7DT8Q1:335:H7TWNBCXX:1:2114:17772:91850/1
CTTACATCAAAGTGAGGGAAGTAGGTCTTGGTGGTGGGGAAGCTAGCAAACATCCTTTCCAGGGCTTCAGC
TCCATATTCAGCACCATGGCCACCAATCTTCCCCCAGGCAGCCTTGATGTTGCTTTTGTCTTCCCCAGAGA
GCACCATGGTTT
>DB7DT8Q1:335:H7TWNBCXX:1:2203:18248:43865/1
TGGTAGAGTCTGCTTGTTTTCCCAGGATATCTGTCAGCGTAATGACCCATCTGGGGGCATCCTCCACTCCC
ACATGGGAAGCACTTATTTGCCGTGAAAAGACTGTAAGAGCTACAGGAGAACCCAGCAAAGCCAGTTGGGT
TGACGATGCTG
>DB7DT8Q1:335:H7TWNBCXX:1:2208:11180:43129/1
CAGTGACTCTCTTAGAAACAAGGTCTGGATTATCATTGGCAACTTGTTGAATCCATGCCTCTATCGTTTCC
CAGTTGTTGTACTTGGTGTAGCTGTGTCCACTTGCACGGGTGTGGC
>DB7DT8Q1:335:H7TWNBCXX:1:2212:4178:69279/1
GCCCTTGCGATCGAGAGGACCTACACAGGTTCAGCATTGGAAGATCGGTCATATTCTCCCAAAACGACAAA
GTGGCGTCCAGGCGTGACTTGGCAGTGGGCAGCCG
>DB7DT8Q1:335:H7TWNBCXX:1:2215:8024:11113/1
ATCGATGTCTTGGTATTGTGCAATATCCATGCTGGCCATGAAAAGCTTACCAAAACATAATTGATATCCTT
AACTTTTAAAAACTGAATATTACAGTGCATTATGCAAATGGTGTATAAGGAACCAAATGACTTCTTATGAT
GCCAGGAATAATATGCTTCAAAAGCAGTAGACATT
>DB7DT8Q1:335:H7TWNBCXX:1:1102:11220:13383/1

TATCCCTTTTACTCAATTATACTGGTGGTAGTTTTACTCGACCTTCCCAGTTTAAGAGTCTCCATTAAAGA
AGAGACAATTTTAATACCTATGTGCATAAAAATAG
>DB7DT8Q1:335:H7TWNBCXX:1:1106:8906:31631/1
GTTAAAACAAGCCCAGGGCCTCTCAAGTATTTTCTTTGGATGCTTCAATTCCAACAAGGTGTTACGGTAGG
GTAGTCCCCACTGCTAATCAAAACCGACCATGATACCACTTTGGTCTCGATACAATACTGCTTGCTTGATT
CTTG
>DB7DT8Q1:335:H7TWNBCXX:1:1115:19761:73354/1
GTCACCTTCTCTATGTGGGCTTTGGCACAGTCATCATTAGGCAGGAGCTTGAGGTTCACACACTGGAGCTC
ATCTGGGTATTCATATTTGACGGGTGTAATGCTG
>DB7DT8Q1:335:H7TWNBCXX:1:1201:4510:15062/1
GGCAAGAGCAGGAAAGGGGGTTTAGTGGTACTTGTGAGCCAGGGCAGCGGCCACTCCAGCCACCACCTTCT
GGAAGGCAGCCTGTGCAGCGGGGGTGAAATCCTTGCCCAGGTGGTGGCCCAGCACAATCACGATCATATTG
CCCAGGAGCCTGAAGTTCTCAGGATCCACATGCA
>DB7DT8Q1:335:H7TWNBCXX:1:1206:3694:26684/1
GTTTGCATTATAGTTGGGGTGGCGGATGATTTTAGCTGCATCAATGAATTGCTCACCACCCTCAAGGGCAT
CAATGTTGTGTTCTCCGAGGCGCACCTGAATTCGGGATTTGTAGCAGTGAGCGGCTGAAACAACCCACTGG
GAATTGATGAG
>DB7DT8Q1:335:H7TWNBCXX:1:1214:19172:59691/1
CAGCAGAGTTACTAATGGAAAAGTGAGCTTTGCCATCACTGCCAACATTCACTCTAAGTCCAGTGCAATTG
CCATCGACCTTATCTCCAGAGATGACATCACAGTATGTGCCAGCAGGAAGACCAG
>DB7DT8Q1:335:H7TWNBCXX:1:2107:18910:77618/1
CGGTGTTGAGGTAAAATGTCTGGTAGAGTCTGCTTGTTTTCCCAGGATATCTGTCAGCGTAATGACCCATC
TGGGGGCATCCTCCACTCCCACATGGGAAGCACTTATTTGCC
>DB7DT8Q1:335:H7TWNBCXX:1:2112:1665:97945/1
CACATCTTCATTCACACAAAGGGTCTGCCTAGAGCCAAGTGAGACTAGCCGGGTCTCCTTGCCAAAAGGGC
TCTTCAGTACTTCCCGCACAAACTGGGCCAGCT
>DB7DT8Q1:335:H7TWNBCXX:1:2116:5028:39517/1
CAAATCTCTTTAATATAGATTCTTACAATTTTGAGTCAGCATGGATTGAAATAAATGGGTCCTCAGCAGAG
TTACAATGGAAAAGTGAGCTTTGC
>DB7DT8Q1:335:H7TWNBCXX:1:1212:13386:79759/1
GGCCAGTCAGACGACAATTTCTGACCTGATAAGCATCATTGTAGTGATCAATTTCTCCATTACATTTATTA
TCGTTAAAGTCCCAAGCAGAGTATGGAACTGCTGGGAATTCCCTGTTATTTGGATTGAGG
>DB7DT8Q1:335:H7TWNBCXX:1:1215:1840:78390/1
CTTTAATTGCCTCACCACCCAGATCAATGACCTCTTGGAAAATGAAAGGTCTGCTTCCTTGGGAGAACCAT
TTTGTATTGAGATTATGCAGTTTGTCCAAAACTGCCTTTATGTCTCCAGGCCACATGTGCTTAG
>DB7DT8Q1:335:H7TWNBCXX:1:1215:4982:79576/1
GTTGGTGCCAGAGCTCAGGGTGTTGCCCCAGCCAGACACAAGACACCTAGTACCAGCTGATGGACAAGATC
TTGGCAGAGCGACAGTGGACACTCGAGAGTTGAGGGTGGCAGCT
>DB7DT8Q1:335:H7TWNBCXX:1:1216:14138:68179/1
TGCAATTACAGTTTAGACATCTTTATGTATATTATGGCTGAAATACATTTTACCGAATTCCCAGTCAAAAT
GCTTTTTCAAGGTTGGAAATGGAGGGAGAGCAACTAGTGATTACATTTTTCAAGAAGCTCTGGAATGCTAG
TCATTAGAGTCAGCGTGAGTGGCTACACTTGCTTGTATTTGTCATGGCTCTGGTCTCTCTTTG
>DB7DT8Q1:335:H7TWNBCXX:1:1216:5237:70006/1
CAAGGGAAGCAGTCATTGTGCTGGAACTTCTCATAGGAGGAACAGGGGTACCCCAGGAAGCCGTCAGGGTT
GAGGATGCTGCTGGCATAGTACTTGTAGCTCCGTAGGTGGTGGCAGGCTGCGAAGTTTCGAG
>DB7DT8Q1:335:H7TWNBCXX:1:2106:9473:25771/1
CATGGATTGCAATAAATGGGTCCTCAGCAGAGTTACTAATGGAAAAGTGAGCTTTGCCATCACTGCCAACA
TTCACTCTAAGTCCAGTGCAATTGCCATCGACCTTATCTCCAGAGATGACATCACAGTATGTGCCAGCAGG
AAGACCAG
>DB7DT8Q1:335:H7TWNBCXX:1:2109:14041:3970/1
CAGACCTTGTGCAGATTTTATAGCTGATTGGTTGGTATCTTTCCCACCAAGGTCTTGATGGGTTATGAACT
ACAACGTTTTCATTGGGTGGAGAGACCTGCA
>DB7DT8Q1:335:H7TWNBCXX:1:2110:12379:37457/1
CTAAACTGTTGTTTTTTTCCCCTCAAGTCATGATTTAGGCAATGCACGATTGTAATAATTCATTCATTATA
TATAGGCACATTAACATAAATATTGCACAAAATATGCCTTTAAATTAAAGCGAGAGGTATAAAAGCATGGT
TTACT
>DB7DT8Q1:335:H7TWNBCXX:1:2116:11456:6795/1

CTGGAGAGCAGTTTCGAGGCTGACGCTCATGTCCAGTGGGTAGGCACACTGGAAGTTGACATTGACACGAA
AGTCCCTGATAATAGAGTCAGTGGCCAAGGAAAGGGTGTT
>DB7DT8Q1:335:H7TWNBCXX:1:2202:7890:83493/1
GGCATCCTGGCATTTCGATTCCTCCGTTTGGAAAGAAATCTAAGTGACCCACAGTCTGGCTCATTCCAAAT
CCTAAGTTGGGGATAATGGGCCCGGCGTCTGTG
>DB7DT8Q1:335:H7TWNBCXX:1:2204:11970:87499/1
CTCTCACATCTGCACTGAAATGCAGCCCCAGTATCCAAACTTTCACACCTAACTTGGCTAAAATAAACAAA
TCAACCAAAACAAACCCTCCCTCTGAATGGTTAGGCAGAATTACTAAGGAGGATCACAGAAACAGGGTCAG
CCCAAACGCTTC
>DB7DT8Q1:335:H7TWNBCXX:1:2211:5474:39846/1
TAAAGTCCCAAGCAGAGTATGGAACTGCTGGGAATTCCCTGTTATTTGGATTGAGGAACTTCCACAGGTAC
TGCTTGTTCCTGCAGGATTGCCTGCGCCACACATGTGGTTAATGACAGC
>DB7DT8Q1:335:H7TWNBCXX:1:2214:3939:49021/1
GCATGGTCTGCCCCCATCCATTTGGGGTAGATAGGCATCCGTGAAGGGTGGGAAAACAGGTAAGAATAGGT
CTTGGCGCTCTTGGCATGGGCTTTGTGCTGGGAGCGCTATCTCCGTGGGGATCAG
>DB7DT8Q1:335:H7TWNBCXX:1:1104:2991:58008/1
CCCAGGCGCCTTCCGCGGCCATGTCGTTGAGGCCCAGCCAGATGTTCGCATCGTTGCCCACGCTGTGGCGC
GCGTACTCGAACAGCGCCTCGTTCTCTAGCTCTGACTGCG
>DB7DT8Q1:335:H7TWNBCXX:1:1109:12552:30139/1
CAACAGTCCTACCACTGGTAGAGTTGGTTATAGCCCACTCGGCCTCCTTCTGTGTCTTAAAGTCTGCTAAG
ACCAACAAGAAAGGGGACTAGGCCATGATTCAC
>DB7DT8Q1:335:H7TWNBCXX:1:1111:20110:66871/1
CGGGGTTTCTAGCTGTAATTCTTTTAGTTAGAAGTTTTCTAGTTAGTTCATTATGCAAAAGGTACAAGGTT
TAATCTTTGCTTGTTCTTACTTTTAATTAGTCTTTCATCTTTCCCTTGCGGTACTAGTTCTATAGCTCCTA
G
>DB7DT8Q1:335:H7TWNBCXX:1:1201:7982:55637/1
CACACAAAGACTCAACAAAGAAAAAGAAGAAAAAGAACTTCAGACCAATTTCCCTTATGAATATCAATGCA
AAAATACTCAATAATTTCACACAAACCAAATCCAAG
>DB7DT8Q1:335:H7TWNBCXX:1:1202:19962:21147/1
GCCACATGTGCTTAGCAGCATCAAGTCTGAACCCTGCTACTCCAATGTCAATGAGATGGTTCATATAGTCA
GCCACCTTGGTACGAACATAATCTTTCTC
>DB7DT8Q1:335:H7TWNBCXX:1:1101:8440:49046/1
GTCAAGAAGTCGTTTCATGTGGATCTCTTGTTTATAGGTACGGACAGCCTCTCTCACAAACCACTGACAGA
ATGCAGGAGAAATCCACTCTCTTGCGTGGAAACCACAG
>DB7DT8Q1:335:H7TWNBCXX:1:1102:6186:94075/1
GTAAACTACTTATGTGTCTAGAAGTTAAAGCCACAAGTCTTAGCTACTCAGTTTATGGCTGAAATCCAATT
CGTGGACCTCAGACAAGAGTCCACCCAGTCTCAAAAACATCTTCTGAGATAAGAATGGACCAAATTAGTGA
TGGGAAAGCAGATCAGCAGGGCAACC
>DB7DT8Q1:335:H7TWNBCXX:1:2102:18920:22685/1
AATAAATGGGTCCTCAGCAGAGTTACTAATGGAAAAGTGAGCTTTGCCATCACTGCCAACATTCACTCTAA
GTCCAGTGCAATTACCATCGACCTTATCTCCAGAGATGACATCAC
>DB7DT8Q1:335:H7TWNBCXX:1:2106:8013:85011/1
CAGGACTGTTCCCATTGGTCTGCAGCAGGCCCCAGCCTGTGACATAGCAGACATAGTTTCTCGGGAGAATG
GTGCCAGCGGGTGGGAGGCAAGCTGTCTGGATGTTCTTGCTCAGGGTCAC
>DB7DT8Q1:335:H7TWNBCXX:1:2106:15147:85417/1
CTGGGTACCAGAACAGGGAGACGAAAGGCCTCTGGGTAGCAGGACATGACCATGAAAATGCGGCTGCAGTC
AGGATCAGGGTTCAAGGTGGCTCCATAGTGAGTTGTGAGGGGGCAGGTCTGCCACTCCCATTCAGGAATAC
ATAGTCAGCTGCAGCCACTCCT
>DB7DT8Q1:335:H7TWNBCXX:1:2212:3323:51766/1
CTTTCAATATGCAATGGACTTCACTAGCTCAGAATGCTGGTTAACTTCTCACCCACTATAGAACAACTGCA
GGGAGAACACTTACTTCCCACCATGCATGTTTTCT
>DB7DT8Q1:335:H7TWNBCXX:1:2212:20907:52680/1
CTCAATTGAATGCAAATCAGTCTCTCCCCTGGAATACCACTAGCCACTCTAGCCCCCTAAGCCCCCATTCT
ACCTGCAAAATATATTCTATTTCCGTTTCCCAGGGAGATCCATGTATCCT
>DB7DT8Q1:335:H7TWNBCXX:1:2216:14650:2061/1
AGCAAATGAGGCCAGCCTTGTTTTTTAGCCCCTTGACTAACTTTTGGAGATTTGGCTGGGGTAGGAAAGCC
TTTAGGAACGAGGTGACTAGGTTAGGGAAATGCATTAGTGTTTGGGGGGGGAAGGAGACGGCGCCTGGGGC
AGAGCCTACCCCAAAG

>DB7DT8Q1:335:H7TWNBCXX:1:1103:20297:61075/1
CTCTTTTTGTGCTGGGGATTGCATTTAGGCCGTCTGTGCCAGGTGAGTAATGACCTCAGCCCTCTCTCACT
GGTGAACAAGACACAGTCTTATAAATTGTTAAGCGGACCCTGAACTATAAGTAGCTTAAGCAGGCTTGAAT
TTGAGATCCTCCTGCCTCCGCCTCCAGAGCAGCTGGC
>DB7DT8Q1:335:H7TWNBCXX:1:1207:16749:86059/1
GCTTCCTTCTCGCAGGCAGCATGCACATCCAGCAACTCCTGGAGAGTGTCTGTGGGAAGCCTCAGTCGCTG
GGCCATCTGCTCACTGTAGTGGTCGGCTGCCTTCTGCACAGCTAC
>DB7DT8Q1:335:H7TWNBCXX:1:1215:8006:32417/1
CGAACTCAGAAATCCACCTGCCTCTGCCTCCCAAGTGCTGGGATTAAAGGCATGCGCCAACACTGCCCGGC
AGGAGCCTCCTGTGTTAAAAGCAGGTTCAGGCTTAAG
>DB7DT8Q1:335:H7TWNBCXX:1:2105:11203:5156/1
ATTTGGTCTTGCCCCAGCCAGTGGTGGCACACAGTGTCCCAGCAGGGAAGTCATCGTCAACAGTGGGCAGA
CACACGGCAGACACAGTCTCAGAGAACTGGGCAGGAGTGGCCAGCTTCAGCAGGGTGATGTCAT
>DB7DT8Q1:335:H7TWNBCXX:1:2110:12416:88545/1
AGCCATTCCACTTGCGGATAACTGTGCCAAGTTTTGCACCATACTTGAATTCTGTCACACGGCCATTTCCA
AAGTACTCACTACCTTTAATTGCCTCACCACCCAGATCAATGACCTCTTTGAAAATGAAAGGTCTGCTTCC
TTGGGAGAACCATTTTGTATTGAGATTATGCAGTTTGTC
>DB7DT8Q1:335:H7TWNBCXX:1:2112:18739:81710/1
CCTGGCTCTGCCCCATAGAGCCACAGGACCCTGGGCACCTTTTCTACTTTCTAGGATTTTGTGATAATCTG
CTTTCAGTATATACAAAAGGGGATTAGC
>DB7DT8Q1:335:H7TWNBCXX:1:2116:6943:17667/1
GGACATTCTCTTCATCGGAGCCCTGATCAAACTCTCCAGCTACCACCACATCGGTTGTCTTGACCCCACAG
TGGGCAGCAGTGACCACCCAGTTTTCGCTGATGAGGAGCCCCCGCAGAAATGGAAGCCAGTTC
>DB7DT8Q1:335:H7TWNBCXX:1:2209:7562:29289/1
GGAATTCACAACTCAGATGAACTCAGAGAAATGTTTATTCCTTTACTGAACCAGAATCACACAAGAGGAAA
ATCATGGCCTAAGTGCCGAGCTAGTAGAGTAATGCCTTTCATATTTTTATGCAAAC
>DB7DT8Q1:335:H7TWNBCXX:1:2209:9323:85654/1
GCCGCGCTGCACGCCATCTGGCTTGATGGCAATGAAGGTACGCTCGAGGTTGGCCATGGTCCGAAAGCCGG
TGGGTCGGCTGGTGTCTGCGATGAGCGACAGAAGCAAAGAGAATAGAACGAACCAGCCGGCGCGGAGAGAG
GAG
>DB7DT8Q1:335:H7TWNBCXX:1:2215:16261:41971/1
GGCAGACACAGTCTCAGAGAACTGGGCAGAGTGGCCAGCTTCAGCAGGGTGATGTCATTACGCACGGTGAA
GGAGTTGAACTTGGGGTTCTTAAAAAC
>DB7DT8Q1:335:H7TWNBCXX:1:2216:10291:32249/1
GTAAGGGTCTATGATTAGCCTTAGAATGATACACCAGACTCAAATATTATGTAAACATAGAGTGTTTTATT
CTTCGTAAGTCCAGCATGCTGGGGTCTACCATCTACAAAGATGGAAAGACCCTGCAGTGAGCTTGCAGGCT
TGATTTAAACCACATTA
>DB7DT8Q1:335:H7TWNBCXX:1:1207:15669:24243/1
ATTTTATGGCTGGGGTTGCCACAACGTGAGGTGAGGAACTGTATTAAAGGGAGCATTAGGAAGGTTGAGAA
CCACTGCTCTAGGCCAGTGAGAGACCCTGTCTCAAAAAACACAAGTTGACTGTAACAATATCCAAG
>DB7DT8Q1:335:H7TWNBCXX:1:1212:2744:69861/1
CAGAGAGGCATGCACCGCGGGGGTGAAATCGGCAGGGTGGTGGCTAGCCAAGGTCAACAGCAGGCAGTGGC
TCAGGAGCTTGAAGTTGACGGGATCCACACGCAGCTTGTGGGCATGCAGGTCGCTCAGAGCAGACAGGGCA
CCGGGCAGG
>DB7DT8Q1:335:H7TWNBCXX:1:1214:1378:63211/1
CAAGGCCTCACGAGCTAGGGGTGCTCCATTCCTTTAACTGCTCCCCCTTTAACTGGAGTTACACATCAAGT
TCTCTGTGCACTTAGGAACTCTGTTCCTTTTCTCTCCCTGTAGTCACTATCTAACCCAATTAAACTCGACA
TTTTTAAAAACACTATCTAACCCAATTAAAC
>DB7DT8Q1:335:H7TWNBCXX:1:1215:17026:3535/1
CTCTGTGTGTAGCTGTCAAGAAGGAAGTTTCTCTTTGTTTTAGGTCTTACCTCACTGTACCATTACCCTGC
CCTGCGTCTGTGTCTGCTGGCTGTAAAGTGGTCATCATAATGCATTTCAC
>DB7DT8Q1:335:H7TWNBCXX:1:2108:18104:69250/1
GTCCACAACCAGCAGGCGGCCCTGCCTCAGGGTGTCAGGACTGTTCCCATTGGTCTGCAGCAGGCCCCAGC
CTGTGACATAGCAGACATAGTTTCTCGGGAGAATGGTGCCAGCGGGTGGGAGGCAAG
>DB7DT8Q1:335:H7TWNBCXX:1:1215:19417:67562/1
GTTTATGGCTAAGCATAGTGGGGTATCTAATCCCAGTTTGAGTCTTAGCTGTCGTGTATTATAAATGACTA
GAATTACTTTCGTTATTGAGTTTAGG
>DB7DT8Q1:335:H7TWNBCXX:1:2206:18794:59630/1

CCCACCAGTTTGAAAAAGGCTGACCATTGACCACATTCCTGAAGGCAACCATGTTCCTGATTTGACGCCAT
CTGTGTTCACAGACCCAGTCATTGCCACAAGTAGTGTCTGCATTAATGGTCACTTCTTTTGTTACTCCATT
GTTATTGGGTGG
>DB7DT8Q1:335:H7TWNBCXX:1:2207:6502:7865/1
CTCCATTGTTATTGGGTGGTCCAATCCAGTCATTCTGATCTTTTCCATTCTGGAAATTTCTATTCCAACGG
TAACTTGACATTACTCTTGTGAATCCATAAGGATGAGCCAAC
>DB7DT8Q1:335:H7TWNBCXX:1:2209:11712:14985/1
CTTGGAGGTCAGCACGGTGCTCACAGAGGCAAGGAATTTGTCCAGAGAGGCATGCACCGCGGGGGTGAAAT
CGGCAGGGTGGTGGCTAGCCAAGGTCACCAGCAGGCAGTGGCTCAGGAGCTTGAAGTTGACGGGATCCACA
CGCAGCTTGTGGGCATGCA
>DB7DT8Q1:335:H7TWNBCXX:1:2212:16975:32401/1
AAAGCTTTCCTGGTCAACACAGTTCCAAGTCAGCCAGGGCTATGTACAGAGAACCTGCCTCAAAAAACAAA
AACCAAACCAAACAAAAATTCCCTGGCCAGTAAATGGCTTAGTGGAG
>DB7DT8Q1:335:H7TWNBCXX:1:2213:1658:74093/1
CTCAAACTCACTAAGTAGCTGAGGGTGACCTTGAACTTCAATCTGCTACCTTCCAAGTAATGCGATCACAG
GTGTGCACCCACAACACTGGCAAAAGAACACGGTCTAGGATAACACAATGGCAGACAGGAAAGACAAT
>DB7DT8Q1:335:H7TWNBCXX:1:2215:18468:3051/1
GTTTGTATATGCTTGGCCCAGGGAATGGCACTATTTGAAGGCGTAGCCTTGTTGGAGCAGGTGTGTCTCTG
TGGGCTTGGGCGTGGGCTTTAATAACCTCACCCTAGCTGCC
>DB7DT8Q1:335:H7TWNBCXX:1:2215:1633:80254/1
ACACCCCCCGGGAAGCTCGCTCCCACCTTCTACCAAGGTCCAAGGAAGGGAAATGTTTCCCCTCTTCAAAT
ATCTATGGTATGTATGTCAGGCCAATTGGGCCTTGCTTTTAATTCAATCAC
>DB7DT8Q1:335:H7TWNBCXX:1:2216:10786:24283/1
CAGCATGGTCTGCCCCCATCCATTTGGGGTAGATAGGCATCCGTGAAGGGTGGAAAAACAGGTAAGAATAG
GTCTTGGCGCTCTTGGCATGGGCTTTTGCTGGGCCAGCGCTATCTCCG
>DB7DT8Q1:335:H7TWNBCXX:1:1102:11233:16192/1
AGCTCATTATTGCAACTGTAAAGTATCCTAACTGGAAAAGTATTCAGTAACATGTTTAATGCAATTTAGCA
CTAACATTATTTCTTCTAATTATTTTGTGTGCAGAAATCATATCAGCA
>DB7DT8Q1:335:H7TWNBCXX:1:1103:5824:93654/1
CTTTTATTCTGGAAATTTCTATTCCAACGGTAACTTGACATTACTCTTGTGAATCCATAGGATGAGCCAAC
ATAAATCCGACAGCCATTTTATACATTCTAGCATCCCAGAATGTCAGGATGGATGATCCTCCA
>DB7DT8Q1:335:H7TWNBCXX:1:1104:17653:53598/1
GGGATGCTTAGTTTGCAGCAATGGTGTTTTGAATCCAGTCCACATAGTTGCAGACCTTGGTGTACACACCA
GGGTTGTCCTTCAGGGCACAGCCATAGCCCCA
>DB7DT8Q1:335:H7TWNBCXX:1:1106:15096:17264/1
GGAAACTTGGGTTTACCACGTGGTTATTCCAAAGAAACTTGACTTTCTCAATTGTTCCCACATCAAGCTTG
GCATCAAACTCATTGGAATGCGTAGCACCGGGCTTGATAATTCCCCTGAAGATATCACACTGGCGAGTATT
GCCGTTACTTCCAAACAGAGACACTTTGACTTGGC
>DB7DT8Q1:335:H7TWNBCXX:1:1107:12620:59939/1
CTCACTTGGACCAGTGGCTCAACAAAATGTTACTAAAAAGCAGAACGTGAGGAGGGTGCATAGAGACATAC
AGAGAACATTTGAGATAAAAAAGAAAAGCAAGAAGGCC
>DB7DT8Q1:335:H7TWNBCXX:1:1115:15222:64472/1
CTCTGCTAAAAGCTACTTGGTTGCTGTTATTATCCCACCAGTTTGAAAAAGGCTGACCATTGACCACATTT
CTGAAGGCAACCATGTTCCTGATTTAACGCCATCTGTGTTCACAGACCCAGTCATTGCCA
>DB7DT8Q1:335:H7TWNBCXX:1:1202:7282:53560/1
CACACATGTGGTTAATGACAGCATCCACATAAATACGGACAACAACATTGTTGCACCTTGTCACCATGTCT
ATGAATTCATCTTCATTTCCAGACCTTGTGCAGATTTTATAGCTGATTGGTTGGTATCTTTCCCACCAAGG
TCTTGATGGG
>DB7DT8Q1:335:H7TWNBCXX:1:1203:7135:57555/1
CCAAGATCTCCTGAACCCAGGGCATGAGGGCAGTGACTCGGGCATACACGGCAGGGGTGGAAGTGGAGCAG
AAACCACTGCCCCAGGACACAATGCCTGCCAGGGTCCAGACTCCGTCCTTCTGGCAGACGAGGGG
>DB7DT8Q1:335:H7TWNBCXX:1:1207:7519:59831/1
AGTTGTTATACGCGTATGCCTGGAGAATTGGAATTCTTGTTACTCATACTAACAGTGTTGCATCTATAAAG
TTATAGATTAACCCAATTTTAAGTTTAGG
>DB7DT8Q1:335:H7TWNBCXX:1:2103:6487:99786/1
GTGAGAAGTACCTGGCTGCAGAGAGCCCTTGAAAACTTCATACTGTTTAGAGTTTCCTCCATTTCCAAACA
AAGAAACTAGAATGTGCCCAGTGACCTTCTGTCCAGACAGGGTGACAGTC
>DB7DT8Q1:335:H7TWNBCXX:1:2104:19021:50916/1

CAATGTCAATGAGATGGTTCAAATAGTCAGCCACCTTGGTACGAACATAATCTTTCTCAAGTGCAAGATCC
AGAAGGCCAGTCAGACGACAATTTCTGACCTGATAAGCATCATTGTAGTTATCATTTCTCCATTACATTTA
TTATCGTTAAAGTCCCAAGCAG
>DB7DT8Q1:335:H7TWNBCXX:1:2111:2706:47528/1
CTGATAGTTGCTGAGGCAATGGGCAGCTGTCAGAACCCAGTTGTTGGCCACCAGGGAGCCTCCGCAGTTGT
GGCGCCACCTTCCGGAGGAAAGGACCTGCAGGGAGACCTGCCAGGGCCAGGTGTTGGGTGTGGC
>DB7DT8Q1:335:H7TWNBCXX:1:2208:18108:28362/1
CAACAACATCCGGGTGTTTACGAACTGTTCCCCTCCAGCGGGAACCTTCAGGATCAGCTTGGAAACACAGG
CAGCCGGCAGCAAGATGTTCACAGGCACAGTGTTATGGAGTGGAACGGCTGCAGCCTCTGCCAGAAGGACA
TGGCTGGGACTGGCCTGCTGGCCATCAAGGAAGGGTTGATTAGTCTCCCTGAGGGCTCCTGCCTCTGCATT
TTCAG
>DB7DT8Q1:335:H7TWNBCXX:1:2208:10527:29738/1
CAAAACTGCTATATTTTATACTCATATGCTATATTAGCATACTCTGGATACAAACTCAAATGAAGCTTGAA
TTCTTTTTTTTTAAAGATTTATTTATTATTATATCTAAGTACACTGTAGCTG
>DB7DT8Q1:335:H7TWNBCXX:1:2212:6854:10917/1
CTGAAATCCATTTTGTGCGCAGATTAGAGCCAGGATGTTTTGACTCTGAGGGTGAACTCCTACTGGGATCT
GCTCATTTTGCAGAGGAAGTAGGGCGGACTTGTCTGTG
>DB7DT8Q1:335:H7TWNBCXX:1:2216:13799:27344/1
AAGGGTCGGGAGCGGAAAAACACGGGAACGACACGGGAACCCACGACCGGCACCGACAGGACCGACCCGCG
GAAATCGGGCAAGCCGGAGGCGCGGG
>DB7DT8Q1:335:H7TWNBCXX:1:1104:11936:91207/1
CCACTGTCACACGGTTCATGCTGGGGACCAATATTTCCCAGGAACATCTGACATTGTTAGGATAGTTTAAA
GGGTAGTGTGGGCTGGAAAATTGTCCAGAGGGTTGGGTCAGGAGACCTCCA
>DB7DT8Q1:335:H7TWNBCXX:1:1109:18934:18655/1
TGGAGGTCAGCACGGTGCTCACAGAGGCAAGGAATTTATCCAGAGAGGCATGCACCGCGGGGGTGAAATCG
GCAGGGTGGTGGCTAGCCAAGGTCACCAGCAGGCAGTGGC
>DB7DT8Q1:335:H7TWNBCXX:1:1202:16135:23720/1
GTACCGCAGTCTCAAGTTCCGCGCGATTGGATTGGAGCAGGCACTGTGATCCACTCACCAGAGGTCTTAGG
GTCCCGTGGGGAGTCCCGTGTGGACCCTTGCGGGTGTTGGGCAAGACTCTGCTGGCAAGGTAGC
>DB7DT8Q1:335:H7TWNBCXX:1:1208:6815:8228/1
AGGCTTCGTCCTGAAGATGCTAAAGTTCAGAAATCCTTCTATGCAGCTATCTAAGGATCTAAGGTCCCCAA
GAATACTCCCTTTACACGGACTAAGAGAGCCAACAAAAGGGCAATATGCA
>DB7DT8Q1:335:H7TWNBCXX:1:2111:15006:85448/1
AGTTGTTATACGCGTATGCCTGGAGAATTGGAATTCTTGTTACTCATACTAACAGTGTTGCATCTATAAAG
TTATAGATTAACCCAATTTTAAGTTTAGGAAGTTGGTGTAAATTATGGAATTAATTGAAATTTTATGTTGA
GCTTGAACGCTTTCTTTATTGGTGGC
>DB7DT8Q1:335:H7TWNBCXX:1:2111:1876:72488/1
GCCATGTAGGTGCTGGGAATTGAACACAGGACCTTTAGAAGAACAGTGAGTGATCTTAATTCCTGACCCAT
CTCTCCAGCCCCAGCTTATAAGACTTTAGAAGCCAATGGTGGCAGGCATCTGAG
>DB7DT8Q1:335:H7TWNBCXX:1:2204:20995:12794/1
CTTGGCAATATCAACCCAGCGCCACTCGAACAGGTGGACAATAGCAGTCCTCCCATCTGAAGTATGTGGGT
CATATTGAGCCCAGCAGAACCCAATGAGGGAAAGCAGCAGAACGAAC
>DB7DT8Q1:335:H7TWNBCXX:1:1105:17619:56422/1
GTACATGGAGCACAGTTCTCTTCAAGGAATATGCATACTTCAGGACATCCTGATTTTGGCAGTAACTATTC
TCATTCTTTCTTTAGGTAAACTAAGAAACAAAGGTATGTATGTTTTTGTTTGTTTGTTTGTTTGTTTTCTC
TTCTAAACAAGGAAC
>DB7DT8Q1:335:H7TWNBCXX:1:1106:9169:75216/1
CTGGTCTACACGGCACGTGCAGCACAGCCAGCTACACAGGAACACCCTGTTCCAAAAACCAACCAAGTACA
CAAATAAATCCTAACAAGGAATCAATCTTCTAATTAATCCTAAAAATTAAATACATCTTGTAGAATTTAAG
AAAACAACTTGGCATATATTTAGAATGATACAGAAAATATAAAAGGCAG
>DB7DT8Q1:335:H7TWNBCXX:1:1108:18116:88824/1
GCGATACTTGGCAGCTTCGTTGACAATCTGATCAAAGGCAGGGAAAATATAGTCGGCAAACTGGATTTCCG
CAATAGCTGTAGCACCGGTGACAGCGATTCCAATGCCAAATCCAACTATTCCTTGTTCACACAACGGGGTG
TTAAAC
>DB7DT8Q1:335:H7TWNBCXX:1:1114:2400:72542/1
CGAGGTGGCCTGCAGCATTGGCCAGAGCATCGGCGACCTTCTTGCCGTGACCCTTGACCTGGGCAGAGCCG
TGGCTTACATCAAAGTGAGGGAAGTAGGTCTTGGTGGTGGGGAAGCTAGCAAACATC
>DB7DT8Q1:335:H7TWNBCXX:1:1203:12168:100113/1

CGACTGCTCTCTTTATTTTAACTTACTTGATTTCGATTAAGAATGCCAAATGTCCTTCTAAAGCATTTAAT
ATACATAATCTTGTTTACCTGTAAAGTATAATCAAG
>DB7DT8Q1:335:H7TWNBCXX:1:1207:19837:40538/1
GGCTCGCTTGCATGTGGTGATTGTGTAGAGAATACATAGTATCTAGAAGACACTCTGAGGAACACAATAGA
GCTACAGGGTGAGTCAGGGTGGGGAAGCTTTTTGTAGCTAGCAACAAATGAACTAAG
>DB7DT8Q1:335:H7TWNBCXX:1:1214:4858:63813/1
GGGGGCCACCAGAATCACCCATACAGGAAGAGACGCCACTGGCGCCTGCGCAGATCATCACATCGGTGATC
TTGGAACCCCAGGACTACTTGCACTTAGCCTCGGAACGATGGGCAGGGCAGCCTGCTGCAGCTTGTCAGGG
GTCTTGAG
>DB7DT8Q1:335:H7TWNBCXX:1:2103:7641:25738/1
GCTGCAGCTTGTCAGGGGTCTTGAGGGCATTGTATTTGGTCTTGCCCCAGCCAGTGGTGGCACACAGTGTC
CCAGCAGGGAAGTCATCGTCAACAGTGGGCAGACACACGGCAGACACAGTCTCAGAGAACTGGGCAGGAGT
GGCC
>DB7DT8Q1:335:H7TWNBCXX:1:2113:13178:55356/1
CAAAAAACACCATAAGTATTCTATCAGTATCTATACTGTAGAAGAATGATGACTCAGTATTAAGAGTCTGA
AGTCAGTCTATCATCTTTCCTCCAAAATTTAACTGGGTTGAATTTGTTGGTTTTTTTCTACTGCCCTAAAA
ATCAGGATTAAAA
>DB7DT8Q1:335:H7TWNBCXX:1:2201:1774:81612/1
GGTCACTGGGCTGGCCAGTTTGATTAAGGCAATGTCATAGCCATTGCCGACGTTTTGGGAGTTCCACCTCT
GGTGGACCACAAGCTTAGAGACTTGAACAGCAGCAGAGCCAGCTCCGGGGTTGG
>DB7DT8Q1:335:H7TWNBCXX:1:2203:10223:2292/1
CAGTATTCTAGGAAGGAGAAGCTAAGGGATTTCTAAAGAGCGAGCAGTTTAGTTGTAGGCTTATAACTCGA
CATTTTGTACATAGTTGAGAAAATAAAATCACC
>DB7DT8Q1:335:H7TWNBCXX:1:2207:16379:96128/1
CCAGGTGCTGGGTTGGTGTATAGGGTGGACACGTCAGAGATACCAATAAACTCTGAAGTGCTTTTTCAAGA
CGGATGGAGAATTCGTAAAACCTCTTCAGCCTCACCACCAGAGGACACACTGCATTCCAAGCCTTCTCCTG
AAGTTGAATGTCATTGGGATTTTGAATTGCATCTCGGATCTCTGGTCCTGCCCCTTTGTAAGCCTG
>DB7DT8Q1:335:H7TWNBCXX:1:2115:19226:17693/1
GGTGGCCTGCAGCATTGGCCAGAGCATCGGCGACCTTCTTGCCGTGACCCTTGACCTGGGCAGAGCCGTGG
CTTACATCAAAGTGAGGGAAGTAGGTCTTGGTGGTGGGGAAGCTAGCAAACATCCTTTCCAGGGCTTCAGC
TCCATATTCAGCAC
>DB7DT8Q1:335:H7TWNBCXX:1:2214:5056:98496/1
CTTGGCAAGTTGCTGTCTAATCTTGTAATAGATGACCACATTTTCTTCATTGGGAATGACTATTTTATTAT
ACTGCTCTTCTGAGTTCTTTACCTTTTCTACGACCCCTGGAATTGCTCTATAATGCTGAAACTGGTAAAAG
>DB7DT8Q1:335:H7TWNBCXX:1:1102:9108:36241/1
CCAAACTTCTTGAAGTACTCCCTGAGCTCCGTCTCCCCGCAGTTGTGGGGAATGCCCCCGACAAAGATCTT
GTTGGATTTGCTGCTGTCGCTCCTGGGTCCTTTCTGCCAGCCTTCCTTGGGCCGTG
>DB7DT8Q1:335:H7TWNBCXX:1:1107:1376:18264/1
AAAGAGAGGGCTTATGAAGAGCTAAAGGCATTTAGTTGGCAGCGATGGTCTGCTGAATCCAGTTCACATAC
TTGCACACCTTGGTGTAGACACCAGGTTTTCCTCTCTGAGCACAGCCATAACCCCAGGAAACAACACCCTG
GAGTTGTCCATTGCAGACTACGGGNCCACCAGAGTCAC
>DB7DT8Q1:335:H7TWNBCXX:1:1116:5672:22044/1
CTGATAAGCATCATTGTAGTTATCAATTTCTCCATTACATTTATTATCGTTAAAGTCCCAAGCAGAGTATG
GAACTGCTGGGAATTCCCTGTTATTTGGATTGAGG
>DB7DT8Q1:335:H7TWNBCXX:1:1203:9555:3317/1
AGGAACAGAAAGTTGTTTCTCAATAAAGACCTAAAACAGGAATTCTCCCCAGCAGTCGGTCAGTAGCTACA
AATCCAACACTTATCTACCATACCCGCTCTTTCACACTTACTCTGGTTAGCTCTTAAAATACTGCACATTC
ACAAAGTTTATTTTATTAAACATGCCAGTTGACTGCAATATAACAGCCATCAAAAAAGCTAAAATTTTGCA
GAGCCCTGA
>DB7DT8Q1:335:H7TWNBCXX:1:1206:15336:89478/1
CAAAAGGCCAACCATGGGCAGAGAAAGAGCACGGGTGACTCCTCCCTCAGGTTTGTTATCAAGGGCATTAG
CCTCAGCCACCTCTGAGCTACTCCTCTCTCAGGCTCAATGTCC
>DB7DT8Q1:335:H7TWNBCXX:1:2102:14619:29295/1
ATCAAACCCCTCCATTCAAGGCTCAGGGATCTATATACAAGAGAAGACAGGAAGACTGTGAGAGCCAGTGA
TGGTGAACTCCAAGGAAACACTGTCTTTCAGACACAGCA
>DB7DT8Q1:335:H7TWNBCXX:1:2204:18917:93159/1
GTAATGCGTGCACCCCAGTACTGCCGACACTGATTCACAGTGACCAGGGGTAGAACAACTTGCTGCAGGCG
AGCTGGTGTCACATTGCCCACACCACTGATTCGGCCCCAGCCAGGGTGACACAGGTGAGCCCCGAAG

>DB7DT8Q1:335:H7TWNBCXX:1:2206:9573:25855/1
GTCAGCACGGTGCTCACAGAGGCAAGGAATTTGTCCAGAGAGGCATGCACCGCGGGGGTGAAATCGGCAGG
GTGGTGGCTAGCCAAGGTCACCAGCAGGCAGTG
>DB7DT8Q1:335:H7TWNBCXX:1:2214:2405:65172/1
AGGATCCACATGCAGCTTGTCACAGTGGAGCTCACTGAGGCTGGCAAAGGTGCCCTTGAGGCTGTCGAAGT
GATTCAGGCCATCGTTAAAGGCAGTTATCACTTTCTTGCCATGGGCCTTCACTTTGGCATTACCCATGATA
GCAGAGGCAGAGGATAGGTCTCCAAAGCTATCAAAGTACCGCTGGGTCCAAG
>DB7DT8Q1:335:H7TWNBCXX:1:1101:5606:19770/1
CACATACAGTGCCCCAGGAACCCTGGTAAAGGATCTCCACTCGACCCTGACACCTGTCTCCTCCGTTCACC
AGCCTCACAGCCAAACCAGAATCTGTTCCTGCAGTGGTTTGTTCAG
>DB7DT8Q1:335:H7TWNBCXX:1:1103:12741:23196/1
CATCAGAGTGACAACTATTTCCAAATCGTGTTGTAAATATGAAACACAATTCTGAAACTATTTGGCTGACT
GACATCATTGACCCTGAAACCAGGGACACATTAATCTCAGAG
>DB7DT8Q1:335:H7TWNBCXX:1:1103:18800:24509/1
TGTTAATATCTGCTGAATACAAAACTTTATGATAACCTCCAACATCTTTCTTAATAACCTTATAGGCAATA
GTTAGAGGAGAACATATGCTGCTAGGTGGCTAACGTTAGCAACCCACTTCCTTTCATCATGTTTTAAGAGA
TTATTTATAAAGAATCTTATGCTCACAAAACTAAAAATCTTTTCCTTCAAGTCAG
>DB7DT8Q1:335:H7TWNBCXX:1:1105:4863:6943/1
TGTTTATAGGTACGGACAGCCTCTCTCACAAACTGACAGAATGCAGGAGAAATCCACTCTCTTGCGTGGAA
ACCACAGTCGATGAAGAAGGCAGGCTTATTTGGTCTATCTTTGCC
>DB7DT8Q1:335:H7TWNBCXX:1:1105:9782:38029/1
GCGAGTATTGCCGTTACTTCCAAACAGAGACACTTTGACTTGGCCTGTGACCGTCCTTCCAGAGAATGTCA
AGGAAACACGGTACCTCCAGCGTGCAAAGTTCTTG
>DB7DT8Q1:335:H7TWNBCXX:1:1111:8984:61920/1
TGGAAATAGGACCATATCAATGCATGGGCATTCACTTGTATAGTCTAATTATTCCTAATTAATGGCTTATT
TGCCTAGTCATATAGAGCGTTGATACTTTCTTTGAATACTAGTGAACATTCCTACAGAGGAATGGATACAA
AAAATGTGGTACATATACTACTCAGCTATTA
>DB7DT8Q1:335:H7TWNBCXX:1:2203:5933:74077/1
GTTTCTGATACACAACACTGTTCATTTTGCTAGACTGGCTGTTTGGACTTGTTGATGTAGCCCTGAATGGA
CTAGATGTTGGCGTGGTGCTTCGACCACCAATTGGAGGTGTTCCTGGTTTGATATCAATGCCACTCCCAAA
GGCTTTTAGTTCCATTTCAGACATTTTTCCTGTGGTCGCCATCATGCTATGCTGTGTTCCAGCAGGGATTA
ATCCTCGGAAAGGCTGGCTTACTTGGGCAGGACGGCTCAAAC
>DB7DT8Q1:335:H7TWNBCXX:1:2206:9825:62638/1
GGGACACCTGATAGGGGACAGAACTCTCTCGGCAGGTGTATCCTCCAACAATATTGTCATCATCATCCACA
GGGAAAGCAACAGCAGCTCCCACAAGGGCTAGG
>DB7DT8Q1:335:H7TWNBCXX:1:1116:3520:75139/1
AGCATGGATTGCAATAAATGGGTCCTCAGCAGAGTTACTAATGGAAAAGTGAGCTTTGCCATCACTGCCAA
CATTCACTCTAAGTCCAGTGCAAATGCCATCGACCTTATCTCCAGAGATGACATCACAGTATGTGCCAGCA
GGAAGAC
>DB7DT8Q1:335:H7TWNBCXX:1:1208:13298:70473/1
GCATCAAGCGCCAAGGTCCTGAGGCAGGAGAAAGGAGACCAGAGAGAGGCTGGAAGGGTGAAGAAGTGAGA
TTGGGCTTAGCTTTGGAGGACATACAGG
>DB7DT8Q1:335:H7TWNBCXX:1:1211:19724:51543/1
GTGACAGTCCCTGAGTTTGGGGTGGGTAGGGTGAGATATAAGCATCCCATTCATAGATGAGTATTCCAATG
ACATTTATTCTCTGGATGTTGACCACTTATAACTAACTCCC
>DB7DT8Q1:335:H7TWNBCXX:1:1213:10760:16046/1
GAGACATTTCAAGGATGGCACACACCATCAAGGCCCAACAAAGCCACCATAGATTACAGGCATAGCAAGAG
GCAAAAGTTCATGAAGGGAACCCAG
>DB7DT8Q1:335:H7TWNBCXX:1:1215:19775:57604/1
GGCATGGATTGCAATAAATGGGTCCTCAGCAGAGTTACTAATGGAAAAGTGAGCTTTGCCATCACTGCCAA
CATTCACTCTAAGTCCAGTGCAATTGGCATCGACCTTATCTCCAGAGATGACATCACAGTATGTGCCAGCA
GGAAGACCAGTCTGTAAAGTGGCTGACAAAGCCCAGTCATCA
>DB7DT8Q1:335:H7TWNBCXX:1:2106:12942:32842/1
CTCATATCACAACCTTTAGAGAGGACAGATTTACAGCCTCTATTTTATCTCTGATGTCAAGGTCATCTACT
ACAAATACGGAGTAAAATGAATGAGGTTGGATTCCTAAGAGGGAGGAGGGATCCA
>DB7DT8Q1:335:H7TWNBCXX:1:2106:16085:88728/1

CTAAATGTGATATTGAGAGTGGTTTTCAAGTAAGGAGCCATGTACCAGAGCTCCATTTTCTTAGGTAAGAT
TCCTATTGCTGTGATAAAACATCATGACCAAAATCAACTTGGGGAAGAAAGGATTCATTTTCTCTTATAGC
TTGTAGTCCATCATCCCGGGAACTCCAGACAAGAACCTTGAGGCAGG
>DB7DT8Q1:335:H7TWNBCXX:1:2112:20571:56170/1
CTGGGCAGAGGTACAAAGGCCACTCTGGCATTGAGGGTCACAGGGGAAGCCAGTTTGATCAGCATGATGTC
ATTGTCCAGGGTCCATGAATTATAATTGGGGTGCCG
>DB7DT8Q1:335:H7TWNBCXX:1:2116:20891:47463/1
TCCAGACCATCACAGGCAGGTTGTGGGACACCTGCTTCCTGCCCTGGGGCACCCAGATATTGAGGTAGAGG
CAGTCTTCTTGCCCATAGGTGTTGTCCTGGGTGATGG
>DB7DT8Q1:335:H7TWNBCXX:1:2203:16071:19222/1
GCAAGATCTGCTTGGCTGGCAGGAGGAAGCCATAGTAGCCTGTGTCTCTCAGTTCAAAGGCAAATGAGTAT
TTGATGCCAAGGTCATAAGCCCAGTCGATGCTTCCGCCACTGGCCTGGTAGATGACAGAACAGATGGGTCC
CAC
>DB7DT8Q1:335:H7TWNBCXX:1:2216:21491:40767/1
TTTCTCTCTGCTCCTGAATCCCGGCTGCACCTGTGCCCCATGCTTCACGTGTCACTCTTGGAGCACATGCC
ACCTCCATGATGTTCCTCCGAATGCTAGCACACTCG
>DB7DT8Q1:335:H7TWNBCXX:1:1202:17348:14589/1
GCTGTGTCCACTTGCACGGGTGTGGCTGTCAAACTGGGATTCCAGAATATGTTTCACGTTGCTTATCAATA
CCTCATAGAGAACTTCATTCTCCTCGAGAAAGTTCTCCACATCA
>DB7DT8Q1:335:H7TWNBCXX:1:1212:5992:45650/1
GCAGGTGCAAGGGAGAGAAGAAGGGCATGGCCAGAAGGCAAGCCCCGCAGAAGGCAGCTTAACGGTACTTG
GAGGTCAGCACGGTGCTCACAGAGGCAAGGAATTTGTCCAGAGAGGCATGCACCGCGGGGGTGAAATCGGC
AGGGTGGTGGCTAGCCAAGGTCACCAGCAGGCAGTGGCTCAGGAGCTTGAAG
>DB7DT8Q1:335:H7TWNBCXX:1:1212:3927:48205/1
AGCAGAGTTACTAATGGAAAAGTGAGCTTTGCCATCACTGCCAACATTCACTCTAAGTCAAGTGCAATTGC
CATCGACCTTATCTCCAGAGATGACATCACAGTATGTGCCAGCAGGAAGACCAGTCTGTAAAGTGGC
>DB7DT8Q1:335:H7TWNBCXX:1:2209:12361:62880/1
CCCACCAGTTTGAAAAAGGCTGACCATTGACCACATTCCTGAAGGCAACCATGTTCCTGATTTGACGCCAT
CTGTGTTCACAGACCCAGTCATTGCCACAAGTAGTGTCTGCATTAATGGTCACTTCTTTTGTTACTCCATT
GTTATTGGGTGG
>DB7DT8Q1:335:H7TWNBCXX:1:1215:13385:39154/1
AATGGCTCCAAAGGTCCTCTTTCCAGCCTCCCCAGCAATGTGGGAACCCAGGCTGTGCCAATCAGGTGGAC
ATTGTTAAGTGAGTATCCCAAATCAGACTGCAGAACGTTAACCAACAATGCCACTTCTGCCCCCACGACCC
GGAC
>DB7DT8Q1:335:H7TWNBCXX:1:2101:7941:45494/1
CTGAGGACGAGCACTGCCACTGGAGTCCCTCCTCTAGCCAATCAAGAGAACTGACAACCAAGTCTTCCAAA
GAGTCCAGCCGCAGTGAACTCAGAGACTTCTGCACCGTGG
>DB7DT8Q1:335:H7TWNBCXX:1:2106:5061:79448/1
AGCTGAGTTACTAATGGAAAAGTGAGCTTTGCCATCACTGCCAACATTCACTCTAAGTCCAGTGCAATTGC
CATCGACCTTATCTCCAGAGATGACATCACAGTATGTGCCAGCAGGAAGACCAG
>DB7DT8Q1:335:H7TWNBCXX:1:2107:7400:90207/1
GGGATCCACACGCAGCTTGTGGGCATGCAGGTCGCTCAGAGCTGACAGGGCACCGGGCAGGTCATCGAGGT
GGCCTGCAGCATTGGCCAGAGCATCGGCGACCTTCTTGCCGTGACCCTTGACCTGGGCAGAGCCGTGGCTT
ACATCAAAGTGAGGGAAG
>DB7DT8Q1:335:H7TWNBCXX:1:2114:12779:65047/1
CACAGTATGTACCAGCAGGAAGACCAGTCTGTAAAGTGGCTGACAAAGCCCAGTCATCATTGTTAAAGACA
ATGAATCCTCTGTTTCCTCTGCTAAAAGCTACTTGGTTGCTGTTATTATCCCACCAGTTTGAAAAAGGCTG
ACCATTGACCACATTTCTG
>DB7DT8Q1:335:H7TWNBCXX:1:2211:9248:6652/1
GTACCGCAGTCTCAAGTTCCGCGCGATTGGATTGGAGCAGGCACTGTGATCCACTCACCAGAGGTCTTAGG
GTCCCGTGGGGAGTCCCGTGTGGACCCTTGCGGGTGTTGGGCAAGACTCTGCTGGCAAGGTAGC
>DB7DT8Q1:335:H7TWNBCXX:1:2104:9594:68454/1
GGGGTCTACTCCACCGAACAACAATGGAAGTATCATCAACCTGGTCCACGGTAGGGTCTGGAGGAGCGTCA
GGTGCTGTAGTCTGTGAGGTAGACAGGATCAAGCTCTGTTTTCCCTCCTCAGATATCTGATAGACATTGAC
>DB7DT8Q1:335:H7TWNBCXX:1:2214:18227:9305/1
CAGCAGAGTTACTAATGGAAAAGTGAGCTTTGCCATCACTGCCAACATTCACTCTAAGTCCAGTGCAATTG
CCATCGACCTTATCTCCAGAGATGACATCACAGTATGTGCCAGCAGGAAGACCAG
>DB7DT8Q1:335:H7TWNBCXX:1:2216:20510:14842/1

CCACAATACCTCTCCTGGGCATATATCCAGAAGATGTTCCAACTGGTAAGAAAGGCACATGCTCCACTATG
TTCATAGCAGGCTTATTTATAATAGCCAGAAGCTGGAAAGAACCCAGATGCCCCACGACAGAGGAATGGAT
ACAGAAAATGTGGTACAATTAATGGTATACTACTCAGCT
>DB7DT8Q1:335:H7TWNBCXX:1:1105:11313:70389/1
CCCCAATTCCCTCTTAAAAACACTAAAACCCAAGTAATAACAGCAATCAAACTAGCCAGGAATGGCGCTAC
AGATCAGTAATCCCTGTACTCTGAAGGTGGAGACTGGAGGACGGGAAACTCAAG

Cairns (Mouse/rat)
TTTCCTCCCCAAGCTACTCTTGGTTATAGTGCTTCATCATAGCAATAGTAACCCCATCTAAGATAACACCT
CTAATACTTTCAACAGAGTTTCTGTTTCTGTCCTCAGACACACCAGAAGAGGGCATAAGATCCCATTACAG
ATGGCTGTGAGACACCATGCGGTTGCTGGGAATTGAACTTTGGACCTCTGG

Mouse DNA sequence from clone RP23-134I10 on chromosome 17, complete sequence
Sequence ID: CT009607.7
Identities: 192/193(99%) Gaps: 0/193(0%)

```
Query  1      TTTCCTCCCCAAGCTACTCTTGGTTATAGTGCTTCATCATAGCAATAGTAACCCCATCTA  60
              ||||||||||||||||||||||||||||||||||||||||||||||||||||||||||||
Sbjct  51815  TTTCCTCCCCAAGCTACTCTTGGTTATAGTGCTTCATCATAGCAATAGTAACCCCATCTA  51756

Query  61     AGATAACACCTCTAATACTTTCAACAGAGTTTCTGTTTCTGTCCTCAGACACACCAGAAG  120
              ||||||||||||||||||||||||||||||||||||||||||||||||||||||||||||
Sbjct  51755  AGATAACACCTCTAATACTTTCAACAGAGTTTCTGTTTCTGTCCTCAGACACACCAGAAG  51696

Query  121    AGGGCATAAGATCCCATTACAGATGGCTGTGAGACACCATGCGGTTGCTGGGAATTGAAC  180
              |||||||||| |||||||||||||||||||||||||||||||||||||||||||||||||
Sbjct  51695  AGGGCATAAGGTCCCATTACAGATGGCTGTGAGACACCATGCGGTTGCTGGGAATTGAAC  51636

Query  181    TTTGGACCTCTGG  193
              |||||||||||||
Sbjct  51635  TTTGGACCTCTGG  51623
```

Homo sapiens chromosome 20, alternate assembly CHM1_1.1
Sequence ID: NC_018931.2
Identities: 24/24(100%) Gaps: 0/24(0%)

```
Query  83        AACAGAGTTTCTGTTTCTGTCCTC  106
                 ||||||||||||||||||||||||
Sbjct  56417389  AACAGAGTTTCTGTTTCTGTCCTC  56417412
```

TCTGTAGACCAGGCTGGCTTCAAAGATCTGCCTTCCTCTGTCTCCTAAGTGCTGGGATTAAAGGTTTACAC
TACCACCACTAGCAATTAAGATACAAATTATAAGACTTAAGATTTTAGGTAGATGTAATTATACTTATAAT
TTTTATTTT

Mus musculus BAC clone RP23-106G24 from chromosome 3, complete sequence
Sequence ID: AC163297.4
Identities: 151/151(100%) Gaps: 0/151(0%)

```
Query  1      TCTGTAGACCAGGCTGGCTTCAAAGATCTGCCTTCCTCTGTCTCCTAAGTGCTGGGATTA  60
              ||||||||||||||||||||||||||||||||||||||||||||||||||||||||||||
Sbjct  30148  TCTGTAGACCAGGCTGGCTTCAAAGATCTGCCTTCCTCTGTCTCCTAAGTGCTGGGATTA  30089

Query  61     AAGGTTTACACTACCACCACTAGCAATTAAGATACAAATTATAAGACTTAAGATTTTAGG  120
              ||||||||||||||||||||||||||||||||||||||||||||||||||||||||||||
Sbjct  30088  AAGGTTTACACTACCACCACTAGCAATTAAGATACAAATTATAAGACTTAAGATTTTAGG  30029

Query  121    TAGATGTAATTATACTTATAATTTTTATTTT  151
              |||||||||||||||||||||||||||||||
Sbjct  30028  TAGATGTAATTATACTTATAATTTTTATTTT  29998
```

PREDICTED: Homo sapiens centrosomal protein 63 (CEP63), transcript variant X31, mRNA
Sequence ID: XM_017007267.1
Identities: 39/49(80%) Gaps: 0/49(0%)

```
Query  44    cctaagtgctgggattaaaggTTTACACTACCACCACTAGCAATTAAGA  92
             || ||||||||| |||| | || |   ||||||||||| ||| ||||||
Sbjct  1741  CCAAAGTGCTGGAATTACAAGTGTGAGCTACCACCACTGGCAGTTAAGA  1789
```

CTAGAAAGACATTTTACCTTGTTTCAATATGAAAGCAATGGAATAAACCATGTACAAACTAAAAAAGAACT
GAGCCAGAACATACACAAGGTCCCAGGTTCTATCCCCAGCACCACAAAAATCAGTAAGTACAGATTTGAAA
GACGGTGTAGTTCCAGAGCTTGACAGAGTTGGTAAAGAATTCCT

Mouse DNA sequence from clone RP23-240F13 on chromosome X, complete sequence
Sequence ID: AL844583.6
Identities: 185/186(99%) Gaps: 0/186(0%)

```
Query  1     CTAGAAAGACATTTTACCTTGTTTCAATATGAAAGCAATGGAATAAACCATGTACAAACT  60
             ||||||||||||||||||||||||||||||||||||||||||||||||||||||||||||
Sbjct  2178  CTAGAAAGACATTTTACCTTGTTTCAATATGAAAGCAATGGAATAAACCATGTACAAACT  2119

Query  61    AAAAAAGAACTGAGCCAGAACATACACAAGGTCCCAGGTTCTATCCCCAGCACCACAAAA  120
             ||||||||||||||||||||||||||||||||||||||||||||||||||||||||||||
Sbjct  2118  AAAAAAGAACTGAGCCAGAACATACACAAGGTCCCAGGTTCTATCCCCAGCACCACAAAA  2059

Query  121   ATCAGTAAGTACAGATTTGAAAGACGGTGTAGTTCCAGAGCTTGACAGAGTTGGTAAAGA  180
             ||||||||||||||||||||||||| ||||||||||||||||||||||||||||||||||
Sbjct  2058  ATCAGTAAGTACAGATTTGAAAGACAGTGTAGTTCCAGAGCTTGACAGAGTTGGTAAAGA  1999

Query  181   ATTCCT  186
             ||||||
Sbjct  1998  ATTCCT  1993
```

Homo sapiens chromosome 2, alternate assembly CHM1_1.1
Sequence ID: NC_018913.2
Identities: 25/26(96%) Gaps: 0/26(0%)

```
Query  61        AAAAAAGAACTGAGCCAGAACATACA  86
                 ||||||||||||||||||| ||||||
Sbjct  32235984  AAAAAAGAACTGAGCCAGACCATACA  32235959
```

AAGAGAAAGACCTTGGTCTTATGACGATCATATGCCCCAGTACAGGGGAATGCCAGGGCCAGGAAGCAGGA
GTGGGTGGGTTGGGGAGCAGGGCGGGGGGAGGGTATAGGGAGCTTTGGGCATAGCATTTGAAATGTAAATG
AAG

Mus musculus 6 BAC RP24-194N1 (Roswell Park Cancer Institute (C57BL/6J Male) Mouse BAC Library) complete sequence
Sequence ID: AC155727.6
Identities: 145/145(100%) Gaps: 0/145(0%)

```
Query  1       AAGAGAAAGACCTTGGTCTTATGACGATCATATGCCCCAGTAcaggggaatgccagggcc  60
               ||||||||||||||||||||||||||||||||||||||||||||||||||||||||||||
Sbjct  164283  AAGAGAAAGACCTTGGTCTTATGACGATCATATGCCCCAGTACAGGGGAATGCCAGGGCC  164342

Query  61      aggaagcaggagtgggtgggttggggagcagggcggggggagggTATAGGGAGCTTTGGG  120
               ||||||||||||||||||||||||||||||||||||||||||||||||||||||||||||
Sbjct  164343  AGGAAGCAGGAGTGGGTGGGTTGGGGAGCAGGGCGGGGGGAGGGTATAGGGAGCTTTGGG  164402

Query  121     CATAGCATTTGAAATGTAAATGAAG  145
               |||||||||||||||||||||||||
Sbjct  164403  CATAGCATTTGAAATGTAAATGAAG  164427
```

Homo sapiens chromosome 7, alternate assembly CHM1_1.1
Sequence ID: NC_018918.2
Identities: 25/28(89%) Gaps: 0/28(0%)

```
Query  116        TTGGGCATAGCATTTGAAATGTAAATGA  143
                  |||||||||  ||||||||||||| |||
Sbjct  133050342  TTGGGCATATTATTTGAAATGTAACTGA  133050369
```

ACCATGAGGTGCTGAGAAGAAGGTATATCCTTTTGTTTTAGGATAAAATGTTCTGTAGATATCTGTCAGAT
CCATTTGTTTCATCACTTCTGTTAGTTTCACTGTGTCCCTGTTTAGTTTCTGTTTCAATGATCTGTCCATT
GGTGAAAGTGGTGTG

Mus musculus immunoglobulin kappa chain complex (Igk) on chromosome 6
Sequence ID: NG_005612.1
Identities: 156/157(99%) Gaps: 0/157(0%)

```
Query  1      ACCATGAGGTGCTGAGAAGAAGGTATATCCTTTTGTTTTAGGATAAAATGTTCTGTAGAT  60
              ||||||||||||||||||||||||||||||||||||||||||||||||||||||||||||
Sbjct  34862  ACCATGAGGTGCTGAGAAGAAGGTATATCCTTTTGTTTTAGGATAAAATGTTCTGTAGAT  34921

Query  61     ATCTGTCAGATCCATTTGTTTCATCACTTCTGTTAGTTTCACTGTGTCCCTGTTTAGTTT  120
              ||||||||||||||||||||||||||||||||||||||||||||||||||||||||||||
Sbjct  34922  ATCTGTCAGATCCATTTGTTTCATCACTTCTGTTAGTTTCACTGTGTCCCTGTTTAGTTT  34981

Query  121    CTGTTTCAATGATCTGTCCATTGGTGAAAGTGGTGTG  157
```

```
                    ||||||| |||||||||||||||||||||||||||||
Sbjct  34982  CTGTTTCCATGATCTGTCCATTGGTGAAAGTGGTGTG  35018
```

No match for *Homo Sapiens*

CCTGAACTTTGAATGCCAAGAAAAGTAATTTCTTTCCAATGAATAAGTCAGTATCAATGTCAAAATCACTG
GTCCCATCAGGTTCACTGCCCATTCTGCAATTTGAAGCCATTCTCATAGAGGAATACACTGAGGATGTTTG

Mus musculus, clone RP23-367H12, complete sequence
Sequence ID: AC117668.9
Identities: 142/142(100%) Gaps:0/142(0%)

```
Query  1       CCTGAACTTTGAATGCCAAGAAAAGTAATTTCTTTCCAATGAATAAGTCAGTATCAATGT  60
               ||||||||||||||||||||||||||||||||||||||||||||||||||||||||||||
Sbjct  173441  CCTGAACTTTGAATGCCAAGAAAAGTAATTTCTTTCCAATGAATAAGTCAGTATCAATGT  173382

Query  61      CAAAATCACTGGTCCCATCAGGTTCACTGCCCATTCTGCAATTTGAAGCCATTCTCATAG  120
               ||||||||||||||||||||||||||||||||||||||||||||||||||||||||||||
Sbjct  173381  CAAAATCACTGGTCCCATCAGGTTCACTGCCCATTCTGCAATTTGAAGCCATTCTCATAG  173322

Query  121     AGGAATACACTGAGGATGTTTG  142
               ||||||||||||||||||||||
Sbjct  173321  AGGAATACACTGAGGATGTTTG  173300
```

Homo sapiens chromosome 13, alternate assembly CHM1_1.1
Sequence ID: NC_018924.2
Identities: 52/64(81%) Gaps: 0/64(0%)

```
Query  3         TGAACTTTGAATGCCAAGAAAAGTAATTTCTTTCCAATGAATAAGTCAGTATCAATGTCA  62
                 ||| | ||| ||| |||| ||||| |||| ||||||||||| ||||||||  |||   |||
Sbjct  59369862  TGATCCTTGCATGACAAGGAAAGTCATTTATTTCCAATGAACAAGTCAGTCACAAAATCA  59369803

Query  63        AAAT  66
                 ||||
Sbjct  59369802  AAAT  59369799
```

AGAGAGACAAGAAAGTGATATGGGCAAAGAATCCAAAGATAGTTCTGAACAATCCCCTGGAGCTCTGGACT
GTACATACTTCAGCAGTAGTCTACTTTCTCATCTGCATAAAGACTGCTTGCTCTGCCCTGGATCAAGACTA
CTACAAAGATCAGAGGGCTGGAGAGATGGTTCAGCAG

Mus musculus BAC clone RP24-107H20 from chromosome 17, complete sequence
Sequence ID: AC183093.2
Identities: 179/179(100%) Gaps: 0/179(0%)

```
Query  1       AGAGAGACAAGAAAGTGATATGGGCAAAGAATCCAAAGATAGTTCTGAACAATCCCCTGG  60
               ||||||||||||||||||||||||||||||||||||||||||||||||||||||||||||
Sbjct  134954  AGAGAGACAAGAAAGTGATATGGGCAAAGAATCCAAAGATAGTTCTGAACAATCCCCTGG  135013

Query  61      AGCTCTGGACTGTACATACTTCAGCAGTAGTCTACTTTCTCATCTGCATAAAGACTGCTT  120
               ||||||||||||||||||||||||||||||||||||||||||||||||||||||||||||
Sbjct  135014  AGCTCTGGACTGTACATACTTCAGCAGTAGTCTACTTTCTCATCTGCATAAAGACTGCTT  135073

Query  121     GCTCTGCCCTGGATCAAGACTACTACAAAGATCAGAGGGCTGGAGAGATGGTTCAGCAG  179
               |||||||||||||||||||||||||||||||||||||||||||||||||||||||||||
Sbjct  135074  GCTCTGCCCTGGATCAAGACTACTACAAAGATCAGAGGGCTGGAGAGATGGTTCAGCAG  135132
```

Homo sapiens chromosome 1 genomic patch of type NOVEL, GRCh38.p7 PATCHES HSCHR1_4_CTG3
Sequence ID: NW_014040926.1
Identities: 29/33(88%) Gaps: 1/33(3%)

```
Query  80      TTCAGCAGTAGTCTACTTTCTCATCTGCATAAA  112
               |||| || ||||||||||| || ||||||||||
Sbjct  181480  TTCACCA-TAGTCTACTTTTTCTTCTGCATAAA  181449
```

CTGTGTTCATTAAGAGATGATGTAACTCTTTAGCAGTGTTCTCCTGTATTTCTTTAAGTGATTTATTAAAG
TCCTTCTTGATGTCCTCTACCATCATCATGAGATATGCTTTTAAATCTAGGTCTAGGTTCTCAGGTGTGTT
GGGGTTCCCTGGACTGGGCGAAGTGGGTG

Mus musculus BAC clone RP23-241M6 from 16, complete sequence
Sequence ID: AC122817.3
Identities: 171/171(100%) Gaps: 0/171(0%)

```
Query  1       CTGTGTTCATTAAGAGATGATGTAACTCTTTAGCAGTGTTCTCCTGTATTTCTTTAAGTG  60
               ||||||||||||||||||||||||||||||||||||||||||||||||||||||||||||
```

```
Sbjct  118365  CTGTGTTCATTAAGAGATGATGTAACTCTTTAGCAGTGTTCTCCTGTATTTCTTTAAGTG  118306

Query  61      ATTTATTAAAGTCCTTCTTGATGTCCTCTACCATCATCATGAGATATGCTTTTAAATCTA  120
               ||||||||||||||||||||||||||||||||||||||||||||||||||||||||||||
Sbjct  118305  ATTTATTAAAGTCCTTCTTGATGTCCTCTACCATCATCATGAGATATGCTTTTAAATCTA  118246

Query  121     GGTCTAGGTTCTCAGGTGTGTTGGGGTTCCCTGGACTGGGCGAAGTGGGTG  171
               |||||||||||||||||||||||||||||||||||||||||||||||||||
Sbjct  118245  GGTCTAGGTTCTCAGGTGTGTTGGGGTTCCCTGGACTGGGCGAAGTGGGTG  118195
```

Homo sapiens programmed cell death 4 (neoplastic transformation inhibitor) (PDCD4), transcript variant 3, mRNA

Identities: 30/35(86%) Gaps: 0/35(0%)

```
Query  60    GATTTATTAAAGTCCTTCTTGATGTCCTCTACCAT  94
             ||||||||||||||| | | || ||| ||||||||
Sbjct  1397  GATTTATTAAAGTCCCTTTGGAAGTCTTCTACCAT  1431
```

CTTTCATAGTCTCTGGTGAAAAGTCTGGTATAATTCTGATAGGCCTGCCTTTATATGTTACTTGACCCTTT
TCCCTTACTACTTTTAATATTCTATCTTTATTTAGTGCATTAGCTGCTCTGATTATTATGTGTTGGGAGGA
ACTTCTTTTCTGGTCCAGTATATTTGGATTTCTGTA

Mus musculus BAC clone RP24-238D1 from chromosome 13, complete sequence

Sequence ID: AC155297.2

Identities: 175/176(99%) Gaps:0/176(0%)

```
Query  1      CTTTCATAGTCTCTGGTGAAAAGTCTGGTATAATTCTGATAGGCCTGCCTTTATATGTTA  60
              |||||| |||||||||||||||||||||||||||||||||||||||||||||||||||||
Sbjct  36942  CTTTCAAAGTCTCTGGTGAAAAGTCTGGTATAATTCTGATAGGCCTGCCTTTATATGTTA  36883

Query  61     CTTGACCCTTTTCCCTTACTACTTTTAATATTCTATCTTTATTTAGTGCATTAGCTGCTC  120
              ||||||||||||||||||||||||||||||||||||||||||||||||||||||||||||
Sbjct  36882  CTTGACCCTTTTCCCTTACTACTTTTAATATTCTATCTTTATTTAGTGCATTAGCTGCTC  36823

Query  121    TGATTATTATGTGTTGGGAGGAACTTCTTTTCTGGTCCAGTATATTTGGATTTCTG  176
              ||||||||||||||||||||||||||||||||||||||||||||||||||||||||
Sbjct  36822  TGATTATTATGTGTTGGGAGGAACTTCTTTTCTGGTCCAGTATATTTGGATTTCTG  36767
```

Homo sapiens chromosome 6, alternate assembly CHM1_1.1

Sequence ID: NC_018917.2

Identities: 28/31(90%) Gaps: 0/31(0%)

```
Query  2          TTTCATAGTCTCTGGtgaaaagtctggtata  32
                  |||||||||||||||||| ||||| | ||||
Sbjct  128154102  TTTCATAGTCTCTGGTGAGAAGTCAGCTATA  128154072
```

AGCTTGCAAGGAGAAGGAGAAGAGCCCTTAGGGAGCTGCTTTCATCTCAGAGCAACCCAGTTTCTGTTTTA
AAAAAAAAACAGTGAGCTCACTGTGTGCTCCAGAGCAATAATCAACTTAGCCTGGCTGGCTTGAAGCAGAG
TGCTGTGTGGCGAGCTGCTCAGGAGTGTCT

Mus musculus BAC clone RP23-205D14 from chromosome 12, complete sequence

Sequence ID: AC153147.7

Identities: 172/172(100%) Gaps: 0/172(0%)

```
Query  1      AGCTTGCAAGGAGAAGGAGAAGAGCCCTTAGGGAGCTGCTTTCATCTCAGAGCAACCCAG  60
              ||||||||||||||||||||||||||||||||||||||||||||||||||||||||||||
Sbjct  13381  AGCTTGCAAGGAGAAGGAGAAGAGCCCTTAGGGAGCTGCTTTCATCTCAGAGCAACCCAG  13440

Query  61     TTTCTGTTTTaaaaaaaaaaCAGTGAGCTCACTGTGTGCTCCAGAGCAATAATCAACTTA  120
              ||||||||||||||||||||||||||||||||||||||||||||||||||||||||||||
Sbjct  13441  TTTCTGTTTTAAAAAAAAAACAGTGAGCTCACTGTGTGCTCCAGAGCAATAATCAACTTA  13500

Query  121    GCCTGGCTGGCTTGAAGCAGAGTGCTGTGTGGCGAGCTGCTCAGGAGTGTCT  172
              ||||||||||||||||||||||||||||||||||||||||||||||||||||
Sbjct  13501  GCCTGGCTGGCTTGAAGCAGAGTGCTGTGTGGCGAGCTGCTCAGGAGTGTCT  13552
```

Homo sapiens chromosome 14, alternate assembly CHM1_1.1

Sequence ID: NC_018925.2

Identities: 93/119(78%) Gaps: 8/119(6%)

```
Query  11        GAGAAGGAGAAGAGCCCTTAGGGAGCTGCT--TTCATCTCAGAGCAACCCAGTTTCTGTT  68
                 ||| |||||  ||||||| |||||||  ||  ||||||||| |||| ||   ||||||||
Sbjct  77436498  GAGGAGGAG--GAGCCCT-AGGGAGCCCCTCTTTCATCTCAAAGCAGCCAGATTTCTGTT  77436554

Query  69        TTaaaaaaaaaaCAGTGAGCTCACTGTGTGCTCCAGAGCAATAATCAACTTAGCCTGGC  127
                 ||   ||||   ||||||||||||||||  ||| ||||||||||| |||||   |||||
```

Sbjct 77436555 TTTGCAAAA---CAGTGAGCTCACTGTGCACTCTAGAGCAATAATAAACTTTCTCTGGC 77436610

>DB7DT8Q1:306:HBCKVADXX:2:2116:15075:44621/1
AGTGCATGCATGCATGCCTGTATGTGTGTAAACATGCATTTGTATCCATGTGTAAACTGGTGTACAAACTC
CTGTGACATCCTCAGAAACAGCTTCTTTGGGATACTTTCTCACTGGCTTCATGCTCACCAACTAGACTAGG
CTGGCACACCAGACAGTCCCAAGAGTCTGTCTGCTTCTGCCTCCCTGGTGTTGGGAAGACAGGCAT

>DB7DT8Q1:306:HBCKVADXX:2:2116:15075:44621/1
AGTGCATGCATGCATGCCTGTATGTGTGTAAACATGCATTTGTATCCATGTGTAAACTGGTGTACAAACTC
CTGTGACATCCTCAGAAACAGCTTCTTTGGGATACTTTCTCACTGGCTTCATGCTCACCAACTAGACTAGG
CTGGCACACCAGACAGTCCCAAGAGTCTGTCTGCTTCTGCCTCCCTGGTGTTGGGAAGACAGGCAT
>DB7DT8Q1:306:HBCKVADXX:2:2116:10488:45536/1
TCTCTACTTGAAAATTACTCTGAGATTTTAAACACACACACACACACTTCTTTGTTCAGTGAAGGGGACTA
GTGGCCATAGTTAGTAGATTGCTCATCACAAATAATGAATGTAAATTGAGTGTGTGAATCAGACCCTATAA
ATGTTGCTGTTATAAATTACTCATCTAAAGTCAAGTACTTACAAGTGATATTGAATTTTGGTCTCTT
>DB7DT8Q1:306:HBCKVADXX:2:2116:7811:46408/1
AGTCCTTCTTCCCAAATGACTCCAGCTTGTATCAAGCAGACATAAAACTAGCCAGGACGGGCACCCAGAAG
AGCGTTAAATGAACTTAGGAAAGCAACTCTGACAAACACCCATGCCCAAGGTTAGTAAAAGTCGAAGATAA
TACAGGAAACAGAAGACATGATTGTTTACGAAGTAATATCATCACCAGACAGTGGGGC
>DB7DT8Q1:306:HBCKVADXX:2:2116:8878:46281/1
AGTGGGGAACTGGTCACAGAGGTCGAAGATATCTAGGCAGCGGCCCTCTGGCATGCCAGAGCCCTGACCTC
GTTTCCTGGGGTCTGAAGTCACTGTGGTCGCACAAGCATAGCTGGCTTTTGCGGGCGGTGGAGGGGGAG
>DB7DT8Q1:306:HBCKVADXX:2:2116:9123:47075/1
ATGTTGTTTAATGTGAGAAAAGAATTTACATGTTTGTCATTACCTAAGCTGACGTGTTTATATTACATTTA
TTTTAATCTCCTTCCCTAATTCTCTCATTTAAGTTTTGTCTAGAAAAGAATAAAATATTACATT
>DB7DT8Q1:306:HBCKVADXX:2:2116:10968:55966/1
ATCCTATCAGACCACCATGGCCTAAGACTGATCTTCAATAACAACATAAATAATGGAAAGCCAACATTCAC
GTGGAAACTGAATAACACTCTTCTCAATGATACCTTGGTCAAGGAAGGAATAAAGAAAGAAATTAAAGACT
TTTTAGAGTTTAATAAAAATGAAGCCACAACGTACCCAAAC
>DB7DT8Q1:306:HBCKVADXX:2:2116:5362:57947/1
TTGCCTCAGTTGAGCAGCAGAGGAAAACACTGGCTAGCATTTAGAACACCAAAGCAGAAAGGCTTATCTCT
GTATGATCAGACACTGCTGTGATCTGACCAGGGAAGGGCCTGTTCATACTCCATCTCTCTCTCTCTCTCAC
ACACACACACATATGCACATACACGTACA
>DB7DT8Q1:306:HBCKVADXX:2:2115:11662:33025/1
CAACCTTTTTACTTTCTTCATGGTATCCTTTGAAAACCAAAAAGCTTAACTTTTTTTTATTATTTTTGTGT
GTACACACACAATTTTTCAGTATTCTTTGGCAACTTCATGCATGTATATATTTTAGTCATTCTCACCCTGA
ATTAGCCTCAGCC
>DB7DT8Q1:306:HBCKVADXX:2:2115:12055:94240/1
CACAGTGCTGCTAATCAAGCAGACTCTGAAATAATATATAAAGATTGAGGCCTAATATTTATAAATCATAT
AGTTACAGATGTAACTATATCTTAATTGAAGGCTTTCTCTCCAAAGAACAGAAACTTAAGTATAGATGTCA
TGAAGAGGAAGCTGAGCCAACTATAGTGGTGCCATCTGTCATCTC
>DB7DT8Q1:306:HBCKVADXX:2:2116:14412:51883/1
TATTGATTATATGTAAGTACACTGTAGCTGTCTTCAGACACTCCAGAAGAGGGCATCAGATTTCATTAGGG
ATGGTTGTGAGCCACCATGTGGTTGCCGGGACCTGAACTCTGGACCTTCGGAAGAGCAATCGGGTGCTCT
>DB7DT8Q1:306:HBCKVADXX:2:2102:2979:92183/1
CACTGTCAGACACAATAAGACACTTGAGTCTTTCTATGTTCCAACAACCATATGTAACGTAATACAAGCTA
TTGCTACATGTCTGCTGTTCCTGACACTAACTCCTTCATGAAACAAGGTAAAAA
>DB7DT8Q1:306:HBCKVADXX:2:2115:10056:90544/1
ATGTGGTATTTAAATTGAGAACATATTAATTAAAAAAAGATATTTTAGTTTTTAATGGACAGCAATAGTGT
TGCAAATATGTACAAAGTCTTATAAGTCAACTTAAAAGGACAAACAACCTTAAAGAGAGAAAGCTATCAGA
TCAAAGGCATGGCTGTTGCTTATGTTCTAGTTCAGTG
>DB7DT8Q1:306:HBCKVADXX:2:2116:13350:47175/1
GTGTCTCATGAATAAAGCTCTTGTCAAACATCATGAGGATCTGTGTCCAAACTTCCAGAACCCATGTAAAA
GCTGGGCAAGGTAACGCACATCTGTAATCCCAGTGTGCACAGTGAGATGGAAGGCAGAGGCAGGAGCATTC
CAGGAAGCTCACAGACAAGATAGCCTGTCATGCACAGCATTGAAAAACAAGGGACCCTGATCT
>DB7DT8Q1:306:HBCKVADXX:2:2116:8926:47425/1

GGATATTCATGTTCCTTATGTTAACAAAGTGTTTAACAATCTAAAGACAGCGAAACCTGGGAGAACTGAGG
CAAAATATGAGGTGGACTAGAGTCCCTTGCAATAGCCCAGCTTACTCAGAGCATCTGACAATTTATACTTT
GAGAGTTAAGAAGAGTCACATGAGTAAAGTGTACAAACACAAGGGCATGTTGCATGTGGCAGACC
>DB7DT8Q1:306:HBCKVADXX:2:2116:10813:48095/1
TCAGAAGCCACTCCATTTGCATCCATATTTCTGGAAAATGGGTATCAGAAAGTGCTACCTTCTGACCTTCC
AAACAGAGTTCATTGATGGAATTTTTAATGAAAGTATACTTTTTACAATGTCATAATTCGGTGTTGTGGTT
TTTCTCTAAGAATGGCAACCCAGCTCAAAC
>DB7DT8Q1:306:HBCKVADXX:2:2116:10141:49694/1
GGACTGAAGGAAATGAAGGAGCTTGCAGCTACATAGGAAGAACAACAATATGAACCAACCAGTTCCCCTAG
AGTTCCCAGGGACTAAACCACTAACCAAAGAGAACCCATGGCTCCAGCTGCATATGTAGCAGAAAATGACC
TTGTTGGA
>DB7DT8Q1:306:HBCKVADXX:2:2116:8771:50099/1
CCATTTTTTCCTTTCATAAACTCCAGAGAAATGGGGGGAAAGTTACTGCCTCAAATTCAAAGAGGGGATAA
CCAATCCTTTGGACATCTACTTCTGTTTGCCAATGTCTCAGAGTCTGATCTTGGTTCTACTCTACTGTGAC
AATGAACAGGTTGGATTGTCAGTGTCCTTTGTTCTTCCAGCAAAAGCCTCCTGCGAATGTGA
>DB7DT8Q1:306:HBCKVADXX:2:2116:14714:5501/1
TATACAAAGAAGATCCACTTAGATAATCACAACATTTGTTTTATATGATAATACATGAAAATATACTATTA
AGAACAAATTAAGTAAATGCACGAGTCCTTTATTCTTGGAATGCTATATTGTTACAGTATTTTTAAAAAAA
AAAAAAAAAAAAAAACCAAAAGAGCACAAGC
>DB7DT8Q1:306:HBCKVADXX:2:2116:6379:32912/1
TGTACACACTGTAATTTACCAAAACCTGGGATATGCATCACATCTATCTGTCAAACATCTAGGGGTGGCAA
TCTGCGAGGATTAACATCCACGGAAGGTGCATTAATAAATTCTACACATTTACCACACTGTAGGACAATAT
CTTGAGCTTCTGTTCATGTGAGCTTAAA
>DB7DT8Q1:306:HBCKVADXX:2:2116:9666:33682/1
AGAGATTTCAAATTTAACTACTCAAGCAGAAGACATCTATTTTTACAACATATTTTCTTTATTTTAAGTTA
TTTCTTTTACTTTCTTCCTTCCTGACCATTTATTATATGCAATTGTTTATATTGGGGGTTGTGAAATATTT
CTTAGGCTAAAATACAAGATTATTTTTGATAATCTTT
>DB7DT8Q1:306:HBCKVADXX:2:2116:8154:33978/1
CCACTACATAATGAGGCCATGTCTCAAAAACACCAATCAATCAATCTTCCCCAGGCAGTGATGGCACATGC
CTTTAATCTCAGCATTTGGGAGGCAGAGGCAGGCGGATTTCTGAGTTTGAAGCCAGCCTGGTCTACAGAGT
GAGTTCCAGGAC
>DB7DT8Q1:306:HBCKVADXX:2:2116:17010:34698/1
GTGGTGGCACAAGCCTTTAGTTCCAGCACTCAGGAGGCAGAGGCAGGTGGATTGCTGTGAGTCAGAAGCCA
ACTTTGGTCTCTAAAGCAAGCACCAGGCCAGCCAGGGTTACATACTGAAACCCTATCTTAAAAAATAAGGG
CAA
>DB7DT8Q1:306:HBCKVADXX:2:2116:15143:35039/1
TAATCAGGTCTGACATGCACAAAAATCTCTAGTAAATCTCTAATATAATTTTATCTCAGGATATTTTTAAA
AAATCAAGTCATAAGGCAAGCATAATCAACTATGAGATCATATATACAGAAATACAGGATACTTTTAGCAG
GAGTCATTTCTGCTATTCTGTTACTGCAGGCTTATT
>DB7DT8Q1:306:HBCKVADXX:2:2116:7833:35733/1
ATTCTGCATATTTACTTATTTTCCAAAATAAATTAAAGAAAGCTATTCACATCTCTTTCCATTGGTTCTTT
AATTACAATAACAATTTGATCTTCAAGACTTCTGAAACATTTCATAAAAGCAAAAAACTTCCTTGTTTCAA
ATAGCATATGCAAGTTAAAATGAAAATTTTATTTCACCTTAATTATTTTTCT
>DB7DT8Q1:306:HBCKVADXX:2:2208:1797:34895/1
AATGTGAATGAAAACAATGGGGAGAGGAAGTGTGGAGGATTGGAATCTCCGCAGAGGCCCCGCAGGTCACT
AGGGACCAATGAGGTTCCTTTCTGCTCTCGGTGGTGGCCGGAAGTGGGAAAGCGGGATCATCTGTTCC
>DB7DT8Q1:306:HBCKVADXX:2:2213:1979:37978/1
CATTCACCATTAGCGCGTTTGTTTCTAAATAAAACAACACTAAACTGTGGAAAGATACCAAGGAGAGAGGT
TTCTGTGATGAAGCTTCTGAAATGTTTAAGAAGTATGACATGGTAGATTATAGGGAGGCTTGAAAAGAGTA
CTGACTTGAAAAATATTTG
>DB7DT8Q1:306:HBCKVADXX:2:2116:5004:40492/1
CAATCTCATGTCATGGGTCTCAGCCTGAGCCACTTTTGGGAGAAAATTAATATTTATATTCAGAAGAAGAA
ATGTTGGTATGTCTTACCATAACTATCAATATCCTGTGAGCTATTTTTCTTTTCTGTCTGATTTAAAAAAA
AAGTTAATTGTTACTGCTTAAGACTTGTGA
>DB7DT8Q1:306:HBCKVADXX:2:2116:11100:40539/1
TTATTTCAGATTCATTCTCTTTGCTTCCTTTGTGCTACCTGGATGTCATCTGCCAGCTTCCTGCTCTGCCC
ACTTGCTACCGCGACTCCCCAGTCATTGTGATATAGAT
>DB7DT8Q1:306:HBCKVADXX:2:2116:14778:40927/1

TGTTTTGTGTATTTTGTTGTTTGAGACAAACTCTCATGACTGTGTATTTCTGGCTGGCCTAGAACTTGCTA
TATGCACCAGGCTGACATCAAACTCACAGAGATCTGAATCTGCCTGACTCTGATTCCTGGGTGCTTAAAAG
CATGACAATAATGTGTTTTTATAATAGCTGTCAG
>DB7DT8Q1:306:HBCKVADXX:2:2116:18046:40969/1
CAATTTGTGTCAGATTCATATATATATGAGACAAAAAAAAAATCTACCACTAAATAGGGATTCATTGTATG
GTAGTGTTGTTAAAACGTGATATCCTAACATCGTAATGTGTTAGCTTATACTCTATCACTAGTTACGATTT
TAGTAAAATTGTATGTTGTAGCTTCATTATCATTTTCTGATCCACTACAG
>DB7DT8Q1:306:HBCKVADXX:2:2116:13582:41205/1
TACGTTTCTGGTTTGTTTGTTTTTTTTTAATCTCTTTTATGTTAGCAATATATAAAAATCATATGTGAAAT
TCACTCAAAAAAATGTCAGTAGATTGACTTTACCTCTAGAACATGGCAAATAACTTATAAAGCATATATTC
TCAATGCCCCCCCTTGATAAAATGCAATGTCAGCAGAAGTTAGCTCTGATATACTCTACTAGC
>DB7DT8Q1:306:HBCKVADXX:2:2116:5461:42578/1
AGGTAAGACCTTTTCAATGGATGCTCCAACTTGTTGCAGTTGTAGAAGTGTACAGGTATGCAGTCAAAGAG
GTACATGAACTGGATCATCCAAGTCTTTTCAGGTCTCTGGATATTAAGGG
>DB7DT8Q1:306:HBCKVADXX:2:2116:6792:42591/1
GCCATCGTGTAGCTAGGAAACTGAAGGCAATACATAAGAAGGAAAAGAATGGAGGCTAGAGAGACGGCTTA
GCAATAGAGGGTACTTGCCACTCACACAGAGGACCTGAGTTCAGTCCCCAGCACTCGCAGGGTGGCACACA
AGTCCACTGTAGTTCAAG
>DB7DT8Q1:306:HBCKVADXX:2:2116:18225:43064/1
TAACAATTTAAGAGAATGTCTCTACTAGCTTCTTCTTGTTGTTATTGTAACCATGTAATATGCTTTACTAT
AGGCTTAATAGCTTAAAAAACAAGAGTTTTAGCTTCTTTGGTTCTGCATTGAGAAGGTCTATATACATATC
TTTTAAAATGTGAGGAATTTGTGTGTCTTTCTAGAACAG
>DB7DT8Q1:306:HBCKVADXX:2:2115:3895:43895/1
AACCTAGGAAGTTAGCCACCCCTCAAGTTTATTTCTGGAGACTTCTTTGAGACATTAACATTTTGTGGATC
TGTGGTTGTGTGTGTGTGTGTGTGGGGGCAGTACTTCTTTGAAGGTAGTTGAGGGTGGAGGGAAAGGGGAG
AAGGGATGGAGAGCTAGGGGTGTAAGCGGTACAGAGATCTCTTTTAAAA
>DB7DT8Q1:306:HBCKVADXX:2:2116:12700:29417/1
ACACATGTCGAGTTCTACAACAGTCAGGGCTGCACAGAATGCTACAAACAAACAAACATATTATTATTTTT
TACTTTGTATTTTCTTGGCATATATTATCGTATATAAATTATAAACTTTCAAGATAGCATGATAGCTTTAC
ACGAAGCATTCATTATCTATGGTCTTGATGCTTAGTCAAAGACTAATCACTGTATTTG
>DB7DT8Q1:306:HBCKVADXX:2:2116:9882:30486/1
TACAAGCTGCATCCAGCTGTGATTCTTCTGCAGCCACCTTTTTATAAAGACAAAAAAAAGAGGGGACTTTT
GCGGTTATATGTCTGTCCTTACACAGAGAGCAAACCTGTCTTCTAGTAGACAGGATGGTGATCACTCAGGC
TGAGAAGAAGGGCTGATGACTGGCCAGGACTCCTTTTAAAACTACTTTGCTGGGGCTGGGGAGAC
>DB7DT8Q1:306:HBCKVADXX:2:2116:5814:31553/1
ATGCCATGTACTGAGTGGTGTTGTGAGTGAGTCCTCTGATGATGGACCATGCCATGTAGCAAGCGCTGCTT
CCTTCCACCCACCACTCTCCTGGAAGCACGTTTGCATGTTTGGATTTACAGCTTACCTTGTCTTAACTCCA
TGTCGATAGATTCACCGTTAGATACTTGTTTCCCAAAGAGACTGTAGTAGAGTTCCA
>DB7DT8Q1:306:HBCKVADXX:2:2116:9569:31776/1
TATATAATTGCTTCAAGTCTATTTCTGAAGTGTCATGATGAAATTATCCCCTGTGAACAAAACAACACAAA
TGAAAAGAAACAATTGTCTTCTGTTTGCAGGCAACACTAGACAGAAGGTTTGTTTCATGAAGAATAAAAGC
CAGTGATTTTTGTCTAAATTTTTGTTATGAAAAGGA
>DB7DT8Q1:306:HBCKVADXX:2:2116:14094:32747/1
AACAAAGAATTCTCACCTGAGGAATACCGAATGGCAGAGAAGCACTTGAAAAAATGTTCAACATCCTTAAT
CATCAGGGAAATGCAAATCAAAACAACCCTGAGATTCCACCTCACACCAGTCAGAATGGCTAAGATCAAAA
ATTCAGGTGACAGCAGATGCTGGCGAGGATG
>DB7DT8Q1:306:HBCKVADXX:2:2116:15474:36484/1
TTTCCTCCCCAAGCTACTCTTGGTTATAGTGCTTCATCATAGCAATAGTAACCCCATCTAAGATAACACCT
CTAATACTTTCAACAGAGTTTCTGTTTCTGTCCTCAGACACACCAGAAGAGGGCATAAGATCCCATTACAG
ATGGCTGTGAGACACCATGCGGTTGCTGGGAATTGAACTTTGGACCTCTGG
>DB7DT8Q1:306:HBCKVADXX:2:2116:8986:36868/1
TCTGTAGACCAGGCTGGCTTCAAAGATCTGCCTTCCTCTGTCTCCTAAGTGCTGGGATTAAAGGTTTACAC
TACCACCACTAGCAATTAAGATACAAATTATAAGACTTAAGATTTTAGGTAGATGTAATTATACTTATAAT
TTTTATTTT
>DB7DT8Q1:306:HBCKVADXX:2:2116:5866:37370/1
CTAGAAAGACATTTTACCTTGTTTCAATATGAAAGCAATGGAATAAACCATGTACAAACTAAAAAAGAACT
GAGCCAGAACATACACAAGGTCCCAGGTTCTATCCCCAGCACCACAAAAATCAGTAAGTACAGATTTGAAA
GACGGTGTAGTTCCAGAGCTTGACAGAGTTGGTAAAGAATTCCT

>DB7DT8Q1:306:HBCKVADXX:2:2116:7248:37537/1
AAGAGAAAGACCTTGGTCTTATGACGATCATATGCCCCAGTACAGGGGAATGCCAGGGCCAGGAAGCAGGA
GTGGGTGGGTTGGGGAGCAGGGCGGGGGGAGGGTATAGGGAGCTTTGGGCATAGCATTTGAAATGTAAATG
AAG
>DB7DT8Q1:306:HBCKVADXX:2:2116:3568:38118/1
ACCATGAGGTGCTGAGAAGAAGGTATATCCTTTTGTTTTAGGATAAAATGTTCTGTAGATATCTGTCAGAT
CCATTTGTTTCATCACTTCTGTTAGTTTCACTGTGTCCCTGTTTAGTTTCTGTTTCAATGATCTGTCCATT
GGTGAAAGTGGTGTG
>DB7DT8Q1:306:HBCKVADXX:2:2116:9169:39421/1
CCTGAACTTTGAATGCCAAGAAAAGTAATTTCTTTCCAATGAATAAGTCAGTATCAATGTCAAAATCACTG
GTCCCATCAGGTTCACTGCCCATTCTGCAATTTGAAGCCATTCTCATAGAGGAATACACTGAGGATGTTTG
>DB7DT8Q1:306:HBCKVADXX:2:2116:17522:39492/1
AGAGAGACAAGAAAGTGATATGGGCAAAGAATCCAAAGATAGTTCTGAACAATCCCCTGGAGCTCTGGACT
GTACATACTTCAGCAGTAGTCTACTTTCTCATCTGCATAAAGACTGCTTGCTCTGCCCTGGATCAAGACTA
CTACAAAGATCAGAGGGCTGGAGAGATGGTTCAGCAG
>DB7DT8Q1:306:HBCKVADXX:2:2116:7569:39565/1
CTGTGTTCATTAAGAGATGATGTAACTCTTTAGCAGTGTTCTCCTGTATTTCTTTAAGTGATTTATTAAAG
TCCTTCTTGATGTCCTCTACCATCATCATGAGATATGCTTTTAAATCTAGGTCTAGGTTCTCAGGTGTGTT
GGGGTTCCCTGGACTGGGCGAAGTGGGTG
>DB7DT8Q1:306:HBCKVADXX:2:2116:7643:39972/1
CTTTCATAGTCTCTGGTGAAAAGTCTGGTATAATTCTGATAGGCCTGCCTTTATATGTTACTTGACCCTTT
TCCCTTACTACTTTTAATATTCTATCTTTATTTAGTGCATTAGCTGCTCTGATTATTATGTGTTGGGAGGA
ACTTCTTTTCTGGTCCAGTATATTTGGATTTCTGTA
>DB7DT8Q1:306:HBCKVADXX:2:2111:14986:11709/1
AGCTTGCAAGGAGAAGGAGAAGAGCCCTTAGGGAGCTGCTTTCATCTCAGAGCAACCCAGTTTCTGTTTTA
AAAAAAAAACAGTGAGCTCACTGTGTGCTCCAGAGCAATAATCAACTTAGCCTGGCTGGCTTGAAGCAGAG
TGCTGTGTGGCGAGCTGCTCAGGAGTGTCT
>DB7DT8Q1:306:HBCKVADXX:2:2116:15075:44621/1
AGTGCATGCATGCATGCCTGTATGTGTGTAAACATGCATTTGTATCCATGTGTAAACTGGTGTACAAACTC
CTGTGACATCCTCAGAAACAGCTTCTTTGGGATACTTTCTCACTGGCTTCATGCTCACCAACTAGACTAGG
CTGGCACACCAGACAGTCCCAAGAGTCTGTCTGCTTCTGCCTCCCTGGTGTTGGGAAGACAGGCAT
>DB7DT8Q1:306:HBCKVADXX:2:2116:10488:45536/1
TCTCTACTTGAAAATTACTCTGAGATTTTAAACACACACACACACACTTCTTTGTTCAGTGAAGGGGACTA
GTGGCCATAGTTAGTAGATTGCTCATCACAAATAATGAATGTAAATTGAGTGTGTGAATCAGACCCTATAA
ATGTTGCTGTTATAAATTACTCATCTAAAGTCAAGTACTTACAAGTGATATTGAATTTTGGTCTCTT
>DB7DT8Q1:306:HBCKVADXX:2:2116:7811:46408/1
AGTCCTTCTTCCCAAATGACTCCAGCTTGTATCAAGCAGACATAAAACTAGCCAGGACGGGCACCCAGAAG
AGCGTTAAATGAACTTAGGAAAGCAACTCTGACAAACACCCATGCCCAAGGTTAGTAAAAGTCGAAGATAA
TACAGGAAACAGAAGACATGATTGTTTACGAAGTAATATCATCACCAGACAGTGGGGC
>DB7DT8Q1:306:HBCKVADXX:2:2116:8878:46281/1
AGTGGGGAACTGGTCACAGAGGTCGAAGATATCTAGGCAGCGGCCCTCTGGCATGCCAGAGCCCTGACCTC
GTTTCCTGGGGTCTGAAGTCACTGTGGTCGCACAAGCATAGCTGGCTTTTGCGGGCGGTGGAGGGGGAG
>DB7DT8Q1:306:HBCKVADXX:2:2116:9123:47075/1
ATGTTGTTTAATGTGAGAAAAGAATTTACATGTTTGTCATTACCTAAGCTGACGTGTTTATATTACATTTA
TTTTAATCTCCTTCCCTAATTCTCTCATTTAAGTTTTGTCTAGAAAAGAATAAAATATTACATT
>DB7DT8Q1:306:HBCKVADXX:2:2115:11662:33025/1
CAACCTTTTTACTTTCTTCATGGTATCCTTTGAAAACCAAAAAGCTTAACTTTTTTTTATTATTTTTGTGT
GTACACACACACAATTTTTCAGTATTCTTTGGCAACTTCATGCATGTATATATTTTAGTCATTCTCACCCTGA
ATTAGCCTCAGCC
>DB7DT8Q1:306:HBCKVADXX:2:2115:12055:94240/1
CACAGTGCTGCTAATCAAGCAGACTCTGAAATAATATATAAAGATTGAGGCCTAATATTTATAAATCATAT
AGTTACAGATGTAACTATATCTTAATTGAAGGCTTTCTCTCCAAAGAACAGAAACTTAAGTATAGATGTCA
TGAAGAGGAAGCTGAGCCAACTATAGTGGTGCCATCTGTCATCTC
>DB7DT8Q1:306:HBCKVADXX:2:2116:14412:51883/1
TATTGATTATATGTAAGTACACTGTAGCTGTCTTCAGACACTCCAGAAGAGGGCATCAGATTTCATTAGGG
ATGGTTGTGAGCCACCATGTGGTTGCCGGGACCTGAACTCTGGACCTTCGGAAGAGCAATCGGGTGCTCT
>DB7DT8Q1:306:HBCKVADXX:2:2102:2979:92183/1

CACTGTCAGACACAATAAGACACTTGAGTCTTTCTATGTTCCAACAACCATATGTAACGTAATACAAGCTA
TTGCTACATGTCTGCTGTTCCTGACACTAACTCCTTCATGAAACAAGGTAAAAA
>DB7DT8Q1:306:HBCKVADXX:2:2116:10968:55966/1
ATCCTATCAGACCACCATGGCCTAAGACTGATCTTCAATAACAACATAAATAATGGAAAGCCAACATTCAC
GTGGAAACTGAATAACACTCTTCTCAATGATACCTTGGTCAAGGAAGGAATAAAGAAAGAAATTAAAGACT
TTTTAGAGTTTAATAAAAATGAAGCCACAACGTACCCAAAC
>DB7DT8Q1:306:HBCKVADXX:2:2116:5362:57947/1
TTGCCTCAGTTGAGCAGCAGAGGAAAACACTGGCTAGCATTTAGAACACCAAAGCAGAAAGGCTTATCTCT
GTATGATCAGACACTGCTGTGATCTGACCAGGGAAGGGCCTGTTCATACTCCATCTCTCTCTCTCTCTCAC
ACACACACACATATGCACATACACGTACA
>DB7DT8Q1:306:HBCKVADXX:2:2115:10056:90544/1
ATGTGGTATTTAAATTGAGAACATATTAATTAAAAAAAGATATTTTAGTTTTTAATGGACAGCAATAGTGT
TGCAAATATGTACAAAGTCTTATAAGTCAACTTAAAAGGACAAACAACCTTAAAGAGAGAAAGCTATCAGA
TCAAAGGCATGGCTGTTGCTTATGTTCTAGTTCAGTG
>DB7DT8Q1:306:HBCKVADXX:2:2116:13350:47175/1
GTGTCTCATGAATAAAGCTCTTGTCAAACATCATGAGGATCTGTGTCCAAACTTCCAGAACCCATGTAAAA
GCTGGGCAAGGTAACGCACATCTGTAATCCCAGTGTGCACAGTGAGATGGAAGGCAGAGGCAGGAGCATTC
CAGGAAGCTCACAGACAAGATAGCCTGTCATGCACAGCATTGAAAAACAAGGGACCCTGATCT
>DB7DT8Q1:306:HBCKVADXX:2:2116:8926:47425/1
GGATATTCATGTTCCTTATGTTAACAAAGTGTTTAACAATCTAAAGACAGCGAAACCTGGGAGAACTGAGG
CAAAATATGAGGTGGACTAGAGTCCCTTGCAATAGCCCAGCTTACTCAGAGCATCTGACAATTTATACTTT
GAGAGTTAAGAAGAGTCACATGAGTAAAGTGTACAAACACAAGGGCATGTTGCATGTGGCAGACC
>DB7DT8Q1:306:HBCKVADXX:2:2116:10813:48095/1
TCAGAAGCCACTCCATTTGCATCCATATTTCTGGAAAATGGGTATCAGAAAGTGCTACCTTCTGACCTTCC
AAACAGAGTTCATTGATGGAATTTTTAATGAAAGTATACTTTTTACAATGTCATAATTCGGTGTTGTGGTT
TTTCTCTAAGAATGGCAACCCAGCTCAAAC
>DB7DT8Q1:306:HBCKVADXX:2:2116:10141:49694/1
GGACTGAAGGAAATGAAGGAGCTTGCAGCTACATAGGAAGAACAACAATATGAACCAACCAGTTCCCCTAG
AGTTCCCAGGGACTAAACCACTAACCAAAGAGAACCCATGGCTCCAGCTGCATATGTAGCAGAAAATGACC
TTGTTGGA
>DB7DT8Q1:306:HBCKVADXX:2:2116:8771:50099/1
CCATTTTTTCCTTTCATAAACTCCAGAGAAATGGGGGGAAAGTTACTGCCTCAAATTCAAAGAGGGGATAA
CCAATCCTTTGGACATCTACTTCTGTTTGCCAATGTCTCAGAGTCTGATCTTGGTTCTACTCTACTGTGAC
AATGAACAGGTTGGATTGTCAGTGTCCTTTGTTCTTCCAGCAAAAGCCTCCTGCGAATGTGA
>DB7DT8Q1:306:HBCKVADXX:2:2116:5004:40492/1
CAATCTCATGTCATGGGTCTCAGCCTGAGCCACTTTTGGGAGAAAATTAATATTTATATTCAGAAGAAGAA
ATGTTGGTATGTCTTACCATAACTATCAATATCCTGTGAGCTATTTTTCTTTTCTGTCTGATTTAAAAAAA
AAGTTAATTGTTACTGCTTAAGACTTGTGA
>DB7DT8Q1:306:HBCKVADXX:2:2116:11100:40539/1
TTATTTCAGATTCATTCTCTTTGCTTCCTTTGTGCTACCTGGATGTCATCTGCCAGCTTCCTGCTCTGCCC
ACTTGCTACCGCGACTCCCCAGTCATTGTGATATAGAT
>DB7DT8Q1:306:HBCKVADXX:2:2116:14778:40927/1
TGTTTTGTGTATTTTGTTGTTTGAGACAAACTCTCATGACTGTGTATTTCTGGCTGGCCTAGAACTTGCTA
TATGCACCAGGCTGACATCAAACTCACAGAGATCTGAATCTGCCTGACTCTGATTCCTGGGTGCTTAAAAG
CATGACAATAATGTGTTTTTATAATAGCTGTCAG
>DB7DT8Q1:306:HBCKVADXX:2:2116:14714:5501/1
TATACAAAGAAGATCCACTTAGATAATCACAACATTTGTTTTATATGATAATACATGAAAATATACTATTA
AGAACAAATTAAGTAAATGCACGAGTCCTTTATTCTTGGAATGCTATATTGTTACAGTATTTTTAAAAAAA
AAAAAAAAAAAAAAAACCAAAAGAGCACAAGC
>DB7DT8Q1:306:HBCKVADXX:2:2116:6379:32912/1
TGTACACACTGTAATTTACCAAAACCTGGGATATGCATCACATCTATCTGTCAAACATCTAGGGGTGGCAA
TCTGCGAGGATTAACATCCACGGAAGGTGCATTAATAAATTCTACACATTTACCACACTGTAGGACAATAT
CTTGAGCTTCTGTTCATGTGAGCTTAAA
>DB7DT8Q1:306:HBCKVADXX:2:2116:9666:33682/1
AGAGATTTCAAATTTAACTACTCAAGCAGAAGACATCTATTTTTACAACATATTTTCTTTATTTTAAGTTA
TTTCTTTTACTTTCTTCCTTCCTGACCATTTATTATATGCAATTGTTTATATTGGGGGTTGTGAAATATTT
CTTAGGCTAAAATACAAGATTATTTTTGATAATCTTT
>DB7DT8Q1:306:HBCKVADXX:2:2116:8154:33978/1

CCACTACATAATGAGGCCATGTCTCAAAAACACCAATCAATCAATCTTCCCCAGGCAGTGATGGCACATGC
CTTTAATCTCAGCATTTGGGAGGCAGAGGCAGGCGGATTTCTGAGTTTGAAGCCAGCCTGGTCTACAGAGT
GAGTTCCAGGAC
>DB7DT8Q1:306:HBCKVADXX:2:2116:17010:34698/1
GTGGTGGCACAAGCCTTTAGTTCCAGCACTCAGGAGGCAGAGGCAGGTGGATTGCTGTGAGTCAGAAGCCA
ACTTTGGTCTCTAAAGCAAGCACCAGGCCAGCCAGGGTTACATACTGAAACCCTATCTTAAAAAATAAGGG
CAA
>DB7DT8Q1:306:HBCKVADXX:2:2116:15143:35039/1
TAATCAGGTCTGACATGCACAAAAATCTCTAGTAAATCTCTAATATAATTTTATCTCAGGATATTTTTAAA
AAATCAAGTCATAAGGCAAGCATAATCAACTATGAGATCATATATACAGAAATACAGGATACTTTTAGCAG
GAGTCATTTCTGCTATTCTGTTACTGCAGGCTTATT
>DB7DT8Q1:306:HBCKVADXX:2:2116:7833:35733/1
ATTCTGCATATTTACTTATTTTCCAAAATAAATTAAAGAAAGCTATTCACATCTCTTTCCATTGGTTCTTT
AATTACAATAACAATTTGATCTTCAAGACTTCTGAAACATTTCATAAAAGCAAAAAACTTCCTTGTTTCAA
ATAGCATATGCAAGTTAAAATGAAAATTTTATTTCACCTTAATTATTTTTCT
>DB7DT8Q1:306:HBCKVADXX:2:2208:1797:34895/1
AATGTGAATGAAAACAATGGGGAGAGGAAGTGTGGAGGATTGGAATCTCCGCAGAGGCCCCGCAGGTCACT
AGGGACCAATGAGGTTCCTTTCTGCTCTCGGTGGTGGCCGGAAGTGGGAAAGCGGGATCATCTGTTCC
>DB7DT8Q1:306:HBCKVADXX:2:2213:1979:37978/1
CATTCACCATTAGCGCGTTTGTTTCTAAATAAAACAACACTAAACTGTGGAAAGATACCAAGGAGAGAGGT
TTCTGTGATGAAGCTTCTGAAATGTTTAAGAAGTATGACATGGTAGATTATAGGGAGGCTTGAAAAGAGTA
CTGACTTGAAAAATATTTG
>DB7DT8Q1:306:HBCKVADXX:2:2116:18046:40969/1
CAATTTGTGTCAGATTCATATATATATGAGACAAAAAAAAAATCTACCACTAAATAGGGATTCATTGTATG
GTAGTGTTGTTAAAACGTGATATCCTAACATCGTAATGTGTTAGCTTATACTCTATCACTAGTTACGATTT
TAGTAAAATTGTATGTTGTAGCTTCATTATCATTTTCTGATCCACTACAG
>DB7DT8Q1:306:HBCKVADXX:2:2116:13582:41205/1
TACGTTTCTGGTTTGTTTGTTTTTTTTTAATCTCTTTTATGTTAGCAATATATAAAAATCATATGTGAAAT
TCACTCAAAAAAATGTCAGTAGATTGACTTTACCTCTAGAACATGGCAAATAACTTATAAAGCATATATTC
TCAATGCCCCCCCTTGATAAAATGCAATGTCAGCAGAAGTTAGCTCTGATATACTCTACTAGC
>DB7DT8Q1:306:HBCKVADXX:2:2116:5461:42578/1
AGGTAAGACCTTTTCAATGGATGCTCCAACTTGTTGCAGTTGTAGAAGTGTACAGGTATGCAGTCAAAGAG
GTACATGAACTGGATCATCCAAGTCTTTTCAGGTCTCTGGATATTAAGGG
>DB7DT8Q1:306:HBCKVADXX:2:2116:6792:42591/1
GCCATCGTGTAGCTAGGAAACTGAAGGCAATACATAAGAAGGAAAAGAATGGAGGCTAGAGAGACGGCTTA
GCAATAGAGGGTACTTGCCACTCACACAGAGGACCTGAGTTCAGTCCCCAGCACTCGCAGGGTGGCACACA
AGTCCACTGTAGTTCAAG
>DB7DT8Q1:306:HBCKVADXX:2:2116:18225:43064/1
TAACAATTTAAGAGAATGTCTCTACTAGCTTCTTCTTGTTGTTATTGTAACCATGTAATATGCTTTACTAT
AGGCTTAATAGCTTAAAAAACAAGAGTTTTAGCTTCTTTGGTTCTGCATTGAGAAGGTCTATATACATATC
TTTTAAAATGTGAGGAATTTGTGTGTCTTTCTAGAACAG
>DB7DT8Q1:306:HBCKVADXX:2:2115:3895:43895/1
AACCTAGGAAGTTAGCCACCCCTCAAGTTTATTTCTGGAGACTTCTTTGAGACATTAACATTTTGTGGATC
TGTGGTTGTGTGTGTGTGTGTGTGGGGGCAGTACTTCTTTGAAGGTAGTTGAGGGTGGAGGGAAAGGGGAG
AAGGGATGGAGAGCTAGGGGTGTAAGCGGTACAGAGATCTCTTTTAAAA
>DB7DT8Q1:306:HBCKVADXX:2:2116:12700:29417/1
ACACATGTCGAGTTCTACAACAGTCAGGGCTGCACAGAATGCTACAAACAAACAAACATATTATTATTTTT
TACTTTGTATTTTCTTGGCATATATTATCGTATATAAATTATAAACTTTCAAGATAGCATGATAGCTTTAC
ACGAAGCATTCATTATCTATGGTCTTGATGCTTAGTCAAAGACTAATCACTGTATTTG
>DB7DT8Q1:306:HBCKVADXX:2:2116:9882:30486/1
TACAAGCTGCATCCAGCTGTGATTCTTCTGCAGCCACCTTTTTATAAAGACAAAAAAAAGAGGGGACTTTT
GCGGTTATATGTCTGTCCTTACACAGAGAGCAAACCTGTCTTCTAGTAGACAGGATGGTGATCACTCAGGC
TGAGAAGAAGGGCTGATGACTGGCCAGGACTCCTTTTAAAACTACTTTGCTGGGGCTGGGGAGAC
>DB7DT8Q1:306:HBCKVADXX:2:2116:5814:31553/1
ATGCCATGTACTGAGTGGTGTTGTGAGTGAGTCCTCTGATGATGGACCATGCCATGTAGCAAGCGCTGCTT
CCTTCCACCCACCACTCTCCTGGAAGCACGTTTGCATGTTTGGATTTACAGCTTACCTTGTCTTAACTCCA
TGTCGATAGATTCACCGTTAGATACTTGTTTCCCAAAGAGACTGTAGTAGAGTTCCA
>DB7DT8Q1:306:HBCKVADXX:2:2116:9569:31776/1

TATATAATTGCTTCAAGTCTATTTCTGAAGTGTCATGATGAAATTATCCCCTGTGAACAAAACAACACAAA
TGAAAAGAAACAATTGTCTTCTGTTTGCAGGCAACACTAGACAGAAGGTTTGTTTCATGAAGAATAAAAGC
CAGTGATTTTTGTCTAAATTTTTGTTATGAAAAGGA
>DB7DT8Q1:306:HBCKVADXX:2:2116:14094:32747/1
AACAAAGAATTCTCACCTGAGGAATACCGAATGGCAGAGAAGCACTTGAAAAAATGTTCAACATCCTTAAT
CATCAGGGAAATGCAAATCAAAACAACCCTGAGATTCCACCTCACACCAGTCAGAATGGCTAAGATCAAAA
ATTCAGGTGACAGCAGATGCTGGCGAGGATG
>DB7DT8Q1:306:HBCKVADXX:2:2112:6442:4866_1:N:0:GGCTACAT/1
ATCTGGCATTCTAAATTAATTCAACATATATAAGAAAGATCTTAACAATTAGCCAAGAAGACTTATACTCT
AAAGAATTCATTCAACCATAAAGTATGATTAATTTCACCATTGTTTTGACACATGGCAGTTCAAGTTCAAC
CTTGTTAC
>DB7DT8Q1:306:HBCKVADXX:2:2112:7581:13704_1:N:0:GTCTACAT/1
CTGACTCGCTAAATACCCTTGGCTGGTTGCTGCCACATCAGCCCCATGCAATGACCGCCCACCTTGGGGTT
AACTTGTGGTTACAATTCTCTGTAACCTAGTAAAATCAAAACTCTAGAAGCTTATAATTAATCAGCCAACT
TTATACGT
>DB7DT8Q1:306:HBCKVADXX:2:2112:3987:25822_1:N:0:GGCTACAT/1
AGAGTGTCGGTGTCTTTGGCACTCTTTACACTCAATTTTTTAAATATTGATGTTCATGGCCATACTGCGGC
TGAACGTCATTGTTCAGAGCCAGATATGCC
>DB7DT8Q1:306:HBCKVADXX:2:2215:9654:89406_1:N:0:GGCTACAT/1
AACTCTCGCCCCTTGCATGGTTAAAACTGCAGTTCCCTCCCTCTTTCAGACAATAGCCAAAAGCCTAAGCC
TGGTTGCCCTCGCTCTCTTAACATTCTTTCTTTCTCTGTGTATCTCCCTCTCTGTGTCTCTGGGTGTATGT
CTGTGTCT
>DB7DT8Q1:306:HBCKVADXX:2:2115:3803:32267_1:N:0:GGCTACAT/1
ACTAGATGCCATAGGCAAACAAAAAATGGGTTACTTCTTTTGGAGTTGTTTGTCAGTGAAGTCCCAAAGTC
CCCAAATTGTGTGTTATTGCCACTGATACTTATTACCATCCAGAATTTTGTGATAATATACAGTTGTTGGA
AACACCAC
>DB7DT8Q1:306:HBCKVADXX:2:2115:14350:36515_1:N:0:AGCTACAT/1
TTACAATAAATGTATTTATTTTAAACTATTTGAAAGATCTTAACCCTGTTCTTAGCACTATAAAAATAAAT
GAATGACAAGCAGTGCGTAGCTTCTGCATAGAAAACATTGACTCCATCTTACACATTGTCCTTTGCTTTAC
AACTAAGA
>DB7DT8Q1:306:HBCKVADXX:2:2115:5085:80292_1:N:0:GGCTACAT/1
TCTGATAGGATGCATGGAACAATTTCAATATTTTTGTATCTGTTGAGGCCAGTTTTGTGACCAATTATATG
GTCAATTTTGGAGAAGGTACCACGAGGTGCTGAGAAGAAGGTATATCCTTTTGTTTTAGGATAAAATGTTC
TGTAGATA
>DB7DT8Q1:306:HBCKVADXX:2:2116:18147:11441_1:N:0:GGCTACAT/1
CCAAAACTCCAGGAGGTGAGTCTCCGTGAAACTCTGCCTTGTGAGTGCTTCCAGGCACTTCACGTGCCAGC
GTGGCACTCCTGGAAGCGCAAATCCTGCTGAACTCAAGCAAGGAGTGACAAGATCTGCTCTATTCTCTTCT
TAACTAGT
>DB7DT8Q1:306:HBCKVADXX:2:2116:4330:24831_1:N:0:GGCTACAT/1
TACTGAAAATATTGAATAACAATATTCTAGAAAGCTGTAAGAGGATGTCCTAATGATCCAAGGAGAAGAAC
CAGTTGTGCAAAAAAAGCAACTTAATTGAATAATCATCCTACAAGTTCTACGCCTACCAAAAGCCGGACTG
CCTAGAAT
>DB7DT8Q1:306:HBCKVADXX:2:2116:7355:34788_1:N:0:AGCTACAT/1
GAGTAAGTCACTCCCAGGGACCTCAGCCATTCTGAACATGCTCCCAGGGTCATAGGCTGAGCAGAGAGAGC
TGGGACCCTGAGGGAAGACAACATAGGAGATAACACTGCAACCCTTAGCTCAGTGAGCTGACCACAGTGCC
TGCCCCTA
>DB7DT8Q1:306:HBCKVADXX:2:2116:13914:37747_1:N:0:GGCTACAT/1
CTTAGGATTGTATTATTTCCTTTAGAGTATACAATGAAACGTTCCTGTGTATATCAAATTCATAGTTGGTG
CTGACAATCTAAGTTTATTCTGATAGAGTGACTGCCAATTAAATGAAGAATGTAGAAAGTGGAACAAGAGG
GGAAAGAT
>DB7DT8Q1:306:HBCKVADXX:2:2116:17591:38065_1:N:0:GGCTACAT/1
TGTTAAATGGGATTTTAATGCGTCTAACAGTTTTAAGTGTTACCCAGGAAGGAGACTGCCGCTCATTTCTT
TCCTCCTCAATGTGGTCATTTGCTTTTCTTGATACCAGGCTGCATTCATTTAAACACAAAGTGTTGGCAGA
AATAAAGT
>DB7DT8Q1:306:HBCKVADXX:2:2116:10117:44860_1:N:0:GGCTACAT/1
ATCCTTACAGATGGATATTTGTAGAGGAAAGACGCATAAGTACACAGTGAGCGGACGCACAGCTTGTAGGA
CACACTTTGTACAAAAAGTGACACTGGCAAGGATGTCAAGTGGAAACAAATGATACTTCTATACTGCACAT
TGATTTCT

>DB7DT8Q1:306:HBCKVADXX:2:2116:5341:46518_1:N:0:GGCTACAT/1
AGAAAGATGAGGCCAAATGGATGAGTGTTTGCTTGGAATTAGAATATTTGTAAGAACTCTATCCAGAATCT
GCCTGTCATCTAGATTTTTCTCCGTTTTCTGCTTTGAGTTTGGGCATATGACCCATCAACCCACTCTGCCA
TTGCTTTC
>DB7DT8Q1:306:HBCKVADXX:2:2116:16530:56595_1:N:0:AGCTACAT/1
AAAGATTTCCAGAATCCAGAAAGGCCATAAGGAATAGGTAAGAGTGTCTCAATAGGACCAGTGAAGGAAAT
GGGAGTACTGAAAATACCCATAAAATACCCAATCCAGTGTCTGGTTAGTGGACTTCCAGTGTTAGGTCTTT
TCCATCCC
>DB7DT8Q1:306:HBCKVADXX:2:2116:19450:61769_1:N:0:TGCTACAT/1
CTCTGTCTATTTTTATTGGCTTTCTTTATGAATCAGAGCAGGTTAAAAATCCCTTAGATCTCTCTGCAAAA
ATGGTGATGACGGTCCTCACTTCACAAGGCTTCTGTAGTTTAAATGAGTAGACACAGTGCTCATATACATT
GGACTGAA
>DB7DT8Q1:306:HBCKVADXX:2:2116:15850:86550_1:N:0:GGCTACAT/1
AGATTCGCTCTTTTCATGCTATTCTCATTAATTTCCATTGAATTGTAATGTATCAGAGATACTTTGGGGAT
GTGTGAAAATTAACATGTATTCTAACACAAACCAACAAAGAAATTTGTGTGTAAGATCACTTCCTCTTAAC
TAAAGATT
>DB7DT8Q1:306:HBCKVADXX:2:1216:8622:63176_1:N:0:GGCTAGAT/1
AAGGCCTCTTGGGAAGTGCTCTGTCCCAGCACAGCAGCCCTGGCTGAGCACATCATGACCCTAACTGCATG
CTCAGAGGCAGTGAGCACACCAGTCAGTAGCTGTAGCTAGGACATC
>DB7DT8Q1:306:HBCKVADXX:2:2115:12407:59788_1:N:0:GGCTACAT/1
ATTTTAAAACATAAAGTGGGTTTTTAAAACATCTCTTCTTCACTGAGTATGTCCTATGTCCAGCTTTTGTT
CCAAGGATTTCATAGGTAGTAACTTACTGGAGTTCACAAAGCATTGCAAGGTGGTCACTGTCATCACAACA
GATCGGAA
>DB7DT8Q1:306:HBCKVADXX:2:2115:6634:68820_1:N:0:GGCTACAT/1
TGAGCAAAGGGGTCAAAGTCATTCACTGGTGAAAGAGGAAACATTTCTAGTCAGGTTCTAGAGCAGCAAGA
TAGAGATGTTGGCCCACTTACCATGCGTAAAATTAGCTTGGGGTAGATCAAGACAACACGGCAATGGTAGA
ACTATAGC
>DB7DT8Q1:306:HBCKVADXX:2:2110:12347:69510_1:N:0:AGCTACAT/1
TTTATAGGAAGCATTTGTGAATAAACAACAAGTATGAAATTAGGATGTACATATTACAGGTACAAGCTTAA
ATTATTGTTTAAACTATTTTGGCACACCTTTTTTTCTCCTACAATACTTTGATATTTGCCATTTGAGAGAA
AAATTTTA
>DB7DT8Q1:306:HBCKVADXX:2:2213:1701:67119_1:N:0:AGCTACAT/1
AGGGAAAAAGGTCAAGTAACATATAAAGGCAAGCCTATCAGAATTACACCAGATTTTTCACCAGAGACTAT
GAAAGCCAGAAGAGCCTGGACAGATGTTATACAGACACTAAGAGAACACAAATTCCAGCCCAGGCTACTAT
ACCCAGCC
>DB7DT8Q1:306:HBCKVADXX:2:2105:20783:89916_1:N:0:AGCTACAT/1
CCTGTTGTAATTATTGCTTGTATATACTAATCATTTGTATGTACAAAGCTTCTCCTCACACGGATACGATT
TCCAAAATGGGAGAGATGATCTTTCACACTATTTAGAATGTGCTGGCAATATAATATCCATTATTCTTGCA
AGTAAATA
>DB7DT8Q1:306:HBCKVADXX:2:2113:9272:82810_1:N:0:GACTACAT/1
GCTCCAAATTAAGTATTATAGTTTCAAAGTAGAATATAAAGACTGGAAGGTGCTTCCATAAACATCATGGC
AGCTCCAAAGACAAAAGGACCTTTGTTGTGGACAGCAGACATGTGATAGAGGAGGCAAAGAACAGCACTGA
AAAGTCAT
>DB7DT8Q1:306:HBCKVADXX:2:2103:15359:14832_1:N:0:GGCTACCT/1
ATGAACATAGTGGAGCATGTGTCCTTCTTACCCGTTGGGGCATCTTCTGGATATATGCCCAGGAGAGGTAT
TGCTGGATCCTCCGGTAGTACTATGTCCAATTTTCTGAGGAACCGCCAGACTGATTTCCAGAGTGGTTGTA
CAAGCCTG
>DB7DT8Q1:306:HBCKVADXX:2:2207:9441:17686_1:N:0:CGCTACAT/1
GCATGTGACTTTTAAAAGTCTACTGTTATCCTTTCCTTATTCCATGAAAATTTTTAGAGAAATTTCCAGAT
GGAAATTGGACACTATCAAATGAGTCATTACACAGCTCA
>DB7DT8Q1:306:HBCKVADXX:2:2116:13068:34185_1:N:0:GGCTACAT/1
TATGAATTAGCTCTTTTACATACAGCATTTATTGATTGAGAAACTGCCCCAAAAGAGCTACTGTGTGTTAG
TTCTGGTTTTTAATAAATTTATTAATCACAGCAGTCCCTTTGGATAGTATTGAGAGAACTCTACACCTGTT
TTGTGCA
>DB7DT8Q1:306:HBCKVADXX:2:2116:5637:38170_1:N:0:AGCTACAT/1
GTAGTTTTGAGCTGCTCTGTGGGTTCTGGGAATCCATATAAGAGCAAGCAGTAACTCCTGACTGCGGAGGG
CCTCTCCGGCTCCACACTTTCCTGATATCCGTGGATGTTTTCTGTTACTTTTGTTATGATCTGTCTTTGCT
GACAAT

>DB7DT8Q1:306:HBCKVADXX:2:2116:4364:48059_1:N:0:GGCTACCT/1
CGAGATTCTTTCTTTACTTTTCCTTGCCCAAGTTCATGCTGTCCAGCTAGTCTTGGGGACAGGCCTGCCCCT
GACGTGTGTACACCCTGTTGGCAGTCACGTCATTACAGAACCTGCAGTTACTGGCAGGATTTTGT
>DB7DT8Q1:306:HBCKVADXX:2:2112:6442:4866_1:N:0:GGCTACAT/1
ATCTGGCATTCTAAATTAATTCAACATATATAAGAAAGATCTTAACAATTAGCCAAGAAGACTTATACTCT
AAAGAATTCATTCAACCATAAAGTATGATTAATTTCACCATTGTTTTGACACATGGCAGTTCAAGTTCAAC
CTTGTTAC
>DB7DT8Q1:306:HBCKVADXX:2:2112:7581:13704_1:N:0:GTCTACAT/1
CTGACTCGCTAAATACCCTTGGCTGGTTGCTGCCACATCAGCCCCATGCAATGACCGCCCACCTTGGGGTT
AACTTGTGGTTACAATTCTCTGTAACCTAGTAAAATCAAAACTCTAGAAGCTTATAATTAATCAGCCAACT
TTATACGT
>DB7DT8Q1:306:HBCKVADXX:2:2112:3987:25822_1:N:0:GGCTACAT/1
AGAGTGTCGGTGTCTTTGGCACTCTTTACACTCAATTTTTAAATATTGATGTTCATGGCCATACTGCGGC
TGAACGTCATTGTTCAGAGCCAGATATGCC
>DB7DT8Q1:306:HBCKVADXX:2:2215:9654:89406_1:N:0:GGCTACAT/1
AACTCTCGCCCCTTGCATGGTTAAAACTGCAGTTCCCTCCCTCTTTCAGACAATAGCCAAAAGCCTAAGCC
TGGTTGCCCTCGCTCTCTTAACATTCTTTCTTTCTCTGTGTATCTCCCTCTCTGTGTCTCTGGGTGTATGT
CTGTGTCT
>DB7DT8Q1:306:HBCKVADXX:2:2115:3803:32267_1:N:0:GGCTACAT/1
ACTAGATGCCATAGGCAAACAAAAAATGGGTTACTTCTTTTGGAGTTGTTTGTCAGTGAAGTCCCAAAGTC
CCCAAATTGTGTGTTATTGCCACTGATACTTATTACCATCCAGAATTTTGTGATAATATACAGTTGTTGGA
AACACCAC
>DB7DT8Q1:306:HBCKVADXX:2:2115:14350:36515_1:N:0:AGCTACAT/1
TTACAATAAATGTATTTATTTTAAACTATTTGAAAGATCTTAACCCTGTTCTTAGCACTATAAAAATAAAT
GAATGACAAGCAGTGCGTAGCTTCTGCATAGAAAACATTGACTCCATCTTACACATTGTCCTTTGCTTTAC
AACTAAGA
>DB7DT8Q1:306:HBCKVADXX:2:2115:5085:80292_1:N:0:GGCTACAT/1
TCTGATAGGATGCATGGAACAATTTCAATATTTTTGTATCTGTTGAGGCCAGTTTTGTGACCAATTATATG
GTCAATTTTGGAGAAGGTACCACGAGGTGCTGAGAAGAAGGTATATCCTTTTGTTTTAGGATAAAATGTTC
TGTAGATA
>DB7DT8Q1:306:HBCKVADXX:2:2116:18147:11441_1:N:0:GGCTACAT/1
CCAAAACTCCAGGAGGTGAGTCTCCGTGAAACTCTGCCTTGTGAGTGCTTCCAGGCACTTCACGTGCCAGC
GTGGCACTCCTGGAAGCGCAAATCCTGCTGAACTCAAGCAAGGAGTGACAAGATCTGCTCTATTCTCTTCT
TAACTAGT
>DB7DT8Q1:306:HBCKVADXX:2:2116:4330:24831_1:N:0:GGCTACAT/1
TACTGAAAATATTGAATAACAATATTCTAGAAAGCTGTAAGAGGATGTCCTAATGATCCAAGGAGAAGAAC
CAGTTGTGCAAAAAAAGCAACTTAATTGAATAATCATCCTACAAGTTCTACGCCTACCAAAAGCCGGACTG
CCTAGAAT
>DB7DT8Q1:306:HBCKVADXX:2:2116:7355:34788_1:N:0:AGCTACAT/1
GAGTAAGTCACTCCCAGGGACCTCAGCCATTCTGAACATGCTCCCAGGGTCATAGGCTGAGCAGAGAGAGC
TGGGACCCTGAGGGAAGACAACATAGGAGATAACACTGCAACCCTTAGCTCAGTGAGCTGACCACAGTGCC
TGCCCCTA
>DB7DT8Q1:306:HBCKVADXX:2:2116:13914:37747_1:N:0:GGCTACAT/1
CTTAGGATTGTATTATTTCCTTTAGAGTATACAATGAAACGTTCCTGTGTATATCAAATTCATAGTTGGTG
CTGACAATCTAAGTTTATTCTGATAGAGTGACTGCCAATTAAATGAAGAATGTAGAAAGTGGAACAAGAGG
GGAAAGAT
>DB7DT8Q1:306:HBCKVADXX:2:2116:17591:38065_1:N:0:GGCTACAT/1
TGTTAAATGGGATTTTAATGCGTCTAACAGTTTTAAGTGTTACCCAGGAAGGAGACTGCCGCTCATTTCTT
TCCTCCTCAATGTGGTCATTTGCTTTTCTTGATACCAGGCTGCATTCATTTAAACACAAAGTGTTGGCAGA
AATAAAGT
>DB7DT8Q1:306:HBCKVADXX:2:2116:10117:44860_1:N:0:GGCTACAT/1
ATCCTTACAGATGGATATTTGTAGAGGAAAGACGCATAAGTACACAGTGAGCGGACGCACAGCTTGTAGGA
CACACTTTGTACAAAAAGTGACACTGGCAAGGATGTCAAGTGGAAACAAATGATACTTCTATACTGCACAT
TGATTTCT
>DB7DT8Q1:306:HBCKVADXX:2:2116:5341:46518_1:N:0:GGCTACAT/1
AGAAAGATGAGGCCAAATGGATGAGTGTTTGCTTGGAATTAGAATATTTGTAAGAACTCTATCCAGAATCT
GCCTGTCATCTAGATTTTTCTCCGTTTTCTGCTTTGAGTTTGGGCATATGACCCATCAACCCACTCTGCCA
TTGCTTTC

>DB7DT8Q1:306:HBCKVADXX:2:2116:16530:56595_1:N:0:AGCTACAT/1
AAAGATTTCCAGAATCCAGAAAGGCCATAAGGAATAGGTAAGAGTGTCTCAATAGGACCAGTGAAGGAAAT
GGGAGTACTGAAAATACCCATAAAATACCCAATCCAGTGTCTGGTTAGTGGACTTCCAGTGTTAGGTCTTT
TCCATCCC
>DB7DT8Q1:306:HBCKVADXX:2:2116:19450:61769_1:N:0:TGCTACAT/1
CTCTGTCTATTTTTATTGGCTTTCTTTATGAATCAGAGCAGGTTAAAAATCCCTTAGATCTCTCTGCAAAA
ATGGTGATGACGGTCCTCACTTCACAAGGCTTCTGTAGTTTAAATGAGTAGACACAGTGCTCATATACATT
GGACTGAA
>DB7DT8Q1:306:HBCKVADXX:2:2116:15850:86550_1:N:0:GGCTACAT/1
AGATTCGCTCTTTTCATGCTATTCTCATTAATTTCCATTGAATTGTAATGTATCAGAGATACTTTGGGGAT
GTGTGAAAATTAACATGTATTCTAACACAAACCAACAAAGAAATTTGTGTGTAAGATCACTTCCTCTTAAC
TAAAGATT
>DB7DT8Q1:306:HBCKVADXX:2:1216:8622:63176_1:N:0:GGCTAGAT/1
AAGGCCTCTTGGGAAGTGCTCTGTCCCAGCACAGCAGCCCTGGCTGAGCACATCATGACCCTAACTGCATG
CTCAGAGGCAGTGAGCACACCAGTCAGTAGCTGTAGCTAGGACATC
>DB7DT8Q1:306:HBCKVADXX:2:2115:12407:59788_1:N:0:GGCTACAT/1
ATTTTAAAACATAAAGTGGGTTTTTAAAACATCTCTTCTTCACTGAGTATGTCCTATGTCCAGCTTTTGTT
CCAAGGATTTCATAGGTAGTAACTTACTGGAGTTCACAAAGCATTGCAAGGTGGTCACTGTCATCACAACA
GATCGGAA
>DB7DT8Q1:306:HBCKVADXX:2:2115:6634:68820_1:N:0:GGCTACAT/1
TGAGCAAAGGGGTCAAAGTCATTCACTGGTGAAAGAGGAAACATTTCTAGTCAGGTTCTAGAGCAGCAAGA
TAGAGATGTTGGCCCACTTACCATGCGTAAAATTAGCTTGGGGTAGATCAAGACAACACGGCAATGGTAGA
ACTATAGC
>DB7DT8Q1:306:HBCKVADXX:2:2110:12347:69510_1:N:0:AGCTACAT/1
TTTATAGGAAGCATTTGTGAATAAACAACAAGTATGAAATTAGGATGTACATATTACAGGTACAAGCTTAA
ATTATTGTTTAAACTATTTTGGCACACCTTTTTTTCTCCTACAATACTTTGATATTTGCCATTTGAGAGAA
AAATTTTA
>DB7DT8Q1:306:HBCKVADXX:2:2213:1701:67119_1:N:0:AGCTACAT/1
AGGGAAAAAGGTCAAGTAACATATAAAGGCAAGCCTATCAGAATTACACCAGATTTTTCACCAGAGACTAT
GAAAGCCAGAAGAGCCTGGACAGATGTTATACAGACACTAAGAGAACACAAATTCCAGCCCAGGCTACTAT
ACCCAGCC
>DB7DT8Q1:306:HBCKVADXX:2:2105:20783:89916_1:N:0:AGCTACAT/1
CCTGTTGTAATTATTGCTTGTATATACTAATCATTTGTATGTACAAAGCTTCTCCTCACACGGATACGATT
TCCAAAATGGGAGAGATGATCTTTCACACTATTTAGAATGTGCTGGCAATATAATATCCATTATTCTTGCA
AGTAAATA
>DB7DT8Q1:306:HBCKVADXX:2:2113:9272:82810_1:N:0:GACTACAT/1
GCTCCAAATTAAGTATTATAGTTTCAAAGTAGAATATAAAGACTGGAAGGTGCTTCCATAAACATCATGGC
AGCTCCAAAGACAAAAGGACCTTTGTTGTGGACAGCAGACATGTGATAGAGGAGGCAAAGAACAGCACTGA
AAAGTCAT
>DB7DT8Q1:306:HBCKVADXX:2:2103:15359:14832_1:N:0:GGCTACCT/1
ATGAACATAGTGGAGCATGTGTCCTTCTTACCCGTTGGGGCATCTTCTGGATATATGCCCAGGAGAGGTAT
TGCTGGATCCTCCGGTAGTACTATGTCCAATTTTCTGAGGAACCGCCAGACTGATTTCCAGAGTGGTTGTA
CAAGCCTG
>DB7DT8Q1:306:HBCKVADXX:2:2207:9441:17686_1:N:0:CGCTACAT/1
GCATGTGACTTTTAAAAGTCTACTGTTATCCTTTCCTTATTCCATGAAAATTTTTAGAGAAATTTCCAGAT
GGAAATTGGACACTATCAAATGAGTCATTACACAGCTCA
>DB7DT8Q1:306:HBCKVADXX:2:2116:13068:34185_1:N:0:GGCTACAT/1
TATGAATTAGCTCTTTTACATACAGCATTTATTGATTGAGAAACTGCCCCAAAAGAGCTACTGTGTGTTAG
TTCTGGTTTTTAATAAATTTATTAATCACAGCAGTCCCTTTGGATAGTATTGAGAGAACTCTACACCTGTT
TTGTGCA
>DB7DT8Q1:306:HBCKVADXX:2:2116:5637:38170_1:N:0:AGCTACAT/1
GTAGTTTTGAGCTGCTCTGTGGGTTCTGGGAATCCATATAAGAGCAAGCAGTAACTCCTGACTGCGGAGGG
CCTCTCCGGCTCCACACTTTCCTGATATCCGTGGATGTTTTCTGTTACTTTTGTTATGATCTGTCTTTGCT
GACAAT
>DB7DT8Q1:306:HBCKVADXX:2:2116:4364:48059_1:N:0:GGCTACCT/1
CGAGATTCTTTCTTTACTTTTCCTTGCCCAAGTTCATGCTGTCCAGCTAGTCTTGGGGACAGGCCTGCCCT
GACGTGTGTACACCCTGTTGGCAGTCACGTCATTACAGAACCTGCAGTTACTGGCAGGATTTTGT

Bangkok (Dog)

TTTTGCAGGCCATCACTAAAGGAGTTCAGCACCTTCTTGCCATGGGCCTTCACTTTAGCATTGCTCATAAC
AACATCAGGAGTGGACAGGTCCCCAAAGGAGTCAAAGAACCTCTGAGTCCAGGGGTAGACAATCAGCAGCC
TGCCCAGG

Canis lupus familiaris GLNA2 gene for globin A2

Sequence ID: LT548211.1

Identities 148/150(99%) Gaps 0/150(0%)

```
Query  1    TTTTGCAGGCCATCACTAAAGGAGTTCAGCACCTTCTTGCCATGGGCCTTCACTTTAGCA  60
            |||| |||||||||||||||||||||||||||||||||||||||||||||||||||||||
Sbjct  233  TTTTTCAGGCCATCACTAAAGGAGTTCAGCACCTTCTTGCCATGGGCCTTCACTTTAGCA  174

Query  61   TTGCTCATAACAACATCAGGAGTGGACAGGTCCCCAAAGGAGTCAAAGAACCTCTGAGTC  120
            |||||||||||| |||||||||||||||||||||||||||||||||||||||||||||||
Sbjct  173  TTGCTCATAACAGCATCAGGAGTGGACAGGTCCCCAAAGGAGTCAAAGAACCTCTGAGTC  114

Query  121  CAGGGGTAGACAATCAGCAGCCTGCCCAGG  150
            ||||||||||||||||||||||||||||||
Sbjct  113  CAGGGGTAGACAATCAGCAGCCTGCCCAGG  84
```

Homo sapiens hemoglobin subunit beta (HBB), mRNA

Sequence ID: NM_000518.4

Identities 127/145(88%) Gaps 0/145(0%)

```
Query  6    CAGGCCATCACTAAAGGAGTTCAGCACCTTCTTGCCATGGGCCTTCACTTTAGCATTGCT  65
            |||||||||||||||||     ||||| ||||||||||| |||||||| ||||  |||| 
Sbjct  278  CAGGCCATCACTAAAGGCACCGAGCACTTTCTTGCCATGAGCCTTCACCTTAGGGTTGCC  219

Query  66   CATAACAACATCAGGAGTGGACAGGTCCCCAAAGGAGTCAAAGAACCTCTGAGTCCAGGG  125
            ||||||| |||||||||||||||| ||||||||||| |||||||||||||| ||||| ||
Sbjct  218  CATAACAGCATCAGGAGTGGACAGATCCCCAAAGGACTCAAAGAACCTCTGGGTCCAAGG  159

Query  126  GTAGACAATCAGCAGCCTGCCCAGG  150
            |||||| | ||||||||||||||||
Sbjct  158  GTAGACCACCAGCAGCCTGCCCAGG  134
```

CATACTGTTGCATTCATGCCAACCCTACTTACAGACATCTCAAAACTAGCAGTAATTAAGTTAAACGGTCC
CCCCAAATCCCACAATTCAAGCTAAACTCGAAGTTAACAGCTACAGGAGTGGCACCTACACGTTAATGCTA
GCTGAAGCACAGGCAGCGGGGGGACGTTTCATCCCGCTCTCCCAAGCACAGGACACTGG

PREDICTED: Canis lupus familiaris ornithine decarboxylase 1 (ODC1), mRNA

Sequence ID:XM_851618.5

Identities 201/201(100%) Gaps 0/201(0%)

```
Query  1     CATACTGTTGCATTCATGCCAACCCTACTTACAGACATCTCAAAACTAGCAGTAATTAAG  60
             ||||||||||||||||||||||||||||||||||||||||||||||||||||||||||||
Sbjct  1850  CATACTGTTGCATTCATGCCAACCCTACTTACAGACATCTCAAAACTAGCAGTAATTAAG  1791

Query  61    TTAAACGGTCCCCCCAAATCCCACAATTCAAGCTAAACTCGAAGTTAACAGCTACAGGAG  120
             ||||||||||||||||||||||||||||||||||||||||||||||||||||||||||||
Sbjct  1790  TTAAACGGTCCCCCCAAATCCCACAATTCAAGCTAAACTCGAAGTTAACAGCTACAGGAG  1731

Query  121   TGGCACCTACACGTTAATGCTAGCTGAAGCACAGGCAGCGGGGGGACGTTTCATCCCGCT  180
             ||||||||||||||||||||||||||||||||||||||||||||||||||||||||||||
Sbjct  1730  TGGCACCTACACGTTAATGCTAGCTGAAGCACAGGCAGCGGGGGGACGTTTCATCCCGCT  1671

Query  181   CTCCCAAGCACAGGACACTGG  201
             |||||||||||||||||||||
Sbjct  1670  CTCCCAAGCACAGGACACTGG  1650
```

Homo sapiens ornithine decarboxylase 1 (ODC1), transcript variant 4, mRNA

Sequence ID: NM_001287190.1

Identities: 170/205(83%) Gaps 5/205(2%)

```
Query  1     CATACTGTTGCATTCATGCCAACCCTACT--TACA--GACATCTCAAAACTAGCAGTAAT  56
             ||||  | ||||| |||| | ||||||||  ||||  ||||| |||||||||||||||||
Sbjct  1814  CATATGGCTGCAT-CATGGCGACCCTACTCTTACAAAGACATTTCAAAACTAGCAGTAAT  1756

Query  57    TAAGTTAAACGGTCCCCCCAAATCCCACAATTCAAGCTAAACTCGAAGTTAACAGCTACA  116
             ||||||| | ||||||||||||||||  ||||||||||||||| | ||||||||||||| 
Sbjct  1755  TAAGTTACATGGTCCCCCCAAATCCCTTAATTCAAGCTAAACTTGCAGTTAACAGCTACC  1696

Query  117   GGAGTGGCACCTACACGTTAATGCTAGCTGAAGCACAGGCAGCGGGGGGACGTTTCATCC  176
              |||||  | |||||| ||||| ||||| ||||||||||| ||   | | ||||||||||
Sbjct  1695  AGAGTGCTATCTACACATTAATACTAGCCGAAGCACAGGCTGCTCTGTGGCGTTTCATCC  1636
```

```
Query  177   CGCTCTCCCAAGCACAGGACACTGG  201
             | |||||||| ||||| ||||| ||
Sbjct  1635  CACTCTCCCAGGCACAAGACACAGG  1611
```

ATATCACTGAGCTGAACAAATAAGTCACCTCTGAGTACATAAGGAAAAATAAACACATGCTAAATGGATAA
AACATTACAAACTACATCTTTACTAAACCATCTATACCTCTAACACCTCTAACACTTCAGAAGCTTAGAGA
TTCA

PREDICTED: Canis lupus familiaris ring finger protein 11 (RNF11), transcript variant X4, mRNA

Sequence ID: XM_005629010.3

Identities: 146/146 (100%) Gaps 0/146(0%)

```
Query  1     ATATCACTGAGCTGAACAAATAAGTCACCTCTGAGTACATAAGGAAAAATAAACACATGC  60
             ||||||||||||||||||||||||||||||||||||||||||||||||||||||||||||
Sbjct  1774  ATATCACTGAGCTGAACAAATAAGTCACCTCTGAGTACATAAGGAAAAATAAACACATGC  1715

Query  61    TAAATGGATAAAACATTACAAACTACATCTTTACTAAACCATCTATACCTCTAACACCTC  120
             ||||||||||||||||||||||||||||||||||||||||||||||||||||||||||||
Sbjct  1714  TAAATGGATAAAACATTACAAACTACATCTTTACTAAACCATCTATACCTCTAACACCTC  1655

Query  121   TAACACTTCAGAAGCTTAGAGATTCA  146
             ||||||||||||||||||||||||||
Sbjct  1654  TAACACTTCAGAAGCTTAGAGATTCA  1629
```

Homo sapiens ring finger protein 11 (RNF11), mRNA

Sequence ID: NM_014372.4

Identitites: 126/144(88%) Gaps: 10/144(6%)

```
Query  1     ATATCACTGAGCTGAACAAATAAGTCACCTCTGAGTACATAAGGAAAAATAAACACATGC  60
             ||||||||||| ||||| |||||||||||| |||||||||| | ||||||||||||||||
Sbjct  2339  ATATCACTGAGGTGAACCAATAAGTCACCTTTGAGTACATATGAAAAAATAAACACATGC  2280

Query  61    TAAATGGATAAAACATTACAAACTACATCTTTACTAAACCATCTATACCTCTAACACCTC  120
             |||||||||||||||||||  ||||||||||||||| ||||||| |         |||||
Sbjct  2279  TAAATGGATAAAACATTAC-TACTACATCTTTACTAGACCATCTCT---------ACCTC  2230

Query  121   TAACACTTCAGAAGCTTAGAGATT  144
             ||||||||||||||||||||||||
Sbjct  2229  TAACACTTCAGAAGCTTAGAGATT  2206
```

CTCAATGGTACTTGTGAGCCAGGGCATTGGCCACACCAGCCACCACCTTCTGATAGGCAGCCTGCACCTGA
GGGGTGAATTCTTTGCCAAAGTGGTGAGCCAGCACACACACAAGCACGTTGCCCAGGAGCTTGAAATTCTC
GGGATCCACGTGCAGCTTGTCACAGTGAAGCTCACTGAGCTTAGCAAAGGTGCCCTTGAGG

Canis lupus familiaris hemoglobin subunit beta-like (LOC480784), mRNA

Sequence ID: NM_001270884.1

Identities: 202/203(99%) Gaps:0/203(0%)

```
Query  1    CTCAATGGTACTTGTGAGCCAGGGCATTGGCCACACCAGCCACCACCTTCTGATAGGCAG  60
            ||||||||||||||||||||||||||||||||||||||||||||||||||||||||||||
Sbjct  513  CTCAATGGTACTTGTGAGCCAGGGCATTGGCCACACCAGCCACCACCTTCTGATAGGCAG  454

Query  61   CCTGCACCTGAGGGGTGAATTCTTTGCCAAAGTGGTGAGCCAGCACACACACAAGCACGT  120
            ||||||||||||||||||||||||||||||||||||||||||||||||||||||||||||
Sbjct  453  CCTGCACCTGAGGGGTGAATTCTTTGCCAAAGTGGTGAGCCAGCACACACACAAGCACGT  394

Query  121  TGCCCAGGAGCTTGAAATTCTCGGGATCCACGTGCAGCTTGTCACAGTGAAGCTCACTGA  180
            |||||||||||||||| |||||||||||||||||||||||||||||||||||||||||||
Sbjct  393  TGCCCAGGAGCTTGAAGTTCTCGGGATCCACGTGCAGCTTGTCACAGTGAAGCTCACTGA  334

Query  181  GCTTAGCAAAGGTGCCCTTGAGG  203
            |||||||||||||||||||||||
Sbjct  333  GCTTAGCAAAGGTGCCCTTGAGG  311
```

Homo sapiens hemoglobin subunit delta (HBD), mRNA

Sequence ID: NM_000519.3

Identities:184/203 (91%) Gaps: 0/203(0%)

```
Query  1    CTCAATGGTACTTGTGAGCCAGGGCATTGGCCACACCAGCCACCACCTTCTGATAGGCAG  60
            |||||||||||||||||||||||||||| |||||||||||||||||||||||||||||||
Sbjct  640  CTCAATGGTACTTGTGAGCCAGGGCATTAGCCACACCAGCCACCACCTTCTGATAGGCAG  581

Query  61   CCTGCACCTGAGGGGTGAATTCTTTGCCAAAGTGGTGAGCCAGCACACACACAAGCACGT  120
```

```
               ||||||  || ||||||||||| |||||||||| | | |||||||||||||| ||||| |
Sbjct  580   CCTGCATTTGTGGGGTGAATTCCTTGCCAAAGTTGCGGGCCAGCACACACACCAGCACAT  521

Query  121   TGCCCAGGAGCTTGAAATTCTCGGGATCCACGTGCAGCTTGTCACAGTGAAGCTCACTGA  180
             |||||| |||| |||| ||||| |||||||||||||||||||||||||| |||||||| |
Sbjct  520   TGCCCAAGAGCCTGAAGTTCTCAGGATCCACGTGCAGCTTGTCACAGTGCAGCTCACTCA  461

Query  181   GCTTAGCAAAGGTGCCCTTGAGG  203
             ||| || ||| ||||||||||||
Sbjct  460   GCTGAGAAAAAGTGCCCTTGAGG  438
```

CCGCATGATCTCTGCAGCTTCTCTTTCGGTGAAAGCCAGGTCACCACGCTCCTGAATCCGGCTGAATAGCT
CACCACCTTCCATGCATTCCATGACGATGAGGAGACAGCGCTTGCTGTGATGCATGTTCTCATACACGTCC
AGGATGCGCACAATGTGAGGGCCACCCGAGGCCTGCCAGTGGTGGTCCACCTCCTGCCGGGCCTTGGGGCT
GTCGTACAGGAGCTTCAGGGCACATTTCTG

PREDICTED: Canis lupus familiaris mitogen-activated protein kinase-activated protein kinase 3 (MAPKAPK3), mRNA
Sequence ID: XM_541872.5
Identities: 243/243(100%) Gaps: 0/243(0%)

```
Query  1     CCGCATGATCTCTGCAGCTTCTCTTTCGGTGAAAGCCAGGTCACCACGCTCCTGAATCCG  60
             ||||||||||||||||||||||||||||||||||||||||||||||||||||||||||||
Sbjct  693   CCGCATGATCTCTGCAGCTTCTCTTTCGGTGAAAGCCAGGTCACCACGCTCCTGAATCCG  634

Query  61    GCTGAATAGCTCACCACCTTCCATGCATTCCATGACGATGAGGAGACAGCGCTTGCTGTG  120
             ||||||||||||||||||||||||||||||||||||||||||||||||||||||||||||
Sbjct  633   GCTGAATAGCTCACCACCTTCCATGCATTCCATGACGATGAGGAGACAGCGCTTGCTGTG  574

Query  121   ATGCATGTTCTCATACACGTCCAGGATGCGCACAATGTGAGGGCCACCCGAGGCCTGCCA  180
             ||||||||||||||||||||||||||||||||||||||||||||||||||||||||||||
Sbjct  573   ATGCATGTTCTCATACACGTCCAGGATGCGCACAATGTGAGGGCCACCCGAGGCCTGCCA  514

Query  181   GTGGTGGTCCACCTCCTGCCGGGCCTTGGGGCTGTCGTACAGGAGCTTCAGGGCACATTT  240
             ||||||||||||||||||||||||||||||||||||||||||||||||||||||||||||
Sbjct  513   GTGGTGGTCCACCTCCTGCCGGGCCTTGGGGCTGTCGTACAGGAGCTTCAGGGCACATTT  454

Query  241   CTG  243
             |||
Sbjct  453   CTG  451
```

PREDICTED: Homo sapiens mitogen-activated protein kinase-activated protein kinase 3 (MAPKAPK3), transcript variant X1, misc_RNA
Sequence ID: XR_940501.1
Identities: 219/243(90%) Gaps: 0/243(0%)

```
Query  1     CCGCATGATCTCTGCAGCTTCTCTTTCGGTGAAAGCCAGGTCACCACGCTCCTGAATCCG  60
             |||||| ||||||||||||||||| || ||||||||| |||| ||||||||||||||||
Sbjct  566   CCGCATTATCTCTGCAGCTTCTCTCTCAGTGAAAGCCTGGTCGCCACGCTCCTGAATCCT  507

Query  61    GCTGAATAGCTCACCACCTTCCATGCATTCCATGACGATGAGGAGACAGCGCTTGCTGTG  120
             |||||| | |||||||||||||||||||||||||| ||||||||||||||||||||  ||
Sbjct  506   GCTGAACAACTCACCACCTTCCATGCATTCCATGATGATGAGGAGACAGCGCTTGCCATG  447

Query  121   ATGCATGTTCTCATACACGTCCAGGATGCGCACAATGTGAGGGCCACCCGAGGCCTGCCA  180
              ||||||||||||||||| ||||||||||  ||||| || ||||| || || ||||||||
Sbjct  446   GTGCATGTTCTCATACACATCCAGGATGCAGACAATATGGGGGCCGCCAGAAGCCTGCCA  387

Query  181   GTGGTGGTCCACCTCCTGCCGGGCCTTGGGGCTGTCGTACAGGAGCTTCAGGGCACATTT  240
             ||| ||||| |||||||||||||||||||||||||| |||||||||||||||||||| ||
Sbjct  386   GTGATGGTCTACCTCCTGCCGGGCCTTGGGGCTGTCATACAGGAGCTTCAGGGCACACTT  327

Query  241   CTG  243
             |||
Sbjct  326   CTG  324
```

Cairns (Cow)

CCCTCCCAGCATCAGGGTCTTTTCCAATGAGTCAACTCTTCGCATGAGATGGCCAAAGTATTGGAGTTTCA
GCTTCAGCATCAGTCCTTCCAATGAACACCCAGGATTGGTCTCCTTTAGGATGGACTGCTTGGATCTCCTT
GCAGTCCAAGGG

PREDICTED: Bos indicus angiotensin II receptor type 1 (AGTR1), mRNA
Sequence ID: XM_019961143

Identities 149/154(97%) Gaps 0/154(0%)

```
Query  1     CCCTCCCAGCATCAGGGTCTTTTCCAATGAGTCAACTCTTCGCATGAGATGGCCAAAGTA  60
             ||||| |||||||||||||||||||||||||||||||||||||||||| |||||||||||
Sbjct  2063  CCCTCTCAGCATCAGGGTCTTTTCCAATGAGTCAACTCTTCGCATGAGGTGGCCAAAGTA  2122

Query  61    TTGGAGTTTCAGCTTCAGCATCAGTCCTTCCAATGAACACCCAGGATTGGTCTCCTTTAG  120
             |||||||||||||||||||||||||||||||||||||||||||||| || ||||||||||
Sbjct  2123  TTGGAGTTTCAGCTTCAGCATCAGTCCTTCCAATGAACACCCAGGACTGATCTCCTTTAG  2182

Query  121   GATGGACTGCTTGGATCTCCTTGCAGTCCAAGGG  154
             ||||||||| ||||||||||||||||||||||||
Sbjct  2183  GATGGACTGGTTGGATCTCCTTGCAGTCCAAGGG  2216
```

Homo sapiens angiotensinogen (AGT), mRNA
Sequence ID: NM_000029.3
Identities 25/26(96%) Gaps 0/26(0%)

```
Query  84    GTCCTTCCAATGAACACCCAGGATTG  109
             |||||||||| |||||||||||||||
Sbjct  1012  GTCCTTCCAAGGAACACCCAGGATTG  987
```

Bangkok (Cow)

GGCTGGATGGCATCACTGACTCAATGGACATGAGTCTGAGTGAACTCCGGGAGTTGGTGATGGACAGGGAG
GCCTGTCGTGCTGCGATTCATGGGGTTGCAAAGAGTCGG

Bos taurus Y Chr BAC CH240-49F14 (Children's Hospital Oakland Research Institute Bovine BAC Library (male)) complete sequence
Sequence ID: AC226423.5
Identities 107/110 (97%) Gaps 0/110 (0%)

```
Query  1     GGCTGGATGGCATCACTGACTCAATGGACATGAGTCTGAGTGAACTCCGGGAGTTGGTGA  60
             ||||||||||||||||||||||||||||| ||||||||||||||||||||||||||||||
Sbjct  5350  GGCTGGATGGCATCACTGACTCAATGGACGTGAGTCTGAGTGAACTCCGGGAGTTGGTGA  5409

Query  61    TGGACAGGGAGGCCTGTCGTGCTGCGATTCATGGGGTTGCAAAGAGTCGG  110
             |||||||||||||||| ||||||| |||||||||||||||||||||||||
Sbjct  5410  TGGACAGGGAGGCCTGGCGTGCTGTGATTCATGGGGTTGCAAAGAGTCGG  5459
```

Homo sapiens chromosome 15, alternate assembly CHM1_1.1
Sequence ID: NC_018926.2
Identitites 27/30(90%) Gaps 1/30(3%)

```
Query  42        GAACTCCGGGAGTTGGTGATGGACAGGGAG  71
                 |||||| || |||| |||||||||||||||
Sbjct  78558460  GAACTCAGG-AGTTTGTGATGGACAGGGAG  78558488
```
